# Peptidylarginine deiminase 2 citrullinates MZB1 and promotes the secretion of IgM and IgA

**DOI:** 10.3389/fimmu.2023.1290585

**Published:** 2023-11-29

**Authors:** Benjamin Geary, Bo Sun, Ronak R. Tilvawala, Leonard Barasa, Konstantin Tsoyi, Ivan O. Rosas, Paul R. Thompson, I-Cheng Ho

**Affiliations:** ^1^ Division of Rheumatology, Inflammation, and Immunity, Department of Medicine, Brigham and Women’s Hospital, Boston, MA, United States; ^2^ Department of Medicine, Harvard Medical School, Boston, MA, United States; ^3^ Department of Biochemistry and Molecular Pharmacology, University of Massachusetts Medical School, Worcester, MA, United States; ^4^ Pulmonary, Critical Care and Sleep Medicine Section, Baylor College of Medicine, Houston, TX, United States

**Keywords:** citrullination, MZB1, PAD2, deiminase, immunoglobulin M, immunoglobulin A, post-translational modification

## Abstract

**Introduction:**

MZB1 is an endoplasmic reticulum residential protein preferentially expressed in plasma cells, marginal zone and B1 B cells. Recent studies on murine B cells show that it interacts with the tail piece of IgM and IgA heavy chain and promotes the secretion of these two classes of immunoglobulin. However, its role in primary human B cells has yet to be determined and how its function is regulated is still unknown. The conversion of peptidylarginine to peptidylcitrulline, also known as citrullination, by peptidylarginine deiminases (PADs) can critically influence the function of proteins in immune cells, such as neutrophils and T cells; however, the role of PADs in B cells remains to be elucidated.

**Method:**

An unbiased analysis of human lung citrullinome was conducted to identify citrullinated proteins that are enriched in several chronic lung diseases, including rheumatoid arthritis-associated interstitial lung disease (RA-ILD), chronic obstructive pulmonary disease, and idiopathic pulmonary fibrosis, compared to healthy controls. Mass spectrometry, site-specific mutagenesis, and western blotting were used to confirm the citrullination of candidate proteins. Their citrullination was suppressed by pharmacological inhibition or genetic ablation of PAD2 and the impact of their citrullination on the function and differentiation of human B cells was examined with enzyme-linked immunosorbent assay, flow cytometry, and co-immunoprecipitation.

**Results:**

Citrullinated MZB1 was preferentially enriched in RA-ILD but not in other chronic lung diseases. MZB1 was a substrate of PAD2 and was citrullinated during the differentiation of human plasmablasts. Ablation or pharmacological inhibition of PAD2 in primary human B cells attenuated the secretion of IgM and IgA but not IgG or the differentiation of IgM or IgA-expressing plasmablasts, recapitulating the effect of ablating MZB1. Furthermore, the physical interaction between endogenous MZB1 and IgM/IgA was attenuated by pharmacological inhibition of PAD2.

**Discussion:**

Our data confirm the function of MZB1 in primary human plasmablasts and suggest that PAD2 promotes IgM/IgA secretion by citrullinating MZB1, thereby contributing to the pathogenesis of rheumatoid arthritis and RA-ILD.

## Introduction

Peptidylarginine deiminases (PADs), including PAD1-4, are responsible for a unique form of post-translational modification, namely peptidylarginine deimination or citrullination, through which positively charged peptidylarginine is converted to neutral non-coding peptidylcitrulline. PADs have been shown to regulate the function of both innate and adaptive immune cells. For example, PAD4 can citrullinate histones and facilitate chromatin decondensation, and is essential for the formation of neutrophil extracellular traps in some experimental settings. We have demonstrated that PAD4-mediated citrullination of NF-kB p65 enhances its interaction with importin and subsequently its nuclear localization, thereby promoting LPS-induced expression of IL-1β and TNFα in neutrophils (1). In addition, citrullination of GATA3 and RORγt by PAD2 can alter the DNA binding of these two transcription factors and consequentially the differentiation of Th2 and Th17 cells (2). While many citrullinated peptides can serve as antigens for anti-citrullinated protein antibodies that are detected in patients with rheumatoid arthritis, an intrinsic role of PADs in B cells has yet to be established. In contrast to neutrophils and T cells, very few citrullinated proteins were detected with AMC or F95, two generic antibodies recognizing citrullinated proteins, in ionomycin-stimulated human primary B cells (3). Nevertheless, low levels of PAD transcripts have been detected in B cells and citrullination of human IgG at the Fc region has been reported (4). However, the impact of such citrullination on the function of IgG is unknown. It is also unclear whether such citrullination takes place inside B cells or extracellularly.

MZB1, a.k.a. pERp1 or CNPY5, belongs to the CONOPY family of ER chaperones (5). It is highly expressed in marginal zone B and B1 B cells as well as plasma cells (6–8). Several recent proteomic and transcriptomic studies have demonstrated that MZB1 is highly expressed in the lymph nodes from patients with lupus or RA (9), fibrotic lung and skin (10), iris tissue and aqueous humor form patients with juvenile idiopathic arthritis-associated uveitis (11), and the gingiva of patients with chronic periodontitis (12–14), a major risk factor of RA. In addition, the protein level of MZB1 in synovial tissue correlates with the histology score in RA (15). Loss of function analyses have convincingly shown that MZB1 is essential for optimal secretion of IgA and IgM (8, 16, 17), but not the differentiation of B cells, class switch, or expression of immunoglobulins. However, whether MZB1 has similar function in primary human B cells is unknown.

The mechanism of action of MZB1 is still not fully understood. Co-immunoprecipitation experiments suggest that it interacts with the tail piece of IgM and IgA (6, 17). While MZB1 does not interact directly with the J chain, its presence stabilizes the light chain/IgA heavy chain complex, facilitates the binding of the J chain to the tail piece, and subsequently promotes the secretion of these two classes of immunoglobulin, particularly the polymeric forms. This mechanism satisfactorily explains why the impact of MZB1 deficiency is more profound on the secretion of IgM/IgA than IgG (17). Interestingly, MZB1 was also co-immunoprecipitated with free light chains and its interaction with IgA heavy chain was more robust in the presence of light chain (6, 17), suggesting the presence of additional points of physical interaction between MZB1 and IgM/IgA. Surprisingly, *in vitro* biolayer interferometry was unable to show any interaction between MZB1, which was generated from *E. coli*, and the C-terminal Ig domain of IgA containing the tail piece (18). This discrepancy may suggest that MZB1 interacts with the IgA tail piece through another protein. This scenario is consistent with the observations that MZB1 interacts with several other ER proteins, including BiP, Grp94, and ERp57, in B cells (6, 8, 16). Alternatively, post-translational modifications of MZB1, which take place only in mammalian cells, may be critical for its interaction with the tail piece.

Here we report that MZB1 is preferentially enriched in the pool of citrullinated lung proteins harvested from patients with rheumatoid arthritis-associated interstitial lung disease and is a substrate of PAD2. Both MZB1 and PAD2 are required for the optimal secretion of IgM and IgA by primary human plasmablasts. In addition, inhibition of PAD2 attenuates the physical interaction between MZB1 and IgM/IgA. Our data confirm the function of MZB1 and identify a novel function of PAD2 in primary human plasmablasts, i.e. promoting IgM and IgA secretion by citrullinating MZB1.

## Materials and methods

### Human lungs and PBMC

Human lung samples were obtained with informed consent from lungs rejected for transplant according to approved Partners IRB (2011P002419). Peripheral blood of healthy donors was obtained through Massachusetts General Brigham Biobank. Peripheral blood mononuclear cells were isolated from whole human peripheral blood by Ficoll-Paque PLUS (17-1440-03, GE Healthcare) density gradient centrifugation.

### Mice

PAD2KO mice was previously described (19, 20) and have been backcrossed to C57BL/6 mice for 12 generations. All mouse work was approved by BWH IACUC (2016N000479).

### Real time PCR

RNA isolation, reverse transcription, and real time PCR were performed as previously described (21). Transcript level thus detected was normalized against that of actin or GAPDH. The sequence of the primers used is listed in **Supplementary Table 1**.

### Recombinant MZB1, site-specific mutagenesis, and *in vitro* citrullination

The full-length human MZB1 was cloned into pGEX-4T3 (GE Healthcare, Chicago, IL). The R-to-K mutants were generated with QuikChange XL Site-Directed Mutagenesis kit (Agilent Technologies, Lexington, MA) according to manufacturer’s instructions. The GST-MZB1 wild type and mutants were expressed in bacteria (BL21, EMD Millipore, Burlington, MA) as GST-fused proteins, and pre-bound to glutathione–agarose beads (#16100, Sigma-Aldrich, St. Louis, MO). The bound GST-MZB1 recombinant proteins were then incubated with purified recombinant PAD2 (20mM) in a buffer containing 100 mM HEPES, 100 mM NaCl, 10 mM CaCl2, 0.1 mM EDTA, and 2 mM DTT for 4 hrs at 37°C.

### ELISA

Sandwich ELISA was performed with the following antibodies: anti-mouse IgA (α chain specific, M1271, Sigma-Aldrich), biotin anti-mouse IgA (clone RMA-1, #407000 BioLegend, San Diego, CA), anti-mouse IgM (µ-chain specific, M8644-1MG, Sigma), biotin anti-mouse IgM (RMM-1, #406504, BioLegend), anti-mouse IgG (whole molecule, M8642, Sigma-Aldrich), biotin goat anti-mouse IgG (#405303, BioLegend), anti-human IgG (#AP112, EMD Millipore), biotin goat anti-human IgG (#AP112B, EMD Millipore), anti-human IgM (#3124502, BioLegend), biotin goat anti-human IgM (#314504, BioLegend). Human IgA ELISA was performed with IgA Human Uncoated ELISA Kit (#88-50600-88, Thermo Fisher, Waltham, MA).

### Western blotting

The following antibodies were used: anti-MZB1 (HPA043745, Sigma-Aldrich; PA5-80838, ThermoFisher), anti-alpha Tubulin (T6199-200IL, Sigma-Aldrich), anti-human IgM (I0759, Sigma-Aldrich), anti-human IgA (A18781, Invitrogen), anti-BiP (#3183S, Cell Signaling Technology), anti-modified citrulline (AMC) antibody (clone C4, MABS487, Sigma-Aldrich), and F95 antibody (MABN328, Sigma-Aldrich). Densitometry readings of western blots were obtained with ImageJ software.

### FACS

Differentiated human and mouse plasmablasts were stained with APC anti-human CD19 (#302212, BioLegend), PE/Cyanine7 anti-human CD27 (#303838, BioLegend), Pacific Blue™ anti-human CD38 (#356627, BioLegend), APC/Cyanine7 anti-human CD138 (#356527, BioLegend), PE anti-human IgM (#314508, BioLegend), FITC anti-human IgA (H14101, Life Technologies). Stained cells were collected with FACSCanto or LSRFortessa (Becton Dickenson, Franklin Lakes, NJ) and the data was analyzed with FlowJo software (Becton Dickinson).

### *In vitro* differentiation of mouse and human B cells

Murine B220^+^ cells were isolated from mouse spleen and lymph node lysate using CD45R(B220) Microbeads (#130-049-501, Miltenyi Biotec, North Rhine-Westphalia, Germany) and LS columns (#130-042-401, Miltenyi Biotec). Cells were cultured in RPMI complete media and plated in 12-well plates. LPS (25µg/mL) (#L2630, Sigma-Aldrich), IL-4 (10ng/mL) (#214-14, Peprotech, East Windsor, NJ), IL-5 (1.5ng/mL) (#581504, BioLegend), and TGF-β1 (2ng/mL) (# 240-B-010, R&D Systems, Minneapolis, MN) were added after plating. Human CD19^+^ cells were purified from PBMCs using CD19 Microbeads (#130-050-301, Miltenyi Biotec) and LS columns. Cells were cultured in IMDM and 10% FBS with 50µg/mL human transferrin (T3309, Sigma-Aldrich) and plated in 24-well plates. IL-2 (20U/mL) (NIH BRB), IL-10 (50ng/mL) (#571004, BioLegend), IL-15 (10ng/mL) (#570304, BioLegend), CpG ODN 2006 (10µg/mL) (ODN-2006, InvivoGen, San Diego, CA), human insulin (5 ug/ml) (Sigma #PHR8925) anti-polyhistidine tag antibody (5µg/mL) (MAB3834, Sigma-Aldrich), and histidine-tagged soluble CD40L (50ng/mL) (#32047, Cayman Chemical, Ann Arbor, MI), and AFM-30a (CAY10723, Cayman Chemical) were added after plating. In latter experiments, anti-human CD40 (#BE0189, BioXCell) 1 ug/ml was used to replace cross-linking of soluble CD40L. Differentiated B cells were analyzed 4 days later.

### CRISPR of human B cells

RNP complexes were assembled with Cas9-NLS purified protein from the QB3 MacroLab (UC Berkeley) and sgRNA from Synthego (Redwood City, CA). Cells were transfected with RNPs via nucleofection (pulse code EO-117, Lonza 4D Nucleofector™, Basel, Switzerland) using the P3 Primary Cell 4D- Nucleofector™ X Kit S (Cat. #V4XP-3032, Lonza). Cells were incubated in pre-warmed media for 2 hours prior to nucleofection. The sequence of the gRNAs is listed in **Supplementary Table 2**.

### Co-immunoprecipitation

Cells were washed in PBS, lysed in Immunoprecipitation Buffer (150 mM NaCl, 10 mM Tris-HCl, 1 mM EDTA, 1 mM EGTA, 2.5 mM PMSF, 1X cOmplete Mini Protease Inhibitor Cocktail (Sigma #11836153001), 1% Triton X-100, 0.5% NP-40) for 1 hour at 4°C, and centrifuged to pellet debris. The lysate was pre-cleared with Protein G Magnetic Beads (New England BioLabs #S1430S) and rabbit IgG (Cell Signaling Technology #2729S) for 1 hour at 4°C with agitation. Pre-cleared lysate was incubated with anti-MZB1 (Thermofisher #PA5-80838) or rabbit IgG for 1 hour at 4°C with agitation, then incubated with protein G beads for another 1 hour at 4°C with agitation. Protein G beads were collected, washed 3 times with Immunoprecipitation Buffer, resuspended in 3X Laemmli SDS-Sample Buffer (Boston BioProducts #BP-111R) at 70°C for 5 minutes, and separated by magnetic field. The supernatant was used in western blotting.

### Biotinylated-PG pulldown

Biotinylated-PG pulldown was carried out according to a published protocol (22).

### Mass spectrometry

Mass spectrometry sample preparation, liquid chromatography-mass spectrometry/mass spectrometry (LC-MS/MS) analysis and data processing, including the identification of citrullinated residues were performed according to published protocols (22).

## Results

### Preferential enrichment of PG-bound MZB1 in RA-ILD lungs

**Figure 1:**
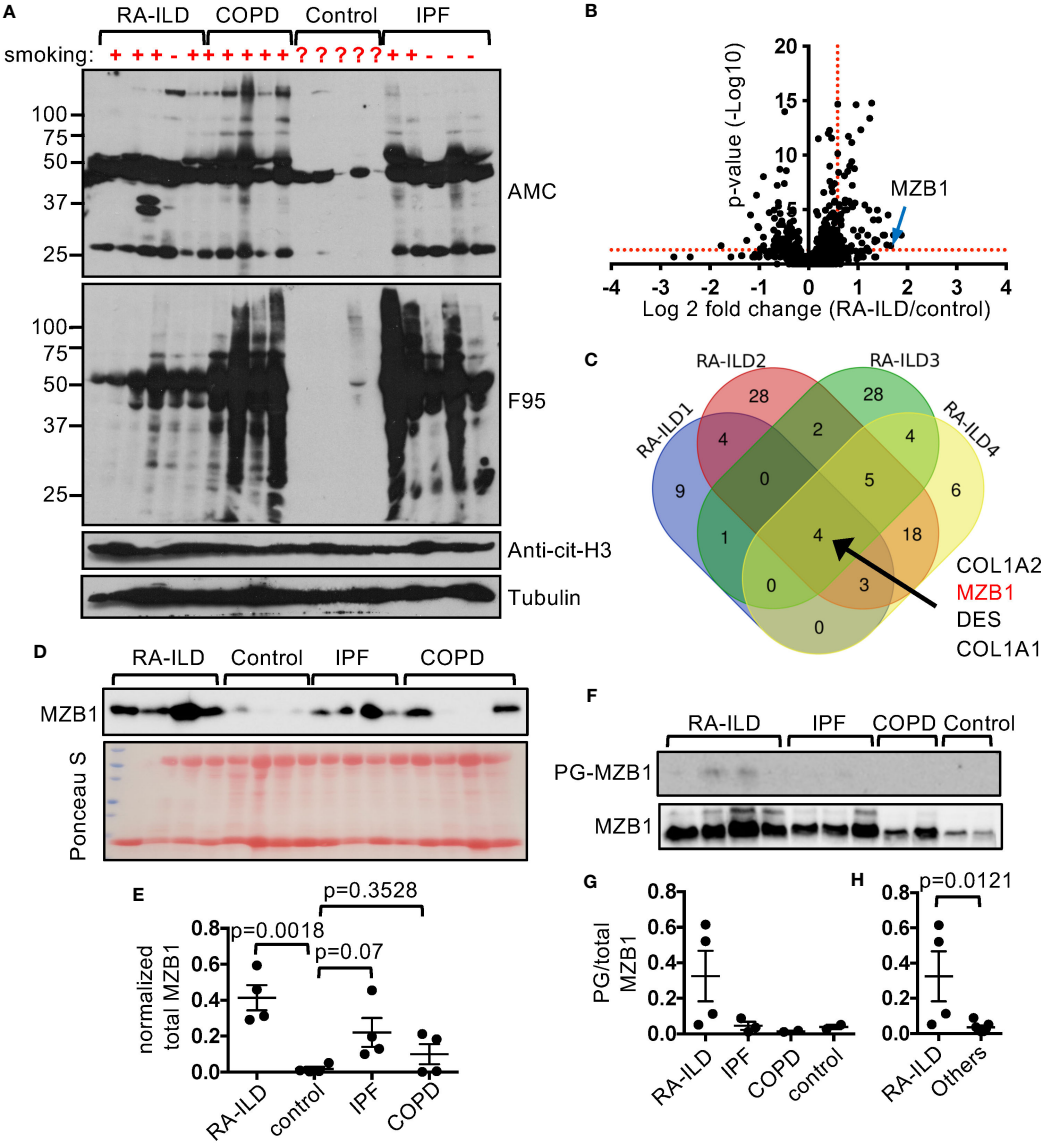
Preferential enrichment of PG-tagged MZB1 in RA-ILD. **(A)** Lung extract of indicated diseases and controls was probed with indicated antibodies in western blotting. **(B, C)** PG-tagged proteins in diseased and control lungs were purified and subjected to mass spectrometry. Each diseased lung sample was compared to two controls and the differentially enriched PG-tagged proteins of each comparison were shown in volcano plots. An example volcano plot comparing RA-ILD and control is shown in **(B)**. The red dotted lines indicate the threshold of fold change and p value. The Venn diagram of PG-tagged lung protein enriched in 4 RA-ILD samples is shown in **(C)**. **(D, E)** Lung extract of indicated diseases and control was probed with anti-MZB1 in western blotting or stained with Ponceau S **(D)**. The density of the MZB1 protein bands was normalized against Ponceau S staining. The normalized density is shown in **(E)**. **(F–H)** PG-tagged lung proteins and whole protein extract of indicated diseases and controls were probed with anti-MZB1 in western blotting **(F)**. The density ratios between PG-tagged and total MZB1 are shown in **(G)**. The density ratios of all non-RA-ILD samples were pooled together to compare with those of RA-ILD samples in **(H)**. Statistical analysis was performed with unpaired Student’s t test in **(H)** and one-way ANOVA followed by multiple comparisons in **(E)**.

Tissue hypercitrullination has been reported in several diseases, including the synovium in rheumatoid arthritis and lung in IPF (23, 24). In agreement with these reports, we found that the levels of F95-reactive and AMC-reactive proteins were highly elevated in the lung tissue from patients with RA-ILD, IPF, and COPD compared to control lungs (**Figure 1A**). By contrast, the levels of cit-H3 were comparable, suggesting that hypercitrullination does not occur to every protein. In addition, there were subtle differences in the patterns of the F95-reactive and AMC-reactive proteins among the diseased lungs. To further identify the proteins that are citrullinated in the diseased lungs, we incubated the whole lung extract with bio-PG, purified the PG-tagged proteins with streptavidin, and then subjected the PG-tagged proteins to MS for protein identification and quantification. Each diseased lung sample (4 RA-ILD, 3 IPF, and 3 COPD) was examined in triplicate and compared to two control samples, which were also examined in triplicate. We then used the threshold of FDR <0.1, p<0.05 and fold change ≥1.5 to identify PG-tagged proteins that were enriched in each diseased sample (**Figure 1B**, **Supplementary Figure 1A**, **Supplementary Tables 3–12**). We subsequently looked for PG-tagged proteins that were enriched in all samples of each disease and found 4, 3, and 11 such PG-tagged proteins for RA-ILD, IPF, and COPD, respectively (**Figure 1C**, **Supplementary Figures 1B, C**). However, these PG-tagged proteins were not uniquely enriched in any specific disease. For example, PG-tagged COL1A2, DES, and COL1A1 were enriched in all 4 RA-ILD samples but were also in 1-2, but not all, samples of IPF and COPD. The only exception is PG-tagged MZB1, which was enriched in all RA-ILD samples but not in any samples from the other two diseases.

Overexpression of MZB1 has been observed in lung tissue from IPF and ILD associated with connective tissue diseases (10). To confirm the MS data, we first subjected the crude lung extract to western blotting using anti-MZB1. In agreement with the published data, the total levels of MZB1 of RA-ILD and IPF were higher than that of COPD and controls (**Figures 1D, E**). It was readily detected in all RA-ILD and IPF samples but also in two out of four COPD samples, and barely detectable in controls. We then adjusted the amount of crude extract from samples with readily detectable MZB1 with the intention of equalizing the total MZB1. The adjusted amount of crude extract was subjected to PG pulldown followed by anti-MZB1 western blotting. We found more MZB1 in PG-tagged pool in RA-ILD compared to that of IPF, COPD and controls (**Figures 1F, G**). The fractions of PG-tagged MZB1 (PG-tagged/total) were also numerically higher in RA-ILD compared to that of the other diseases as a group (**Figure 1H**). These results confirm our MS data; however, we cannot confidently conclude that the fraction of PG-tagged MZB1 or the total level of MZB1 are higher in RA-ILD compared to IPF given the wide variation in the level of total MZB1 among the diseases and the limited numbers of samples.

### MZB1 is a substrate of PAD2 *in vitro* and *in vivo*

**Figure 2:**
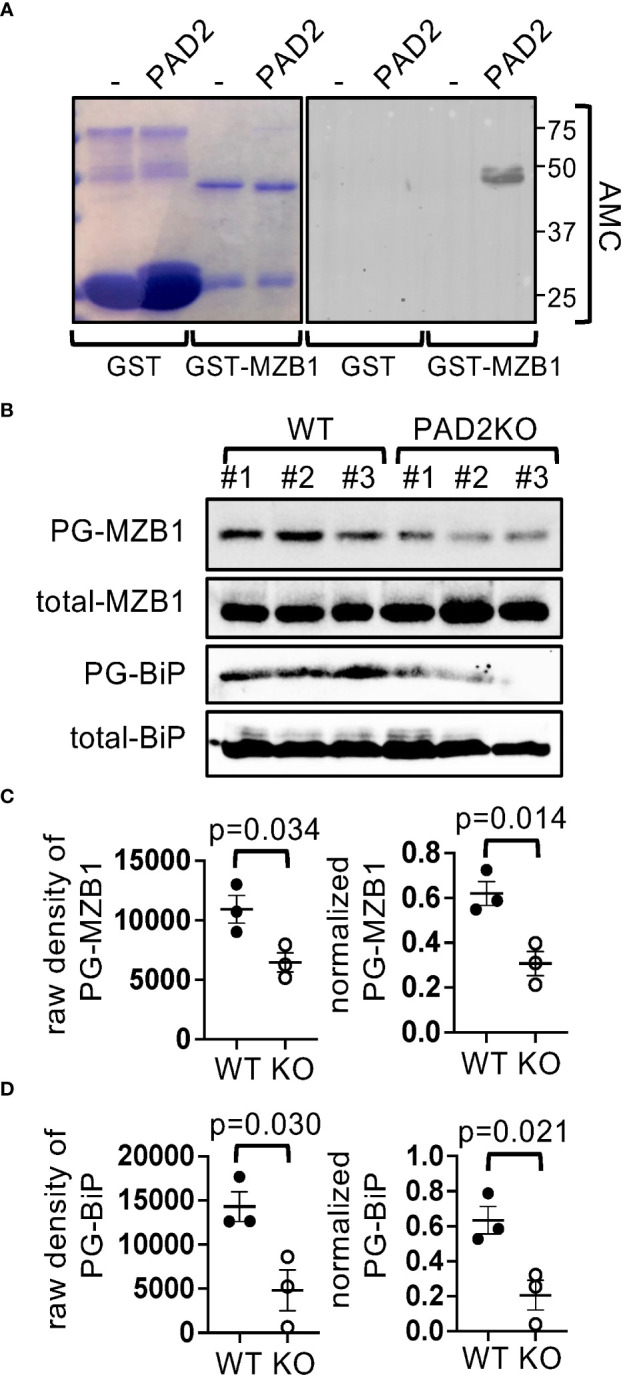
MZB1 is a substrate of PAD2. **(A)** Recombinant GST or GST-human MZB1 were incubated with PAD2 and stained with Coomassie Blue or AMC in western blotting. **(B–D)** PG-tagged and total MZB1 and BiP in splenic cell lysate from WT or PAD2KO mice (N=3 per genotype) was examined in western blotting **(B)**. The raw density of PG-tagged MZB1 and BiP was normalized against the density of total MZB1 and BiP, respectively. The raw density and normalized level of MZB1 **(C)** and BiP **(D)** are shown. The p values in **(C, D)** were calculated with unpaired Student’s t test.

Citrullination of COL1 and DES, but not MZB1, has been previously reported (25). To confirm that MZB1 is a PAD substrate and to identify its potential citrullination sites, we incubated recombinant human GST-MZB1 *in vitro* with PAD2, the dominant PAD in lymphoid cells, and found that GST-MZB1 but not GST became reactive to antibody against modified citrulline (AMC) after incubation with PAD2 (**Figure 2A**). In agreement with the *in vitro* citrullination results, the level of PG-tagged MZB1, but not total MZB1, was reduced by approximately 50% in PAD2-deficient mouse B cells (**Figures 2B, C**), further confirming that MZB1 is a substrate of PAD2. Interestingly, the level of PG-tagged BiP, another ER protein, was also reduced in PAD2KO B cells (**Figures 2B, D**), indicating that PAD2 can citrullinate several ER proteins in mouse B cells under physiological conditions. The residual PG-tagged MZB1 and PG-tagged BiP was probably due to citrullination by other PADs or other post-translational modifications of MZB1 and BiP, such as homocitrullination.

**Figure 3:**
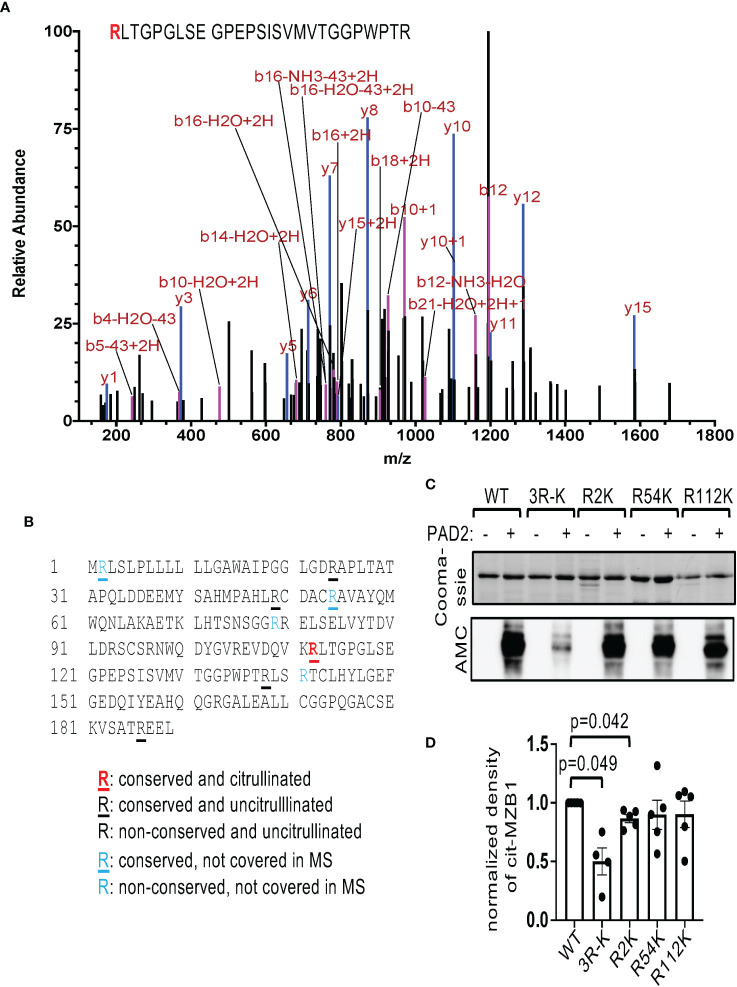
Identification of citrullination sites of MZB1. **(A**, **B)** The PAD2 citrullinated GST-MZB1 shown in **Figure 2A** was subjected to mass spectrometry to map the location of peptidylcitrulline. The MS/MS tracing of R112 is shown in **(A)**. The amino acid sequence of human MZB1 is shown in **(B)**. The status of citrullination of each arginine residue is indicated. **(C, D)** Recombinant GST-tagged human MZB1, either WT or carrying the indicated R-K mutations, were incubated with or without PAD2, and examined with Coomassie Blue staining [the top panel of **(C)**] and AMC in western blotting [the bottom panel of **(C)**]. The normalized density of citrullinated MZB1 from 4-5 experiments is shown in **(D)**. The p values were calculated with one-way ANOVA followed by multiple comparisons.

We subsequently submitted PAD2-citrullinated human GST-MZB1 for mass spectrometry. We followed the neutral loss of isocyanic acid (-CNOH, -43.0058 Da), which is unique to citrulline-containing ion fragments and is frequently observed during high-energy collision dissociation. Using this method, ten out of the 14 arginine residues in MZB1 were covered and R112, which is conserved between human and mouse, was confirmed to be citrullinated (**Figures 3A, B**). However, converting R112 of MZB1 to lysine, which maintains the positive charge but cannot be citrullinated, did not attenuate the susceptibility of MZB1 to PAD2-mediated citrullination when examined with AMC (**Figures 3C, D**); suggesting the presence of additional citrullination sites. There are four arginine residues in human MZB1, namely R2, R54, R79, and R141, that were not covered in the MS analysis. Among them, only R2 and R54 are evolutionary conserved. We therefore also converted R2 and R54 of human MZB1 individually to K and incubated the mutants with PAD2. The reactivity to AMC of citrullinated R2K but not R54K was subtly reduced (**Figures 3C, D**). When all three R were converted to K, the density of the AMC-reactive MZB1 was reduced by approximately 50% (3R-K in **Figures 3C, D**), suggesting that multiple arginine residues, including R2, R54, and R112, contribute to the PAD2-mediated citrullination.

Despite the reduced level of PG-tagged MZB1 in splenocytes of PAD2KO mice, the serum levels of various immunoglobulin isotypes, including IgM, IgA, and IgE were comparable between WT and PAD2KO littermates (**Supplementary Figure 2A**). There was a subtle reduction in the level of IgG, a finding consistent with published data (26). The levels of IgM, IgA, and IgG in the supernatant of *in vitro* differentiated PAD2KO plasmablasts were also normal (**Supplementary Figure 2B**).

### MZB1 is citrullinated in primary human plasamablasts

**Figure 4:**
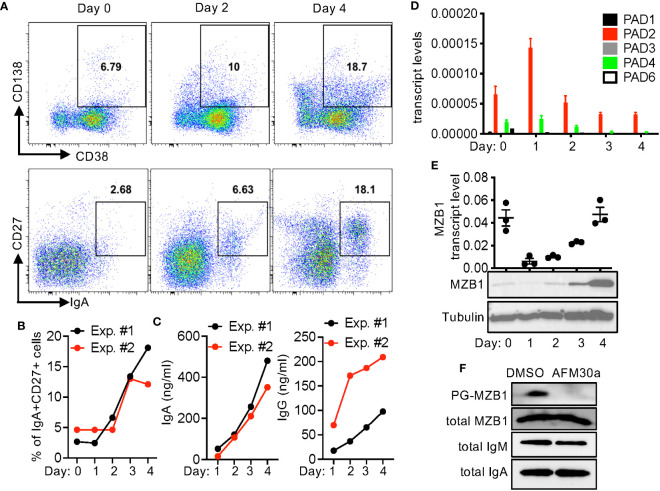
*In vitro* plasmablast differentiation of primary human B cells. **(A–E)** Primary B cells were purified from PBMC of healthy donors and differentiated into plasmablasts *in vitro*. The cells were stained for indicated surface markers and intracellular IgA and analyzed with FACS. Representative CD138/CD38 and CD27/IgA plots at indicated time points are shown in **(A)**. The percentages of IgA^+^CD27^+^ cells are shown in **(B)**. The levels of IgA and IgG in the supernatant were quantified with ELISA **(C)**. The transcript levels of PADs **(D)** and MZB1 [**(E)**, the top panel] were measured with qPCR. Whole cell extract was analyzed in western blotting using anti-MZB1 and anti-tubulin [**(E)**, the bottom two panels]. **(F)** Primary human B cells were differentiated in the presence of DMSO or AFM-30a (20 uM). Cell extract from two experiments was pooled together; the levels of total and PG-tagged MZB, total IgM, and total IgA were examined with western blotting.

The expression of MZB1 is induced during human plasma cell differentiation. To examine whether MZB1 is also citrullinated in human primary B cells, we set up an *in vitro* human plasmablasts differentiation protocol. Purified primary human B cells were stimulated with IL-2, IL-10, IL-15, CpG ODN, and soluble CD40L (or anti-CD40) for 4 days. The differentiation of plasmablasts was confirmed by the upregulation of CD27, CD138, and CD38. The expression of IgA was examined with intracellular staining. Approximately 12-18% of cells were positive for both IgA and CD27 on day 4 (**Figures 4A, B**). The appearance of CD27^+^ plasmablasts correlated with the rise in the levels of IgA and IgG in supernatant (**Figure 4C**). We then examined the expression of PADs with qPCR. Expectedly, PAD2 was the dominant PAD and its level remained relatively steady through the differentiation, with the highest level detected on day 1 (**Figure 4D**). The levels of other PADs were too low for meaningful assessment. We were also unable to detect PAD2 proteins by western blot.

By contrast, MZB1 transcripts were readily detectable on day 0 (**Figure 4E**); the level dropped precipitously on day 1 but then rose along with the appearance of CD27^+^ plasmablasts. This increase in the expression from day 1 to day 4 is consistent with the preferential expression of MZB1 in plasmablasts/plasma cells. We were able to confirm the high expression of MZB1 on day 4 by western blot (**Figure 4E**). Surprisingly, the protein level of MZB1 on day 0 was much lower than that of day 4 despite the comparable transcript levels. This discrepancy suggests that the protein level of MZB1 may be also susceptible to post-transcriptional regulation. A fraction of the MZB1 proteins on day 4 could be tagged by bio-PG (**Figure 4F**). The level of PG-tagged MZB1, but not total MZB1, was almost undetectable when the B cells were differentiated in the presence of AFM-30a, a PAD2-specific inhibitor (27), suggesting that MZB1 in primary human plasmablasts is mainly citrullinated by PAD2. Unexpectedly, treatment with GSK199, a PAD4-specific inhibitor, or BB-Cl-amidine, a pan-PAD inhibitor, markedly reduced the survival of the differentiating plasmablasts even at a lower dose (**Supplementary Figure 3**).

**Figure 5:**
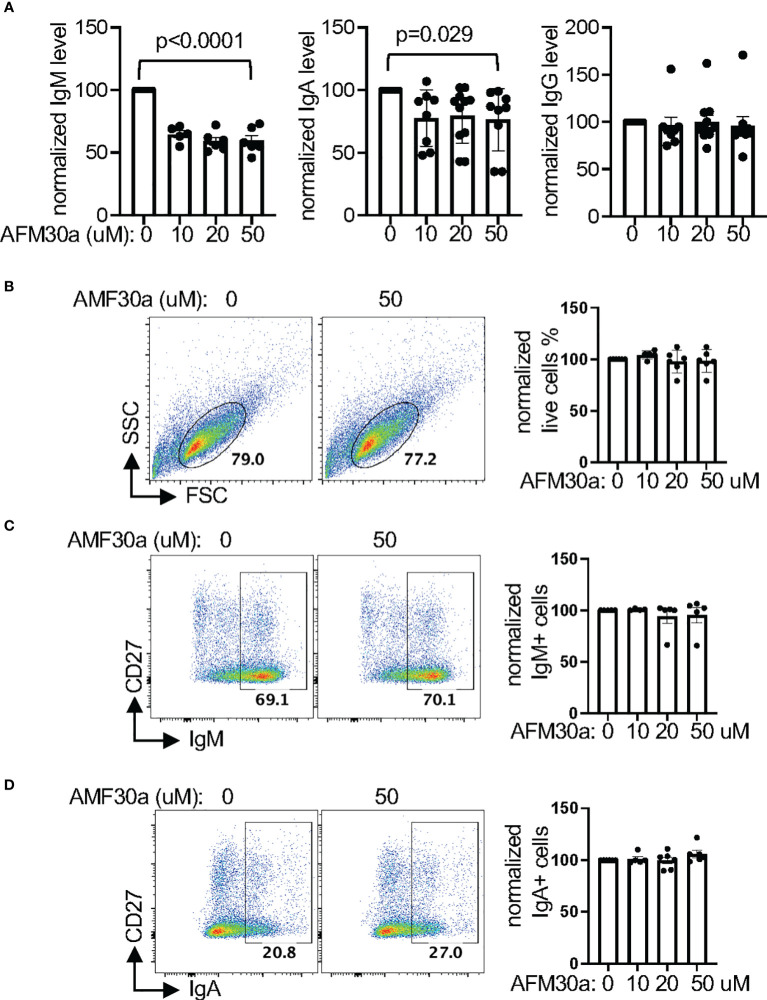
Pharmacological inhibition of PAD2 attenuates the secretion of IgM and IgA. **(A–D)** Primary human B cells were differentiated into plasmablasts in the presence of indicated concentration of AFM-30a. The normalized levels of indicated immunoglobulin in supernatant were shown in **(A)**. The raw levels of immunoglobulin in supernatant in the absence of AFM-30a range from 198.4 ng/ml to 39794.6 ng/ml for IgM, 48.1 ng/ml to 1368.4 ng/ml for IgA, and 122.6 ng/ml to 2009 ng/ml for IgG. The differentiated plasmblasts were also subjected to FACS analysis. Representative FSC/SSC plots of all cells are shown in **(B)** and representative IgM/CD27 and IgA/CD27 plots of live cells from **(B)** are shown in **(C, D)**, respectively. The normalized percentages of indicated populations from at least four experiments are shown in the bar graph of each panel. The raw percentages of the indicated gates from untreated samples (0 uM) range from 45% to 79% in **(B)**, 67% to 70% in **(C)**, and 14% to 27% in **(D)**, which were then arbitrarily set as 100% for normalization. The p values were calculated with one-way ANOVA.

Interestingly, we found that AFM-30a reduced the levels of IgM in the supernatant of *in vitro* differentiated primary human plasmablasts (**Figure 5A**). AFM-30a also reduced the level of IgA in supernatant; however, this effect was less robust and more variable. By contrast, the level of IgG was unaffected by AFM-30a. The cell survival/recovery (**Figure 5B**, **Supplementary Figure 4A**) and the upregulation of CD27 and CD38 (**Supplementary Figure 4B**), two markers of plasmablasts, were comparable between treated and un-treated cells. In addition, the percentage of IgM^+^ or IgA^+^ cells within live cells (**Figures 5C, D**), the percentages of IgM^+^IgA^-^, IgM^-^IgA^+^, and IgM^-^IgA^-^ (presumably IgG^+^) cells within the CD27^+^ population (**Supplementary Figure 4C**) were also not affected. Taken together, the data suggest that AFM-30a does not affect the differentiation of plasmablasts or the ratio of IgM, IgA, or IgG-expressing plasmalasts but instead inhibits the secretion of IgM and to a lesser degree IgA, a phenotype similar to that of MZB1-deficient mouse plasmablasts.

### Both MZB1 and PAD2, but not PAD4, are required for optimal IgM and IgA secretion by primary human plasmablasts

**Figure 6:**
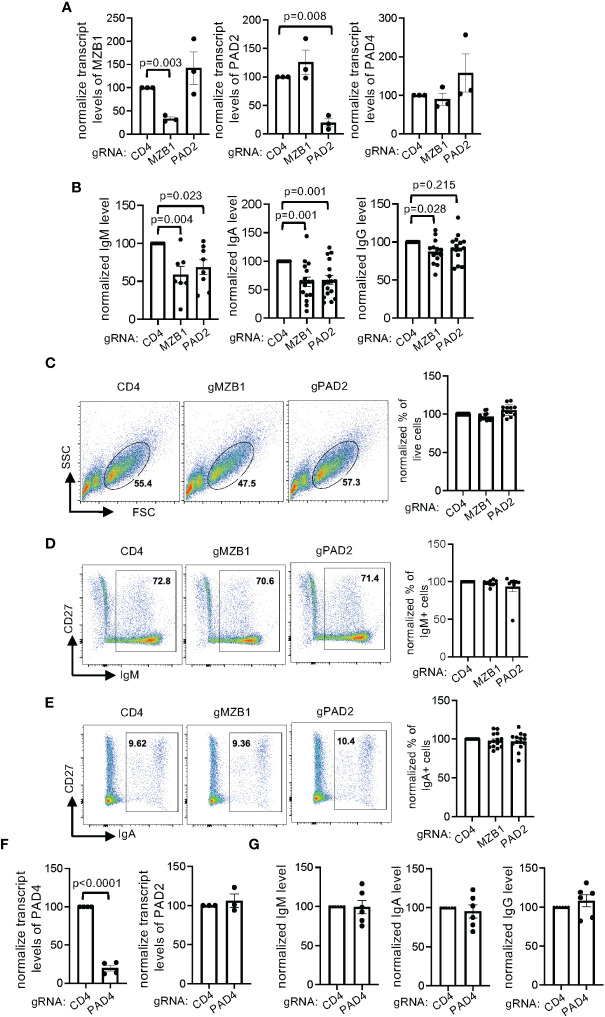
Regulation of IgM and IgA secretion by MZB1 and PAD2, but not PAD4, in primary human B cells. **(A–G)** Primary human B cells from healthy donors were subjected to CRISPR using indicated gRNAs and subsequently *in vitro* differentiation into plasmablasts. The expression of MZB1, PAD2, and PAD4 in the differentiated cells was examined with qPCR **(A, F)**. The levels of IgM, IgA, and IgG in supernatant were measured with ELISA. The normalized levels are shown in **(B, G)**. The absolute levels of CD4 gRNA-transfected samples range from 378 ng/ml to 8442 ng/ml for IgM, 284 ng/ml to 1782 ng/ml for IgA, and 145 ng/ml to 4977 ng/ml for IgG. The plasmablasts were stained for surface CD27 and intracellular IgM and IgA. Representative plots of FSC/SSC **(C)**, CD27/IgM **(D)** and CD27/IgA **(E)** are shown. In each experiment, the percentage obtained from CD4 gRNA was arbitrarily set as 100%. The raw values of the indicated gates ranges from 37% to 74% for live cells, 39% to 85% for IgM^+^ cells, 7% to 44% for IgA^+^ cells. The statistical analysis was performed with paired Student’s t test **(A, F)** and one-way ANOVA followed by multiple comparisons **(B)**.

The role of MZB1 in primary human B cells has never been examined and AFM-30a can have off-target effects. To confirm the role of MZB1 and PAD2 in promoting the secretion of IgM and IgA in primary human plasmablasts, we chose to use CRISPR to ablate each gene in primary human plasmablasts. To this end, we optimized a CRISPR protocol and determined whether it was compatible with *in vitro* plasmablast differentiation. We selected CD19 as a model target and used CD4 small guiding RNA (gRNA) as a negative control. We were able to reduce the surface level of CD19 by almost 90%; the transfected cells were still able to differentiate into IgA-expressing cells *in vitro* (**Supplementary Figure 5A**). We subsequently designed two gRNAs, separately targeting MZB1 and PAD2 in primary human B cells. To estimate the efficiency of CRISPR, we designed qPCR primers that extend 3bp beyond the expected CRISPR-induced breakpoint of the gRNAs to measure the transcript level of MZB1 and PAD2. This approach very likely underestimated the CRISPR efficiency but still indicated that the gRNAs reduced the expression of MZB1 and PAD2 by more than 60% (the left and the middle panels of **Figure 6A**). We were unable to confirm the efficiency of CRISPR with western blotting because of the low cell recovery after CRISPR. The ablated cells were then subjected to *in vitro* differentiation and the levels of secreted immunoglobulin were quantified with ELISA. We found that ablation of MZB1 reduced the level of IgM and IgA in the supernatant by approximately 50% (**Figure 6B**), phenocopying murine MZB1-deficient B cells. The level of IgG in the supernatant was only subtly affected by the MZB1 gRNA. Ablation of PAD2 also comparably reduced the level of IgM and IgA, an effect similar to that of MZB1 ablation or AFM-30a treatment, but had no impact on the level of IgG. This effect of PAD2 ablation is not due to an alteration in the expression of MZB1 because PAD2 gRNA had no impact on the transcript level of MZB1, and vice versa the left and the middle panels of (**Figure 6A**). Neither gRNA affected the expression of PAD4 (the right panel of **Figure 6A**), which is located at the same locus with PAD2 in the human genome. By contrast, ablation of neither MZB1 nor PAD2 had any effect on the cell survival/recovery (**Figure 6C**, **Supplementary Figure 5B**), the induction of CD27 and CD38 (**Supplementary Figure 5C**), the percentage of IgM^+^ or IgA^+^ cells among live cells (**Figures 6D, E**), the distribution of IgM^+^IgA^-^, IgM^-^IgA^+^, and IgM^-^IgA^-^ cells within the CD27^+^ population (**Supplementary Figure 5D**), or the MFI of IgM or IgA staining (**Supplementary Figure 5E**). Taken together, the results strongly suggest that both MZB1 and PAD2 are required for optimal secretion of IgM and IgA in primary human plasmablasts.

PAD4 is the second highest expressed PADs in differentiating plasmablast (**Figure 4D**). To determine whether PAD4 has a function similar to that of PAD2, we used CRISPR to reduce the expression of PAD4 by almost 80% (**Figure 6F**). Ablation of PAD4 did not lead to compensatory overexpression of PAD2 (**Figure 6F**), affect the differentiation of IgA or IgM-expressing plasmablasts (**Supplementary Figure 6**), or alter the level of IgM, IgA, and IgG in supernatant (**Figure 6G**).

**Figure 7:**
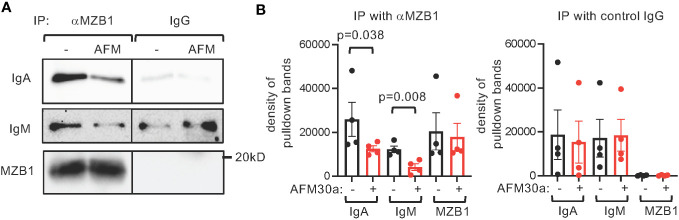
Attenuated interaction between endogenous MZB1 and IgM/IgA by AFM-30a. Human primary B cells were differentiated *in vitro* into plasmablasts in the absence or presence of AFM-30a (25 uM). Whole cell extract was harvested and subjected to immunoprecipitation with anti-MZB1 or control IgG. The precipitate was probed with indicated antibodies. Representative western blots are showin in **(A)**. The density of the pulldown bands was quantified with ImageJ. The cumulated results are shown in **(B)**. The p values were calculated with paired Student’s t test.

MZB1 promotes the secretion of IgM and IgA by physically interacting with the tail piece of their heavy chain and subsequently mediating their binding to J chain. We postulated that citrullination of MZB1 facilitates its interaction with IgM and IgA. We therefore carried out co-immunoprecipitation with anti-MZB1 in protein extract obtained from primary human plasmablasts differentiated in the presence or absence of AFM-30a. Expectedly, anti-MZB1 but not control IgG immunoprecipitated MZB1; anti-MZB1 also co-immunoprecipitated IgM and IgA (**Figure 7A**). The physical interaction between MZB1 and IgM/IgA in plasmablasts, but not precipitated MZB1 or total IgM/IgA, was attenuated in the presence of AFM-30a (**Figures 4F**, **7A, B**). There was non-specific interaction between control IgG and IgM/IgA in some experiments despite extensive pre-clearing; however, the non-specific interaction was not affected by AFM-30a.

## Discussion

PADs have been shown to regulate the function of several types of immune cells, including neutrophils (1, 19), T cells (2, 28, 29), and macrophages (30). The results generated from this study further expand the role of PAD2 to B cells, i.e. regulating the secretion of IgM and IgA. The results are somewhat unexpected given that B cells only express very low levels of PAD2 and contain barely detectable AMC-reactive proteins even after stimulation with ionomycin (3), which is known to induce calcium influx and increase PAD activity. This unexpected function of PAD2 in B cells further strengthens the notion that PAD activity cannot be reliably predicted based on their transcript and protein levels. Similarly, our data also indicate that the expression of MZB1 is not solely regulated by transcription, highlighting the critical role of post-transcriptional regulation of protein function and the need for sensitive assays to detect citrullinated proteins.

MZB1 interacts with the tail piece of IgA (aTP) in B cells, and this intracellular interaction is dependent on the penultimate Cys residue (C482) of aTP. Surprisingly, *in vitro* biolayer interferometry was unable to show any interaction between MZB1, which was generated from *E. coli*, and the C-terminal Ig domain of IgA containing aTP (18). This discrepancy may suggest that MZB1 interacts with aTP indirectly through a third-party protein. Our data showing that the interaction between MZB1 and IgM/IgA is attenuated by AFM-30a, however, provides an alternative explanation, i.e. the lack of interaction in biolayer interferometry is due to uncitrullinated status of MZB1 generated in *E. coli*. It will be critical to test whether *in vitro* citrullinated MZB1 can interact with IgA in the biolayer interferometry. This approach will also enable us to identify the citrullination sites that are critical for such interaction. Although our data strongly suggests that PAD2 promotes the secretion of IgM and IgA by citrullinating MZB1, direct evidence demonstrating this linear causal relationship is still lacking. It is still possible that PAD2 acts on other proteins to facilitate the secretion of IgM and IgA. The observation that the level of PG-tagged BiP, another ER protein, was also reduced in PAD2KO B cells is in support of this scenario. Introduction of PAD2-resistant or constitutively citrullinated MZB1 to primary human plasmablasts will be very informative. Such an approach will require thorough mapping of citrullination sites of MZB1 and the development of a method to introduce site-specific citrullination in live cells. While we recently developed a protocol to introduce site-specific citrullination through codon engineering in mammalian cells (31), this protocol in its current state is still not suitable for primary cells.

MZB1 is also citrullinated by PAD2 in murine splenocytes. Thus, the observation that PAD2 deficiency has little impact on the homeostasis of IgM and IgA in mouse is somewhat unexpected. This unexpected result could be due to functional compensation from other PADs, such as PAD4. Indeed, PAD2 deficiency only partially reduces the level of PG-tagged MZB1 in murine splenocytes and R112 of MZB1 can also be citrullinated *in vitro* by PAD4. Such functional compensation by PAD4, if it does occur in murine B cells, probably plays a negligible role in human plasmablasts given the data shown in **Figures 4F**, **6G**. Alternatively, the mechanisms regulating the secretion of IgA and IgM may differ between mouse and human plasmablasts. Examining B cells derived from mice deficient in both PAD2 and PAD4 (32) will help distinguish these two scenarios.

Nevertheless, our findings are consistent with a report by Schiller et al. showing increased levels of MZB1 proteins in the lung tissue of several chronic lung diseases, including IPF, hypersensitive pneumonitis, and connective tissue disease-associated ILD (10), which have distinct but overlapping pathogenesis. However, the status of MZB1 citrullination was not examined in the Schiller study. While PG-tagged MZB1 was preferentially enriched in RA-ILD lungs in our study, it is too premature to conclude that hyper-citrullination of MZB1 is a unique feature of RA-ILD given the small sample size of the current study. In addition, PG also binds to homocitrullinated proteins and it is still unclear whether the PG-tagged MZB1 in the diseased lung is citrullinated or homocitrullinated MZB1. Despite these caveats, the possibility that hypercitrullination of MZB1 is a unique feature of RA-ILD is supported by recent publications showing that several major genetic and environmental risk factors of RA, such as family history, SNPs at the PTPN22 gene, periodontitis, and cigarette smoking, are associated with local or systemic hypercitrullination in healthy individuals (33–36).

Our results also add insights to the origin of RA. Rheumatoid factor is antibody directed against the Fc portion of IgG, and was first detected in patients with RA. It can be made up of various Ig isotypes with IgM the dominant isotype. Interestingly, IgM RF typically appears first in serum, followed by IgA RF and then IgG RF, before the diagnosis of RA (37); and high level of IgM RF and IgA RF are relatively specific for RA (37, 38). Emerging data has strongly suggested that the autoimmune process of RA very likely originates in mucosal sites (39), where IgA is the major component of the humoral immune defense. For example, IgA ACPA can be detected in sputum or periodontal inflammatory exudate from healthy individuals even without serum ACPA (40, 41). In addition, a higher percentage of IgA^+^ plasmablasts has been observed in peripheral bloods derived from healthy at-risk (ACPA+) individuals compared to ACPA- individuals (42). Thus, altered IgM and IgA immune response probably predates RF and ACPA sero-positivity and clinical rheumatoid arthritis. As overexpression of MZB1 has been detected in RA synovium and periodontitis gingival tissues and citrullination very likely promotes the function of MZB1, one may wonder whether aberrant expression along with hyper-citrullination of MZB1 contribute to the altered IgM and IgA immune response and the development of RF, ACPA and RA. It will be very informative to examine whether major risk factors of RA, such as PTPN22 SNP and family history, are associated with overexpression and hypercitrullination of MZB1.

One unique feature of ACPAs is their cross-reactivity to many citrullinated peptides bearing no sequence or structural homology other than the presence of peptidylcitrulline residues (43, 44). Hence, it is very intriguing to see that BiP is also a PAD substrate, a finding consistent with the observations that citrullinated BiP is an auto-antigen in rheumatoid arthritis and type 1 diabetes (45, 46). It is possible that the major RA risk factors lead to hypercitrullination of MZB1 and many other ER proteins. Such a hypercitrullinated ER environment, enriched with peptidylcitrulline residues, not only is a rich source of citrullinated antigens but also promotes the secretion of IgM/IgA and contributes to the unique feature of ACPAs. Generation of antibodies specific for citrullinated MZB1 and other ER proteins will allow us to test this hypothesis.

## Data availability statement

The mass spectrometry proteomics data have been deposited to the ProteomeXchange Consortium via the PRIDE partner repository (https://www.ebi.ac.uk/training/online/courses/proteomics-an-introduction/proteomics-resources-at-the-ebi/pride/) with the dataset identifier PXD047044 (47).

## Ethics statement

The studies involving humans were approved by Mass General Brigham Institutional Review Board. The studies were conducted in accordance with the local legislation and institutional requirements. The participants provided their written informed consent to participate in this study. The animal study was approved by IACUC Brigham and Women’s Hospital. The study was conducted in accordance with the local legislation and institutional requirements.

## Author contributions

BG: Conceptualization, Data curation, Formal Analysis, Investigation, Methodology, Validation, Writing – original draft, Writing – review & editing. BS: Conceptualization, Data curation, Formal Analysis, Investigation, Methodology, Project administration, Supervision, Validation, Writing – original draft, Writing – review & editing. RT: Conceptualization, Data curation, Formal Analysis, Methodology, Resources, Visualization, Writing – original draft, Writing – review & editing. LB: Data curation, Formal Analysis, Methodology, Resources, Writing – original draft, Writing – review & editing. KT: Conceptualization, Data curation, Formal Analysis, Funding acquisition, Resources, Writing – original draft, Writing – review & editing. IR: Conceptualization, Data curation, Formal Analysis, Funding acquisition, Methodology, Resources, Supervision, Writing – original draft, Writing – review & editing. PT: Conceptualization, Data curation, Formal Analysis, Funding acquisition, Investigation, Methodology, Resources, Supervision, Writing – original draft, Writing – review & editing. I-CH: Conceptualization, Data curation, Formal Analysis, Funding acquisition, Investigation, Methodology, Project administration, Resources, Supervision, Validation, Visualization, Writing – original draft, Writing – review & editing.
