# Supplementary material for: Peptidylarginine deiminase 2 citrullinates MZB1 and promotes the secretion of IgM and IgA
Source: Front Immunol. 2023 Nov 29;14:1290585. doi: 10.3389/fimmu.2023.1290585 (PMC10716219; doi:10.3389/fimmu.2023.1290585)
Supplement: Supplementary file 1 [file DataSheet_1.pdf]

Supplemental Table 1: The sequence of qPCR primers

Regular qPCR primers		
Gene	Forward	Reverse
hPAD1	5′-CCTCACTGGCGTCGATATTT-3′	5′-TCACAGTTCACCAGCAAGATAG-3′
hPAD2	5'- GGTCAGCCATATCCATCTTCTC -3'	5'- GACAGATGGCACCAGGAATAA -3'
hPAD3	5'- GGCCAAGATAAGGTGTCCTATG -3'	5'- CAGAGTGACATGGAAGGAGATG -3'
hPAD4	5'- CCCTCCAGCCAAGAAGAAAT -3'	5'- AGTCTTGGGTCCGTAGTATGA -3'
hPAD6	5'- GCAGATCAGCTCCTGTCTAATG -3'	5'- TGAATGCACTTCTCCACGTATT -3'
hMZB1	5'- GGCAGGACTACGGAGTTCGAGAAG -3'	5'- TTCTCCAAACTCCCCCAAGTAGTG -3'
hACTIN	5'- GTGACAGCAGTCGGTTGGAG -3'	5'- AGGACTGGGCCATTCTCCTT -3'
hGAPDH	5'-AGGTGAAGGTCGGAGTCAACG-3'	5'-GATGACAAGCTTCCCGTTCTCAG-3'
qPCR primers for determining CRISPR efficiency		
Gene	Forward	Reverse
hPAD2	5'- ATGTCTACAGCGCGGCCCCAGCCG -3'	5'- CTTGTCACTGCTGGCCTCGGTGCT -3'
hPAD4	5'- GACTCAGCTTGACATCTGCAGCTC -3'	5'- ATGAAATCTGAACCTTCTGGTCGC -3'
hMZB1	5- AGTGGACCAAGTGAAACGTCTCAC -3′	5- TTCTCCAAACTCCCCCAAGTAGTG -3′
