# Supplementary material for: Peptidylarginine deiminase 2 citrullinates MZB1 and promotes the secretion of IgM and IgA
Source: Front Immunol. 2023 Nov 29;14:1290585. doi: 10.3389/fimmu.2023.1290585 (PMC10716219; doi:10.3389/fimmu.2023.1290585)
Supplement: Supplementary file 2 [file DataSheet_2.pdf]

Supplemental Table 2: Sequences of CRISPR sgRNAs

hCD4	UUUAAGCACGACUCUGCAGA
hCD19	CACAGCGUUAUCUCCCUCUG
hPAD2	CCACAGCGCGGCCCCAGCCG
hPAD4	GAAGGACGUGCAGUCCUCAG
hMZB1	CAGCUACGGAGUUCGAGAAG
