# Supplementary material for: Peptidylarginine deiminase 2 citrullinates MZB1 and promotes the secretion of IgM and IgA
Source: Front Immunol. 2023 Nov 29;14:1290585. doi: 10.3389/fimmu.2023.1290585 (PMC10716219; doi:10.3389/fimmu.2023.1290585)
Supplement: Supplementary file 3 [file DataSheet_3.pdf]

Supplemental table 3: RA-ILD1 vs controls

Accession #	Fold Change	p value (-log10)
sp|Q15109-10|RAGE_HUMAN	-1.4849911	1.6997428
sp|P22748|CAH4_HUMAN	-1.2166309	2.1818786
sp|P59666|DEF3_HUMAN	-0.8102055	2.1818786
sp|P08174-3|DAF_HUMAN	-0.6758537	1.6960104
sp|P05164-3|PERM_HUMAN	-0.6667957	8.726646
sp|P01011|AACT_HUMAN	-0.6346245	6.6933327
sp|P12429|ANXA3_HUMAN	-0.6327896	7.6602716
sp|P50897-2|PPT1_HUMAN	-0.6278381	1.6960104
sp|P62805|H4_HUMAN	-0.5995846	6.3204336
sp|P80188|NGAL_HUMAN	-0.5571365	1.6092666
sp|P12821-2|ACE_HUMAN	-0.5417194	2.151766
sp|P60903|S10AA_HUMAN	-0.539362	1.6960104
sp|Q01469|FABP5_HUMAN	-0.4978695	4.534284
sp|P07339|CATD_HUMAN	-0.466135	7.044066
sp|P26006-1|ITA3_HUMAN	-0.4449863	2.8793902
sp|Q9NZA1-2|CLIC5_HUMAN	-0.4436836	2.5461748
sp|P08311|CATG_HUMAN	-0.4411736	2.838374
sp|P16284-3|PECA1_HUMAN	-0.4323654	4.0434685
sp|P61626|LYSC_HUMAN	-0.4291267	2.8384566
sp|Q9UGT4|SUSD2_HUMAN	-0.422863	2.058973
sp|P02769|ALBU_BOVIN	-0.4188442	6.464415
sp|O95810|CAVN2_HUMAN	-0.4095364	2.653196
sp|P02462|CO4A1_HUMAN	-0.4016218	1.4278674
sp|Q9Y624|JAM1_HUMAN	-0.3983898	1.9413493
sp|P50895|BCAM_HUMAN	-0.3900127	4.005173
sp|P35241|RADI_HUMAN	-0.3824883	1.9190748
sp|P11047|LAMC1_HUMAN	-0.368084	4.1101046
sp|O00159|MYO1C_HUMAN	-0.3436394	4.488742
sp|P10253|LYAG_HUMAN	-0.3214264	3.5908275
sp|P84103-2|SRSF3_HUMAN	-0.3206997	1.3635377
sp|Q13510-2|ASAH1_HUMAN	-0.3182621	4.146542
sp|P56199|ITA1_HUMAN	-0.3035851	4.0218377
sp|P02786|TFR1_HUMAN	-0.3028202	1.8842831
sp|Q13740-2|CD166_HUMAN	-0.2930183	1.6797456
sp|P14543-2|NID1_HUMAN	-0.2909279	2.5617142
sp|Q9UHG3|PCYOX_HUMAN	-0.2907658	3.3983748
sp|P51659|DHB4_HUMAN	-0.2898865	1.3815327
sp|Q8NBQ5|DHB11_HUMAN	-0.2863827	1.9413493
sp|P0CG47|UBB_HUMAN	-0.2799454	1.9951487
sp|P84243|H33_HUMAN	-0.2711144	1.6960104
sp|P55268|LAMB2_HUMAN	-0.2685852	3.1110814
sp|P05556|ITB1_HUMAN	-0.2590523	2.7528272
sp|P00352|AL1A1_HUMAN	-0.2516193	1.4067543

sp|P07858|CATB_HUMAN	-0.2434006	1.6853416
sp|P05026-2|AT1B1_HUMAN	-0.2374115	1.560883
sp|Q6NZI2|CAVN1_HUMAN	-0.2358055	2.1004598
sp|P31949|S10AB_HUMAN	-0.2310772	2.4829872
sp|P10412|H14_HUMAN	-0.2092686	1.6092666
sp|Q01082|SPTB2_HUMAN	-0.1629105	1.3056548
sp|P21810|PGS1_HUMAN	-0.1581726	1.804513
sp|P43652|AFAM_HUMAN	-0.1481495	2.3424563
sp|P23141-3|EST1_HUMAN	0.09622192	1.6817658
sp|Q03252|LMNB2_HUMAN	0.1172657	2.5059617
sp|P22626|ROA2_HUMAN	0.12138367	1.3403429
sp|Q07955-2|SRSF1_HUMAN	0.16879654	1.3481187
sp|P02768|ALBU_HUMAN	0.17430878	6.4079614
sp|P04040|CATA_HUMAN	0.18007088	2.157161
sp|P07988|PSPB_HUMAN	0.18580627	2.4945192
sp|P02749|APOH_HUMAN	0.1886177	1.827201
sp|Q08380|LG3BP_HUMAN	0.2004242	1.7703108
sp|Q07065|CKAP4_HUMAN	0.20416069	1.7865183
sp|P35908|K22E_HUMAN	0.22387695	6.1391983
sp|Q96AG4|LRC59_HUMAN	0.22819138	1.4278674
sp|P20700|LMNB1_HUMAN	0.22917938	6.4130163
sp|P04217|A1BG_HUMAN	0.2332077	1.5071542
sp|Q9UL46|PSME2_HUMAN	0.23475456	2.7624686
sp|P15088|CBPA3_HUMAN	0.23485565	1.6997428
sp|P0DOY2|IGLC2_HUMAN	0.2370243	1.3815327
sp|P0DOX5|IGG1_HUMAN	0.23784065	1.5121045
sp|P00450|CERU_HUMAN	0.2545414	3.274217
sp|P02730|B3AT_HUMAN	0.25569534	3.364204
sp|P45974-2|UBP5_HUMAN	0.26000977	1.662449
sp|P35749-4|MYH11_HUMAN	0.26433372	4.355441
sp|P01833|PIGR_HUMAN	0.26675797	3.8058946
sp|P0DOX8|IGL1_HUMAN	0.26750183	1.6997428
sp|P51911|CNN1_HUMAN	0.2717781	1.3211795
sp|P61978-3|HNRPK_HUMAN	0.28123093	2.3420908
sp|P02549-2|SPTA1_HUMAN	0.28153992	4.4263687
sp|P22105-1|TENX_HUMAN	0.28668213	6.682845
sp|P13645|K1C10_HUMAN	0.291193	8.18605
sp|P23456|Trypsin	0.2934265	2.441399
sp|O15061-2|SYNEM_HUMAN	0.30245972	2.2323174
sp|P15090|FABP4_HUMAN	0.30282974	2.6997027
sp|P02774-3|VTDB_HUMAN	0.30657387	5.736467
sp|P07951|TPM2_HUMAN	0.308424	1.321681
sp|P51888|PRELP_HUMAN	0.31163025	2.8435178
sp|Q15746-3|MYLK_HUMAN	0.3164673	3.2507055
sp|Q14315-2|FLNC_HUMAN	0.3172512	6.63471

sp|P00747|PLMN_HUMAN	0.3202095	2.2522726
sp|P31146|COR1A_HUMAN	0.32240677	1.4236159
sp|Q9BXN1|ASPN_HUMAN	0.32358932	1.3815327
sp|P35527|K1C9_HUMAN	0.32535744	3.7723744
sp|Q07507|DERM_HUMAN	0.3278618	1.9509854
sp|P08729|K2C7_HUMAN	0.33331108	3.9572785
sp|Q06323|PSME1_HUMAN	0.33463097	2.6576471
sp|P05455|LA_HUMAN	0.33516312	1.5121045
sp|P20591|MX1_HUMAN	0.33783722	1.4104178
sp|Q14624-2|ITIH4_HUMAN	0.33802986	3.125233
sp|P69891|HBG1_HUMAN	0.3394966	2.4829872
sp|Q13263|TIF1B_HUMAN	0.34016037	1.9697601
sp|P80303-2|NUCB2_HUMAN	0.34031868	1.7590232
sp|Q9P2E9|RRBP1_HUMAN	0.3441429	3.5386477
sp|P00734|THRB_HUMAN	0.3480358	3.0495367
sp|Q14019|COTL1_HUMAN	0.3601284	2.8384566
sp|P01861|IGHG4_HUMAN	0.36026	1.9951487
sp|P00492|HPRT_HUMAN	0.36094475	1.5646582
sp|P13647|K2C5_HUMAN	0.36424446	4.964395
sp|O96000-2|NDUBA_HUMAN	0.3657608	1.4104178
sp|P61254|RL26_HUMAN	0.36811447	1.7590232
sp|P19823|ITIH2_HUMAN	0.3681984	4.628043
sp|P11277-3|SPTB1_HUMAN	0.37147903	2.2752137
sp|P28331-3|NDUS1_HUMAN	0.38436508	1.3561004
sp|P30837|AL1B1_HUMAN	0.38653946	1.560883
sp|P21266|GSTM3_HUMAN	0.39004135	2.0272346
sp|P13671|CO6_HUMAN	0.39025116	1.9975306
sp|P01024|CO3_HUMAN	0.39404106	15.176438
sp|O15127|SCAM2_HUMAN	0.39644623	1.6960104
sp|P01008|ANT3_HUMAN	0.39755058	3.5678737
sp|P01023|A2MG_HUMAN	0.4022045	7.3725133
sp|Q14767|LTBP2_HUMAN	0.40995407	4.717375
sp|O75937|DNJC8_HUMAN	0.42467117	2.1818786
sp|P13716-2|HEM2_HUMAN	0.43418312	1.6832623
sp|P04264|K2C1_HUMAN	0.4428978	12.437526
sp|Q9UBX5|FBLN5_HUMAN	0.4511795	2.6576471
sp|Q8N2S1-3|LTBP4_HUMAN	0.45837402	2.4402673
sp|P67936-2|TPM4_HUMAN	0.4607849	1.6997428
sp|P02766|TTHY_HUMAN	0.4621868	1.3815327
sp|P04196|HRG_HUMAN	0.4672985	2.987788
sp|P07451|CAH3_HUMAN	0.469553	1.827201
sp|P01876|IGHA1_HUMAN	0.4720955	3.0251918
sp|P09493-8|TPM1_HUMAN	0.47280884	2.432837
sp|Q01995|TAGL_HUMAN	0.49588966	4.6997666
sp|P08670|VIME_HUMAN	0.498312	14.423111

sp|P05546|HEP2_HUMAN	0.50211525	2.4982266
sp|P01042-2|KNG1_HUMAN	0.5089836	4.7071037
sp|Q9UMS6-2|SYNP2_HUMAN	0.5150337	2.653196
sp|P08603|CFAH_HUMAN	0.5193043	13.473147
sp|P08727|K1C19_HUMAN	0.5260792	5.644248
sp|Q15102|PA1B3_HUMAN	0.52825356	1.7590232
sp|P07585|PGS2_HUMAN	0.5282879	4.4994555
sp|P30043|BLVRB_HUMAN	0.5303421	1.3815327
sp|P00918|CAH2_HUMAN	0.530674	4.534284
sp|Q96PD5-2|PGRP2_HUMAN	0.53073883	1.6960104
sp|P29622|KAIN_HUMAN	0.55370903	2.1818786
sp|P19827|ITIH1_HUMAN	0.56381035	1.827201
sp|P06727|APOA4_HUMAN	0.57061386	5.523198
sp|P20774|MIME_HUMAN	0.59256077	3.3668416
sp|P09493-9|TPM1_HUMAN	0.6567993	1.6960104
sp|P51884|LUM_HUMAN	0.65681076	7.7561703
sp|P31947-2|1433S_HUMAN	0.67071915	1.4716977
sp|P07738|PMGE_HUMAN	0.6928406	2.1818786
sp|Q15063-3|POSTN_HUMAN	0.70222855	6.936907
sp|P05783|K1C18_HUMAN	0.71536255	8.532816
sp|Q8WU39|MZB1_HUMAN	0.72480774	1.6960104
sp|P32119|PRDX2_HUMAN	0.74201584	4.964395
sp|P27169|PON1_HUMAN	0.87080765	1.6960104
sp|P04114|APOB_HUMAN	0.93634224	11.791697
sp|Q05707-2|COEA1_HUMAN	0.941391	14.875408
sp|P17661|DESM_HUMAN	0.99001694	14.675837
sp|P00915|CAH1_HUMAN	1.0056343	6.253077
sp|P02042|HBD_HUMAN	1.022974	3.5908275
sp|P02647|APOA1_HUMAN	1.1238194	13.320112
sp|P02652|APOA2_HUMAN	1.2140732	2.9807727
sp|P68871|HBB_HUMAN	1.3070164	4.051346
sp|P08123|CO1A2_HUMAN	1.4247503	2.6576471
sp|P02452|CO1A1_HUMAN	1.4977016	2.1800864
sp|P69905|HBA_HUMAN	1.5590076	3.5908275
sp|P04229|2B11_HUMAN	-2.6177425	0.7827403
sp|Q04826|1B40_HUMAN	-2.2847443	0.656254
sp|P13761|2B17_HUMAN	-1.9574509	0.656254
sp|P13760|2B14_HUMAN	-1.4635582	0.91601294
sp|P08246|ELNE_HUMAN	-1.156393	1.1932944
sp|P10316|1A69_HUMAN	-1.0575752	0.656254
sp|P25815|S100P_HUMAN	-0.8416023	0.656254
sp|P30453|1A34_HUMAN	-0.7585144	0.656254
sp|Q03135-2|CAV1_HUMAN	-0.7307243	0.656254
sp|P41218|MNDA_HUMAN	-0.6715603	0.7588735
sp|P49913|CAMP_HUMAN	-0.6248436	1.1932944

sp|P15559-3|NQO1_HUMAN	-0.571106	0.91601294
sp|Q14956-2|GPNMB_HUMAN	-0.5528946	1.1932944
sp|Q96AP7|ESAM_HUMAN	-0.5377541	0.7827403
sp|Q9BXM0|PRAX_HUMAN	-0.5347652	0.45033538
sp|Q92522|H1X_HUMAN	-0.4904251	1.1932944
sp|Q07075|AMPE_HUMAN	-0.4777718	0.5204253
sp|P13686|PPA5_HUMAN	-0.4512615	0.7061832
sp|P37235|HPCL1_HUMAN	-0.4495316	0.656254
sp|P00167-2|CYB5_HUMAN	-0.4426498	0.7061832
sp|P55290-4|CAD13_HUMAN	-0.4328804	0.7061832
sp|Q10589-2|BST2_HUMAN	-0.4305191	1.1932944
sp|Q9NZN3|EHD3_HUMAN	-0.3933554	0.7061832
sp|Q9NPY3|C1QR1_HUMAN	-0.3917694	0.97209185
sp|P06703|S10A6_HUMAN	-0.3906746	0.35795313
sp|P63218|GBG5_HUMAN	-0.3845348	0.97209185
sp|P24158|PRTN3_HUMAN	-0.3808861	0.7061832
sp|O95716|RAB3D_HUMAN	-0.3805027	0.656254
sp|P04908|H2A1B_HUMAN	-0.3765888	0.656254
sp|P18428|LBP_HUMAN	-0.3648052	0.7588735
sp|P16671-4|CD36_HUMAN	-0.3615761	0.8983557
sp|P09758|TACD2_HUMAN	-0.356247	1.120602
sp|Q03135|CAV1_HUMAN	-0.352932	0.6298893
sp|O95837|GNA14_HUMAN	-0.3483887	0.656254
sp|P33151|CADH5_HUMAN	-0.3418236	0.35795313
sp|P02743|SAMP_HUMAN	-0.3396702	0.37060758
sp|Q16777|H2A2C_HUMAN	-0.3395596	0.656254
sp|P51153|RAB13_HUMAN	-0.3281403	0
sp|P02750|A2GL_HUMAN	-0.3270435	0.65625405
sp|P49407-2|ARRB1_HUMAN	-0.3249893	0.8059303
sp|P20160|CAP7_HUMAN	-0.324501	1.1505735
sp|Q8WWI1-5|LMO7_HUMAN	-0.3186894	0.45720983
sp|P99999|CYC_HUMAN	-0.3171883	0.52598757
sp|P25774|CATS_HUMAN	-0.3110886	0.7827403
sp|Q99715-4|COCA1_HUMAN	-0.3090267	0.69308156
sp|Q9H8H3|MET7A_HUMAN	-0.3041687	0
sp|Q53FA7|QORX_HUMAN	-0.3010483	0.19149946
sp|P09429|HMGB1_HUMAN	-0.3004284	0.45033538
sp|O43615|TIM44_HUMAN	-0.2971563	0.45033538
sp|P20702|ITAX_HUMAN	-0.2964859	0.312067
sp|P09601|HMOX1_HUMAN	-0.2961636	1.0301651
sp|P06753|TPM3_HUMAN	-0.2957573	0.656254
sp|P05109|S10A8_HUMAN	-0.2935581	0
sp|P11233|RALA_HUMAN	-0.2903061	0.35795313
sp|P62070-4|RRAS2_HUMAN	-0.2899017	0
sp|Q08722-2|CD47_HUMAN	-0.2858563	0.7061832

sp|Q6P4A8|PLBL1_HUMAN	-0.2758942	0.5204253
sp|P23229-4|ITA6_HUMAN	-0.2708569	0
sp|P11279|LAMP1_HUMAN	-0.2640305	1.1932944
sp|Q9UBR2|CATZ_HUMAN	-0.2583561	0
sp|Q15286-2|RAB35_HUMAN	-0.2580032	0
sp|Q9UM07|PADI4_HUMAN	-0.2550411	0.5204253
sp|P33121-3|ACSL1_HUMAN	-0.2547607	0.6509351
sp|Q9H223|EHD4_HUMAN	-0.2436485	0.9847622
sp|P07910-2|HNRPC_HUMAN	-0.2431908	1.1791906
sp|Q9NX46|ARHL2_HUMAN	-0.2430897	0.45033538
sp|P43304|GPDM_HUMAN	-0.2405071	0.26737198
sp|P13498|CY24A_HUMAN	-0.2403488	0.45033538
sp|P11215-2|ITAM_HUMAN	-0.239502	0.65625405
sp|P01009|A1AT_HUMAN	-0.2387085	1.0809385
sp|P56134-3|ATPK_HUMAN	-0.2385349	0
sp|Q9UBQ0-2|VPS29_HUMAN	-0.2363052	0.45033538
sp|P28676|GRAN_HUMAN	-0.2357407	0.1342476
sp|P17213|BPI_HUMAN	-0.2332134	0
sp|P21397|AOFA_HUMAN	-0.2287598	0.34853485
sp|Q92747|ARC1A_HUMAN	-0.2270966	0
sp|Q15126|PMVK_HUMAN	-0.220541	0.45033538
sp|Q02318|CP27A_HUMAN	-0.2205219	0
sp|Q9H2U2-2|IPYR2_HUMAN	-0.2185535	0.19149946
sp|P05107|ITB2_HUMAN	-0.2167511	0.37410614
sp|P48735|IDHP_HUMAN	-0.2166233	0.08307569
sp|P61019|RAB2A_HUMAN	-0.2162361	0.2178309
sp|O95994|AGR2_HUMAN	-0.2161255	0.26737198
sp|P14780|MMP9_HUMAN	-0.2156277	0.48520416
sp|P63220|RS21_HUMAN	-0.2119122	0
sp|Q13813-3|SPTN1_HUMAN	-0.2105942	0
sp|P20340-2|RAB6A_HUMAN	-0.2096367	0
sp|O15230|LAMA5_HUMAN	-0.2090282	0.59872687
sp|Q15599-2|NHRF2_HUMAN	-0.2073498	0
sp|P07355|ANXA2_HUMAN	-0.2068405	0
sp|Q13231-2|CHIT1_HUMAN	-0.2052994	0
sp|P15153|RAC2_HUMAN	-0.2044334	0.656254
sp|Q9NVJ2|ARL8B_HUMAN	-0.2029686	0
sp|P43490|NAMPT_HUMAN	-0.201683	0.290567
sp|Q9BXP5-5|SRRT_HUMAN	-0.2015801	0.45563722
sp|Q9Y2J2-4|E41L3_HUMAN	-0.2011318	0
sp|P13987-2|CD59_HUMAN	-0.1987686	1.1932944
sp|Q9BTZ2|DHRS4_HUMAN	-0.1922302	0.35795313
sp|P04179-4|SODM_HUMAN	-0.1915474	1.3006523
sp|Q6PIU2-2|NCEH1_HUMAN	-0.1912212	0.7498006
sp|Q9H0U4|RAB1B_HUMAN	-0.1898956	0.30372584

sp|P09871|C1S_HUMAN	-0.1851311	0.21439649
sp|P19525-2|E2AK2_HUMAN	-0.1837769	0
sp|P07942|LAMB1_HUMAN	-0.1778374	0.4566054
sp|P04004|VTNC_HUMAN	-0.1770763	0.23541966
sp|O75923-15|DYSF_HUMAN	-0.176199	0
sp|A0A0B4J1X8|HV343_HUMAN	-0.1757622	0.656254
sp|P05023-4|AT1A1_HUMAN	-0.1746635	0.33162096
sp|Q9HDC9|APMAP_HUMAN	-0.1734428	0.56639653
sp|Q00839|HNRPU_HUMAN	-0.1733227	0.21680334
sp|P13284|GILT_HUMAN	-0.171484	0.45033538
sp|Q96EE3-1|SEH1_HUMAN	-0.1711922	0
sp|Q14344|GNA13_HUMAN	-0.1709042	0.06291623
sp|P62873|GBB1_HUMAN	-0.1708813	0
sp|P05091|ALDH2_HUMAN	-0.1705856	0.01115908
sp|P12236|ADT3_HUMAN	-0.1701927	0.19149946
sp|P51648-2|AL3A2_HUMAN	-0.1696701	0.37060758
sp|P30838|AL3A1_HUMAN	-0.1693993	1.1932944
sp|O15296|LX15B_HUMAN	-0.1693955	0
sp|P30740|ILEU_HUMAN	-0.1671524	0
sp|Q14011|CIRBP_HUMAN	-0.1661167	0.19149946
sp|P38159|RBMX_HUMAN	-0.1635819	1.1932944
sp|O94760|DDAH1_HUMAN	-0.1633377	0
sp|P22307-8|NLTP_HUMAN	-0.1632214	0.91272414
sp|Q9UFN0|NPS3A_HUMAN	-0.163002	0.61034113
sp|Q02083-2|NAAA_HUMAN	-0.1626244	0
sp|Q9NZN4|EHD2_HUMAN	-0.1615982	0.8817905
sp|P51636-2|CAV2_HUMAN	-0.1593514	0.19149946
sp|O95573|ACSL3_HUMAN	-0.1586475	0
sp|P36957|ODO2_HUMAN	-0.1583786	0
sp|Q15907|RB11B_HUMAN	-0.1563931	0.02662771
sp|P05141|ADT2_HUMAN	-0.1560822	0.26737198
sp|Q92597|NDRG1_HUMAN	-0.1556244	0.06291623
sp|Q13813-2|SPTN1_HUMAN	-0.1550064	0.35795313
sp|P61224|RAP1B_HUMAN	-0.154829	0
sp|P45880-2|VDAC2_HUMAN	-0.1546593	0.22702569
sp|Q06830|PRDX1_HUMAN	-0.1530838	0.06210651
sp|Q6YHK3-4|CD109_HUMAN	-0.1530495	0.09894868
sp|Q9HD89|RETN_HUMAN	-0.1520729	0.19149946
sp|P61421|VA0D1_HUMAN	-0.1515675	0
sp|O75955-2|FLOT1_HUMAN	-0.1510868	0.02174174
sp|P30044|PRDX5_HUMAN	-0.1506081	0.05621411
sp|O60504-2|VINEX_HUMAN	-0.150547	0
sp|P51452-2|DUS3_HUMAN	-0.1500263	0
sp|P11717|MPRI_HUMAN	-0.1487541	0.2178309
sp|P08575-10|PTPRC_HUMAN	-0.1481094	0.21680334

sp|P23381|SYWC_HUMAN	-0.1479664	0.42828327
sp|P98160|PGBM_HUMAN	-0.146904	1.1440369
sp|Q16658|FSCN1_HUMAN	-0.1455631	0.37554002
sp|P30048-2|PRDX3_HUMAN	-0.1449051	0
sp|Q8N335|GPD1L_HUMAN	-0.1446495	0.30372584
sp|O75390|CISY_HUMAN	-0.1439781	0
sp|P51149|RAB7A_HUMAN	-0.1429119	0.24613085
sp|Q9UL25|RAB21_HUMAN	-0.1407414	0.06291623
sp|Q99536|VAT1_HUMAN	-0.1402054	0.01819076
sp|O15143|ARC1B_HUMAN	-0.139822	0
sp|P09110|THIK_HUMAN	-0.1374493	0.47871065
sp|Q7Z406-2|MYH14_HUMAN	-0.1374455	1.1815972
sp|Q5JWF2-2|GNAS1_HUMAN	-0.1352158	0.09339783
sp|P09619|PGFRB_HUMAN	-0.1345196	0.30372584
sp|Q00577|PURA_HUMAN	-0.1343765	0.45563722
sp|P26447|S10A4_HUMAN	-0.1316738	0.09339783
sp|Q86X76-2|NIT1_HUMAN	-0.1315212	0
sp|Q9Y277-2|VDAC3_HUMAN	-0.1308136	0.48520416
sp|P11310-2|ACADM_HUMAN	-0.1304321	0.3298487
sp|P00505|AATM_HUMAN	-0.1280289	0.06876189
sp|O00757|F16P2_HUMAN	-0.1264935	0
sp|P42330|AK1C3_HUMAN	-0.1263981	0
sp|Q04837|SSBP_HUMAN	-0.1252232	0.2178309
sp|Q32MZ4-3|LRRF1_HUMAN	-0.1245537	0.1558116
sp|P61106|RAB14_HUMAN	-0.1234436	0.02347357
sp|P42126-2|ECI1_HUMAN	-0.1222305	0.09894868
sp|Q53GQ0|DHB12_HUMAN	-0.1214123	0.33495146
sp|O94905|ERLN2_HUMAN	-0.1205406	0
sp|P08648|ITA5_HUMAN	-0.1194725	0
sp|P07686|HEXB_HUMAN	-0.1190796	0.74639726
sp|P08572|CO4A2_HUMAN	-0.1189308	0.56808305
sp|O00423-3|EMAL1_HUMAN	-0.1187458	0
sp|P67809|YBOX1_HUMAN	-0.1177521	0
sp|P06899|H2B1J_HUMAN	-0.1168289	0.7827403
sp|P24557-2|THAS_HUMAN	-0.1149826	0.19149946
sp|Q07157|ZO1_HUMAN	-0.114378	0.8473529
sp|P47897|SYQ_HUMAN	-0.1136436	0.04454162
sp|P0DOX2|IGA2_HUMAN	-0.11343	0.45033538
sp|P40926|MDHM_HUMAN	-0.1107864	0.03984664
sp|Q05682-4|CALD1_HUMAN	-0.1103745	0
sp|Q9UPQ0-3|LIMC1_HUMAN	-0.1096535	0
sp|P84095|RHOG_HUMAN	-0.1090698	0.35795313
sp|Q9NV96-3|CC50A_HUMAN	-0.1090374	0
sp|P61769|B2MG_HUMAN	-0.1085701	0
sp|Q8IWL2-2|SFTA1_HUMAN	-0.1085358	0.91072154

sp|P01920|DQB1_HUMAN	-0.106678	0
sp|O43813|LANC1_HUMAN	-0.1066208	0
sp|P06737-2|PYGL_HUMAN	-0.106617	0.06644611
sp|O43707|ACTN4_HUMAN	-0.1066151	0.01698457
sp|Q16363-2|LAMA4_HUMAN	-0.10495	0.595364
sp|Q9UPN3|MACF1_HUMAN	-0.1043549	0
sp|P00367|DHE3_HUMAN	-0.1040497	0.34244674
sp|Q09666|AHNK_HUMAN	-0.1039734	0.20721748
sp|Q00325-2|MPCP_HUMAN	-0.1039505	0.20467198
sp|P62879|GBB2_HUMAN	-0.103262	0
sp|P62993|GRB2_HUMAN	-0.1018829	0
sp|P78347-2|GTF2I_HUMAN	-0.1018448	0.19149946
sp|Q99729-2|ROAA_HUMAN	-0.1018353	0
sp|P10515|ODP2_HUMAN	-0.1005497	0
sp|Q9HB40|RISC_HUMAN	-0.1003399	0.19149946
sp|Q13451|FKBP5_HUMAN	-0.1000557	0
sp|Q13011|ECH1_HUMAN	-0.0998497	0.33495146
sp|P63167|DYL1_HUMAN	-0.0989018	0
sp|P12694-2|ODBA_HUMAN	-0.098733	0
sp|P06396-2|GELS_HUMAN	-0.0987225	0
sp|Q9NNW7|TRXR2_HUMAN	-0.0985985	0.21439649
sp|Q6P1N9|TATD1_HUMAN	-0.0981407	0
sp|Q5T440|CAF17_HUMAN	-0.0978546	0.09894868
sp|P10599-2|THIO_HUMAN	-0.0970917	0.06291623
sp|P09960|LKHA4_HUMAN	-0.0939026	0.40484723
sp|Q9ULV4-3|COR1C_HUMAN	-0.0931721	0
sp|P42167-2|LAP2B_HUMAN	-0.0924606	0.51870745
sp|O75367-2|H2AY_HUMAN	-0.0920334	0.8675019
sp|O43760-2|SNG2_HUMAN	-0.0913429	0
sp|Q8NBX0|SCPDL_HUMAN	-0.0910492	0
sp|P50148|GNAQ_HUMAN	-0.090477	0.06291623
sp|Q7Z4W1|DCXR_HUMAN	-0.089365	0
sp|Q969H8|MYDGF_HUMAN	-0.0891743	0
sp|Q9NPJ3-2|ACO13_HUMAN	-0.0891399	0
sp|P04080|CYTB_HUMAN	-0.0887718	0
sp|P60709|ACTB_HUMAN	-0.0871487	0
sp|O15247|CLIC2_HUMAN	-0.0865593	0
sp|P16219|ACADS_HUMAN	-0.086134	0.06291623
sp|P61586|RHOA_HUMAN	-0.0856733	0
sp|P22033|MUTA_HUMAN	-0.0834789	0
sp|Q9UDY2-3|ZO2_HUMAN	-0.0830059	0.356152
sp|P43353-2|AL3B1_HUMAN	-0.082758	0
sp|Q8NFW8|NEUA_HUMAN	-0.0822811	0
sp|P62714|PP2AB_HUMAN	-0.08218	0
sp|P09467|F16P1_HUMAN	-0.0812511	0.08684197

sp|Q16762|THTR_HUMAN	-0.0808868	0
sp|P51608|MECP2_HUMAN	-0.0799484	0.45033538
sp|P29218|IMPA1_HUMAN	-0.079937	0.04454162
sp|Q9Y2Q5|LTOR2_HUMAN	-0.0797033	0.19149946
sp|Q9HCC0-2|MCCB_HUMAN	-0.0793953	0
sp|Q04941|PLP2_HUMAN	-0.0790253	0
sp|P01111|RASN_HUMAN	-0.0788479	0
sp|Q96MM6|HS12B_HUMAN	-0.0780754	0.5176613
sp|Q96RQ3|MCCA_HUMAN	-0.0778961	0
sp|Q9H4M9|EHD1_HUMAN	-0.0778694	0
sp|P61604|CH10_HUMAN	-0.0772438	0
sp|P23634-2|AT2B4_HUMAN	-0.0772076	0
sp|P59998|ARPC4_HUMAN	-0.0768776	0.09339783
sp|O94925-3|GLSK_HUMAN	-0.0763054	0
sp|Q9NZM1-3|MYOF_HUMAN	-0.0762882	0
sp|P04839|CY24B_HUMAN	-0.0760574	0
sp|P30050|RL12_HUMAN	-0.0759697	0.26737198
sp|P49593|PPM1F_HUMAN	-0.075572	0
sp|Q9NP72-2|RAB18_HUMAN	-0.0751934	0.21439649
sp|P0DP25|CALM3_HUMAN	-0.0751019	0
sp|Q6P179|ERAP2_HUMAN	-0.0742302	0
sp|P50213|IDH3A_HUMAN	-0.0742111	0
sp|P61081|UBC12_HUMAN	-0.0734367	0
sp|Q02218-2|ODO1_HUMAN	-0.0731201	0
sp|P29992|GNA11_HUMAN	-0.0725842	0
sp|P63010-2|AP2B1_HUMAN	-0.0725155	0
sp|P62820|RAB1A_HUMAN	-0.0709534	0
sp|Q14254|FLOT2_HUMAN	-0.070282	0.05681695
sp|P60228|EIF3E_HUMAN	-0.0699692	0.30372584
sp|P67775-2|PP2AA_HUMAN	-0.0672531	0
sp|P04075|ALDOA_HUMAN	-0.0670624	0.04788631
sp|Q8IWB7|WDFY1_HUMAN	-0.0669003	0
sp|P23284|PPIB_HUMAN	-0.0661087	0
sp|P53041|PPP5_HUMAN	-0.0659714	0.09339783
sp|P42765|THIM_HUMAN	-0.0656052	0.16150911
sp|Q9UBW8|CSN7A_HUMAN	-0.0651398	0
sp|Q96EM0|T3HPD_HUMAN	-0.0645275	0
sp|P36543-2|VATE1_HUMAN	-0.0641708	0.09894868
sp|Q02252-2|MMSA_HUMAN	-0.0635395	0.19149946
sp|Q6P587-3|FAHD1_HUMAN	-0.0631809	0.09894868
sp|Q16698-2|DECR_HUMAN	-0.0631199	0.25761655
sp|P51553-2|IDH3G_HUMAN	-0.0630798	0
sp|P52788|SPSY_HUMAN	-0.0630493	0.7061832
sp|P17931|LEG3_HUMAN	-0.0627365	0
sp|Q13232|NDK3_HUMAN	-0.0620251	0

sp|P00488|F13A_HUMAN	-0.0616589	0
sp|P10301|RRAS_HUMAN	-0.0607433	0.2178309
sp|Q9P2R7-2|SUCB1_HUMAN	-0.0583572	0
sp|Q13938-4|CAYP1_HUMAN	-0.058342	0
sp|Q96TC7|RMD3_HUMAN	-0.0578175	0
sp|O43684-2|BUB3_HUMAN	-0.0575161	0
sp|P37837|TALDO_HUMAN	-0.0571766	0.02575658
sp|P01903|DRA_HUMAN	-0.0571518	0.10017801
sp|P27695|APEX1_HUMAN	-0.0570107	0
sp|P52565|GDIR1_HUMAN	-0.0567131	0.02174174
sp|Q8N9N7|LRC57_HUMAN	-0.056675	0
sp|Q13404|UB2V1_HUMAN	-0.0565834	0
sp|O14786|NRP1_HUMAN	-0.0562477	0.09894868
sp|P06865|HEXA_HUMAN	-0.0561848	0.40256184
sp|P54136|SYRC_HUMAN	-0.0553188	0
sp|P09382|LEG1_HUMAN	-0.0550194	0
sp|P61158|ARP3_HUMAN	-0.0540199	0.21807808
sp|O15173-2|PGRC2_HUMAN	-0.0530396	0.09750395
sp|P21980|TGM2_HUMAN	-0.0525208	0.01949666
sp|O95571|ETHE1_HUMAN	-0.0523853	0.26546443
sp|P22695|QCR2_HUMAN	-0.0522156	0
sp|P51649-2|SSDH_HUMAN	-0.0520935	0
sp|Q9BXS5-2|AP1M1_HUMAN	-0.05126	0
sp|P63261|ACTG_HUMAN	-0.0491333	0
sp|Q02978|M2OM_HUMAN	-0.0478115	0.19410844
sp|Q10567-2|AP1B1_HUMAN	-0.0477428	0.21439649
sp|P61160|ARP2_HUMAN	-0.0475006	0
sp|P50995-2|ANX11_HUMAN	-0.0466366	0.10328458
sp|Q15181|IPYR_HUMAN	-0.0463409	0
sp|P28074|PSB5_HUMAN	-0.0457764	0
sp|P05362|ICAM1_HUMAN	-0.0457478	0.01551361
sp|Q9NSD9|SYFB_HUMAN	-0.0456467	0
sp|Q9Y6N5|SQOR_HUMAN	-0.0455418	0.09215608
sp|O60814|H2B1K_HUMAN	-0.0445538	0.45033538
sp|P09211|GSTP1_HUMAN	-0.0441055	0.17871295
sp|P16435|NCPR_HUMAN	-0.0438252	0.02114108
sp|P41091|IF2G_HUMAN	-0.0414162	0
sp|Q08209-2|PP2BA_HUMAN	-0.0412712	0
sp|Q15369-2|ELOC_HUMAN	-0.0408573	0
sp|Q15366-2|PCBP2_HUMAN	-0.0406456	0
sp|O75643|U520_HUMAN	-0.0404797	0
sp|Q16629-4|SRSF7_HUMAN	-0.0399265	0.1342476
sp|Q9NQC3|RTN4_HUMAN	-0.0399132	0.4075265
sp|Q6NUK1-2|SCMC1_HUMAN	-0.039896	0.17511293
sp|O75695|XRP2_HUMAN	-0.0397873	0.2178309

sp|Q92973-2|TNPO1_HUMAN	-0.0393868	0.09894868
sp|P35237|SPB6_HUMAN	-0.0389004	0.00764478
sp|P04899-3|GNAI2_HUMAN	-0.0388412	0
sp|O75608-2|LYPA1_HUMAN	-0.0381641	0
sp|P10809|CH60_HUMAN	-0.037878	0.00415907
sp|O43747-2|AP1G1_HUMAN	-0.037508	0
sp|Q5EBM0-3|CMPK2_HUMAN	-0.0375061	0
sp|P07099|HYEP_HUMAN	-0.0374985	0.07020342
sp|P60900|PSA6_HUMAN	-0.0364418	0
sp|Q6UVK1|CSPG4_HUMAN	-0.035902	0.06986027
sp|P62258|1433E_HUMAN	-0.035635	0
sp|P01116-2|RASK_HUMAN	-0.0354443	0
sp|P15121|ALDR_HUMAN	-0.035429	0
sp|P40121|CAPG_HUMAN	-0.035202	0
sp|Q6YN16|HSDL2_HUMAN	-0.0347013	0.02662771
sp|P49327|FAS_HUMAN	-0.0344219	0.03060993
sp|Q16853|AOC3_HUMAN	-0.0342922	0.01542275
sp|O00391|QSOX1_HUMAN	-0.0342007	0.06291623
sp|P21796|VDAC1_HUMAN	-0.0341911	0
sp|P06744|G6PI_HUMAN	-0.0334797	0
sp|Q96N66-3|MBOA7_HUMAN	-0.0333996	0
sp|Q04917|1433F_HUMAN	-0.0333157	0
sp|P16278-2|BGAL_HUMAN	-0.0328274	0.09894868
sp|O60437|PEPL_HUMAN	-0.0321579	0.04344815
sp|O75521-2|ECI2_HUMAN	-0.03162	0
sp|Q86Y82|STX12_HUMAN	-0.0311699	0.20467198
sp|Q04760-2|LGUL_HUMAN	-0.03092	0
sp|P39060-1|COIA1_HUMAN	-0.0303364	0.3005443
sp|Q96AC1|FERM2_HUMAN	-0.029604	0.1549097
sp|P46063|RECQ1_HUMAN	-0.0288677	0
sp|O75369-2|FLNB_HUMAN	-0.0280018	0.04946801
sp|P53634|CATC_HUMAN	-0.0277901	0.06291623
sp|Q96FV2-2|SCRN2_HUMAN	-0.0271988	0
sp|P19474|RO52_HUMAN	-0.0268974	0
sp|P02795|MT2_HUMAN	-0.0268574	0
sp|P43034|LIS1_HUMAN	-0.0266228	0
sp|Q12931-2|TRAP1_HUMAN	-0.0264473	0
sp|O00231-2|PSD11_HUMAN	-0.0253716	0.24097534
sp|Q9H845|ACAD9_HUMAN	-0.0251465	0
sp|O95425-4|SVIL_HUMAN	-0.02458	0
sp|Q8IUZ5|AT2L2_HUMAN	-0.0245037	0
sp|P24752|THIL_HUMAN	-0.0230942	0
sp|P49748-2|ACADV_HUMAN	-0.023037	0
sp|P38646|GRP75_HUMAN	-0.0230274	0.12274049
sp|P49902-2|5NTC_HUMAN	-0.0229912	0

sp|Q96I99|SUCB2_HUMAN	-0.0228519	0.01073991
sp|P17858-2|PFKAL_HUMAN	-0.0227928	0.20174193
sp|P43121|MUC18_HUMAN	-0.0224133	0
sp|P15311|EZRI_HUMAN	-0.0219498	0
sp|Q9UQ80|PA2G4_HUMAN	-0.0217819	0
sp|Q969X5|ERGI1_HUMAN	-0.0212288	0
sp|O75323|NIPS2_HUMAN	-0.021183	0
sp|P08758|ANXA5_HUMAN	-0.0211067	0.01259616
sp|P35579|MYH9_HUMAN	-0.0209904	0.2094525
sp|P24539|AT5F1_HUMAN	-0.0206585	0
sp|A0A0B4J2D5|GAL3B_HUMAN	-0.0205364	0
sp|P12081-4|SYHC_HUMAN	-0.0201035	0
sp|P61960|UFM1_HUMAN	-0.0200462	0
sp|P35221|CTNA1_HUMAN	-0.0200386	0.02812482
sp|P55072|TERA_HUMAN	-0.0199585	0.05115994
sp|Q96CW1-2|AP2M1_HUMAN	-0.0198536	0
sp|O75439|MPPB_HUMAN	-0.0197163	0.1558116
sp|Q3KQV9|UAP1L_HUMAN	-0.0194626	0.19149946
sp|Q9UHL4|DPP2_HUMAN	-0.0192623	0
sp|P06702|S10A9_HUMAN	-0.0189247	0.1897449
sp|P51991|ROA3_HUMAN	-0.018899	0
sp|P04424-2|ARLY_HUMAN	-0.0187416	0
sp|O15400-2|STX7_HUMAN	-0.0184574	0
sp|O00483|NDUA4_HUMAN	-0.0172997	0.09894868
sp|P15170-2|ERF3A_HUMAN	-0.017025	0
sp|P09496-2|CLCA_HUMAN	-0.01618	0
sp|O15144|ARPC2_HUMAN	-0.0150414	0.19149946
sp|P27824-2|CALX_HUMAN	-0.0150185	0.06534385
sp|Q14165|MLEC_HUMAN	-0.0144615	0
sp|Q02750|MP2K1_HUMAN	-0.0143299	0
sp|Q9P2T1-2|GMPR2_HUMAN	-0.0137787	0
sp|P53597|SUCA_HUMAN	-0.0136414	0
sp|P38606-2|VATA_HUMAN	-0.0135651	0
sp|P46926|GNPI1_HUMAN	-0.0131416	0
sp|P63244|RACK1_HUMAN	-0.0123329	0
sp|P27105|STOM_HUMAN	-0.011404	0.1804695
sp|P04275|VWF_HUMAN	-0.0111637	0.02421166
sp|P49903-2|SPS1_HUMAN	-0.0106354	0
sp|Q02543|RL18A_HUMAN	-0.0104809	0
sp|Q7Z4I7-3|LIMS2_HUMAN	-0.0096493	0
sp|Q96I15|SCLY_HUMAN	-0.0094109	0
sp|Q13228-4|SBP1_HUMAN	-0.0091763	0.00148549
sp|P38919|IF4A3_HUMAN	-0.0090446	0
sp|P32456|GBP2_HUMAN	-0.0088711	0
sp|Q9P0V9-2|SEP10_HUMAN	-0.0088425	0.19410844

sp|Q00169|PIPNA_HUMAN	-0.0073109	0
sp|Q06136|KDSR_HUMAN	-0.006752	0
sp|Q9Y2Q3-3|GSTK1_HUMAN	-0.0065079	0
sp|Q15365|PCBP1_HUMAN	-0.0062275	0.18414244
sp|P13489|RINI_HUMAN	-0.0061684	0.00508175
sp|Q15056-2|IF4H_HUMAN	-0.0061264	0
sp|O95833|CLIC3_HUMAN	-0.005825	0.04454162
sp|O75534-2|CSDE1_HUMAN	-0.0044003	0
sp|P09543-2|CN37_HUMAN	-0.0041237	0
sp|Q14203-4|DCTN1_HUMAN	-0.0038528	0.07727676
sp|Q9Y6B6|SAR1B_HUMAN	-0.0036201	0
sp|P12109|CO6A1_HUMAN	-0.0033703	0.3905819
sp|Q29865|1C18_HUMAN	-0.0028801	0.09894868
sp|P24821-4|TENA_HUMAN	-0.0024757	0.13308895
sp|Q92598-2|HS105_HUMAN	-0.002121	0
sp|Q9BPW8|NIPS1_HUMAN	-0.0017624	0
sp|Q8TC12-2|RDH11_HUMAN	-0.0016689	0
sp|Q9NQR4|NIT2_HUMAN	-0.0013809	0
sp|P63000-2|RAC1_HUMAN	-0.0012589	0
sp|P17612|KAPCA_HUMAN	-0.0010223	0
sp|P35754|GLRX1_HUMAN	-5.57E-04	0
sp|P00387-3|NB5R3_HUMAN	-5.46E-04	0
sp|P28482|MK01_HUMAN	-4.84E-04	0
sp|Q07954|LRP1_HUMAN	-1.79E-04	0.05779712
sp|Q9HBL0|TENS1_HUMAN	6.81E-04	0.03357808
sp|P36551|HEM6_HUMAN	8.30E-04	0
sp|Q08211|DHX9_HUMAN	9.80E-04	0.07934754
sp|P14618-3|KPYM_HUMAN	0.00149727	0
sp|O75436|VP26A_HUMAN	0.00152397	0.06291623
sp|A6NMZ7|CO6A6_HUMAN	0.00166512	0.20281352
sp|P61163|ACTZ_HUMAN	0.00239754	0
sp|Q8IY17-3|PLPL6_HUMAN	0.00239754	0
sp|P11216|PYGB_HUMAN	0.00259972	0.15874931
sp|Q14558-2|KPRA_HUMAN	0.00291729	0
sp|Q15393|SF3B3_HUMAN	0.00319672	0.03630202
sp|P04406|G3P_HUMAN	0.0032177	0
sp|P12111|CO6A3_HUMAN	0.00324821	0.30513477
sp|O75083|WDR1_HUMAN	0.00363922	0
sp|O94919|ENDD1_HUMAN	0.00400162	0.06291623
sp|Q9BSJ8-2|ESYT1_HUMAN	0.00407028	0.01044662
sp|P32969|RL9_HUMAN	0.00432205	0.09894868
sp|P35222|CTNB1_HUMAN	0.00497437	0
sp|P23396|RS3_HUMAN	0.00514603	0
sp|Q05655-2|KPCD_HUMAN	0.00515747	0
sp|P62834|RAP1A_HUMAN	0.00518799	0

sp|P52790|HXK3_HUMAN	0.00534821	0
sp|Q9UNH7|SNX6_HUMAN	0.00569916	0
sp|O15498-2|YKT6_HUMAN	0.00575256	0
sp|P55010|IF5_HUMAN	0.00667	0
sp|P51178-2|PLCD1_HUMAN	0.0069561	0
sp|P46781|RS9_HUMAN	0.00741768	0.2207585
sp|Q9H2G2-2|SLK_HUMAN	0.00749588	0
sp|P08754|GNAI3_HUMAN	0.00764656	0
sp|Q14118|DAG1_HUMAN	0.0077877	0
sp|O00468-7|AGRIN_HUMAN	0.00798988	0.45033538
sp|Q14139-2|UBE4A_HUMAN	0.008564	0
sp|P49065|ALBU_RABIT	0.00875473	0
sp|Q13884|SNTB1_HUMAN	0.00881767	0.09894868
sp|P62942|FKB1A_HUMAN	0.00917816	0
sp|Q9NTX5-3|ECHD1_HUMAN	0.00949097	0.02662771
sp|P04083|ANXA1_HUMAN	0.01080132	0.00699881
sp|P46782|RS5_HUMAN	0.01151848	0.12897664
sp|B5ME19|EIFCL_HUMAN	0.01169014	0.05483269
sp|Q92734-2|TFG_HUMAN	0.0118866	0
sp|P01859|IGHG2_HUMAN	0.01242065	0.02174174
sp|O75874|IDHC_HUMAN	0.01261902	0
sp|Q9Y2A7-2|NCKP1_HUMAN	0.01264572	0
sp|Q13751|LAMB3_HUMAN	0.01329041	0.30372584
sp|P0CG39|POTEJ_HUMAN	0.0135231	0
sp|Q9H0W9-2|CK054_HUMAN	0.01432419	0
sp|P26038|MOES_HUMAN	0.01443863	0.00435698
sp|P50990|TCPQ_HUMAN	0.01478577	0.06941727
sp|Q13492-2|PICAL_HUMAN	0.01506233	0
sp|P61026|RAB10_HUMAN	0.01510239	0
sp|P48960-2|CD97_HUMAN	0.0154438	0
sp|P06576|ATPB_HUMAN	0.0158062	0.01100408
sp|P14866|HNRPL_HUMAN	0.01631928	0
sp|O75340-2|PDCD6_HUMAN	0.01663971	0
sp|Q9GZM7-3|TINAL_HUMAN	0.01736927	0
sp|P13804-2|ETFA_HUMAN	0.01768112	0
sp|P52272-2|HNRPM_HUMAN	0.01786041	0.2074024
sp|P05388-2|RLA0_HUMAN	0.01787186	0
sp|O75348|VATG1_HUMAN	0.01818085	0
sp|P09525|ANXA4_HUMAN	0.01832008	0
sp|Q9Y394-2|DHRS7_HUMAN	0.01836777	0
sp|Q9H9G7-2|AGO3_HUMAN	0.01861572	0
sp|Q13423|NNTM_HUMAN	0.01872826	0.14338206
sp|Q15005|SPCS2_HUMAN	0.01896286	0
sp|Q9H9B4|SFXN1_HUMAN	0.01897621	0
sp|P07996|TSP1_HUMAN	0.01931191	0.04454162

sp|Q9HB07|MYG1_HUMAN	0.01963806	0
sp|Q12797-10|ASPH_HUMAN	0.02005959	0.20467198
sp|Q9P0K7-2|RAI14_HUMAN	0.02087402	0
sp|P08779|K1C16_HUMAN	0.02138138	0
sp|Q86TX2|ACOT1_HUMAN	0.02140999	0
sp|P21964-2|COMT_HUMAN	0.02254868	0
sp|O00299|CLIC1_HUMAN	0.02430534	0
sp|P23246|SFPQ_HUMAN	0.02437782	0
sp|P07948-2|LYN_HUMAN	0.02453995	0
sp|P62316|SMD2_HUMAN	0.02456474	0
sp|O60256-3|KPRB_HUMAN	0.02458191	0
sp|Q9TQE0|2B19_HUMAN	0.02531815	0
sp|Q9H299|SH3L3_HUMAN	0.02545548	0
sp|P00403|COX2_HUMAN	0.02589035	0
sp|P23368|MAOM_HUMAN	0.02618027	0
sp|P41250|GARS_HUMAN	0.02626896	0.09851671
sp|P42025|ACTY_HUMAN	0.02687073	0
sp|P52209-2|6PGD_HUMAN	0.02689934	0
sp|Q8N5K1|CISD2_HUMAN	0.02707863	0.45033538
sp|Q00765|REEP5_HUMAN	0.0271225	0
sp|Q8WUP2-3|FBLI1_HUMAN	0.02734375	0
sp|P05198|IF2A_HUMAN	0.02742958	0
sp|P48449-3|ERG7_HUMAN	0.02744865	0.09339783
sp|P0DMV9|HS71B_HUMAN	0.02748871	0
sp|P09668|CATH_HUMAN	0.027565	0.20467198
sp|P14868|SYDC_HUMAN	0.02780724	0.01551361
sp|P30491|1B53_HUMAN	0.028759	0
sp|O95197-2|RTN3_HUMAN	0.02938271	0
sp|P54920|SNAA_HUMAN	0.02948189	0
sp|P29590|PML_HUMAN	0.0295639	0.00697919
sp|Q9UJ70-2|NAGK_HUMAN	0.02975655	0
sp|O43175|SERA_HUMAN	0.02996445	0.16077396
sp|Q15833-2|STXB2_HUMAN	0.03025818	0
sp|Q01130-2|SRSF2_HUMAN	0.03046417	0
sp|P13797|PLST_HUMAN	0.03051949	0
sp|P0DJI8|SAA1_HUMAN	0.03063965	0
sp|P15880|RS2_HUMAN	0.03068352	0.29066643
sp|Q5JTV8-3|TOIP1_HUMAN	0.03082085	0.21439649
sp|Q15836|VAMP3_HUMAN	0.03103638	0
sp|P17655|CAN2_HUMAN	0.03115273	0.39074486
sp|O14980|XPO1_HUMAN	0.0314064	0.6298893
sp|Q9NTJ5|SAC1_HUMAN	0.03169441	0
sp|P30040|ERP29_HUMAN	0.03192329	0.04454162
sp|P08134|RHOC_HUMAN	0.03199005	0
sp|P26196|DDX6_HUMAN	0.0321312	0

sp|O14828-2|SCAM3_HUMAN	0.03214073	0
sp|P07237|PDIA1_HUMAN	0.03288269	0.22529724
sp|P26639-2|SYTC_HUMAN	0.03307533	0
sp|Q6NY19-2|KANK3_HUMAN	0.03355789	0
sp|P11686-2|PSPC_HUMAN	0.03434372	0
sp|Q13185|CBX3_HUMAN	0.03453064	0.2178309
sp|O60234|GMFG_HUMAN	0.03465653	0.14672586
sp|P09497-2|CLCB_HUMAN	0.03475952	0
sp|P32455|GBP1_HUMAN	0.03506088	0.02174174
sp|P31040|SDHA_HUMAN	0.03513527	0.03712561
sp|P18206-2|VINC_HUMAN	0.03515816	0.03297596
sp|P31930|QCR1_HUMAN	0.03517342	0.0315703
sp|Q9BVG4|PBDC1_HUMAN	0.0354538	0
sp|P30626-2|SORCN_HUMAN	0.03548813	0
sp|P26641|EF1G_HUMAN	0.03574181	0.01576272
sp|O75688|PPM1B_HUMAN	0.03590393	0
sp|Q13162|PRDX4_HUMAN	0.03639984	0
sp|Q6UWY5|OLFL1_HUMAN	0.03668976	0.16702904
sp|P28072|PSB6_HUMAN	0.03678894	0
sp|Q14103-3|HNRPD_HUMAN	0.03725624	0.24660711
sp|P49458|SRP09_HUMAN	0.03726959	0
sp|Q13555-7|KCC2G_HUMAN	0.03734493	0
sp|Q99459|CDC5L_HUMAN	0.03734589	0
sp|Q99460-2|PSMD1_HUMAN	0.03762436	0
sp|P20073-2|ANXA7_HUMAN	0.03764916	0.34244674
sp|P61006|RAB8A_HUMAN	0.03779984	0
sp|P01019|ANGT_HUMAN	0.03803825	0
sp|P25705|ATPA_HUMAN	0.03844261	0.08325488
sp|P62330|ARF6_HUMAN	0.03918266	0
sp|P55196-5|AFAD_HUMAN	0.03976822	0
sp|Q9Y3I0|RTCB_HUMAN	0.03981972	0.1897449
sp|P23526|SAHH_HUMAN	0.04010773	0.02863072
sp|P06733|ENOA_HUMAN	0.04094505	0
sp|P53602|MVD1_HUMAN	0.04097557	0
sp|O00764-2|PDXK_HUMAN	0.04101372	0.02662771
sp|Q9Y6C2|EMIL1_HUMAN	0.04167175	0.01542275
sp|P08559-2|ODPA_HUMAN	0.04231262	0.1791607
sp|P47755|CAZA2_HUMAN	0.04243469	0
sp|P46736-3|BRCC3_HUMAN	0.04274368	0
sp|P02679-2|FIBG_HUMAN	0.04277802	0
sp|O00264|PGRC1_HUMAN	0.04297638	0
sp|A5A3E0|POTEF_HUMAN	0.04332256	0
sp|Q9UI12-2|VATH_HUMAN	0.04372025	0
sp|Q13636|RAB31_HUMAN	0.04442406	0
sp|Q8N392|RHG18_HUMAN	0.04458046	0.02174174

sp|P61981|1433G_HUMAN	0.04481697	0
sp|Q16531|DDB1_HUMAN	0.04485703	0.14321187
sp|O95352|ATG7_HUMAN	0.04512024	0
sp|Q8NBF2-2|NHLC2_HUMAN	0.04528999	0
sp|Q9ULC5-3|ACSL5_HUMAN	0.04535484	0
sp|P17844-2|DDX5_HUMAN	0.04536629	0
sp|Q96HC4|PDLI5_HUMAN	0.04553413	0.10017801
sp|P54619-2|AAKG1_HUMAN	0.0455513	0
sp|O75964|ATP5L_HUMAN	0.04594612	0
sp|P25398|RS12_HUMAN	0.0460968	0
sp|Q9NUV9|GIMA4_HUMAN	0.0460968	0
sp|P05166-2|PCCB_HUMAN	0.04623604	0
sp|Q16787-3|LAMA3_HUMAN	0.04623795	0
sp|P09972|ALDOC_HUMAN	0.04627228	0
sp|Q13418|ILK_HUMAN	0.04687119	0
sp|A1L0T0|ILVBL_HUMAN	0.04741478	0
sp|O43865|SAHH2_HUMAN	0.04747391	0.14672586
sp|Q14533|KRT81_HUMAN	0.04792976	0
sp|O43795-2|MYO1B_HUMAN	0.04884434	0.44572112
sp|O95865|DDAH2_HUMAN	0.04929733	0.1791607
sp|P12110|CO6A2_HUMAN	0.04941559	0.27953532
sp|P60174|TPIS_HUMAN	0.04993248	0.01949666
sp|P28070|PSB4_HUMAN	0.05032539	0.04454162
sp|O00186|STXB3_HUMAN	0.05043125	0
sp|P34897-3|GLYM_HUMAN	0.05061913	0.30372584
sp|Q9Y2X3|NOP58_HUMAN	0.05094719	0
sp|P36871|PGM1_HUMAN	0.05101204	0.12625508
sp|P23786|CPT2_HUMAN	0.05138779	0
sp|P09622|DLDH_HUMAN	0.05140686	0.04817784
sp|Q99829|CPNE1_HUMAN	0.05209351	0
sp|P58107|EPIPL_HUMAN	0.05236816	0.16702904
sp|P63096|GNAI1_HUMAN	0.0532074	0
sp|Q9H8L6|MMRN2_HUMAN	0.05323792	0.312067
sp|Q13217|DNJC3_HUMAN	0.05344582	0.17319627
sp|P62917|RL8_HUMAN	0.05354118	0.3005443
sp|P68036-3|UB2L3_HUMAN	0.05360603	0
sp|P12955|PEPD_HUMAN	0.05370331	0
sp|P0C0S5|H2AZ_HUMAN	0.05388069	0.656254
sp|Q9NVD7|PARVA_HUMAN	0.05441475	0.06986027
sp|P28066|PSA5_HUMAN	0.05455399	0.09894868
sp|Q9Y281|COF2_HUMAN	0.05474854	0
sp|O95202|LETM1_HUMAN	0.0548687	0.35193655
sp|Q14240-2|IF4A2_HUMAN	0.05547905	0
sp|Q16527|CSRP2_HUMAN	0.05551529	0
sp|P26440|IVD_HUMAN	0.0557785	0

sp|P02760|AMBP_HUMAN	0.05607224	0.03367973
sp|P49257|LMAN1_HUMAN	0.05617905	0
sp|P06748-3|NPM_HUMAN	0.05630112	0
sp|P62244|RS15A_HUMAN	0.05633354	0
sp|Q8WXH0-2|SYNE2_HUMAN	0.05664539	0
sp|Q6PCB0|VWA1_HUMAN	0.05681992	0
sp|O00291|HIP1_HUMAN	0.05726624	0
sp|P07900-2|HS90A_HUMAN	0.05734634	0.14132895
sp|P19367-3|HXK1_HUMAN	0.05799103	0.15079153
sp|P40227|TCPZ_HUMAN	0.05835342	0.02388411
sp|O60701|UGDH_HUMAN	0.05865097	0
sp|P84090|ERH_HUMAN	0.05871582	0
sp|Q9NSE4|SYIM_HUMAN	0.05878258	0.26665917
sp|Q9Y3C8|UFC1_HUMAN	0.0589695	0
sp|P62318-2|SMD3_HUMAN	0.05955124	0
sp|Q15942|ZYX_HUMAN	0.05965042	0.20404659
sp|O94973-2|AP2A2_HUMAN	0.0598526	0.28377196
sp|P78417|GSTO1_HUMAN	0.06028557	0.30819008
sp|P35232|PHB_HUMAN	0.06056786	0.3516469
sp|P61225|RAP2B_HUMAN	0.06067085	0
sp|Q8TD19|NEK9_HUMAN	0.06069374	0
sp|O95861-4|BPNT1_HUMAN	0.06074524	0
sp|P10155-3|RO60_HUMAN	0.06083679	0.2591514
sp|P10768|ESTD_HUMAN	0.06092072	0.26737198
sp|P17987|TCPA_HUMAN	0.06126213	0.03765291
sp|P12830|CADH1_HUMAN	0.06126976	0
sp|P60953|CDC42_HUMAN	0.06267929	0.06842031
sp|O95834|EMAL2_HUMAN	0.06279373	0
sp|Q9BS26|ERP44_HUMAN	0.06285858	0
sp|P11021|BIP_HUMAN	0.06287766	0.17886412
sp|P48681|NEST_HUMAN	0.06297112	0
sp|P68371|TBB4B_HUMAN	0.06346512	0
sp|O95831-3|AIFM1_HUMAN	0.06350136	0
sp|P02675|FIBB_HUMAN	0.06351662	0.12625508
sp|Q96EP5-2|DAZP1_HUMAN	0.06481934	0
sp|P48047|ATPO_HUMAN	0.06501389	0.21989624
sp|Q14112-2|NID2_HUMAN	0.06511307	0.02662771
sp|P84077|ARF1_HUMAN	0.06516647	0.06291623
sp|P35606-2|COPB2_HUMAN	0.06537628	0.19818917
sp|Q9H3U1-2|UN45A_HUMAN	0.06546783	0
sp|P09417|DHPR_HUMAN	0.06553268	0
sp|P22234-2|PUR6_HUMAN	0.06580734	0
sp|Q9UHQ9|NB5R1_HUMAN	0.0660286	0
sp|Q08431|MFGM_HUMAN	0.06607246	0.40256184
sp|P55735|SEC13_HUMAN	0.06641769	0

sp|P21283|VATC1_HUMAN	0.0669632	0
sp|P18077|RL35A_HUMAN	0.06792641	0.19149946
sp|P50453|SPB9_HUMAN	0.06805611	0.01343579
sp|P22352|GPX3_HUMAN	0.06806946	0
sp|P62906|RL10A_HUMAN	0.06838417	0
sp|P05165-2|PCCA_HUMAN	0.06858063	0
sp|Q9NZL9-2|MAT2B_HUMAN	0.06881332	0
sp|P14625|ENPL_HUMAN	0.06884956	0.2601681
sp|Q9UJW0-3|DCTN4_HUMAN	0.06891441	0
sp|Q15084-5|PDIA6_HUMAN	0.06954193	0.07131392
sp|Q9BUQ8|DDX23_HUMAN	0.06990624	0
sp|Q86WV6|STING_HUMAN	0.07000732	0
sp|P11766|ADHX_HUMAN	0.07005501	0.05387706
sp|P02794|FRIH_HUMAN	0.07018089	0
sp|Q7KZF4|SND1_HUMAN	0.07024574	0
sp|P18669|PGAM1_HUMAN	0.07079887	0.10280301
sp|Q9P2X0-2|DPM3_HUMAN	0.0708046	0
sp|P19338|NUCL_HUMAN	0.07111168	0.13374822
sp|O75746-2|CMC1_HUMAN	0.07116127	0
sp|P62826|RAN_HUMAN	0.0716629	0
sp|Q5SSJ5-2|HP1B3_HUMAN	0.07176971	0.14927356
sp|P27361|MK03_HUMAN	0.07177353	0
sp|Q02878|RL6_HUMAN	0.07178688	0
sp|O43491|E41L2_HUMAN	0.07218933	0.02174174
sp|Q9NX63|MIC19_HUMAN	0.0723381	0
sp|O43776|SYNC_HUMAN	0.07250977	0
sp|P56537|IF6_HUMAN	0.0730648	0.40256184
sp|P47985|UCRI_HUMAN	0.07312012	0.2178309
sp|P48643|TCPE_HUMAN	0.07312393	0.14871502
sp|Q96HE7|ERO1A_HUMAN	0.0735321	0.09894868
sp|O00567|NOP56_HUMAN	0.07359505	0
sp|P63151-2|2ABA_HUMAN	0.07365799	0
sp|P11413-3|G6PD_HUMAN	0.07382011	0.01343579
sp|Q9P258|RCC2_HUMAN	0.07383728	0
sp|P55795|HNRH2_HUMAN	0.07424736	0
sp|Q96C19|EFHD2_HUMAN	0.07498741	0.06291623
sp|Q96TA1-2|NIBL1_HUMAN	0.07521057	0
sp|P46976|GLYG_HUMAN	0.07565117	0.30372584
sp|Q14914-2|PTGR1_HUMAN	0.07600403	0
sp|Q8WUY1|THEM6_HUMAN	0.07616997	0.2178309
sp|Q99714|HCD2_HUMAN	0.07623673	0.16702904
sp|P98095-2|FBLN2_HUMAN	0.07711601	0.01551361
sp|P00338|LDHA_HUMAN	0.07722855	0
sp|P20339-2|RAB5A_HUMAN	0.07762528	0
sp|O95340-2|PAPS2_HUMAN	0.07837677	0

sp|P51148-2|RAB5C_HUMAN	0.07847786	0.20467198
sp|Q9BUJ2-4|HNRL1_HUMAN	0.078825	0.24097534
sp|Q15637-3|SF01_HUMAN	0.07902145	0
sp|P48059-3|LIMS1_HUMAN	0.07912064	0
sp|P21912|SDHB_HUMAN	0.07996178	0
sp|Q15185-3|TEBP_HUMAN	0.08051491	0
sp|Q15417|CNN3_HUMAN	0.08053017	0
sp|Q07866-10|KLC1_HUMAN	0.0806427	0
sp|P02792|FRIL_HUMAN	0.08101082	0
sp|P46777|RL5_HUMAN	0.08114433	0
sp|P20618|PSB1_HUMAN	0.08125496	0
sp|Q9Y3D6|FIS1_HUMAN	0.08141804	0
sp|Q9P2M7|CING_HUMAN	0.08184147	0
sp|P46108|CRK_HUMAN	0.08195305	0
sp|Q96BW5-2|PTER_HUMAN	0.08200073	0
sp|Q13308-6|PTK7_HUMAN	0.08230972	0.2207585
sp|Q16576-2|RBBP7_HUMAN	0.08239746	0
sp|Q9BR76|COR1B_HUMAN	0.08240509	0
sp|P47756-2|CAPZB_HUMAN	0.08270073	0.31786543
sp|Q15370-2|ELOB_HUMAN	0.08307648	0
sp|P15144|AMPN_HUMAN	0.08320046	0.35193655
sp|O43837|IDH3B_HUMAN	0.08327675	0.09894868
sp|Q13557-10|KCC2D_HUMAN	0.08366585	0
sp|Q8TBC4|UBA3_HUMAN	0.08375931	0
sp|Q08945|SSRP1_HUMAN	0.08408928	0.20467198
sp|Q9NRN5|OLFL3_HUMAN	0.08420563	0
sp|P30520|PURA2_HUMAN	0.08427429	0
sp|P19971|TYPH_HUMAN	0.08457947	0.9765129
sp|P55786|PSA_HUMAN	0.08464241	0.19276452
sp|Q9HAV0|GBB4_HUMAN	0.08483887	0
sp|Q02790|FKBP4_HUMAN	0.08503723	0.04454162
sp|P17301|ITA2_HUMAN	0.08506393	0
sp|Q9H3N1|TMX1_HUMAN	0.08511353	0
sp|O15372|EIF3H_HUMAN	0.08524513	0
sp|P36776-2|LONM_HUMAN	0.08535004	0
sp|Q13724-2|MOGS_HUMAN	0.08566856	0.06291623
sp|Q9UIJ7|KAD3_HUMAN	0.08631134	0
sp|P01860|IGHG3_HUMAN	0.08657455	0.14672586
sp|Q9H0E2|TOLIP_HUMAN	0.08667183	0.45033538
sp|Q9UNM6-2|PSD13_HUMAN	0.08668327	0
sp|P13073|COX41_HUMAN	0.08675385	0
sp|Q9Y3A5|SBDS_HUMAN	0.08681297	0.06291623
sp|Q92542|NICA_HUMAN	0.08685494	0.35795313
sp|Q86UX7-2|URP2_HUMAN	0.0874958	0.19852771
sp|P22392-2|NDKB_HUMAN	0.08755684	0.01819076

sp|Q01105-2|SET_HUMAN	0.08774757	0.35795313
sp|P26599-2|PTBP1_HUMAN	0.08837128	0
sp|P22897|MRC1_HUMAN	0.08843803	0.45546502
sp|Q53EL6-2|PDCD4_HUMAN	0.08864212	0
sp|P62913|RL11_HUMAN	0.08882713	0.2178309
sp|P08294|SODE_HUMAN	0.08921433	0
sp|Q9BZQ8|NIBAN_HUMAN	0.08931923	0
sp|P49189|AL9A1_HUMAN	0.08943939	0
sp|Q9Y3F4-2|STRAP_HUMAN	0.08987999	0
sp|Q8TAT6-2|NPL4_HUMAN	0.09014702	0
sp|Q93052|LPP_HUMAN	0.0903225	0
sp|Q01813|PFKAP_HUMAN	0.09037399	0
sp|Q9Y490|TLN1_HUMAN	0.09083176	0.31346214
sp|O95336|6PGL_HUMAN	0.09099579	0.11612091
sp|O75131|CPNE3_HUMAN	0.09101105	0.01551361
sp|P35580|MYH10_HUMAN	0.09104157	0.29888293
sp|P35555|FBN1_HUMAN	0.09129906	0.57848686
sp|Q13126-2|MTAP_HUMAN	0.09188843	0
sp|Q92817|EVPL_HUMAN	0.09188843	0.00842351
sp|Q7Z7G0|TARSH_HUMAN	0.09222031	0
sp|P55884-2|EIF3B_HUMAN	0.0928154	0.37060758
sp|P68104|EF1A1_HUMAN	0.09283829	0.00764478
sp|Q00610-2|CLH1_HUMAN	0.09337616	0.15806404
sp|Q15582|BGH3_HUMAN	0.09342194	0.27351177
sp|Q13425|SNTB2_HUMAN	0.09349251	0
sp|P08237-3|PFKAM_HUMAN	0.09384155	0
sp|Q13547|HDAC1_HUMAN	0.09444428	0
sp|P11142|HSP7C_HUMAN	0.0947094	0
sp|Q8TDZ2-4|MICA1_HUMAN	0.09477425	0
sp|E9PAV3|NACAM_HUMAN	0.09501457	0
sp|Q92506|DHB8_HUMAN	0.09529114	0.19149946
sp|P62424|RL7A_HUMAN	0.09550285	0.24295025
sp|Q96AE4-2|FUBP1_HUMAN	0.09566498	0.4546338
sp|Q6NVY1|HIBCH_HUMAN	0.0959301	0
sp|P62937|PPIA_HUMAN	0.09604836	0
sp|P13796|PLSL_HUMAN	0.09612656	0.28716654
sp|P49961|ENTP1_HUMAN	0.09698486	0.04454162
sp|P62136|PP1A_HUMAN	0.0970192	0
sp|O95782-2|AP2A1_HUMAN	0.09708405	0.19149946
sp|Q9H4A4|AMPB_HUMAN	0.09711075	0.1556212
sp|P55036-2|PSMD4_HUMAN	0.09733391	0
sp|P14317|HCLS1_HUMAN	0.09779358	0
sp|P51571|SSRD_HUMAN	0.09819794	0.09894868
sp|P49591|SYSC_HUMAN	0.09839249	0
sp|Q9BWM7|SFXN3_HUMAN	0.09849739	0

sp|Q96C23|GALM_HUMAN	0.09855557	0
sp|P00738-2|HPT_HUMAN	0.09858131	0.5452582
sp|P61923-5|COPZ1_HUMAN	0.098629	0
sp|O60716-14|CTND1_HUMAN	0.09872818	0
sp|O15031|PLXB2_HUMAN	0.09885788	0.2838047
sp|Q9Y2S2|CRYL1_HUMAN	0.09898186	0.21439649
sp|O75396|SC22B_HUMAN	0.09906006	0
sp|P46940|IQGA1_HUMAN	0.09915161	0.09832569
sp|P62888|RL30_HUMAN	0.09946251	0
sp|Q13464|ROCK1_HUMAN	0.09952164	0
sp|P25788-2|PSA3_HUMAN	0.09973145	0
sp|P31153|METK2_HUMAN	0.10010719	0
sp|Q9Y285|SYFA_HUMAN	0.1001358	0
sp|P63104|1433Z_HUMAN	0.10021019	0.01542275
sp|P40925-2|MDHC_HUMAN	0.10038185	0.01178462
sp|P16152|CBR1_HUMAN	0.10062408	0
sp|Q99426|TBCB_HUMAN	0.10071564	0
sp|P12814|ACTN1_HUMAN	0.10161591	0
sp|P30153|2AAA_HUMAN	0.10173416	0
sp|Q00796|DHSO_HUMAN	0.1017437	0
sp|O76003|GLRX3_HUMAN	0.10189056	0
sp|Q15029-2|U5S1_HUMAN	0.10219765	0.12893641
sp|P58546|MTPN_HUMAN	0.10245514	0
sp|Q8WUM4|PDC6I_HUMAN	0.10313034	0.12320797
sp|Q9ULA0|DNPEP_HUMAN	0.10323715	0.02174174
sp|P08571|CD14_HUMAN	0.10335922	0.20467198
sp|Q09028-3|RBBP4_HUMAN	0.10438538	0
sp|Q99436|PSB7_HUMAN	0.1043911	0.09894868
sp|P25787|PSA2_HUMAN	0.10449791	0.09339783
sp|Q15691|MARE1_HUMAN	0.10486984	0
sp|Q14204|DYHC1_HUMAN	0.10540772	0.02421166
sp|Q12907|LMAN2_HUMAN	0.10570908	0
sp|P48444|COPD_HUMAN	0.10570908	0.21287349
sp|Q7L2H7|EIF3M_HUMAN	0.10590172	0.5204253
sp|P61970|NTF2_HUMAN	0.10608101	0
sp|Q86UP2-4|KTN1_HUMAN	0.10622406	0
sp|O95479|G6PE_HUMAN	0.10625649	0.20467198
sp|P36955|PEDF_HUMAN	0.10642242	0
sp|P08397-2|HEM3_HUMAN	0.1065464	0
sp|Q92930|RAB8B_HUMAN	0.10659409	0
sp|P62495-2|ERF1_HUMAN	0.10716629	0.19149946
sp|Q9NRV9|HEBP1_HUMAN	0.1072731	0
sp|Q99798|ACON_HUMAN	0.10728264	0.07570045
sp|P55809|SCOT1_HUMAN	0.10798645	0
sp|Q9NYL9|TMOD3_HUMAN	0.10807419	0.33495146

sp|Q15046-2|SYK_HUMAN	0.10836792	0
sp|Q9Y5S9-2|RBM8A_HUMAN	0.10866928	0.7061832
sp|P02747|C1QC_HUMAN	0.10870552	0
sp|Q08257|QOR_HUMAN	0.10913467	0.1791607
sp|P02671-2|FIBA_HUMAN	0.10933304	0.01437165
sp|Q12905|ILF2_HUMAN	0.10935211	0.15332921
sp|P42224-2|STAT1_HUMAN	0.10956001	0.02174174
sp|P07360|CO8G_HUMAN	0.10957909	0
sp|Q9UKK9|NUDT5_HUMAN	0.10979843	0.09894868
sp|P78527|PRKDC_HUMAN	0.10988617	0
sp|P51572-2|BAP31_HUMAN	0.11037445	0.23541966
sp|Q15124|PGM5_HUMAN	0.11067009	0.01189081
sp|Q8N1G4|LRC47_HUMAN	0.11081505	0.03367973
sp|P25789|PSA4_HUMAN	0.11083031	0.06986027
sp|P13639|EF2_HUMAN	0.11121368	0.16813631
sp|P02545|LMNA_HUMAN	0.1113205	0.01551361
sp|Q92499|DDX1_HUMAN	0.1114502	0.12756637
sp|P16070-16|CD44_HUMAN	0.11148453	0
sp|P35914|HMGCL_HUMAN	0.11152077	0.09894868
sp|P40429|RL13A_HUMAN	0.11291885	0
sp|O14672|ADA10_HUMAN	0.11303139	0
sp|P17174|AATC_HUMAN	0.11317825	0
sp|P35611-2|ADDA_HUMAN	0.11340332	0
sp|O75947-2|ATP5H_HUMAN	0.11355782	0.20467198
sp|P61018|RAB4B_HUMAN	0.11357307	0
sp|P01782|HV309_HUMAN	0.11370087	0
sp|Q13347|EIF3I_HUMAN	0.11396408	0
sp|P00558|PGK1_HUMAN	0.11434364	0.00473908
sp|P07305|H10_HUMAN	0.11436844	0.35795313
sp|Q9NR45|SIAS_HUMAN	0.11443901	0.20467198
sp|P07384|CAN1_HUMAN	0.11462784	0
sp|P20292|AL5AP_HUMAN	0.11472511	0
sp|Q8IV08|PLD3_HUMAN	0.11523438	0.48520416
sp|Q9Y5X3|SNX5_HUMAN	0.11540222	0.17171621
sp|P53396-2|ACLY_HUMAN	0.11558914	0.1242725
sp|P61247|RS3A_HUMAN	0.11568832	0
sp|P08236-2|BGLR_HUMAN	0.11611939	0.1342476
sp|P02649|APOE_HUMAN	0.11621285	0.34197828
sp|A0A0C4DH31|HV118_HUMAN	0.1164856	0
sp|P42704|LPPRC_HUMAN	0.11654282	0
sp|Q6DD88|ATLA3_HUMAN	0.11655045	0.03367973
sp|Q92900-2|RENT1_HUMAN	0.11665726	0.1791607
sp|Q5JRX3-2|PREP_HUMAN	0.11710739	0
sp|P60981-2|DEST_HUMAN	0.1175499	0
sp|P31689|DNJA1_HUMAN	0.11793518	0.35795313

sp|P00390-2|GSHR_HUMAN	0.11816979	0
sp|Q9NYU2-2|UGGG1_HUMAN	0.11903381	0.7121611
sp|O14818|PSA7_HUMAN	0.11923027	0
sp|Q92890-1|UFD1_HUMAN	0.1194973	0
sp|P62249|RS16_HUMAN	0.11953354	0.19149946
sp|Q8NF37|PCAT1_HUMAN	0.11955357	0.03367973
sp|Q9H3H3-1|CK068_HUMAN	0.11981201	0
sp|P04066|FUCO_HUMAN	0.11994553	0.45033538
sp|Q9Y262|EIF3L_HUMAN	0.11997604	0
sp|P52907|CAZA1_HUMAN	0.12018585	0.43103603
sp|Q15404|RSU1_HUMAN	0.12040901	0.08307569
sp|P08238|HS90B_HUMAN	0.12069321	0.41334116
sp|O60547-2|GMDS_HUMAN	0.12099648	0
sp|P30519|HMOX2_HUMAN	0.12103272	0.25789237
sp|P50552|VASP_HUMAN	0.12141037	0.04454162
sp|O60832-2|DKC1_HUMAN	0.12163544	0
sp|Q06124-2|PTN11_HUMAN	0.12164879	0
sp|Q8WXF1|PSPC1_HUMAN	0.12169075	0
sp|P13861|KAP2_HUMAN	0.12173462	0.22032139
sp|P31937|3HIDH_HUMAN	0.12177086	0
sp|P30533|AMRP_HUMAN	0.12236404	0
sp|Q16647|PTGIS_HUMAN	0.12253952	0
sp|P12235|ADT1_HUMAN	0.12263298	0
sp|P62854|RS26_HUMAN	0.12265205	0
sp|Q13098-5|CSN1_HUMAN	0.12302017	0.04454162
sp|Q92841-1|DDX17_HUMAN	0.12321281	0
sp|Q9P265|DIP2B_HUMAN	0.12343025	0
sp|P30084|ECHM_HUMAN	0.1235199	0
sp|P01780|HV307_HUMAN	0.12398529	0
sp|Q6WCQ1-2|MPRIP_HUMAN	0.12406921	0.38617682
sp|Q92882|OSTF1_HUMAN	0.12434197	0.06291623
sp|P27338|AOFB_HUMAN	0.12434578	0.01178462
sp|P50991|TCPD_HUMAN	0.12492561	0.17006111
sp|Q9H4G4|GAPR1_HUMAN	0.12501144	0
sp|O94832|MYO1D_HUMAN	0.12517166	0
sp|Q5VW32|BROX_HUMAN	0.1257801	0
sp|P48147|PPCE_HUMAN	0.1261406	0.05483269
sp|P02748|CO9_HUMAN	0.12615585	0.14750385
sp|Q8IZ83-3|A16A1_HUMAN	0.1261921	0.07727676
sp|Q13310-2|PABP4_HUMAN	0.12673378	0
sp|A0A0C4DH25|KVD20_HUMAN	0.1267891	0
sp|Q8NHP8|PLBL2_HUMAN	0.12719727	0
sp|Q9Y5P6-2|GMPPB_HUMAN	0.12752724	0
sp|Q7Z6Z7-2|HUWE1_HUMAN	0.12787819	0
sp|P62701|RS4X_HUMAN	0.12789154	0.8983557

sp|P13667|PDIA4_HUMAN	0.12792778	0.17653626
sp|O95394-3|AGM1_HUMAN	0.12798691	0
sp|P09651-3|ROA1_HUMAN	0.12817764	0.04454162
sp|P63027|VAMP2_HUMAN	0.12822914	0
sp|Q9BUF5|TBB6_HUMAN	0.12824821	0
sp|Q14974|IMB1_HUMAN	0.12840271	0
sp|P60660-2|MYL6_HUMAN	0.12846756	0.06842031
sp|P27348|1433T_HUMAN	0.12852097	0
sp|Q9P0L0-2|VAPA_HUMAN	0.12866783	0.44572112
sp|P13693|TCTP_HUMAN	0.12907791	0
sp|P14174|MIF_HUMAN	0.12907791	0
sp|Q9Y230|RUVB2_HUMAN	0.1298523	0
sp|Q13148|TADBP_HUMAN	0.12998009	0.09894868
sp|Q9UBV8|PEF1_HUMAN	0.13002396	0
sp|P50570-5|DYN2_HUMAN	0.1304512	0.18414244
sp|P35268|RL22_HUMAN	0.13089752	0.06291623
sp|Q96QK1|VPS35_HUMAN	0.13160324	0.41804093
sp|Q96KP4|CNDP2_HUMAN	0.13173676	0.03138232
sp|P00325|ADH1B_HUMAN	0.13199234	0.5886828
sp|P30086|PEBP1_HUMAN	0.132164	0.20467198
sp|P46459|NSF_HUMAN	0.13249588	0
sp|P33176|KINH_HUMAN	0.13259697	0.21133235
sp|P49720|PSB3_HUMAN	0.13298607	0.4075265
sp|P49368|TCPG_HUMAN	0.13309288	0.28377232
sp|Q9Y696|CLIC4_HUMAN	0.13314629	0
sp|P25786-2|PSA1_HUMAN	0.13315392	0.06210651
sp|Q93009-3|UBP7_HUMAN	0.13316345	0
sp|Q9H0R4|HDHD2_HUMAN	0.13393593	0
sp|P60891|PRPS1_HUMAN	0.1340847	0
sp|P26368-2|U2AF2_HUMAN	0.13432884	0.7061832
sp|P18085|ARF4_HUMAN	0.13444519	0.7827403
sp|P53621-2|COPA_HUMAN	0.13453293	0
sp|O15382|BCAT2_HUMAN	0.13466263	0
sp|P46939-2|UTRO_HUMAN	0.13513947	1.019293
sp|P31948|STIP1_HUMAN	0.13515472	0
sp|Q13363|CTBP1_HUMAN	0.13537025	0
sp|O15511|ARPC5_HUMAN	0.13604355	0.45563722
sp|P62081|RS7_HUMAN	0.13617134	0.28516325
sp|O00232|PSD12_HUMAN	0.13714314	0.316588
sp|P08133|ANXA6_HUMAN	0.13827324	0.15771674
sp|P23142|FBLN1_HUMAN	0.1385231	0
sp|P00491|PNPH_HUMAN	0.13855171	0.14672586
sp|Q71U36-2|TBA1A_HUMAN	0.13899231	0
sp|P49915-2|GUAA_HUMAN	0.13899803	0
sp|Q9Y678|COPG1_HUMAN	0.13906288	0.09960519

sp|Q9BT78|CSN4_HUMAN	0.13944054	0.24097534
sp|Q9UHB6-4|LIMA1_HUMAN	0.1395626	0.20467198
sp|Q13409-2|DC1I2_HUMAN	0.13964748	0.06986027
sp|Q6P2Q9|PRP8_HUMAN	0.14004898	0
sp|P49419-2|AL7A1_HUMAN	0.14018631	0.02410217
sp|P00751|CFAB_HUMAN	0.14069366	0.02866885
sp|Q9Y6W5|WASF2_HUMAN	0.14110756	0.3005443
sp|Q96M27-3|PRRC1_HUMAN	0.14166832	0.19149946
sp|Q9BVK6|TMED9_HUMAN	0.14188766	0
sp|P10606|COX5B_HUMAN	0.1418953	0.06291623
sp|Q12906-2|ILF3_HUMAN	0.14201736	0
sp|Q6KB66-2|K2C80_HUMAN	0.14237595	0
sp|Q16775-2|GLO2_HUMAN	0.14277458	0
sp|Q9NUJ1|ABHDA_HUMAN	0.14279747	0.45033538
sp|P63279|UBC9_HUMAN	0.14311218	0
sp|P62314|SMD1_HUMAN	0.14340878	0.45033538
sp|P50579-2|MAP2_HUMAN	0.14354515	0.7061832
sp|Q9BTV4|TMM43_HUMAN	0.14391708	0.44572112
sp|Q9UKG1|DP13A_HUMAN	0.14430428	0
sp|P60033|CD81_HUMAN	0.14437103	0.45033538
sp|P07737|PROF1_HUMAN	0.14471245	0.07367946
sp|Q7Z3D6-3|GLUCM_HUMAN	0.1447277	0
sp|P50454|SERPH_HUMAN	0.14475441	0.0846519
sp|P40306|PSB10_HUMAN	0.14476776	0.28516325
sp|P00746|CFAD_HUMAN	0.1451397	0
sp|Q99497|PARK7_HUMAN	0.14624023	0.17511293
sp|Q1KMD3|HNRL2_HUMAN	0.14635468	0.06644611
sp|P27797|CALR_HUMAN	0.14638138	0.2699497
sp|Q13561-2|DCTN2_HUMAN	0.14639664	0
sp|Q16539-2|MK14_HUMAN	0.14666176	0
sp|P35998|PRS7_HUMAN	0.14679718	0.20174193
sp|P08621-2|RU17_HUMAN	0.14701653	0
sp|P11217-2|PYGM_HUMAN	0.14731789	0
sp|P53618|COPB_HUMAN	0.14769554	0.2718748
sp|P02746|C1QB_HUMAN	0.14808655	0
sp|P53992|SC24C_HUMAN	0.14824295	0
sp|Q8WVM8|SCFD1_HUMAN	0.1484108	0
sp|Q9Y3B3|TMED7_HUMAN	0.14879227	0
sp|P61088|UBE2N_HUMAN	0.14937592	0.656254
sp|P49411|EFTU_HUMAN	0.14958954	0.07841589
sp|O00116|ADAS_HUMAN	0.14964104	0
sp|P62829|RL23_HUMAN	0.14967155	0
sp|Q15233|NONO_HUMAN	0.15001488	0.04817784
sp|P27694|RFA1_HUMAN	0.15039253	0
sp|Q9UEY8|ADDG_HUMAN	0.15048599	0.05483269

sp|Q3LXA3|TKFC_HUMAN	0.15092278	0
sp|P28062-2|PSB8_HUMAN	0.1511612	0.5111962
sp|P37802|TAGL2_HUMAN	0.15117645	0.03208591
sp|Q9NZ01|TECR_HUMAN	0.15149212	0
sp|P10643|CO7_HUMAN	0.15197754	0
sp|Q8TE77-3|SSH3_HUMAN	0.1529541	0
sp|P27816-2|MAP4_HUMAN	0.15299988	0
sp|Q9Y4D7-2|PLXD1_HUMAN	0.1534853	0
sp|O14950|ML12B_HUMAN	0.15365219	0
sp|Q96CN7|ISOC1_HUMAN	0.15366364	0.24097534
sp|P29144|TPP2_HUMAN	0.15380669	0
sp|P62269|RS18_HUMAN	0.15443039	1.0485198
sp|P62333|PRS10_HUMAN	0.15485764	0
sp|O00487|PSDE_HUMAN	0.15506554	0
sp|P00568|KAD1_HUMAN	0.15527153	0.04817784
sp|P08865|RSSA_HUMAN	0.1556549	0.04817784
sp|P48668|K2C6C_HUMAN	0.15607262	0.33495146
sp|P60842|IF4A1_HUMAN	0.15628433	0.01178462
sp|Q9HC35-2|EMAL4_HUMAN	0.15666962	0.356152
sp|Q9NUQ9|FA49B_HUMAN	0.15686035	0.30372584
sp|P40763-2|STAT3_HUMAN	0.15698624	0.40787253
sp|Q96FW1|OTUB1_HUMAN	0.15729523	0
sp|Q16543|CDC37_HUMAN	0.15735817	0
sp|Q8WXF7-2|ATLA1_HUMAN	0.15738106	0
sp|P01624|KV315_HUMAN	0.15765762	0.45033538
sp|Q13838-2|DX39B_HUMAN	0.1577549	0.06986027
sp|P61457|PHS_HUMAN	0.15788269	0.19149946
sp|P0DOX7|IGK_HUMAN	0.15815163	0.19149946
sp|O00571-2|DDX3X_HUMAN	0.15830421	0
sp|P28838-2|AMPL_HUMAN	0.1587944	0.18313305
sp|P21281|VATB2_HUMAN	0.15921974	0.8706143
sp|P13746|1A11_HUMAN	0.15927696	0.7827403
sp|Q9UMS4|PRP19_HUMAN	0.15937042	0
sp|Q9Y315|DEOC_HUMAN	0.15950394	0
sp|P34896-2|GLYC_HUMAN	0.15960503	0.5111962
sp|Q13045-3|FLII_HUMAN	0.15986633	0.29066643
sp|P06756-3|ITAV_HUMAN	0.1599865	0.23973103
sp|O95373|IPO7_HUMAN	0.16041279	0.45033538
sp|Q13188|STK3_HUMAN	0.16043758	0
sp|Q99873-2|ANM1_HUMAN	0.1604557	0
sp|Q99758|ABCA3_HUMAN	0.1604805	0.19410844
sp|P48637|GSHB_HUMAN	0.16068077	0.24613085
sp|O75368|SH3L1_HUMAN	0.16140938	0
sp|P54802|ANAG_HUMAN	0.16149902	0.43633315
sp|P63241|IF5A1_HUMAN	0.16177368	0.8983557

sp|P48163|MAOX_HUMAN	0.16192245	0.6509351
sp|P62191-2|PRS4_HUMAN	0.16195107	0.1342476
sp|Q9BVC6|TM109_HUMAN	0.1621933	0.5204253
sp|P27487|DPP4_HUMAN	0.16226959	0.1558116
sp|P50440-3|GATM_HUMAN	0.162817	0.45033538
sp|Q14498-2|RBM39_HUMAN	0.16312218	0.40256184
sp|P31150|GDIA_HUMAN	0.16324615	0
sp|Q7Z5L7-2|PODN_HUMAN	0.1633358	0
sp|P06310|KV230_HUMAN	0.16343117	0
sp|P61601|NCALD_HUMAN	0.16374111	0
sp|P29350-4|PTN6_HUMAN	0.16388893	0.21719235
sp|Q96S97|MYADM_HUMAN	0.1639061	0
sp|Q8NBJ7|SUMF2_HUMAN	0.16399956	0.1342476
sp|P62851|RS25_HUMAN	0.16444969	1.1932944
sp|Q16822|PCKGM_HUMAN	0.1646576	0.04454162
sp|Q16851-2|UGPA_HUMAN	0.16479492	0.11768232
sp|P55060-3|XPO2_HUMAN	0.16480255	0
sp|Q9BRA2|TXD17_HUMAN	0.16549301	0
sp|P09104-2|ENOG_HUMAN	0.16604996	0
sp|Q9BZZ5-3|API5_HUMAN	0.16605186	0.04454162
sp|Q9C0C2-2|TB182_HUMAN	0.16621017	0
sp|P29401-2|TKT_HUMAN	0.16700554	0.05702926
sp|Q14764|MVP_HUMAN	0.16703796	0.11168098
sp|Q13642-1|FHL1_HUMAN	0.16710854	0
sp|P23946|CMA1_HUMAN	0.16713715	0.40256184
sp|P22061-2|PIMT_HUMAN	0.16728592	0.19410844
sp|Q9UHB9-4|SRP68_HUMAN	0.16768265	0
sp|Q99623|PHB2_HUMAN	0.16775513	0.694615
sp|Q9HCB6|SPON1_HUMAN	0.16854286	0.00900315
sp|Q96HD1-2|CREL1_HUMAN	0.16856575	0.7827403
sp|A0AVT1|UBA6_HUMAN	0.16866302	0
sp|P61353|RL27_HUMAN	0.16867828	0.5204253
sp|P07203|GPX1_HUMAN	0.16873169	0.3005443
sp|P25325-2|THTM_HUMAN	0.16882706	0.25789237
sp|Q9UNF0-2|PACN2_HUMAN	0.16903687	0
sp|Q6XQN6|PNCB_HUMAN	0.16942406	0.15517515
sp|P11177-3|ODPB_HUMAN	0.16973877	0
sp|Q969V3-2|NCLN_HUMAN	0.17005157	0
sp|Q5K4L6-2|S27A3_HUMAN	0.17039204	0
sp|Q5TFE4|NT5D1_HUMAN	0.17056465	0
sp|P30101|PDIA3_HUMAN	0.1706562	0.6213773
sp|Q96CX2|KCD12_HUMAN	0.17203903	0
sp|P20338|RAB4A_HUMAN	0.172081	0
sp|Q9UBS4|DJB11_HUMAN	0.17224312	0
sp|Q92947|GCDH_HUMAN	0.17232513	0

sp|Q63ZY3-3|KANK2_HUMAN	0.17242813	0.5538428
sp|P06753-2|TPM3_HUMAN	0.17256165	0.00934436
sp|O14617-4|AP3D1_HUMAN	0.17288017	0
sp|Q9UHX1-2|PUF60_HUMAN	0.17323112	0
sp|Q2TAY7|SMU1_HUMAN	0.17338371	0
sp|P50452|SPB8_HUMAN	0.17377472	0
sp|P55327-3|TPD52_HUMAN	0.17412567	0.2178309
sp|Q06210-2|GFPT1_HUMAN	0.17416191	0.05681695
sp|O43148-2|MCES_HUMAN	0.17417526	0.45033538
sp|Q15019-2|SEPT2_HUMAN	0.17417908	0.3812125
sp|Q5TDH0-3|DDI2_HUMAN	0.17428398	0.06291623
sp|Q9NTK5|OLA1_HUMAN	0.174963	0.6298893
sp|Q96RF0-2|SNX18_HUMAN	0.17496872	0.45033538
sp|P11586|C1TC_HUMAN	0.1750145	0.11612091
sp|P07357|CO8A_HUMAN	0.17515945	0.34137914
sp|P15428-5|PGDH_HUMAN	0.1757412	0.09894868
sp|P21333-2|FLNA_HUMAN	0.17578125	0.4486304
sp|P78371|TCPB_HUMAN	0.17579079	0.01888734
sp|O94811|TPPP_HUMAN	0.1759119	0
sp|P55084|ECHB_HUMAN	0.17599297	0.7118947
sp|Q86VS8|HOOK3_HUMAN	0.17634583	0
sp|P52566|GDIR2_HUMAN	0.17639732	0
sp|P12956|XRCC6_HUMAN	0.17679405	0.23970154
sp|P55209-2|NP1L1_HUMAN	0.1770668	0
sp|Q6DKJ4|NXN_HUMAN	0.1773529	0
sp|O14964-2|HGS_HUMAN	0.17785263	0
sp|P62266|RS23_HUMAN	0.1783371	0.35795313
sp|Q04206-3|TF65_HUMAN	0.1783514	0
sp|O43681|ASNA_HUMAN	0.17842484	0
sp|P30085|KCY_HUMAN	0.17847061	0
sp|P18124|RL7_HUMAN	0.17853546	0.5032694
sp|Q9UGI8-2|TES_HUMAN	0.17863846	0.04454162
sp|P16615|AT2A2_HUMAN	0.17863846	0.5717351
sp|Q03154-4|ACY1_HUMAN	0.17923641	0.3005443
sp|P24534|EF1B_HUMAN	0.17992783	0.35795313
sp|Q9NY33|DPP3_HUMAN	0.18068123	0.4087177
sp|P62736|ACTA_HUMAN	0.18072224	0.01888734
sp|O00410|IPO5_HUMAN	0.18083191	0.7217418
sp|P23142-4|FBLN1_HUMAN	0.18107224	0
sp|P02511|CRYAB_HUMAN	0.18114662	0
sp|P14678|RSMB_HUMAN	0.1819706	0.45033538
sp|Q27J81-2|INF2_HUMAN	0.18200874	0.28516325
sp|Q15121|PEA15_HUMAN	0.18217087	0.2178309
sp|P06681|CO2_HUMAN	0.18219376	0.29934928
sp|Q13496|MTM1_HUMAN	0.18238449	0

sp|Q16891-2|MIC60_HUMAN	0.18252373	0.02174174
sp|P26373|RL13_HUMAN	0.1830864	0.4075265
sp|Q99439|CNN2_HUMAN	0.18348503	0
sp|P53004|BIEA_HUMAN	0.1835556	0
sp|Q15631|TSN_HUMAN	0.18361282	0
sp|P30041|PRDX6_HUMAN	0.1836834	0.22784016
sp|P0DP03|HV335_HUMAN	0.1842537	0.45033538
sp|O94826|TOM70_HUMAN	0.18439102	0
sp|Q16401-2|PSMD5_HUMAN	0.18458176	0.30819008
sp|P54886-2|P5CS_HUMAN	0.18480492	0
sp|Q8NBJ5|GT251_HUMAN	0.18501472	0.61034113
sp|P11940-2|PABP1_HUMAN	0.18563461	0.2022302
sp|Q9H008|LHPP_HUMAN	0.18650436	0
sp|Q92696|PGTA_HUMAN	0.18691254	0
sp|P19404|NDUV2_HUMAN	0.18764687	0
sp|O14558|HSPB6_HUMAN	0.18797207	0.1342476
sp|Q96IJ6-2|GMPPA_HUMAN	0.18797684	0.48520416
sp|Q7L1Q6-2|BZW1_HUMAN	0.1885891	0.1342476
sp|P04632|CPNS1_HUMAN	0.18891525	0.52598757
sp|P31939|PUR9_HUMAN	0.18894196	0.33213052
sp|P21399|ACOC_HUMAN	0.18906212	0.26737198
sp|Q9UH65|SWP70_HUMAN	0.1895771	0
sp|P01911|2B1F_HUMAN	0.18989563	0
sp|P39656-3|OST48_HUMAN	0.19059181	1.1344727
sp|P50914|RL14_HUMAN	0.19086075	0.2178309
sp|O94788-4|AL1A2_HUMAN	0.19105911	0
sp|P39687|AN32A_HUMAN	0.19112015	0
sp|P10523|ARRS_HUMAN	0.19165993	0.656254
sp|P49959-3|MRE11_HUMAN	0.19208145	0
sp|Q9H0D6|XRN2_HUMAN	0.19330978	0.45563722
sp|P02753|RET4_HUMAN	0.19359207	0
sp|O14579|COPE_HUMAN	0.19364357	0.11612091
sp|P27635|RL10_HUMAN	0.19372177	0.61034113
sp|P22314-2|UBA1_HUMAN	0.19376945	0.12252306
sp|P61956-2|SUMO2_HUMAN	0.19382095	0
sp|Q15661|TRYB1_HUMAN	0.19398117	0.13604218
sp|P50225|ST1A1_HUMAN	0.19411278	0.35795313
sp|P51858|HDGF_HUMAN	0.19427872	0
sp|O75306-2|NDUS2_HUMAN	0.19436073	0.09894868
sp|Q96DG6|CMBL_HUMAN	0.19447136	0
sp|P68366-2|TBA4A_HUMAN	0.19459534	0
sp|P31942-2|HNRH3_HUMAN	0.19482231	0.7498006
sp|P60983|GMFB_HUMAN	0.19499207	0
sp|P07741|APT_HUMAN	0.19506454	0.04454162
sp|Q99598|TSNAX_HUMAN	0.19517517	0.09894868

sp|P49755|TMEDA_HUMAN	0.1953106	0.29066643
sp|Q02952-2|AKA12_HUMAN	0.19578362	0.7827403
sp|P13010|XRCC5_HUMAN	0.19595718	0.24452867
sp|Q9Y3Z3|SAMH1_HUMAN	0.19641304	0
sp|P52306|GDS1_HUMAN	0.1968689	0
sp|O75116|ROCK2_HUMAN	0.19695282	0
sp|Q9UBI6|GBG12_HUMAN	0.19713974	0.19149946
sp|P25311|ZA2G_HUMAN	0.1972313	0.09339783
sp|P14618-2|KPYM_HUMAN	0.19723892	0.29066643
sp|Q9UBF2|COPG2_HUMAN	0.1972866	0.2178309
sp|Q9H3K6-2|BOLA2_HUMAN	0.19737244	0
sp|Q14108-2|SCRB2_HUMAN	0.19755745	0.19149946
sp|Q96HY6|DDRGK_HUMAN	0.19806099	0.30372584
sp|Q7Z7H5-3|TMED4_HUMAN	0.19816971	0
sp|P14923|PLAK_HUMAN	0.19826317	0
sp|Q14980-2|NUMA1_HUMAN	0.1988926	0.63199323
sp|Q16181-2|SEPT7_HUMAN	0.19943237	0.1017544
sp|P62857|RS28_HUMAN	0.19986725	0
sp|O96009|NAPSA_HUMAN	0.2002449	1.0634323
sp|Q9Y4L1|HYOU1_HUMAN	0.20067978	0.4378217
sp|Q96G03|PGM2_HUMAN	0.20082474	0.51870745
sp|P30566|PUR8_HUMAN	0.20121574	0.35795313
sp|P09874|PARP1_HUMAN	0.20191574	0.47871065
sp|O00478-2|BT3A3_HUMAN	0.20253754	0.19149946
sp|Q9UBQ7|GRHPR_HUMAN	0.2026825	0.09894868
sp|P00966|ASSY_HUMAN	0.20328331	0
sp|Q13435|SF3B2_HUMAN	0.20432281	0
sp|Q9UNZ2-5|NSF1C_HUMAN	0.20435905	0.27902296
sp|Q15436|SC23A_HUMAN	0.20482635	0.09339783
sp|P40939|ECHA_HUMAN	0.20495796	0.6942292
sp|P52597|HNRPF_HUMAN	0.20555878	0.04454162
sp|Q96BM9|ARL8A_HUMAN	0.20648193	0
sp|Q14BN4-2|SLMAP_HUMAN	0.20676231	0
sp|P49354|FNTA_HUMAN	0.20732117	1.1505735
sp|Q13177|PAK2_HUMAN	0.2077694	0
sp|P24666-2|PPAC_HUMAN	0.20807171	0.2178309
sp|Q9BRF8|CPPED_HUMAN	0.20828629	0
sp|Q13488|VPP3_HUMAN	0.20840073	0.09894868
sp|P62753|RS6_HUMAN	0.20856667	0.35193655
sp|Q9Y265|RUVB1_HUMAN	0.20879745	0
sp|P26640|SYVC_HUMAN	0.20883942	0.31759265
sp|P33316|DUT_HUMAN	0.2088852	0
sp|O00303|EIF3F_HUMAN	0.20911598	0.5692702
sp|Q9UKV3-5|ACINU_HUMAN	0.20917511	1.2095301
sp|O95372|LYPA2_HUMAN	0.20920563	0

sp|Q12805-2|FBLN3_HUMAN	0.20944214	0.4301026
sp|Q9NR19-2|ACSA_HUMAN	0.20993423	0
sp|Q9UBT2|SAE2_HUMAN	0.21095085	0.34323573
sp|P22059|OSBP1_HUMAN	0.21105385	0
sp|O60749-2|SNX2_HUMAN	0.21106529	0.03712561
sp|P12268|IMDH2_HUMAN	0.21128464	0.33495146
sp|Q96IU4|ABHEB_HUMAN	0.21172333	0.312067
sp|P13798|ACPH_HUMAN	0.21183968	0.2174103
sp|O75155|CAND2_HUMAN	0.2118454	0.656254
sp|P50502|F10A1_HUMAN	0.2118988	0.52598757
sp|Q9NZU5|LMCD1_HUMAN	0.21191216	0
sp|O60763-2|USO1_HUMAN	0.21193314	0.14338206
sp|Q13438-4|OS9_HUMAN	0.21309662	0.7827403
sp|O14791-2|APOL1_HUMAN	0.21347904	0.19149946
sp|Q16555|DPYL2_HUMAN	0.21391487	0.43348378
sp|P14207|FOLR2_HUMAN	0.21403599	0.19149946
sp|Q99832|TCPH_HUMAN	0.214365	0
sp|P23528|COF1_HUMAN	0.2143898	0.20174193
sp|Q9UBG0|MRC2_HUMAN	0.21456909	0.2591514
sp|O43390-2|HNRPR_HUMAN	0.21466827	0.984558
sp|Q00341|VIGLN_HUMAN	0.21529198	0.7498006
sp|P46109|CRKL_HUMAN	0.2153759	0.09894868
sp|O94776|MTA2_HUMAN	0.21562576	0
sp|Q92945|FUBP2_HUMAN	0.21606827	0.7259058
sp|Q5T013-4|HYI_HUMAN	0.21646118	0.2178309
sp|P11498|PYC_HUMAN	0.21711922	0.312067
sp|P04003|C4BPA_HUMAN	0.21757889	0.368128
sp|P07814|SYEP_HUMAN	0.21759987	0.54838234
sp|P07437|TBB5_HUMAN	0.21766663	0.06291623
sp|P11678|PERE_HUMAN	0.21773338	0
sp|P39059|COFA1_HUMAN	0.21779823	0.30372584
sp|Q9Y376|CAB39_HUMAN	0.21825027	0.28516325
sp|O43294|TGFI1_HUMAN	0.21877861	0.1342476
sp|Q96JB5-4|CK5P3_HUMAN	0.21882248	0.06291623
sp|P50395|GDIB_HUMAN	0.21931076	0.23508528
sp|P67936|TPM4_HUMAN	0.21975899	0.16702904
sp|Q9BW30|TPPP3_HUMAN	0.22031975	0
sp|P49756|RBM25_HUMAN	0.22051048	0.19149946
sp|Q53GG5-2|PDLI3_HUMAN	0.22098732	0.2178309
sp|O60664-4|PLIN3_HUMAN	0.22168541	0.10861364
sp|Q5TZA2|CROCC_HUMAN	0.22179794	0.20467198
sp|Q15257-2|PTPA_HUMAN	0.22195244	0
sp|Q8WYA6-2|CTBL1_HUMAN	0.22208595	0.19149946
sp|P62750|RL23A_HUMAN	0.22258568	0.19149946
sp|P05155-2|IC1_HUMAN	0.22349167	0.23541966

sp|P51570-2|GALK1_HUMAN	0.22349358	0
sp|P01031|CO5_HUMAN	0.22389603	1.0297993
sp|O15145|ARPC3_HUMAN	0.22407532	0.09894868
sp|P61020|RAB5B_HUMAN	0.2242775	0.5204253
sp|Q13618-2|CUL3_HUMAN	0.22444153	0.5832693
sp|P49588-2|SYAC_HUMAN	0.2251358	1.0224596
sp|O60884|DNJA2_HUMAN	0.22546291	0
sp|O60488-2|ACSL4_HUMAN	0.22574425	0
sp|O43143|DHX15_HUMAN	0.22576904	0.66088426
sp|Q9Y3A3-3|PHOCN_HUMAN	0.22587967	0.19149946
sp|P39019|RS19_HUMAN	0.22600174	0.5204253
sp|Q5JPE7-2|NOMO2_HUMAN	0.22629738	0.656254
sp|Q68EM7-2|RHG17_HUMAN	0.22640228	0
sp|P14550|AK1A1_HUMAN	0.22681808	0.1742838
sp|P02751-15|FINC_HUMAN	0.22683525	0
sp|P25685-2|DNJB1_HUMAN	0.22693253	0.09894868
sp|O75915|PRAF3_HUMAN	0.2272911	0.21439649
sp|P10909-5|CLUS_HUMAN	0.22731972	0.24295025
sp|O43242|PSMD3_HUMAN	0.22759628	0.312067
sp|Q96P70|IPO9_HUMAN	0.22790813	0.19149946
sp|O43396|TXNL1_HUMAN	0.22840118	0
sp|P07195|LDHB_HUMAN	0.22863388	0.5077588
sp|Q07960|RHG01_HUMAN	0.22877502	0.2207585
sp|O75475|PSIP1_HUMAN	0.22907639	0.656254
sp|Q8TCS8|PNPT1_HUMAN	0.22950554	0
sp|P12277|KCRB_HUMAN	0.22960854	0.48353976
sp|P31513|FMO3_HUMAN	0.22968864	0
sp|Q53H82|LACB2_HUMAN	0.22979927	0
sp|Q9HC38-2|GLOD4_HUMAN	0.23008156	0
sp|Q9NT62-2|ATG3_HUMAN	0.2307167	0
sp|Q9Y224|RTRAF_HUMAN	0.23096466	1.1791906
sp|O60506-3|HNRPQ_HUMAN	0.2313137	0.3852666
sp|P19013|K2C4_HUMAN	0.23154831	0.65625405
sp|Q08379|GOGA2_HUMAN	0.23167801	0.45033538
sp|P16298-2|PP2BB_HUMAN	0.23256874	0
sp|Q9NP79|VTA1_HUMAN	0.23262978	0.19149946
sp|P62140|PP1B_HUMAN	0.23300934	0.09894868
sp|Q9UBB4-2|ATX10_HUMAN	0.23322487	0
sp|P54652|HSP72_HUMAN	0.23387527	0
sp|Q9Y570-2|PPME1_HUMAN	0.23418427	0.6070219
sp|P46779-2|RL28_HUMAN	0.2343235	0.7827403
sp|P48741|HSP77_HUMAN	0.23472786	0
sp|O00429-3|DNM1L_HUMAN	0.23500443	0.42646354
sp|O76011|KRT34_HUMAN	0.23675728	0
sp|Q06787-10|FMR1_HUMAN	0.23683357	0.43633315

sp|Q09161|NCBP1_HUMAN	0.23715782	0
sp|Q02818|NUCB1_HUMAN	0.23736191	0.85449874
sp|Q8NHV1|GIMA7_HUMAN	0.23782349	0.7061832
sp|A0A0C4DH38|HV551_HUMAN	0.23828888	0.19149946
sp|P04843|RPN1_HUMAN	0.2382946	0.7225199
sp|Q92629-2|SGCD_HUMAN	0.23854065	0
sp|P43686|PRS6B_HUMAN	0.23908901	0.11949348
sp|Q15435|PP1R7_HUMAN	0.23923588	0
sp|P62995-3|TRA2B_HUMAN	0.23948288	1.1932944
sp|Q9NY15|STAB1_HUMAN	0.23983765	0.19149946
sp|Q15075|EEA1_HUMAN	0.2403984	0.48133445
sp|P62304|RUXE_HUMAN	0.24076653	0
sp|P04792|HSPB1_HUMAN	0.24077797	0.3731982
sp|P43243|MATR3_HUMAN	0.24085045	0.92667
sp|Q15642-3|CIP4_HUMAN	0.24129581	0
sp|O60610-2|DIAP1_HUMAN	0.24146557	0.7957244
sp|P34932|HSP74_HUMAN	0.24190903	0.28765488
sp|P54727|RD23B_HUMAN	0.24205017	0.40256184
sp|P53990-2|IST1_HUMAN	0.24277878	0
sp|Q96A33-2|CCD47_HUMAN	0.24336433	0.7827403
sp|Q15274|NADC_HUMAN	0.24604607	0.21439649
sp|P31321|KAP1_HUMAN	0.24619293	0
sp|P05156|CFAI_HUMAN	0.24628448	0.57986265
sp|P35813-3|PPM1A_HUMAN	0.24656105	0
sp|P14314-2|GLU2B_HUMAN	0.24713326	0
sp|Q14258|TRI25_HUMAN	0.24726486	1.1932943
sp|P30419-2|NMT1_HUMAN	0.24793243	0.19149946
sp|Q9Y2B0|CNPY2_HUMAN	0.24871731	0.45033538
sp|P36873-2|PP1G_HUMAN	0.24905014	0
sp|Q9Y6E0|STK24_HUMAN	0.24922943	0
sp|P52943|CRIP2_HUMAN	0.24931717	0.40256184
sp|P04350|TBB4A_HUMAN	0.24949455	0
sp|O60313-10|OPA1_HUMAN	0.25048637	0
sp|Q86VP6|CAND1_HUMAN	0.2513256	0.5204253
sp|Q9NR31|SAR1A_HUMAN	0.2520256	0.7827403
sp|Q8WVV9-5|HNRLL_HUMAN	0.2520275	0
sp|Q9UH99-2|SUN2_HUMAN	0.25211334	0.84879977
sp|P02790|HEMO_HUMAN	0.2526703	1.0902835
sp|P43307|SSRA_HUMAN	0.25373077	0.7827403
sp|P17980|PRS6A_HUMAN	0.25574303	0.2699497
sp|P04844|RPN2_HUMAN	0.25634193	0.84879977
sp|P10644|KAP0_HUMAN	0.2564354	0.98032326
sp|P46821|MAP1B_HUMAN	0.25689125	0.45033538
sp|Q6UW02|CP20A_HUMAN	0.2570076	0.45033538
sp|Q9UJU6-2|DBNL_HUMAN	0.25735474	0.06291623

sp|Q15717|ELAV1_HUMAN	0.25787354	0.43633315
sp|P22894|MMP8_HUMAN	0.2582302	0.45033538
sp|O60825-2|F262_HUMAN	0.26019478	0
sp|P31323|KAP3_HUMAN	0.26031303	0.7827403
sp|Q14194-2|DPYL1_HUMAN	0.26057053	0
sp|Q14847|LASP1_HUMAN	0.2610798	0
sp|Q9BY32|ITPA_HUMAN	0.26141834	0.06291623
sp|P31943|HNRH1_HUMAN	0.2616558	0.29066643
sp|P55265-5|DSRAD_HUMAN	0.26235008	0.45033538
sp|Q9BWD1|THIC_HUMAN	0.2632103	0
sp|Q709C8-2|VP13C_HUMAN	0.26381016	0.35795313
sp|Q8TDL5|BPIB1_HUMAN	0.2653618	0.24097534
sp|Q9BUT1|BDH2_HUMAN	0.26598167	0.26737198
sp|Q92820|GGH_HUMAN	0.2662773	0.19149946
sp|O00170|AIP_HUMAN	0.26633453	0.35795313
sp|P36542|ATPG_HUMAN	0.26643467	0
sp|Q04828|AK1C1_HUMAN	0.26759148	0.2178309
sp|P36578|RL4_HUMAN	0.26762962	0.62912476
sp|O60271-5|JIP4_HUMAN	0.26769638	0
sp|O14745|NHRF1_HUMAN	0.26817894	1.0801278
sp|Q92896-2|GSLG1_HUMAN	0.26848984	0.33495146
sp|Q8N8S7-2|ENAH_HUMAN	0.26861954	0
sp|Q7Z4H8|PLGT3_HUMAN	0.26868248	1.0485198
sp|P14324|FPPS_HUMAN	0.2703514	0
sp|Q99961-2|SH3G1_HUMAN	0.27054787	0
sp|P29466-2|CASP1_HUMAN	0.2710228	0.7061832
sp|Q9BS40|LXN_HUMAN	0.27104378	0
sp|O00560-2|SDCB1_HUMAN	0.27181053	0.45033538
sp|P55854-2|SUMO3_HUMAN	0.27313805	0
sp|O43252|PAPS1_HUMAN	0.2736473	0
sp|Q8IUX7|AEBP1_HUMAN	0.27447128	0.09894868
sp|P09917-3|LOX5_HUMAN	0.27487183	0.5204253
sp|Q14195-2|DPYL3_HUMAN	0.27539062	1.2859163
sp|Q969G5|CAVN3_HUMAN	0.27624702	1.0485198
sp|O15260-2|SURF4_HUMAN	0.27662086	0.19149946
sp|Q9UQ16-2|DYN3_HUMAN	0.27667046	0
sp|P22102|PUR2_HUMAN	0.2773304	0.5111962
sp|O43488|ARK72_HUMAN	0.27781677	0
sp|Q6UW68|TM205_HUMAN	0.2779274	0.45033538
sp|P00740-2|FA9_HUMAN	0.27911377	0.7498006
sp|P21291|CSRP1_HUMAN	0.27935028	0.694615
sp|Q8WZA0-2|LZIC_HUMAN	0.27945423	0.45033538
sp|Q13243-3|SRSF5_HUMAN	0.2803688	0.45033538
sp|Q8N1B4-2|VPS52_HUMAN	0.28052902	0
sp|Q14697|GANAB_HUMAN	0.28157806	0

sp|P54578-2|UBP14_HUMAN	0.28347778	0.19149946
sp|P11171-2|41_HUMAN	0.28372002	0.7061832
sp|Q8WX93-5|PALLD_HUMAN	0.28443336	0.3188726
sp|P48506|GSH1_HUMAN	0.28477287	0.7827403
sp|Q8TCJ2|STT3B_HUMAN	0.28507996	0.7827403
sp|P55263|ADK_HUMAN	0.2851658	0
sp|O00151|PDLI1_HUMAN	0.28569794	0.5176613
sp|Q9Y266|NUDC_HUMAN	0.2858143	0
sp|P35573|GDE_HUMAN	0.28666115	0.7061832
sp|P57737-4|CORO7_HUMAN	0.28685188	0.45033538
sp|P35270|SPRE_HUMAN	0.28704834	0
sp|P62195|PRS8_HUMAN	0.287117	0.21439649
sp|P23083|HV102_HUMAN	0.28789902	0.656254
sp|P00441|SODC_HUMAN	0.2880268	0.65625405
sp|Q13043|STK4_HUMAN	0.28811264	0.7827403
sp|Q9P0J0-2|NDUAD_HUMAN	0.29042816	0.19149946
sp|P18084|ITB5_HUMAN	0.29086494	0.7061832
sp|Q13505-3|MTX1_HUMAN	0.29124165	0
sp|Q9BU23-3|LMF2_HUMAN	0.29179573	0.45033538
sp|Q16204|CCDC6_HUMAN	0.29298973	0
sp|Q01518|CAP1_HUMAN	0.29440403	0
sp|Q13200|PSMD2_HUMAN	0.29512978	0.09894868
sp|P0C0L5|CO4B_HUMAN	0.29516602	0.656254
sp|Q9NR12-2|PDLI7_HUMAN	0.29543304	0.633381
sp|P36269-2|GGT5_HUMAN	0.29641342	1.120602
sp|P30046|DOPD_HUMAN	0.2969036	0.45033538
sp|Q9NQG5|RPR1B_HUMAN	0.29812813	0.2178309
sp|P54725-2|RD23A_HUMAN	0.2998581	0.19149946
sp|O15254-2|ACOX3_HUMAN	0.3006897	0
sp|P07358|CO8B_HUMAN	0.30174446	1.2095301
sp|O14974-3|MYPT1_HUMAN	0.3021469	0.7217418
sp|P28161|GSTM2_HUMAN	0.30265808	0.7827403
sp|O43852-5|CALU_HUMAN	0.30275726	0.09894868
sp|P54819-2|KAD2_HUMAN	0.30295944	0.40256184
sp|P62277|RS13_HUMAN	0.30319595	0.19149946
sp|Q3SY69|AL1L2_HUMAN	0.30352783	0.45033538
sp|O60879-2|DIAP2_HUMAN	0.3037567	0.43633315
sp|P29692|EF1D_HUMAN	0.3046627	0
sp|P05387|RLA2_HUMAN	0.306036	0.45033538
sp|O60784|TOM1_HUMAN	0.3063259	0.30372584
sp|Q15149-9|PLEC_HUMAN	0.3064804	0.656254
sp|P49821-2|NDUV1_HUMAN	0.3065834	0.42646354
sp|Q9UBE0|SAE1_HUMAN	0.3079586	0.40256184
sp|Q9NVA2|SEP11_HUMAN	0.30962753	0.8244277
sp|P55039|DRG2_HUMAN	0.3097725	0

sp|P31946-2|1433B_HUMAN	0.31101227	0
sp|P04440|DPB1_HUMAN	0.31212616	0.45033538
sp|Q08170|SRSF4_HUMAN	0.31435204	0.656254
sp|P12270|TPR_HUMAN	0.3149414	0.19149946
sp|Q9Y5Z4-2|HEBP2_HUMAN	0.31522274	0.45033538
sp|Q04446|GLGB_HUMAN	0.3153305	0.14927356
sp|Q92688-2|AN32B_HUMAN	0.31555176	0.19149946
sp|P08708|RS17_HUMAN	0.31569672	0.7588735
sp|P50135|HNMT_HUMAN	0.3163185	0
sp|Q00013-2|EM55_HUMAN	0.31648827	0
sp|Q16630-3|CPSF6_HUMAN	0.3171463	0.7061832
sp|O94875-11|SRBS2_HUMAN	0.3171959	0.656254
sp|O94911-3|ABCA8_HUMAN	0.31796074	0.45033538
sp|P01834|IGKC_HUMAN	0.32026672	0.7827403
sp|P12111-4|CO6A3_HUMAN	0.3215065	0.656254
sp|P62241|RS8_HUMAN	0.32235336	1.0634323
sp|O00534|VMA5A_HUMAN	0.32294464	0.91072154
sp|P49841-2|GSK3B_HUMAN	0.32541656	0
sp|P12931-2|SRC_HUMAN	0.32631874	0.312067
sp|Q13976|KGP1_HUMAN	0.32652473	0.87291557
sp|P02765|FETUA_HUMAN	0.32713127	1.3006523
sp|P48426-2|PI42A_HUMAN	0.32749176	0.7827403
sp|Q9NZ08-2|ERAP1_HUMAN	0.32867432	1.2064548
sp|Q96AQ6-2|PBIP1_HUMAN	0.33159828	0.26737198
sp|P15374|UCHL3_HUMAN	0.33196068	0.19149946
sp|Q0ZGT2-4|NEXN_HUMAN	0.33286476	0
sp|P0CG38|POTEI_HUMAN	0.33434296	0
sp|P11166|GTR1_HUMAN	0.3353138	1.1932944
sp|Q8N163-2|CCAR2_HUMAN	0.33559227	0.30372584
sp|Q9NRX4|PHP14_HUMAN	0.33570576	0.35795313
sp|P41240|CSK_HUMAN	0.33655167	0.19149946
sp|P31946|1433B_HUMAN	0.33868027	0
sp|Q13885|TBB2A_HUMAN	0.34007263	0.656254
sp|Q9UJZ1-2|STML2_HUMAN	0.34022903	0.45033538
sp|Q15293|RCN1_HUMAN	0.34101677	0.2178309
sp|Q13596-2|SNX1_HUMAN	0.34108734	0.656254
sp|P39023|RL3_HUMAN	0.3431759	0.45033538
sp|O15355|PPM1G_HUMAN	0.34332848	0.35795313
sp|P24844|MYL9_HUMAN	0.34516525	1.1505735
sp|Q14152-2|EIF3A_HUMAN	0.34660912	0.87291557
sp|Q14651|PLSI_HUMAN	0.34743404	0.656254
sp|P09493-5|TPM1_HUMAN	0.34773254	0.45033538
sp|Q8TD06|AGR3_HUMAN	0.3487854	0
sp|P00736|C1R_HUMAN	0.35061646	0.45033538
sp|Q99538-2|LGMN_HUMAN	0.35564995	0.7827403

sp|Q07812-2|BAX_HUMAN	0.35780334	0.40256184
sp|P05452|TETN_HUMAN	0.36541176	0.7061832
sp|Q15172-2|2A5A_HUMAN	0.36954308	0.19149946
sp|Q96C86|DCPS_HUMAN	0.36987495	0.7217418
sp|Q9BTW9-4|TBCD_HUMAN	0.37113953	0.7061832
sp|Q7L576|CYFP1_HUMAN	0.37778187	0
sp|P36542-2|ATPG_HUMAN	0.37857914	0.656254
sp|O60831|PRAF2_HUMAN	0.38008308	1.1505735
sp|A1L4H1|SRCRL_HUMAN	0.38066483	0.45033538
sp|Q9UHD8-3|SEPT9_HUMAN	0.38508606	0.19149946
sp|Q96RU3-3|FNBP1_HUMAN	0.38759136	0
sp|P01619|KV320_HUMAN	0.3877735	0.656254
sp|P42226|STAT6_HUMAN	0.38802147	0.2178309
sp|P06396|GELS_HUMAN	0.38851357	0.656254
sp|P62633-3|CNBP_HUMAN	0.39071083	1.1932944
sp|Q9NWV4|CZIB_HUMAN	0.39134598	0.19149946
sp|Q13247-3|SRSF6_HUMAN	0.39260006	1.1932944
sp|P54577|SYYC_HUMAN	0.3930416	0.61034113
sp|P13646-3|K1C13_HUMAN	0.39367294	0.7498006
sp|P20810-10|ICAL_HUMAN	0.39757156	0.5204253
sp|A0MZ66-5|SHOT1_HUMAN	0.39935303	0.45033538
sp|Q99733-2|NP1L4_HUMAN	0.39956474	0.91601294
sp|Q04637-4|IF4G1_HUMAN	0.3996868	1.2095301
sp|O00233-2|PSMD9_HUMAN	0.40013885	0.2178309
sp|P07951-3|TPM2_HUMAN	0.40236473	0.7588735
sp|P35612-2|ADDB_HUMAN	0.4024086	0.656254
sp|Q92878-2|RAD50_HUMAN	0.40428734	0.09894868
sp|P35637-2|FUS_HUMAN	0.40535355	0.45033538
sp|P01743|HV146_HUMAN	0.40740585	0.45033538
sp|P12814-2|ACTN1_HUMAN	0.40767097	0.19149946
sp|Q562R1|ACTBL_HUMAN	0.40929794	0.35795313
sp|O75881|CP7B1_HUMAN	0.4105034	0.19149946
sp|Q7LG56-6|RIR2B_HUMAN	0.411726	0.7827403
sp|P01591|IGJ_HUMAN	0.41192818	0.19149946
sp|P51692|STA5B_HUMAN	0.41471195	0.656254
sp|P40261|NNMT_HUMAN	0.41786194	1.1932944
sp|Q10713-2|MPPA_HUMAN	0.42167854	0.45033538
sp|P30443|1A01_HUMAN	0.42248535	1.1932944
sp|P84098|RL19_HUMAN	0.4258461	0.91601294
sp|Q06278|AOXA_HUMAN	0.42956638	0.7827403
sp|Q01518-2|CAP1_HUMAN	0.43112755	0
sp|O94979-10|SC31A_HUMAN	0.43284416	0.21439649
sp|Q92556|ELMO1_HUMAN	0.4385643	0.7061832
sp|P20674|COX5A_HUMAN	0.44174385	1.1932944
sp|O75489|NDUS3_HUMAN	0.44189644	0.35795313

sp|O14787-2|TNPO2_HUMAN	0.4427414	0.656254
sp|Q13630|FCL_HUMAN	0.45207787	1.1505735
sp|Q99584|S10AD_HUMAN	0.45292473	1.1932944
sp|P00739-2|HPTR_HUMAN	0.45766068	0.84879977
sp|Q05682|CALD1_HUMAN	0.46874237	1.1276597
sp|A0A075B6P5|KV228_HUMAN	0.472147	0.656254
sp|Q92888-2|ARHG1_HUMAN	0.47855282	1.1505735
sp|Q9UNS2|CSN3_HUMAN	0.4799242	0.656254
sp|Q9Y4G6|TLN2_HUMAN	0.48121643	1.1932944
sp|Q96JJ3-3|ELMO2_HUMAN	0.48792267	0
sp|Q8IUD2-2|RB6I2_HUMAN	0.5035095	0.656254
sp|P05090|APOD_HUMAN	0.5132694	1.1505735
sp|Q6IBS0|TWF2_HUMAN	0.5144615	0.19149946
sp|Q92552-2|RT27_HUMAN	0.51666737	1.1932944
sp|P55058|PLTP_HUMAN	0.51915836	0.656254
sp|Q92599-2|SEPT8_HUMAN	0.5222473	0.6070219
sp|P49721|PSB2_HUMAN	0.52494526	0.656254
sp|P0C0L4|CO4A_HUMAN	0.5280838	0.45033538
sp|Q9P2B2|FPRP_HUMAN	0.5334892	0.7588735
sp|O95050-2|INMT_HUMAN	0.53518295	1.1932944
sp|O95816|BAG2_HUMAN	0.5363312	1.1932944
sp|Q9Y295|DRG1_HUMAN	0.556797	1.1932944
sp|P50238|CRIP1_HUMAN	0.559618	1.1932944
sp|P00748|FA12_HUMAN	0.5597954	0.45033538
sp|P05534|1A24_HUMAN	0.61688423	0
sp|P02533|K1C14_HUMAN	0.61904144	0.656254
sp|Q9NZT2-2|OGFR_HUMAN	0.62480545	1.1505735
sp|O00499-10|BIN1_HUMAN	0.6847954	1.1932944
sp|P55083-2|MFAP4_HUMAN	0.68790627	1.1932944
sp|Q14697-2|GANAB_HUMAN	0.71342564	0.656254
sp|Q9BV20|MTNA_HUMAN	0.7195835	0.7827403
sp|P35542|SAA4_HUMAN	0.73840714	1.1932944
sp|P79483|DRB3_HUMAN	0.7456951	0.656254
sp|P19012-2|K1C15_HUMAN	0.75746155	0.656254
sp|Q9P289|STK26_HUMAN	0.7981491	0.656254
sp|Q9H6R3|ACSS3_HUMAN	0.86244965	0.45033538
sp|P08842|STS_HUMAN	0.8742943	0.45033538
sp|P01871-2|IGHM_HUMAN	0.91207314	1.2941489
sp|Q31612|1B73_HUMAN	1.0859318	0.656254
sp|Q04695|K1C17_HUMAN	1.2461987	0.656254
sp|P02461|CO3A1_HUMAN	1.3876724	1.1932944
sp|O94804|STK10_HUMAN	2.159443	0.656254
sp|Q30134|2B18_HUMAN	2.4108124	0.656254
