# Supplementary material for: Peptidylarginine deiminase 2 citrullinates MZB1 and promotes the secretion of IgM and IgA
Source: Front Immunol. 2023 Nov 29;14:1290585. doi: 10.3389/fimmu.2023.1290585 (PMC10716219; doi:10.3389/fimmu.2023.1290585)
Supplement: Supplementary file 4 [file DataSheet_4.pdf]

Supplemental Table 4: RA-ILD2 vs controls

Accession #	Fold Change	p value (-log10)
sp|Q15109-10|RAGE_HUMAN	-1.7763214	1.6997428
sp|P22748|CAH4_HUMAN	-1.2560749	2.1818786
sp|Q8IWL2-2|SFTA1_HUMAN	-1.1781044	4.509013
sp|P62805|H4_HUMAN	-1.0572586	6.3204336
sp|O95810|CAVN2_HUMAN	-1.0290108	3.4656596
sp|Q9NZA1-2|CLIC5_HUMAN	-1.0119743	3.342776
sp|P00167-2|CYB5_HUMAN	-0.9791355	1.4104178
sp|P12429|ANXA3_HUMAN	-0.9170113	8.032379
sp|O96009|NAPSA_HUMAN	-0.9151058	2.4829872
sp|P08174-3|DAF_HUMAN	-0.9129753	1.6960104
sp|P09467|F16P1_HUMAN	-0.7995834	4.7229815
sp|P33151|CADH5_HUMAN	-0.7079163	1.4104178
sp|Q9UGT4|SUSD2_HUMAN	-0.7060204	3.1266599
sp|Q9NZN3|EHD3_HUMAN	-0.7051048	1.6960104
sp|P50895|BCAM_HUMAN	-0.6978416	4.964395
sp|P41218|MNDA_HUMAN	-0.6947212	2.1818786
sp|P26006-1|ITA3_HUMAN	-0.6929436	4.2046685
sp|P60903|S10AA_HUMAN	-0.6840897	1.6960104
sp|P51659|DHB4_HUMAN	-0.6814957	1.9647322
sp|P11233|RALA_HUMAN	-0.6700573	1.6960104
sp|P12821-2|ACE_HUMAN	-0.6501007	2.4829872
sp|P07339|CATD_HUMAN	-0.636694	7.2183566
sp|P49407-2|ARRB1_HUMAN	-0.6357803	2.4935064
sp|P35241|RADI_HUMAN	-0.6244202	2.838374
sp|P56199|ITA1_HUMAN	-0.6150456	5.663637
sp|Q6NZI2|CAVN1_HUMAN	-0.6097794	5.3240805
sp|P50897-2|PPT1_HUMAN	-0.6068974	1.6960104
sp|P05362|ICAM1_HUMAN	-0.595871	3.6211643
sp|Q9NPY3|C1QR1_HUMAN	-0.5870018	1.4716977
sp|P05164-3|PERM_HUMAN	-0.5861893	8.709611
sp|Q9Y624|JAM1_HUMAN	-0.5785332	2.3047035
sp|Q13510-2|ASAH1_HUMAN	-0.5773602	4.6488075
sp|P06703|S10A6_HUMAN	-0.5683365	1.4104178
sp|P09668|CATH_HUMAN	-0.5467377	2.0337436
sp|Q01469|FABP5_HUMAN	-0.5442257	3.9658737
sp|P55290-4|CAD13_HUMAN	-0.5330181	1.4104178
sp|P21397|AOFA_HUMAN	-0.5290012	3.6182032
sp|P26447|S10A4_HUMAN	-0.5194874	2.3143692
sp|Q07157|ZO1_HUMAN	-0.4937744	7.3871665
sp|P02788|TRFL_HUMAN	-0.4864807	13.995548
sp|Q6PIU2-2|NCEH1_HUMAN	-0.4733734	1.4278674
sp|P09110|THIK_HUMAN	-0.4640579	1.7703108
sp|Q92817|EVPL_HUMAN	-0.4540634	2.3801057

sp|P05091|ALDH2_HUMAN	-0.4523239	8.576053
sp|P22307-8|NLTP_HUMAN	-0.4496727	2.9807727
sp|P59666|DEF3_HUMAN	-0.4464665	1.3815327
sp|P02786|TFR1_HUMAN	-0.4317322	3.292093
sp|P63218|GBG5_HUMAN	-0.4301987	1.6604291
sp|Q08722-2|CD47_HUMAN	-0.4292622	1.6960104
sp|P61626|LYSC_HUMAN	-0.4142609	2.8384566
sp|Q8NF37|PCAT1_HUMAN	-0.4002323	1.827201
sp|P01903|DRA_HUMAN	-0.3992081	2.1892438
sp|P61106|RAB14_HUMAN	-0.3828964	3.6211643
sp|P16284-3|PECA1_HUMAN	-0.3819466	2.9846454
sp|P02769|ALBU_BOVIN	-0.3751183	3.7944438
sp|P11717|MPRI_HUMAN	-0.3667221	1.4104178
sp|Q9UPQ0-3|LIMC1_HUMAN	-0.3599548	1.4050349
sp|P84243|H33_HUMAN	-0.3545895	1.6960104
sp|P00325|ADH1B_HUMAN	-0.3475304	3.2450993
sp|Q9H0U4|RAB1B_HUMAN	-0.3448601	1.3815327
sp|P05556|ITB1_HUMAN	-0.3443565	4.899806
sp|P31949|S10AB_HUMAN	-0.3413658	2.4829872
sp|Q9UHG3|PCYOX_HUMAN	-0.3219681	2.894491
sp|P04040|CATA_HUMAN	-0.3154316	4.974138
sp|O00159|MYO1C_HUMAN	-0.3077164	3.9690554
sp|Q7Z406-2|MYH14_HUMAN	-0.2855721	2.918292
sp|Q96TC7|RMD3_HUMAN	-0.2838249	1.6997428
sp|Q9UDY2-3|ZO2_HUMAN	-0.27454	1.7451575
sp|P04179-4|SODM_HUMAN	-0.266674	1.8444856
sp|P51149|RAB7A_HUMAN	-0.2517967	1.6545225
sp|P14543-2|NID1_HUMAN	-0.2501488	1.6208863
sp|A6NMZ7|CO6A6_HUMAN	-0.2481041	2.629145
sp|O60437|PEPL_HUMAN	-0.2464066	1.8593699
sp|P10253|LYAG_HUMAN	-0.2394714	2.9306097
sp|Q13228-4|SBP1_HUMAN	-0.2341557	1.3849562
sp|P55268|LAMB2_HUMAN	-0.2166557	2.10832
sp|P42167-2|LAP2B_HUMAN	-0.2158413	1.5054473
sp|P09960|LKHA4_HUMAN	-0.2085648	2.2420065
sp|P07988|PSPB_HUMAN	-0.2075329	3.0139608
sp|P42765|THIM_HUMAN	-0.2022533	2.0527554
sp|Q9NZN4|EHD2_HUMAN	-0.1955891	1.4936472
sp|Q01082|SPTB2_HUMAN	-0.189949	2.279627
sp|P35908|K22E_HUMAN	0.15756989	4.827397
sp|P07305|H10_HUMAN	0.16381264	1.4104178
sp|P35527|K1C9_HUMAN	0.16681099	2.0528736
sp|P02730|B3AT_HUMAN	0.17526627	2.312627
sp|P01011|AACT_HUMAN	0.18772888	4.505435
sp|P02768|ALBU_HUMAN	0.19329453	11.515463

sp|Q14980-2|NUMA1_HUMAN	0.19429779	1.3332586
sp|Q16629-4|SRSF7_HUMAN	0.20429039	1.3815327
sp|P08238|HS90B_HUMAN	0.22286987	1.7659291
sp|P11021|BIP_HUMAN	0.2579193	2.8963943
sp|P15090|FABP4_HUMAN	0.25868988	2.5644996
sp|P62701|RS4X_HUMAN	0.26350212	2.1818786
sp|P32119|PRDX2_HUMAN	0.26542664	3.2217336
sp|P62424|RL7A_HUMAN	0.28075218	1.867911
sp|O14745|NHRF1_HUMAN	0.28127098	1.7165122
sp|Q12906-2|ILF3_HUMAN	0.28769684	1.3279703
sp|Q15084-5|PDIA6_HUMAN	0.28888607	2.703525
sp|P23456|Trypsin	0.28990936	1.9509854
sp|P39656-3|OST48_HUMAN	0.2981472	2.1404836
sp|P62913|RL11_HUMAN	0.3025341	1.6960104
sp|P04844|RPN2_HUMAN	0.3040371	1.560883
sp|P11277-3|SPTB1_HUMAN	0.3042488	1.6587203
sp|P13645|K1C10_HUMAN	0.30434036	6.57691
sp|P61353|RL27_HUMAN	0.30615234	1.6960104
sp|Q15582|BGH3_HUMAN	0.30750275	3.340024
sp|P19013|K2C4_HUMAN	0.30840683	1.4278674
sp|Q7Z5L7-2|PODN_HUMAN	0.31939316	1.6960104
sp|P23396|RS3_HUMAN	0.31954002	3.7962992
sp|P18124|RL7_HUMAN	0.3200817	1.7703108
sp|O96000-2|NDUBA_HUMAN	0.32143402	1.6960104
sp|P51888|PRELP_HUMAN	0.32188034	3.095067
sp|P35606-2|COPB2_HUMAN	0.3263073	2.9337258
sp|P46781|RS9_HUMAN	0.33075714	1.8146555
sp|O00534|VMA5A_HUMAN	0.33579636	2.0928013
sp|P53621-2|COPA_HUMAN	0.33592987	1.4663166
sp|Q12907|LMAN2_HUMAN	0.33626747	1.3080103
sp|P28838-2|AMPL_HUMAN	0.3380108	4.9486732
sp|P55884-2|EIF3B_HUMAN	0.34392166	1.4936475
sp|P68871|HBB_HUMAN	0.34472656	4.051346
sp|P07900-2|HS90A_HUMAN	0.34499168	2.5153658
sp|Q9UBX5|FBLN5_HUMAN	0.34620094	1.9951487
sp|Q99798|ACON_HUMAN	0.34692764	3.2804387
sp|Q8IZ83-3|A16A1_HUMAN	0.34781647	1.4564189
sp|P09874|PARP1_HUMAN	0.34880257	2.653196
sp|P49419-2|AL7A1_HUMAN	0.350832	3.649813
sp|P07814|SYEP_HUMAN	0.3517475	1.9265962
sp|Q9Y4L1|HYOU1_HUMAN	0.35731316	2.0526927
sp|O95834|EMAL2_HUMAN	0.35865784	2.0727112
sp|P07357|CO8A_HUMAN	0.36079216	2.5461748
sp|P32969|RL9_HUMAN	0.36273193	1.6960104
sp|Q14112-2|NID2_HUMAN	0.36377716	2.987788

sp|P21266|GSTM3_HUMAN	0.36417198	2.1594222
sp|Q9UBS4|DJB11_HUMAN	0.36675453	1.4104178
sp|O15061-2|SYNEM_HUMAN	0.36860085	2.838374
sp|Q06210-2|GFPT1_HUMAN	0.36960983	1.5403327
sp|Q00341|VIGLN_HUMAN	0.36987495	2.2323174
sp|P02042|HBD_HUMAN	0.37037086	3.5908275
sp|P69905|HBA_HUMAN	0.3715973	3.4656596
sp|Q15124|PGM5_HUMAN	0.37667084	3.520947
sp|P07738|PMGE_HUMAN	0.37748337	1.9647322
sp|P02649|APOE_HUMAN	0.38094902	2.7847733
sp|Q15436|SC23A_HUMAN	0.38419724	1.3635377
sp|P15144|AMPN_HUMAN	0.38817024	1.6604291
sp|P04264|K2C1_HUMAN	0.39756775	11.985547
sp|P09493-8|TPM1_HUMAN	0.39928246	1.5054473
sp|O43175|SERA_HUMAN	0.40094376	1.3319147
sp|Q96IJ6-2|GMPPA_HUMAN	0.40485	1.4278674
sp|O00567|NOP56_HUMAN	0.40821648	1.3815327
sp|P14550|AK1A1_HUMAN	0.40859604	1.818702
sp|A0AVT1|UBA6_HUMAN	0.41140366	1.3815327
sp|Q07507|DERM_HUMAN	0.41759872	2.3388627
sp|P23946|CMA1_HUMAN	0.41834068	1.5646582
sp|P40429|RL13A_HUMAN	0.41859055	1.4104178
sp|Q9P2E9|RRBP1_HUMAN	0.41902542	5.3586526
sp|P00966|ASSY_HUMAN	0.42215347	1.4548165
sp|P04843|RPN1_HUMAN	0.42381096	4.3238716
sp|P08670|VIME_HUMAN	0.42474556	12.28203
sp|P35555|FBN1_HUMAN	0.42723083	8.998554
sp|P05155-2|IC1_HUMAN	0.43045425	2.4829872
sp|Q07065|CKAP4_HUMAN	0.430645	7.5853944
sp|O60763-2|USO1_HUMAN	0.43125916	2.3305986
sp|P15880|RS2_HUMAN	0.4337616	3.1266599
sp|P24844|MYL9_HUMAN	0.44065094	1.4104178
sp|Q01995|TAGL_HUMAN	0.4581108	4.578568
sp|P04196|HRG_HUMAN	0.4598465	2.7624686
sp|Q8WX93-5|PALLD_HUMAN	0.46160507	1.7026864
sp|O15127|SCAM2_HUMAN	0.46229553	1.6960104
sp|P14625|ENPL_HUMAN	0.46427727	11.565885
sp|Q16647|PTGIS_HUMAN	0.46470642	1.4278674
sp|Q9UMS6-2|SYNP2_HUMAN	0.46769142	2.2385595
sp|Q92896-2|GSLG1_HUMAN	0.46783257	1.9190748
sp|P67936-2|TPM4_HUMAN	0.46808243	1.9951487
sp|P08727|K1C19_HUMAN	0.46850395	7.105818
sp|Q9UHL4|DPP2_HUMAN	0.47174072	1.4278674
sp|P13667|PDIA4_HUMAN	0.48226738	6.634171
sp|Q9NR12-2|PDLI7_HUMAN	0.48425293	2.044628

sp|Q9Y266|NUDC_HUMAN	0.48587418	1.4104178
sp|Q00796|DHSO_HUMAN	0.48885155	1.4104178
sp|P28331-3|NDUS1_HUMAN	0.4906044	3.1266599
sp|Q8N2S1-3|LTBP4_HUMAN	0.4912567	3.218121
sp|P04217|A1BG_HUMAN	0.4924717	4.7039127
sp|Q96JB5-4|CK5P3_HUMAN	0.49322128	1.4716977
sp|P16615|AT2A2_HUMAN	0.50805855	4.2870226
sp|P20774|MIME_HUMAN	0.5086565	2.3559585
sp|Q92598-2|HS105_HUMAN	0.50919914	1.6832623
sp|P06727|APOA4_HUMAN	0.51867294	5.6770253
sp|P42224-2|STAT1_HUMAN	0.5186825	2.702259
sp|P80303-2|NUCB2_HUMAN	0.5206585	2.1818786
sp|P02748|CO9_HUMAN	0.5214462	3.7671235
sp|Q7KZF4|SND1_HUMAN	0.52262497	3.0251918
sp|P02774-3|VTDB_HUMAN	0.52303886	7.3871665
sp|P49821-2|NDUV1_HUMAN	0.5230732	2.3143692
sp|P62753|RS6_HUMAN	0.52308273	2.1818786
sp|P09493-9|TPM1_HUMAN	0.523304	1.6960104
sp|P02749|APOH_HUMAN	0.52918243	3.1266599
sp|P00915|CAH1_HUMAN	0.53087234	5.792018
sp|Q14767|LTBP2_HUMAN	0.53265	5.742677
sp|P10909-5|CLUS_HUMAN	0.5327606	4.509013
sp|P06681|CO2_HUMAN	0.53534317	2.8739974
sp|P36578|RL4_HUMAN	0.54533386	4.0843706
sp|P84098|RL19_HUMAN	0.54554176	1.4104178
sp|P36955|PEDF_HUMAN	0.5464134	2.987788
sp|P13671|CO6_HUMAN	0.54961777	2.8739974
sp|P20591|MX1_HUMAN	0.5519428	1.6960104
sp|P26373|RL13_HUMAN	0.5578518	2.6576471
sp|P07451|CAH3_HUMAN	0.5697098	2.8384566
sp|Q13162|PRDX4_HUMAN	0.572731	3.1266599
sp|P02766|TTHY_HUMAN	0.579113	1.3815327
sp|P62241|RS8_HUMAN	0.5850105	2.4829872
sp|P22105-1|TENX_HUMAN	0.5887146	10.149043
sp|Q14315-2|FLNC_HUMAN	0.58979416	14.675837
sp|Q14195-2|DPYL3_HUMAN	0.6077366	8.413603
sp|Q9Y265|RUVB1_HUMAN	0.61265945	4.051346
sp|P00488|F13A_HUMAN	0.6133499	5.4724083
sp|P07585|PGS2_HUMAN	0.619297	5.2991114
sp|P27635|RL10_HUMAN	0.6229019	1.6960104
sp|Q5TZA2|CROCC_HUMAN	0.62591934	2.151766
sp|P10643|CO7_HUMAN	0.631794	2.556708
sp|P01019|ANGT_HUMAN	0.6346588	2.4829872
sp|P43652|AFAM_HUMAN	0.63516426	4.4091725
sp|P25311|ZA2G_HUMAN	0.6381264	4.051346

sp|Q96PD5-2|PGRP2_HUMAN	0.6445122	1.6960104
sp|Q9Y230|RUVB2_HUMAN	0.6496773	4.5635147
sp|Q9NR45|SIAS_HUMAN	0.65431786	4.051346
sp|P05156|CFAI_HUMAN	0.66049004	3.5908275
sp|P15559-3|NQO1_HUMAN	0.67393875	1.6960104
sp|P29622|KAIN_HUMAN	0.6818886	2.1818786
sp|P39059|COFA1_HUMAN	0.6894436	2.1818786
sp|P49257|LMAN1_HUMAN	0.7063675	2.1818786
sp|P51884|LUM_HUMAN	0.7253151	8.112776
sp|Q13938-4|CAYP1_HUMAN	0.7299347	4.964395
sp|P01008|ANT3_HUMAN	0.7316532	5.604593
sp|P19823|ITIH2_HUMAN	0.7410965	5.869831
sp|Q9P2B2|FPRP_HUMAN	0.770277	1.4716977
sp|P01031|CO5_HUMAN	0.777935	4.991322
sp|P02675|FIBB_HUMAN	0.79257965	8.504989
sp|P04114|APOB_HUMAN	0.8047943	11.732811
sp|P01042-2|KNG1_HUMAN	0.8206043	5.776476
sp|P02790|HEMO_HUMAN	0.83267975	9.004438
sp|P02679-2|FIBG_HUMAN	0.85416794	5.5742316
sp|P27169|PON1_HUMAN	0.86538506	1.6960104
sp|P02671-2|FIBA_HUMAN	0.871006	9.406504
sp|P00450|CERU_HUMAN	0.87106323	11.15183
sp|Q14624-2|ITIH4_HUMAN	0.88396835	7.2183566
sp|P00734|THRB_HUMAN	0.88994217	5.259211
sp|Q9BW30|TPPP3_HUMAN	0.90703964	3.1266599
sp|Q15063-3|POSTN_HUMAN	0.92873	8.762705
sp|P00747|PLMN_HUMAN	0.938818	5.6000037
sp|Q05707-2|COEA1_HUMAN	0.9609566	14.632371
sp|P19827|ITIH1_HUMAN	0.99650574	2.5644996
sp|P00751|CFAB_HUMAN	1.0556736	12.326815
sp|P05546|HEP2_HUMAN	1.071825	3.1266599
sp|P02652|APOA2_HUMAN	1.078186	1.9413493
sp|P00738-2|HPT_HUMAN	1.1364479	5.417897
sp|P01871-2|IGHM_HUMAN	1.1812077	1.4716977
sp|P08123|CO1A2_HUMAN	1.1951942	2.6576471
sp|P13647|K2C5_HUMAN	1.2192612	4.964395
sp|P02647|APOA1_HUMAN	1.2369003	13.381719
sp|P17661|DESM_HUMAN	1.2759018	14.778499
sp|P01876|IGHA1_HUMAN	1.3102608	4.051346
sp|P02452|CO1A1_HUMAN	1.3388386	2.058973
sp|Q8TDL5|BPIB1_HUMAN	1.3455372	2.2419565
sp|P0DOY2|IGLC2_HUMAN	1.3830357	2.1818786
sp|P04003|C4BPA_HUMAN	1.3915482	4.964395
sp|P01859|IGHG2_HUMAN	1.4165516	4.051346
sp|P01860|IGHG3_HUMAN	1.5109997	2.6576471

sp|P00739-2|HPTR_HUMAN	1.5397644	2.6576471
sp|Q9BXN1|ASPN_HUMAN	1.5830898	1.7590232
sp|P0DOX5|IGG1_HUMAN	1.6106529	4.509013
sp|Q8WU39|MZB1_HUMAN	1.66259	1.6960104
sp|P0DOX8|IGL1_HUMAN	1.7183723	2.6576471
sp|P15088|CBPA3_HUMAN	1.8788643	2.6576471
sp|Q04826|1B40_HUMAN	-2.7332363	0.656254
sp|Q01518-2|CAP1_HUMAN	-2.4013128	0.656254
sp|P30453|1A34_HUMAN	-1.5764227	0.656254
sp|P04229|2B11_HUMAN	-1.361269	0.7827403
sp|Q9BXM0|PRAX_HUMAN	-1.3528557	0.7827403
sp|P10316|1A69_HUMAN	-1.1327953	0.656254
sp|P13760|2B14_HUMAN	-1.0903473	0.91601294
sp|P37235|HPCL1_HUMAN	-1.0884094	0.656254
sp|Q16777|H2A2C_HUMAN	-1.0067043	0.656254
sp|P15428-5|PGDH_HUMAN	-1.0043144	0.91601294
sp|P49913|CAMP_HUMAN	-0.8804131	1.1932944
sp|O00757|F16P2_HUMAN	-0.8454495	0.7827403
sp|P04908|H2A1B_HUMAN	-0.8253841	0.656254
sp|P08246|ELNE_HUMAN	-0.7930603	1.1932944
sp|Q96AP7|ESAM_HUMAN	-0.7297859	0.7827403
sp|P13284|GILT_HUMAN	-0.7143536	1.1932944
sp|Q07075|AMPE_HUMAN	-0.6946716	1.1505735
sp|Q03135-2|CAV1_HUMAN	-0.6821823	0.656254
sp|Q14956-2|GPNMB_HUMAN	-0.6606026	1.1932944
sp|Q31612|1B73_HUMAN	-0.6392098	0
sp|Q9ULC5-3|ACSL5_HUMAN	-0.5783138	0.656254
sp|P13686|PPA5_HUMAN	-0.5460091	0.91601294
sp|P09601|HMOX1_HUMAN	-0.5420981	0.7061832
sp|P24158|PRTN3_HUMAN	-0.5192642	1.1505735
sp|Q10589-2|BST2_HUMAN	-0.5155411	1.1932944
sp|Q03135|CAV1_HUMAN	-0.4722023	0.7588735
sp|P06753|TPM3_HUMAN	-0.470295	0.656254
sp|Q9NVJ2|ARL8B_HUMAN	-0.4462185	0.45033538
sp|O43615|TIM44_HUMAN	-0.4450645	0.7827403
sp|Q9H2U2-2|IPYR2_HUMAN	-0.4391174	1.1932944
sp|P02462|CO4A1_HUMAN	-0.4389906	1.1791906
sp|P51608|MECP2_HUMAN	-0.4376831	1.1932944
sp|Q8WWI1-5|LMO7_HUMAN	-0.4296417	0.7957244
sp|P13498|CY24A_HUMAN	-0.4170971	0.7827403
sp|P62714|PP2AB_HUMAN	-0.4122963	0.656254
sp|P06396-2|GELS_HUMAN	-0.3984642	0
sp|O95837|GNA14_HUMAN	-0.3984032	0.656254
sp|P02792|FRIL_HUMAN	-0.3898068	0.87291557
sp|P61026|RAB10_HUMAN	-0.3845444	0.656254

sp|Q13231-2|CHIT1_HUMAN	-0.3775559	0.6070219
sp|P04440|DPB1_HUMAN	-0.3734894	0.7827403
sp|Q99715-4|COCA1_HUMAN	-0.3639832	0.69308156
sp|P06702|S10A9_HUMAN	-0.3631573	0.5896263
sp|P25774|CATS_HUMAN	-0.3610477	0.7827403
sp|O43760-2|SNG2_HUMAN	-0.3581982	0.45033538
sp|Q15599-2|NHRF2_HUMAN	-0.357296	0.8983557
sp|Q9UPN3|MACF1_HUMAN	-0.3403549	0.35795313
sp|P06899|H2B1J_HUMAN	-0.3392944	1.1932944
sp|P05109|S10A8_HUMAN	-0.3323383	0.09339783
sp|Q53FA7|QORX_HUMAN	-0.3318386	0.19149946
sp|P38159|RBMX_HUMAN	-0.3316174	1.1932944
sp|O95716|RAB3D_HUMAN	-0.3268147	0.656254
sp|Q96HD1-2|CREL1_HUMAN	-0.3237953	1.1932944
sp|P62070-4|RRAS2_HUMAN	-0.3237038	0
sp|P11279|LAMP1_HUMAN	-0.3221149	1.1932944
sp|Q9Y3D6|FIS1_HUMAN	-0.3191748	0.91601294
sp|P20702|ITAX_HUMAN	-0.3174076	0.6070219
sp|O15296|LX15B_HUMAN	-0.3167534	0
sp|P51153|RAB13_HUMAN	-0.3104134	0
sp|Q86Y82|STX12_HUMAN	-0.2986774	1.1791906
sp|Q9Y2Q5|LTOR2_HUMAN	-0.2978172	0.7827403
sp|P01920|DQB1_HUMAN	-0.2950306	0
sp|P11686-2|PSPC_HUMAN	-0.2927246	0.09894868
sp|Q9NTX5-3|ECHD1_HUMAN	-0.2858315	0.8059303
sp|P33121-3|ACSL1_HUMAN	-0.282135	0.6509351
sp|P04080|CYTB_HUMAN	-0.2811546	0
sp|Q9NX46|ARHL2_HUMAN	-0.2797604	0.45033538
sp|Q6YN16|HSDL2_HUMAN	-0.2741604	1.1344727
sp|P20340-2|RAB6A_HUMAN	-0.2734051	0.656254
sp|P05107|ITB2_HUMAN	-0.2725143	0.96367663
sp|P05534|1A24_HUMAN	-0.2672291	0
sp|P20292|AL5AP_HUMAN	-0.2663889	0.7827403
sp|P62314|SMD1_HUMAN	-0.266099	0.19149946
sp|P61019|RAB2A_HUMAN	-0.2644978	0.5204253
sp|Q9NNW7|TRXR2_HUMAN	-0.2635613	0.5111962
sp|Q05682-4|CALD1_HUMAN	-0.2618351	0
sp|Q9P289|STK26_HUMAN	-0.2614012	0.656254
sp|Q15907|RB11B_HUMAN	-0.261322	0.9968222
sp|Q9P2M7|CING_HUMAN	-0.2542343	0.7588735
sp|P26038|MOES_HUMAN	-0.2539578	0.1446394
sp|P67809|YBOX1_HUMAN	-0.2513628	0.45033538
sp|P0DP25|CALM3_HUMAN	-0.249157	0.44572112
sp|Q15286-2|RAB35_HUMAN	-0.2487011	0
sp|Q13813-2|SPTN1_HUMAN	-0.2475433	0.2178309

sp|P09429|HMGB1_HUMAN	-0.2467041	0.7827403
sp|Q13636|RAB31_HUMAN	-0.2456513	0.5204253
sp|Q8N9N7|LRC57_HUMAN	-0.2454662	0
sp|P17213|BPI_HUMAN	-0.2443886	0
sp|P18428|LBP_HUMAN	-0.241457	0.40256184
sp|A0MZ66-5|SHOT1_HUMAN	-0.2408466	0.19149946
sp|P28676|GRAN_HUMAN	-0.2372646	0.35193655
sp|O75695|XRP2_HUMAN	-0.2362232	0.09894868
sp|Q04837|SSBP_HUMAN	-0.2353325	0.91601294
sp|P49327|FAS_HUMAN	-0.2350369	0.50552267
sp|P13746|1A11_HUMAN	-0.2346973	0.7827403
sp|O75348|VATG1_HUMAN	-0.2327538	0
sp|Q02318|CP27A_HUMAN	-0.2324886	0.7827403
sp|P23381|SYWC_HUMAN	-0.2323132	0.97180504
sp|O15247|CLIC2_HUMAN	-0.2321835	0.51870745
sp|Q9UBQ0-2|VPS29_HUMAN	-0.2283859	0.19149946
sp|A5A3E0|POTEF_HUMAN	-0.2278156	0
sp|Q99536|VAT1_HUMAN	-0.2268047	0.12054814
sp|O60234|GMFG_HUMAN	-0.2244225	0.33495146
sp|P00403|COX2_HUMAN	-0.2230263	0.19149946
sp|Q04760-2|LGUL_HUMAN	-0.2190781	0.21439649
sp|P36543-2|VATE1_HUMAN	-0.218977	0.35795313
sp|P13804-2|ETFA_HUMAN	-0.2185745	0.14672586
sp|P55196-5|AFAD_HUMAN	-0.2164002	0.25789237
sp|Q13813-3|SPTN1_HUMAN	-0.2160187	0
sp|P26440|IVD_HUMAN	-0.2155075	0
sp|P01116-2|RASK_HUMAN	-0.2148056	0
sp|Q01518|CAP1_HUMAN	-0.2132626	0
sp|P11047|LAMC1_HUMAN	-0.2131538	0.51660806
sp|Q02750|MP2K1_HUMAN	-0.2099381	0
sp|Q92522|H1X_HUMAN	-0.20965	0.19149946
sp|P13987-2|CD59_HUMAN	-0.209425	0.45033538
sp|P62937|PPIA_HUMAN	-0.2088661	0.2178309
sp|Q9UBR2|CATZ_HUMAN	-0.2085934	0
sp|P13797|PLST_HUMAN	-0.208046	0.28186345
sp|Q15833-2|STXB2_HUMAN	-0.2073774	0.35795313
sp|P43304|GPDM_HUMAN	-0.2010727	0.54687065
sp|P38606-2|VATA_HUMAN	-0.2005234	0.10280301
sp|P09758|TACD2_HUMAN	-0.1997929	0.3005443
sp|Q14254|FLOT2_HUMAN	-0.198698	0.95533764
sp|Q13751|LAMB3_HUMAN	-0.1986713	0.1342476
sp|P16219|ACADS_HUMAN	-0.1986637	0.06291623
sp|P59998|ARPC4_HUMAN	-0.1982117	0.52598757
sp|P20160|CAP7_HUMAN	-0.1969872	0.7061832
sp|P61421|VA0D1_HUMAN	-0.1955948	0.10861364

sp|Q15121|PEA15_HUMAN	-0.1946068	0.5204253
sp|P0CG47|UBB_HUMAN	-0.1939488	0.84879977
sp|Q99598|TSNAX_HUMAN	-0.1928024	0.5204253
sp|Q6P4A8|PLBL1_HUMAN	-0.1926003	0.35795313
sp|P05141|ADT2_HUMAN	-0.1914158	0.4075265
sp|O15230|LAMA5_HUMAN	-0.1896324	0.5717351
sp|Q8IV08|PLD3_HUMAN	-0.1895046	0.33495146
sp|P51648-2|AL3A2_HUMAN	-0.1880283	0.70235044
sp|Q9UM07|PADI4_HUMAN	-0.1870537	0.5204253
sp|P13761|2B17_HUMAN	-0.1866264	0
sp|Q15126|PMVK_HUMAN	-0.1846809	0.45033538
sp|O00499-10|BIN1_HUMAN	-0.184474	0.19149946
sp|O95833|CLIC3_HUMAN	-0.1815834	0.14672586
sp|O43491|E41L2_HUMAN	-0.1803494	0.35262254
sp|Q96MM6|HS12B_HUMAN	-0.1798859	0.9780152
sp|P61586|RHOA_HUMAN	-0.1798172	0
sp|Q16853|AOC3_HUMAN	-0.1791344	0.65625405
sp|P36542-2|ATPG_HUMAN	-0.1789951	0
sp|P08572|CO4A2_HUMAN	-0.1770325	0.7498006
sp|P30740|ILEU_HUMAN	-0.1758518	0.051214
sp|P08729|K2C7_HUMAN	-0.1758156	0.26971197
sp|Q96AC1|FERM2_HUMAN	-0.1756392	0.63267636
sp|Q9UL25|RAB21_HUMAN	-0.1679916	0.21439649
sp|P80188|NGAL_HUMAN	-0.1668739	0.39873424
sp|P40121|CAPG_HUMAN	-0.1665344	0
sp|Q9NPJ3-2|ACO13_HUMAN	-0.1662064	0.45033538
sp|P06865|HEXA_HUMAN	-0.1651917	0.7588735
sp|Q8N335|GPD1L_HUMAN	-0.1651459	0.30372584
sp|P51452-2|DUS3_HUMAN	-0.1648312	0
sp|P49593|PPM1F_HUMAN	-0.1646004	0.06291623
sp|Q9NV96-3|CC50A_HUMAN	-0.1641808	0
sp|P30048-2|PRDX3_HUMAN	-0.1641712	0.09339783
sp|P62820|RAB1A_HUMAN	-0.1632423	0.09339783
sp|Q13308-6|PTK7_HUMAN	-0.1622677	0.34137914
sp|P04839|CY24B_HUMAN	-0.1620445	0
sp|Q16698-2|DECR_HUMAN	-0.1618881	0.4918224
sp|P61006|RAB8A_HUMAN	-0.1614361	0
sp|P13796|PLSL_HUMAN	-0.1610031	0.66367525
sp|O75955-2|FLOT1_HUMAN	-0.1604767	0.46530083
sp|Q6WCQ1-2|MPRIP_HUMAN	-0.1604233	0.6563392
sp|O60814|H2B1K_HUMAN	-0.1600323	1.1932944
sp|P07355|ANXA2_HUMAN	-0.1594715	0.10328458
sp|P07686|HEXB_HUMAN	-0.1592484	0.9968222
sp|P05026-2|AT1B1_HUMAN	-0.1579571	0.20467198
sp|O94760|DDAH1_HUMAN	-0.1567936	0

sp|Q32MZ4-3|LRRF1_HUMAN	-0.1566162	0
sp|Q15181|IPYR_HUMAN	-0.155365	0
sp|P50148|GNAQ_HUMAN	-0.1553612	0.21439649
sp|P68036-3|UB2L3_HUMAN	-0.1542759	0
sp|Q9Y376|CAB39_HUMAN	-0.154171	0.1558116
sp|Q7LG56-6|RIR2B_HUMAN	-0.1503515	0
sp|O00423-3|EMAL1_HUMAN	-0.1501179	0.5204253
sp|P07910-2|HNRPC_HUMAN	-0.1489639	0.7498006
sp|P60953|CDC42_HUMAN	-0.1486454	0.06842031
sp|Q09666|AHNK_HUMAN	-0.1477814	0.45600381
sp|Q8NBQ5|DHB11_HUMAN	-0.1472149	0.7217418
sp|P61224|RAP1B_HUMAN	-0.1463175	0
sp|P35221|CTNA1_HUMAN	-0.1460552	0.5992246
sp|P23528|COF1_HUMAN	-0.1460075	0.1804695
sp|Q13188|STK3_HUMAN	-0.1452313	0
sp|P28074|PSB5_HUMAN	-0.1450176	0
sp|P16278-2|BGAL_HUMAN	-0.1444321	0.35795313
sp|Q99729-2|ROAA_HUMAN	-0.1431179	0
sp|Q92973-2|TNPO1_HUMAN	-0.1421681	0.43633315
sp|Q07866-10|KLC1_HUMAN	-0.1418419	0
sp|P25815|S100P_HUMAN	-0.1413727	0
sp|P36551|HEM6_HUMAN	-0.1411781	0
sp|Q15404|RSU1_HUMAN	-0.1409779	0.22362706
sp|P27105|STOM_HUMAN	-0.1405811	0.7284051
sp|Q00839|HNRPU_HUMAN	-0.1404381	0.45033535
sp|Q6DKJ4|NXN_HUMAN	-0.1403933	0
sp|P49756|RBM25_HUMAN	-0.1394854	0.19149946
sp|P84103-2|SRSF3_HUMAN	-0.1394787	0.65625405
sp|Q6IBS0|TWF2_HUMAN	-0.1390801	0
sp|Q6P587-3|FAHD1_HUMAN	-0.1388702	0.2178309
sp|Q00577|PURA_HUMAN	-0.1387482	0.21439649
sp|O75155|CAND2_HUMAN	-0.1383896	0
sp|Q9H8L6|MMRN2_HUMAN	-0.1381283	0.312067
sp|Q9P0K7-2|RAI14_HUMAN	-0.137394	0
sp|P14780|MMP9_HUMAN	-0.1359158	0.26737198
sp|P60033|CD81_HUMAN	-0.1351204	0
sp|P10412|H14_HUMAN	-0.1341629	0.45720983
sp|P24752|THIL_HUMAN	-0.1335087	0.12246842
sp|Q15366-2|PCBP2_HUMAN	-0.133461	0.09894868
sp|Q9H4M9|EHD1_HUMAN	-0.1327725	0
sp|Q9BWD1|THIC_HUMAN	-0.1321735	0
sp|O15400-2|STX7_HUMAN	-0.1295662	0.25789237
sp|Q9BXP5-5|SRRT_HUMAN	-0.1293926	0.35193655
sp|P51148-2|RAB5C_HUMAN	-0.1282959	0
sp|P15170-2|ERF3A_HUMAN	-0.1280727	0

sp|Q15417|CNN3_HUMAN	-0.1280289	0
sp|P07942|LAMB1_HUMAN	-0.1263256	0.10280301
sp|Q6P179|ERAP2_HUMAN	-0.1256104	0.25789237
sp|P78347-2|GTF2I_HUMAN	-0.1256104	0.19149946
sp|Q92882|OSTF1_HUMAN	-0.1247072	0.06291623
sp|P16435|NCPR_HUMAN	-0.1244965	0.12893641
sp|O43795-2|MYO1B_HUMAN	-0.1244068	0.17511293
sp|Q16822|PCKGM_HUMAN	-0.1243687	0
sp|P52943|CRIP2_HUMAN	-0.1239185	0.1342476
sp|P12235|ADT1_HUMAN	-0.1236782	0
sp|Q14533|KRT81_HUMAN	-0.1233578	0
sp|Q13555-7|KCC2G_HUMAN	-0.1224794	0
sp|Q9HDC9|APMAP_HUMAN	-0.1219988	0.08307569
sp|P63220|RS21_HUMAN	-0.1215763	0
sp|Q5T013-4|HYI_HUMAN	-0.1214714	0
sp|Q9UBW8|CSN7A_HUMAN	-0.1214581	0.09894868
sp|P20339-2|RAB5A_HUMAN	-0.1209717	0.1558116
sp|P26599-2|PTBP1_HUMAN	-0.1208515	0.26737198
sp|Q9Y5X3|SNX5_HUMAN	-0.1201363	0.1500082
sp|P06753-2|TPM3_HUMAN	-0.1197014	0.13745423
sp|O14786|NRP1_HUMAN	-0.1195717	0.09894868
sp|Q15056-2|IF4H_HUMAN	-0.1193123	0.19149946
sp|Q9NR31|SAR1A_HUMAN	-0.1192932	0.19149946
sp|Q02083-2|NAAA_HUMAN	-0.1172504	0
sp|O15144|ARPC2_HUMAN	-0.1170979	0.24295025
sp|Q13232|NDK3_HUMAN	-0.1167564	0
sp|Q99436|PSB7_HUMAN	-0.115757	0
sp|P60983|GMFB_HUMAN	-0.1150188	0
sp|P55010|IF5_HUMAN	-0.1144867	0
sp|P11215-2|ITAM_HUMAN	-0.1135349	0.37060758
sp|P17931|LEG3_HUMAN	-0.1134224	0
sp|P62834|RAP1A_HUMAN	-0.1102219	0
sp|Q9Y281|COF2_HUMAN	-0.1090565	0
sp|P78417|GSTO1_HUMAN	-0.1088715	0.15332921
sp|P52566|GDIR2_HUMAN	-0.1078758	0.18414244
sp|P61604|CH10_HUMAN	-0.1078606	0
sp|O15511|ARPC5_HUMAN	-0.1068878	0
sp|Q9Y6N5|SQOR_HUMAN	-0.1067276	0.2810592
sp|P37837|TALDO_HUMAN	-0.1060047	0.11202396
sp|Q9UHB6-4|LIMA1_HUMAN	-0.1052265	0
sp|Q9BTZ2|DHRS4_HUMAN	-0.1048012	0.09894868
sp|A0A0B4J2D5|GAL3B_HUMAN	-0.1037006	0
sp|P23634-2|AT2B4_HUMAN	-0.1036205	0.03367973
sp|Q6NUK1-2|SCMC1_HUMAN	-0.1034603	0.04454162
sp|P61088|UBE2N_HUMAN	-0.1034565	0

sp|O00468-7|AGRIN_HUMAN	-0.1026421	0
sp|Q53GQ0|DHB12_HUMAN	-0.1023579	0.20467198
sp|P12694-2|ODBA_HUMAN	-0.1016598	0
sp|P51636-2|CAV2_HUMAN	-0.1014309	0.19149946
sp|P62318-2|SMD3_HUMAN	-0.1003876	0.19149946
sp|P40926|MDHM_HUMAN	-0.0999737	0.3556601
sp|Q3KQV9|UAP1L_HUMAN	-0.0996733	0.45033538
sp|P61601|NCALD_HUMAN	-0.0993519	0
sp|Q99459|CDC5L_HUMAN	-0.098444	0
sp|O15498-2|YKT6_HUMAN	-0.0978489	0
sp|Q13011|ECH1_HUMAN	-0.0975323	0.33495146
sp|Q02218-2|ODO1_HUMAN	-0.0966034	0.01343579
sp|P50225|ST1A1_HUMAN	-0.0959721	0
sp|Q6NY19-2|KANK3_HUMAN	-0.0958328	0.43633315
sp|P30040|ERP29_HUMAN	-0.0956612	0.31759265
sp|Q14118|DAG1_HUMAN	-0.0937271	0
sp|P61769|B2MG_HUMAN	-0.0934792	0
sp|Q15942|ZYX_HUMAN	-0.0933533	0.07727676
sp|P61020|RAB5B_HUMAN	-0.093235	0
sp|Q14344|GNA13_HUMAN	-0.0927734	0
sp|P10599-2|THIO_HUMAN	-0.0927486	0
sp|P50213|IDH3A_HUMAN	-0.0919552	0
sp|P62993|GRB2_HUMAN	-0.09058	0
sp|Q9UFN0|NPS3A_HUMAN	-0.0905609	0.43633315
sp|P11413-3|G6PD_HUMAN	-0.0902538	0
sp|P08311|CATG_HUMAN	-0.0900612	0.44572112
sp|P60981-2|DEST_HUMAN	-0.0893822	0
sp|Q16658|FSCN1_HUMAN	-0.0887184	0.22784016
sp|Q13492-2|PICAL_HUMAN	-0.0885696	0
sp|Q96TA1-2|NIBL1_HUMAN	-0.0882149	0
sp|P10301|RRAS_HUMAN	-0.0869656	0.35795313
sp|P07948-2|LYN_HUMAN	-0.0867424	0
sp|O75390|CISY_HUMAN	-0.0861015	0.11094732
sp|P02750|A2GL_HUMAN	-0.0860615	0
sp|O43707|ACTN4_HUMAN	-0.0860424	0.04597474
sp|O75915|PRAF3_HUMAN	-0.0859671	0.21439649
sp|P07858|CATB_HUMAN	-0.0843411	0.356152
sp|P12236|ADT3_HUMAN	-0.0843067	0
sp|P53634|CATC_HUMAN	-0.0834293	0.1342476
sp|P37802|TAGL2_HUMAN	-0.0833855	0.01044662
sp|P28066|PSA5_HUMAN	-0.0830326	0
sp|P28070|PSB4_HUMAN	-0.0822239	0.33495146
sp|Q9ULV4-3|COR1C_HUMAN	-0.0820999	0
sp|Q9H845|ACAD9_HUMAN	-0.0813675	0
sp|P21796|VDAC1_HUMAN	-0.0810242	0

sp|Q15369-2|ELOC_HUMAN	-0.080286	0
sp|Q8N1B4-2|VPS52_HUMAN	-0.0798836	0.19149946
sp|P09211|GSTP1_HUMAN	-0.0787926	0
sp|Q16630-3|CPSF6_HUMAN	-0.0786152	0.09894868
sp|P84077|ARF1_HUMAN	-0.0760727	0.06291623
sp|O95340-2|PAPS2_HUMAN	-0.0758724	0.14927356
sp|P22695|QCR2_HUMAN	-0.0757904	0
sp|P28482|MK01_HUMAN	-0.075119	0
sp|P00568|KAD1_HUMAN	-0.0748978	0
sp|O60504-2|VINEX_HUMAN	-0.0747032	0.09894868
sp|P30046|DOPD_HUMAN	-0.0733147	0
sp|Q16363-2|LAMA4_HUMAN	-0.0730095	0.04904167
sp|P36957|ODO2_HUMAN	-0.0727768	0
sp|Q13740-2|CD166_HUMAN	-0.0727024	0.11075022
sp|Q6YHK3-4|CD109_HUMAN	-0.0726929	0.09894868
sp|Q9H0R4|HDHD2_HUMAN	-0.0713644	0
sp|Q9H299|SH3L3_HUMAN	-0.069746	0
sp|Q13177|PAK2_HUMAN	-0.0695667	0
sp|Q04941|PLP2_HUMAN	-0.0690918	0
sp|P62873|GBB1_HUMAN	-0.0681915	0
sp|P45880-2|VDAC2_HUMAN	-0.0673237	0.05621411
sp|P43490|NAMPT_HUMAN	-0.0672569	0.01044662
sp|Q7Z4I7-3|LIMS2_HUMAN	-0.0671597	0.1558116
sp|Q15365|PCBP1_HUMAN	-0.0667362	0
sp|P54819-2|KAD2_HUMAN	-0.0666943	0.06291623
sp|Q9NX63|MIC19_HUMAN	-0.0662041	0.04454162
sp|Q16775-2|GLO2_HUMAN	-0.0657921	0
sp|O75367-2|H2AY_HUMAN	-0.0655289	0.7173901
sp|Q14011|CIRBP_HUMAN	-0.0650826	0
sp|P15311|EZRI_HUMAN	-0.0648251	0
sp|Q16762|THTR_HUMAN	-0.064209	0
sp|Q16555|DPYL2_HUMAN	-0.0632153	0.00697919
sp|O60825-2|F262_HUMAN	-0.0624828	0
sp|P30519|HMOX2_HUMAN	-0.0616455	0.06291623
sp|Q9NZL9-2|MAT2B_HUMAN	-0.061552	0
sp|P42330|AK1C3_HUMAN	-0.0611553	0
sp|P63261|ACTG_HUMAN	-0.0602112	0
sp|Q86TX2|ACOT1_HUMAN	-0.0600452	0
sp|Q9NYL9|TMOD3_HUMAN	-0.059082	0.26737198
sp|Q99439|CNN2_HUMAN	-0.0587101	0
sp|Q99758|ABCA3_HUMAN	-0.0586472	0.12897664
sp|Q9HCC0-2|MCCB_HUMAN	-0.058363	0
sp|P07996|TSP1_HUMAN	-0.057539	0.11949348
sp|Q92930|RAB8B_HUMAN	-0.0572701	0
sp|P99999|CYC_HUMAN	-0.0570908	0.17511293

sp|Q8TC12-2|RDH11_HUMAN	-0.0570679	0
sp|Q7Z4W1|DCXR_HUMAN	-0.0556526	0.2207585
sp|O00186|STXB3_HUMAN	-0.0546236	0
sp|Q96C23|GALM_HUMAN	-0.0544777	0
sp|P50552|VASP_HUMAN	-0.0520458	0.1063047
sp|P25787|PSA2_HUMAN	-0.051259	0.09339783
sp|P23141-3|EST1_HUMAN	-0.050314	0.3280279
sp|Q02252-2|MMSA_HUMAN	-0.0499554	0
sp|P16070-16|CD44_HUMAN	-0.0492497	0
sp|P23246|SFPQ_HUMAN	-0.048542	0.01178462
sp|Q00169|PIPNA_HUMAN	-0.0477562	0
sp|P08397-2|HEM3_HUMAN	-0.0477161	0
sp|P51572-2|BAP31_HUMAN	-0.0475101	0
sp|P47755|CAZA2_HUMAN	-0.0473042	0
sp|O75368|SH3L1_HUMAN	-0.0472336	0
sp|Q9UJ70-2|NAGK_HUMAN	-0.0471306	0
sp|P60660-2|MYL6_HUMAN	-0.0464764	0.02174174
sp|P29992|GNA11_HUMAN	-0.0463944	0
sp|Q9BV20|MTNA_HUMAN	-0.0460339	0
sp|Q9UBF2|COPG2_HUMAN	-0.0459118	0
sp|P01861|IGHG4_HUMAN	-0.0455151	0.09339783
sp|P0C0S5|H2AZ_HUMAN	-0.0445843	0.656254
sp|Q02818|NUCB1_HUMAN	-0.0444717	0.13242109
sp|Q86X76-2|NIT1_HUMAN	-0.0444679	0
sp|Q9H6R3|ACSS3_HUMAN	-0.0442333	0
sp|P36542|ATPG_HUMAN	-0.0442181	0
sp|Q53H82|LACB2_HUMAN	-0.0441055	0
sp|Q5JWF2-2|GNAS1_HUMAN	-0.0434113	0.14672586
sp|Q13185|CBX3_HUMAN	-0.0433979	0
sp|O95831-3|AIFM1_HUMAN	-0.042366	0
sp|P15153|RAC2_HUMAN	-0.0420895	0
sp|O95865|DDAH2_HUMAN	-0.0420628	0.10943281
sp|Q01105-2|SET_HUMAN	-0.0419197	0
sp|P29401-2|TKT_HUMAN	-0.0417862	0.00283325
sp|P84095|RHOG_HUMAN	-0.0417824	0.09894868
sp|P43121|MUC18_HUMAN	-0.0415993	0.09894868
sp|P07737|PROF1_HUMAN	-0.0405941	0.01542275
sp|O75923-15|DYSF_HUMAN	-0.0405731	0
sp|Q9UBQ7|GRHPR_HUMAN	-0.0393753	0
sp|P35754|GLRX1_HUMAN	-0.0369988	0
sp|P07099|HYEP_HUMAN	-0.0364685	0.07334998
sp|Q9HBL0|TENS1_HUMAN	-0.035553	0.03357808
sp|Q9UHQ9|NB5R1_HUMAN	-0.0352039	0
sp|Q969H8|MYDGF_HUMAN	-0.0350952	0
sp|P42126-2|ECI1_HUMAN	-0.0346661	0

sp|Q15435|PP1R7_HUMAN	-0.0337143	0
sp|P52565|GDIR1_HUMAN	-0.0334282	0
sp|P28072|PSB6_HUMAN	-0.0333176	0
sp|P62826|RAN_HUMAN	-0.0331192	0
sp|P35914|HMGCL_HUMAN	-0.0307579	0
sp|Q9UIJ7|KAD3_HUMAN	-0.02985	0
sp|P09497-2|CLCB_HUMAN	-0.029747	0
sp|P54619-2|AAKG1_HUMAN	-0.0290756	0
sp|P09382|LEG1_HUMAN	-0.0286884	0.07727676
sp|Q9BTW9-4|TBCD_HUMAN	-0.0284729	0.35795313
sp|P09651-3|ROA1_HUMAN	-0.0281925	0
sp|P09622|DLDH_HUMAN	-0.0281639	0
sp|O95425-4|SVIL_HUMAN	-0.0280705	0.19149946
sp|Q92597|NDRG1_HUMAN	-0.0280476	0
sp|P00367|DHE3_HUMAN	-0.0277596	0.1804695
sp|Q99829|CPNE1_HUMAN	-0.027256	0
sp|P35237|SPB6_HUMAN	-0.0268784	0
sp|O00764-2|PDXK_HUMAN	-0.0257845	0
sp|Q9Y696|CLIC4_HUMAN	-0.0255718	0
sp|O60488-2|ACSL4_HUMAN	-0.025095	0
sp|P48449-3|ERG7_HUMAN	-0.024744	0.09339783
sp|Q9P0V9-2|SEP10_HUMAN	-0.0247288	0.19410844
sp|Q8IWB7|WDFY1_HUMAN	-0.0238762	0
sp|Q9NQC3|RTN4_HUMAN	-0.0238743	0.4075265
sp|P60900|PSA6_HUMAN	-0.0237389	0
sp|P17612|KAPCA_HUMAN	-0.0231209	0
sp|P35232|PHB_HUMAN	-0.0230751	0.10280301
sp|P16671-4|CD36_HUMAN	-0.0226364	0
sp|Q13126-2|MTAP_HUMAN	-0.0224609	0
sp|Q06830|PRDX1_HUMAN	-0.0222092	0
sp|O94925-3|GLSK_HUMAN	-0.0220337	0
sp|Q63ZY3-3|KANK2_HUMAN	-0.021616	0.10017801
sp|P35222|CTNB1_HUMAN	-0.0215702	0
sp|P06576|ATPB_HUMAN	-0.0206337	0.02802646
sp|P35579|MYH9_HUMAN	-0.0205517	0.08383793
sp|O75131|CPNE3_HUMAN	-0.0202427	0
sp|Q14103-3|HNRPD_HUMAN	-0.020031	0.2174103
sp|Q9UBI6|GBG12_HUMAN	-0.0200176	0
sp|P21964-2|COMT_HUMAN	-0.0195427	0.05681695
sp|P06733|ENOA_HUMAN	-0.0193062	0
sp|Q86UX7-2|URP2_HUMAN	-0.0184803	0
sp|P07437|TBB5_HUMAN	-0.01824	0
sp|P12111|CO6A3_HUMAN	-0.0179119	0.06534385
sp|P43034|LIS1_HUMAN	-0.0177155	0
sp|Q96EE3-1|SEH1_HUMAN	-0.0176563	0

sp|Q9Y3A3-3|PHOCN_HUMAN	-0.0176315	0
sp|Q16787-3|LAMA3_HUMAN	-0.0172977	0.19149946
sp|Q30134|2B18_HUMAN	-0.0172691	0
sp|P08575-10|PTPRC_HUMAN	-0.0172577	0.03712561
sp|P02545|LMNA_HUMAN	-0.0169697	0
sp|P58546|MTPN_HUMAN	-0.0166969	0
sp|Q9NUJ1|ABHDA_HUMAN	-0.0163937	0
sp|Q27J81-2|INF2_HUMAN	-0.0160847	0
sp|O00291|HIP1_HUMAN	-0.015564	0
sp|P61978-3|HNRPK_HUMAN	-0.0155582	0.07488067
sp|O15143|ARC1B_HUMAN	-0.0134087	0.16077396
sp|Q9Y4D7-2|PLXD1_HUMAN	-0.0131512	0
sp|O00232|PSD12_HUMAN	-0.0130339	0
sp|P35580|MYH10_HUMAN	-0.0128746	0.22968848
sp|P61960|UFM1_HUMAN	-0.0125237	0.19149946
sp|P14678|RSMB_HUMAN	-0.0124016	0.19149946
sp|P39060-1|COIA1_HUMAN	-0.0120888	0.1329664
sp|Q96I99|SUCB2_HUMAN	-0.0115719	0.05634482
sp|P24539|AT5F1_HUMAN	-0.0113602	0
sp|O95571|ETHE1_HUMAN	-0.0110626	0.12897664
sp|Q00325-2|MPCP_HUMAN	-0.0107231	0.04454162
sp|P06737-2|PYGL_HUMAN	-0.0093956	0
sp|Q13423|NNTM_HUMAN	-0.0086002	0.06695005
sp|Q92542|NICA_HUMAN	-0.0081158	0
sp|P46939-2|UTRO_HUMAN	-0.0080109	0.08490886
sp|P55036-2|PSMD4_HUMAN	-0.0079174	0
sp|P51553-2|IDH3G_HUMAN	-0.0075531	0
sp|O75323|NIPS2_HUMAN	-0.0074463	0
sp|P10606|COX5B_HUMAN	-0.0064774	0
sp|Q8IY17-3|PLPL6_HUMAN	-0.0059624	0
sp|Q9H223|EHD4_HUMAN	-0.0055313	0.12992796
sp|Q9NTK5|OLA1_HUMAN	-0.0051537	0.30372584
sp|P19338|NUCL_HUMAN	-0.0046253	0.05276814
sp|P30041|PRDX6_HUMAN	-0.0042515	0.04897772
sp|Q6P1N9|TATD1_HUMAN	-0.0037842	0
sp|Q09028-3|RBBP4_HUMAN	-0.0037346	0
sp|O43684-2|BUB3_HUMAN	-0.0033245	0
sp|Q08431|MFGM_HUMAN	-0.0025101	0
sp|Q96AG4|LRC59_HUMAN	-0.0024853	0.04454162
sp|O14950|ML12B_HUMAN	-0.0018234	0
sp|P62258|1433E_HUMAN	-9.19E-04	0.01406953
sp|P63241|IF5A1_HUMAN	-2.57E-04	0.17319627
sp|P14923|PLAK_HUMAN	-1.83E-04	0
sp|Q13043|STK4_HUMAN	7.30E-04	0
sp|O00264|PGRC1_HUMAN	0.00156021	0

sp|P27816-2|MAP4_HUMAN	0.00198746	0
sp|Q8TE77-3|SSH3_HUMAN	0.00264931	0
sp|P05023-4|AT1A1_HUMAN	0.00332642	0.02068257
sp|P00387-3|NB5R3_HUMAN	0.00403404	0.01819076
sp|Q9Y5S9-2|RBM8A_HUMAN	0.00444222	0.35795313
sp|P08758|ANXA5_HUMAN	0.00541687	0
sp|P61158|ARP3_HUMAN	0.00552177	0.00952955
sp|Q03252|LMNB2_HUMAN	0.00562668	0.41310343
sp|Q04917|1433F_HUMAN	0.00649834	0
sp|O95372|LYPA2_HUMAN	0.00664902	0
sp|O94832|MYO1D_HUMAN	0.0075779	0
sp|Q9C0C2-2|TB182_HUMAN	0.0075779	0
sp|Q13596-2|SNX1_HUMAN	0.00821877	0
sp|Q9UBV8|PEF1_HUMAN	0.00862312	0
sp|Q13642-1|FHL1_HUMAN	0.0088768	0
sp|Q13884|SNTB1_HUMAN	0.00986671	0
sp|P49748-2|ACADV_HUMAN	0.01005364	0.08626214
sp|P61018|RAB4B_HUMAN	0.01009369	0
sp|Q13561-2|DCTN2_HUMAN	0.0104332	0
sp|Q02978|M2OM_HUMAN	0.01054192	0.03367973
sp|P48047|ATPO_HUMAN	0.01060486	0.35262254
sp|Q6UWY5|OLFL1_HUMAN	0.01118851	0.356152
sp|P22894|MMP8_HUMAN	0.01152992	0
sp|P61225|RAP2B_HUMAN	0.01169395	0
sp|P05198|IF2A_HUMAN	0.01189995	0
sp|P22626|ROA2_HUMAN	0.01221085	0.01700861
sp|Q16204|CCDC6_HUMAN	0.01342773	0
sp|Q8WUY1|THEM6_HUMAN	0.01368332	0.09894868
sp|Q5TDH0-3|DDI2_HUMAN	0.01380539	0
sp|P61981|1433G_HUMAN	0.01423264	0.1329664
sp|Q14165|MLEC_HUMAN	0.01428223	0
sp|Q15370-2|ELOB_HUMAN	0.01439858	0
sp|Q96CX2|KCD12_HUMAN	0.0146637	0
sp|P47985|UCRI_HUMAN	0.01494503	0.09894868
sp|Q99584|S10AD_HUMAN	0.01558876	0.45033538
sp|Q9NT62-2|ATG3_HUMAN	0.01576042	0
sp|P30050|RL12_HUMAN	0.01670456	0.26737198
sp|O43813|LANC1_HUMAN	0.01743889	0
sp|Q9UNH7|SNX6_HUMAN	0.01864815	0
sp|O60271-5|JIP4_HUMAN	0.01882553	0
sp|Q14498-2|RBM39_HUMAN	0.01942635	0.21439649
sp|O75643|U520_HUMAN	0.0198822	0
sp|Q15836|VAMP3_HUMAN	0.02014923	0
sp|Q9BXS5-2|AP1M1_HUMAN	0.02041245	0
sp|Q9HAV0|GBB4_HUMAN	0.02114296	0

sp|P30084|ECHM_HUMAN	0.02119064	0
sp|Q9H0E2|TOLIP_HUMAN	0.02149773	0
sp|P10809|CH60_HUMAN	0.02242279	0
sp|P48681|NEST_HUMAN	0.02249146	0
sp|P02511|CRYAB_HUMAN	0.02252579	0
sp|P21810|PGS1_HUMAN	0.02258492	0.31786543
sp|P50453|SPB9_HUMAN	0.02312088	0
sp|O94905|ERLN2_HUMAN	0.02335548	0
sp|P21283|VATC1_HUMAN	0.02342606	0
sp|Q99426|TBCB_HUMAN	0.02366257	0
sp|Q9HB40|RISC_HUMAN	0.02371597	0
sp|P29218|IMPA1_HUMAN	0.02402496	0.09339783
sp|Q15637-3|SF01_HUMAN	0.02417946	0
sp|P18206-2|VINC_HUMAN	0.02446365	0.01248471
sp|Q14651|PLSI_HUMAN	0.02458	0
sp|P63167|DYL1_HUMAN	0.02480698	0
sp|P51649-2|SSDH_HUMAN	0.02513886	0
sp|O75436|VP26A_HUMAN	0.02536964	0
sp|Q96C19|EFHD2_HUMAN	0.02555084	0
sp|P27361|MK03_HUMAN	0.02566528	0
sp|Q13451|FKBP5_HUMAN	0.02602577	0
sp|P25786-2|PSA1_HUMAN	0.02628326	0.06210651
sp|Q13418|ILK_HUMAN	0.02643395	0
sp|P52209-2|6PGD_HUMAN	0.02648544	0
sp|Q8N8S7-2|ENAH_HUMAN	0.02660561	0
sp|P11216|PYGB_HUMAN	0.02708817	0.1007388
sp|Q9UHX1-2|PUF60_HUMAN	0.02717781	0
sp|Q14847|LASP1_HUMAN	0.02728081	0
sp|P62136|PP1A_HUMAN	0.02768898	0
sp|P38646|GRP75_HUMAN	0.02771378	0
sp|Q9HC35-2|EMAL4_HUMAN	0.02794838	0.07727676
sp|P49354|FNTA_HUMAN	0.02811432	0
sp|P09525|ANXA4_HUMAN	0.02853203	0
sp|O60716-14|CTND1_HUMAN	0.02931595	0
sp|P08571|CD14_HUMAN	0.02953911	0.3005443
sp|Q9UNF0-2|PACN2_HUMAN	0.02977753	0
sp|P51991|ROA3_HUMAN	0.02988625	0
sp|P41250|GARS_HUMAN	0.02997494	0
sp|Q14019|COTL1_HUMAN	0.03052521	0.03367973
sp|Q9NVD7|PARVA_HUMAN	0.03081894	0.08891098
sp|O95202|LETM1_HUMAN	0.03083038	0.45563722
sp|P41240|CSK_HUMAN	0.03131485	0
sp|O75947-2|ATP5H_HUMAN	0.03141403	0
sp|P52788|SPSY_HUMAN	0.03185272	0.7061832
sp|P61457|PHS_HUMAN	0.03261375	0

sp|P05452|TETN_HUMAN	0.03282547	0.09894868
sp|Q9Y315|DEOC_HUMAN	0.03310871	0
sp|Q9NTJ5|SAC1_HUMAN	0.03327942	0
sp|P36776-2|LONM_HUMAN	0.03398514	0
sp|P23786|CPT2_HUMAN	0.0344944	0.02662771
sp|P09543-2|CN37_HUMAN	0.03514099	0
sp|Q10567-2|AP1B1_HUMAN	0.03515816	0.21439649
sp|P11310-2|ACADM_HUMAN	0.03532028	0.14969608
sp|Q5SSJ5-2|HP1B3_HUMAN	0.03573608	0.0846519
sp|P25398|RS12_HUMAN	0.03617477	0
sp|O15145|ARPC3_HUMAN	0.03647995	0
sp|P09917-3|LOX5_HUMAN	0.03720856	0
sp|P62330|ARF6_HUMAN	0.03725815	0
sp|Q86UP2-4|KTN1_HUMAN	0.0373764	0
sp|P02794|FRIH_HUMAN	0.03756332	0
sp|P61160|ARP2_HUMAN	0.03810692	0.01343579
sp|P10515|ODP2_HUMAN	0.03873444	0.29934928
sp|P07237|PDIA1_HUMAN	0.03928375	0.12736098
sp|Q9UKK9|NUDT5_HUMAN	0.03969002	0
sp|P06756-3|ITAV_HUMAN	0.04034042	0.3201336
sp|Q6KB66-2|K2C80_HUMAN	0.04043961	0
sp|P27487|DPP4_HUMAN	0.04086685	0.1558116
sp|Q96CN7|ISOC1_HUMAN	0.04141235	0
sp|P60228|EIF3E_HUMAN	0.04163742	0.30372584
sp|P04899-3|GNAI2_HUMAN	0.04178619	0
sp|Q92696|PGTA_HUMAN	0.04196549	0
sp|P98160|PGBM_HUMAN	0.0420475	0.58386195
sp|O00391|QSOX1_HUMAN	0.04207039	0.17319627
sp|Q9NP72-2|RAB18_HUMAN	0.04227066	0
sp|O75881|CP7B1_HUMAN	0.04227352	0
sp|Q8NBF2-2|NHLC2_HUMAN	0.04236221	0
sp|Q8WZA0-2|LZIC_HUMAN	0.04313564	0
sp|P17980|PRS6A_HUMAN	0.04345322	0
sp|P62191-2|PRS4_HUMAN	0.04355621	0
sp|P12830|CADH1_HUMAN	0.04360199	0
sp|P55072|TERA_HUMAN	0.04423523	0.072567
sp|P31321|KAP1_HUMAN	0.04428101	0
sp|O00231-2|PSD11_HUMAN	0.04453468	0.24097534
sp|P62942|FKB1A_HUMAN	0.04472351	0
sp|P54920|SNAA_HUMAN	0.04498482	0.01551361
sp|P62879|GBB2_HUMAN	0.04508209	0.20467198
sp|P13861|KAP2_HUMAN	0.04535294	0
sp|P39687|AN32A_HUMAN	0.04599381	0
sp|Q9Y2J2-4|E41L3_HUMAN	0.04604149	0
sp|P51178-2|PLCD1_HUMAN	0.04709053	0.09894868

sp|P13693|TCTP_HUMAN	0.04713059	0
sp|Q9P0L0-2|VAPA_HUMAN	0.04745483	0.20467198
sp|Q13505-3|MTX1_HUMAN	0.04804421	0
sp|P53597|SUCA_HUMAN	0.04840851	0
sp|P53992|SC24C_HUMAN	0.04844284	0
sp|Q9Y3F4-2|STRAP_HUMAN	0.04914284	0
sp|O15031|PLXB2_HUMAN	0.04950523	0.35262254
sp|P38919|IF4A3_HUMAN	0.04988289	0.11949348
sp|Q8N1G4|LRC47_HUMAN	0.0504036	0
sp|O75608-2|LYPA1_HUMAN	0.05043411	0
sp|O95573|ACSL3_HUMAN	0.05059433	0
sp|Q04206-3|TF65_HUMAN	0.05144405	0.19149946
sp|Q9BS26|ERP44_HUMAN	0.05167961	0
sp|O14558|HSPB6_HUMAN	0.05219555	0.09750395
sp|Q96HY6|DDRGK_HUMAN	0.05244064	0.09750395
sp|P22392-2|NDKB_HUMAN	0.05315399	0
sp|O94804|STK10_HUMAN	0.05322647	0
sp|Q9NRN5|OLFL3_HUMAN	0.05368614	0
sp|P20618|PSB1_HUMAN	0.0538578	0
sp|P51858|HDGF_HUMAN	0.05406761	0
sp|P46976|GLYG_HUMAN	0.05490494	0
sp|P68104|EF1A1_HUMAN	0.05498886	0.1007388
sp|P63010-2|AP2B1_HUMAN	0.0558815	0.16649151
sp|P01111|RASN_HUMAN	0.0561161	0
sp|P56537|IF6_HUMAN	0.05626106	0.5111962
sp|Q9H4G4|GAPR1_HUMAN	0.05633926	0
sp|Q6UVK1|CSPG4_HUMAN	0.05686188	0.1500082
sp|Q9UH99-2|SUN2_HUMAN	0.05696964	0.14672586
sp|P25705|ATPA_HUMAN	0.05798721	0.00348549
sp|P21281|VATB2_HUMAN	0.05834484	0.31741163
sp|Q13404|UB2V1_HUMAN	0.05894852	0.45033538
sp|P09871|C1S_HUMAN	0.05919647	0.7588735
sp|P61163|ACTZ_HUMAN	0.05922508	0
sp|P19367-3|HXK1_HUMAN	0.0592556	0.02228302
sp|Q9Y394-2|DHRS7_HUMAN	0.05980682	0.06291623
sp|Q07954|LRP1_HUMAN	0.06020355	0.00822741
sp|Q9UMS4|PRP19_HUMAN	0.06027031	0.09894868
sp|Q9NZ01|TECR_HUMAN	0.06038475	0
sp|P36871|PGM1_HUMAN	0.06097794	0
sp|Q9UQ80|PA2G4_HUMAN	0.06113434	0
sp|Q9H0W9-2|CK054_HUMAN	0.06166077	0.10861364
sp|Q96QK1|VPS35_HUMAN	0.06183624	0
sp|Q9Y490|TLN1_HUMAN	0.06209374	0.15866357
sp|P19474|RO52_HUMAN	0.06228447	0
sp|P25789|PSA4_HUMAN	0.06246948	0

sp|O60313-10|OPA1_HUMAN	0.06249523	0
sp|P55854-2|SUMO3_HUMAN	0.06281662	0
sp|P60174|TPIS_HUMAN	0.06317329	0
sp|P04083|ANXA1_HUMAN	0.06331444	0
sp|Q96EP5-2|DAZP1_HUMAN	0.06368446	0
sp|P62316|SMD2_HUMAN	0.0638504	0
sp|P00505|AATM_HUMAN	0.06422043	0.01178462
sp|P46940|IQGA1_HUMAN	0.06481934	0.07054878
sp|P17174|AATC_HUMAN	0.06544876	0
sp|P12109|CO6A1_HUMAN	0.06548691	0.19276452
sp|P62333|PRS10_HUMAN	0.06593704	0
sp|P05166-2|PCCB_HUMAN	0.066082	0.16214149
sp|P35998|PRS7_HUMAN	0.06610298	0
sp|P12081-4|SYHC_HUMAN	0.06632614	0
sp|P22061-2|PIMT_HUMAN	0.06648254	0.34323573
sp|P23142|FBLN1_HUMAN	0.0667038	0
sp|P30086|PEBP1_HUMAN	0.06760597	0
sp|P46108|CRK_HUMAN	0.06768036	0
sp|Q16543|CDC37_HUMAN	0.06779671	0
sp|P61923-5|COPZ1_HUMAN	0.06784821	0
sp|Q8TAT6-2|NPL4_HUMAN	0.06818676	0
sp|O75521-2|ECI2_HUMAN	0.06826401	0
sp|P04632|CPNS1_HUMAN	0.06835365	0.06842031
sp|Q9BR76|COR1B_HUMAN	0.06848145	0
sp|Q8NBJ7|SUMF2_HUMAN	0.06858444	0
sp|O00478-2|BT3A3_HUMAN	0.06892586	0
sp|Q9UKV3-5|ACINU_HUMAN	0.07006455	0.5111962
sp|P01911|2B1F_HUMAN	0.07015991	0
sp|P49591|SYSC_HUMAN	0.07018089	0.14338206
sp|Q9ULA0|DNPEP_HUMAN	0.07032967	0.26737198
sp|P60709|ACTB_HUMAN	0.07057571	0
sp|P63000-2|RAC1_HUMAN	0.07070541	0
sp|Q9BVG4|PBDC1_HUMAN	0.07104492	0.45033538
sp|Q8WYA6-2|CTBL1_HUMAN	0.07161903	0
sp|P53004|BIEA_HUMAN	0.07174492	0
sp|P48643|TCPE_HUMAN	0.07200813	0.08255737
sp|Q07812-2|BAX_HUMAN	0.07214356	0
sp|P55039|DRG2_HUMAN	0.07259178	0
sp|O94911-3|ABCA8_HUMAN	0.07261467	0
sp|P30838|AL3A1_HUMAN	0.072855	0
sp|O00483|NDUA4_HUMAN	0.07288742	0
sp|Q96FW1|OTUB1_HUMAN	0.07293129	0
sp|P25788-2|PSA3_HUMAN	0.07302094	0
sp|Q7Z6Z7-2|HUWE1_HUMAN	0.07323456	0
sp|Q13488|VPP3_HUMAN	0.07357979	0

sp|P46926|GNPI1_HUMAN	0.07376862	0
sp|Q9UJU6-2|DBNL_HUMAN	0.07385635	0
sp|P63104|1433Z_HUMAN	0.07459259	0.07367946
sp|O75439|MPPB_HUMAN	0.07481575	0
sp|P49720|PSB3_HUMAN	0.07499313	0.09339783
sp|P30491|1B53_HUMAN	0.07558823	0
sp|O15372|EIF3H_HUMAN	0.07565498	0
sp|Q9H0D6|XRN2_HUMAN	0.07584381	0
sp|P67936|TPM4_HUMAN	0.07620239	0
sp|Q9NUQ9|FA49B_HUMAN	0.076231	0
sp|P26368-2|U2AF2_HUMAN	0.07719231	0.7061832
sp|Q99623|PHB2_HUMAN	0.07824326	0.35520625
sp|Q9BRA2|TXD17_HUMAN	0.07856178	0
sp|Q8N392|RHG18_HUMAN	0.07903481	0
sp|P61956-2|SUMO2_HUMAN	0.07972908	0
sp|Q9H3H3-1|CK068_HUMAN	0.08019257	0
sp|Q9NZM1-3|MYOF_HUMAN	0.08030701	0
sp|O75369-2|FLNB_HUMAN	0.080616	0.08459944
sp|O60256-3|KPRB_HUMAN	0.0806675	0.06291623
sp|Q9Y6B6|SAR1B_HUMAN	0.08084106	0
sp|P31946|1433B_HUMAN	0.08097267	0
sp|P42025|ACTY_HUMAN	0.08220291	0
sp|Q06136|KDSR_HUMAN	0.08253479	0
sp|Q99460-2|PSMD1_HUMAN	0.08286476	0
sp|P17655|CAN2_HUMAN	0.08290672	0.3425803
sp|Q9BSJ8-2|ESYT1_HUMAN	0.08322525	0.07841589
sp|P54802|ANAG_HUMAN	0.08432007	0
sp|Q9NR19-2|ACSA_HUMAN	0.08446121	0
sp|Q9TQE0|2B19_HUMAN	0.08470917	0
sp|P01833|PIGR_HUMAN	0.08501816	0.7118947
sp|E9PAV3|NACAM_HUMAN	0.08581924	0
sp|Q9UI12-2|VATH_HUMAN	0.08588409	0
sp|Q7Z7H5-3|TMED4_HUMAN	0.08642197	0
sp|Q08380|LG3BP_HUMAN	0.08642387	0.9968222
sp|Q5EBM0-3|CMPK2_HUMAN	0.08713341	0.09894868
sp|P08648|ITA5_HUMAN	0.08716202	0
sp|Q6PCB0|VWA1_HUMAN	0.08772659	0
sp|Q9NRV9|HEBP1_HUMAN	0.08802223	0.21439649
sp|O14672|ADA10_HUMAN	0.0883522	0
sp|Q00765|REEP5_HUMAN	0.08835793	0
sp|P08134|RHOC_HUMAN	0.08866882	0
sp|P20700|LMNB1_HUMAN	0.08867455	0.23855431
sp|P63096|GNAI1_HUMAN	0.08873749	0
sp|P25325-2|THTM_HUMAN	0.08904266	0.09750395
sp|Q86WV6|STING_HUMAN	0.08918572	0

sp|Q9Y6E0|STK24_HUMAN	0.0892334	0
sp|Q96IU4|ABHEB_HUMAN	0.08974838	0
sp|P61970|NTF2_HUMAN	0.09016991	0
sp|Q8NHV1|GIMA7_HUMAN	0.09054756	0.2178309
sp|P16152|CBR1_HUMAN	0.09055901	0
sp|O00429-3|DNM1L_HUMAN	0.09119415	0
sp|Q96BW5-2|PTER_HUMAN	0.0912075	0
sp|O43148-2|MCES_HUMAN	0.09197617	0
sp|Q96HC4|PDLI5_HUMAN	0.0922966	0.23973103
sp|P52790|HXK3_HUMAN	0.0926342	0
sp|Q96BM9|ARL8A_HUMAN	0.09316063	0
sp|Q9BRF8|CPPED_HUMAN	0.09344864	0
sp|P17844-2|DDX5_HUMAN	0.09367561	0
sp|Q16527|CSRP2_HUMAN	0.09371567	0
sp|P55795|HNRH2_HUMAN	0.0942955	0
sp|P49903-2|SPS1_HUMAN	0.09481049	0
sp|P07741|APT_HUMAN	0.09528923	0
sp|O60784|TOM1_HUMAN	0.09541512	0
sp|P21291|CSRP1_HUMAN	0.09549713	0.0315703
sp|O75083|WDR1_HUMAN	0.09617996	0
sp|P55263|ADK_HUMAN	0.09657288	0
sp|Q5JRX3-2|PREP_HUMAN	0.09781075	0
sp|Q9BUT1|BDH2_HUMAN	0.09787369	0
sp|P14868|SYDC_HUMAN	0.09796715	0.01551361
sp|O75937|DNJC8_HUMAN	0.0989666	0.30372584
sp|Q03154-4|ACY1_HUMAN	0.09907532	0.23541966
sp|Q8NBX0|SCPDL_HUMAN	0.09908104	0
sp|P18669|PGAM1_HUMAN	0.10009193	0
sp|P13073|COX41_HUMAN	0.10057449	0
sp|P54652|HSP72_HUMAN	0.10092545	0
sp|O60506-3|HNRPQ_HUMAN	0.1015892	0.02662771
sp|Q9UGI8-2|TES_HUMAN	0.10428906	0.02174174
sp|P55327-3|TPD52_HUMAN	0.10555649	0.2178309
sp|O95197-2|RTN3_HUMAN	0.10565186	0
sp|P06748-3|NPM_HUMAN	0.10582733	0
sp|P30533|AMRP_HUMAN	0.10610771	0
sp|P55084|ECHB_HUMAN	0.10658073	0.11920157
sp|Q5T440|CAF17_HUMAN	0.10663605	0
sp|P13489|RINI_HUMAN	0.10691452	0.1069263
sp|Q13148|TADBP_HUMAN	0.10728836	0
sp|Q9BPW8|NIPS1_HUMAN	0.10748482	0
sp|Q96HE7|ERO1A_HUMAN	0.1076088	0
sp|P07195|LDHB_HUMAN	0.10762405	0.12561582
sp|O94919|ENDD1_HUMAN	0.10768318	0.06291623
sp|Q92820|GGH_HUMAN	0.10769844	0.19149946

sp|P60842|IF4A1_HUMAN	0.10802651	0.01178462
sp|Q13724-2|MOGS_HUMAN	0.10865212	0.03043297
sp|P55209-2|NP1L1_HUMAN	0.1091919	0
sp|P17301|ITA2_HUMAN	0.10932732	0
sp|P51692|STA5B_HUMAN	0.10950661	0
sp|Q9Y285|SYFA_HUMAN	0.10950947	0
sp|P07203|GPX1_HUMAN	0.10984421	0
sp|P27694|RFA1_HUMAN	0.11042786	0.20467198
sp|O15254-2|ACOX3_HUMAN	0.11067009	0
sp|Q99961-2|SH3G1_HUMAN	0.11090469	0
sp|Q01130-2|SRSF2_HUMAN	0.11103058	0.2178309
sp|Q14139-2|UBE4A_HUMAN	0.1117096	0
sp|Q92947|GCDH_HUMAN	0.11206818	0
sp|O00116|ADAS_HUMAN	0.11249733	0
sp|P31942-2|HNRH3_HUMAN	0.11266518	0.7498006
sp|Q16576-2|RBBP7_HUMAN	0.11292267	0
sp|P34897-3|GLYM_HUMAN	0.11415291	0
sp|P02743|SAMP_HUMAN	0.11421204	0
sp|P50570-5|DYN2_HUMAN	0.11500931	0.14927356
sp|O95861-4|BPNT1_HUMAN	0.11545181	0.03367973
sp|Q14203-4|DCTN1_HUMAN	0.11605454	0.12054814
sp|P21980|TGM2_HUMAN	0.11677933	0.12200338
sp|P00918|CAH2_HUMAN	0.1169529	0.13973783
sp|P09493-5|TPM1_HUMAN	0.11726761	0.19149946
sp|O75475|PSIP1_HUMAN	0.11728859	0
sp|O75396|SC22B_HUMAN	0.11754799	0
sp|P08621-2|RU17_HUMAN	0.11837006	0.04454162
sp|P12955|PEPD_HUMAN	0.1183815	0
sp|Q9UNZ2-5|NSF1C_HUMAN	0.11868858	0
sp|P52907|CAZA1_HUMAN	0.11879921	0.47871065
sp|Q96RU3-3|FNBP1_HUMAN	0.11950207	0
sp|Q15691|MARE1_HUMAN	0.11964798	0
sp|Q99714|HCD2_HUMAN	0.1196785	0.16702904
sp|Q9Y224|RTRAF_HUMAN	0.11976051	0.48520416
sp|P49755|TMEDA_HUMAN	0.11987495	0
sp|Q9Y2Q3-3|GSTK1_HUMAN	0.11991692	0
sp|P35612-2|ADDB_HUMAN	0.12085724	0
sp|Q9UJW0-3|DCTN4_HUMAN	0.12145424	0
sp|Q9Y277-2|VDAC3_HUMAN	0.12161255	0.09339783
sp|Q06323|PSME1_HUMAN	0.12192917	0.3005443
sp|O14787-2|TNPO2_HUMAN	0.12201309	0
sp|P84090|ERH_HUMAN	0.12206459	0
sp|Q92900-2|RENT1_HUMAN	0.12231445	0
sp|Q13838-2|DX39B_HUMAN	0.12234116	0
sp|O14964-2|HGS_HUMAN	0.12244034	0

sp|O43776|SYNC_HUMAN	0.12399864	0
sp|P50395|GDIB_HUMAN	0.12454796	0.11403357
sp|P35611-2|ADDA_HUMAN	0.12461281	0.02662771
sp|Q9UBB4-2|ATX10_HUMAN	0.12462616	0
sp|Q14764|MVP_HUMAN	0.12462807	0.1746542
sp|P62906|RL10A_HUMAN	0.12513542	0.03367973
sp|Q9NSD9|SYFB_HUMAN	0.12550354	0
sp|Q08257|QOR_HUMAN	0.12612152	0.12054814
sp|Q92888-2|ARHG1_HUMAN	0.1264019	0.35795313
sp|P39019|RS19_HUMAN	0.12644577	0.2178309
sp|Q7L1Q6-2|BZW1_HUMAN	0.126791	0
sp|Q15149-9|PLEC_HUMAN	0.12710953	0
sp|P04406|G3P_HUMAN	0.12740326	0.00508175
sp|P22102|PUR2_HUMAN	0.12744522	0.30372584
sp|Q709C8-2|VP13C_HUMAN	0.12767315	0
sp|Q14204|DYHC1_HUMAN	0.12778854	0.21807808
sp|Q6XQN6|PNCB_HUMAN	0.12788773	0.17077933
sp|Q9H9B4|SFXN1_HUMAN	0.12808228	0
sp|P00491|PNPH_HUMAN	0.12810516	0.17511293
sp|P50452|SPB8_HUMAN	0.12842941	0
sp|P14618-3|KPYM_HUMAN	0.1285286	0
sp|Q14240-2|IF4A2_HUMAN	0.12876129	0.40256184
sp|P43243|MATR3_HUMAN	0.12906456	0.37060758
sp|P48735|IDHP_HUMAN	0.130167	0.01551361
sp|P48147|PPCE_HUMAN	0.13035583	0
sp|P12110|CO6A2_HUMAN	0.1312561	0.64348376
sp|Q9H9G7-2|AGO3_HUMAN	0.1313324	0.45033538
sp|P23284|PPIB_HUMAN	0.13142395	0.6255959
sp|P62495-2|ERF1_HUMAN	0.13161278	0
sp|Q15019-2|SEPT2_HUMAN	0.13185501	0.0315703
sp|Q9HD89|RETN_HUMAN	0.13196373	0.19149946
sp|P55786|PSA_HUMAN	0.13228226	0.04950076
sp|Q96RQ3|MCCA_HUMAN	0.13260269	0
sp|Q9Y6W5|WASF2_HUMAN	0.1327591	0.09339783
sp|Q5K4L6-2|S27A3_HUMAN	0.1336937	0
sp|Q9BT78|CSN4_HUMAN	0.13414001	0
sp|Q06787-10|FMR1_HUMAN	0.13447762	0
sp|Q15102|PA1B3_HUMAN	0.13451767	0.45563722
sp|Q8TBC4|UBA3_HUMAN	0.13464165	0
sp|Q9H3N1|TMX1_HUMAN	0.13529205	0.09894868
sp|P53602|MVD1_HUMAN	0.13576698	0
sp|Q13045-3|FLII_HUMAN	0.13578033	0
sp|O60610-2|DIAP1_HUMAN	0.13582802	0.29066643
sp|P26196|DDX6_HUMAN	0.13583755	0
sp|Q9Y6C2|EMIL1_HUMAN	0.13645744	0.54687065

sp|Q8NHP8|PLBL2_HUMAN	0.13647842	0
sp|P10644|KAP0_HUMAN	0.1366005	0.09339783
sp|P10768|ESTD_HUMAN	0.13720894	0
sp|Q08379|GOGA2_HUMAN	0.13800812	0
sp|Q96AE4-2|FUBP1_HUMAN	0.1387024	0.5032694
sp|P04275|VWF_HUMAN	0.13884735	0.02921007
sp|P63027|VAMP2_HUMAN	0.1397152	0
sp|P30153|2AAA_HUMAN	0.13983154	0.02333495
sp|P00338|LDHA_HUMAN	0.13991928	0
sp|P19971|TYPH_HUMAN	0.13994217	1.1323998
sp|P43307|SSRA_HUMAN	0.1401329	0.7827403
sp|Q8WXF1|PSPC1_HUMAN	0.14052963	0
sp|O00233-2|PSMD9_HUMAN	0.14076805	0
sp|P53041|PPP5_HUMAN	0.14076996	0
sp|O43837|IDH3B_HUMAN	0.14077759	0.2178309
sp|P13716-2|HEM2_HUMAN	0.14160919	0.02662771
sp|Q9HCB6|SPON1_HUMAN	0.1418953	0.04687595
sp|P02549-2|SPTA1_HUMAN	0.14203072	1.2435468
sp|O15173-2|PGRC2_HUMAN	0.14232826	0.09750395
sp|Q9Y2A7-2|NCKP1_HUMAN	0.14236069	0
sp|Q9Y4G6|TLN2_HUMAN	0.14330578	0.19149946
sp|Q13409-2|DC1I2_HUMAN	0.14404869	0.03367973
sp|Q8NBJ5|GT251_HUMAN	0.1442833	0.28516325
sp|P09619|PGFRB_HUMAN	0.14434814	0.1342476
sp|P40306|PSB10_HUMAN	0.14477158	0.5204253
sp|Q93052|LPP_HUMAN	0.14510536	0.29301754
sp|P43686|PRS6B_HUMAN	0.14520264	0
sp|O15382|BCAT2_HUMAN	0.14529991	0.30372584
sp|P11766|ADHX_HUMAN	0.14594269	0.25398457
sp|P12268|IMDH2_HUMAN	0.14605713	0.26737198
sp|O75874|IDHC_HUMAN	0.1462059	0.16293353
sp|P47756-2|CAPZB_HUMAN	0.14623833	0.31786543
sp|P62195|PRS8_HUMAN	0.14627266	0
sp|Q13098-5|CSN1_HUMAN	0.14645004	0
sp|O94826|TOM70_HUMAN	0.14679718	0.11949348
sp|P48444|COPD_HUMAN	0.14711761	0.22784016
sp|Q9UEY8|ADDG_HUMAN	0.14720154	0.0846519
sp|Q8WUM4|PDC6I_HUMAN	0.14736557	0.07588609
sp|P11586|C1TC_HUMAN	0.14741898	0.02662771
sp|P11166|GTR1_HUMAN	0.14767838	0
sp|P40261|NNMT_HUMAN	0.14795685	0.19149946
sp|P10155-3|RO60_HUMAN	0.1481018	0.633381
sp|Q16851-2|UGPA_HUMAN	0.14813614	0
sp|Q12905|ILF2_HUMAN	0.14822578	0.11920157
sp|P31948|STIP1_HUMAN	0.14896965	0.13745423

sp|Q9P2R7-2|SUCB1_HUMAN	0.14929199	0.26546443
sp|Q92890-1|UFD1_HUMAN	0.14958191	0.19149946
sp|P24666-2|PPAC_HUMAN	0.14968014	0.09894868
sp|P48163|MAOX_HUMAN	0.14998817	0.19410844
sp|P28161|GSTM2_HUMAN	0.15043068	0.7827403
sp|Q8IUX7|AEBP1_HUMAN	0.15058136	0
sp|P22897|MRC1_HUMAN	0.15070724	0.91458976
sp|P27348|1433T_HUMAN	0.15093994	0
sp|P61081|UBC12_HUMAN	0.15109825	0
sp|P31943|HNRH1_HUMAN	0.15121078	0
sp|P24534|EF1B_HUMAN	0.15185356	0.35795313
sp|P17987|TCPA_HUMAN	0.15195465	0.05473607
sp|P22059|OSBP1_HUMAN	0.15225983	0
sp|Q9UBG0|MRC2_HUMAN	0.15250015	0.2591514
sp|Q15233|NONO_HUMAN	0.15251541	0.29301754
sp|Q96CW1-2|AP2M1_HUMAN	0.15259743	0
sp|Q9NY33|DPP3_HUMAN	0.15279388	0.3188726
sp|P11177-3|ODPB_HUMAN	0.15342712	0
sp|P04424-2|ARLY_HUMAN	0.15388107	0.09339783
sp|P49458|SRP09_HUMAN	0.1539917	0
sp|P50990|TCPQ_HUMAN	0.15492249	0.2524271
sp|P55083-2|MFAP4_HUMAN	0.15528679	0.19149946
sp|Q99497|PARK7_HUMAN	0.15587616	0.14672586
sp|Q96M27-3|PRRC1_HUMAN	0.15600586	0.19149946
sp|O00299|CLIC1_HUMAN	0.15637779	0.01700861
sp|O14818|PSA7_HUMAN	0.15765762	0
sp|P29590|PML_HUMAN	0.15786934	0.051214
sp|P24557-2|THAS_HUMAN	0.15789413	0
sp|Q96A33-2|CCD47_HUMAN	0.1581707	0
sp|P08559-2|ODPA_HUMAN	0.15849113	0
sp|Q6DD88|ATLA3_HUMAN	0.1595459	0
sp|Q86VS8|HOOK3_HUMAN	0.15970421	0
sp|Q9NVA2|SEP11_HUMAN	0.1598053	0.1791607
sp|Q9BUQ8|DDX23_HUMAN	0.15992355	0
sp|P23229-4|ITA6_HUMAN	0.15995598	0
sp|Q07955-2|SRSF1_HUMAN	0.16004562	1.1932943
sp|P49961|ENTP1_HUMAN	0.16033936	0
sp|P05783|K1C18_HUMAN	0.16143608	0.23826522
sp|Q14152-2|EIF3A_HUMAN	0.16150475	0.26546443
sp|Q92945|FUBP2_HUMAN	0.16151619	0.63371044
sp|P41091|IF2G_HUMAN	0.16210938	0
sp|Q9Y2B0|CNPY2_HUMAN	0.1631813	0
sp|Q16181-2|SEPT7_HUMAN	0.16361237	0.01700861
sp|P31930|QCR1_HUMAN	0.16381836	0.10138063
sp|O95336|6PGL_HUMAN	0.16398239	0.14927356

sp|Q71U36-2|TBA1A_HUMAN	0.16408348	0
sp|P09496-2|CLCA_HUMAN	0.1643467	0
sp|O43747-2|AP1G1_HUMAN	0.16435242	0
sp|Q2TAY7|SMU1_HUMAN	0.1644764	0
sp|Q9BZZ5-3|API5_HUMAN	0.1645298	0.23541966
sp|P30520|PURA2_HUMAN	0.16458893	0
sp|Q9NZU5|LMCD1_HUMAN	0.16489029	0.22032139
sp|O60884|DNJA2_HUMAN	0.16568375	0
sp|O14974-3|MYPT1_HUMAN	0.16607857	0.34323573
sp|P40227|TCPZ_HUMAN	0.16646957	0.3563347
sp|Q8TDZ2-4|MICA1_HUMAN	0.16662979	0
sp|P00558|PGK1_HUMAN	0.16675949	0.10770471
sp|P33176|KINH_HUMAN	0.16731834	0.79939914
sp|P53396-2|ACLY_HUMAN	0.1677227	0.00764478
sp|P35270|SPRE_HUMAN	0.16773129	0.30372584
sp|P55735|SEC13_HUMAN	0.16778374	0.35795313
sp|Q08211|DHX9_HUMAN	0.16790771	0.46284658
sp|P52272-2|HNRPM_HUMAN	0.16797829	0.52929556
sp|P22033|MUTA_HUMAN	0.16858482	0
sp|O60879-2|DIAP2_HUMAN	0.16873169	0
sp|P18085|ARF4_HUMAN	0.16873169	0
sp|P29350-4|PTN6_HUMAN	0.16886139	0.37061578
sp|P29466-2|CASP1_HUMAN	0.16899681	0.5204253
sp|Q15029-2|U5S1_HUMAN	0.16939926	0.4584066
sp|O00487|PSDE_HUMAN	0.16942406	0
sp|Q16401-2|PSMD5_HUMAN	0.16947937	0
sp|O43681|ASNA_HUMAN	0.17002869	0
sp|P05387|RLA2_HUMAN	0.17012215	0
sp|P20073-2|ANXA7_HUMAN	0.17082977	0.76289684
sp|O14617-4|AP3D1_HUMAN	0.1708641	0
sp|P49902-2|5NTC_HUMAN	0.17142487	0
sp|P08294|SODE_HUMAN	0.1721878	0
sp|Q68EM7-2|RHG17_HUMAN	0.17220592	0.19149946
sp|P50454|SERPH_HUMAN	0.17228317	0.02662771
sp|P21333-2|FLNA_HUMAN	0.17266464	0.53565615
sp|P62750|RL23A_HUMAN	0.17352295	0
sp|Q9Y2S2|CRYL1_HUMAN	0.17484283	0.30372584
sp|P00746|CFAD_HUMAN	0.17489052	0
sp|P54725-2|RD23A_HUMAN	0.17518234	0.45033538
sp|Q96I15|SCLY_HUMAN	0.17591286	0.45033538
sp|P11940-2|PABP1_HUMAN	0.17591667	0
sp|P09417|DHPR_HUMAN	0.17593002	0
sp|O43294|TGFI1_HUMAN	0.17602539	0
sp|Q9Y3A5|SBDS_HUMAN	0.17613411	0.06291623
sp|Q13547|HDAC1_HUMAN	0.17650223	0

sp|Q15257-2|PTPA_HUMAN	0.17729378	0
sp|P11217-2|PYGM_HUMAN	0.177845	0
sp|P32456|GBP2_HUMAN	0.17787361	0
sp|Q9UL46|PSME2_HUMAN	0.17835426	0.68888646
sp|P08133|ANXA6_HUMAN	0.17867279	0.07278968
sp|P00352|AL1A1_HUMAN	0.1786747	0.00443329
sp|O94811|TPPP_HUMAN	0.17873764	0
sp|Q9NQR4|NIT2_HUMAN	0.17885971	0
sp|Q9Y2X3|NOP58_HUMAN	0.17908859	0.45033538
sp|Q7Z3D6-3|GLUCM_HUMAN	0.17970276	0
sp|P14317|HCLS1_HUMAN	0.17977142	0
sp|Q12931-2|TRAP1_HUMAN	0.17979813	0
sp|Q8WUP2-3|FBLI1_HUMAN	0.18039703	0
sp|P68366-2|TBA4A_HUMAN	0.18054199	0
sp|Q9P2X0-2|DPM3_HUMAN	0.18130302	0
sp|Q9UKG1|DP13A_HUMAN	0.18146706	0.28516325
sp|Q9P2T1-2|GMPR2_HUMAN	0.18171501	0.09894868
sp|Q14914-2|PTGR1_HUMAN	0.18198395	0
sp|Q08945|SSRP1_HUMAN	0.18230629	0.84879977
sp|Q969X5|ERGI1_HUMAN	0.18241882	0
sp|P23526|SAHH_HUMAN	0.18295097	0.00934436
sp|O94973-2|AP2A2_HUMAN	0.18310928	0.53506804
sp|Q05655-2|KPCD_HUMAN	0.18321228	0.28516325
sp|P33316|DUT_HUMAN	0.18376923	0.19149946
sp|P21912|SDHB_HUMAN	0.18379593	0
sp|P04075|ALDOA_HUMAN	0.18391418	0.31106147
sp|Q13557-10|KCC2D_HUMAN	0.18395805	0.3630891
sp|P11171-2|41_HUMAN	0.1844368	0
sp|P26641|EF1G_HUMAN	0.18451881	0.2824735
sp|P15121|ALDR_HUMAN	0.1846199	0
sp|P07384|CAN1_HUMAN	0.18479347	0.02261831
sp|P30101|PDIA3_HUMAN	0.18501282	0.6730828
sp|P27797|CALR_HUMAN	0.18621445	0.8244277
sp|Q96P70|IPO9_HUMAN	0.18668556	0.19149946
sp|P30044|PRDX5_HUMAN	0.18727875	0.09476809
sp|Q13425|SNTB2_HUMAN	0.18793488	0
sp|P34896-2|GLYC_HUMAN	0.18807602	0.21439649
sp|O00303|EIF3F_HUMAN	0.18844414	0.35193655
sp|P30626-2|SORCN_HUMAN	0.18855286	0
sp|O75340-2|PDCD6_HUMAN	0.1885624	0
sp|Q06124-2|PTN11_HUMAN	0.18870735	0
sp|P55265-5|DSRAD_HUMAN	0.18986893	0.45033538
sp|P40939|ECHA_HUMAN	0.19029427	0.33566394
sp|Q7L2H7|EIF3M_HUMAN	0.1903801	0
sp|Q13243-3|SRSF5_HUMAN	0.191082	0.19149946

sp|P40925-2|MDHC_HUMAN	0.19128609	0.06210651
sp|Q9BUJ2-4|HNRL1_HUMAN	0.19139099	0.24097534
sp|P51911|CNN1_HUMAN	0.1913948	0.22362706
sp|Q16531|DDB1_HUMAN	0.1919117	0.2711284
sp|Q02790|FKBP4_HUMAN	0.19206238	0.09339783
sp|P13798|ACPH_HUMAN	0.1920929	0.3188726
sp|P22234-2|PUR6_HUMAN	0.19211578	0
sp|P30419-2|NMT1_HUMAN	0.19263458	0
sp|Q6NVY1|HIBCH_HUMAN	0.19263458	0.1342476
sp|O43390-2|HNRPR_HUMAN	0.1926403	0.21133235
sp|Q9NUV9|GIMA4_HUMAN	0.19271088	0
sp|P12270|TPR_HUMAN	0.19298172	0.01819076
sp|P52597|HNRPF_HUMAN	0.19428253	0.20467198
sp|P42226|STAT6_HUMAN	0.19448662	0
sp|Q15393|SF3B3_HUMAN	0.19503975	0.19484499
sp|P62304|RUXE_HUMAN	0.1950512	0
sp|Q16539-2|MK14_HUMAN	0.19543457	0.28516325
sp|P30566|PUR8_HUMAN	0.19545174	0.2178309
sp|P05388-2|RLA0_HUMAN	0.1964798	0.14927356
sp|P29692|EF1D_HUMAN	0.19672966	0
sp|P31153|METK2_HUMAN	0.19674683	0
sp|P67775-2|PP2AA_HUMAN	0.19763374	0
sp|Q1KMD3|HNRL2_HUMAN	0.19768906	0.23542999
sp|P27824-2|CALX_HUMAN	0.19798279	0.62912476
sp|Q13976|KGP1_HUMAN	0.19871521	0.06986027
sp|P48059-3|LIMS1_HUMAN	0.19976044	0.1342476
sp|Q15075|EEA1_HUMAN	0.19983101	0.6287559
sp|P05455|LA_HUMAN	0.20001984	0.41804093
sp|Q15642-3|CIP4_HUMAN	0.20054436	0
sp|P09104-2|ENOG_HUMAN	0.2006836	0
sp|P51571|SSRD_HUMAN	0.20080948	0.09894868
sp|Q96KP4|CNDP2_HUMAN	0.20158195	0.32334426
sp|O15260-2|SURF4_HUMAN	0.20162201	0.19149946
sp|P30085|KCY_HUMAN	0.20166779	0.09894868
sp|Q13263|TIF1B_HUMAN	0.20228958	0.4490988
sp|Q15717|ELAV1_HUMAN	0.20274925	0.28516325
sp|P02795|MT2_HUMAN	0.20489883	0.45033538
sp|O75688|PPM1B_HUMAN	0.2051239	0
sp|P50991|TCPD_HUMAN	0.205904	0
sp|O43242|PSMD3_HUMAN	0.20652199	0.16702904
sp|Q9NRX4|PHP14_HUMAN	0.20680618	0.28516325
sp|Q9NQG5|RPR1B_HUMAN	0.20775318	0.35795313
sp|Q7Z4H8|PLGT3_HUMAN	0.20808792	0.30372584
sp|O60749-2|SNX2_HUMAN	0.20819473	0.10943281
sp|P18077|RL35A_HUMAN	0.20835686	0.45033538

sp|P45974-2|UBP5_HUMAN	0.2086525	0.45546502
sp|Q10713-2|MPPA_HUMAN	0.20888329	0
sp|Q8NFW8|NEUA_HUMAN	0.20949936	0
sp|Q9H3U1-2|UN45A_HUMAN	0.2098217	0
sp|P54727|RD23B_HUMAN	0.21026802	0.30372584
sp|O60701|UGDH_HUMAN	0.21157646	0.42759705
sp|Q9P258|RCC2_HUMAN	0.21190262	0.26737198
sp|P62140|PP1B_HUMAN	0.21201324	0
sp|Q9NSE4|SYIM_HUMAN	0.2128315	0.38551986
sp|Q9NZ08-2|ERAP1_HUMAN	0.21288872	0.3852666
sp|P14618-2|KPYM_HUMAN	0.21300888	0.34323573
sp|Q9H4A4|AMPB_HUMAN	0.21303558	0.12561582
sp|Q92734-2|TFG_HUMAN	0.21315765	0
sp|Q9BY32|ITPA_HUMAN	0.2133131	0.69308156
sp|O43143|DHX15_HUMAN	0.21375656	0.2207585
sp|P62269|RS18_HUMAN	0.21443176	1.1276597
sp|P50914|RL14_HUMAN	0.21491623	0
sp|Q13618-2|CUL3_HUMAN	0.2151928	0.26546443
sp|P63151-2|2ABA_HUMAN	0.21600533	0
sp|O95782-2|AP2A1_HUMAN	0.21647263	0.7022581
sp|O75964|ATP5L_HUMAN	0.21719933	0.19149946
sp|P50579-2|MAP2_HUMAN	0.21724701	0.35795313
sp|P13639|EF2_HUMAN	0.21741104	0.8487422
sp|P49065|ALBU_RABIT	0.21774101	0.656254
sp|Q9H8H3|MET7A_HUMAN	0.21783638	0
sp|P12277|KCRB_HUMAN	0.21800613	0.41804093
sp|P12931-2|SRC_HUMAN	0.21819305	0
sp|Q00013-2|EM55_HUMAN	0.21881676	0
sp|Q5JTV8-3|TOIP1_HUMAN	0.21926498	0.06291623
sp|Q14558-2|KPRA_HUMAN	0.21953964	0.656254
sp|P17858-2|PFKAL_HUMAN	0.219553	0.08307569
sp|Q9UNM6-2|PSD13_HUMAN	0.21985245	0.04454162
sp|O75534-2|CSDE1_HUMAN	0.22018623	0.28516325
sp|Q8TD19|NEK9_HUMAN	0.22083664	0.19149946
sp|B5ME19|EIFCL_HUMAN	0.22174263	0.29934928
sp|Q92747|ARC1A_HUMAN	0.22278404	0
sp|P12956|XRCC6_HUMAN	0.222826	0.22948365
sp|Q8N163-2|CCAR2_HUMAN	0.22332	0.06291623
sp|P00736|C1R_HUMAN	0.2236824	0.19149946
sp|O60664-4|PLIN3_HUMAN	0.22424126	0.10861364
sp|P04350|TBB4A_HUMAN	0.2245121	0
sp|Q99873-2|ANM1_HUMAN	0.22496223	0.30372584
sp|Q9UH65|SWP70_HUMAN	0.22536659	0
sp|P09972|ALDOC_HUMAN	0.2254467	0.3630891
sp|Q9UQ16-2|DYN3_HUMAN	0.22607136	0

sp|Q9BVC6|TM109_HUMAN	0.22655487	0.35795313
sp|P69891|HBG1_HUMAN	0.22709656	1.0634323
sp|P47897|SYQ_HUMAN	0.22750664	0
sp|Q92688-2|AN32B_HUMAN	0.22795677	0
sp|P55809|SCOT1_HUMAN	0.22814369	0
sp|P27695|APEX1_HUMAN	0.22825241	0.30372584
sp|P48960-2|CD97_HUMAN	0.22896004	0
sp|Q93009-3|UBP7_HUMAN	0.22908592	0.04454162
sp|Q86VP6|CAND1_HUMAN	0.22944832	0.5204253
sp|P01009|A1AT_HUMAN	0.2296009	0.6948127
sp|O00571-2|DDX3X_HUMAN	0.22968292	0
sp|Q8WVM8|SCFD1_HUMAN	0.23050499	0.21439649
sp|P24821-4|TENA_HUMAN	0.23088837	0.3985966
sp|Q12797-10|ASPH_HUMAN	0.23137093	0.23541966
sp|P31040|SDHA_HUMAN	0.23169327	0.5004855
sp|P0CG39|POTEJ_HUMAN	0.23171997	0
sp|P21399|ACOC_HUMAN	0.23277283	0.26737198
sp|O75746-2|CMC1_HUMAN	0.23308563	0.5111962
sp|O14828-2|SCAM3_HUMAN	0.23425293	0
sp|P53990-2|IST1_HUMAN	0.23501778	0
sp|Q9Y3I0|RTCB_HUMAN	0.23587036	0.42646354
sp|Q12805-2|FBLN3_HUMAN	0.23622894	1.2773042
sp|P53618|COPB_HUMAN	0.23713684	0.5834694
sp|O00410|IPO5_HUMAN	0.23731613	0.7957244
sp|P50995-2|ANX11_HUMAN	0.23804855	0.3761346
sp|P62277|RS13_HUMAN	0.23821831	0
sp|P78371|TCPB_HUMAN	0.23830986	0.4053777
sp|P32455|GBP1_HUMAN	0.23971176	0.1897449
sp|P11142|HSP7C_HUMAN	0.23977661	0.25004795
sp|Q92552-2|RT27_HUMAN	0.2398901	0.45033538
sp|Q9BTV4|TMM43_HUMAN	0.24052238	0.84879977
sp|P49721|PSB2_HUMAN	0.24056435	0.656254
sp|Q9Y678|COPG1_HUMAN	0.24083138	0.53821
sp|P62736|ACTA_HUMAN	0.24247551	0.2823809
sp|P13646-3|K1C13_HUMAN	0.24306488	0.44572112
sp|P49189|AL9A1_HUMAN	0.24346352	0.00764478
sp|Q6P2Q9|PRP8_HUMAN	0.24346542	0.316588
sp|Q96S97|MYADM_HUMAN	0.24379349	0
sp|P08754|GNAI3_HUMAN	0.24407196	0
sp|P13010|XRCC5_HUMAN	0.24528503	0.47446895
sp|Q9H2G2-2|SLK_HUMAN	0.24547005	0
sp|P31150|GDIA_HUMAN	0.24604797	0.01551361
sp|P28062-2|PSB8_HUMAN	0.24656868	0.30372584
sp|P08708|RS17_HUMAN	0.24667168	0.06291623
sp|P58107|EPIPL_HUMAN	0.2474289	0.7622591

sp|P35637-2|FUS_HUMAN	0.24816895	0.45033538
sp|Q13200|PSMD2_HUMAN	0.2481842	0
sp|P00441|SODC_HUMAN	0.24861526	0.20467198
sp|P63244|RACK1_HUMAN	0.2487297	0
sp|Q02952-2|AKA12_HUMAN	0.24904251	0.19149946
sp|Q8IUD2-2|RB6I2_HUMAN	0.2493515	0
sp|Q07960|RHG01_HUMAN	0.24938583	0.47871065
sp|Q16891-2|MIC60_HUMAN	0.24964142	0.3005443
sp|Q92506|DHB8_HUMAN	0.2500782	1.1932944
sp|Q02543|RL18A_HUMAN	0.25014877	1.2392054
sp|O76003|GLRX3_HUMAN	0.25028038	0
sp|Q9Y570-2|PPME1_HUMAN	0.2505169	0.6070219
sp|O60832-2|DKC1_HUMAN	0.25065994	0.09894868
sp|Q02878|RL6_HUMAN	0.25127792	0.7827403
sp|P35573|GDE_HUMAN	0.25155067	0.7061832
sp|P27338|AOFB_HUMAN	0.2517109	0.2453838
sp|P31939|PUR9_HUMAN	0.25193024	0.57855076
sp|P62917|RL8_HUMAN	0.25195312	0.70235044
sp|Q9Y262|EIF3L_HUMAN	0.25200653	0.08779849
sp|O94979-10|SC31A_HUMAN	0.25299835	0
sp|P29144|TPP2_HUMAN	0.25335503	0.42759705
sp|Q92841-1|DDX17_HUMAN	0.2537117	0.14388667
sp|P49915-2|GUAA_HUMAN	0.2540512	0
sp|O94776|MTA2_HUMAN	0.2542572	0
sp|Q14258|TRI25_HUMAN	0.25450134	0.6634523
sp|P02751-15|FINC_HUMAN	0.25452614	0
sp|P08865|RSSA_HUMAN	0.25471497	0.15980355
sp|Q14108-2|SCRB2_HUMAN	0.25575256	0.45033538
sp|Q8WXF7-2|ATLA1_HUMAN	0.25647736	0
sp|P52306|GDS1_HUMAN	0.25827217	0
sp|P31689|DNJA1_HUMAN	0.25894928	0.5204253
sp|P49588-2|SYAC_HUMAN	0.25924683	1.1593928
sp|P49411|EFTU_HUMAN	0.25951385	0.91601294
sp|Q15293|RCN1_HUMAN	0.26233864	0
sp|P35268|RL22_HUMAN	0.2625122	0.21439649
sp|P04004|VTNC_HUMAN	0.2630558	0.95332193
sp|O95352|ATG7_HUMAN	0.26350212	0.19149946
sp|P62995-3|TRA2B_HUMAN	0.264431	1.1932944
sp|P22352|GPX3_HUMAN	0.26452637	0
sp|Q13438-4|OS9_HUMAN	0.26464272	0.7827403
sp|Q562R1|ACTBL_HUMAN	0.26511002	0.2178309
sp|P0DMV9|HS71B_HUMAN	0.26700974	0.46176907
sp|P07951|TPM2_HUMAN	0.26861572	1.071201
sp|P00390-2|GSHR_HUMAN	0.2686844	0
sp|O14579|COPE_HUMAN	0.26916122	0.0846519

sp|O43852-5|CALU_HUMAN	0.2701416	0
sp|Q15046-2|SYK_HUMAN	0.2714405	0
sp|Q9NYU2-2|UGGG1_HUMAN	0.27148438	1.1419085
sp|Q15746-3|MYLK_HUMAN	0.2716751	1.0552052
sp|P20674|COX5A_HUMAN	0.27179337	0.7827403
sp|Q969V3-2|NCLN_HUMAN	0.27233124	0.40256184
sp|P31323|KAP3_HUMAN	0.2727127	0.45033538
sp|O95994|AGR2_HUMAN	0.27318764	0.09339783
sp|P31937|3HIDH_HUMAN	0.2732334	0
sp|Q13363|CTBP1_HUMAN	0.2738495	0.06291623
sp|Q8WXH0-2|SYNE2_HUMAN	0.27435684	0.312067
sp|P62857|RS28_HUMAN	0.27459335	0
sp|P08237-3|PFKAM_HUMAN	0.27486038	0.06291623
sp|Q969G5|CAVN3_HUMAN	0.2754326	0.40256184
sp|O60831|PRAF2_HUMAN	0.27578545	0.35795313
sp|P61247|RS3A_HUMAN	0.27610588	1.0634323
sp|P14314-2|GLU2B_HUMAN	0.27657127	0
sp|Q15274|NADC_HUMAN	0.27695465	0.5111962
sp|P51570-2|GALK1_HUMAN	0.2773037	0
sp|Q96AQ6-2|PBIP1_HUMAN	0.27765656	0
sp|Q15172-2|2A5A_HUMAN	0.27780914	0
sp|P19404|NDUV2_HUMAN	0.27798462	0.19149946
sp|P46821|MAP1B_HUMAN	0.27834702	0.19149946
sp|P62244|RS15A_HUMAN	0.27879333	0.45033538
sp|Q15631|TSN_HUMAN	0.2792778	0.40256184
sp|O43865|SAHH2_HUMAN	0.27943134	1.0076748
sp|O00151|PDLI1_HUMAN	0.27974892	0.55270666
sp|P23142-4|FBLN1_HUMAN	0.27979374	0
sp|P48637|GSHB_HUMAN	0.28071213	0.5511424
sp|Q00610-2|CLH1_HUMAN	0.2810135	0.7365137
sp|P04792|HSPB1_HUMAN	0.2815132	0.8469722
sp|P11678|PERE_HUMAN	0.28230095	0
sp|P40763-2|STAT3_HUMAN	0.2827587	0.5511424
sp|Q53EL6-2|PDCD4_HUMAN	0.28328133	0.19149946
sp|P34932|HSP74_HUMAN	0.28370667	0.36969694
sp|Q29865|1C18_HUMAN	0.28378868	0.7061832
sp|P50502|F10A1_HUMAN	0.28383636	1.0634323
sp|Q96EM0|T3HPD_HUMAN	0.28399277	0
sp|Q04637-4|IF4G1_HUMAN	0.28476334	0.7588735
sp|P0CG38|POTEI_HUMAN	0.285614	0
sp|P49368|TCPG_HUMAN	0.2860031	0.91881096
sp|P55060-3|XPO2_HUMAN	0.28613663	0.70235044
sp|Q53GG5-2|PDLI3_HUMAN	0.28671837	0.35795313
sp|Q3LXA3|TKFC_HUMAN	0.28683853	1.1025577
sp|Q9HC38-2|GLOD4_HUMAN	0.28807068	0.51870745

sp|P62249|RS16_HUMAN	0.2902546	0.7827403
sp|P06744|G6PI_HUMAN	0.29073906	0
sp|Q9NWV4|CZIB_HUMAN	0.29211998	0
sp|P31146|COR1A_HUMAN	0.29327393	0.49789834
sp|P30837|AL1B1_HUMAN	0.29371834	0.65625405
sp|Q13217|DNJC3_HUMAN	0.2937851	1.1276597
sp|Q96FV2-2|SCRN2_HUMAN	0.2944317	0.45033538
sp|Q9Y3B3|TMED7_HUMAN	0.2944851	0.45033538
sp|Q9UHB9-4|SRP68_HUMAN	0.2957306	0.43633315
sp|A0A0C4DH25|KVD20_HUMAN	0.29577255	0
sp|Q99733-2|NP1L4_HUMAN	0.29583454	0.35795313
sp|Q9HB07|MYG1_HUMAN	0.29623413	0.61034113
sp|O14980|XPO1_HUMAN	0.29625893	1.2095301
sp|P14174|MIF_HUMAN	0.29696655	0.35795313
sp|Q9Y295|DRG1_HUMAN	0.29714012	0.19149946
sp|Q14974|IMB1_HUMAN	0.29805183	0.34197828
sp|O75489|NDUS3_HUMAN	0.29852676	0.61034113
sp|P16298-2|PP2BB_HUMAN	0.29991722	0
sp|Q99832|TCPH_HUMAN	0.30250168	0
sp|P98095-2|FBLN2_HUMAN	0.30307388	0.9847622
sp|P36873-2|PP1G_HUMAN	0.303545	0.19149946
sp|P14866|HNRPL_HUMAN	0.3055172	0
sp|P31947-2|1433S_HUMAN	0.3057289	0
sp|O15355|PPM1G_HUMAN	0.3061123	0.35795313
sp|Q9BUF5|TBB6_HUMAN	0.30717468	0
sp|P46782|RS5_HUMAN	0.30729675	0.99478513
sp|Q9H008|LHPP_HUMAN	0.30768967	0.37060758
sp|P46777|RL5_HUMAN	0.30955124	0.37410614
sp|Q9GZM7-3|TINAL_HUMAN	0.30975342	0.45033538
sp|O75306-2|NDUS2_HUMAN	0.31104088	0.7061832
sp|Q92629-2|SGCD_HUMAN	0.3122902	0
sp|P05165-2|PCCA_HUMAN	0.31305504	0.15517515
sp|P62081|RS7_HUMAN	0.31306076	0.5204253
sp|Q8TD06|AGR3_HUMAN	0.31314468	0
sp|P12814|ACTN1_HUMAN	0.3149929	0
sp|P46779-2|RL28_HUMAN	0.31513214	0.7827403
sp|Q13347|EIF3I_HUMAN	0.31519127	0.7061832
sp|Q13464|ROCK1_HUMAN	0.31635857	0
sp|Q9NY15|STAB1_HUMAN	0.3177986	0.7827403
sp|P26639-2|SYTC_HUMAN	0.31922913	0.5283125
sp|O00170|AIP_HUMAN	0.3196411	0.35795313
sp|Q92499|DDX1_HUMAN	0.320755	0.80229825
sp|P54578-2|UBP14_HUMAN	0.32333755	0.19149946
sp|P02753|RET4_HUMAN	0.32358932	0.7827403
sp|P56134-3|ATPK_HUMAN	0.32423782	0

sp|P62633-3|CNBP_HUMAN	0.32574654	0.45033538
sp|P48426-2|PI42A_HUMAN	0.3271141	0.7827403
sp|Q5TFE4|NT5D1_HUMAN	0.32800102	0
sp|Q01813|PFKAP_HUMAN	0.3284359	1.2607617
sp|Q9Y5Z4-2|HEBP2_HUMAN	0.32896042	0.7827403
sp|P02760|AMBP_HUMAN	0.3293171	0.6509351
sp|Q5JPE7-2|NOMO2_HUMAN	0.32949066	0.656254
sp|Q9NP79|VTA1_HUMAN	0.32958794	0.45033538
sp|P22314-2|UBA1_HUMAN	0.33034515	1.1102507
sp|P25685-2|DNJB1_HUMAN	0.33055305	0.2178309
sp|P36269-2|GGT5_HUMAN	0.33127022	1.0076748
sp|Q7Z7G0|TARSH_HUMAN	0.33253098	0
sp|Q9BZQ8|NIBAN_HUMAN	0.33382416	0
sp|Q96G03|PGM2_HUMAN	0.33461952	0.87291557
sp|Q13310-2|PABP4_HUMAN	0.33524895	0
sp|P78527|PRKDC_HUMAN	0.33714294	0.06291623
sp|P62829|RL23_HUMAN	0.33789444	0.91601294
sp|Q96JJ3-3|ELMO2_HUMAN	0.3385849	0
sp|O95373|IPO7_HUMAN	0.3390751	0.19149946
sp|Q8N5K1|CISD2_HUMAN	0.3394699	0.7827403
sp|P20338|RAB4A_HUMAN	0.34140015	0.656254
sp|P00740-2|FA9_HUMAN	0.341465	1.0634323
sp|P20810-10|ICAL_HUMAN	0.3430748	0.5204253
sp|Q9UJZ1-2|STML2_HUMAN	0.34315586	1.1932944
sp|Q15185-3|TEBP_HUMAN	0.34316254	0
sp|P46459|NSF_HUMAN	0.34316254	1.120602
sp|P46063|RECQ1_HUMAN	0.34387207	0.57986265
sp|Q15005|SPCS2_HUMAN	0.34413147	0.7827403
sp|Q15661|TRYB1_HUMAN	0.34524155	0.99757636
sp|Q8IUZ5|AT2L2_HUMAN	0.34602356	0.7827403
sp|P18084|ITB5_HUMAN	0.3482628	1.1505735
sp|Q09161|NCBP1_HUMAN	0.351614	0.7827403
sp|P42704|LPPRC_HUMAN	0.35178375	0.21439649
sp|P62266|RS23_HUMAN	0.3520584	1.1505735
sp|Q13435|SF3B2_HUMAN	0.35315514	0.45033538
sp|P19525-2|E2AK2_HUMAN	0.3538971	0
sp|P07951-3|TPM2_HUMAN	0.35395432	0.6298893
sp|Q96C86|DCPS_HUMAN	0.35472488	0.9533461
sp|Q14BN4-2|SLMAP_HUMAN	0.35473633	0
sp|P23368|MAOM_HUMAN	0.35550308	0.37060758
sp|Q9Y5P6-2|GMPPB_HUMAN	0.3557663	0.47871065
sp|Q08209-2|PP2BA_HUMAN	0.35632706	0
sp|P62851|RS25_HUMAN	0.35944366	1.1932944
sp|Q9UBT2|SAE2_HUMAN	0.3601818	0.9533461
sp|Q9UHD8-3|SEPT9_HUMAN	0.36175537	0.19149946

sp|P06396|GELS_HUMAN	0.361763	0.656254
sp|P50135|HNMT_HUMAN	0.36283493	0
sp|Q6UW02|CP20A_HUMAN	0.36527252	0.7827403
sp|Q9Y3Z3|SAMH1_HUMAN	0.36686516	1.0745447
sp|A0A0C4DH31|HV118_HUMAN	0.3687935	0
sp|Q9BVK6|TMED9_HUMAN	0.3698864	1.1932944
sp|Q9UBE0|SAE1_HUMAN	0.3715353	0.8272904
sp|Q13496|MTM1_HUMAN	0.37345982	0.19149946
sp|Q04446|GLGB_HUMAN	0.37421417	0.3852666
sp|Q04828|AK1C1_HUMAN	0.37528992	0.35795313
sp|P01591|IGJ_HUMAN	0.37645912	0.19149946
sp|P35813-3|PPM1A_HUMAN	0.37686157	0.656254
sp|P00492|HPRT_HUMAN	0.37829018	0.5111962
sp|O76011|KRT34_HUMAN	0.38426018	0
sp|O95394-3|AGM1_HUMAN	0.3846321	0
sp|Q9BWM7|SFXN3_HUMAN	0.38568306	0.21439649
sp|P50440-3|GATM_HUMAN	0.3866005	1.1932944
sp|P54136|SYRC_HUMAN	0.38690186	0.18414244
sp|P48668|K2C6C_HUMAN	0.3878994	1.2392054
sp|P08236-2|BGLR_HUMAN	0.38990402	0.7588735
sp|P14324|FPPS_HUMAN	0.3903885	0.19149946
sp|Q13630|FCL_HUMAN	0.39142227	0.7061832
sp|P49959-3|MRE11_HUMAN	0.3927002	0.7827403
sp|P0DJI8|SAA1_HUMAN	0.3928795	0
sp|O60547-2|GMDS_HUMAN	0.39291573	1.2095301
sp|P60891|PRPS1_HUMAN	0.39474106	1.1932944
sp|P49841-2|GSK3B_HUMAN	0.39494514	0.6070219
sp|P62888|RL30_HUMAN	0.3954277	0.7827403
sp|O95479|G6PE_HUMAN	0.39764023	0.95332193
sp|P15374|UCHL3_HUMAN	0.39779663	0.45033538
sp|P62854|RS26_HUMAN	0.40515137	1.1932944
sp|Q9P0J0-2|NDUAD_HUMAN	0.40541267	0.45033538
sp|P48506|GSH1_HUMAN	0.40666008	0.7827403
sp|O43252|PAPS1_HUMAN	0.40681458	0.7827403
sp|P54886-2|P5CS_HUMAN	0.40784454	0
sp|Q6UW68|TM205_HUMAN	0.408741	1.1932944
sp|P46109|CRKL_HUMAN	0.4094982	0.5204253
sp|Q13885|TBB2A_HUMAN	0.40954018	0.656254
sp|P26640|SYVC_HUMAN	0.40983963	0.90036625
sp|P30043|BLVRB_HUMAN	0.40997314	0.8983557
sp|P31513|FMO3_HUMAN	0.41505623	0.7827403
sp|Q9Y3C8|UFC1_HUMAN	0.41950607	0.19149946
sp|O95816|BAG2_HUMAN	0.4224491	1.1932944
sp|Q8WVV9-5|HNRLL_HUMAN	0.42247772	0.91601294
sp|Q5VW32|BROX_HUMAN	0.4275608	1.1932944

sp|Q05682|CALD1_HUMAN	0.4298973	0.97209185
sp|P63279|UBC9_HUMAN	0.43180084	0
sp|P11498|PYC_HUMAN	0.43257332	0.6070219
sp|P61254|RL26_HUMAN	0.4333992	1.2941489
sp|Q06278|AOXA_HUMAN	0.4445114	1.1932944
sp|Q7L576|CYFP1_HUMAN	0.4464178	0
sp|Q92556|ELMO1_HUMAN	0.44747925	0.7061832
sp|P57737-4|CORO7_HUMAN	0.44751167	1.1932944
sp|O43488|ARK72_HUMAN	0.45007896	0.5692702
sp|Q96RF0-2|SNX18_HUMAN	0.4574108	0.7827403
sp|Q96N66-3|MBOA7_HUMAN	0.4582367	0.45033538
sp|Q96DG6|CMBL_HUMAN	0.4596176	0.6070219
sp|P07360|CO8G_HUMAN	0.467165	1.1932944
sp|P46736-3|BRCC3_HUMAN	0.4681282	0.19149946
sp|Q92599-2|SEPT8_HUMAN	0.47459412	0.6070219
sp|Q14194-2|DPYL1_HUMAN	0.4849434	0.656254
sp|P23083|HV102_HUMAN	0.48884583	0.656254
sp|P79483|DRB3_HUMAN	0.49033737	0
sp|P10523|ARRS_HUMAN	0.49035835	0.656254
sp|A0A0C4DH38|HV551_HUMAN	0.49225616	1.1932944
sp|O43396|TXNL1_HUMAN	0.49342155	1.1932944
sp|P02765|FETUA_HUMAN	0.49550247	1.3006523
sp|P08842|STS_HUMAN	0.49656868	0.45033538
sp|Q9H3K6-2|BOLA2_HUMAN	0.4970131	0.656254
sp|P07358|CO8B_HUMAN	0.4978714	1.2095301
sp|P05090|APOD_HUMAN	0.5016899	1.1505735
sp|P02746|C1QB_HUMAN	0.5036011	1.1932944
sp|Q13247-3|SRSF6_HUMAN	0.51187515	1.1932944
sp|Q9P265|DIP2B_HUMAN	0.51350975	0.656254
sp|Q8TCS8|PNPT1_HUMAN	0.5220642	0.6070219
sp|Q92878-2|RAD50_HUMAN	0.52233696	1.1505735
sp|O14791-2|APOL1_HUMAN	0.52560043	1.1932944
sp|Q9NZT2-2|OGFR_HUMAN	0.53010654	1.1505735
sp|A1L0T0|ILVBL_HUMAN	0.55163574	0.6070219
sp|P68371|TBB4B_HUMAN	0.5518646	0
sp|P39023|RL3_HUMAN	0.5535717	1.1932944
sp|Q9BS40|LXN_HUMAN	0.5641861	1.1505735
sp|Q14697|GANAB_HUMAN	0.5652733	1.1932944
sp|O94788-4|AL1A2_HUMAN	0.5664654	0.656254
sp|Q9UNS2|CSN3_HUMAN	0.5744152	0.656254
sp|A1L4H1|SRCRL_HUMAN	0.58546066	1.1932944
sp|P12111-4|CO6A3_HUMAN	0.5869484	0.656254
sp|Q9BU23-3|LMF2_HUMAN	0.58914566	1.1932944
sp|O00560-2|SDCB1_HUMAN	0.59214973	0.45033538
sp|O95050-2|INMT_HUMAN	0.5978737	1.1932944

sp|P04066|FUCO_HUMAN	0.6091118	1.1932944
sp|P14207|FOLR2_HUMAN	0.6140003	1.1932944
sp|A0A0B4J1X8|HV343_HUMAN	0.6163807	0.656254
sp|P12814-2|ACTN1_HUMAN	0.6196747	1.1932944
sp|P43353-2|AL3B1_HUMAN	0.64328575	0
sp|P0DOX7|IGK_HUMAN	0.6444702	1.1932944
sp|Q8TCJ2|STT3B_HUMAN	0.65098286	1.1932944
sp|O75116|ROCK2_HUMAN	0.6530819	0.6070219
sp|P06310|KV230_HUMAN	0.6617775	0.656254
sp|P55058|PLTP_HUMAN	0.6653309	0.656254
sp|P0C0L4|CO4A_HUMAN	0.67402077	0.45033538
sp|Q99538-2|LGMN_HUMAN	0.6775875	1.1932944
sp|P08779|K1C16_HUMAN	0.68990517	0.656254
sp|Q14697-2|GANAB_HUMAN	0.6995659	0.656254
sp|Q3SY69|AL1L2_HUMAN	0.70443344	1.1932944
sp|P01743|HV146_HUMAN	0.7168102	0.7827403
sp|Q08170|SRSF4_HUMAN	0.7343111	0.656254
sp|P31946-2|1433B_HUMAN	0.7377186	0.656254
sp|P30443|1A01_HUMAN	0.7401924	1.1932944
sp|P50238|CRIP1_HUMAN	0.76348686	1.1932944
sp|P02747|C1QC_HUMAN	0.76496506	1.1932944
sp|Q0ZGT2-4|NEXN_HUMAN	0.7787266	0.656254
sp|P01624|KV315_HUMAN	0.78562355	1.1932944
sp|P01782|HV309_HUMAN	0.7932701	0.656254
sp|P02533|K1C14_HUMAN	0.7994385	0.656254
sp|P54577|SYYC_HUMAN	0.807806	0.35795313
sp|P0DOX2|IGA2_HUMAN	0.8562794	1.1932944
sp|P01834|IGKC_HUMAN	0.9578514	1.1932944
sp|P00748|FA12_HUMAN	0.9743099	0.45033538
sp|P48741|HSP77_HUMAN	0.9889412	0.656254
sp|P19012-2|K1C15_HUMAN	1.027462	0.656254
sp|P01780|HV307_HUMAN	1.0536861	0.656254
sp|P0C0L5|CO4B_HUMAN	1.0536976	0.656254
sp|P0DP03|HV335_HUMAN	1.0576782	1.1932944
sp|P01619|KV320_HUMAN	1.1066151	0.656254
sp|P35542|SAA4_HUMAN	1.1087036	1.1932944
sp|P02461|CO3A1_HUMAN	1.2196217	1.1932944
sp|O94875-11|SRBS2_HUMAN	1.3355141	0.656254
sp|A0A075B6P5|KV228_HUMAN	1.3551102	0.656254
sp|Q04695|K1C17_HUMAN	1.3604717	0.656254
