# Supplementary material for: Peptidylarginine deiminase 2 citrullinates MZB1 and promotes the secretion of IgM and IgA
Source: Front Immunol. 2023 Nov 29;14:1290585. doi: 10.3389/fimmu.2023.1290585 (PMC10716219; doi:10.3389/fimmu.2023.1290585)
Supplement: Supplementary file 5 [file DataSheet_5.pdf]

Supplemental Table 5: RA-ILD3 vs controls

Accession #	Fold Change	p value (-log10)
sp|Q15109-10|RAGE_HUMAN	-1.7946758	2.1818786
sp|P12429|ANXA3_HUMAN	-1.4201756	7.665951
sp|P22748|CAH4_HUMAN	-1.4111576	2.6576471
sp|O96009|NAPSA_HUMAN	-1.3504906	2.6576471
sp|P60903|S10AA_HUMAN	-1.212883	1.6960104
sp|Q9NZA1-2|CLIC5_HUMAN	-1.1563549	3.5908275
sp|P12821-2|ACE_HUMAN	-1.1470909	2.6576471
sp|P13686|PPA5_HUMAN	-1.1128521	1.6960104
sp|P09668|CATH_HUMAN	-1.0090942	4.051346
sp|P59665|DEF1_HUMAN	-0.9286194	2.1818786
sp|P26440|IVD_HUMAN	-0.9248428	1.6960104
sp|Q01469|FABP5_HUMAN	-0.8989906	4.051346
sp|Q10589-2|BST2_HUMAN	-0.8961144	1.6960104
sp|P62805|H4_HUMAN	-0.8817921	6.3204336
sp|P05362|ICAM1_HUMAN	-0.880209	5.869831
sp|P11233|RALA_HUMAN	-0.8714981	2.1818786
sp|P51659|DHB4_HUMAN	-0.8707886	12.110734
sp|O95810|CAVN2_HUMAN	-0.8615322	3.0251918
sp|Q9Y624|JAM1_HUMAN	-0.8435745	2.6576471
sp|P31949|S10AB_HUMAN	-0.8298607	2.6576471
sp|P21397|AOFA_HUMAN	-0.8230629	4.890839
sp|P02792|FRIL_HUMAN	-0.8212318	3.7273152
sp|P05164-3|PERM_HUMAN	-0.8195458	10.055915
sp|P26447|S10A4_HUMAN	-0.8189716	2.6576471
sp|P02743|SAMP_HUMAN	-0.8025971	3.760603
sp|P41218|MNDA_HUMAN	-0.7939835	1.9647322
sp|P50895|BCAM_HUMAN	-0.7849693	6.7698927
sp|P49913|CAMP_HUMAN	-0.767683	1.6960104
sp|P00167-2|CYB5_HUMAN	-0.7625084	2.1818786
sp|P61626|LYSC_HUMAN	-0.7623711	2.6576471
sp|Q8WWI1-5|LMO7_HUMAN	-0.7521801	4.6488075
sp|P49407-2|ARRB1_HUMAN	-0.746706	2.6576471
sp|P05091|ALDH2_HUMAN	-0.7458096	10.844091
sp|Q6PIU2-2|NCEH1_HUMAN	-0.7194748	3.1266599
sp|P01903|DRA_HUMAN	-0.7106037	2.1818786
sp|Q96TC7|RMD3_HUMAN	-0.6974945	1.6960104
sp|Q08722-2|CD47_HUMAN	-0.6934414	1.6960104
sp|P09467|F16P1_HUMAN	-0.6905098	5.180782
sp|Q8NF37|PCAT1_HUMAN	-0.6820755	2.3143692
sp|Q6NZI2|CAVN1_HUMAN	-0.6769028	6.119484
sp|P09110|THIK_HUMAN	-0.6737862	2.1818786
sp|Q02318|CP27A_HUMAN	-0.6718674	1.6960104
sp|P08311|CATG_HUMAN	-0.6708489	4.051346

sp|P09758|TACD2_HUMAN	-0.6634941	2.1818786
sp|O43760-2|SNG2_HUMAN	-0.6594238	1.6960104
sp|O00159-3|MYO1C_HUMAN	-0.6518765	6.113284
sp|Q9UGT4|SUSD2_HUMAN	-0.6466389	2.3979244
sp|P20160|CAP7_HUMAN	-0.6432076	1.6960104
sp|Q07157|ZO1_HUMAN	-0.6279945	4.964395
sp|P16284-3|PECA1_HUMAN	-0.6210651	6.3098373
sp|Q13510-2|ASAH1_HUMAN	-0.6092529	6.3204336
sp|P18428|LBP_HUMAN	-0.6047058	1.6960104
sp|P12111-2|CO6A3_HUMAN	-0.6045895	2.8384566
sp|P05107|ITB2_HUMAN	-0.594799	4.509013
sp|P61106|RAB14_HUMAN	-0.5914383	4.7039127
sp|P08473|NEP_HUMAN	-0.5878916	1.6960104
sp|Q9UFN0|NPS3A_HUMAN	-0.5808258	1.6960104
sp|P50225|ST1A1_HUMAN	-0.5761528	1.6960104
sp|P23786|CPT2_HUMAN	-0.575634	1.5646582
sp|Q15599-2|NHRF2_HUMAN	-0.568903	2.6576471
sp|P35241|RADI_HUMAN	-0.5682592	2.5461748
sp|P84103-2|SRSF3_HUMAN	-0.5641499	2.058973
sp|P02786|TFR1_HUMAN	-0.558136	4.051346
sp|Q6YN16|HSDL2_HUMAN	-0.5546532	1.6960104
sp|Q9HB40|RISC_HUMAN	-0.5458317	1.6960104
sp|Q03135|CAV1_HUMAN	-0.5396805	1.9647322
sp|Q86VB7-2|C163A_HUMAN	-0.5356674	8.558914
sp|P07339|CATD_HUMAN	-0.5223236	6.689198
sp|P51149|RAB7A_HUMAN	-0.5221939	4.876761
sp|P02750|A2GL_HUMAN	-0.5206299	2.058973
sp|P24158|PRTN3_HUMAN	-0.5085659	1.6960104
sp|P05026-2|AT1B1_HUMAN	-0.5036888	2.2323174
sp|P07355-2|ANXA2_HUMAN	-0.5031147	4.6930394
sp|P09525|ANXA4_HUMAN	-0.4916973	6.6426344
sp|Q13740-2|CD166_HUMAN	-0.490305	4.005173
sp|Q7Z406|MYH14_HUMAN	-0.4891434	6.9318466
sp|Q8IV08|PLD3_HUMAN	-0.4888783	1.6960104
sp|Q9UHG3|PCYOX_HUMAN	-0.4877491	4.3100786
sp|P01011|AACT_HUMAN	-0.4869289	4.822602
sp|P0DP25|CALM3_HUMAN	-0.4857369	3.5908275
sp|P04083|ANXA1_HUMAN	-0.4837818	7.554525
sp|Q16698-2|DECR_HUMAN	-0.4823761	3.9411435
sp|P51648-2|AL3A2_HUMAN	-0.4745617	4.051346
sp|P42765|THIM_HUMAN	-0.4709015	4.3259945
sp|Q9NZN4|EHD2_HUMAN	-0.4679947	6.5985336
sp|Q96TA1-2|NIBL1_HUMAN	-0.4605503	3.5908275
sp|Q16762|THTR_HUMAN	-0.4599819	2.1818786
sp|P08729|K2C7_HUMAN	-0.4582329	11.59981

sp|P17213|BPI_HUMAN	-0.4571991	1.5646582
sp|Q5SSJ5-2|HP1B3_HUMAN	-0.4551811	2.1818786
sp|P02769|ALBU_BOVIN	-0.4551582	2.8963943
sp|P55290-4|CAD13_HUMAN	-0.4547901	1.5646582
sp|P13804|ETFA_HUMAN	-0.4528637	4.509013
sp|Q9H0U4|RAB1B_HUMAN	-0.4520569	1.6960104
sp|P61421|VA0D1_HUMAN	-0.4447193	3.103845
sp|P04040|CATA_HUMAN	-0.4403496	8.905797
sp|P05109|S10A8_HUMAN	-0.4378967	3.103845
sp|Q15907-2|RB11B_HUMAN	-0.4344215	3.4656596
sp|O75390|CISY_HUMAN	-0.4323235	4.538682
sp|Q13228-4|SBP1_HUMAN	-0.423996	10.208771
sp|Q13011|ECH1_HUMAN	-0.4182987	4.964395
sp|Q9H223|EHD4_HUMAN	-0.4151306	4.051346
sp|P62987|RL40_HUMAN	-0.4110222	2.4829872
sp|P51572-2|BAP31_HUMAN	-0.4093552	3.5908275
sp|P80188|NGAL_HUMAN	-0.4045868	2.3047035
sp|O95340-2|PAPS2_HUMAN	-0.4039936	1.3815327
sp|P49327|FAS_HUMAN	-0.4026756	7.8719664
sp|P16671-4|CD36_HUMAN	-0.4025955	2.1818786
sp|P43304|GPDM_HUMAN	-0.4015522	3.5908275
sp|P23381|SYWC_HUMAN	-0.4003544	3.9651341
sp|Q92817|EVPL_HUMAN	-0.3982086	3.863062
sp|P05783|K1C18_HUMAN	-0.3964844	7.252534
sp|P07099|HYEP_HUMAN	-0.3942452	4.8271813
sp|O43837|IDH3B_HUMAN	-0.3930492	1.6960104
sp|Q99715-4|COCA1_HUMAN	-0.3927822	1.6960104
sp|O15247|CLIC2_HUMAN	-0.3889275	2.3047035
sp|P48735-2|IDHP_HUMAN	-0.3881397	4.051346
sp|P50995-2|ANX11_HUMAN	-0.3852654	3.9439213
sp|Q9UHQ9|NB5R1_HUMAN	-0.3827858	2.1818786
sp|P30512|1A29_HUMAN	-0.3758812	1.4104178
sp|P09960|LKHA4_HUMAN	-0.3755417	6.9708586
sp|P04179-4|SODM_HUMAN	-0.3748474	2.6997027
sp|P50148|GNAQ_HUMAN	-0.3715019	2.1818786
sp|Q6NUK1-2|SCMC1_HUMAN	-0.3695698	2.4829872
sp|P40121|CAPG_HUMAN	-0.3683872	2.4314263
sp|P17931|LEG3_HUMAN	-0.3678207	2.6576471
sp|O60234|GMFG_HUMAN	-0.3625107	1.6960104
sp|O15230|LAMA5_HUMAN	-0.3601475	6.0711555
sp|Q9UBQ0-2|VPS29_HUMAN	-0.35709	1.6960104
sp|Q14344|GNA13_HUMAN	-0.3550758	2.1818786
sp|O75695|XRP2_HUMAN	-0.3537598	1.4104178
sp|P30048-2|PRDX3_HUMAN	-0.3534584	2.6576471
sp|P10253|LYAG_HUMAN	-0.3475781	3.1266599

sp|Q99536|VAT1_HUMAN	-0.3471508	2.9903855
sp|P11310-2|ACADM_HUMAN	-0.3446541	4.051346
sp|P37837|TALDO_HUMAN	-0.3439293	7.9589314
sp|Q9H4M9|EHD1_HUMAN	-0.3435116	2.56956
sp|P62820|RAB1A_HUMAN	-0.3433304	2.1818786
sp|Q9UL25|RAB21_HUMAN	-0.3342934	2.151766
sp|Q06830|PRDX1_HUMAN	-0.3335342	4.7229815
sp|P61604|CH10_HUMAN	-0.3313484	3.1266599
sp|Q9HDC9|APMAP_HUMAN	-0.3300076	3.545398
sp|O75348|VATG1_HUMAN	-0.3287506	1.6960104
sp|P22897|MRC1_HUMAN	-0.3262749	4.80653
sp|P36543-2|VATE1_HUMAN	-0.3259201	1.6960104
sp|P20645|MPRD_HUMAN	-0.3256989	1.6960104
sp|Q02218-2|ODO1_HUMAN	-0.3247566	3.316884
sp|Q96I99|SUCB2_HUMAN	-0.3229885	2.923088
sp|P30040|ERP29_HUMAN	-0.3214607	1.827201
sp|Q02252-2|MMSA_HUMAN	-0.3211117	2.4829872
sp|Q00765|REEP5_HUMAN	-0.3208427	3.1266599
sp|Q9Y6N5|SQOR_HUMAN	-0.3194675	4.42992
sp|Q9NQC3|RTN4_HUMAN	-0.3193054	1.5646582
sp|P56199|ITA1_HUMAN	-0.3186779	5.4113336
sp|P04080|CYTB_HUMAN	-0.3173714	1.6960104
sp|P07910-2|HNRPC_HUMAN	-0.3160648	2.1818786
sp|O94760|DDAH1_HUMAN	-0.3137894	1.4716977
sp|Q6YHK3|CD109_HUMAN	-0.3137074	3.8053288
sp|P24752|THIL_HUMAN	-0.3118401	4.0641108
sp|P50213|IDH3A_HUMAN	-0.3099766	2.9091687
sp|O15144|ARPC2_HUMAN	-0.3026543	2.9807727
sp|P62258|1433E_HUMAN	-0.2992439	2.1818786
sp|P05787-2|K2C8_HUMAN	-0.2991314	8.819769
sp|P00568|KAD1_HUMAN	-0.2982254	3.4731379
sp|P51148-2|RAB5C_HUMAN	-0.2973766	1.6960104
sp|Q13404|UB2V1_HUMAN	-0.29599	1.6960104
sp|P09917|LOX5_HUMAN	-0.2958374	1.560883
sp|P07988|PSPB_HUMAN	-0.2955666	2.5461748
sp|P16435|NCPR_HUMAN	-0.2932892	2.457975
sp|Q9H3N1|TMX1_HUMAN	-0.2904739	1.6960104
sp|P14780|MMP9_HUMAN	-0.2851467	2.1190927
sp|Q9NTX5-6|ECHD1_HUMAN	-0.2830849	2.4982266
sp|P40926|MDHM_HUMAN	-0.2827225	6.8298965
sp|P22061-2|PIMT_HUMAN	-0.2820168	2.1818786
sp|P26038|MOES_HUMAN	-0.2779617	9.830367
sp|Q04917|1433F_HUMAN	-0.2776279	4.2870226
sp|P10301|RRAS_HUMAN	-0.2775421	1.6960104
sp|P27105|STOM_HUMAN	-0.276453	2.8132849

sp|P55268|LAMB2_HUMAN	-0.2762642	6.357367
sp|A6NMZ7|CO6A6_HUMAN	-0.2754116	1.8087089
sp|P30740|ILEU_HUMAN	-0.2719536	3.5908275
sp|P84095|RHOG_HUMAN	-0.2705193	1.4104178
sp|Q14011-2|CIRBP_HUMAN	-0.2665091	1.6960104
sp|P22695|QCR2_HUMAN	-0.266325	3.9417877
sp|P02763|A1AG1_HUMAN	-0.2661858	1.6960104
sp|P28072|PSB6_HUMAN	-0.2643738	1.6960104
sp|P54819-2|KAD2_HUMAN	-0.2643604	1.6960104
sp|P36957|ODO2_HUMAN	-0.2633457	4.509013
sp|P13797|PLST_HUMAN	-0.2613106	2.352714
sp|P11177-3|ODPB_HUMAN	-0.2592125	1.6832623
sp|P08571|CD14_HUMAN	-0.2581139	1.7590232
sp|O00743-3|PPP6_HUMAN	-0.2577343	1.6960104
sp|P35221|CTNA1_HUMAN	-0.2567787	6.218706
sp|P13073|COX41_HUMAN	-0.2543755	1.6960104
sp|O75955|FLOT1_HUMAN	-0.2521362	2.024977
sp|P59998|ARPC4_HUMAN	-0.252079	2.6576471
sp|P61019|RAB2A_HUMAN	-0.2519512	3.1627245
sp|P35914|HMGCL_HUMAN	-0.2471294	2.151766
sp|P09497-2|CLCB_HUMAN	-0.2460728	2.1818786
sp|P20073-2|ANXA7_HUMAN	-0.2444668	1.9509854
sp|Q92597|NDRG1_HUMAN	-0.2420425	1.3815327
sp|P48047|ATPO_HUMAN	-0.2368012	1.5270832
sp|Q9H4G4|GAPR1_HUMAN	-0.2343731	1.4104178
sp|Q9UIJ7|KAD3_HUMAN	-0.2327251	3.1266599
sp|P84090|ERH_HUMAN	-0.2316456	2.1818786
sp|Q00577|PURA_HUMAN	-0.2309227	2.151766
sp|P21796|VDAC1_HUMAN	-0.228796	2.4331102
sp|P35222|CTNB1_HUMAN	-0.2280865	1.5121045
sp|P35232|PHB_HUMAN	-0.2238922	3.2217336
sp|Q6NVY1|HIBCH_HUMAN	-0.221098	1.3815327
sp|P51858|HDGF_HUMAN	-0.2206936	2.1818786
sp|P99999|CYC_HUMAN	-0.2190323	2.1818786
sp|P22626|ROA2_HUMAN	-0.2182941	4.0610723
sp|Q9HCC0|MCCB_HUMAN	-0.2178021	1.5054473
sp|P11413-2|G6PD_HUMAN	-0.2163086	2.0598862
sp|P01009|A1AT_HUMAN	-0.216116	6.016212
sp|O43491|E41L2_HUMAN	-0.2146015	1.9308543
sp|Q9BS26|ERP44_HUMAN	-0.2145557	2.838374
sp|P17844-2|DDX5_HUMAN	-0.2141151	1.560883
sp|Q9UHB6-4|LIMA1_HUMAN	-0.2113991	1.3815327
sp|P02760|AMBP_HUMAN	-0.2072048	1.9509854
sp|P62873|GBB1_HUMAN	-0.2058067	1.3184075
sp|P23246|SFPQ_HUMAN	-0.204464	2.1952481

sp|P62316-2|SMD2_HUMAN	-0.2037029	1.6960104
sp|P20340-2|RAB6A_HUMAN	-0.2027226	2.2323174
sp|Q08257|QOR_HUMAN	-0.2006283	2.2535503
sp|O75367-2|H2AY_HUMAN	-0.2003784	1.4278674
sp|Q16853|AOC3_HUMAN	-0.198143	1.5942118
sp|P05556|ITB1_HUMAN	-0.1957722	4.9799447
sp|P35237|SPB6_HUMAN	-0.1930389	3.1533113
sp|P11215-2|ITAM_HUMAN	-0.1915836	1.6997428
sp|P27361|MK03_HUMAN	-0.1914883	1.6960104
sp|P10515|ODP2_HUMAN	-0.1908073	1.9190748
sp|P30533|AMRP_HUMAN	-0.1884346	1.6997428
sp|P49748-2|ACADV_HUMAN	-0.1879902	5.5088854
sp|P40429|RL13A_HUMAN	-0.1871948	1.6960104
sp|P38606|VATA_HUMAN	-0.1865616	2.9903855
sp|Q9Y277-2|VDAC3_HUMAN	-0.1813965	2.653196
sp|P50552|VASP_HUMAN	-0.1753101	2.432837
sp|P08727|K1C19_HUMAN	-0.1746159	4.5162024
sp|P08758|ANXA5_HUMAN	-0.1741943	1.5011283
sp|P11047|LAMC1_HUMAN	-0.1715164	4.874187
sp|P30041|PRDX6_HUMAN	-0.1709061	3.7671235
sp|P00352|AL1A1_HUMAN	-0.1706467	4.9527197
sp|P62993|GRB2_HUMAN	-0.169838	1.827201
sp|P38646|GRP75_HUMAN	-0.1698265	2.8603818
sp|Q99729-3|ROAA_HUMAN	-0.1661644	1.9997257
sp|P10606|COX5B_HUMAN	-0.1642456	1.6960104
sp|P52209-2|6PGD_HUMAN	-0.162281	1.5175526
sp|P14543-2|NID1_HUMAN	-0.1590519	3.4268153
sp|Q00839|HNRPU_HUMAN	-0.1577263	1.872644
sp|P54920|SNAA_HUMAN	-0.1562367	1.6265627
sp|O00264|PGRC1_HUMAN	-0.1554585	2.3143692
sp|Q03252|LMNB2_HUMAN	-0.15485	5.8857155
sp|P16219|ACADS_HUMAN	-0.1546516	1.4278674
sp|Q02543|RL18A_HUMAN	-0.1546459	1.9413493
sp|P05023|AT1A1_HUMAN	-0.1519222	4.0484858
sp|P13796|PLSL_HUMAN	-0.1516838	5.3970337
sp|P00325|ADH1B_HUMAN	-0.1501389	1.3635377
sp|P23141-2|EST1_HUMAN	-0.1501236	4.322409
sp|P08572|CO4A2_HUMAN	-0.1487522	1.867911
sp|Q13185|CBX3_HUMAN	-0.1469383	2.1818786
sp|P27487|DPP4_HUMAN	-0.1467362	1.7590232
sp|P52565|GDIR1_HUMAN	-0.1457233	1.8595492
sp|P21281|VATB2_HUMAN	-0.144105	1.6295799
sp|P00390-2|GSHR_HUMAN	-0.1436386	2.3143692
sp|P04004|VTNC_HUMAN	-0.1404648	2.0727112
sp|P06737-2|PYGL_HUMAN	-0.1395302	2.096182

sp|P10809|CH60_HUMAN	-0.1387253	2.152236
sp|P09622|DLDH_HUMAN	-0.1370926	2.173994
sp|O75369-2|FLNB_HUMAN	-0.1360016	4.2932334
sp|Q07020-2|RL18_HUMAN	-0.1313477	1.6960104
sp|Q02878|RL6_HUMAN	-0.1270618	1.6960104
sp|P20700|LMNB1_HUMAN	-0.1253357	4.6877613
sp|Q14103-3|HNRPD_HUMAN	-0.1238289	1.9473329
sp|Q16555|DPYL2_HUMAN	-0.1199493	1.6404219
sp|P00367|DHE3_HUMAN	-0.1185989	1.9153107
sp|P35580-3|MYH10_HUMAN	-0.1126404	5.4172683
sp|P23528|COF1_HUMAN	-0.110918	1.7565529
sp|P02545|LMNA_HUMAN	-0.1102696	7.0661864
sp|P05198|IF2A_HUMAN	-0.107914	1.9647322
sp|P00387-3|NB5R3_HUMAN	-0.1078396	1.7713763
sp|O14745|NHRF1_HUMAN	-0.1066952	1.6960104
sp|P06576|ATPB_HUMAN	-0.1066494	2.450404
sp|P35579|MYH9_HUMAN	-0.1042519	2.6082938
sp|P15586-2|GNS_HUMAN	-0.1024418	1.6960104
sp|Q86UP2-4|KTN1_HUMAN	-0.0962372	2.3499942
sp|O95831-3|AIFM1_HUMAN	-0.0960865	1.4068714
sp|P07237|PDIA1_HUMAN	-0.0943966	3.2734182
sp|P17655|CAN2_HUMAN	-0.0934277	1.3924598
sp|Q9NRN5-2|OLFL3_HUMAN	-0.0910015	1.7265131
sp|P00505|AATM_HUMAN	-0.0807552	1.3908802
sp|P23396|RS3_HUMAN	-0.0792408	1.572624
sp|O43707|ACTN4_HUMAN	-0.0664043	2.6716063
sp|Q13561-2|DCTN2_HUMAN	-0.0615501	1.3079358
sp|P12109|CO6A1_HUMAN	-0.0550308	1.4578869
sp|Q07954|LRP1_HUMAN	-0.0521545	1.406226
sp|P98160|PGBM_HUMAN	0.0792675	2.398297
sp|P23456|Trypsin	0.08312607	2.653196
sp|P19971|TYPH_HUMAN	0.08837318	2.3149889
sp|P55072|TERA_HUMAN	0.08870125	2.9725993
sp|Q14258|TRI25_HUMAN	0.08972931	2.0727112
sp|P48444|COPD_HUMAN	0.09312439	3.2952836
sp|Q9NYU2-2|UGGG1_HUMAN	0.09363747	1.8576962
sp|P00751|CFAB_HUMAN	0.09413147	3.3201993
sp|P11766|ADHX_HUMAN	0.09490776	2.1242583
sp|P13010|XRCC5_HUMAN	0.09754181	1.7944541
sp|P12270|TPR_HUMAN	0.10106278	1.6099055
sp|P02790|HEMO_HUMAN	0.10350418	2.593232
sp|P62277|RS13_HUMAN	0.10745239	1.5646582
sp|P04075|ALDOA_HUMAN	0.10763741	2.8820472
sp|P27797|CALR_HUMAN	0.10840225	2.8438601
sp|P15374|UCHL3_HUMAN	0.10953617	1.9647322

sp|P60174|TPIS_HUMAN	0.11014938	1.7658234
sp|Q15631|TSN_HUMAN	0.11097145	1.3815327
sp|P35606|COPB2_HUMAN	0.11163712	3.0497963
sp|Q13409-3|DC1I2_HUMAN	0.11255074	1.3080103
sp|Q9BRF8-2|CPPED_HUMAN	0.11283493	1.6960104
sp|Q9UEY8|ADDG_HUMAN	0.1131134	1.3319147
sp|P11277-2|SPTB1_HUMAN	0.1154995	3.213026
sp|P49368|TCPG_HUMAN	0.11598778	3.3390138
sp|Q15019-2|SEPT2_HUMAN	0.11602211	3.8199413
sp|P55786|PSA_HUMAN	0.11661148	1.4931748
sp|P13489|RINI_HUMAN	0.11842728	3.8108344
sp|P04003|C4BPA_HUMAN	0.11902046	1.7647069
sp|P08238|HS90B_HUMAN	0.11961556	2.1952481
sp|P01008|ANT3_HUMAN	0.11963654	3.056411
sp|P02748|CO9_HUMAN	0.11990547	2.3801057
sp|O75083|WDR1_HUMAN	0.12046433	1.7198673
sp|Q92900-2|RENT1_HUMAN	0.12333679	1.3567923
sp|P14868|SYDC_HUMAN	0.12359428	1.8831652
sp|Q15582|BGH3_HUMAN	0.12419128	3.5922449
sp|P35611-2|ADDA_HUMAN	0.12532997	1.6997428
sp|P04196|HRG_HUMAN	0.13068771	1.6545225
sp|P17987|TCPA_HUMAN	0.13184357	3.4120767
sp|P50991-2|TCPD_HUMAN	0.13451958	1.4595377
sp|P12956|XRCC6_HUMAN	0.13542175	2.6867676
sp|P78371|TCPB_HUMAN	0.13794613	3.3269932
sp|Q16181-2|SEPT7_HUMAN	0.14226913	2.2401297
sp|Q15084-2|PDIA6_HUMAN	0.14301682	4.3492994
sp|Q13838-2|DX39B_HUMAN	0.14522362	1.7590232
sp|P36955|PEDF_HUMAN	0.14533997	1.560883
sp|P17174|AATC_HUMAN	0.14637756	3.1092827
sp|P35527|K1C9_HUMAN	0.1491394	1.8521893
sp|Q15942|ZYX_HUMAN	0.1492405	1.9190748
sp|P05166-2|PCCB_HUMAN	0.15037727	1.7713763
sp|P05546|HEP2_HUMAN	0.15332985	1.4278674
sp|P01042-2|KNG1_HUMAN	0.15333557	4.192454
sp|P60660-2|MYL6_HUMAN	0.15379333	3.5844223
sp|P32969|RL9_HUMAN	0.15526009	1.6960104
sp|P11216|PYGB_HUMAN	0.15541649	3.4001613
sp|O75306-2|NDUS2_HUMAN	0.15639114	1.9647322
sp|Q9NZ08-2|ERAP1_HUMAN	0.15642929	2.2613342
sp|P28838-2|AMPL_HUMAN	0.15711403	4.0538487
sp|P07195|LDHB_HUMAN	0.16043854	3.3575702
sp|P10768|ESTD_HUMAN	0.16093445	2.151766
sp|P39656-3|OST48_HUMAN	0.16125488	3.2450993
sp|P50454|SERPH_HUMAN	0.16126823	3.7273152

sp|P62917|RL8_HUMAN	0.16289902	2.4829872
sp|P00747|PLMN_HUMAN	0.16430664	2.329737
sp|Q15293|RCN1_HUMAN	0.16615105	2.151766
sp|P10909-5|CLUS_HUMAN	0.16880798	3.364204
sp|P49591|SYSC_HUMAN	0.17050743	1.5688182
sp|P21980|TGM2_HUMAN	0.17184067	5.034626
sp|P09104-2|ENOG_HUMAN	0.17235947	1.827201
sp|Q9Y5Z4|HEBP2_HUMAN	0.17297363	1.6960104
sp|P13716-2|HEM2_HUMAN	0.1766491	2.1404836
sp|Q15075|EEA1_HUMAN	0.17808533	3.1957111
sp|P04264|K2C1_HUMAN	0.17863846	3.8732924
sp|Q07507|DERM_HUMAN	0.18115997	1.5983955
sp|Q14112-2|NID2_HUMAN	0.18191719	2.4945192
sp|P13798|ACPH_HUMAN	0.18277359	3.4366283
sp|P32119|PRDX2_HUMAN	0.18462372	3.324656
sp|Q8IUX7|AEBP1_HUMAN	0.18579865	2.058973
sp|O43852-3|CALU_HUMAN	0.18707085	1.5646582
sp|Q96G03|PGM2_HUMAN	0.18795776	3.0819354
sp|P06744|G6PI_HUMAN	0.19141388	5.1049714
sp|Q7KZF4|SND1_HUMAN	0.1934967	4.033051
sp|P11021|BIP_HUMAN	0.19404411	9.761793
sp|P34932|HSP74_HUMAN	0.19512367	2.5322723
sp|P08670|VIME_HUMAN	0.19625092	12.945139
sp|P45974-2|UBP5_HUMAN	0.19674873	3.4260464
sp|Q00341-2|VIGLN_HUMAN	0.19779205	1.4751658
sp|P53618|COPB_HUMAN	0.20006752	2.5975564
sp|P29622|KAIN_HUMAN	0.20021439	1.4278674
sp|O14828-2|SCAM3_HUMAN	0.2006855	1.4104178
sp|Q3LXA3|TKFC_HUMAN	0.20126152	1.8992519
sp|P06756-3|ITAV_HUMAN	0.20303535	2.0727112
sp|P23526|SAHH_HUMAN	0.20618057	3.939045
sp|P01031|CO5_HUMAN	0.20630074	4.5719495
sp|Q9Y5M8|SRPRB_HUMAN	0.2074585	2.1818786
sp|P00338|LDHA_HUMAN	0.20765495	2.3798928
sp|P08133|ANXA6_HUMAN	0.20774078	7.768831
sp|O75112-7|LDB3_HUMAN	0.2108059	1.6960104
sp|O00534|VMA5A_HUMAN	0.2110672	2.792622
sp|P53621-2|COPA_HUMAN	0.2111969	2.8653378
sp|P49419-2|AL7A1_HUMAN	0.2119484	4.8271813
sp|Q8IZ83-3|A16A1_HUMAN	0.21495247	2.923088
sp|P18669|PGAM1_HUMAN	0.21733093	3.3575702
sp|P22314|UBA1_HUMAN	0.21953583	3.4109392
sp|Q6XQN6-2|PNCB_HUMAN	0.22079849	2.312627
sp|P13647|K2C5_HUMAN	0.22121048	4.177984
sp|Q9Y4L1|HYOU1_HUMAN	0.22472382	5.1043024

sp|P26373|RL13_HUMAN	0.22521782	1.9647322
sp|P41250|GARS_HUMAN	0.22646332	2.4364867
sp|P13639|EF2_HUMAN	0.22688866	10.2286415
sp|O00151|PDLI1_HUMAN	0.22835922	3.0019732
sp|O00567|NOP56_HUMAN	0.22846031	2.987788
sp|P08236-2|BGLR_HUMAN	0.23007011	2.1818786
sp|Q14847|LASP1_HUMAN	0.23071861	1.3567923
sp|P04259|K2C6B_HUMAN	0.23261642	1.4716977
sp|Q00796|DHSO_HUMAN	0.23573303	1.6960104
sp|Q16643-3|DREB_HUMAN	0.2362442	1.3815327
sp|Q86VP6|CAND1_HUMAN	0.23660469	4.302124
sp|P51888|PRELP_HUMAN	0.23730278	5.4389644
sp|P68871|HBB_HUMAN	0.23765182	1.9190748
sp|Q16647|PTGIS_HUMAN	0.23812866	1.7713763
sp|P04843|RPN1_HUMAN	0.24079323	7.128238
sp|Q9Y315|DEOC_HUMAN	0.24085617	1.5646582
sp|P07814|SYEP_HUMAN	0.2413969	1.5983955
sp|P49961-6|ENTP1_HUMAN	0.24535179	1.4104178
sp|P07738|PMGE_HUMAN	0.24539757	2.1818786
sp|P26640|SYVC_HUMAN	0.24635315	1.8595492
sp|P43243|MATR3_HUMAN	0.24765778	2.4829872
sp|P46782|RS5_HUMAN	0.24921417	1.5270832
sp|O60763-2|USO1_HUMAN	0.25033188	3.292093
sp|Q9Y6C2|EMIL1_HUMAN	0.2532196	5.964717
sp|Q9BS40|LXN_HUMAN	0.25531197	1.3815327
sp|Q08378|GOGA3_HUMAN	0.25557804	1.6960104
sp|P07437|TBB5_HUMAN	0.25600052	1.3815327
sp|Q13263|TIF1B_HUMAN	0.25677872	4.2451677
sp|Q9NPH2|INO1_HUMAN	0.25792885	1.9413493
sp|P39060-1|COIA1_HUMAN	0.2592697	2.6576471
sp|Q63ZY3-3|KANK2_HUMAN	0.26375866	2.4829872
sp|Q9H008|LHPP_HUMAN	0.2661295	1.9413493
sp|P22105-1|TENX_HUMAN	0.27624702	7.847662
sp|Q9NR45|SIAS_HUMAN	0.27723694	3.734273
sp|Q9UBG0|MRC2_HUMAN	0.2777338	2.3388627
sp|O14974-3|MYPT1_HUMAN	0.27806664	3.1208909
sp|P48147|PPCE_HUMAN	0.27841568	2.044628
sp|P27338|AOFB_HUMAN	0.2805977	4.6488075
sp|P36578|RL4_HUMAN	0.28272247	3.103845
sp|P26639-2|SYTC_HUMAN	0.2840023	2.6196775
sp|P00915|CAH1_HUMAN	0.28445053	7.665951
sp|O43175|SERA_HUMAN	0.28564072	2.0928013
sp|Q07065|CKAP4_HUMAN	0.2858925	9.554357
sp|P00450|CERU_HUMAN	0.28695488	11.220342
sp|A1L4H1|SRCRL_HUMAN	0.29042244	1.6960104

sp|O00754-2|MA2B1_HUMAN	0.29284477	2.1818786
sp|P11498|PYC_HUMAN	0.29406834	1.6960104
sp|Q01813-2|PFKAP_HUMAN	0.29421425	1.4278674
sp|P62701|RS4X_HUMAN	0.29473305	2.1818786
sp|P62140|PP1B_HUMAN	0.2952404	1.4104178
sp|P08648|ITA5_HUMAN	0.29639435	1.6960104
sp|Q96C86|DCPS_HUMAN	0.2985916	1.6960104
sp|P12277|KCRB_HUMAN	0.30036163	1.8350728
sp|P00488|F13A_HUMAN	0.30101776	5.226002
sp|Q6PCB0|VWA1_HUMAN	0.303154	1.6960104
sp|P08294|SODE_HUMAN	0.30364227	2.058973
sp|P05534|1A24_HUMAN	0.3069992	1.6960104
sp|P13667|PDIA4_HUMAN	0.31037712	10.021521
sp|P22234-2|PUR6_HUMAN	0.3150158	2.6576471
sp|P35555|FBN1_HUMAN	0.31578445	9.35835
sp|Q9Y678|COPG1_HUMAN	0.31764698	6.253077
sp|P62241|RS8_HUMAN	0.31874084	3.1266599
sp|P06727|APOA4_HUMAN	0.3200302	6.3204336
sp|Q9UBT2|SAE2_HUMAN	0.32409096	2.1818786
sp|P80303-2|NUCB2_HUMAN	0.32975006	2.1818786
sp|P08603|CFAH_HUMAN	0.33151627	14.632371
sp|P04114|APOB_HUMAN	0.33409882	9.866501
sp|O60547-2|GMDS_HUMAN	0.33465576	1.6960104
sp|P46779-2|RL28_HUMAN	0.34303093	1.3815327
sp|Q92896-2|GSLG1_HUMAN	0.34387589	4.051346
sp|P02647|APOA1_HUMAN	0.35307884	11.261091
sp|P08708|RS17_HUMAN	0.35313797	2.0727112
sp|P62266|RS23_HUMAN	0.35681725	1.560883
sp|P18206-2|VINC_HUMAN	0.35928726	14.699317
sp|P49257|LMAN1_HUMAN	0.36786652	3.4656596
sp|O60831|PRAF2_HUMAN	0.37101555	1.6960104
sp|P55809|SCOT1_HUMAN	0.37223244	1.6960104
sp|Q07960|RHG01_HUMAN	0.37650108	3.9417877
sp|P23142|FBLN1_HUMAN	0.37883377	2.432837
sp|P49588-2|SYAC_HUMAN	0.37958527	5.1321483
sp|Q13162|PRDX4_HUMAN	0.38264084	3.1266599
sp|P08237-3|PFKAM_HUMAN	0.39874268	1.9951487
sp|O75746-2|CMC1_HUMAN	0.40784454	1.6960104
sp|P21399|ACOC_HUMAN	0.40868092	3.5908275
sp|Q9HCB6|SPON1_HUMAN	0.40994644	3.218121
sp|P16615|AT2A2_HUMAN	0.41054535	7.665951
sp|P28331-2|NDUS1_HUMAN	0.41399384	2.6576471
sp|Q96D15|RCN3_HUMAN	0.41708374	1.6960104
sp|O94979-10|SC31A_HUMAN	0.418602	1.6960104
sp|P07585|PGS2_HUMAN	0.4290676	5.417897

sp|P51884|LUM_HUMAN	0.43702698	8.112776
sp|P01860|IGHG3_HUMAN	0.445076	2.6576471
sp|P14618-2|KPYM_HUMAN	0.44867325	1.4104178
sp|Q8TDL5|BPIB1_HUMAN	0.45905113	2.1404836
sp|P27169|PON1_HUMAN	0.462965	1.6960104
sp|P21266|GSTM3_HUMAN	0.46615982	3.2221727
sp|P15090|FABP4_HUMAN	0.47262955	3.1266599
sp|P20774|MIME_HUMAN	0.4789791	4.051346
sp|Q9BXN1|ASPN_HUMAN	0.4850235	2.6576471
sp|P21291|CSRP1_HUMAN	0.48706055	5.417897
sp|Q8N2S1|LTBP4_HUMAN	0.489254	5.1954384
sp|P46821|MAP1B_HUMAN	0.49632072	2.1818786
sp|Q9P2E9|RRBP1_HUMAN	0.50429535	14.010107
sp|P40261|NNMT_HUMAN	0.5057678	1.6960104
sp|Q9P2B2|FPRP_HUMAN	0.51496696	2.4982266
sp|Q8TAQ2-2|SMRC2_HUMAN	0.5172024	1.6960104
sp|O43301|HS12A_HUMAN	0.5412369	2.1818786
sp|P0DOX7|IGK_HUMAN	0.5487232	2.1818786
sp|P10643|CO7_HUMAN	0.5596638	4.6930394
sp|Q93052|LPP_HUMAN	0.57123566	4.0641108
sp|Q14195-2|DPYL3_HUMAN	0.57148933	7.6308155
sp|P13611|CSPG2_HUMAN	0.5725651	5.417897
sp|Q15746-2|MYLK_HUMAN	0.58844376	7.023118
sp|P04792|HSPB1_HUMAN	0.5965004	7.2183566
sp|P49821-2|NDUV1_HUMAN	0.59955406	3.5908275
sp|Q12765|SCRN1_HUMAN	0.6042862	2.1818786
sp|P98095-2|FBLN2_HUMAN	0.6089096	5.727641
sp|P07951-3|TPM2_HUMAN	0.61615753	2.1818786
sp|Q12805-2|FBLN3_HUMAN	0.62044334	6.3204336
sp|Q0ZGT2-4|NEXN_HUMAN	0.6363869	4.509013
sp|O14558|HSPB6_HUMAN	0.64740086	2.4829872
sp|Q8TCJ2|STT3B_HUMAN	0.6737709	1.6960104
sp|P24844|MYL9_HUMAN	0.6821308	1.6960104
sp|P39059|COFA1_HUMAN	0.6852684	2.1818786
sp|P01876|IGHA1_HUMAN	0.6911583	3.8503277
sp|P08123|CO1A2_HUMAN	0.6953697	2.4829872
sp|P0DOX8|IGL1_HUMAN	0.7290058	2.1818786
sp|Q15124|PGM5_HUMAN	0.7313175	7.7619967
sp|Q53GG5-2|PDLI3_HUMAN	0.7598858	2.1818786
sp|Q14767|LTBP2_HUMAN	0.7632408	5.4738517
sp|P36269-3|GGT5_HUMAN	0.7655754	3.1266599
sp|Q8NBS9|TXND5_HUMAN	0.7702637	7.665951
sp|P09493-9|TPM1_HUMAN	0.7703991	1.6960104
sp|Q14192|FHL2_HUMAN	0.77052116	2.1818786
sp|P02452|CO1A1_HUMAN	0.80664444	2.058973

sp|P07951|TPM2_HUMAN	0.8124237	3.1266599
sp|O15061|SYNEM_HUMAN	0.8277254	3.5190198
sp|Q9UBX5|FBLN5_HUMAN	0.82930183	4.4116287
sp|P67936-2|TPM4_HUMAN	0.8699379	1.6960104
sp|P09493-8|TPM1_HUMAN	0.87311935	3.5908275
sp|P07451|CAH3_HUMAN	0.8733654	3.1266599
sp|P51911|CNN1_HUMAN	0.98700523	4.8353806
sp|Q9NYL4|FKB11_HUMAN	0.9936676	1.6960104
sp|Q9NR12-2|PDLI7_HUMAN	1.0516167	2.1800864
sp|Q14315-2|FLNC_HUMAN	1.0989723	15.95459
sp|Q8WU39|MZB1_HUMAN	1.1754417	3.1266599
sp|P0DOY3|IGLC3_HUMAN	1.1758022	2.1818786
sp|Q8WX93-5|PALLD_HUMAN	1.1878853	1.6960104
sp|P01859|IGHG2_HUMAN	1.2014732	4.509013
sp|Q01995|TAGL_HUMAN	1.2401638	9.004438
sp|Q9UMS6-2|SYNP2_HUMAN	1.2932491	4.051346
sp|P01861|IGHG4_HUMAN	1.3670502	3.5908275
sp|P01871-2|IGHM_HUMAN	1.4475918	2.1818786
sp|P0DOX5|IGG1_HUMAN	1.6017609	4.964395
sp|Q05682|CALD1_HUMAN	1.6363449	1.6960104
sp|P17661|DESM_HUMAN	1.7278194	14.778499
sp|P04229|2B11_HUMAN	-2.8930798	0.656254
sp|O00757|F16P2_HUMAN	-1.7075348	1.1932944
sp|Q9BW04|SARG_HUMAN	-1.6432972	0.7827403
sp|P08246|ELNE_HUMAN	-1.3534603	1.1932944
sp|P02741|CRP_HUMAN	-1.256094	1.1932944
sp|P13761|2B17_HUMAN	-1.1777973	0
sp|P16422|EPCAM_HUMAN	-1.1038246	1.1932944
sp|P37235|HPCL1_HUMAN	-1.0896969	0.656254
sp|O00592-2|PODXL_HUMAN	-0.9956284	1.1932944
sp|Q29963|1C06_HUMAN	-0.9643707	0.656254
sp|P13760|2B14_HUMAN	-0.9356289	1.2773042
sp|P08263|GSTA1_HUMAN	-0.9255199	0
sp|P20702|ITAX_HUMAN	-0.8820229	0.656254
sp|P33151|CADH5_HUMAN	-0.8706608	1.1932944
sp|Q16777|H2A2C_HUMAN	-0.8573284	1.1932944
sp|Q9UHN6-2|CEIP2_HUMAN	-0.8241749	1.1932944
sp|P63218|GBG5_HUMAN	-0.818264	1.1932944
sp|Q93077|H2A1C_HUMAN	-0.7896423	0.7827403
sp|Q01955|CO4A3_HUMAN	-0.7775497	1.1932944
sp|Q9HD89|RETN_HUMAN	-0.7740288	0.7827403
sp|O43795-2|MYO1B_HUMAN	-0.7717695	0.45033538
sp|P12830|CADH1_HUMAN	-0.7701187	0.19149946
sp|P28330|ACADL_HUMAN	-0.7540493	1.1932944
sp|P54108-2|CRIS3_HUMAN	-0.7452393	0.656254

sp|O95837|GNA14_HUMAN	-0.7261391	0.656254
sp|P28799-3|GRN_HUMAN	-0.7151375	1.1505735
sp|Q8NBQ5|DHB11_HUMAN	-0.6837521	1.1932944
sp|P06753-6|TPM3_HUMAN	-0.6835861	0.656254
sp|Q13751|LAMB3_HUMAN	-0.6515122	0.7061832
sp|P22894|MMP8_HUMAN	-0.6477661	0.19149946
sp|P38159|RBMX_HUMAN	-0.6335316	0.656254
sp|A6NMY6|AXA2L_HUMAN	-0.6320591	0.656254
sp|Q9H8L6|MMRN2_HUMAN	-0.6018677	0.7827403
sp|Q9NPY3|C1QR1_HUMAN	-0.5997353	1.1932944
sp|P01111|RASN_HUMAN	-0.5851975	0.656254
sp|Q7L2H7|EIF3M_HUMAN	-0.574894	0.656254
sp|Q9NVJ2|ARL8B_HUMAN	-0.5640602	1.1932944
sp|Q9H2U2-3|IPYR2_HUMAN	-0.5592861	0.656254
sp|P06703|S10A6_HUMAN	-0.5528927	1.1932944
sp|P51606-2|RENBP_HUMAN	-0.5432911	1.1932944
sp|P20292|AL5AP_HUMAN	-0.5374985	0.7061832
sp|Q9NPJ3|ACO13_HUMAN	-0.5284443	1.1932944
sp|Q96BM9|ARL8A_HUMAN	-0.527935	0.656254
sp|Q03135-2|CAV1_HUMAN	-0.5211258	0.656254
sp|P63167|DYL1_HUMAN	-0.5180721	0.656254
sp|P61601|NCALD_HUMAN	-0.5154629	0.656254
sp|O76041-2|NEBL_HUMAN	-0.5078144	0.656254
sp|Q12846|STX4_HUMAN	-0.5061188	0.656254
sp|Q07075|AMPE_HUMAN	-0.5038376	1.0485198
sp|Q9NVD7|PARVA_HUMAN	-0.5024605	0.7827403
sp|Q32MZ4-3|LRRF1_HUMAN	-0.497015	0.7827403
sp|Q14956-2|GPNMB_HUMAN	-0.4906883	1.1932944
sp|Q8TD06|AGR3_HUMAN	-0.4839592	1.1932944
sp|P28676|GRAN_HUMAN	-0.4839382	1.1932944
sp|Q16822|PCKGM_HUMAN	-0.464283	1.1932944
sp|P29972-2|AQP1_HUMAN	-0.463192	1.1932944
sp|P33121-3|ACSL1_HUMAN	-0.4621334	1.0485198
sp|Q9UPQ0|LIMC1_HUMAN	-0.4512787	0.65625405
sp|P10599-2|THIO_HUMAN	-0.4511719	0.6298893
sp|P16403|H12_HUMAN	-0.4468346	0
sp|P30049|ATPD_HUMAN	-0.4359455	1.1932944
sp|Q6P4A8|PLBL1_HUMAN	-0.4328232	0.8983557
sp|Q16836-3|HCDH_HUMAN	-0.4220142	0.2178309
sp|P02462|CO4A1_HUMAN	-0.419445	0.91601294
sp|Q9Y376|CAB39_HUMAN	-0.416811	1.2773042
sp|Q53GQ0|DHB12_HUMAN	-0.4128914	1.1505735
sp|P61916-2|NPC2_HUMAN	-0.4101582	1.1932944
sp|O75323|NIPS2_HUMAN	-0.3890457	1.1932944
sp|P52789|HXK2_HUMAN	-0.3833809	0.656254

sp|O15400-2|STX7_HUMAN	-0.3751507	1.3006523
sp|P05141|ADT2_HUMAN	-0.3730087	0.84879977
sp|P09493-5|TPM1_HUMAN	-0.3656731	1.1932944
sp|Q07812-7|BAX_HUMAN	-0.3610706	1.1932944
sp|Q9ULZ3-2|ASC_HUMAN	-0.3588562	0.7827403
sp|Q9UBR2|CATZ_HUMAN	-0.3559399	1.1932944
sp|Q8N335|GPD1L_HUMAN	-0.3549767	0.5204253
sp|O60504-2|VINEX_HUMAN	-0.3538971	1.1791906
sp|Q6DN03|H2B2C_HUMAN	-0.3506718	1.1932944
sp|Q7Z7H5-3|TMED4_HUMAN	-0.3497849	1.1932944
sp|Q13283|G3BP1_HUMAN	-0.3474045	0.6070219
sp|P62834|RAP1A_HUMAN	-0.3465195	0.656254
sp|Q8N684-3|CPSF7_HUMAN	-0.3429146	1.1932944
sp|P57088|TMM33_HUMAN	-0.3418636	1.1932944
sp|P11717|MPRI_HUMAN	-0.335989	1.1932944
sp|Q13938-4|CAYP1_HUMAN	-0.334919	0
sp|P45954-2|ACDSB_HUMAN	-0.3328743	1.1932944
sp|P11166|GTR1_HUMAN	-0.3292885	1.1932944
sp|Q92930|RAB8B_HUMAN	-0.3292084	0.656254
sp|P06396-2|GELS_HUMAN	-0.3284798	0.656254
sp|O15296|LX15B_HUMAN	-0.3269501	1.2095301
sp|P00740|FA9_HUMAN	-0.3240032	1.1932944
sp|P06702|S10A9_HUMAN	-0.3234787	1.2805575
sp|P62745|RHOB_HUMAN	-0.3185463	0.656254
sp|P02794|FRIH_HUMAN	-0.3166008	1.3006523
sp|Q9UKK9|NUDT5_HUMAN	-0.3162594	1.1932944
sp|P0DPI2-2|GAL3A_HUMAN	-0.3143559	0.7061832
sp|Q9Y394-2|DHRS7_HUMAN	-0.3138447	1.0485198
sp|Q9NNW7|TRXR2_HUMAN	-0.3117695	0.6298893
sp|P22307-8|NLTP_HUMAN	-0.3102665	0.7061832
sp|Q92522|H1X_HUMAN	-0.3070889	0.45033538
sp|P29992|GNA11_HUMAN	-0.3059158	1.1932944
sp|P61956-2|SUMO2_HUMAN	-0.3058548	0.656254
sp|O95833|CLIC3_HUMAN	-0.2999783	0.7061832
sp|Q86Y82|STX12_HUMAN	-0.2991276	1.3006523
sp|P69891|HBG1_HUMAN	-0.2987309	0.656254
sp|P07954-2|FUMH_HUMAN	-0.2981339	1.0634323
sp|O00483|NDUA4_HUMAN	-0.2944012	1.1932944
sp|P78417-3|GSTO1_HUMAN	-0.2861843	0.7827403
sp|Q9NUB1-2|ACS2L_HUMAN	-0.2843056	0.7827403
sp|O00186|STXB3_HUMAN	-0.2841625	0.45033538
sp|Q8NBX0|SCPDL_HUMAN	-0.2840042	1.2773042
sp|P63096|GNAI1_HUMAN	-0.2818565	1.1505735
sp|P09601|HMOX1_HUMAN	-0.277462	0.7588735
sp|Q15056-2|IF4H_HUMAN	-0.2769585	0.7827403

sp|P62070-4|RRAS2_HUMAN	-0.2692165	0.656254
sp|P15559-2|NQO1_HUMAN	-0.266222	1.1932944
sp|Q9NZK5|ADA2_HUMAN	-0.2661934	0.7827403
sp|P51636-2|CAV2_HUMAN	-0.2661018	1.1932944
sp|P10619-2|PPGB_HUMAN	-0.2650528	0.6070219
sp|P06753-2|TPM3_HUMAN	-0.2645645	1.1932944
sp|P07948-2|LYN_HUMAN	-0.2641106	1.1932944
sp|P25685-2|DNJB1_HUMAN	-0.2640648	1.1932944
sp|Q13813-3|SPTN1_HUMAN	-0.2621231	0.7827403
sp|Q16787-3|LAMA3_HUMAN	-0.2618942	0.656254
sp|O95197-2|RTN3_HUMAN	-0.2593765	1.1932944
sp|P11279|LAMP1_HUMAN	-0.2590313	0.45033538
sp|P10412|H14_HUMAN	-0.2583695	0
sp|P06239-3|LCK_HUMAN	-0.2564507	0.656254
sp|P26368-2|U2AF2_HUMAN	-0.2558193	1.1932944
sp|P12236|ADT3_HUMAN	-0.2554035	1.1932944
sp|Q06136|KDSR_HUMAN	-0.2553501	1.1932944
sp|P31947|1433S_HUMAN	-0.2546768	1.2095301
sp|Q9Y5S9-2|RBM8A_HUMAN	-0.2542372	0.2178309
sp|P61586|RHOA_HUMAN	-0.2525559	1.1932944
sp|P02749|APOH_HUMAN	-0.2522049	0.23973103
sp|P29400-2|CO4A5_HUMAN	-0.2473364	0.656254
sp|Q92542|NICA_HUMAN	-0.2469273	1.1505735
sp|Q9NP72|RAB18_HUMAN	-0.2454052	1.0485198
sp|P14866-2|HNRPL_HUMAN	-0.2419987	1.1505735
sp|Q92769|HDAC2_HUMAN	-0.2412472	0.656254
sp|P48059-3|LIMS1_HUMAN	-0.2395668	0.656254
sp|P15153|RAC2_HUMAN	-0.2393036	0.656254
sp|P11234-2|RALB_HUMAN	-0.2353172	0.656254
sp|Q13884|SNTB1_HUMAN	-0.2346668	0.19149946
sp|P62937|PPIA_HUMAN	-0.2326107	0.7061832
sp|O00712-4|NFIB_HUMAN	-0.2323837	0.656254
sp|Q02978-2|M2OM_HUMAN	-0.2321529	0.7061832
sp|P63092-3|GNAS2_HUMAN	-0.2309876	1.0634323
sp|P08631-2|HCK_HUMAN	-0.2304287	0.656254
sp|P0DJI8|SAA1_HUMAN	-0.2288914	0.45033538
sp|Q08431|MFGM_HUMAN	-0.2264481	0.91601294
sp|Q7Z4W1|DCXR_HUMAN	-0.224781	1.2941489
sp|P08962-2|CD63_HUMAN	-0.2241116	1.1932944
sp|P30838|AL3A1_HUMAN	-0.2211876	1.1932944
sp|O60271-4|JIP4_HUMAN	-0.2198963	0.35795313
sp|Q53T59|H1BP3_HUMAN	-0.2198544	1.1932944
sp|P12235|ADT1_HUMAN	-0.2194901	0.656254
sp|P30626-2|SORCN_HUMAN	-0.2190437	0.9968222
sp|P60953|CDC42_HUMAN	-0.217598	1.214352

sp|P02746|C1QB_HUMAN	-0.2163715	0.19149946
sp|O76003|GLRX3_HUMAN	-0.2138844	0.656254
sp|P51153|RAB13_HUMAN	-0.213274	0.656254
sp|P21964-2|COMT_HUMAN	-0.2127342	0.7759099
sp|P09429|HMGB1_HUMAN	-0.2124062	1.1932944
sp|Q6IAA8|LTOR1_HUMAN	-0.211792	1.1932944
sp|P52788-2|SPSY_HUMAN	-0.2112789	0.7827403
sp|P29350-3|PTN6_HUMAN	-0.2065773	1.3006523
sp|Q96RQ3|MCCA_HUMAN	-0.2057762	0.6298893
sp|Q9BZF9-2|UACA_HUMAN	-0.2011404	0.19149946
sp|P09417-2|DHPR_HUMAN	-0.201004	0.19149946
sp|Q9NYL9|TMOD3_HUMAN	-0.1977615	0.30372584
sp|Q9UGI8-2|TES_HUMAN	-0.1959992	1.2954973
sp|Q9Y3D6|FIS1_HUMAN	-0.1959915	0.7061832
sp|O15511|ARPC5_HUMAN	-0.1945438	0.40256184
sp|Q9H8H3|MET7A_HUMAN	-0.19454	0.2178309
sp|P15311|EZRI_HUMAN	-0.1936665	0.3664326
sp|O15143|ARC1B_HUMAN	-0.192049	1.0076748
sp|P61006|RAB8A_HUMAN	-0.1918068	1.1932944
sp|Q00325-2|MPCP_HUMAN	-0.1916618	1.0801278
sp|Q9BW30|TPPP3_HUMAN	-0.1900234	0.7217418
sp|Q6NY19-2|KANK3_HUMAN	-0.1893435	1.1932944
sp|O00391|QSOX1_HUMAN	-0.1877213	1.1932944
sp|P35754|GLRX1_HUMAN	-0.1856155	0.7498006
sp|P04222|1C03_HUMAN	-0.1854057	0
sp|Q86TX2|ACOT1_HUMAN	-0.1840019	0.656254
sp|P28482|MK01_HUMAN	-0.1826344	1.1791906
sp|P42167|LAP2B_HUMAN	-0.1817761	1.1932944
sp|P51991-2|ROA3_HUMAN	-0.1808529	0.656254
sp|P68371|TBB4B_HUMAN	-0.1786995	0
sp|P63220|RS21_HUMAN	-0.1783333	1.1932944
sp|Q14019|COTL1_HUMAN	-0.1780624	1.3006523
sp|Q10713|MPPA_HUMAN	-0.1779327	0.48520416
sp|P13987-2|CD59_HUMAN	-0.1771298	0.35795313
sp|Q92688-2|AN32B_HUMAN	-0.1770287	1.1505735
sp|P62263|RS14_HUMAN	-0.1758843	0.7061832
sp|P51649-2|SSDH_HUMAN	-0.1735516	1.0485198
sp|Q15185-3|TEBP_HUMAN	-0.1718864	0.45033538
sp|P53597|SUCA_HUMAN	-0.1715469	0.7061832
sp|Q9UBV8|PEF1_HUMAN	-0.1676807	0.7061832
sp|P35270|SPRE_HUMAN	-0.1676788	0.7498006
sp|P04839|CY24B_HUMAN	-0.1676426	0.7827403
sp|P58107|EPIPL_HUMAN	-0.1673594	0.98032326
sp|Q9HC35-2|EMAL4_HUMAN	-0.1654797	0.84879977
sp|P62942|FKB1A_HUMAN	-0.1652355	0.656254

sp|Q9UDY2-3|ZO2_HUMAN	-0.163496	1.1932944
sp|P31946-2|1433B_HUMAN	-0.1625366	0.656254
sp|P30566|PUR8_HUMAN	-0.1617203	0.656254
sp|Q03154-4|ACY1_HUMAN	-0.1615448	0.7061832
sp|P63000-2|RAC1_HUMAN	-0.161396	0.35795313
sp|Q15833-2|STXB2_HUMAN	-0.1613522	0.19149946
sp|P07305-2|H10_HUMAN	-0.1610603	0.1558116
sp|Q96EP5-2|DAZP1_HUMAN	-0.1608219	0.45033538
sp|P08559-2|ODPA_HUMAN	-0.1605473	0.5886828
sp|O75608-2|LYPA1_HUMAN	-0.1600056	1.1932944
sp|P62879|GBB2_HUMAN	-0.1586113	0.40256184
sp|P42126-2|ECI1_HUMAN	-0.1578217	1.1932944
sp|Q86U42-2|PABP2_HUMAN	-0.1578064	0.656254
sp|P35908|K22E_HUMAN	-0.1570816	0.7740364
sp|Q15181|IPYR_HUMAN	-0.1569786	0.30372584
sp|P04217|A1BG_HUMAN	-0.1550922	1.1895121
sp|Q14254|FLOT2_HUMAN	-0.1550598	0.7217418
sp|P09651-3|ROA1_HUMAN	-0.1545811	0.80804527
sp|P09211|GSTP1_HUMAN	-0.1543999	0.08684197
sp|P29466-2|CASP1_HUMAN	-0.152626	0.656254
sp|P63261|ACTG_HUMAN	-0.1507282	0
sp|Q9P0L0-2|VAPA_HUMAN	-0.1500397	1.214352
sp|P61204|ARF3_HUMAN	-0.1494961	1.0634323
sp|Q16630-2|CPSF6_HUMAN	-0.1469078	0.2178309
sp|P01920|DQB1_HUMAN	-0.145792	0.7827403
sp|Q9UMS4|PRP19_HUMAN	-0.14571	1.1240381
sp|P60983|GMFB_HUMAN	-0.1456051	1.1505735
sp|Q86W92-2|LIPB1_HUMAN	-0.1451817	0.45033538
sp|Q969X5-2|ERGI1_HUMAN	-0.1436367	0.91601294
sp|P53004|BIEA_HUMAN	-0.1435127	0.70235044
sp|P24539|AT5F1_HUMAN	-0.1435108	0.3852666
sp|P78417-2|GSTO1_HUMAN	-0.143261	0.656254
sp|P19367-2|HXK1_HUMAN	-0.1431255	0.68352973
sp|Q9P2R7-2|SUCB1_HUMAN	-0.1426697	1.1240381
sp|P49593-2|PPM1F_HUMAN	-0.142581	1.0485198
sp|P06865|HEXA_HUMAN	-0.1420288	0.43633315
sp|P58546|MTPN_HUMAN	-0.1418476	0.5204253
sp|O15145|ARPC3_HUMAN	-0.1415844	1.1505735
sp|Q9UQ80|PA2G4_HUMAN	-0.1398811	0
sp|P07203|GPX1_HUMAN	-0.1395035	0.2591514
sp|P52272-2|HNRPM_HUMAN	-0.1391411	0.92609
sp|O95716|RAB3D_HUMAN	-0.1389542	0.656254
sp|P06733|ENOA_HUMAN	-0.1366272	0.79965526
sp|Q27J81-2|INF2_HUMAN	-0.1364479	0.04707252
sp|P62826|RAN_HUMAN	-0.1359272	0.7957244

sp|P54727|RD23B_HUMAN	-0.1358814	1.1505735
sp|P62906|RL10A_HUMAN	-0.1355286	1.0370445
sp|P02751-15|FINC_HUMAN	-0.1352348	0.656254
sp|Q9NX63|MIC19_HUMAN	-0.1345177	0.45033538
sp|P63010-2|AP2B1_HUMAN	-0.1341934	0.7957244
sp|O15127|SCAM2_HUMAN	-0.1339932	0.7061832
sp|P37802|TAGL2_HUMAN	-0.1325092	0.7604814
sp|Q9UBQ5|EIF3K_HUMAN	-0.1314697	0.45033538
sp|P07360|CO8G_HUMAN	-0.1306877	0.7827403
sp|Q9BWD1|THIC_HUMAN	-0.1304359	0.45033538
sp|P48681|NEST_HUMAN	-0.1303425	0.9943782
sp|P30466|1B18_HUMAN	-0.1303062	0.656254
sp|P33316|DUT_HUMAN	-0.1302338	1.1932944
sp|P53007|TXTP_HUMAN	-0.1298752	0.84879977
sp|P36542-2|ATPG_HUMAN	-0.1291056	0
sp|P04424-2|ARLY_HUMAN	-0.1289539	0.68888646
sp|O94811|TPPP_HUMAN	-0.1277542	0.45033538
sp|P46108|CRK_HUMAN	-0.1268578	0.9533461
sp|P61758|PFD3_HUMAN	-0.1266956	0.7827403
sp|P52790|HXK3_HUMAN	-0.1261158	0.48520416
sp|Q9NZL9|MAT2B_HUMAN	-0.1241074	1.1276597
sp|P61088|UBE2N_HUMAN	-0.1229248	0.19149946
sp|Q9H0W9-2|CK054_HUMAN	-0.1227112	0.90036625
sp|P16157-21|ANK1_HUMAN	-0.1226025	1.120602
sp|O95994|AGR2_HUMAN	-0.1214485	0.6115017
sp|O00231-2|PSD11_HUMAN	-0.1206017	0.6298893
sp|Q15233|NONO_HUMAN	-0.1199627	1.1054204
sp|Q07955-3|SRSF1_HUMAN	-0.1190777	0.7022581
sp|P98082-2|DAB2_HUMAN	-0.118824	1.1932944
sp|Q04760-2|LGUL_HUMAN	-0.118763	0.35193655
sp|Q9H2G2-2|SLK_HUMAN	-0.1180744	0
sp|O14791-2|APOL1_HUMAN	-0.1177902	1.1932944
sp|Q9HC38|GLOD4_HUMAN	-0.1176682	0.19410844
sp|P08754|GNAI3_HUMAN	-0.1175327	0.21439649
sp|P54577|SYYC_HUMAN	-0.1172314	1.1505735
sp|P42285|MTREX_HUMAN	-0.1170683	0.19149946
sp|P31946|1433B_HUMAN	-0.1170654	0.656254
sp|P52566|GDIR2_HUMAN	-0.1168938	1.0370445
sp|P0C0S5|H2AZ_HUMAN	-0.1168327	0
sp|P13284|GILT_HUMAN	-0.1167755	0.7061832
sp|Q96MM6|HS12B_HUMAN	-0.1164475	0.06986027
sp|P11387|TOP1_HUMAN	-0.1160851	0.19149946
sp|A0A0C4DH31|HV118_HUMAN	-0.1152687	0.656254
sp|P25788-2|PSA3_HUMAN	-0.1143723	1.1791906
sp|Q13451|FKBP5_HUMAN	-0.1140347	1.0076748

sp|P28066|PSA5_HUMAN	-0.1140213	1.1791906
sp|P50570-2|DYN2_HUMAN	-0.1139145	1.1240381
sp|P62304|RUXE_HUMAN	-0.113884	0.45033538
sp|B5ME19|EIFCL_HUMAN	-0.1137829	0.91601294
sp|O60825-2|F262_HUMAN	-0.1137791	0.656254
sp|P15121|ALDR_HUMAN	-0.1134338	0.28377196
sp|P35268|RL22_HUMAN	-0.1128674	0.7061832
sp|O75874|IDHC_HUMAN	-0.1124954	0.694615
sp|Q16629-2|SRSF7_HUMAN	-0.1123314	0.1342476
sp|P0DMV9|HS71B_HUMAN	-0.1119881	0.7687336
sp|Q5JPE7-2|NOMO2_HUMAN	-0.1111984	0.35795313
sp|P53634|CATC_HUMAN	-0.1099701	0.09894868
sp|Q13308-6|PTK7_HUMAN	-0.1095448	1.0485198
sp|Q9UNF0-2|PACN2_HUMAN	-0.1090317	0.84879977
sp|P48509|CD151_HUMAN	-0.1081238	0.45033538
sp|O75436|VP26A_HUMAN	-0.1075745	0.7498006
sp|O75347|TBCA_HUMAN	-0.1070881	0.7061832
sp|P18077|RL35A_HUMAN	-0.1067886	0.40256184
sp|O60749|SNX2_HUMAN	-0.10672	0.8339981
sp|O00299|CLIC1_HUMAN	-0.1056786	0.26737198
sp|Q96HD1|CREL1_HUMAN	-0.1053982	0.45033538
sp|Q15717-2|ELAV1_HUMAN	-0.1041279	0.7061832
sp|Q9NUQ9|FA49B_HUMAN	-0.1032372	0.35795313
sp|Q9UI12-2|VATH_HUMAN	-0.103199	0.84879977
sp|P30519|HMOX2_HUMAN	-0.103035	0.19149946
sp|Q14118|DAG1_HUMAN	-0.1027203	0.09894868
sp|O43681|ASNA_HUMAN	-0.1021805	0.09894868
sp|P61225|RAP2B_HUMAN	-0.1021709	0.7827403
sp|Q92841-3|DDX17_HUMAN	-0.1016274	0.25398457
sp|Q9NZM1-6|MYOF_HUMAN	-0.1002102	0.22836551
sp|Q16881-2|TRXR1_HUMAN	-0.0990295	0.65625405
sp|Q92905|CSN5_HUMAN	-0.0989533	0
sp|Q96HE7|ERO1A_HUMAN	-0.0988007	0.91601294
sp|Q13813-2|SPTN1_HUMAN	-0.0983734	0.19149946
sp|Q13177|PAK2_HUMAN	-0.0980644	0.45033538
sp|Q9BVC6|TM109_HUMAN	-0.0979576	0.09894868
sp|O14950|ML12B_HUMAN	-0.0974922	0.7588735
sp|P23497|SP100_HUMAN	-0.0965347	0.7827403
sp|Q9Y2Q5|LTOR2_HUMAN	-0.0964394	0
sp|O60814|H2B1K_HUMAN	-0.09622	0
sp|P07858|CATB_HUMAN	-0.0958443	0.7827403
sp|P48449-3|ERG7_HUMAN	-0.0957089	0.40256184
sp|Q10567-2|AP1B1_HUMAN	-0.0949135	0.09894868
sp|Q9HAV0|GBB4_HUMAN	-0.0945149	0.35795313
sp|P49411|EFTU_HUMAN	-0.0935936	1.2203159

sp|P02768|ALBU_HUMAN	-0.0935249	0.06678823
sp|P60033|CD81_HUMAN	-0.0935249	0.45033538
sp|P20674|COX5A_HUMAN	-0.0928268	0.35795313
sp|O43747-2|AP1G1_HUMAN	-0.0926857	0.69308156
sp|P50452|SPB8_HUMAN	-0.0926743	0.45033538
sp|P47755|CAZA2_HUMAN	-0.092514	1.0242546
sp|O75947-2|ATP5H_HUMAN	-0.091856	0.7217418
sp|Q96C23|GALM_HUMAN	-0.0918427	0
sp|Q5R3I4|TTC38_HUMAN	-0.0910559	0.45033538
sp|P00403|COX2_HUMAN	-0.0908966	0.30372584
sp|P36776-3|LONM_HUMAN	-0.0906334	1.1791906
sp|Q9H2D6-2|TARA_HUMAN	-0.090229	0.43103603
sp|O75340-2|PDCD6_HUMAN	-0.0898037	0.2178309
sp|Q9UH99-3|SUN2_HUMAN	-0.0896778	0
sp|Q8WUM4|PDC6I_HUMAN	-0.0895557	0.8068939
sp|P49756|RBM25_HUMAN	-0.0893898	0.35795313
sp|P08575-10|PTPRC_HUMAN	-0.0892601	1.3006523
sp|P61020|RAB5B_HUMAN	-0.0889206	0.35795313
sp|P48163-2|MAOX_HUMAN	-0.0887032	0.5204253
sp|O43813|LANC1_HUMAN	-0.0886879	0.35795313
sp|Q13347|EIF3I_HUMAN	-0.0886688	0
sp|O43390-2|HNRPR_HUMAN	-0.088604	0.49383867
sp|Q9NSE4|SYIM_HUMAN	-0.0884571	0.37060758
sp|P61160|ARP2_HUMAN	-0.0884438	0.7827403
sp|Q9BR76|COR1B_HUMAN	-0.088131	0.0846519
sp|P23946|CMA1_HUMAN	-0.0880203	0.33495146
sp|Q99623|PHB2_HUMAN	-0.0877876	0.6262363
sp|Q9BRA2|TXD17_HUMAN	-0.0875015	0.6298893
sp|P29218|IMPA1_HUMAN	-0.0864697	1.0992733
sp|Q9Y6A4|CFA20_HUMAN	-0.0863152	0.19149946
sp|Q96DG6|CMBL_HUMAN	-0.0855045	0.7061832
sp|P30085|KCY_HUMAN	-0.0853329	0.91601294
sp|Q9NZB2-6|F120A_HUMAN	-0.084465	0.6070219
sp|P20618|PSB1_HUMAN	-0.0839424	1.2064548
sp|P63027|VAMP2_HUMAN	-0.0838299	0.19149946
sp|P01112|RASH_HUMAN	-0.083725	0
sp|P42330|AK1C3_HUMAN	-0.0830498	0.68888646
sp|P61353|RL27_HUMAN	-0.0829582	0.7827403
sp|P08621-3|RU17_HUMAN	-0.0827694	0.48520416
sp|Q15393|SF3B3_HUMAN	-0.0826454	0.5832693
sp|Q9UPN3|MACF1_HUMAN	-0.0822964	0.37554002
sp|O75131|CPNE3_HUMAN	-0.0821877	0.46889037
sp|Q99538-2|LGMN_HUMAN	-0.0814867	0.19149946
sp|Q96KP4|CNDP2_HUMAN	-0.0808411	0.9735724
sp|P02753|RET4_HUMAN	-0.0808086	0.5204253

sp|P51991|ROA3_HUMAN	-0.0803766	0.1342476
sp|P52597|HNRPF_HUMAN	-0.0800953	0.06986027
sp|O95571|ETHE1_HUMAN	-0.0794792	0.95332193
sp|Q92888-2|ARHG1_HUMAN	-0.0793495	0.1558116
sp|Q13217|DNJC3_HUMAN	-0.0791626	0.6298893
sp|Q9NZU5-2|LMCD1_HUMAN	-0.0788631	0.32654193
sp|P43652|AFAM_HUMAN	-0.0787449	0.25634286
sp|Q14165|MLEC_HUMAN	-0.0770035	0.91601294
sp|P40763-3|STAT3_HUMAN	-0.0761776	0
sp|P31937|3HIDH_HUMAN	-0.0758228	0.2178309
sp|P02747|C1QC_HUMAN	-0.0753136	0.45033538
sp|P30050|RL12_HUMAN	-0.0750847	0.06291623
sp|Q9UJS0-2|CMC2_HUMAN	-0.0741615	0.19149946
sp|P02774-3|VTDB_HUMAN	-0.0732918	0.98237437
sp|P17612|KAPCA_HUMAN	-0.0730276	0.8983557
sp|Q86UX7-2|URP2_HUMAN	-0.0728245	0.62912476
sp|Q13045-2|FLII_HUMAN	-0.0721798	0.09339783
sp|P29590|PML_HUMAN	-0.07164	0.35795313
sp|P60842|IF4A1_HUMAN	-0.0710335	0.29781067
sp|Q01130-2|SRSF2_HUMAN	-0.0692883	0.35795313
sp|Q06033-2|ITIH3_HUMAN	-0.0692368	0.45033538
sp|E9PAV3|NACAM_HUMAN	-0.0692081	0
sp|P53396-2|ACLY_HUMAN	-0.0689735	0.01343579
sp|P55084|ECHB_HUMAN	-0.0684109	0.5176613
sp|Q99439|CNN2_HUMAN	-0.0682907	0.52598757
sp|Q02818|NUCB1_HUMAN	-0.0679588	1.0848553
sp|P22033|MUTA_HUMAN	-0.0679073	0.09894868
sp|P25789|PSA4_HUMAN	-0.0678654	0.5176613
sp|P61081|UBC12_HUMAN	-0.0677776	0.48520416
sp|O60256-3|KPRB_HUMAN	-0.0675793	0
sp|O95865|DDAH2_HUMAN	-0.0672646	0.4668234
sp|O43615|TIM44_HUMAN	-0.0667868	0.45033538
sp|P23284|PPIB_HUMAN	-0.0663891	0.6255959
sp|Q9H299|SH3L3_HUMAN	-0.0649128	0.7827403
sp|Q13492-2|PICAL_HUMAN	-0.0645027	0.2178309
sp|Q9Y3A5|SBDS_HUMAN	-0.0637817	0.56808305
sp|Q99714|HCD2_HUMAN	-0.0633144	1.1701994
sp|P61163|ACTZ_HUMAN	-0.0629177	0.30372584
sp|P13861|KAP2_HUMAN	-0.0622692	0.45876312
sp|Q96QK1|VPS35_HUMAN	-0.0614777	0.07727676
sp|P02766|TTHY_HUMAN	-0.0605965	0.6298893
sp|P07357|CO8A_HUMAN	-0.0604153	0
sp|P46926|GNPI1_HUMAN	-0.0596008	0.21439649
sp|Q9BT78|CSN4_HUMAN	-0.0594711	0.30372584
sp|Q9NUJ1-3|ABHDA_HUMAN	-0.0594635	0.19149946

sp|P24557-2|THAS_HUMAN	-0.059103	0
sp|P26196|DDX6_HUMAN	-0.0588417	0.09894868
sp|P62314|SMD1_HUMAN	-0.0586605	0
sp|Q9P258|RCC2_HUMAN	-0.0581379	0.7588735
sp|Q13363-2|CTBP1_HUMAN	-0.0580483	0.19149946
sp|P46940|IQGA1_HUMAN	-0.0575256	0.32928494
sp|P62081|RS7_HUMAN	-0.0575161	0.04707252
sp|Q14152|EIF3A_HUMAN	-0.0573874	0.09894868
sp|P46939-2|UTRO_HUMAN	-0.0572643	1.2669417
sp|Q969G5|CAVN3_HUMAN	-0.057045	0.35795313
sp|Q92973-2|TNPO1_HUMAN	-0.0568047	0.5111962
sp|P01019|ANGT_HUMAN	-0.0564232	0.5111962
sp|O43684-2|BUB3_HUMAN	-0.0559635	0.7827403
sp|P63151-2|2ABA_HUMAN	-0.0543289	0.312067
sp|Q9Y383-3|LC7L2_HUMAN	-0.0533829	0.5204253
sp|Q9BSJ8-2|ESYT1_HUMAN	-0.0528889	0.53847194
sp|P25311|ZA2G_HUMAN	-0.0516529	0.17511293
sp|Q16658|FSCN1_HUMAN	-0.0514832	0.6073585
sp|P18085|ARF4_HUMAN	-0.0509453	0.656254
sp|P31150|GDIA_HUMAN	-0.0507813	0.3516469
sp|Q13232|NDK3_HUMAN	-0.0501213	0.21439649
sp|P47756-2|CAPZB_HUMAN	-0.0500183	0.79939914
sp|Q6WCQ1-2|MPRIP_HUMAN	-0.0495777	0.14672586
sp|Q96S97|MYADM_HUMAN	-0.0489998	0
sp|Q96C19|EFHD2_HUMAN	-0.0482101	0.35795313
sp|P19338|NUCL_HUMAN	-0.0481396	0.24485865
sp|Q9UN86-2|G3BP2_HUMAN	-0.0480766	0
sp|P28074|PSB5_HUMAN	-0.0478649	0.4075265
sp|Q9Y3B3|TMED7_HUMAN	-0.0478058	0.19149946
sp|Q16891-2|MIC60_HUMAN	-0.047718	0.12054814
sp|Q9ULV4-3|COR1C_HUMAN	-0.0467587	0.02662771
sp|Q9Y305-4|ACOT9_HUMAN	-0.0466328	0
sp|P61457|PHS_HUMAN	-0.0453148	0.19149946
sp|P27635|RL10_HUMAN	-0.0452824	0
sp|O75915|PRAF3_HUMAN	-0.0452051	0.2178309
sp|P20339-2|RAB5A_HUMAN	-0.0448065	0
sp|O75396|SC22B_HUMAN	-0.044344	0.51870745
sp|P61158|ARP3_HUMAN	-0.0439873	0.4685232
sp|P40306|PSB10_HUMAN	-0.0435944	0.45563722
sp|Q15404|RSU1_HUMAN	-0.0429096	0.656254
sp|O75964|ATP5L_HUMAN	-0.0427208	0.19149946
sp|P61313|RL15_HUMAN	-0.0424709	0
sp|P39687|AN32A_HUMAN	-0.0420475	0.09894868
sp|P05155-2|IC1_HUMAN	-0.0418358	0
sp|P28062|PSB8_HUMAN	-0.0411339	0.03043297

sp|P62495-2|ERF1_HUMAN	-0.0404701	0.09894868
sp|Q9BQE3|TBA1C_HUMAN	-0.040062	0
sp|O60716-14|CTND1_HUMAN	-0.0400391	0.2178309
sp|P25786-2|PSA1_HUMAN	-0.0399895	0.46799397
sp|P16930|FAAA_HUMAN	-0.0399704	0
sp|Q5EBM0|CMPK2_HUMAN	-0.0398598	0
sp|Q13423|NNTM_HUMAN	-0.0397606	0.4075265
sp|P07737|PROF1_HUMAN	-0.0391426	0.43193293
sp|Q92556|ELMO1_HUMAN	-0.0386887	0.06291623
sp|P49458|SRP09_HUMAN	-0.0385628	0
sp|Q15257-2|PTPA_HUMAN	-0.0384178	0.33495146
sp|Q9BTV4|TMM43_HUMAN	-0.0382252	0.14672586
sp|Q99961-3|SH3G1_HUMAN	-0.0380516	0
sp|Q6UW68|TM205_HUMAN	-0.0374908	0.19149946
sp|O75643|U520_HUMAN	-0.0368099	0.28516325
sp|Q9Y224|RTRAF_HUMAN	-0.0366421	0.33495146
sp|P51553-2|IDH3G_HUMAN	-0.0366077	0.312067
sp|Q96CX2|KCD12_HUMAN	-0.0364475	0.25398457
sp|P38919|IF4A3_HUMAN	-0.0350685	0.0846519
sp|Q9P0V9-2|SEP10_HUMAN	-0.0350018	0
sp|P30101|PDIA3_HUMAN	-0.034729	0.33077648
sp|Q13724-2|MOGS_HUMAN	-0.0344543	0.12897664
sp|P30084|ECHM_HUMAN	-0.034339	0.20467198
sp|P09496-2|CLCA_HUMAN	-0.0336075	0.7588735
sp|P09382|LEG1_HUMAN	-0.0329819	0.12026574
sp|P16152|CBR1_HUMAN	-0.0325584	0.07727676
sp|Q9NZ32|ARP10_HUMAN	-0.0322304	0.45033538
sp|Q16539-2|MK14_HUMAN	-0.0308666	0
sp|P60900|PSA6_HUMAN	-0.0307159	0.19102953
sp|P43034|LIS1_HUMAN	-0.0305157	0.08891098
sp|O15173-2|PGRC2_HUMAN	-0.0305138	0.06291623
sp|P36551|HEM6_HUMAN	-0.0302429	0.45033538
sp|O14773|TPP1_HUMAN	-0.0296345	0.06291623
sp|Q15365|PCBP1_HUMAN	-0.0295448	0.19410844
sp|Q9GZP4-2|PITH1_HUMAN	-0.0295048	0
sp|Q96CW1-2|AP2M1_HUMAN	-0.0291405	0.21439649
sp|Q99497|PARK7_HUMAN	-0.0291328	0.52598757
sp|P31321|KAP1_HUMAN	-0.029089	0
sp|P11171-7|41_HUMAN	-0.0288239	0.21439649
sp|P12694|ODBA_HUMAN	-0.0278969	0.09894868
sp|Q08945|SSRP1_HUMAN	-0.0274086	0.20467198
sp|P46781|RS9_HUMAN	-0.0269623	0.1500082
sp|Q15149-3|PLEC_HUMAN	-0.0269165	0
sp|P43121|MUC18_HUMAN	-0.0268841	0.3005443
sp|P40939|ECHA_HUMAN	-0.0267239	0.7192765

sp|P27824-2|CALX_HUMAN	-0.0265045	0.24295025
sp|P23193-2|TCEA1_HUMAN	-0.026474	0.04707252
sp|P04440|DPB1_HUMAN	-0.0262241	0.7827403
sp|Q9Y5K5-2|UCHL5_HUMAN	-0.0260925	0
sp|P60709|ACTB_HUMAN	-0.0258732	0
sp|P00492|HPRT_HUMAN	-0.0251923	0
sp|Q00610-2|CLH1_HUMAN	-0.0249443	0.09291492
sp|P26599-2|PTBP1_HUMAN	-0.0247307	0.1500082
sp|Q12905|ILF2_HUMAN	-0.0246696	0.22977081
sp|P11678|PERE_HUMAN	-0.0245762	0
sp|P55327-3|TPD52_HUMAN	-0.0245457	0
sp|P55265-4|DSRAD_HUMAN	-0.0242767	0.1558116
sp|Q9NQR4|NIT2_HUMAN	-0.0241222	0.02174174
sp|P14314-2|GLU2B_HUMAN	-0.0239639	0.7173901
sp|Q8NBJ5|GT251_HUMAN	-0.0237122	0.09894868
sp|P02511|CRYAB_HUMAN	-0.0237045	0.09894868
sp|Q9BXP5-2|SRRT_HUMAN	-0.0227909	0.06291623
sp|P43490|NAMPT_HUMAN	-0.0225716	0.26099685
sp|Q9UNE7-2|CHIP_HUMAN	-0.0209084	0.19149946
sp|Q9BUJ2-4|HNRL1_HUMAN	-0.0207176	0.49383867
sp|Q12907|LMAN2_HUMAN	-0.0206909	0.19410844
sp|P04632|CPNS1_HUMAN	-0.0202189	0.09851671
sp|Q16851|UGPA_HUMAN	-0.0200768	0.1558116
sp|P15144|AMPN_HUMAN	-0.0198078	0.01653191
sp|P13645|K1C10_HUMAN	-0.0194702	0.04882055
sp|Q969H8|MYDGF_HUMAN	-0.0184135	0.2178309
sp|Q12931-2|TRAP1_HUMAN	-0.01828	0.35795313
sp|Q9Y371-2|SHLB1_HUMAN	-0.0179062	0
sp|O00571-2|DDX3X_HUMAN	-0.0169868	0
sp|Q9Y262-2|EIF3L_HUMAN	-0.0167274	0.45720983
sp|P23368|MAOM_HUMAN	-0.016674	0.14927356
sp|P25398|RS12_HUMAN	-0.0162201	0.1897449
sp|Q9Y3Z3|SAMH1_HUMAN	-0.0151577	0.05898649
sp|Q8N1G4|LRC47_HUMAN	-0.0149994	0.7173901
sp|Q8WXF1|PSPC1_HUMAN	-0.0145931	0.14672586
sp|P55795|HNRH2_HUMAN	-0.0141068	0.25789237
sp|P25705|ATPA_HUMAN	-0.0139198	0.06291623
sp|Q8NDH3|PEPL1_HUMAN	-0.0136032	0.17319627
sp|Q9UNM6-2|PSD13_HUMAN	-0.0126839	0.04454162
sp|P62888|RL30_HUMAN	-0.0126553	0.7061832
sp|Q9Y3I0|RTCB_HUMAN	-0.0121574	0.02662771
sp|P30086|PEBP1_HUMAN	-0.0115852	0.48520416
sp|P33176|KINH_HUMAN	-0.0114784	0.26665917
sp|P12110|CO6A2_HUMAN	-0.011116	0.22630788
sp|P04275|VWF_HUMAN	-0.0104198	0.13193025

sp|Q96JB5-4|CK5P3_HUMAN	-0.0101051	0.09894868
sp|Q6UVK1|CSPG4_HUMAN	-0.0100288	0.20467198
sp|P54886-2|P5CS_HUMAN	-0.009964	0.09894868
sp|O75368|SH3L1_HUMAN	-0.0097332	0.2178309
sp|Q9NRV9|HEBP1_HUMAN	-0.0091591	0
sp|Q969V3-2|NCLN_HUMAN	-0.0087624	0.2178309
sp|Q04323-2|UBXN1_HUMAN	-0.0084553	0
sp|P27918|PROP_HUMAN	-0.0081987	0
sp|O43143|DHX15_HUMAN	-0.0072269	0
sp|P31153|METK2_HUMAN	-0.0071049	0
sp|Q08209-5|PP2BA_HUMAN	-0.0057812	0
sp|O14818|PSA7_HUMAN	-0.0054054	0.30819008
sp|P29401-2|TKT_HUMAN	-0.0053768	0.09297622
sp|Q9NZ01|TECR_HUMAN	-0.0050716	0
sp|P54578-3|UBP14_HUMAN	-0.004612	0.03043297
sp|Q9UL18|AGO1_HUMAN	-0.0045738	0.35795313
sp|P07686|HEXB_HUMAN	-0.0045509	0.17171621
sp|Q9BXS5-2|AP1M1_HUMAN	-0.0043888	0
sp|P03952|KLKB1_HUMAN	-0.0041294	0.30372584
sp|P61970|NTF2_HUMAN	-0.0040646	0.2178309
sp|Q9BRR6-2|ADPGK_HUMAN	-0.0039234	0
sp|P0C0L4|CO4A_HUMAN	-0.0036011	0.19149946
sp|P24666|PPAC_HUMAN	-0.0035439	0
sp|P41091|IF2G_HUMAN	-0.0030613	0
sp|O14936-2|CSKP_HUMAN	-0.0029068	0.33495146
sp|Q04837|SSBP_HUMAN	-0.0023975	0.19149946
sp|P16278-2|BGAL_HUMAN	-0.0019455	0.20467198
sp|Q14204|DYHC1_HUMAN	-0.0013695	0.02575658
sp|Q9Y265|RUVB1_HUMAN	-0.0011616	0
sp|P51688|SPHM_HUMAN	-5.74E-04	0.21439649
sp|P10155|RO60_HUMAN	-2.10E-04	0.06842031
sp|Q16401-2|PSMD5_HUMAN	9.59E-04	0.23541966
sp|P31942-2|HNRH3_HUMAN	0.00144959	0.20467198
sp|P50914|RL14_HUMAN	0.00206375	0.2178309
sp|P08134|RHOC_HUMAN	0.00225449	0
sp|Q16543|CDC37_HUMAN	0.00232315	0.2810592
sp|P50579-2|MAP2_HUMAN	0.00288773	0
sp|Q96CN7|ISOC1_HUMAN	0.00303268	0.09339783
sp|Q14764|MVP_HUMAN	0.00321579	0.3627779
sp|P49189|AL9A1_HUMAN	0.00362396	0.02127174
sp|Q12792-3|TWF1_HUMAN	0.00458717	0
sp|Q8NHV1|GIMA7_HUMAN	0.00492287	0
sp|P28065-2|PSB9_HUMAN	0.0051918	0.04454162
sp|Q14498-2|RBM39_HUMAN	0.00542831	0.20467198
sp|Q16775-2|GLO2_HUMAN	0.007061	0.09894868

sp|Q8N392|RHG18_HUMAN	0.00748062	0.2178309
sp|P02549-2|SPTA1_HUMAN	0.00771713	0.6083561
sp|P47985|UCRI_HUMAN	0.00845909	0.09894868
sp|P14550|AK1A1_HUMAN	0.00852776	0.05908025
sp|P42566|EPS15_HUMAN	0.00860024	0
sp|Q13435|SF3B2_HUMAN	0.00902939	0.312067
sp|Q16527|CSRP2_HUMAN	0.00948715	0.09894868
sp|P62269|RS18_HUMAN	0.00970078	0.09750395
sp|O00764-2|PDXK_HUMAN	0.00989246	0.45720983
sp|Q9UNZ2-5|NSF1C_HUMAN	0.01033783	0.02662771
sp|P51178-2|PLCD1_HUMAN	0.01044655	0
sp|P53041|PPP5_HUMAN	0.0110321	0
sp|Q96AE4-2|FUBP1_HUMAN	0.0111084	0.12054814
sp|Q9UBW8|CSN7A_HUMAN	0.01127434	0.2178309
sp|O15498-2|YKT6_HUMAN	0.01170921	0.45033538
sp|P0CG39|POTEJ_HUMAN	0.01203919	0
sp|O60506-3|HNRPQ_HUMAN	0.01266098	0.06291623
sp|P56537|IF6_HUMAN	0.01311302	0.04454162
sp|Q15691|MARE1_HUMAN	0.013134	0.37060758
sp|Q6UWY5|OLFL1_HUMAN	0.01407623	0.06695005
sp|P22392-2|NDKB_HUMAN	0.01420212	0.04454162
sp|P67809|YBOX1_HUMAN	0.01423645	0
sp|P60891|PRPS1_HUMAN	0.01424599	0
sp|Q9NSK0-5|KLC4_HUMAN	0.01435852	0.656254
sp|P12955|PEPD_HUMAN	0.0148983	0.24097534
sp|P00441|SODC_HUMAN	0.01498222	0.09339783
sp|P49755|TMEDA_HUMAN	0.01505852	0.21439649
sp|P31943|HNRH1_HUMAN	0.01603317	0.316588
sp|Q14980-2|NUMA1_HUMAN	0.01617622	0.32702127
sp|P62136|PP1A_HUMAN	0.01692581	0
sp|Q06323|PSME1_HUMAN	0.01695061	0.37060758
sp|P15529-10|MCP_HUMAN	0.01705933	0
sp|Q9H9B4|SFXN1_HUMAN	0.0171814	0
sp|Q99829|CPNE1_HUMAN	0.01723576	0.1500082
sp|P47897|SYQ_HUMAN	0.01755905	0.20467198
sp|Q16531|DDB1_HUMAN	0.01773071	0.77930474
sp|Q16363-2|LAMA4_HUMAN	0.01823235	0.16339546
sp|P02649|APOE_HUMAN	0.01863289	0.24702984
sp|P84085|ARF5_HUMAN	0.01880837	0
sp|P30153|2AAA_HUMAN	0.01882935	0.11475069
sp|P60228|EIF3E_HUMAN	0.01891136	0.06291623
sp|P07942|LAMB1_HUMAN	0.01927185	0.25200537
sp|Q15661|TRYB1_HUMAN	0.02106857	0.2699497
sp|P31930|QCR1_HUMAN	0.02133751	0.06210651
sp|P12004|PCNA_HUMAN	0.02169037	0.19149946

sp|O43242|PSMD3_HUMAN	0.0224247	0.31759265
sp|Q15366-2|PCBP2_HUMAN	0.02262116	0.2178309
sp|P52306-4|GDS1_HUMAN	0.02323341	0.09894868
sp|P11586|C1TC_HUMAN	0.02326202	0.2178309
sp|Q6DD88|ATLA3_HUMAN	0.02345085	0.24097534
sp|P05455|LA_HUMAN	0.02352524	0.65625405
sp|P46777|RL5_HUMAN	0.02373695	0.0315703
sp|P26885|FKBP2_HUMAN	0.02394104	0.19149946
sp|O60784-3|TOM1_HUMAN	0.02417755	0
sp|Q5TZA2|CROCC_HUMAN	0.02497482	0
sp|P07384|CAN1_HUMAN	0.02507019	0.03677253
sp|P05387|RLA2_HUMAN	0.02529907	0.45033538
sp|P30043|BLVRB_HUMAN	0.02540588	0.1342476
sp|P21810|PGS1_HUMAN	0.02547836	0.21133235
sp|P62714|PP2AB_HUMAN	0.02552414	0
sp|P60866-2|RS20_HUMAN	0.02590942	0.19149946
sp|Q9NQG5|RPR1B_HUMAN	0.02593803	0.35795313
sp|P36873-2|PP1G_HUMAN	0.02647018	0
sp|Q04695|K1C17_HUMAN	0.02650356	0
sp|P11940-2|PABP1_HUMAN	0.02678299	0.35795313
sp|Q9C0C2|TB182_HUMAN	0.02717209	0.4075265
sp|Q1KMD3|HNRL2_HUMAN	0.02827644	0.04904167
sp|Q92696|PGTA_HUMAN	0.02902031	0
sp|P30520|PURA2_HUMAN	0.0293026	0.06986027
sp|P19823|ITIH2_HUMAN	0.03021622	0.83349776
sp|P61018-2|RAB4B_HUMAN	0.03073311	0
sp|O14786|NRP1_HUMAN	0.03074074	0
sp|Q9H4A4|AMPB_HUMAN	0.03086853	0.05473607
sp|Q9BZZ5-5|API5_HUMAN	0.03087044	0.04454162
sp|P68400|CSK21_HUMAN	0.03104019	0
sp|P09619|PGFRB_HUMAN	0.03155899	0.09894868
sp|P00918|CAH2_HUMAN	0.03281021	0.48353976
sp|P02675|FIBB_HUMAN	0.03284454	0.33477
sp|P50453|SPB9_HUMAN	0.03290176	0.13248472
sp|P48637|GSHB_HUMAN	0.03376579	1.0951192
sp|P07900-2|HS90A_HUMAN	0.03393745	0.43756488
sp|P21953|ODBB_HUMAN	0.03399658	0.09894868
sp|P40227|TCPZ_HUMAN	0.03437233	0.4490988
sp|P36871|PGM1_HUMAN	0.03442192	0
sp|P61026|RAB10_HUMAN	0.03585625	0.45033538
sp|P46063|RECQ1_HUMAN	0.03608894	0
sp|P53602|MVD1_HUMAN	0.03680992	0.09894868
sp|P63244|RACK1_HUMAN	0.03735733	0.3731982
sp|Q71UM5|RS27L_HUMAN	0.03779221	0
sp|Q9P2X0-2|DPM3_HUMAN	0.03816986	0.19149946

sp|P48739|PIPNB_HUMAN	0.03841019	0
sp|O00232|PSD12_HUMAN	0.03842545	0.25789237
sp|P55010|IF5_HUMAN	0.03859711	0.09750395
sp|O95394-3|AGM1_HUMAN	0.03868866	0
sp|Q14914-2|PTGR1_HUMAN	0.0394783	0
sp|Q9UBQ7|GRHPR_HUMAN	0.03952789	0.45563722
sp|P02730|B3AT_HUMAN	0.03960419	0.2131149
sp|P13928|ANXA8_HUMAN	0.03993988	0.19149946
sp|P01833|PIGR_HUMAN	0.04096603	0.35520625
sp|O94905|ERLN2_HUMAN	0.04128647	0.312067
sp|P18124|RL7_HUMAN	0.04191589	0.56808305
sp|P25787|PSA2_HUMAN	0.04196549	0.93112767
sp|Q14108|SCRB2_HUMAN	0.04209709	0.19149946
sp|O95336|6PGL_HUMAN	0.04227829	0.95332193
sp|P32455|GBP1_HUMAN	0.04247284	0.14672586
sp|P11142|HSP7C_HUMAN	0.04288292	0.7263896
sp|O15372|EIF3H_HUMAN	0.04302788	0.09894868
sp|Q9NYF8-2|BCLF1_HUMAN	0.04328346	0.312067
sp|P15170-2|ERF3A_HUMAN	0.04387665	0
sp|Q96BW5-2|PTER_HUMAN	0.04388046	0
sp|O00233-2|PSMD9_HUMAN	0.04449272	0.19149946
sp|P55209-2|NP1L1_HUMAN	0.04484177	0
sp|O00429-6|DNM1L_HUMAN	0.0450058	0.06986027
sp|P20591|MX1_HUMAN	0.04503632	0.26737198
sp|Q02790|FKBP4_HUMAN	0.04589272	0.21439649
sp|P00736|C1R_HUMAN	0.04620361	0.35795313
sp|Q15637-2|SF01_HUMAN	0.04620743	0.19149946
sp|P49721|PSB2_HUMAN	0.04671669	0.19149946
sp|P54652|HSP72_HUMAN	0.04735756	0.2178309
sp|Q08211|DHX9_HUMAN	0.04820442	0.3703328
sp|Q99460|PSMD1_HUMAN	0.04829216	0.3761346
sp|P61201-2|CSN2_HUMAN	0.04847908	0.19149946
sp|Q13557-12|KCC2D_HUMAN	0.04870415	0.45720983
sp|P61224|RAP1B_HUMAN	0.04888725	0
sp|P62424|RL7A_HUMAN	0.04949188	0.14672586
sp|Q09028-3|RBBP4_HUMAN	0.05097199	0.656254
sp|P05165-2|PCCA_HUMAN	0.05122757	0.4065472
sp|P61978-3|HNRPK_HUMAN	0.05140877	0.79155684
sp|O94973-2|AP2A2_HUMAN	0.05221558	0.2699497
sp|O43252|PAPS1_HUMAN	0.05237579	0.28516325
sp|P23229-4|ITA6_HUMAN	0.05253029	0.45033538
sp|P52907|CAZA1_HUMAN	0.05271339	0.18414244
sp|O60701-2|UGDH_HUMAN	0.05283356	0
sp|Q15005|SPCS2_HUMAN	0.05302048	0.7061832
sp|O60664-4|PLIN3_HUMAN	0.0531807	0.14672586

sp|Q08380|LG3BP_HUMAN	0.05326462	0.55060285
sp|Q15417|CNN3_HUMAN	0.05327416	0.2178309
sp|O60884|DNJA2_HUMAN	0.05330086	0.40256184
sp|P12081-4|SYHC_HUMAN	0.0540123	0.1558116
sp|Q13200|PSMD2_HUMAN	0.05428886	0.09894868
sp|P46976-2|GLYG_HUMAN	0.05477715	0.33495146
sp|Q9Y3F4-2|STRAP_HUMAN	0.05480003	0.5204253
sp|Q7Z4H8|PLGT3_HUMAN	0.05485916	0
sp|Q9UJU6-2|DBNL_HUMAN	0.05522919	0.06291623
sp|P24534|EF1B_HUMAN	0.05677986	0.19149946
sp|Q14651|PLSI_HUMAN	0.05678368	0
sp|P67936|TPM4_HUMAN	0.05710793	0.47741386
sp|Q8WXX5|DNJC9_HUMAN	0.05849457	0.19149946
sp|O95861-4|BPNT1_HUMAN	0.05903435	0
sp|Q16774|KGUA_HUMAN	0.0590477	0
sp|Q8N163-2|CCAR2_HUMAN	0.05933476	0.5204253
sp|O14980|XPO1_HUMAN	0.05957794	0.09894868
sp|P31040|SDHA_HUMAN	0.05993462	0.2453838
sp|P31146|COR1A_HUMAN	0.06005478	1.2873641
sp|P02679-2|FIBG_HUMAN	0.06007004	0.24452867
sp|P55263|ADK_HUMAN	0.06012535	0.06291623
sp|P26641|EF1G_HUMAN	0.0604744	0.21971017
sp|P34897-2|GLYM_HUMAN	0.06052971	0.37061578
sp|Q9BTE1|DCTN5_HUMAN	0.06085968	0
sp|P54136|SYRC_HUMAN	0.0609169	0.02174174
sp|Q9BVK6|TMED9_HUMAN	0.06093979	0.2178309
sp|P29144|TPP2_HUMAN	0.06097603	0.43633315
sp|Q6P2Q9|PRP8_HUMAN	0.06131554	0.09894868
sp|P30044-2|PRDX5_HUMAN	0.06144714	0.656254
sp|O15031|PLXB2_HUMAN	0.06144905	1.0644729
sp|Q7L5N1|CSN6_HUMAN	0.06279469	0.19149946
sp|P02765|FETUA_HUMAN	0.06394196	0.7740364
sp|P62195|PRS8_HUMAN	0.06417847	0
sp|O95782-2|AP2A1_HUMAN	0.06424332	0.7498006
sp|P27694|RFA1_HUMAN	0.06432533	0.06842031
sp|Q9UL46|PSME2_HUMAN	0.06471443	0.7498006
sp|P05388|RLA0_HUMAN	0.06477547	0.7061832
sp|P35542|SAA4_HUMAN	0.06480789	0.19149946
sp|P35998|PRS7_HUMAN	0.06542397	0.22977081
sp|P01034|CYTC_HUMAN	0.06632996	0.45033538
sp|P25325-2|THTM_HUMAN	0.06658745	0.1342476
sp|Q9Y230|RUVB2_HUMAN	0.06678772	0.00519248
sp|P23142-4|FBLN1_HUMAN	0.06681442	0
sp|P49354-2|FNTA_HUMAN	0.06683922	0
sp|P50395|GDIB_HUMAN	0.06686783	0.01269578

sp|P02671|FIBA_HUMAN	0.06690407	1.1204947
sp|Q12906-4|ILF3_HUMAN	0.06692314	0.9380495
sp|P50990|TCPQ_HUMAN	0.06718254	0.90534914
sp|Q99426|TBCB_HUMAN	0.06762886	0.5204253
sp|P48643|TCPE_HUMAN	0.06800652	0.9609878
sp|Q9ULA0|DNPEP_HUMAN	0.06804085	0.9847622
sp|Q8NBF2-2|NHLC2_HUMAN	0.0697155	0
sp|Q562R1|ACTBL_HUMAN	0.06974983	0
sp|P05452|TETN_HUMAN	0.07024002	0.5204253
sp|P00558|PGK1_HUMAN	0.07025528	0
sp|Q8WVM8|SCFD1_HUMAN	0.07125282	0.37060758
sp|P27348|1433T_HUMAN	0.07145309	0.6452372
sp|Q13630|FCL_HUMAN	0.07267761	0.19149946
sp|Q9UHL4|DPP2_HUMAN	0.07271195	1.2805575
sp|Q15323|K1H1_HUMAN	0.07345963	0.656254
sp|O60313-10|OPA1_HUMAN	0.07358551	0.19149946
sp|P55884-2|EIF3B_HUMAN	0.07394028	0.33495146
sp|Q14515-2|SPRL1_HUMAN	0.07395744	0
sp|Q709C8-3|VP13C_HUMAN	0.07405853	0.6167118
sp|P19827|ITIH1_HUMAN	0.07424164	0.8654178
sp|Q13418|ILK_HUMAN	0.07429123	0
sp|Q14240-2|IF4A2_HUMAN	0.07466984	0.30372584
sp|O75923-11|DYSF_HUMAN	0.07471657	0.61034113
sp|P68366-2|TBA4A_HUMAN	0.07545471	0
sp|P14923|PLAK_HUMAN	0.07551384	0.35193655
sp|P51692|STA5B_HUMAN	0.07562161	0.19149946
sp|Q9NP79|VTA1_HUMAN	0.07610321	0.2178309
sp|O43488|ARK72_HUMAN	0.07673264	0.1342476
sp|Q9UK22|FBX2_HUMAN	0.07680321	0
sp|P34896-2|GLYC_HUMAN	0.07711315	0
sp|Q12797-10|ASPH_HUMAN	0.07777023	0.7220187
sp|O00487|PSDE_HUMAN	0.0782547	0
sp|Q99832|TCPH_HUMAN	0.07941818	0.54838234
sp|P05156|CFAI_HUMAN	0.07979584	1.3006523
sp|P00734|THRB_HUMAN	0.08078957	0.97180504
sp|P23588|IF4B_HUMAN	0.08080101	0.97209185
sp|Q9UNH7-2|SNX6_HUMAN	0.08080292	0
sp|Q86WV6|STING_HUMAN	0.08148384	0.35795313
sp|A0FGR8-2|ESYT2_HUMAN	0.08227348	0
sp|P14618|KPYM_HUMAN	0.08251953	0.04454162
sp|P00966|ASSY_HUMAN	0.08257484	0.29066643
sp|Q14203-3|DCTN1_HUMAN	0.08350754	0.7217418
sp|P50502|F10A1_HUMAN	0.08363724	0.6791336
sp|Q9P2J5-2|SYLC_HUMAN	0.08431339	0.19149946
sp|P23634-8|AT2B4_HUMAN	0.08445931	0.47871065

sp|Q13425|SNTB2_HUMAN	0.08481598	0.09750395
sp|P62244|RS15A_HUMAN	0.08587265	1.1932944
sp|Q96FV2-2|SCRN2_HUMAN	0.08617592	0.19149946
sp|Q9Y2X3|NOP58_HUMAN	0.08624649	0.70235044
sp|P17980|PRS6A_HUMAN	0.08662415	0.31759265
sp|Q9Y6W5|WASF2_HUMAN	0.08720779	0.2178309
sp|P63104|1433Z_HUMAN	0.08720779	1.1437052
sp|Q99873-3|ANM1_HUMAN	0.0873661	0.33495146
sp|Q6DKJ4|NXN_HUMAN	0.08885384	0.45033538
sp|O75937|DNJC8_HUMAN	0.08929825	0.1342476
sp|Q99436|PSB7_HUMAN	0.08984757	0.19149946
sp|O95466-2|FMNL1_HUMAN	0.09017658	0
sp|P61981|1433G_HUMAN	0.09052658	1.2954973
sp|P62995-3|TRA2B_HUMAN	0.09158707	0.45033538
sp|P11217|PYGM_HUMAN	0.09164429	0.656254
sp|P68104|EF1A1_HUMAN	0.09178543	0.3852666
sp|P12268|IMDH2_HUMAN	0.09181595	1.1932944
sp|P46459|NSF_HUMAN	0.09199905	0.30372584
sp|O14579|COPE_HUMAN	0.09277535	0.95332193
sp|P18583-10|SON_HUMAN	0.09290505	0.19149946
sp|Q9NUV9|GIMA4_HUMAN	0.09391975	1.1687785
sp|Q9GZT8|NIF3L_HUMAN	0.09394455	1.1932944
sp|Q92945|FUBP2_HUMAN	0.09409332	0.31759265
sp|Q8NF91-4|SYNE1_HUMAN	0.09464455	0.19149946
sp|P15088|CBPA3_HUMAN	0.09506798	0.5111962
sp|O43776|SYNC_HUMAN	0.09523773	0.63371044
sp|P16070-7|CD44_HUMAN	0.09542084	0
sp|Q92598-2|HS105_HUMAN	0.09544563	0.656254
sp|P56134-3|ATPK_HUMAN	0.09576988	0
sp|P15880|RS2_HUMAN	0.09662819	0.40256184
sp|P31939|PUR9_HUMAN	0.09669113	0.4214492
sp|Q13126-2|MTAP_HUMAN	0.09687042	1.0370445
sp|P49720|PSB3_HUMAN	0.09692001	1.1791906
sp|Q9HBL0|TENS1_HUMAN	0.09734154	1.2872459
sp|Q9Y696|CLIC4_HUMAN	0.09803009	0.44572112
sp|Q9Y3A3-3|PHOCN_HUMAN	0.09875488	0.45033538
sp|Q32P44|EMAL3_HUMAN	0.0996666	0.35795313
sp|P39019|RS19_HUMAN	0.1011467	0.2178309
sp|Q8NCW5|NNRE_HUMAN	0.10137367	0.7588735
sp|Q9NQW7-3|XPP1_HUMAN	0.10151482	0.03367973
sp|P61247|RS3A_HUMAN	0.10171509	0.8744098
sp|P62913-2|RL11_HUMAN	0.10208511	0.7827403
sp|Q99798|ACON_HUMAN	0.10246086	1.0902835
sp|P37108|SRP14_HUMAN	0.10256195	0.45033538
sp|O43865|SAHH2_HUMAN	0.10307884	0.09894868

sp|P23083|HV102_HUMAN	0.10418892	0
sp|P07358|CO8B_HUMAN	0.10424233	0.25789237
sp|P09543-2|CN37_HUMAN	0.10435867	0.70235044
sp|O75489|NDUS3_HUMAN	0.10625649	0.6298893
sp|P02042|HBD_HUMAN	0.10668564	0.33495146
sp|P36542|ATPG_HUMAN	0.10767651	0.656254
sp|Q14894|CRYM_HUMAN	0.10821533	1.1932944
sp|Q15008-4|PSMD6_HUMAN	0.10825157	0
sp|P84243|H33_HUMAN	0.10838413	0
sp|A5A3E0|POTEF_HUMAN	0.10857201	0
sp|P46783|RS10_HUMAN	0.10948944	0.19149946
sp|P02652-2|APOA2_HUMAN	0.11044502	0.29066643
sp|Q5K4L6|S27A3_HUMAN	0.11058807	1.0301651
sp|Q9NSD9|SYFB_HUMAN	0.1108036	0.1342476
sp|Q13526|PIN1_HUMAN	0.11107636	0.19149946
sp|P29692-2|EF1D_HUMAN	0.11126709	1.1505735
sp|P18084|ITB5_HUMAN	0.11136246	0.7827403
sp|P00491|PNPH_HUMAN	0.11140442	1.0951192
sp|P55060-3|XPO2_HUMAN	0.11250305	0.79861414
sp|Q9BUT1|BDH2_HUMAN	0.1125412	0.44572112
sp|O95479|G6PE_HUMAN	0.11265183	0.5111962
sp|Q14974|IMB1_HUMAN	0.11315537	1.1551039
sp|Q15029-2|U5S1_HUMAN	0.11359787	0.6828178
sp|P78406|RAE1L_HUMAN	0.11487198	0.312067
sp|O75503|CLN5_HUMAN	0.11552811	0.5204253
sp|P16298-4|PP2BB_HUMAN	0.11629486	0.312067
sp|Q13867|BLMH_HUMAN	0.11675263	0.19149946
sp|P08865|RSSA_HUMAN	0.11711502	0.91072154
sp|P28070|PSB4_HUMAN	0.11728859	1.1687785
sp|P42704|LPPRC_HUMAN	0.11781693	0
sp|Q93034|CUL5_HUMAN	0.11852455	0
sp|Q5JRX3-3|PREP_HUMAN	0.11852646	1.1932944
sp|P30419-2|NMT1_HUMAN	0.11968613	0.656254
sp|Q9NY15|STAB1_HUMAN	0.11970139	0
sp|Q06210-2|GFPT1_HUMAN	0.11972809	0.84879977
sp|Q15121|PEA15_HUMAN	0.11996651	0.7061832
sp|P24821-4|TENA_HUMAN	0.12018585	0.6615227
sp|P55735-2|SEC13_HUMAN	0.12021256	0.8983557
sp|Q15149-9|PLEC_HUMAN	0.12071991	0.656254
sp|P62333|PRS10_HUMAN	0.12212753	0.48520416
sp|P25774|CATS_HUMAN	0.12368584	0.35795313
sp|P31948|STIP1_HUMAN	0.12405586	0
sp|P52888|THOP1_HUMAN	0.12430573	0.45033538
sp|Q15819|UB2V2_HUMAN	0.12491036	0.656254
sp|O00170|AIP_HUMAN	0.12524986	1.2095301

sp|P42224-2|STAT1_HUMAN	0.1257	0.6115017
sp|P21912|SDHB_HUMAN	0.12613869	0.7061832
sp|O14744|ANM5_HUMAN	0.12630844	0.45033538
sp|P22352|GPX3_HUMAN	0.1270523	0.91601294
sp|P07741|APT_HUMAN	0.12751389	0.8059303
sp|Q96AG4|LRC59_HUMAN	0.12767029	1.1791906
sp|P62857|RS28_HUMAN	0.12777138	0.7061832
sp|P06748-2|NPM_HUMAN	0.12779617	0.80804527
sp|Q9Y639-4|NPTN_HUMAN	0.12938786	0.19149946
sp|P31689-2|DNJA1_HUMAN	0.12968254	0.6070219
sp|Q9Y2B0|CNPY2_HUMAN	0.1301918	0.91601294
sp|Q02952-2|AKA12_HUMAN	0.13088608	0.7827403
sp|P19404|NDUV2_HUMAN	0.1311245	1.1932944
sp|Q9UHX1-6|PUF60_HUMAN	0.13143158	0.2178309
sp|O94776|MTA2_HUMAN	0.13183784	1.2941489
sp|Q13464|ROCK1_HUMAN	0.13236618	0.2178309
sp|O43790|KRT86_HUMAN	0.13237	0.656254
sp|P06681-3|CO2_HUMAN	0.13250732	1.1791906
sp|P52758|RIDA_HUMAN	0.13308525	0.7827403
sp|P22102|PUR2_HUMAN	0.13341522	0.21439649
sp|Q5TFE4|NT5D1_HUMAN	0.1351223	0
sp|Q9UKV3-5|ACINU_HUMAN	0.135458	0.8272904
sp|Q12882|DPYD_HUMAN	0.1360817	1.1932944
sp|Q06828|FMOD_HUMAN	0.1363945	0.7827403
sp|Q8TD55|PKHO2_HUMAN	0.13746071	0.7827403
sp|P20810-4|ICAL_HUMAN	0.13751793	0.5204253
sp|P53992|SC24C_HUMAN	0.1377964	0.6298893
sp|Q93009-3|UBP7_HUMAN	0.13781166	1.1932944
sp|Q9UBC2-2|EP15R_HUMAN	0.1389761	0.19149946
sp|Q9NY33|DPP3_HUMAN	0.14040756	0.63371044
sp|Q15437|SC23B_HUMAN	0.14055634	0.656254
sp|Q99733-2|NP1L4_HUMAN	0.14081669	0.65625405
sp|P39023|RL3_HUMAN	0.14105034	0.7588735
sp|Q8NHP8|PLBL2_HUMAN	0.14107323	0.7827403
sp|P30046|DOPD_HUMAN	0.14273834	1.1932944
sp|Q8TAT6-2|NPL4_HUMAN	0.14385319	0.35193655
sp|P51665|PSMD7_HUMAN	0.14427185	0.7827403
sp|P30613-2|KPYR_HUMAN	0.14460182	0.656254
sp|A0A0B4J1X8|HV343_HUMAN	0.1452713	0
sp|Q9Y281|COF2_HUMAN	0.14557648	0.91601294
sp|O43396|TXNL1_HUMAN	0.14881516	0.7827403
sp|Q14697|GANAB_HUMAN	0.15060043	1.1932944
BirA-TRIP6_BirAT6	0.15146828	0.45033538
sp|Q7Z4I7-3|LIMS2_HUMAN	0.15323544	1.1932944
sp|Q9BUF5|TBB6_HUMAN	0.1535492	0.7588735

sp|Q9P2T1-2|GMPR2_HUMAN	0.15413094	0.70235044
sp|Q92499|DDX1_HUMAN	0.15468407	0.74639726
sp|P04066|FUCO_HUMAN	0.15529633	1.1505735
sp|O95302-3|FKBP9_HUMAN	0.15555954	0.45033538
sp|Q96P70|IPO9_HUMAN	0.15565109	1.0485198
sp|P10644|KAP0_HUMAN	0.15668297	0.312067
sp|Q96FN4|CPNE2_HUMAN	0.15696144	0
sp|O75828|CBR3_HUMAN	0.15713882	0.45033538
sp|P35579-2|MYH9_HUMAN	0.15755844	0.656254
sp|Q15274|NADC_HUMAN	0.15886497	0.91601294
sp|Q86VS8|HOOK3_HUMAN	0.15958023	0.7827403
sp|P13693|TCTP_HUMAN	0.16000938	0.45033538
sp|Q96PD5-2|PGRP2_HUMAN	0.1618309	0.8983557
sp|P08185|CBG_HUMAN	0.1627922	1.1932944
sp|P00748|FA12_HUMAN	0.16456223	0.91601294
sp|Q9HB71|CYBP_HUMAN	0.16623497	0.312067
sp|P62249|RS16_HUMAN	0.1674099	1.1505735
sp|Q16134-3|ETFD_HUMAN	0.16784286	0.45033538
sp|Q7L576|CYFP1_HUMAN	0.16806793	0
sp|P49903-2|SPS1_HUMAN	0.16813278	0.91601294
sp|P12111-4|CO6A3_HUMAN	0.16972733	0.656254
sp|P22059|OSBP1_HUMAN	0.17030907	0.45033538
sp|P43686|PRS6B_HUMAN	0.17045021	1.2109947
sp|P60981|DEST_HUMAN	0.17071533	0.7498006
sp|Q9UBE0|SAE1_HUMAN	0.17100525	0.35795313
sp|P00738|HPT_HUMAN	0.17247391	0.656254
sp|O15260-2|SURF4_HUMAN	0.17302513	0.19149946
sp|P21589-2|5NTD_HUMAN	0.17370796	0.7827403
sp|O60610-2|DIAP1_HUMAN	0.17458534	0.30372584
sp|Q8TBC4-2|UBA3_HUMAN	0.17540741	1.1932944
sp|P09972|ALDOC_HUMAN	0.17605019	1.1497186
sp|A0AVT1|UBA6_HUMAN	0.17720222	0.69308156
sp|Q99598|TSNAX_HUMAN	0.17725754	0.5204253
sp|Q96HC4|PDLI5_HUMAN	0.17884064	0.656254
sp|Q9BTE3-2|MCMBP_HUMAN	0.18108082	0.45033538
sp|P01911|2B1F_HUMAN	0.18162155	1.1932944
sp|P62854|RS26_HUMAN	0.18262863	0.7827403
sp|Q7LG56-6|RIR2B_HUMAN	0.18697548	1.1932944
sp|Q9NTK5|OLA1_HUMAN	0.187294	0.45033538
sp|Q13243-3|SRSF5_HUMAN	0.18746376	0.656254
sp|P01892|1A02_HUMAN	0.18762016	0.7061832
sp|Q13976|KGP1_HUMAN	0.18936348	0
sp|Q9BWS9-3|CHID1_HUMAN	0.18950844	0.656254
sp|P52895|AK1C2_HUMAN	0.18975067	0.19149946
sp|P14174|MIF_HUMAN	0.19006538	0.7061832

sp|P0C0L5|CO4B_HUMAN	0.19034195	0.7827403
sp|P08779|K1C16_HUMAN	0.19146633	1.1932944
sp|P63241|IF5A1_HUMAN	0.19202042	0.656254
sp|P23219-2|PGH1_HUMAN	0.19739151	1.2095301
sp|P48741|HSP77_HUMAN	0.20043945	0.656254
sp|P69905|HBA_HUMAN	0.20126534	0.95332193
sp|P09874|PARP1_HUMAN	0.20211601	0.29066643
sp|Q5TDH0-3|DDI2_HUMAN	0.20318222	0.5204253
sp|Q6ZVM7-3|TM1L2_HUMAN	0.20527744	0.45033538
sp|P09871|C1S_HUMAN	0.2063675	0.09894868
sp|Q9H6S3|ES8L2_HUMAN	0.20638752	0.45033538
sp|Q9HD45|TM9S3_HUMAN	0.2080555	0.7827403
sp|Q92629-3|SGCD_HUMAN	0.20808029	1.2095301
sp|Q9BZE9-2|ASPC1_HUMAN	0.21119308	0.656254
sp|P13671|CO6_HUMAN	0.21279335	1.1505735
sp|O94919|ENDD1_HUMAN	0.21400642	0.19149946
sp|P62191-2|PRS4_HUMAN	0.21520424	0.5204253
sp|P01743|HV146_HUMAN	0.21647453	0.656254
sp|O60832|DKC1_HUMAN	0.22153854	1.1932944
sp|Q13885|TBB2A_HUMAN	0.2215538	0
sp|P20039|2B1B_HUMAN	0.22213745	0.656254
sp|Q8NC51-3|PAIRB_HUMAN	0.2227602	1.1932944
sp|P42785-2|PCP_HUMAN	0.22432518	1.2773042
sp|Q9UBS4|DJB11_HUMAN	0.22594261	1.1932944
sp|P06132|DCUP_HUMAN	0.2283001	0.97209185
sp|Q04446|GLGB_HUMAN	0.22938156	0.06291623
sp|Q9UNS2|CSN3_HUMAN	0.23162842	0.656254
sp|Q13642-1|FHL1_HUMAN	0.2323494	0.656254
sp|Q96HN2-2|SAHH3_HUMAN	0.23542023	0.656254
sp|P35637-2|FUS_HUMAN	0.23897076	0.7827403
sp|Q15286|RAB35_HUMAN	0.24458218	0.656254
sp|O75533|SF3B1_HUMAN	0.24580002	1.1932944
sp|Q9NR56-2|MBNL1_HUMAN	0.2474289	0.45033538
sp|Q93084-2|AT2A3_HUMAN	0.24872208	1.1932944
sp|P61923|COPZ1_HUMAN	0.2528267	0.7827403
sp|P07996|TSP1_HUMAN	0.26031017	0.7061832
sp|P14207|FOLR2_HUMAN	0.26111794	0.656254
sp|Q96HY6|DDRGK_HUMAN	0.2616663	0.656254
sp|P62633-3|CNBP_HUMAN	0.2631588	0.7827403
sp|P01782|HV309_HUMAN	0.26563644	0.656254
sp|Q15436|SC23A_HUMAN	0.2750883	1.0485198
sp|P62753|RS6_HUMAN	0.275774	0.7061832
sp|Q9UJZ1-2|STML2_HUMAN	0.2758541	0.7061832
sp|Q96M27-3|PRRC1_HUMAN	0.27661705	1.1932944
sp|Q13310-2|PABP4_HUMAN	0.28466034	0.656254

sp|P67775|PP2AA_HUMAN	0.28541183	0.656254
sp|Q7Z7G0|TARSH_HUMAN	0.28611946	1.1932944
sp|Q03519|TAP2_HUMAN	0.28821945	0.656254
sp|P27695|APEX1_HUMAN	0.2895584	0.45033538
sp|P35080-2|PROF2_HUMAN	0.29071808	0.656254
sp|P0DOX3|IGD_HUMAN	0.2919445	0.656254
sp|P84098|RL19_HUMAN	0.29273224	0.7827403
sp|Q9P1F3|ABRAL_HUMAN	0.2951107	0.656254
sp|P35858-2|ALS_HUMAN	0.29603195	1.1932944
sp|Q8TD19|NEK9_HUMAN	0.29684448	1.1932944
sp|O14617-4|AP3D1_HUMAN	0.2973404	1.1932944
sp|P04844|RPN2_HUMAN	0.30129433	1.0634323
sp|Q13596-2|SNX1_HUMAN	0.30274963	0.656254
sp|P12814|ACTN1_HUMAN	0.3041172	0.656254
sp|Q92747|ARC1A_HUMAN	0.3043518	0.656254
sp|P01591|IGJ_HUMAN	0.3056984	1.1932944
sp|Q08379|GOGA2_HUMAN	0.3082018	1.1932944
sp|P57737-4|CORO7_HUMAN	0.3101902	1.1932944
sp|P61803|DAD1_HUMAN	0.31290817	0.656254
sp|Q06278|AOXA_HUMAN	0.31308937	1.1932944
sp|Q03113|GNA12_HUMAN	0.317461	0.656254
sp|P12814-2|ACTN1_HUMAN	0.31881714	1.1932944
sp|P54802|ANAG_HUMAN	0.3263626	1.1932944
sp|Q14141-2|SEPT6_HUMAN	0.3324852	0.656254
sp|Q9Y5P6-2|GMPPB_HUMAN	0.3346386	1.0301651
sp|Q15046|SYK_HUMAN	0.33969498	0.656254
sp|P55145|MANF_HUMAN	0.34036064	1.1932944
sp|P62910|RL32_HUMAN	0.34047318	1.1932944
sp|P55058|PLTP_HUMAN	0.34212875	1.1932944
sp|Q7L1Q6-2|BZW1_HUMAN	0.3424816	1.2773042
sp|O15067|PUR4_HUMAN	0.3431015	0.19149946
sp|Q92599-3|SEPT8_HUMAN	0.34591866	1.1932944
sp|A0A0C4DH38|HV551_HUMAN	0.3508091	1.1932944
sp|Q86UX2-2|ITIH5_HUMAN	0.3509674	1.1932944
sp|Q66K74-2|MAP1S_HUMAN	0.3526907	1.1932944
sp|O76074-2|PDE5A_HUMAN	0.35488987	1.0301651
sp|Q8TCD5|NT5C_HUMAN	0.36183548	1.1932944
sp|P50135|HNMT_HUMAN	0.36248398	1.1932944
sp|P02533|K1C14_HUMAN	0.36699486	1.1932944
sp|P16104|H2AX_HUMAN	0.38312817	0
sp|P30711|GSTT1_HUMAN	0.38586044	1.1505735
sp|Q9GZM7-3|TINAL_HUMAN	0.3895607	1.1932944
sp|Q96AY3|FKB10_HUMAN	0.39074326	1.1932944
sp|Q9C0B1|FTO_HUMAN	0.39145374	1.1932944
sp|P47895|AL1A3_HUMAN	0.3915739	0.656254

sp|O94788-3|AL1A2_HUMAN	0.39335442	0.656254
sp|P04406|G3P_HUMAN	0.4095478	0.656254
sp|Q01518|CAP1_HUMAN	0.41464996	0.656254
sp|P01780|HV307_HUMAN	0.4202652	0.656254
sp|Q9HCN8|SDF2L_HUMAN	0.4269333	1.1932944
sp|P09012|SNRPA_HUMAN	0.44374275	0.6070219
sp|Q05315|LEG10_HUMAN	0.44470978	1.1932944
sp|P02461|CO3A1_HUMAN	0.4537716	1.1932944
sp|P62736|ACTA_HUMAN	0.45960617	0.656254
sp|P01619|KV320_HUMAN	0.46038246	0.656254
sp|P40123-2|CAP2_HUMAN	0.46378326	0.656254
sp|O14787-2|TNPO2_HUMAN	0.46917534	0.656254
sp|P69892|HBG2_HUMAN	0.46977615	0.656254
sp|P28161|GSTM2_HUMAN	0.4734354	0.656254
sp|P0CG38|POTEI_HUMAN	0.48222923	0.656254
sp|P16144-2|ITB4_HUMAN	0.49498558	1.1932944
sp|Q13247-3|SRSF6_HUMAN	0.49838257	0.45033538
sp|Q96IJ6-2|GMPPA_HUMAN	0.50512123	1.1932944
sp|P62829|RL23_HUMAN	0.50683784	1.1932944
sp|Q15102|PA1B3_HUMAN	0.5094795	1.1505735
sp|P63267|ACTH_HUMAN	0.5139313	0.656254
sp|P46977|STT3A_HUMAN	0.5155163	1.1932944
sp|Q03591|FHR1_HUMAN	0.52656937	1.1932944
sp|P0DOX2|IGA2_HUMAN	0.54795265	0.91601294
sp|O94855-2|SC24D_HUMAN	0.548151	1.1932944
sp|A0A0C4DH29|HV103_HUMAN	0.57790375	0.656254
sp|O94804|STK10_HUMAN	0.6008053	0.7827403
sp|Q9Y4G6|TLN2_HUMAN	0.62131214	0.656254
sp|P32456|GBP2_HUMAN	0.62660027	0.19149946
sp|P06310|KV230_HUMAN	0.6333065	0.656254
sp|P42226|STAT6_HUMAN	0.63524246	0.656254
sp|Q9C0E8-4|LNP_HUMAN	0.6457863	0
sp|P30837|AL1B1_HUMAN	0.6652775	1.1791906
sp|O43294|TGFI1_HUMAN	0.66576195	1.1932944
sp|P04062-2|GLCM_HUMAN	0.6959076	1.1932944
sp|P01624|KV315_HUMAN	0.7047386	1.1932944
sp|Q687X5|STEA4_HUMAN	0.7505169	1.1932944
sp|Q9BX97|PLVAP_HUMAN	0.758543	1.1932944
sp|Q08170|SRSF4_HUMAN	0.8705864	0.656254
sp|Q15063-3|POSTN_HUMAN	0.8774166	1.1932944
sp|Q30154|DRB5_HUMAN	0.9771919	0.656254
sp|Q15063-2|POSTN_HUMAN	1.0057259	0.656254
sp|A0A0C4DH25|KVD20_HUMAN	1.0268784	0.656254
sp|P01834|IGKC_HUMAN	1.1237373	1.1932944
sp|O76011|KRT34_HUMAN	1.1389542	0.656254

sp|P0DP03|HV335_HUMAN	1.1739845	1.1932944
sp|A0A0C4DH41|HV461_HUMAN	1.3298035	0.656254
sp|Q13976-2|KGP1_HUMAN	1.406908	0.656254
sp|A0A075B6P5|KV228_HUMAN	1.4804668	0.656254
