# Supplementary material for: Peptidylarginine deiminase 2 citrullinates MZB1 and promotes the secretion of IgM and IgA
Source: Front Immunol. 2023 Nov 29;14:1290585. doi: 10.3389/fimmu.2023.1290585 (PMC10716219; doi:10.3389/fimmu.2023.1290585)
Supplement: Supplementary file 6 [file DataSheet_6.pdf]

Supplemental Table 6: RA-ILD4 vs controls

Accession #	Fold Change	p value (-log10)
sp|Q15109-10|RAGE_HUMAN	-1.8763103	2.1818786
sp|Q9NZA1-2|CLIC5_HUMAN	-1.4462833	3.5908275
sp|P13760|2B14_HUMAN	-1.4182129	1.6960104
sp|P22748|CAH4_HUMAN	-1.39781	2.6576471
sp|O96009|NAPSA_HUMAN	-1.3222275	2.6576471
sp|P12429|ANXA3_HUMAN	-1.2847366	7.665951
sp|O95810|CAVN2_HUMAN	-1.1478958	3.7803535
sp|P09668|CATH_HUMAN	-1.1342812	4.051346
sp|P62805|H4_HUMAN	-1.1264305	6.3204336
sp|P12821-2|ACE_HUMAN	-1.0942383	2.6576471
sp|P49407-2|ARRB1_HUMAN	-1.0702648	2.6576471
sp|P05362|ICAM1_HUMAN	-1.0369568	5.869831
sp|Q01469|FABP5_HUMAN	-0.9558582	4.051346
sp|Q10589-2|BST2_HUMAN	-0.935112	1.6960104
sp|P50895|BCAM_HUMAN	-0.9252377	6.7698927
sp|Q6NZI2|CAVN1_HUMAN	-0.9243584	6.3204336
sp|P11233|RALA_HUMAN	-0.8924561	2.1818786
sp|Q9UGT4|SUSD2_HUMAN	-0.8921204	4.051346
sp|P51659|DHB4_HUMAN	-0.8718166	12.110734
sp|P21397|AOFA_HUMAN	-0.8706131	5.2991114
sp|P09467|F16P1_HUMAN	-0.8620758	5.417897
sp|Q9Y624|JAM1_HUMAN	-0.8580589	2.6576471
sp|P26447|S10A4_HUMAN	-0.8483505	2.6576471
sp|P61626|LYSC_HUMAN	-0.8436203	2.6576471
sp|P60903|S10AA_HUMAN	-0.8416977	1.6960104
sp|Q8WWI1-5|LMO7_HUMAN	-0.8331699	4.873151
sp|P35241|RADI_HUMAN	-0.8298674	3.5908275
sp|P41218|MNDA_HUMAN	-0.8036041	2.1818786
sp|P59665|DEF1_HUMAN	-0.7842903	2.1818786
sp|Q13510-2|ASAH1_HUMAN	-0.782917	6.3204336
sp|P08473|NEP_HUMAN	-0.7751885	1.6960104
sp|P05164-3|PERM_HUMAN	-0.7705879	10.685196
sp|P56199|ITA1_HUMAN	-0.7598114	6.3204336
sp|Q15599-2|NHRF2_HUMAN	-0.7541599	2.6576471
sp|O43760-2|SNG2_HUMAN	-0.7535286	1.6960104
sp|P02792|FRIL_HUMAN	-0.7494049	3.8337784
sp|Q07157|ZO1_HUMAN	-0.7483292	4.964395
sp|P00167-2|CYB5_HUMAN	-0.747221	2.1818786
sp|P07339|CATD_HUMAN	-0.7462177	8.112776
sp|P01903|DRA_HUMAN	-0.7282181	2.1818786
sp|Q03135|CAV1_HUMAN	-0.726717	2.1818786
sp|Q8NF37|PCAT1_HUMAN	-0.7252331	2.0727112
sp|P16284-3|PECA1_HUMAN	-0.7139416	6.0646477

sp|P55290-4|CAD13_HUMAN	-0.7075768	2.1818786
sp|Q08722-2|CD47_HUMAN	-0.7018814	1.6960104
sp|P05091|ALDH2_HUMAN	-0.6987038	11.597818
sp|P02786|TFR1_HUMAN	-0.6983261	4.051346
sp|Q6YN16|HSDL2_HUMAN	-0.6957808	1.6960104
sp|P05109|S10A8_HUMAN	-0.6911373	3.5908275
sp|P09110|THIK_HUMAN	-0.6760807	2.1818786
sp|P00325|ADH1B_HUMAN	-0.6486321	4.051346
sp|P61106|RAB14_HUMAN	-0.6089516	4.964395
sp|P23381|SYWC_HUMAN	-0.6081715	6.6615443
sp|Q6PIU2-2|NCEH1_HUMAN	-0.6041546	2.9807727
sp|O00159-3|MYO1C_HUMAN	-0.5983868	6.560051
sp|P33121-3|ACSL1_HUMAN	-0.5919914	2.1818786
sp|Q9UHG3|PCYOX_HUMAN	-0.5882683	4.8244376
sp|P06702|S10A9_HUMAN	-0.5822144	3.5908275
sp|P05107|ITB2_HUMAN	-0.5678253	4.509013
sp|Q92817|EVPL_HUMAN	-0.5635586	5.505251
sp|P05556|ITB1_HUMAN	-0.5578499	10.337908
sp|Q9NZN4|EHD2_HUMAN	-0.5495834	8.079533
sp|P04040|CATA_HUMAN	-0.5422745	10.295993
sp|P26440|IVD_HUMAN	-0.5417919	1.6960104
sp|P26038|MOES_HUMAN	-0.5404587	14.55665
sp|Q02318|CP27A_HUMAN	-0.5314999	1.6960104
sp|P51149|RAB7A_HUMAN	-0.5314236	4.964395
sp|P09960|LKHA4_HUMAN	-0.5247154	9.748629
sp|P20160|CAP7_HUMAN	-0.5222836	1.6960104
sp|P09758|TACD2_HUMAN	-0.5166416	2.1818786
sp|P07988|PSPB_HUMAN	-0.5127544	3.5908275
sp|P42765|THIM_HUMAN	-0.5087395	5.5742316
sp|P50225|ST1A1_HUMAN	-0.5047188	1.6960104
sp|Q9H0U4|RAB1B_HUMAN	-0.4991264	1.6960104
sp|P31949|S10AB_HUMAN	-0.4925346	2.6576471
sp|P12111-2|CO6A3_HUMAN	-0.4890022	2.8384566
sp|P20645|MPRD_HUMAN	-0.487114	1.6960104
sp|O60437|PEPL_HUMAN	-0.4855194	14.808462
sp|Q86Y82|STX12_HUMAN	-0.4777937	1.9951487
sp|Q8IV08|PLD3_HUMAN	-0.4744377	1.6960104
sp|O60504-2|VINEX_HUMAN	-0.4704742	2.6576471
sp|Q7Z406|MYH14_HUMAN	-0.467144	9.577684
sp|O15247|CLIC2_HUMAN	-0.4659996	3.1266599
sp|P23193-2|TCEA1_HUMAN	-0.4631729	1.6960104
sp|P04179-4|SODM_HUMAN	-0.4603348	3.1266599
sp|Q14118|DAG1_HUMAN	-0.4579563	1.6960104
sp|Q15907-2|RB11B_HUMAN	-0.456152	3.5908275
sp|Q6YHK3|CD109_HUMAN	-0.4540329	4.509013

sp|O15230|LAMA5_HUMAN	-0.4516983	5.2716713
sp|P40121|CAPG_HUMAN	-0.4441738	4.873151
sp|P05026-2|AT1B1_HUMAN	-0.4392281	2.6576471
sp|P59998|ARPC4_HUMAN	-0.4386444	1.8444856
sp|P08572|CO4A2_HUMAN	-0.4343147	1.867911
sp|Q9NTX5-6|ECHD1_HUMAN	-0.4341583	2.9091687
sp|Q9UL25|RAB21_HUMAN	-0.4336624	2.6576471
sp|P43304|GPDM_HUMAN	-0.4303513	3.5908275
sp|P13284|GILT_HUMAN	-0.4261856	1.6960104
sp|P02769|ALBU_BOVIN	-0.4240131	3.7246199
sp|Q13228-4|SBP1_HUMAN	-0.4237785	11.943951
sp|P16671-4|CD36_HUMAN	-0.4223003	2.1818786
sp|P09601|HMOX1_HUMAN	-0.4217701	1.3815327
sp|P36543-2|VATE1_HUMAN	-0.4207859	1.6960104
sp|P15586-2|GNS_HUMAN	-0.4155407	1.6960104
sp|P11047|LAMC1_HUMAN	-0.4139519	10.176739
sp|P10253|LYAG_HUMAN	-0.4128494	3.1266599
sp|P04080|CYTB_HUMAN	-0.4122162	1.6960104
sp|P49913|CAMP_HUMAN	-0.4119186	1.6960104
sp|Q99536|VAT1_HUMAN	-0.4108467	4.2870226
sp|P17213|BPI_HUMAN	-0.4105015	1.9647322
sp|Q16698-2|DECR_HUMAN	-0.4069099	4.509013
sp|P0DP25|CALM3_HUMAN	-0.406414	3.5908275
sp|P14780|MMP9_HUMAN	-0.4057999	2.768635
sp|P08729|K2C7_HUMAN	-0.4054318	13.799253
sp|P51688|SPHM_HUMAN	-0.4054241	1.8604537
sp|P13987-2|CD59_HUMAN	-0.4048805	1.6960104
sp|P10301|RRAS_HUMAN	-0.4021626	1.6960104
sp|Q99715-4|COCA1_HUMAN	-0.4001656	1.6960104
sp|P14543-2|NID1_HUMAN	-0.3990078	7.665951
sp|P51648-2|AL3A2_HUMAN	-0.3987465	4.051346
sp|Q27J81-2|INF2_HUMAN	-0.3943968	1.4104178
sp|Q9Y3D6|FIS1_HUMAN	-0.3936567	1.6960104
sp|O95340-2|PAPS2_HUMAN	-0.3915939	2.1818786
sp|Q96TC7|RMD3_HUMAN	-0.3905621	1.6960104
sp|Q9UBQ0-2|VPS29_HUMAN	-0.3890362	1.6960104
sp|P49327|FAS_HUMAN	-0.3887196	10.290007
sp|P13797|PLST_HUMAN	-0.3857708	5.856767
sp|O95833|CLIC3_HUMAN	-0.3826351	1.6960104
sp|P62987|RL40_HUMAN	-0.38134	1.8444856
sp|P05141|ADT2_HUMAN	-0.3804932	2.6576471
sp|Q6WCQ1-2|MPRIP_HUMAN	-0.379633	2.7472508
sp|P07355-2|ANXA2_HUMAN	-0.377758	4.628043
sp|P55268|LAMB2_HUMAN	-0.3772316	8.569753
sp|P36957|ODO2_HUMAN	-0.373621	4.4116287

sp|P07910-2|HNRPC_HUMAN	-0.3729038	2.1818786
sp|O75348|VATG1_HUMAN	-0.3720017	1.6960104
sp|P05534|1A24_HUMAN	-0.3719826	1.6960104
sp|Q96TA1-2|NIBL1_HUMAN	-0.3713303	3.103845
sp|P50148|GNAQ_HUMAN	-0.3709412	2.1818786
sp|P27105|STOM_HUMAN	-0.3703537	4.789934
sp|P51148-2|RAB5C_HUMAN	-0.3673286	1.6960104
sp|A6NMZ7|CO6A6_HUMAN	-0.3658981	3.9658737
sp|Q14344|GNA13_HUMAN	-0.364521	2.1818786
sp|P61421|VA0D1_HUMAN	-0.3624477	3.5908275
sp|P13796|PLSL_HUMAN	-0.3598614	11.115791
sp|P13804|ETFA_HUMAN	-0.3589592	4.509013
sp|Q16836-3|HCDH_HUMAN	-0.3562756	1.4104178
sp|Q86VB7-2|C163A_HUMAN	-0.3545952	8.558914
sp|P49593-2|PPM1F_HUMAN	-0.3519402	2.1818786
sp|Q99439|CNN2_HUMAN	-0.3515797	1.6997428
sp|O60234|GMFG_HUMAN	-0.3505001	1.6960104
sp|P30048-2|PRDX3_HUMAN	-0.3480492	2.6576471
sp|P62937|PPIA_HUMAN	-0.3432598	1.6960104
sp|P30040|ERP29_HUMAN	-0.342123	2.9807727
sp|P24752|THIL_HUMAN	-0.3417397	4.890839
sp|P20340-2|RAB6A_HUMAN	-0.3415661	3.8337784
sp|P38606|VATA_HUMAN	-0.3400993	4.760296
sp|P84103-2|SRSF3_HUMAN	-0.3383122	1.7165122
sp|P18428|LBP_HUMAN	-0.3371124	1.6960104
sp|O43491|E41L2_HUMAN	-0.3342419	3.760603
sp|Q13740-2|CD166_HUMAN	-0.3339768	4.5763893
sp|P62820|RAB1A_HUMAN	-0.3310661	2.1818786
sp|P35221|CTNA1_HUMAN	-0.3278141	9.457676
sp|P22307-8|NLTP_HUMAN	-0.3261461	1.6960104
sp|P17931|LEG3_HUMAN	-0.3258095	2.6576471
sp|P23528|COF1_HUMAN	-0.3257999	4.964395
sp|P43121|MUC18_HUMAN	-0.3249073	4.051346
sp|P84095|RHOG_HUMAN	-0.3198948	1.6960104
sp|Q13011|ECH1_HUMAN	-0.3188019	4.964395
sp|Q99729-3|ROAA_HUMAN	-0.3173122	2.9091687
sp|P0DPI2-2|GAL3A_HUMAN	-0.317009	1.6960104
sp|P60983|GMFB_HUMAN	-0.3119202	1.6960104
sp|P25774|CATS_HUMAN	-0.3104687	1.6960104
sp|Q02818|NUCB1_HUMAN	-0.3103485	4.964395
sp|P48163-2|MAOX_HUMAN	-0.3096685	1.6960104
sp|Q6NUK1-2|SCMC1_HUMAN	-0.309372	2.4829872
sp|Q15942|ZYX_HUMAN	-0.3068924	4.051346
sp|Q53GQ0|DHB12_HUMAN	-0.3059387	1.6960104
sp|P61604|CH10_HUMAN	-0.3054676	3.1266599

sp|Q16555|DPYL2_HUMAN	-0.3047829	6.5560675
sp|P11310-2|ACADM_HUMAN	-0.3035774	4.051346
sp|P60953|CDC42_HUMAN	-0.3026238	2.432837
sp|P54819-2|KAD2_HUMAN	-0.3022232	1.6960104
sp|P07954-2|FUMH_HUMAN	-0.2999878	2.3143692
sp|Q5SSJ5-2|HP1B3_HUMAN	-0.2999649	2.1818786
sp|Q9H4M9|EHD1_HUMAN	-0.2997551	2.7472508
sp|P80188|NGAL_HUMAN	-0.2996826	2.3047035
sp|O43707|ACTN4_HUMAN	-0.2959995	14.9131975
sp|P07686|HEXB_HUMAN	-0.2959213	2.9807727
sp|Q92597|NDRG1_HUMAN	-0.2957058	1.3815327
sp|P99999|CYC_HUMAN	-0.2956009	2.1818786
sp|P52566|GDIR2_HUMAN	-0.2955513	3.1266599
sp|P30740|ILEU_HUMAN	-0.2954063	3.342776
sp|O75390|CISY_HUMAN	-0.2953606	3.7395706
sp|O75955|FLOT1_HUMAN	-0.2937965	4.4923797
sp|O43837|IDH3B_HUMAN	-0.2935677	1.6960104
sp|Q02252-2|MMSA_HUMAN	-0.2928391	1.9190748
sp|P07942|LAMB1_HUMAN	-0.2908382	6.016212
sp|P51572-2|BAP31_HUMAN	-0.2901135	3.5908275
sp|Q00577|PURA_HUMAN	-0.2882156	2.6576471
sp|Q6UWY5|OLFL1_HUMAN	-0.2868023	3.412591
sp|O95865|DDAH2_HUMAN	-0.286129	2.0598862
sp|Q969G5|CAVN3_HUMAN	-0.2855263	1.6960104
sp|P37837|TALDO_HUMAN	-0.2841549	7.4078336
sp|P35222|CTNB1_HUMAN	-0.2827263	2.2613342
sp|Q96MM6|HS12B_HUMAN	-0.2817917	1.4050349
sp|P27361|MK03_HUMAN	-0.2817192	1.4104178
sp|P40926|MDHM_HUMAN	-0.2812653	6.412339
sp|Q15417|CNN3_HUMAN	-0.2805634	1.6960104
sp|O75367-2|H2AY_HUMAN	-0.2798977	3.0251918
sp|Q9P0V9-2|SEP10_HUMAN	-0.277729	1.9190748
sp|Q16658|FSCN1_HUMAN	-0.2773457	5.0928617
sp|Q04917|1433F_HUMAN	-0.2760124	4.3581686
sp|Q9H223|EHD4_HUMAN	-0.2748432	2.977915
sp|Q9UPN3|MACF1_HUMAN	-0.2719307	4.2386756
sp|O15144|ARPC2_HUMAN	-0.2697754	3.1266599
sp|P09497-2|CLCB_HUMAN	-0.268919	2.1818786
sp|P08311|CATG_HUMAN	-0.2675076	4.051346
sp|Q16853|AOC3_HUMAN	-0.2653198	5.4738517
sp|Q9HDC9|APMAP_HUMAN	-0.2633972	5.5196576
sp|Q9UHB6-4|LIMA1_HUMAN	-0.2613297	1.5646582
sp|P11413-2|G6PD_HUMAN	-0.2611218	2.7685447
sp|Q00765|REEP5_HUMAN	-0.2595348	3.1266599
sp|P23141-2|EST1_HUMAN	-0.2594585	8.994744

sp|P62873|GBB1_HUMAN	-0.2592945	3.342776
sp|Q9UGI8-2|TES_HUMAN	-0.2571316	2.7685447
sp|Q9P0L0-2|VAPA_HUMAN	-0.2562065	2.9807727
sp|O14950|ML12B_HUMAN	-0.2557735	1.9647322
sp|P08575-10|PTPRC_HUMAN	-0.2533054	4.051346
sp|O15511|ARPC5_HUMAN	-0.2533035	1.3815327
sp|Q9HCC0|MCCB_HUMAN	-0.2531147	1.6092666
sp|Q16363-2|LAMA4_HUMAN	-0.2508469	4.403177
sp|P09622|DLDH_HUMAN	-0.2505989	3.7395706
sp|P16219|ACADS_HUMAN	-0.2498417	2.6576471
sp|P02750|A2GL_HUMAN	-0.2494125	1.3080103
sp|P28074|PSB5_HUMAN	-0.2454014	1.560883
sp|P61020|RAB5B_HUMAN	-0.2440567	1.6960104
sp|P53634|CATC_HUMAN	-0.2434406	1.4104178
sp|Q9NNW7|TRXR2_HUMAN	-0.2425518	1.7590232
sp|Q9NP72|RAB18_HUMAN	-0.2422981	1.9647322
sp|Q14019|COTL1_HUMAN	-0.242096	2.4829872
sp|P00568|KAD1_HUMAN	-0.2399063	2.556708
sp|Q96CX2|KCD12_HUMAN	-0.2396431	5.351797
sp|Q9H0W9-2|CK054_HUMAN	-0.2394218	1.560883
sp|P21281|VATB2_HUMAN	-0.2393055	3.7273152
sp|Q96C19|EFHD2_HUMAN	-0.2391357	1.6960104
sp|Q96I99|SUCB2_HUMAN	-0.2378635	2.923088
sp|P35232|PHB_HUMAN	-0.2358513	4.789934
sp|P09651-3|ROA1_HUMAN	-0.2349625	1.6960104
sp|P22061-2|PIMT_HUMAN	-0.2348919	2.1818786
sp|Q9Y6N5|SQOR_HUMAN	-0.2346802	3.261252
sp|P11215-2|ITAM_HUMAN	-0.2338638	2.151766
sp|Q06830|PRDX1_HUMAN	-0.2330494	5.338249
sp|P52565|GDIR1_HUMAN	-0.232851	3.5908275
sp|P25788-2|PSA3_HUMAN	-0.2307301	2.6576471
sp|P43490|NAMPT_HUMAN	-0.2307186	6.7073655
sp|P46939-2|UTRO_HUMAN	-0.2305641	5.8330364
sp|P21796|VDAC1_HUMAN	-0.2300644	2.9462817
sp|P60660-2|MYL6_HUMAN	-0.2294884	3.671968
sp|Q15365|PCBP1_HUMAN	-0.2289505	2.6997027
sp|P22695|QCR2_HUMAN	-0.2282982	2.7472508
sp|P10606|COX5B_HUMAN	-0.2250004	1.6960104
sp|P09917|LOX5_HUMAN	-0.2249355	2.3143692
sp|P29401-2|TKT_HUMAN	-0.2247963	9.7664795
sp|Q14103-3|HNRPD_HUMAN	-0.2246933	3.9141645
sp|Q9ULV4-3|COR1C_HUMAN	-0.2236576	2.3388627
sp|Q9UIJ7|KAD3_HUMAN	-0.2230701	3.1266599
sp|P35237|SPB6_HUMAN	-0.2210293	4.2515793
sp|P08648|ITA5_HUMAN	-0.2198601	1.6960104

sp|P62993|GRB2_HUMAN	-0.2172852	1.9413493
sp|P09211|GSTP1_HUMAN	-0.2152176	1.6587203
sp|P06737-2|PYGL_HUMAN	-0.2148209	5.1043024
sp|P25786-2|PSA1_HUMAN	-0.2137871	4.865345
sp|Q9HBL0|TENS1_HUMAN	-0.2134819	1.9681813
sp|P63092-3|GNAS2_HUMAN	-0.2113724	1.7713763
sp|Q9H3N1|TMX1_HUMAN	-0.2098579	1.4104178
sp|Q04760-2|LGUL_HUMAN	-0.2097931	1.4716977
sp|Q9H2D6-2|TARA_HUMAN	-0.2097054	3.4656596
sp|O00764-2|PDXK_HUMAN	-0.2090864	3.1266599
sp|Q16881-2|TRXR1_HUMAN	-0.2088242	2.1918545
sp|P06733|ENOA_HUMAN	-0.2085266	1.3563709
sp|P16435|NCPR_HUMAN	-0.2068176	2.816493
sp|Q00839|HNRPU_HUMAN	-0.2059403	2.5602834
sp|P24539|AT5F1_HUMAN	-0.2047844	3.342776
sp|P62258|1433E_HUMAN	-0.2045002	2.1818786
sp|P09382|LEG1_HUMAN	-0.2036724	3.8106754
sp|P61019|RAB2A_HUMAN	-0.2033558	2.817951
sp|P47755|CAZA2_HUMAN	-0.2032661	4.005173
sp|P13073|COX41_HUMAN	-0.201416	1.6960104
sp|Q00325-2|MPCP_HUMAN	-0.2010212	2.4982266
sp|P50213|IDH3A_HUMAN	-0.2006645	2.4982266
sp|P67936|TPM4_HUMAN	-0.197794	2.4945192
sp|P37802|TAGL2_HUMAN	-0.1976986	2.3660793
sp|P43034|LIS1_HUMAN	-0.197506	1.827201
sp|Q9Y6W5|WASF2_HUMAN	-0.1974068	1.6960104
sp|P27487|DPP4_HUMAN	-0.1973724	1.9647322
sp|Q9BRA2|TXD17_HUMAN	-0.1973686	1.5646582
sp|P35914|HMGCL_HUMAN	-0.1966248	2.6576471
sp|P53007|TXTP_HUMAN	-0.1963253	2.4829872
sp|P22626|ROA2_HUMAN	-0.1962147	4.3709893
sp|P48681|NEST_HUMAN	-0.1960888	1.9828281
sp|Q13561-2|DCTN2_HUMAN	-0.1952591	4.797763
sp|P08571|CD14_HUMAN	-0.1950665	1.9647322
sp|P61158|ARP3_HUMAN	-0.1948128	5.815884
sp|P49748-2|ACADV_HUMAN	-0.1937447	7.5214024
sp|P46108|CRK_HUMAN	-0.1900902	2.9807727
sp|P46976-2|GLYG_HUMAN	-0.1883621	1.6997428
sp|P06576|ATPB_HUMAN	-0.1881218	8.452712
sp|Q99623|PHB2_HUMAN	-0.1877079	2.5385003
sp|P38646|GRP75_HUMAN	-0.1856461	5.089066
sp|Q9NYL9|TMOD3_HUMAN	-0.1854973	1.7590232
sp|P19338|NUCL_HUMAN	-0.1800919	6.412339
sp|P20618|PSB1_HUMAN	-0.1792908	3.342776
sp|P39060-1|COIA1_HUMAN	-0.1791649	2.2323174

sp|Q9NRN5-2|OLFL3_HUMAN	-0.1783733	3.5908275
sp|P07737|PROF1_HUMAN	-0.1773605	2.4402673
sp|O15143|ARC1B_HUMAN	-0.1772156	1.5270832
sp|P98160|PGBM_HUMAN	-0.1764088	6.62782
sp|Q03252|LMNB2_HUMAN	-0.1760731	7.7913103
sp|P10515|ODP2_HUMAN	-0.1755219	1.560883
sp|P51858|HDGF_HUMAN	-0.1724396	1.7590232
sp|Q92542|NICA_HUMAN	-0.1720104	1.6960104
sp|P23246|SFPQ_HUMAN	-0.1719952	2.846239
sp|P51649-2|SSDH_HUMAN	-0.171196	1.5646582
sp|P07858|CATB_HUMAN	-0.1688271	2.12598
sp|P18206-2|VINC_HUMAN	-0.1679058	15.95459
sp|P05198|IF2A_HUMAN	-0.1675453	2.1818786
sp|P07099|HYEP_HUMAN	-0.166069	5.045351
sp|O15031|PLXB2_HUMAN	-0.1653004	2.1663873
sp|Q8N1G4|LRC47_HUMAN	-0.165266	2.2884321
sp|Q86UP2-4|KTN1_HUMAN	-0.1650467	4.40577
sp|Q14152|EIF3A_HUMAN	-0.164113	1.6960104
sp|P25789|PSA4_HUMAN	-0.1622791	4.033051
sp|O00743-3|PPP6_HUMAN	-0.1605988	1.4104178
sp|Q13409-3|DC1I2_HUMAN	-0.1597834	2.432837
sp|Q9UNZ2-5|NSF1C_HUMAN	-0.1594524	1.6832623
sp|P00367|DHE3_HUMAN	-0.1575165	2.4331102
sp|P28066|PSA5_HUMAN	-0.1564026	2.6576471
sp|P04275|VWF_HUMAN	-0.1560917	2.8820472
sp|P60900|PSA6_HUMAN	-0.1549072	2.7155995
sp|P25787|PSA2_HUMAN	-0.1536369	2.653196
sp|Q9BS26|ERP44_HUMAN	-0.1517162	2.3143692
sp|Q13185|CBX3_HUMAN	-0.1505776	1.5646582
sp|P52209-2|6PGD_HUMAN	-0.1499519	2.2158308
sp|Q9Y3F4-2|STRAP_HUMAN	-0.1456661	1.6960104
sp|Q13308-6|PTK7_HUMAN	-0.1454697	1.9647322
sp|Q13451|FKBP5_HUMAN	-0.1447868	1.4936475
sp|O15127|SCAM2_HUMAN	-0.1438561	1.6960104
sp|Q86UX7-2|URP2_HUMAN	-0.143467	2.5880952
sp|P05023|AT1A1_HUMAN	-0.1423473	4.7194524
sp|P40306|PSB10_HUMAN	-0.1403656	1.4716977
sp|P30050|RL12_HUMAN	-0.1401024	1.5646582
sp|O15145|ARPC3_HUMAN	-0.1382332	1.6960104
sp|O75369-2|FLNB_HUMAN	-0.1357689	6.2336907
sp|Q14847|LASP1_HUMAN	-0.1317062	1.4351699
sp|P61981|1433G_HUMAN	-0.1309948	1.8992519
sp|P30041|PRDX6_HUMAN	-0.1297951	1.9891415
sp|P61978-3|HNRPK_HUMAN	-0.1294975	2.409984
sp|P55072|TERA_HUMAN	-0.1274414	6.0030184

sp|Q16543|CDC37_HUMAN	-0.1263008	3.4366283
sp|P38919|IF4A3_HUMAN	-0.1241837	1.7703108
sp|Q07954|LRP1_HUMAN	-0.123806	3.5392559
sp|P62316-2|SMD2_HUMAN	-0.1189861	1.6960104
sp|Q99714|HCD2_HUMAN	-0.1188946	2.3388627
sp|P11216|PYGB_HUMAN	-0.118	3.0333433
sp|P10809|CH60_HUMAN	-0.1164379	1.9237356
sp|P00505|AATM_HUMAN	-0.1139488	3.0491316
sp|P07237|PDIA1_HUMAN	-0.1138649	8.419361
sp|P49720|PSB3_HUMAN	-0.1126251	2.3143692
sp|Q9UHQ9|NB5R1_HUMAN	-0.1091118	1.7590232
sp|P47756-2|CAPZB_HUMAN	-0.1077042	2.2718198
sp|O14818|PSA7_HUMAN	-0.1057358	2.9027722
sp|P54920|SNAA_HUMAN	-0.1015854	1.7451575
sp|Q9Y490|TLN1_HUMAN	-0.1001263	7.125478
sp|P22897|MRC1_HUMAN	-0.0982876	3.0253785
sp|Q9BSJ8-2|ESYT1_HUMAN	-0.0976658	2.2178984
sp|O95571|ETHE1_HUMAN	-0.0972614	1.560883
sp|Q96AE4-2|FUBP1_HUMAN	-0.0961247	1.7451575
sp|P21810|PGS1_HUMAN	-0.0924568	1.4262929
sp|P13489|RINI_HUMAN	-0.090992	1.9696252
sp|P52907|CAZA1_HUMAN	-0.0904522	1.5983955
sp|P12109|CO6A1_HUMAN	-0.0899448	3.3101625
sp|O95831-3|AIFM1_HUMAN	-0.0880356	1.4068714
sp|P00387-3|NB5R3_HUMAN	-0.0868492	1.7713763
sp|P21291|CSRP1_HUMAN	-0.0811329	1.4068714
sp|P20700|LMNB1_HUMAN	-0.074213	2.669452
sp|P12110|CO6A2_HUMAN	-0.0720196	1.4641514
sp|P16157-21|ANK1_HUMAN	-0.0608616	1.4278674
sp|P21980|TGM2_HUMAN	-0.050108	1.4413939
sp|P05783|K1C18_HUMAN	-0.046957	1.9913259
sp|P11021|BIP_HUMAN	0.05473709	1.8804649
sp|P04792|HSPB1_HUMAN	0.0691433	1.9405096
sp|Q15746-2|MYLK_HUMAN	0.07836533	1.3095477
sp|P04004|VTNC_HUMAN	0.07925606	1.3635377
sp|P23396|RS3_HUMAN	0.08415794	2.4802232
sp|P11277-2|SPTB1_HUMAN	0.08441544	1.713281
sp|P09543-2|CN37_HUMAN	0.08536148	1.3635377
sp|P13010|XRCC5_HUMAN	0.0997448	1.4413342
sp|P13645|K1C10_HUMAN	0.10048866	1.4202096
sp|O60884|DNJA2_HUMAN	0.10574341	2.1818786
sp|P35606|COPB2_HUMAN	0.10971069	3.7420487
sp|P31040|SDHA_HUMAN	0.11091423	1.5167314
sp|Q9Y5Z4|HEBP2_HUMAN	0.1117878	1.6960104
sp|Q9UBX5|FBLN5_HUMAN	0.11547279	1.6265627

sp|P49411|EFTU_HUMAN	0.11693955	1.804513
sp|Q14112-2|NID2_HUMAN	0.11881447	2.37248
sp|P31146|COR1A_HUMAN	0.11898613	1.6896294
sp|P32119|PRDX2_HUMAN	0.12033081	3.456225
sp|P01009|A1AT_HUMAN	0.12061882	5.919944
sp|P05455|LA_HUMAN	0.12414932	4.051346
sp|Q8IZ83-3|A16A1_HUMAN	0.12590218	1.8060131
sp|P50914|RL14_HUMAN	0.12662125	1.6960104
sp|Q5EBM0|CMPK2_HUMAN	0.12701035	1.6960104
sp|P51911|CNN1_HUMAN	0.12716484	1.6587203
sp|Q9NYU2-2|UGGG1_HUMAN	0.12771988	1.7791386
sp|P49368|TCPG_HUMAN	0.13132286	2.2371037
sp|Q96G03|PGM2_HUMAN	0.13406944	1.867911
sp|Q07960|RHG01_HUMAN	0.13558578	1.3635377
sp|P13611|CSPG2_HUMAN	0.13620567	1.7697086
sp|O14828-2|SCAM3_HUMAN	0.13633537	1.4104178
sp|P04196|HRG_HUMAN	0.1386795	1.5071542
sp|P08236-2|BGLR_HUMAN	0.13996124	2.1818786
sp|Q9NZ08-2|ERAP1_HUMAN	0.14599991	1.8060131
sp|P46777|RL5_HUMAN	0.14676476	1.3895186
sp|Q15582|BGH3_HUMAN	0.14845657	3.9748769
sp|P61247|RS3A_HUMAN	0.15209961	2.4829872
sp|P23456|Trypsin	0.15538788	2.987788
sp|Q07507|DERM_HUMAN	0.15685463	2.2385595
sp|P05165-2|PCCA_HUMAN	0.15688324	2.7936864
sp|P27338|AOFB_HUMAN	0.15852547	2.2752137
sp|P18124|RL7_HUMAN	0.1622467	2.3143692
sp|Q9Y678|COPG1_HUMAN	0.16227913	2.4140475
sp|P15090|FABP4_HUMAN	0.1638794	1.9997257
sp|P46782|RS5_HUMAN	0.1702652	1.4278674
sp|Q15084-2|PDIA6_HUMAN	0.17501545	3.8108692
sp|P04264|K2C1_HUMAN	0.17563248	4.3733654
sp|P62701|RS4X_HUMAN	0.17564392	2.1818786
sp|P07357|CO8A_HUMAN	0.17887497	1.560883
sp|Q9Y2X3|NOP58_HUMAN	0.17954445	1.7713763
sp|P39656-3|OST48_HUMAN	0.18312836	2.400925
sp|P21399|ACOC_HUMAN	0.18331718	1.6405568
sp|Q99798|ACON_HUMAN	0.18606186	2.1260412
sp|P51888|PRELP_HUMAN	0.1930275	5.4389644
sp|P07900-2|HS90A_HUMAN	0.19477654	4.4093137
sp|P28838-2|AMPL_HUMAN	0.1955967	6.2617097
sp|P46063|RECQ1_HUMAN	0.19648743	1.4936475
sp|P21912|SDHB_HUMAN	0.19827461	1.4104178
sp|O15061|SYNEM_HUMAN	0.20263195	1.6997428
sp|O60763-2|USO1_HUMAN	0.20669174	2.9318283

sp|Q9Y4L1|HYOU1_HUMAN	0.2067852	3.5053878
sp|P62424|RL7A_HUMAN	0.20786858	2.2323174
sp|Q9UHL4|DPP2_HUMAN	0.20915985	2.987788
sp|P35555|FBN1_HUMAN	0.2127552	7.1820946
sp|P32969|RL9_HUMAN	0.21372795	1.6960104
sp|P53618|COPB_HUMAN	0.21648026	2.9455793
sp|Q99873-3|ANM1_HUMAN	0.2170639	2.3143692
sp|P02765|FETUA_HUMAN	0.21829796	1.560883
sp|A1L4H1|SRCRL_HUMAN	0.21851921	1.4104178
sp|P04844|RPN2_HUMAN	0.21877575	1.9190748
sp|P21266|GSTM3_HUMAN	0.22295761	2.5461748
sp|Q96PD5-2|PGRP2_HUMAN	0.22309685	1.4716977
sp|P07814|SYEP_HUMAN	0.22724724	2.044628
sp|Q16647|PTGIS_HUMAN	0.22934341	2.3143692
sp|P00915|CAH1_HUMAN	0.23254204	6.016213
sp|P49419-2|AL7A1_HUMAN	0.23557663	4.957485
sp|P23219-2|PGH1_HUMAN	0.23933029	1.5646582
sp|Q15124|PGM5_HUMAN	0.24077415	5.489956
sp|P02649|APOE_HUMAN	0.24200058	1.9986866
sp|P08708|RS17_HUMAN	0.2457695	1.560883
sp|Q9BS40|LXN_HUMAN	0.2472763	1.7590232
sp|Q9P2E9|RRBP1_HUMAN	0.24790955	11.459226
sp|P22352|GPX3_HUMAN	0.24960709	1.6960104
sp|Q07065|CKAP4_HUMAN	0.2504692	7.9042997
sp|Q9UBT2|SAE2_HUMAN	0.25064087	2.1818786
sp|P24844|MYL9_HUMAN	0.25358582	1.6960104
sp|P26640|SYVC_HUMAN	0.2538948	2.653196
sp|P04843|RPN1_HUMAN	0.25589943	7.3409
sp|P05156|CFAI_HUMAN	0.25873184	3.417183
sp|P62266|RS23_HUMAN	0.2628498	1.560883
sp|Q9Y3Z3|SAMH1_HUMAN	0.26325417	3.4253109
sp|O60547-2|GMDS_HUMAN	0.26397705	1.6960104
sp|Q9H008|LHPP_HUMAN	0.26474285	1.6092666
sp|Q8N2S1|LTBP4_HUMAN	0.26700974	3.4805238
sp|P55884-2|EIF3B_HUMAN	0.2679901	1.9951487
sp|O60831|PRAF2_HUMAN	0.26823616	1.6960104
sp|P67936-2|TPM4_HUMAN	0.2710743	1.6960104
sp|P09493-9|TPM1_HUMAN	0.27181244	1.6960104
sp|O00567|NOP56_HUMAN	0.2743225	3.5908275
sp|Q92896-2|GSLG1_HUMAN	0.27923203	4.051346
sp|P53621-2|COPA_HUMAN	0.27968788	5.4724083
sp|O00534|VMA5A_HUMAN	0.28222847	3.6746628
sp|P22314|UBA1_HUMAN	0.2824669	6.5729823
sp|P08670|VIME_HUMAN	0.28351784	14.398287
sp|P02753|RET4_HUMAN	0.2837162	1.6960104

sp|P09493-8|TPM1_HUMAN	0.2854805	3.4656596
sp|P20774|MIME_HUMAN	0.28650475	2.6576471
sp|P13667|PDIA4_HUMAN	0.2994175	9.884588
sp|P43652|AFAM_HUMAN	0.3023033	3.1629772
sp|Q9UMS6-2|SYNP2_HUMAN	0.30309296	2.838374
sp|Q7KZF4|SND1_HUMAN	0.3034973	2.6928825
sp|Q96C86|DCPS_HUMAN	0.30522537	1.6960104
sp|P14550|AK1A1_HUMAN	0.3059845	4.578167
sp|O43175|SERA_HUMAN	0.31017685	2.923088
sp|P14625|ENPL_HUMAN	0.31710434	13.666788
sp|P40261|NNMT_HUMAN	0.31984138	1.4104178
sp|Q00341-2|VIGLN_HUMAN	0.32055855	2.1404836
sp|P02774-3|VTDB_HUMAN	0.3233528	7.445339
sp|Q8TCJ2|STT3B_HUMAN	0.3306694	1.6960104
sp|Q5TZA2|CROCC_HUMAN	0.33731842	2.1818786
sp|Q14767|LTBP2_HUMAN	0.3391304	3.0253785
sp|P49257|LMAN1_HUMAN	0.3395157	2.7075508
sp|P26373|RL13_HUMAN	0.34363365	2.1818786
sp|Q01995|TAGL_HUMAN	0.34490585	6.892501
sp|P03952|KLKB1_HUMAN	0.35798836	2.1818786
sp|P02766|TTHY_HUMAN	0.35956192	2.1818786
sp|P10909-5|CLUS_HUMAN	0.36676407	4.964395
sp|P02748|CO9_HUMAN	0.36956787	6.8719745
sp|P04217|A1BG_HUMAN	0.3714943	3.8106754
sp|P39023|RL3_HUMAN	0.37233925	2.1818786
sp|P08727|K1C19_HUMAN	0.37722778	9.893875
sp|P39059|COFA1_HUMAN	0.3824768	2.1818786
sp|Q9NR12-2|PDLI7_HUMAN	0.3890381	2.058973
sp|P02749|APOH_HUMAN	0.39386368	1.3567923
sp|P28331-2|NDUS1_HUMAN	0.39700127	2.6576471
sp|P01019|ANGT_HUMAN	0.39979362	1.5646582
sp|P36578|RL4_HUMAN	0.41420937	3.5908275
sp|P06727|APOA4_HUMAN	0.42101288	5.727641
sp|Q9P2B2|FPRP_HUMAN	0.42125893	3.1266599
sp|P22105-1|TENX_HUMAN	0.42806244	11.11263
sp|Q14192|FHL2_HUMAN	0.43423462	2.1818786
sp|Q14195-2|DPYL3_HUMAN	0.45190048	7.0677576
sp|P06681-3|CO2_HUMAN	0.45391083	1.4278674
sp|P05787-2|K2C8_HUMAN	0.45598602	14.230314
sp|P25311|ZA2G_HUMAN	0.45905685	4.051346
sp|P36955|PEDF_HUMAN	0.46026993	3.8337784
sp|P0DOX7|IGK_HUMAN	0.46348763	2.1818786
sp|P62753|RS6_HUMAN	0.46616554	1.6960104
sp|P36269-3|GGT5_HUMAN	0.46954727	3.1266599
sp|P16615|AT2A2_HUMAN	0.4719906	6.2357526

sp|Q14315-2|FLNC_HUMAN	0.4813919	15.051499
sp|P49821-2|NDUV1_HUMAN	0.48513794	3.5908275
sp|P23946|CMA1_HUMAN	0.48846245	2.4829872
sp|P04259|K2C6B_HUMAN	0.49025345	2.1818786
sp|P29622|KAIN_HUMAN	0.49585724	2.6576471
sp|P10643|CO7_HUMAN	0.49674225	4.5635147
sp|P19827|ITIH1_HUMAN	0.4969883	4.517996
sp|P07585|PGS2_HUMAN	0.49738693	5.102962
sp|Q9Y265|RUVB1_HUMAN	0.5025749	4.051346
sp|P42224-2|STAT1_HUMAN	0.50473976	2.6576471
sp|P00488|F13A_HUMAN	0.5065956	5.856767
sp|P0DOX2|IGA2_HUMAN	0.508091	1.6960104
sp|P62241|RS8_HUMAN	0.51481247	2.9807727
sp|Q12765|SCRN1_HUMAN	0.52611923	1.9647322
sp|Q9NR45|SIAS_HUMAN	0.52801895	4.964395
sp|P80303-2|NUCB2_HUMAN	0.5286598	2.1818786
sp|P13671|CO6_HUMAN	0.53061676	1.6960104
sp|P51884|LUM_HUMAN	0.53601074	8.112776
sp|P01008|ANT3_HUMAN	0.53883743	8.061325
sp|Q9Y230|RUVB2_HUMAN	0.56750107	6.3204336
sp|Q00796|DHSO_HUMAN	0.5684681	1.6960104
sp|Q13162|PRDX4_HUMAN	0.5702286	3.1266599
sp|P20591|MX1_HUMAN	0.578125	2.6576471
sp|P02675|FIBB_HUMAN	0.5934715	9.769767
sp|P07451|CAH3_HUMAN	0.5950985	1.9997257
sp|P01031|CO5_HUMAN	0.6045685	6.2111583
sp|P02679-2|FIBG_HUMAN	0.6104412	6.878014
sp|P19823|ITIH2_HUMAN	0.61401176	5.417897
sp|P02790|HEMO_HUMAN	0.64281464	7.831889
sp|P00450|CERU_HUMAN	0.64979553	10.83602
sp|P04114|APOB_HUMAN	0.6733074	12.475591
sp|P00748|FA12_HUMAN	0.679533	1.6960104
sp|P01023|A2MG_HUMAN	0.68457985	14.386388
sp|P01042-2|KNG1_HUMAN	0.68932724	7.2183566
sp|Q8NBS9|TXND5_HUMAN	0.71315575	7.665951
sp|P02652-2|APOA2_HUMAN	0.7177887	3.1266599
sp|P00734|THRB_HUMAN	0.7510433	6.314202
sp|Q9BW30|TPPP3_HUMAN	0.76011086	3.1266599
sp|Q05707|COEA1_HUMAN	0.77067566	14.507432
sp|P02671|FIBA_HUMAN	0.77326393	11.667864
sp|P00751|CFAB_HUMAN	0.7750225	10.241625
sp|Q8WU39|MZB1_HUMAN	0.8037529	3.1266599
sp|Q9NYL4|FKB11_HUMAN	0.82024765	1.6960104
sp|P08123|CO1A2_HUMAN	0.82531166	2.6576471
sp|P13647|K2C5_HUMAN	0.8616257	6.7698927

sp|P08603|CFAH_HUMAN	0.8723297	15.35253
sp|P00747|PLMN_HUMAN	0.9109764	6.7698927
sp|P27169|PON1_HUMAN	0.9162636	1.6960104
sp|P17661|DESM_HUMAN	0.94592094	14.840647
sp|P01859|IGHG2_HUMAN	0.95537186	4.509013
sp|P04003|C4BPA_HUMAN	0.9865322	5.869831
sp|P02647|APOA1_HUMAN	1.0032425	13.420564
sp|P01876|IGHA1_HUMAN	1.0350742	4.509013
sp|P01871-2|IGHM_HUMAN	1.0938072	2.1818786
sp|P05546|HEP2_HUMAN	1.094387	2.6576471
sp|P0DOY3|IGLC3_HUMAN	1.1071568	2.1818786
sp|P02452|CO1A1_HUMAN	1.1218872	2.1800864
sp|P0DOX8|IGL1_HUMAN	1.1790733	2.1818786
sp|Q8TDL5|BPIB1_HUMAN	1.2697239	2.044628
sp|P0DOX5|IGG1_HUMAN	1.2765732	4.964395
sp|Q9BXN1|ASPN_HUMAN	1.3565903	2.6576471
sp|P01860|IGHG3_HUMAN	1.5645103	2.6576471
sp|P15088|CBPA3_HUMAN	1.9092846	2.1818786
sp|Q9BW04|SARG_HUMAN	-1.9911194	1.1932944
sp|P04229|2B11_HUMAN	-1.6544609	0.656254
sp|O00757|F16P2_HUMAN	-1.3538017	1.1932944
sp|P13686|PPA5_HUMAN	-1.2505798	1.2773042
sp|P37235|HPCL1_HUMAN	-1.1461496	0.656254
sp|P08246|ELNE_HUMAN	-1.102642	1.1932944
sp|P33151|CADH5_HUMAN	-1.0502663	1.1932944
sp|Q93077|H2A1C_HUMAN	-1.0113544	1.1932944
sp|Q16777|H2A2C_HUMAN	-1.0048599	1.1932944
sp|P28330|ACADL_HUMAN	-0.9859142	1.1932944
sp|P08263|GSTA1_HUMAN	-0.9842415	0
sp|O00592-2|PODXL_HUMAN	-0.9434471	1.1932944
sp|P54108-2|CRIS3_HUMAN	-0.8950558	0.656254
sp|Q9UHN6-2|CEIP2_HUMAN	-0.883255	1.1932944
sp|P20702|ITAX_HUMAN	-0.8786964	0.656254
sp|Q01955|CO4A3_HUMAN	-0.8611202	1.1932944
sp|Q14956-2|GPNMB_HUMAN	-0.820713	1.1932944
sp|P16422|EPCAM_HUMAN	-0.8109779	1.1932944
sp|Q03135-2|CAV1_HUMAN	-0.8097458	0.656254
sp|Q13751|LAMB3_HUMAN	-0.8041515	1.2773042
sp|P63218|GBG5_HUMAN	-0.7971249	1.1932944
sp|P29972-2|AQP1_HUMAN	-0.7817631	1.1932944
sp|Q9UDY2-3|ZO2_HUMAN	-0.7722426	1.1932944
sp|P02462|CO4A1_HUMAN	-0.7241011	1.1505735
sp|Q9NPY3|C1QR1_HUMAN	-0.7229805	1.1932944
sp|Q08209-5|PP2BA_HUMAN	-0.7123013	0.7827403
sp|O76041-2|NEBL_HUMAN	-0.69207	0.656254

sp|Q96BM9|ARL8A_HUMAN	-0.6917801	0.656254
sp|O95837|GNA14_HUMAN	-0.6908684	0.656254
sp|P02741|CRP_HUMAN	-0.6864872	1.1932944
sp|P24158|PRTN3_HUMAN	-0.6850205	1.1505735
sp|Q15286|RAB35_HUMAN	-0.6650314	0.656254
sp|P04440|DPB1_HUMAN	-0.6330109	1.1932944
sp|Q9UPQ0|LIMC1_HUMAN	-0.6275845	1.2392054
sp|P06703|S10A6_HUMAN	-0.614624	1.1932944
sp|O43795-2|MYO1B_HUMAN	-0.6130638	1.1932944
sp|P16403|H12_HUMAN	-0.5995483	0.656254
sp|P06396-2|GELS_HUMAN	-0.589962	0.656254
sp|Q9NVJ2|ARL8B_HUMAN	-0.5896835	1.1932944
sp|Q9H8L6|MMRN2_HUMAN	-0.5797596	0.7827403
sp|Q9H2U2-3|IPYR2_HUMAN	-0.576313	0.656254
sp|P51606-2|RENBP_HUMAN	-0.5749989	1.1932944
sp|P11279|LAMP1_HUMAN	-0.5684185	1.1932944
sp|P51153|RAB13_HUMAN	-0.5536308	0.656254
sp|P61916-2|NPC2_HUMAN	-0.5519371	1.1932944
sp|P08962-2|CD63_HUMAN	-0.5452595	0.7827403
sp|P20292|AL5AP_HUMAN	-0.5372086	0.91601294
sp|Q08431|MFGM_HUMAN	-0.5339108	1.1505735
sp|P36542-2|ATPG_HUMAN	-0.5288067	0.656254
sp|P28799-3|GRN_HUMAN	-0.5285082	0.2178309
sp|P01920|DQB1_HUMAN	-0.526331	1.1932944
sp|P22894|MMP8_HUMAN	-0.5245647	0.19149946
sp|P48509|CD151_HUMAN	-0.5219727	1.1932944
sp|O60271-4|JIP4_HUMAN	-0.5205402	1.0301651
sp|Q7Z4I7-3|LIMS2_HUMAN	-0.5158291	1.1932944
sp|P11717|MPRI_HUMAN	-0.507185	1.1932944
sp|P06753-6|TPM3_HUMAN	-0.5033169	0.656254
sp|Q9HD89|RETN_HUMAN	-0.5021477	1.1932944
sp|Q6DN03|H2B2C_HUMAN	-0.4989319	1.1932944
sp|Q9NVD7|PARVA_HUMAN	-0.4988899	0.7827403
sp|Q9UBR2|CATZ_HUMAN	-0.4966736	1.1932944
sp|Q9BZF9-2|UACA_HUMAN	-0.4929657	0.7827403
sp|P01111|RASN_HUMAN	-0.4795322	0.656254
sp|Q8NBQ5|DHB11_HUMAN	-0.4729729	1.1932944
sp|P61601|NCALD_HUMAN	-0.4726429	0.656254
sp|A6NMY6|AXA2L_HUMAN	-0.4706039	0.656254
sp|P38159|RBMX_HUMAN	-0.4679642	0.656254
sp|P62745|RHOB_HUMAN	-0.4676495	0.656254
sp|Q7L2H7|EIF3M_HUMAN	-0.4674988	0.656254
sp|Q96HD1|CREL1_HUMAN	-0.4549599	0.7827403
sp|P62834|RAP1A_HUMAN	-0.4510593	0.656254
sp|P69891|HBG1_HUMAN	-0.4465962	0.656254

sp|P10619-2|PPGB_HUMAN	-0.4459305	1.1932944
sp|Q07075|AMPE_HUMAN	-0.4347382	1.0485198
sp|Q13813-3|SPTN1_HUMAN	-0.4271793	1.1932944
sp|Q92522|H1X_HUMAN	-0.4260712	0.7827403
sp|Q9NPJ3|ACO13_HUMAN	-0.4256153	1.1932944
sp|Q13813-2|SPTN1_HUMAN	-0.4245844	1.1932944
sp|P12830|CADH1_HUMAN	-0.4204903	1.1932944
sp|Q86TX2|ACOT1_HUMAN	-0.4181728	0.656254
sp|Q9Y4G6|TLN2_HUMAN	-0.4112597	0.656254
sp|P61006|RAB8A_HUMAN	-0.4077721	1.1932944
sp|P78417-3|GSTO1_HUMAN	-0.4045334	1.1932944
sp|Q9Y376|CAB39_HUMAN	-0.401516	1.2773042
sp|Q07812-7|BAX_HUMAN	-0.3921318	1.1932944
sp|Q32MZ4-3|LRRF1_HUMAN	-0.3904533	1.1932944
sp|Q6NY19-2|KANK3_HUMAN	-0.3844643	1.1932944
sp|Q6IAA8|LTOR1_HUMAN	-0.3835869	1.1932944
sp|P08631-2|HCK_HUMAN	-0.3789673	0.656254
sp|P28676|GRAN_HUMAN	-0.3788109	1.1932944
sp|P13761|2B17_HUMAN	-0.3781185	0
sp|Q13976|KGP1_HUMAN	-0.3720741	0
sp|P06753-2|TPM3_HUMAN	-0.3594627	1.1932944
sp|Q12846|STX4_HUMAN	-0.3551788	0.656254
sp|P29466-2|CASP1_HUMAN	-0.3542461	0.656254
sp|Q13283|G3BP1_HUMAN	-0.3516502	0.6070219
sp|O15296|LX15B_HUMAN	-0.3506241	0.69308156
sp|P15170-2|ERF3A_HUMAN	-0.3471737	1.1932944
sp|P06865|HEXA_HUMAN	-0.3452396	1.2773042
sp|Q16822|PCKGM_HUMAN	-0.3429833	1.1932944
sp|Q9BQE3|TBA1C_HUMAN	-0.3429632	0.656254
sp|P61586|RHOA_HUMAN	-0.3418312	1.1932944
sp|P51636-2|CAV2_HUMAN	-0.3390656	1.1932944
sp|P63241|IF5A1_HUMAN	-0.3367519	0.656254
sp|O75695|XRP2_HUMAN	-0.3318119	1.1505735
sp|P09493-5|TPM1_HUMAN	-0.3316631	1.1932944
sp|Q9ULZ3-2|ASC_HUMAN	-0.3308754	1.1932944
sp|P52789|HXK2_HUMAN	-0.3306599	0.656254
sp|Q96FN4|CPNE2_HUMAN	-0.328063	0.656254
sp|Q9Y2Q5|LTOR2_HUMAN	-0.3256674	0.7827403
sp|Q15056-2|IF4H_HUMAN	-0.3248253	1.1932944
sp|P30049|ATPD_HUMAN	-0.322937	1.1932944
sp|P06239-3|LCK_HUMAN	-0.3208847	0.656254
sp|P10412|H14_HUMAN	-0.3168945	0.19149946
sp|Q7Z7H5-3|TMED4_HUMAN	-0.3149433	1.1932944
sp|Q13404|UB2V1_HUMAN	-0.3139248	1.2773042
sp|Q8N335|GPD1L_HUMAN	-0.3130608	1.1505735

sp|Q96EP5-2|DAZP1_HUMAN	-0.3117504	1.1932944
sp|P61956-2|SUMO2_HUMAN	-0.3098946	0.656254
sp|P30044-2|PRDX5_HUMAN	-0.3091354	0.656254
sp|P57088|TMM33_HUMAN	-0.3031445	1.1932944
sp|Q6P4A8|PLBL1_HUMAN	-0.299511	1.0485198
sp|P29992|GNA11_HUMAN	-0.2987671	1.1932944
sp|P62314|SMD1_HUMAN	-0.2978888	0.7827403
sp|Q8NC51-3|PAIRB_HUMAN	-0.2975025	1.1932944
sp|P62070-4|RRAS2_HUMAN	-0.2966995	0.656254
sp|Q15149-9|PLEC_HUMAN	-0.2964306	0.656254
sp|Q30154|DRB5_HUMAN	-0.2930164	0.656254
sp|Q6ZVM7-3|TM1L2_HUMAN	-0.290946	0.7827403
sp|Q9H2G2-2|SLK_HUMAN	-0.2883568	0.7827403
sp|Q13177|PAK2_HUMAN	-0.2882862	1.1932944
sp|Q9HB71|CYBP_HUMAN	-0.2855434	1.1932944
sp|Q01518|CAP1_HUMAN	-0.285039	0.656254
sp|P48059-3|LIMS1_HUMAN	-0.2836418	0.656254
sp|Q99436|PSB7_HUMAN	-0.2812386	1.1932944
sp|P31946-2|1433B_HUMAN	-0.279213	0.656254
sp|Q92930|RAB8B_HUMAN	-0.2779121	0.656254
sp|Q9H299|SH3L3_HUMAN	-0.2760468	1.1932944
sp|Q7LG56-6|RIR2B_HUMAN	-0.275178	1.1932944
sp|P42285|MTREX_HUMAN	-0.2736645	0.45033538
sp|P20339-2|RAB5A_HUMAN	-0.2726879	0.656254
sp|P02794|FRIH_HUMAN	-0.2708988	0.84879977
sp|P42167|LAP2B_HUMAN	-0.2702198	1.1932944
sp|P60033|CD81_HUMAN	-0.2699928	1.1932944
sp|P61225|RAP2B_HUMAN	-0.2691708	1.1932944
sp|P26368-2|U2AF2_HUMAN	-0.2689734	1.1932944
sp|Q03113|GNA12_HUMAN	-0.2669525	0.656254
sp|P01112|RASH_HUMAN	-0.2651081	0.656254
sp|P63096|GNAI1_HUMAN	-0.2648754	1.1505735
sp|P62942|FKB1A_HUMAN	-0.2647018	0.656254
sp|P61457|PHS_HUMAN	-0.2581797	1.1932944
sp|Q8NHP8|PLBL2_HUMAN	-0.2579041	0.45033538
sp|O14786|NRP1_HUMAN	-0.2572918	1.1932944
sp|Q99961-3|SH3G1_HUMAN	-0.2567253	0.656254
sp|P35579-2|MYH9_HUMAN	-0.2551422	0.656254
sp|Q9UFN0|NPS3A_HUMAN	-0.2535267	0.7061832
sp|Q14254|FLOT2_HUMAN	-0.2502251	1.214352
sp|P11234-2|RALB_HUMAN	-0.2501926	0.656254
sp|P35754|GLRX1_HUMAN	-0.2496758	1.1791906
sp|Q9UL18|AGO1_HUMAN	-0.2490635	1.0301651
sp|Q9NUB1-2|ACS2L_HUMAN	-0.2487145	0.45033538
sp|P52788-2|SPSY_HUMAN	-0.2482758	0.7827403

sp|Q9NUJ1-3|ABHDA_HUMAN	-0.247879	0.7827403
sp|P45954-2|ACDSB_HUMAN	-0.247572	0.45033538
sp|P63220|RS21_HUMAN	-0.2471924	1.1932944
sp|P12236|ADT3_HUMAN	-0.2471237	1.1932944
sp|Q9NZ32|ARP10_HUMAN	-0.2464581	1.1932944
sp|Q15121|PEA15_HUMAN	-0.2463894	0.5204253
sp|P04839|CY24B_HUMAN	-0.2462091	1.1932944
sp|O60814|H2B1K_HUMAN	-0.2459583	0.2178309
sp|P25685-2|DNJB1_HUMAN	-0.2451	0.45033538
sp|O15400-2|STX7_HUMAN	-0.2450676	0.20467198
sp|O00186|STXB3_HUMAN	-0.2432032	0.45033538
sp|Q15181|IPYR_HUMAN	-0.24263	0.45563722
sp|A5A3E0|POTEF_HUMAN	-0.2423496	0
sp|P15529-10|MCP_HUMAN	-0.2395859	0.656254
sp|Q9BWD1|THIC_HUMAN	-0.2393532	0.7827403
sp|Q04695|K1C17_HUMAN	-0.23839	0.656254
sp|Q04837|SSBP_HUMAN	-0.2370281	1.1932944
sp|Q53T59|H1BP3_HUMAN	-0.2353993	0.45033538
sp|O95716|RAB3D_HUMAN	-0.2349243	0.656254
sp|P49756|RBM25_HUMAN	-0.2318668	1.1505735
sp|P12694|ODBA_HUMAN	-0.2310982	0.35795313
sp|P02511|CRYAB_HUMAN	-0.2300072	1.0301651
sp|Q05315|LEG10_HUMAN	-0.2274513	0
sp|Q14011-2|CIRBP_HUMAN	-0.2270289	0.91601294
sp|P61160|ARP2_HUMAN	-0.2253075	1.1932944
sp|Q9UH99-3|SUN2_HUMAN	-0.2233753	0.40256184
sp|P15311|EZRI_HUMAN	-0.2230864	0.1806812
sp|Q15404|RSU1_HUMAN	-0.2161007	0.656254
sp|Q9NX63|MIC19_HUMAN	-0.2152042	1.1932944
sp|P28072|PSB6_HUMAN	-0.2130413	1.1505735
sp|P10599-2|THIO_HUMAN	-0.2129173	0.6298893
sp|P46783|RS10_HUMAN	-0.2125902	0.45033538
sp|P31946|1433B_HUMAN	-0.2124577	0.656254
sp|O75347|TBCA_HUMAN	-0.2123451	0.5204253
sp|Q5JRX3-3|PREP_HUMAN	-0.2104549	1.1932944
sp|P50552|VASP_HUMAN	-0.2102919	1.0370445
sp|P12235|ADT1_HUMAN	-0.2080097	0.656254
sp|Q16762|THTR_HUMAN	-0.2074986	0.40256184
sp|P00740|FA9_HUMAN	-0.2072277	0.312067
sp|P63167|DYL1_HUMAN	-0.2069092	0.656254
sp|P01892|1A02_HUMAN	-0.2059727	0.2178309
sp|P62826|RAN_HUMAN	-0.2036114	1.214352
sp|Q8N684-3|CPSF7_HUMAN	-0.2028027	0.6070219
sp|P63261|ACTG_HUMAN	-0.2024479	0
sp|P28482|MK01_HUMAN	-0.2024307	1.3006523

sp|Q02978-2|M2OM_HUMAN	-0.2014694	1.1505735
sp|P61026|RAB10_HUMAN	-0.200552	0.7827403
sp|P61224|RAP1B_HUMAN	-0.1998682	0
sp|Q5JPE7-2|NOMO2_HUMAN	-0.1992645	1.1505735
sp|Q06136|KDSR_HUMAN	-0.1982708	1.1932944
sp|Q15819|UB2V2_HUMAN	-0.198204	0.656254
sp|Q9Y394-2|DHRS7_HUMAN	-0.1981049	0.5111962
sp|O60784-3|TOM1_HUMAN	-0.1937523	0.656254
sp|P51553-2|IDH3G_HUMAN	-0.1933632	1.1932944
sp|Q9Y696|CLIC4_HUMAN	-0.1920815	0.90036625
sp|O00231-2|PSD11_HUMAN	-0.1916943	1.0485198
sp|Q92905|CSN5_HUMAN	-0.1913796	0.19149946
sp|Q04323-2|UBXN1_HUMAN	-0.1912251	1.1932944
sp|P30046|DOPD_HUMAN	-0.1903477	1.1932944
sp|P07948-2|LYN_HUMAN	-0.1902199	0.7827403
sp|P60981|DEST_HUMAN	-0.1898918	0.7498006
sp|P23786|CPT2_HUMAN	-0.1895332	0.8983557
sp|Q03154-4|ACY1_HUMAN	-0.1890774	0.91601294
sp|Q9BXS5-2|AP1M1_HUMAN	-0.1877632	1.1276597
sp|P01911|2B1F_HUMAN	-0.1877251	0.45033538
sp|Q9NQC3|RTN4_HUMAN	-0.1867485	0.7588735
sp|Q8NBF2-2|NHLC2_HUMAN	-0.186039	0.7827403
sp|Q02218-2|ODO1_HUMAN	-0.1848354	1.1791906
sp|P55010|IF5_HUMAN	-0.1847973	1.1276597
sp|Q9NZB2-6|F120A_HUMAN	-0.1836395	0.6070219
sp|P16298-4|PP2BB_HUMAN	-0.1834526	0.6070219
sp|O43813|LANC1_HUMAN	-0.1832848	1.1505735
sp|P06748-2|NPM_HUMAN	-0.1826496	0.35795313
sp|Q8NHV1|GIMA7_HUMAN	-0.1798935	0.7827403
sp|P52758|RIDA_HUMAN	-0.1791325	0.19149946
sp|P31153|METK2_HUMAN	-0.1781483	0.656254
sp|Q9HB40|RISC_HUMAN	-0.1751652	1.0301651
sp|P08134|RHOC_HUMAN	-0.1747513	0.19149946
sp|Q16630-2|CPSF6_HUMAN	-0.1740704	1.1505735
sp|P53004|BIEA_HUMAN	-0.1740665	0.84879977
sp|Q9UBV8|PEF1_HUMAN	-0.1739674	0.91601294
sp|Q15833-2|STXB2_HUMAN	-0.17313	0.19149946
sp|O94760|DDAH1_HUMAN	-0.1719494	1.0485198
sp|Q86W92-2|LIPB1_HUMAN	-0.1718235	0.45033538
sp|Q14651|PLSI_HUMAN	-0.1715412	0
sp|Q29963|1C06_HUMAN	-0.1700954	0.656254
sp|P23142-4|FBLN1_HUMAN	-0.1674681	0.656254
sp|Q09028-3|RBBP4_HUMAN	-0.1669903	0.656254
sp|P67809|YBOX1_HUMAN	-0.1634827	0.19149946
sp|P12955|PEPD_HUMAN	-0.1634293	1.1240381

sp|P58546|MTPN_HUMAN	-0.1627369	0.91601294
sp|P51178-2|PLCD1_HUMAN	-0.1612358	0.2178309
sp|Q9UKK9|NUDT5_HUMAN	-0.1611366	0.45033538
sp|P00403|COX2_HUMAN	-0.1593933	0.5111962
sp|Q9NUQ9|FA49B_HUMAN	-0.1592751	0.91601294
sp|Q9C0B1|FTO_HUMAN	-0.158823	0.45033538
sp|Q9NRV9|HEBP1_HUMAN	-0.1574154	0.40256184
sp|P62136|PP1A_HUMAN	-0.1569748	1.1505735
sp|P61163|ACTZ_HUMAN	-0.1568661	1.0485198
sp|Q9NZU5-2|LMCD1_HUMAN	-0.1568489	0.09851671
sp|P13693|TCTP_HUMAN	-0.1565743	1.1932944
sp|P62879|GBB2_HUMAN	-0.1560669	0.5111962
sp|P61758|PFD3_HUMAN	-0.1555023	1.1932944
sp|P30533|AMRP_HUMAN	-0.155468	0.4075265
sp|Q15637-2|SF01_HUMAN	-0.1552792	1.1932944
sp|Q9Y305-4|ACOT9_HUMAN	-0.1548615	0
sp|P47985|UCRI_HUMAN	-0.1544685	0.2178309
sp|P16930|FAAA_HUMAN	-0.153244	0.656254
sp|P50452|SPB8_HUMAN	-0.1529732	0.45033538
sp|P09525|ANXA4_HUMAN	-0.1523819	0.565784
sp|Q9GZP4-2|PITH1_HUMAN	-0.151515	1.1932944
sp|P60891|PRPS1_HUMAN	-0.1481819	0.656254
sp|P21964-2|COMT_HUMAN	-0.1475315	0.43103603
sp|P48449-3|ERG7_HUMAN	-0.1474056	1.0485198
sp|Q9UBW8|CSN7A_HUMAN	-0.1473217	0.5204253
sp|Q9BTE1|DCTN5_HUMAN	-0.145649	0.19149946
sp|Q9UNF0-2|PACN2_HUMAN	-0.1441193	0.95332193
sp|P07437|TBB5_HUMAN	-0.1437683	0.5111962
sp|P18084|ITB5_HUMAN	-0.1430092	0.7827403
sp|O75937|DNJC8_HUMAN	-0.1421814	1.2095301
sp|P53597|SUCA_HUMAN	-0.1418877	0.5204253
sp|P61204|ARF3_HUMAN	-0.1414852	1.3006523
sp|P61088|UBE2N_HUMAN	-0.1411648	0.45033538
sp|O15067|PUR4_HUMAN	-0.1408625	0.7827403
sp|Q9NZK5|ADA2_HUMAN	-0.1407204	0.45033538
sp|O00712-4|NFIB_HUMAN	-0.1395435	0.656254
sp|O15498-2|YKT6_HUMAN	-0.1387692	0.7827403
sp|Q99426|TBCB_HUMAN	-0.1382484	0.43633315
sp|Q9Y3A3-3|PHOCN_HUMAN	-0.1375942	0.7827403
sp|P42126-2|ECI1_HUMAN	-0.1367989	1.1932944
sp|O75131|CPNE3_HUMAN	-0.1356545	0.5065026
sp|P12081-4|SYHC_HUMAN	-0.1355972	0.43633315
sp|P62263|RS14_HUMAN	-0.1349258	0.35795313
sp|Q10713|MPPA_HUMAN	-0.1346893	0.70235044
sp|P14923|PLAK_HUMAN	-0.1339569	0.7588735

sp|O00391|QSOX1_HUMAN	-0.1329842	1.1932944
sp|P09429|HMGB1_HUMAN	-0.131155	0
sp|P02743|SAMP_HUMAN	-0.1305866	0.21680334
sp|P36551|HEM6_HUMAN	-0.1294289	0
sp|Q9UBQ7|GRHPR_HUMAN	-0.1294022	0.30372584
sp|P34896-2|GLYC_HUMAN	-0.1279163	0
sp|Q14515-2|SPRL1_HUMAN	-0.1277466	0
sp|P0C0S5|H2AZ_HUMAN	-0.1270752	0.656254
sp|P19367-2|HXK1_HUMAN	-0.1270504	0.8941546
sp|P16104|H2AX_HUMAN	-0.126173	0
sp|P51991|ROA3_HUMAN	-0.1251984	0.25789237
sp|P63000-2|RAC1_HUMAN	-0.1245899	0.09894868
sp|P84085|ARF5_HUMAN	-0.1245689	0.656254
sp|P15153|RAC2_HUMAN	-0.1237011	0.656254
sp|P23588|IF4B_HUMAN	-0.1228333	0.5692702
sp|P30566|PUR8_HUMAN	-0.1227951	0.656254
sp|Q16787-3|LAMA3_HUMAN	-0.122551	0.656254
sp|P04083|ANXA1_HUMAN	-0.122488	0.19318101
sp|Q96S97|MYADM_HUMAN	-0.1224232	0.45033538
sp|P62495-2|ERF1_HUMAN	-0.1218433	0.35795313
sp|O75915|PRAF3_HUMAN	-0.1216221	0.7061832
sp|O75503|CLN5_HUMAN	-0.1213989	0.91601294
sp|P60709|ACTB_HUMAN	-0.1209984	0
sp|P28070|PSB4_HUMAN	-0.1203194	0.48757824
sp|P11217|PYGM_HUMAN	-0.1198521	0.656254
sp|Q9Y371-2|SHLB1_HUMAN	-0.1185017	0
sp|Q6DKJ4|NXN_HUMAN	-0.1180649	0.19149946
sp|O00232|PSD12_HUMAN	-0.1178989	0.40256184
sp|E9PAV3|NACAM_HUMAN	-0.1166382	0.7827403
sp|Q9UBC2-2|EP15R_HUMAN	-0.1155148	0.45033538
sp|P09417-2|DHPR_HUMAN	-0.1146145	0.19149946
sp|Q9C0C2|TB182_HUMAN	-0.1143608	1.0634323
sp|Q9H4G4|GAPR1_HUMAN	-0.1138916	0.5204253
sp|Q9Y277-2|VDAC3_HUMAN	-0.1135922	1.0992733
sp|O95466-2|FMNL1_HUMAN	-0.1134176	1.1932944
sp|P01833|PIGR_HUMAN	-0.1132603	0.13973783
sp|Q15366-2|PCBP2_HUMAN	-0.1125736	0.7061832
sp|Q9NZL9|MAT2B_HUMAN	-0.1122246	0.5111962
sp|O14974-3|MYPT1_HUMAN	-0.1115379	0.90036625
sp|O60716-14|CTND1_HUMAN	-0.1115189	0.09894868
sp|P08758|ANXA5_HUMAN	-0.1102486	0.5741409
sp|Q7Z4W1|DCXR_HUMAN	-0.1093521	0.8983557
sp|Q969V3-2|NCLN_HUMAN	-0.1088257	0.5204253
sp|P51692|STA5B_HUMAN	-0.1087952	0.7827403
sp|O43615|TIM44_HUMAN	-0.1083775	0.45033538

sp|P48047|ATPO_HUMAN	-0.1082649	0.37060758
sp|P54578-3|UBP14_HUMAN	-0.1075096	0.17319627
sp|Q8TD55|PKHO2_HUMAN	-0.1068344	0
sp|P21953|ODBB_HUMAN	-0.1057358	0.35795313
sp|P30711|GSTT1_HUMAN	-0.1051559	0.2178309
sp|Q13642-1|FHL1_HUMAN	-0.1048355	0.656254
sp|Q15717-2|ELAV1_HUMAN	-0.104578	0.5204253
sp|Q9UQ80|PA2G4_HUMAN	-0.1040726	0
sp|Q9HC35-2|EMAL4_HUMAN	-0.1022949	0.84879977
sp|P63267|ACTH_HUMAN	-0.101696	0.656254
sp|Q9UJS0-2|CMC2_HUMAN	-0.1015892	1.1932944
sp|P17612|KAPCA_HUMAN	-0.1001759	0.6298893
sp|P50453|SPB9_HUMAN	-0.0992966	0.8059303
sp|P08621-3|RU17_HUMAN	-0.0990315	1.3006523
sp|P24557-2|THAS_HUMAN	-0.0986977	0.7827403
sp|P31937|3HIDH_HUMAN	-0.0984936	0.5204253
sp|Q6UVK1|CSPG4_HUMAN	-0.0983162	0.04454162
sp|O75436|VP26A_HUMAN	-0.098217	0.65625405
sp|O75608-2|LYPA1_HUMAN	-0.0978413	0.7827403
sp|Q16527|CSRP2_HUMAN	-0.0977268	0.7061832
sp|O00483|NDUA4_HUMAN	-0.0976105	0.45033538
sp|P52790|HXK3_HUMAN	-0.0974636	0.33495146
sp|P84090|ERH_HUMAN	-0.0969086	1.2095301
sp|Q9Y5S9-2|RBM8A_HUMAN	-0.0965166	0.09894868
sp|P16070-7|CD44_HUMAN	-0.0963268	0.35795313
sp|Q08945|SSRP1_HUMAN	-0.0960979	0.90036625
sp|P55263|ADK_HUMAN	-0.0948887	0.6298893
sp|Q9Y5K5-2|UCHL5_HUMAN	-0.0948772	0.43633315
sp|Q6DD88|ATLA3_HUMAN	-0.0936184	0.87291557
sp|Q96C23|GALM_HUMAN	-0.0934925	0
sp|P30084|ECHM_HUMAN	-0.0926285	0.84879977
sp|P30520|PURA2_HUMAN	-0.0925522	0.5832693
sp|P30838|AL3A1_HUMAN	-0.0923157	0
sp|P30466|1B18_HUMAN	-0.0919228	0
sp|P61081|UBC12_HUMAN	-0.0907497	0.84879977
sp|P13861|KAP2_HUMAN	-0.0906696	1.1034192
sp|Q969H8|MYDGF_HUMAN	-0.0903549	0.7061832
sp|P30086|PEBP1_HUMAN	-0.0900326	0.90036625
sp|O75083|WDR1_HUMAN	-0.0900173	0.8200627
sp|Q9NQR4|NIT2_HUMAN	-0.0896988	0.84879977
sp|P46940|IQGA1_HUMAN	-0.0893326	0.73115146
sp|P05452|TETN_HUMAN	-0.089138	0.7061832
sp|P11177-3|ODPB_HUMAN	-0.0882645	0.34137914
sp|P16278-2|BGAL_HUMAN	-0.0880718	1.0634323
sp|P48637|GSHB_HUMAN	-0.0880184	1.071201

sp|Q02878|RL6_HUMAN	-0.0880051	0.5204253
sp|P07203|GPX1_HUMAN	-0.0878944	0.0846519
sp|Q13045-2|FLII_HUMAN	-0.0872936	0.44572112
sp|P31930|QCR1_HUMAN	-0.0868149	0.8826702
sp|Q92769|HDAC2_HUMAN	-0.0866928	0.656254
sp|P25398|RS12_HUMAN	-0.0861301	0.84879977
sp|P30512|1A29_HUMAN	-0.0849991	0.5204253
sp|O75112-7|LDB3_HUMAN	-0.0845871	0.91601294
sp|P16152|CBR1_HUMAN	-0.0843391	0.41804093
sp|P09496-2|CLCA_HUMAN	-0.0834827	0.8983557
sp|O15260-2|SURF4_HUMAN	-0.0827379	0.19149946
sp|P23634-8|AT2B4_HUMAN	-0.0826836	0.68888646
sp|P63010-2|AP2B1_HUMAN	-0.0826607	0.45720983
sp|Q9HAV0|GBB4_HUMAN	-0.082653	0
sp|P04632|CPNS1_HUMAN	-0.0823536	1.0241117
sp|Q5K4L6|S27A3_HUMAN	-0.0819635	0.35795313
sp|Q08257|QOR_HUMAN	-0.0813828	0.94599336
sp|P26599-2|PTBP1_HUMAN	-0.080843	0.51870745
sp|O95197-2|RTN3_HUMAN	-0.0808334	0
sp|O00264|PGRC1_HUMAN	-0.0807533	0.70235044
sp|P46926|GNPI1_HUMAN	-0.0801086	0.30372584
sp|P00736|C1R_HUMAN	-0.079567	0.35795313
sp|P36871|PGM1_HUMAN	-0.079319	0
sp|Q86VS8|HOOK3_HUMAN	-0.0780697	0.19149946
sp|P61970|NTF2_HUMAN	-0.0778408	0.91601294
sp|Q92556|ELMO1_HUMAN	-0.0773964	0.1342476
sp|P42330|AK1C3_HUMAN	-0.0773296	1.0992733
sp|Q13884|SNTB1_HUMAN	-0.0772743	0
sp|P42566|EPS15_HUMAN	-0.0763321	0.19149946
sp|Q9Y2B0|CNPY2_HUMAN	-0.0761004	0.35795313
sp|Q9UI12-2|VATH_HUMAN	-0.0751247	0.20467198
sp|Q14108|SCRB2_HUMAN	-0.0741806	0.7827403
sp|Q13492-2|PICAL_HUMAN	-0.0739212	0.09894868
sp|P60842|IF4A1_HUMAN	-0.0729561	1.2954973
sp|Q96HC4|PDLI5_HUMAN	-0.0724926	0
sp|Q9Y639-4|NPTN_HUMAN	-0.0724716	0.19149946
sp|Q13526|PIN1_HUMAN	-0.0723667	0.45033538
sp|P01861|IGHG4_HUMAN	-0.0722504	0.01311907
sp|Q96HN2-2|SAHH3_HUMAN	-0.0717754	0
sp|P49961-6|ENTP1_HUMAN	-0.0711002	0.5204253
sp|Q9BZZ5-5|API5_HUMAN	-0.0709276	0.4075265
sp|O75368|SH3L1_HUMAN	-0.0705757	0.35795313
sp|P50570-2|DYN2_HUMAN	-0.070549	1.0370445
sp|P29400-2|CO4A5_HUMAN	-0.0697517	0
sp|Q13418|ILK_HUMAN	-0.0696125	0.16649151

sp|P33176|KINH_HUMAN	-0.0687332	0.44444597
sp|O75947-2|ATP5H_HUMAN	-0.0673466	0.51870745
sp|Q16629-2|SRSF7_HUMAN	-0.0671806	0.21439649
sp|Q9NUV9|GIMA4_HUMAN	-0.0670357	0.316588
sp|Q02952-2|AKA12_HUMAN	-0.0669861	0
sp|Q96P70|IPO9_HUMAN	-0.0666142	0.5111962
sp|P12004|PCNA_HUMAN	-0.0662708	1.1932944
sp|Q9Y6A4|CFA20_HUMAN	-0.0661659	0.19149946
sp|Q01130-2|SRSF2_HUMAN	-0.0655937	0.09894868
sp|P36873-2|PP1G_HUMAN	-0.0655727	0.45033538
sp|Q9UHX1-6|PUF60_HUMAN	-0.0639801	0.5204253
sp|Q15691|MARE1_HUMAN	-0.0636597	0.20467198
sp|Q16775-2|GLO2_HUMAN	-0.0634441	0.09894868
sp|Q10567-2|AP1B1_HUMAN	-0.063427	0.91601294
sp|Q96BW5-2|PTER_HUMAN	-0.0623436	0
sp|Q9NSE4|SYIM_HUMAN	-0.0622978	0.3005443
sp|O60749|SNX2_HUMAN	-0.0622406	0.35520625
sp|Q16181-2|SEPT7_HUMAN	-0.0620117	0.91458976
sp|P01743|HV146_HUMAN	-0.0619583	0
sp|P63104|1433Z_HUMAN	-0.0617886	0.24295025
sp|P49354-2|FNTA_HUMAN	-0.0616751	1.0485198
sp|Q15075|EEA1_HUMAN	-0.0613651	0.34969378
sp|P78417-2|GSTO1_HUMAN	-0.0610695	0.656254
sp|P06132|DCUP_HUMAN	-0.0610199	0.09750395
sp|P26196|DDX6_HUMAN	-0.0609245	0.2178309
sp|Q86U42-2|PABP2_HUMAN	-0.0608692	0
sp|P08294|SODE_HUMAN	-0.0595818	0.45720983
sp|Q96HE7|ERO1A_HUMAN	-0.0594406	0.09894868
sp|Q14165|MLEC_HUMAN	-0.0583363	0.5204253
sp|P31942-2|HNRH3_HUMAN	-0.0579376	1.3006523
sp|Q06323|PSME1_HUMAN	-0.0578041	0.79861414
sp|O75396|SC22B_HUMAN	-0.0577717	0.39873424
sp|Q9HCB6|SPON1_HUMAN	-0.0575771	0
sp|P00918|CAH2_HUMAN	-0.0569019	1.1932943
sp|P49903-2|SPS1_HUMAN	-0.0568085	0.2178309
sp|O00571-2|DDX3X_HUMAN	-0.0565624	0.2178309
sp|P48444|COPD_HUMAN	-0.0565147	1.0984834
sp|Q9UL46|PSME2_HUMAN	-0.0552387	0.79861414
sp|Q9P2R7-2|SUCB1_HUMAN	-0.0543137	0.19410844
sp|Q99733-2|NP1L4_HUMAN	-0.0537062	0.04454162
sp|Q8NCW5|NNRE_HUMAN	-0.0536461	0.40256184
sp|P26885|FKBP2_HUMAN	-0.0536308	0.19149946
sp|P36542|ATPG_HUMAN	-0.0524874	0
sp|P54727|RD23B_HUMAN	-0.0523491	0.35795313
sp|P04424-2|ARLY_HUMAN	-0.0512848	0.0846519

sp|O75874|IDHC_HUMAN	-0.051281	0.34244674
sp|Q9Y281|COF2_HUMAN	-0.0512218	0
sp|Q13838-2|DX39B_HUMAN	-0.051178	0.21439649
sp|O43747-2|AP1G1_HUMAN	-0.050724	0.35193655
sp|O94905|ERLN2_HUMAN	-0.0501022	0.312067
sp|P33316|DUT_HUMAN	-0.0499935	0
sp|Q9BR76|COR1B_HUMAN	-0.0495338	0
sp|P35998|PRS7_HUMAN	-0.0494537	0.5004855
sp|O00299|CLIC1_HUMAN	-0.0493507	0.07367946
sp|P35268|RL22_HUMAN	-0.0490608	0.09894868
sp|P53602|MVD1_HUMAN	-0.0489502	0.28516325
sp|O60313-10|OPA1_HUMAN	-0.0481873	0.19149946
sp|Q9NZM1-6|MYOF_HUMAN	-0.0481586	0.04711977
sp|Q9NYF8-2|BCLF1_HUMAN	-0.0480137	0.312067
sp|P62304|RUXE_HUMAN	-0.0479012	0
sp|P60174|TPIS_HUMAN	-0.047596	0.95974463
sp|P17655|CAN2_HUMAN	-0.0466652	1.1164713
sp|Q9BTE3-2|MCMBP_HUMAN	-0.046483	0.19149946
sp|P35908|K22E_HUMAN	-0.0455971	0.13882811
sp|P51991-2|ROA3_HUMAN	-0.045332	0
sp|P23284|PPIB_HUMAN	-0.0447521	1.2438905
sp|Q9UMS4|PRP19_HUMAN	-0.044323	0.29066643
sp|Q14203-3|DCTN1_HUMAN	-0.044117	0.45720983
sp|Q9Y3A5|SBDS_HUMAN	-0.0435791	0.48520416
sp|Q13126-2|MTAP_HUMAN	-0.0426674	0.34323573
sp|P08754|GNAI3_HUMAN	-0.04245	0
sp|Q14894|CRYM_HUMAN	-0.0418873	0.19149946
sp|Q13867|BLMH_HUMAN	-0.0407505	0.19149946
sp|P30613-2|KPYR_HUMAN	-0.0406609	0.656254
sp|P10155|RO60_HUMAN	-0.0406332	0.16077396
sp|P34897-2|GLYM_HUMAN	-0.039608	0.48757824
sp|Q13423|NNTM_HUMAN	-0.0394478	0.52598757
sp|P60866-2|RS20_HUMAN	-0.0386524	0.19149946
sp|O00429-6|DNM1L_HUMAN	-0.0386162	0.1500082
sp|O43390-2|HNRPR_HUMAN	-0.0384121	0.5717351
sp|P11387|TOP1_HUMAN	-0.0382118	0.19149946
sp|Q92629-3|SGCD_HUMAN	-0.0381241	0.21439649
sp|P49591|SYSC_HUMAN	-0.038044	0.312067
sp|P18669|PGAM1_HUMAN	-0.0370216	0.8654178
sp|Q99598|TSNAX_HUMAN	-0.0365372	0
sp|P28065-2|PSB9_HUMAN	-0.0359993	0.04454162
sp|P62906|RL10A_HUMAN	-0.0359325	0
sp|Q9UN86-2|G3BP2_HUMAN	-0.0355797	0
sp|Q07955-3|SRSF1_HUMAN	-0.0351944	0.05681695
sp|Q16643-3|DREB_HUMAN	-0.0345497	0.17319627

sp|Q15019-2|SEPT2_HUMAN	-0.034153	0.3653036
sp|Q9NZ01|TECR_HUMAN	-0.0333328	0.21439649
sp|Q13232|NDK3_HUMAN	-0.0332603	0.1342476
sp|Q12931-2|TRAP1_HUMAN	-0.0332375	0.5204253
sp|O94979-10|SC31A_HUMAN	-0.033226	0.1558116
sp|P68104|EF1A1_HUMAN	-0.0326462	0.18414244
sp|P07195|LDHB_HUMAN	-0.0325298	0.1159057
sp|P62857|RS28_HUMAN	-0.0323677	0.2178309
sp|O15372|EIF3H_HUMAN	-0.0323467	0.2178309
sp|P11678|PERE_HUMAN	-0.0322361	0
sp|Q15233|NONO_HUMAN	-0.0308323	0.10138063
sp|Q96QK1|VPS35_HUMAN	-0.0307388	0.29781067
sp|Q92973-2|TNPO1_HUMAN	-0.0300884	0.21439649
sp|P22033|MUTA_HUMAN	-0.0297623	0
sp|Q99829|CPNE1_HUMAN	-0.029417	0.29066643
sp|P55786|PSA_HUMAN	-0.0291805	0.3985966
sp|P29590|PML_HUMAN	-0.0290871	0.09064758
sp|P20073-2|ANXA7_HUMAN	-0.0290318	0.2591514
sp|P62269|RS18_HUMAN	-0.0283661	0.1342476
sp|Q92688-2|AN32B_HUMAN	-0.0278702	0
sp|P00390-2|GSHR_HUMAN	-0.0277252	0.11949348
sp|O94811|TPPP_HUMAN	-0.0276546	0
sp|O00233-2|PSMD9_HUMAN	-0.0276241	0.19149946
sp|Q9UJU6-2|DBNL_HUMAN	-0.0274467	0.1342476
sp|Q9BT78|CSN4_HUMAN	-0.026226	0.06291623
sp|P11766|ADHX_HUMAN	-0.0260468	0.44444597
sp|Q9BUJ2-4|HNRL1_HUMAN	-0.0258369	0.7118947
sp|O75923-11|DYSF_HUMAN	-0.0255775	0
sp|P29350-3|PTN6_HUMAN	-0.0248261	0.04454162
sp|Q7L5N1|CSN6_HUMAN	-0.0238209	0
sp|P43686|PRS6B_HUMAN	-0.0234642	0.6615227
sp|P41250|GARS_HUMAN	-0.0230951	0.12054814
sp|P53041|PPP5_HUMAN	-0.0224152	0.2178309
sp|Q15437|SC23B_HUMAN	-0.022274	0
sp|Q16891-2|MIC60_HUMAN	-0.0221672	0.32654193
sp|Q9ULA0|DNPEP_HUMAN	-0.0220451	0.4490988
sp|O00754-2|MA2B1_HUMAN	-0.0218506	0.21439649
sp|Q0ZGT2-4|NEXN_HUMAN	-0.0217609	0.09851671
sp|P01034|CYTC_HUMAN	-0.0216331	0.45033538
sp|Q969X5-2|ERGI1_HUMAN	-0.0210686	0.2178309
sp|Q8TCD5|NT5C_HUMAN	-0.020997	0.19149946
sp|O60506-3|HNRPQ_HUMAN	-0.0209656	0.1342476
sp|P00491|PNPH_HUMAN	-0.0206528	0.386649
sp|Q12905|ILF2_HUMAN	-0.0201359	0.02756438
sp|Q14498-2|RBM39_HUMAN	-0.0186272	0.14672586

sp|P23497|SP100_HUMAN	-0.0181122	0.45033538
sp|P25705|ATPA_HUMAN	-0.017992	0.25789237
sp|P22059|OSBP1_HUMAN	-0.0178566	0
sp|Q13435|SF3B2_HUMAN	-0.0171127	0
sp|P07741|APT_HUMAN	-0.0166435	0.11612091
sp|P40227|TCPZ_HUMAN	-0.0162869	0.09215608
sp|Q6NVY1|HIBCH_HUMAN	-0.0160599	0.21439649
sp|Q8WUM4|PDC6I_HUMAN	-0.0156002	0.01406953
sp|P54652|HSP72_HUMAN	-0.0148907	0.09894868
sp|Q15393|SF3B3_HUMAN	-0.0146275	0.19410844
sp|P10768|ESTD_HUMAN	-0.0139503	0.09339783
sp|P11166|GTR1_HUMAN	-0.0135994	0
sp|P49755|TMEDA_HUMAN	-0.0133514	0.06291623
sp|P35611-2|ADDA_HUMAN	-0.0132122	0.20467198
sp|P27694|RFA1_HUMAN	-0.0127792	0.17511293
sp|P40429|RL13A_HUMAN	-0.0124302	0.09894868
sp|Q92945|FUBP2_HUMAN	-0.0121479	0.02174174
sp|Q92747|ARC1A_HUMAN	-0.0121269	0
sp|Q9NY33|DPP3_HUMAN	-0.0121155	0.05634482
sp|P27348|1433T_HUMAN	-0.0120277	0.04817784
sp|P04066|FUCO_HUMAN	-0.0117531	0.09894868
sp|Q9BVC6|TM109_HUMAN	-0.0117512	0.09894868
sp|Q9NY15|STAB1_HUMAN	-0.0117493	0.19149946
sp|O43242|PSMD3_HUMAN	-0.0116959	0.05634482
sp|P48735-2|IDHP_HUMAN	-0.0113678	0.79861414
sp|P30153|2AAA_HUMAN	-0.0097198	0.15874931
sp|P17844-2|DDX5_HUMAN	-0.0094337	0.02174174
sp|P27824-2|CALX_HUMAN	-0.0086594	0.03712561
sp|P17980|PRS6A_HUMAN	-0.0074635	0.1329664
sp|P02768|ALBU_HUMAN	-0.0071392	0.09927666
sp|P27797|CALR_HUMAN	-0.0069942	0.0426314
sp|P00492|HPRT_HUMAN	-0.006424	0.21439649
sp|Q9UNH7-2|SNX6_HUMAN	-0.0059757	0
sp|P61313|RL15_HUMAN	-0.0059528	0.45033538
sp|Q16539-2|MK14_HUMAN	-0.0058708	0
sp|Q96M27-3|PRRC1_HUMAN	-0.005785	0.45033538
sp|P61201-2|CSN2_HUMAN	-0.0052242	0
sp|Q9UKV3-5|ACINU_HUMAN	-0.005043	0.21439649
sp|Q96KP4|CNDP2_HUMAN	-0.0044498	0.12274049
sp|P09619|PGFRB_HUMAN	-0.0043831	0.09894868
sp|Q02790|FKBP4_HUMAN	-0.0043354	0.30372584
sp|Q9BXP5-2|SRRT_HUMAN	-0.0042877	0.21439649
sp|P48643|TCPE_HUMAN	-0.0040035	0.16178675
sp|Q9UNE7-2|CHIP_HUMAN	-0.0039272	0
sp|P98082-2|DAB2_HUMAN	-0.0038414	0

sp|P23142|FBLN1_HUMAN	-0.0031528	0
sp|P08133|ANXA6_HUMAN	-0.0030918	0.14408782
sp|P04075|ALDOA_HUMAN	-0.0030708	0.09088494
sp|Q14980-2|NUMA1_HUMAN	-0.0029163	0.10770471
sp|Q15631|TSN_HUMAN	-0.0027332	0.06291623
sp|O75643|U520_HUMAN	-0.002636	0
sp|Q13630|FCL_HUMAN	-0.0024433	0
sp|Q9H9B4|SFXN1_HUMAN	-0.0023117	0.19149946
sp|P30085|KCY_HUMAN	-0.0021105	0.5204253
sp|P49458|SRP09_HUMAN	-0.0015221	0
sp|P06756-3|ITAV_HUMAN	-0.0014992	0.20467198
sp|Q9BRR6-2|ADPGK_HUMAN	-0.0011101	0
sp|Q9BTV4|TMM43_HUMAN	-9.54E-04	0.04454162
sp|O75323|NIPS2_HUMAN	-1.37E-04	0
sp|P37108|SRP14_HUMAN	6.31E-04	0
sp|P22234-2|PUR6_HUMAN	0.00112534	0.11949348
sp|P18085|ARF4_HUMAN	0.00129128	0
sp|Q08211|DHX9_HUMAN	0.0021019	0.24362843
sp|Q96RQ3|MCCA_HUMAN	0.00326729	0.30372584
sp|P55209-2|NP1L1_HUMAN	0.00358582	0
sp|P02549-2|SPTA1_HUMAN	0.0038414	0.1827406
sp|Q06828|FMOD_HUMAN	0.00400734	0.19149946
sp|Q9P2J5-2|SYLC_HUMAN	0.00457001	0
sp|Q9NSD9|SYFB_HUMAN	0.00504303	0.06291623
sp|Q8WVM8|SCFD1_HUMAN	0.00568199	0.04454162
sp|Q96CW1-2|AP2M1_HUMAN	0.00719452	0.06291623
sp|P62888|RL30_HUMAN	0.00719833	0.2178309
sp|Q562R1|ACTBL_HUMAN	0.00748253	0
sp|P30101|PDIA3_HUMAN	0.00777054	0.00597242
sp|P53396-2|ACLY_HUMAN	0.0078392	0.03435687
sp|P50990|TCPQ_HUMAN	0.00803947	0.12618111
sp|Q15029-2|U5S1_HUMAN	0.00949287	0.04153777
sp|P11171-7|41_HUMAN	0.00995255	0.03043297
sp|P25325-2|THTM_HUMAN	0.01003456	0.06291623
sp|P29692-2|EF1D_HUMAN	0.01012421	0.2178309
sp|A0FGR8-2|ESYT2_HUMAN	0.0104847	0
sp|P15121|ALDR_HUMAN	0.01081276	0.06695005
sp|P55084|ECHB_HUMAN	0.01090431	0.37128913
sp|P24666|PPAC_HUMAN	0.01184654	0
sp|P68366-2|TBA4A_HUMAN	0.01196671	0
sp|Q8NBJ5|GT251_HUMAN	0.01199913	0.2178309
sp|Q86UX2-2|ITIH5_HUMAN	0.01248646	0.19149946
sp|Q08380|LG3BP_HUMAN	0.01252937	0.316588
sp|Q15293|RCN1_HUMAN	0.01265526	0.20467198
sp|P30626-2|SORCN_HUMAN	0.01325417	0.14927356

sp|O95861-4|BPNT1_HUMAN	0.0133934	0.1342476
sp|Q9Y6C2|EMIL1_HUMAN	0.01347351	0.08173559
sp|Q8WXF1|PSPC1_HUMAN	0.01347351	0.09339783
sp|P19971|TYPH_HUMAN	0.01401138	0.03228646
sp|P04222|1C03_HUMAN	0.01441574	0
sp|Q9BRF8-2|CPPED_HUMAN	0.01468658	0.09894868
sp|P00441|SODC_HUMAN	0.01516342	0.14672586
sp|O60256-3|KPRB_HUMAN	0.01529121	0.45033538
sp|P13716-2|HEM2_HUMAN	0.01548958	0.34137914
sp|O60610-2|DIAP1_HUMAN	0.01551437	0.30372584
sp|O15173-2|PGRC2_HUMAN	0.01570129	0.09750395
sp|P14868|SYDC_HUMAN	0.01596832	0.02863072
sp|P36776-3|LONM_HUMAN	0.01679039	0.26737198
sp|Q5TDH0-3|DDI2_HUMAN	0.0168438	0.2178309
sp|P62633-3|CNBP_HUMAN	0.01806641	0
sp|Q1KMD3|HNRL2_HUMAN	0.01820183	0.10328458
sp|P22392-2|NDKB_HUMAN	0.01853943	0.7498006
sp|Q9GZT8|NIF3L_HUMAN	0.01866913	0.19149946
sp|P52272-2|HNRPM_HUMAN	0.01896095	0.29692787
sp|P56537|IF6_HUMAN	0.01909828	0.26737198
sp|Q71UM5|RS27L_HUMAN	0.0193615	0
sp|Q9BUF5|TBB6_HUMAN	0.01964378	0
BirA-TRIP6_BirAT6	0.01972485	0
sp|Q8NDH3|PEPL1_HUMAN	0.02028465	0.40256184
sp|Q8WXX5|DNJC9_HUMAN	0.02042389	0
sp|O76003|GLRX3_HUMAN	0.02052879	0
sp|Q14764|MVP_HUMAN	0.0207119	0.0099764
sp|P28062|PSB8_HUMAN	0.02075195	0.1342476
sp|P31943|HNRH1_HUMAN	0.02076531	0
sp|Q96JB5-4|CK5P3_HUMAN	0.02108383	0.2178309
sp|Q9Y3B3|TMED7_HUMAN	0.02114487	0.19149946
sp|P21333-2|FLNA_HUMAN	0.02136612	0.07781328
sp|Q9UBQ5|EIF3K_HUMAN	0.02169991	0.19149946
sp|Q96CN7|ISOC1_HUMAN	0.02174377	0.20467198
sp|Q63ZY3-3|KANK2_HUMAN	0.02177429	0.33495146
sp|Q13425|SNTB2_HUMAN	0.02206039	0
sp|P41091|IF2G_HUMAN	0.02211189	0.4075265
sp|Q6PCB0|VWA1_HUMAN	0.02249527	0.28516325
sp|P27918|PROP_HUMAN	0.0230217	0.45033538
sp|P20674|COX5A_HUMAN	0.02323532	0.2178309
sp|P11940-2|PABP1_HUMAN	0.0233078	0.2178309
sp|P30519|HMOX2_HUMAN	0.02408981	0
sp|P62714|PP2AB_HUMAN	0.02511787	0
sp|P0DOX3|IGD_HUMAN	0.02513886	0.656254
sp|P0CG39|POTEJ_HUMAN	0.0263691	0

sp|Q8NBX0|SCPDL_HUMAN	0.02645111	0.09894868
sp|P28161|GSTM2_HUMAN	0.02708721	0.656254
sp|Q9P1F3|ABRAL_HUMAN	0.02797794	0
sp|P12270|TPR_HUMAN	0.02862358	0.20304409
sp|P17987|TCPA_HUMAN	0.0288353	0.7817134
sp|P07996|TSP1_HUMAN	0.02908707	0.09894868
sp|P07951-3|TPM2_HUMAN	0.02933502	0.40256184
sp|Q13217|DNJC3_HUMAN	0.02974892	0.6298893
sp|O14936-2|CSKP_HUMAN	0.03014755	0.33495146
sp|O95336|6PGL_HUMAN	0.03043556	0.4075265
sp|Q96AG4|LRC59_HUMAN	0.03138733	0.20467198
sp|Q709C8-3|VP13C_HUMAN	0.03237152	0.316588
sp|P00338|LDHA_HUMAN	0.0336647	0.01542275
sp|P39687|AN32A_HUMAN	0.03402519	0.35795313
sp|Q8N163-2|CCAR2_HUMAN	0.03408623	0.5204253
sp|P78371|TCPB_HUMAN	0.03455067	0.53140414
sp|P40763-3|STAT3_HUMAN	0.03530693	0.30372584
sp|O43684-2|BUB3_HUMAN	0.03552628	0.19149946
sp|Q99497|PARK7_HUMAN	0.03566742	0.26737198
sp|Q9BUT1|BDH2_HUMAN	0.03575134	0.04454162
sp|P05388|RLA0_HUMAN	0.03588486	0.61034113
sp|Q16531|DDB1_HUMAN	0.03607559	0.54672104
sp|Q12805-2|FBLN3_HUMAN	0.03607559	0.7214603
sp|B5ME19|EIFCL_HUMAN	0.03650475	0.09894868
sp|P50991-2|TCPD_HUMAN	0.03660584	0.29301754
sp|Q93052|LPP_HUMAN	0.03691101	0.61945313
sp|P14866-2|HNRPL_HUMAN	0.0371418	0
sp|P04406|G3P_HUMAN	0.03726959	0
sp|P55327-3|TPD52_HUMAN	0.03730393	0.19149946
sp|Q99460|PSMD1_HUMAN	0.03751564	0.3563347
sp|P63151-2|2ABA_HUMAN	0.03809738	0.312067
sp|O94973-2|AP2A2_HUMAN	0.03825951	0.28377196
sp|P18077|RL35A_HUMAN	0.03856659	0.30372584
sp|Q14141-2|SEPT6_HUMAN	0.03906441	0.656254
sp|Q14204|DYHC1_HUMAN	0.03932762	0.30084842
sp|P31947|1433S_HUMAN	0.03966522	0
sp|Q13596-2|SNX1_HUMAN	0.040411	0
sp|P00352|AL1A1_HUMAN	0.04094696	0.6864217
sp|Q9NTK5|OLA1_HUMAN	0.0412941	0
sp|P40939|ECHA_HUMAN	0.04155159	0.00415907
sp|P35270|SPRE_HUMAN	0.04191208	0.11949348
sp|Q16401-2|PSMD5_HUMAN	0.04201889	0.0330081
sp|Q9Y5M8|SRPRB_HUMAN	0.04217529	0.25789237
sp|Q9P258|RCC2_HUMAN	0.04280853	0.40256184
sp|P63027|VAMP2_HUMAN	0.04290962	0

sp|Q06278|AOXA_HUMAN	0.04292297	0
sp|P31948|STIP1_HUMAN	0.04299545	0
sp|Q93034|CUL5_HUMAN	0.04318237	0
sp|O43143|DHX15_HUMAN	0.04345512	0.33495146
sp|P61923|COPZ1_HUMAN	0.04438019	0.45033538
sp|P01011|AACT_HUMAN	0.04458618	0.28377232
sp|Q9H8H3|MET7A_HUMAN	0.04465103	0
sp|Q7L1Q6-2|BZW1_HUMAN	0.04481316	0
sp|O94804|STK10_HUMAN	0.04552841	0.19149946
sp|Q9Y262-2|EIF3L_HUMAN	0.04572678	0.10861364
sp|Q13263|TIF1B_HUMAN	0.04595566	0.13973783
sp|P50395|GDIB_HUMAN	0.04655838	0.18887028
sp|P45974-2|UBP5_HUMAN	0.04734421	0.5368848
sp|P08559-2|ODPA_HUMAN	0.04737473	0.12054814
sp|P14314-2|GLU2B_HUMAN	0.0474453	0.47871065
sp|P09104-2|ENOG_HUMAN	0.04744911	0.37061578
sp|P39019|RS19_HUMAN	0.04754067	0.2178309
sp|P11142|HSP7C_HUMAN	0.04762077	0.26694477
sp|Q13363-2|CTBP1_HUMAN	0.04790878	0.19149946
sp|P06744|G6PI_HUMAN	0.04798889	0.22627127
sp|Q12907|LMAN2_HUMAN	0.04886246	0.10861364
sp|Q92888-2|ARHG1_HUMAN	0.04933167	0
sp|O00151|PDLI1_HUMAN	0.05038452	0.12054814
sp|P50502|F10A1_HUMAN	0.05066299	0.33495146
sp|P21589-2|5NTD_HUMAN	0.05078125	0.45033538
sp|P54886-2|P5CS_HUMAN	0.05123901	0
sp|P17174|AATC_HUMAN	0.05263901	0.58516645
sp|P02730|B3AT_HUMAN	0.05330277	0.7187941
sp|O43852-3|CALU_HUMAN	0.05338287	0.30372584
sp|P23229-4|ITA6_HUMAN	0.05359459	0
sp|Q12792-3|TWF1_HUMAN	0.05382156	0
sp|Q9UEY8|ADDG_HUMAN	0.05391693	0.02174174
sp|P52597|HNRPF_HUMAN	0.05508804	0.7217418
sp|Q96DG6|CMBL_HUMAN	0.05581856	0.2178309
sp|P78406|RAE1L_HUMAN	0.05666351	0.312067
sp|P09972|ALDOC_HUMAN	0.05899429	0.26737198
sp|P00558|PGK1_HUMAN	0.05928421	0.01406953
sp|P0DMV9|HS71B_HUMAN	0.05935097	0.27482995
sp|P55265-4|DSRAD_HUMAN	0.05960846	0.43633315
sp|Q12906-4|ILF3_HUMAN	0.06050301	0.9764444
sp|P62995-3|TRA2B_HUMAN	0.06073189	0.7827403
sp|P29144|TPP2_HUMAN	0.06146622	0
sp|P58107|EPIPL_HUMAN	0.06224251	0.01073991
sp|P05166-2|PCCB_HUMAN	0.06230545	0.3005443
sp|Q92900-2|RENT1_HUMAN	0.06300163	0.16649151

sp|P51665|PSMD7_HUMAN	0.06344414	0
sp|P62736|ACTA_HUMAN	0.06345367	0
sp|Q8TAT6-2|NPL4_HUMAN	0.06371117	0.35193655
sp|P62333|PRS10_HUMAN	0.06420898	0.79861414
sp|P07384|CAN1_HUMAN	0.06424904	0.3096179
sp|Q13347|EIF3I_HUMAN	0.0647049	0.20467198
sp|P26641|EF1G_HUMAN	0.06483841	0.22817776
sp|O43294|TGFI1_HUMAN	0.06512451	0.45033538
sp|Q02543|RL18A_HUMAN	0.06532478	0.51870745
sp|P30419-2|NMT1_HUMAN	0.06567955	0.656254
sp|P35637-2|FUS_HUMAN	0.0662899	0.45033538
sp|Q9P2X0-2|DPM3_HUMAN	0.06633186	0.19149946
sp|Q15257-2|PTPA_HUMAN	0.06745338	0.48520416
sp|P14618|KPYM_HUMAN	0.06746864	0.04454162
sp|P05387|RLA2_HUMAN	0.06751251	0.7827403
sp|P62081|RS7_HUMAN	0.0676918	0.2178309
sp|Q9UBG0|MRC2_HUMAN	0.06895065	0.16649151
sp|P55809|SCOT1_HUMAN	0.0693531	0.09894868
sp|P68400|CSK21_HUMAN	0.06963158	0.2178309
sp|Q9Y224|RTRAF_HUMAN	0.07017899	0.26737198
sp|P11498|PYC_HUMAN	0.07024765	0.28516325
sp|O43681|ASNA_HUMAN	0.07034683	0
sp|P07358|CO8B_HUMAN	0.07044411	0.1342476
sp|Q13724-2|MOGS_HUMAN	0.07069969	0.34323573
sp|Q13464|ROCK1_HUMAN	0.0721302	0
sp|A0A0C4DH31|HV118_HUMAN	0.07217789	0
sp|A0AVT1|UBA6_HUMAN	0.07246018	0
sp|P13639|EF2_HUMAN	0.07258034	0.8991083
sp|Q5TFE4|NT5D1_HUMAN	0.0727005	0
sp|O95782-2|AP2A1_HUMAN	0.07276726	0.65625405
sp|Q9UNM6-2|PSD13_HUMAN	0.07379913	0.20467198
sp|Q14258|TRI25_HUMAN	0.07408142	0.7740364
sp|O43776|SYNC_HUMAN	0.07533836	0.37060758
sp|Q8NF91-4|SYNE1_HUMAN	0.07565498	0
sp|O14579|COPE_HUMAN	0.07670975	0.48520416
sp|O14617-4|AP3D1_HUMAN	0.07688141	0
sp|Q13200|PSMD2_HUMAN	0.07711601	0
sp|P49189|AL9A1_HUMAN	0.07730484	0.79074126
sp|P60228|EIF3E_HUMAN	0.07822418	0.45563722
sp|P62249|RS16_HUMAN	0.07849121	0.09894868
sp|P07305-2|H10_HUMAN	0.0786171	0
sp|P11586|C1TC_HUMAN	0.07862473	0.2178309
sp|Q16774|KGUA_HUMAN	0.07881165	0
sp|P48739|PIPNB_HUMAN	0.07889748	0
sp|P08238|HS90B_HUMAN	0.07923126	1.2765499

sp|Q15008-4|PSMD6_HUMAN	0.07954788	0.45033538
sp|Q92696|PGTA_HUMAN	0.07984924	0.37060758
sp|P20039|2B1B_HUMAN	0.08019829	0.656254
sp|Q99832|TCPH_HUMAN	0.08023834	0.95492345
sp|Q8N392|RHG18_HUMAN	0.08029366	0
sp|P08237-3|PFKAM_HUMAN	0.0805378	0.56808305
sp|P13798|ACPH_HUMAN	0.08077622	1.1054204
sp|Q08379|GOGA2_HUMAN	0.08097744	0.7827403
sp|Q16851|UGPA_HUMAN	0.08346367	0.03208591
sp|Q92841-3|DDX17_HUMAN	0.08366394	0.25398457
sp|O14558|HSPB6_HUMAN	0.08368778	0.65625405
sp|O14773|TPP1_HUMAN	0.08375359	0.6298893
sp|P57737-4|CORO7_HUMAN	0.08432007	0.45033538
sp|P42785-2|PCP_HUMAN	0.08449554	1.0301651
sp|Q9NR56-2|MBNL1_HUMAN	0.08587074	0
sp|Q14240-2|IF4A2_HUMAN	0.08611298	0.40256184
sp|Q15102|PA1B3_HUMAN	0.08682442	0.5204253
sp|O60664-4|PLIN3_HUMAN	0.08714867	0.56808305
sp|Q9UBE0|SAE1_HUMAN	0.08721924	0.5204253
sp|O95394-3|AGM1_HUMAN	0.08790588	0
sp|P31150|GDIA_HUMAN	0.08963585	0.17106695
sp|P35527|K1C9_HUMAN	0.08966255	0.57177407
sp|P62244|RS15A_HUMAN	0.09090424	0.45033538
sp|P67775|PP2AA_HUMAN	0.09098625	0
sp|P62140|PP1B_HUMAN	0.091259	0.09894868
sp|Q9H6S3|ES8L2_HUMAN	0.0915823	0.19149946
sp|Q86WV6|STING_HUMAN	0.09177589	0.35795313
sp|P23526|SAHH_HUMAN	0.09204483	0.95888454
sp|P55795|HNRH2_HUMAN	0.09245873	0.35193655
sp|Q9P2T1-2|GMPR2_HUMAN	0.09339905	0.4075265
sp|Q13243-3|SRSF5_HUMAN	0.09363937	0.656254
sp|P47897|SYQ_HUMAN	0.09436417	0.14672586
sp|P12956|XRCC6_HUMAN	0.09550285	0.82809293
sp|P29218|IMPA1_HUMAN	0.09562111	0.02662771
sp|Q96FV2-2|SCRN2_HUMAN	0.09573746	0.45033538
sp|O00170|AIP_HUMAN	0.09653664	1.2095301
sp|P26639-2|SYTC_HUMAN	0.0976162	0.907945
sp|O43865|SAHH2_HUMAN	0.09841442	0.35795313
sp|P43243|MATR3_HUMAN	0.09852219	0.33495146
sp|P52306-4|GDS1_HUMAN	0.09864235	0.5204253
sp|P23368|MAOM_HUMAN	0.10067368	1.2805575
sp|Q9GZM7-3|TINAL_HUMAN	0.10087681	0
sp|P69905|HBA_HUMAN	0.10139465	0.23541966
sp|Q07020-2|RL18_HUMAN	0.10142326	0.35795313
sp|P62277|RS13_HUMAN	0.10164261	1.2095301

sp|Q03519|TAP2_HUMAN	0.10328865	0.656254
sp|P24821-4|TENA_HUMAN	0.10340309	0.76289684
sp|P62913-2|RL11_HUMAN	0.10391617	0.7827403
sp|P12814|ACTN1_HUMAN	0.10469055	0
sp|Q9UK22|FBX2_HUMAN	0.10483551	0
sp|O14980|XPO1_HUMAN	0.10488129	0.2178309
sp|Q9NPH2|INO1_HUMAN	0.10540199	0.9533461
sp|Q9HC38|GLOD4_HUMAN	0.10557556	0.9533461
sp|P15144|AMPN_HUMAN	0.10570717	0.87291557
sp|Q01813-2|PFKAP_HUMAN	0.10647774	0.26737198
sp|P63244|RACK1_HUMAN	0.10650253	0.7187941
sp|Q9H4A4|AMPB_HUMAN	0.10728645	0.52713376
sp|Q9Y3I0|RTCB_HUMAN	0.10778427	0.83646035
sp|P31321|KAP1_HUMAN	0.10791397	0
sp|P55735-2|SEC13_HUMAN	0.10798836	0.6298893
sp|Q14974|IMB1_HUMAN	0.10857773	0.8826702
sp|O14745|NHRF1_HUMAN	0.10873032	1.1505735
sp|P12277|KCRB_HUMAN	0.10874367	0.11075022
sp|Q8IUX7|AEBP1_HUMAN	0.10882568	0.7957244
sp|Q12797-10|ASPH_HUMAN	0.10895729	1.1932943
sp|P09012|SNRPA_HUMAN	0.10974693	0.19149946
sp|O95479|G6PE_HUMAN	0.10991097	0.7588735
sp|P50579-2|MAP2_HUMAN	0.11003876	0.19149946
sp|P12814-2|ACTN1_HUMAN	0.11042023	0.7827403
sp|P07360|CO8G_HUMAN	0.11166954	0.7827403
sp|Q92499|DDX1_HUMAN	0.11171722	0.66088426
sp|P55060-3|XPO2_HUMAN	0.11266327	0.52598757
sp|Q92598-2|HS105_HUMAN	0.1128521	0.656254
sp|P50454|SERPH_HUMAN	0.11289597	1.1791906
sp|P49588-2|SYAC_HUMAN	0.11331368	0.7187941
sp|P56134-3|ATPK_HUMAN	0.11456299	0
sp|P02042|HBD_HUMAN	0.11460877	0.56808305
sp|Q08378|GOGA3_HUMAN	0.11497593	0
sp|P00966|ASSY_HUMAN	0.11553955	0.48757824
sp|O43301|HS12A_HUMAN	0.11652565	1.2095301
sp|O94788-3|AL1A2_HUMAN	0.1166935	0.656254
sp|Q32P44|EMAL3_HUMAN	0.11697578	0.35795313
sp|Q9NQW7-3|XPP1_HUMAN	0.11754608	0.05145278
sp|P09874|PARP1_HUMAN	0.11950111	0.51870745
sp|O75746-2|CMC1_HUMAN	0.12026596	0.80804527
sp|P32455|GBP1_HUMAN	0.12030029	0.3005443
sp|P0CG38|POTEI_HUMAN	0.12036324	0.656254
sp|O75306-2|NDUS2_HUMAN	0.12068939	1.0485198
sp|P01591|IGJ_HUMAN	0.12128067	0.19149946
sp|P34932|HSP74_HUMAN	0.12243652	0.6083562

sp|O43790|KRT86_HUMAN	0.12249756	0.656254
sp|O00487|PSDE_HUMAN	0.12338257	0.19149946
sp|Q14914-2|PTGR1_HUMAN	0.12379646	0.1342476
sp|Q15661|TRYB1_HUMAN	0.12404823	0.8244277
sp|P48147|PPCE_HUMAN	0.12406921	0.3201336
sp|P08865|RSSA_HUMAN	0.12421227	1.0951192
sp|Q8WX93-5|PALLD_HUMAN	0.12454987	0.5204253
sp|Q3LXA3|TKFC_HUMAN	0.12480545	0.8887816
sp|P50995-2|ANX11_HUMAN	0.12741852	1.0916015
sp|Q9Y383-3|LC7L2_HUMAN	0.12790298	1.1505735
sp|P20810-4|ICAL_HUMAN	0.12795639	0.2178309
sp|Q9NSK0-5|KLC4_HUMAN	0.12862778	0.656254
sp|O94776|MTA2_HUMAN	0.12961197	0.6298893
sp|Q00610-2|CLH1_HUMAN	0.13000488	0.9237222
sp|P07951|TPM2_HUMAN	0.13087273	0.7957244
sp|O14744|ANM5_HUMAN	0.13118362	0.45033538
sp|P98095-2|FBLN2_HUMAN	0.13200378	1.0757319
sp|O75340-2|PDCD6_HUMAN	0.13351631	0.7061832
sp|O75964|ATP5L_HUMAN	0.13371277	0.7827403
sp|Q6P2Q9|PRP8_HUMAN	0.13410568	0.5204253
sp|Q9UNS2|CSN3_HUMAN	0.13467789	0.656254
sp|Q9NP79|VTA1_HUMAN	0.13520241	0.91601294
sp|Q9NQG5|RPR1B_HUMAN	0.13522911	0.91601294
sp|Q12882|DPYD_HUMAN	0.13636017	1.1932944
sp|P62195|PRS8_HUMAN	0.13661194	0
sp|P12268|IMDH2_HUMAN	0.13677597	0.19149946
sp|P0DJI8|SAA1_HUMAN	0.13787079	0.19149946
sp|Q15005|SPCS2_HUMAN	0.13838768	1.1505735
sp|P24534|EF1B_HUMAN	0.13842773	0.45033538
sp|P30043|BLVRB_HUMAN	0.13893127	0.8983557
sp|P15374|UCHL3_HUMAN	0.14108086	1.2095301
sp|Q86VP6|CAND1_HUMAN	0.1420784	1.1610394
sp|O43396|TXNL1_HUMAN	0.14236069	0.7827403
sp|P46821|MAP1B_HUMAN	0.14398575	0.69308156
sp|P54802|ANAG_HUMAN	0.1440506	0.19149946
sp|Q8TD06|AGR3_HUMAN	0.14652634	0
sp|Q93009-3|UBP7_HUMAN	0.1489582	1.1932944
sp|Q15274|NADC_HUMAN	0.14901352	0.91601294
sp|O43488|ARK72_HUMAN	0.14978409	0.1342476
sp|P27695|APEX1_HUMAN	0.15175629	0.19149946
sp|P68871|HBB_HUMAN	0.1518364	1.1791906
sp|O76074-2|PDE5A_HUMAN	0.15264416	0.80804527
sp|Q9Y315|DEOC_HUMAN	0.15409279	1.0485198
sp|Q8TD19|NEK9_HUMAN	0.15450478	0.45033538
sp|Q53GG5-2|PDLI3_HUMAN	0.15462303	0.30372584

sp|Q9C0E8-4|LNP_HUMAN	0.15482998	0
sp|P19404|NDUV2_HUMAN	0.15567398	0.312067
sp|Q9BZE9-2|ASPC1_HUMAN	0.15625954	0.656254
sp|O95302-3|FKBP9_HUMAN	0.1583252	0.19149946
sp|Q9BVK6|TMED9_HUMAN	0.16076088	0.91601294
sp|P22102|PUR2_HUMAN	0.1655693	0.8983557
sp|P52888|THOP1_HUMAN	0.16568375	0.45033538
sp|Q7Z4H8|PLGT3_HUMAN	0.16685867	0.656254
sp|Q06210-2|GFPT1_HUMAN	0.17160034	0.95332193
sp|Q9UBS4|DJB11_HUMAN	0.17198372	1.1932944
sp|P62191-2|PRS4_HUMAN	0.17450333	0.5204253
sp|Q13557-12|KCC2D_HUMAN	0.17508698	1.214352
sp|O60825-2|F262_HUMAN	0.17518616	0.656254
sp|Q13885|TBB2A_HUMAN	0.17782974	0
sp|P84243|H33_HUMAN	0.18048954	0
sp|O94919|ENDD1_HUMAN	0.18122292	0
sp|P62829|RL23_HUMAN	0.18126678	0.7827403
sp|P15880|RS2_HUMAN	0.1818428	1.0485198
sp|P68371|TBB4B_HUMAN	0.18252563	0
sp|P35858-2|ALS_HUMAN	0.18305016	0.19149946
sp|Q96D15|RCN3_HUMAN	0.18352413	0.7061832
sp|P14174|MIF_HUMAN	0.18365479	1.1505735
sp|P62917|RL8_HUMAN	0.18475723	1.1791906
sp|P31939|PUR9_HUMAN	0.18611145	0.8706143
sp|Q15185-3|TEBP_HUMAN	0.18625641	0.45033538
sp|P15559-2|NQO1_HUMAN	0.18990898	0.45033538
sp|O43252|PAPS1_HUMAN	0.19108582	1.1505735
sp|O60832|DKC1_HUMAN	0.19112968	1.1932944
sp|P02760|AMBP_HUMAN	0.19190979	0.47871065
sp|O75489|NDUS3_HUMAN	0.19235802	0.8983557
sp|P46781|RS9_HUMAN	0.19284439	0.68594354
sp|P10644|KAP0_HUMAN	0.19339752	0.312067
sp|P14618-2|KPYM_HUMAN	0.19566917	0.7061832
sp|Q8TAQ2-2|SMRC2_HUMAN	0.1957264	0.80804527
sp|Q8TBC4-2|UBA3_HUMAN	0.19703674	1.1932944
sp|P07738|PMGE_HUMAN	0.1993866	0.8983557
sp|Q93084-2|AT2A3_HUMAN	0.19970131	0.45033538
sp|P53992|SC24C_HUMAN	0.20035744	0.6298893
sp|Q16134-3|ETFD_HUMAN	0.20175076	1.1932944
sp|P30837|AL1B1_HUMAN	0.20492554	1.1791906
sp|Q7Z7G0|TARSH_HUMAN	0.20555115	1.1932944
sp|P49721|PSB2_HUMAN	0.2063961	0.19149946
sp|P32456|GBP2_HUMAN	0.20656204	0
sp|P02751-15|FINC_HUMAN	0.20698357	0.656254
sp|Q15436|SC23A_HUMAN	0.20734501	0.1342476

sp|P46459|NSF_HUMAN	0.20743942	0.8983557
sp|P61353|RL27_HUMAN	0.2120781	1.1932944
sp|Q66K74-2|MAP1S_HUMAN	0.21217251	1.1932944
sp|O75828|CBR3_HUMAN	0.21236229	1.1932944
sp|Q6XQN6-2|PNCB_HUMAN	0.212574	0.9847622
sp|Q99538-2|LGMN_HUMAN	0.21278763	1.1932944
sp|Q96AY3|FKB10_HUMAN	0.22075272	0.6070219
sp|O95994|AGR2_HUMAN	0.22115898	1.3006523
sp|P42704|LPPRC_HUMAN	0.22696877	1.0076748
sp|P18583-10|SON_HUMAN	0.23055267	1.1932944
sp|P54136|SYRC_HUMAN	0.24118996	0.84879977
sp|P55145|MANF_HUMAN	0.24543	1.1932944
sp|Q5R3I4|TTC38_HUMAN	0.24547386	0.45033538
sp|P42226|STAT6_HUMAN	0.24783897	0.656254
sp|O14787-2|TNPO2_HUMAN	0.24894047	0.656254
sp|O76011|KRT34_HUMAN	0.256485	0.656254
sp|Q92599-3|SEPT8_HUMAN	0.25716972	1.1932944
sp|Q9UJZ1-2|STML2_HUMAN	0.2575674	0.91601294
sp|P02747|C1QC_HUMAN	0.2587185	1.1932944
sp|P50135|HNMT_HUMAN	0.25972748	0.45033538
sp|P31689-2|DNJA1_HUMAN	0.2666092	1.1932944
sp|Q9Y5P6-2|GMPPB_HUMAN	0.26906586	1.0301651
sp|Q96HY6|DDRGK_HUMAN	0.2733717	0.656254
sp|Q6UW68|TM205_HUMAN	0.2736187	1.1932944
sp|P02763|A1AG1_HUMAN	0.27404594	0.80804527
sp|Q13310-2|PABP4_HUMAN	0.27405357	0.656254
sp|P61803|DAD1_HUMAN	0.27931404	0.656254
sp|P40123-2|CAP2_HUMAN	0.2829666	0
sp|Q04446|GLGB_HUMAN	0.28380966	0.5692702
sp|O14791-2|APOL1_HUMAN	0.2875023	1.1932944
sp|P04062-2|GLCM_HUMAN	0.28876114	0.6070219
sp|P08779|K1C16_HUMAN	0.28970242	0.19149946
sp|Q06033-2|ITIH3_HUMAN	0.29623985	0.7827403
sp|P02746|C1QB_HUMAN	0.29746628	1.1932944
sp|P62854|RS26_HUMAN	0.29994965	1.1932944
sp|P69892|HBG2_HUMAN	0.3071804	0.656254
sp|Q14697|GANAB_HUMAN	0.31155396	1.1932944
sp|P27635|RL10_HUMAN	0.31227493	0.656254
sp|O60701-2|UGDH_HUMAN	0.3138733	0.656254
sp|P55058|PLTP_HUMAN	0.31573105	1.1932944
sp|Q13247-3|SRSF6_HUMAN	0.31818962	0.45033538
sp|P16144-2|ITB4_HUMAN	0.31824398	0.7827403
sp|P12111-4|CO6A3_HUMAN	0.32121277	0.656254
sp|P01619|KV320_HUMAN	0.3221016	0.656254
sp|Q9HD45|TM9S3_HUMAN	0.32566452	1.1932944

sp|O75533|SF3B1_HUMAN	0.32688332	1.1932944
sp|P54577|SYYC_HUMAN	0.3303299	0.7061832
sp|P09871|C1S_HUMAN	0.3389778	0.09894868
sp|P05155-2|IC1_HUMAN	0.33935928	1.1932944
sp|Q7L576|CYFP1_HUMAN	0.3530922	0.6070219
sp|Q05682|CALD1_HUMAN	0.35460186	0.2178309
sp|P52895|AK1C2_HUMAN	0.36023903	0.19149946
sp|P84098|RL19_HUMAN	0.36663246	0.7827403
sp|P23083|HV102_HUMAN	0.3686409	0.656254
sp|P02533|K1C14_HUMAN	0.37032318	0.45033538
sp|P13928|ANXA8_HUMAN	0.37114716	1.1932944
sp|Q15149-3|PLEC_HUMAN	0.37446213	0.656254
sp|Q15323|K1H1_HUMAN	0.3802147	0.656254
sp|A0A0B4J1X8|HV343_HUMAN	0.38097763	0.656254
sp|Q9HCN8|SDF2L_HUMAN	0.3959732	1.1932944
sp|Q9BX97|PLVAP_HUMAN	0.40327835	1.1932944
sp|Q96IJ6-2|GMPPA_HUMAN	0.40448952	1.1932944
sp|O94855-2|SC24D_HUMAN	0.40451622	1.1932944
sp|A0A0C4DH29|HV103_HUMAN	0.40841484	0.656254
sp|P08185|CBG_HUMAN	0.41263962	1.1932944
sp|P61018-2|RAB4B_HUMAN	0.4138813	0.656254
sp|Q15063-2|POSTN_HUMAN	0.42027092	0.656254
sp|Q15063-3|POSTN_HUMAN	0.46837616	1.1932944
sp|P46779-2|RL28_HUMAN	0.47846222	1.2095301
sp|P62910|RL32_HUMAN	0.4838543	1.1932944
sp|P06310|KV230_HUMAN	0.48698616	0.656254
sp|P47895|AL1A3_HUMAN	0.4906149	0.656254
sp|Q9BWS9-3|CHID1_HUMAN	0.49173355	0.656254
sp|Q08170|SRSF4_HUMAN	0.5091982	0.656254
sp|Q15046|SYK_HUMAN	0.55125046	0.656254
sp|Q687X5|STEA4_HUMAN	0.5844059	1.1932944
sp|P35080-2|PROF2_HUMAN	0.6588135	0.656254
sp|A0A0C4DH25|KVD20_HUMAN	0.6743965	0.656254
sp|P48741|HSP77_HUMAN	0.7063198	0.656254
sp|P46977|STT3A_HUMAN	0.7264805	1.1932944
sp|P35542|SAA4_HUMAN	0.72792625	1.1932944
sp|A0A0C4DH38|HV551_HUMAN	0.7502117	1.1932944
sp|P01782|HV309_HUMAN	0.75719833	0.656254
sp|P01624|KV315_HUMAN	0.7973633	1.1932944
sp|P14207|FOLR2_HUMAN	0.85708046	0.656254
sp|P01834|IGKC_HUMAN	0.8639355	1.1932944
sp|Q03591|FHR1_HUMAN	0.8907776	1.1932944
sp|A0A075B6P5|KV228_HUMAN	0.91993904	0.656254
sp|P0DP03|HV335_HUMAN	0.9296398	1.1932944
sp|P01780|HV307_HUMAN	0.9698124	0.656254

sp|Q13938-4|CAYP1_HUMAN	1.0082302	0.656254
sp|P02461|CO3A1_HUMAN	1.0347652	1.1932944
sp|Q13976-2|KGP1_HUMAN	1.081934	0.656254
sp|A0A0C4DH41|HV461_HUMAN	1.139904	0.656254
sp|P0C0L4|CO4A_HUMAN	1.151926	1.1932944
sp|P00738|HPT_HUMAN	1.2329216	0.656254
sp|P0C0L5|CO4B_HUMAN	1.5534401	1.1932944
