# Supplementary material for: Peptidylarginine deiminase 2 citrullinates MZB1 and promotes the secretion of IgM and IgA
Source: Front Immunol. 2023 Nov 29;14:1290585. doi: 10.3389/fimmu.2023.1290585 (PMC10716219; doi:10.3389/fimmu.2023.1290585)
Supplement: Supplementary file 7 [file DataSheet_7.pdf]

Supplemental Table 7: IPF1 vs controls

Accession #	Fold Change	p value (-log10)
sp|P13760|2B14_HUMAN	-1.2406063	1.6960104
sp|P05164-3|PERM_HUMAN	-0.964447	9.925412
sp|Q15109-10|RAGE_HUMAN	-0.7791596	1.6997428
sp|P50897-2|PPT1_HUMAN	-0.730835	1.6960104
sp|P59666|DEF3_HUMAN	-0.7227097	2.1818786
sp|P41218|MNDA_HUMAN	-0.704237	1.6604291
sp|P07339|CATD_HUMAN	-0.6734581	7.2183566
sp|P22748|CAH4_HUMAN	-0.6611595	2.1818786
sp|P62805|H4_HUMAN	-0.6488762	6.3204336
sp|P31949|S10AB_HUMAN	-0.6433296	2.6576471
sp|P60903|S10AA_HUMAN	-0.6258736	1.6960104
sp|P80188|NGAL_HUMAN	-0.600193	2.1800864
sp|P08174-3|DAF_HUMAN	-0.557827	1.6960104
sp|P12821-2|ACE_HUMAN	-0.5410538	2.3143692
sp|P10253|LYAG_HUMAN	-0.5363674	3.5908275
sp|P24158|PRTN3_HUMAN	-0.5284939	1.6960104
sp|Q13510-2|ASAH1_HUMAN	-0.527401	4.2164803
sp|P02786|TFR1_HUMAN	-0.5253449	3.4802763
sp|P84103-2|SRSF3_HUMAN	-0.5169964	1.6295799
sp|P09467|F16P1_HUMAN	-0.49646	2.9903855
sp|P07305|H10_HUMAN	-0.4863873	1.6960104
sp|P61626|LYSC_HUMAN	-0.4641457	3.1266599
sp|P18428|LBP_HUMAN	-0.4521103	1.3815327
sp|P07858|CATB_HUMAN	-0.4483414	2.400925
sp|P20160|CAP7_HUMAN	-0.4368668	1.4104178
sp|P08311|CATG_HUMAN	-0.4357147	2.6576471
sp|P02769|ALBU_BOVIN	-0.4114609	6.879847
sp|Q01469|FABP5_HUMAN	-0.3950081	3.364204
sp|P09758|TACD2_HUMAN	-0.3827324	1.3635377
sp|P50895|BCAM_HUMAN	-0.3640003	4.285869
sp|P01011|AACT_HUMAN	-0.3496418	5.3834457
sp|Q9UM07|PADI4_HUMAN	-0.3349419	1.4104178
sp|Q9Y624|JAM1_HUMAN	-0.3313084	1.6092666
sp|P05091|ALDH2_HUMAN	-0.3143368	2.066954
sp|P26006-1|ITA3_HUMAN	-0.3118286	1.4595377
sp|P21810|PGS1_HUMAN	-0.3114948	4.0641108
sp|Q6PIU2-2|NCEH1_HUMAN	-0.3086166	1.4278674
sp|Q13740-2|CD166_HUMAN	-0.2976551	1.7565529
sp|P05023-4|AT1A1_HUMAN	-0.2892971	2.1937726
sp|P35527|K1C9_HUMAN	-0.2881165	3.9267747
sp|P07686|HEXB_HUMAN	-0.2813873	1.9509854
sp|Q9UGT4|SUSD2_HUMAN	-0.278225	1.4050349
sp|P43490|NAMPT_HUMAN	-0.2754631	2.3935137

sp|P06865|HEXA_HUMAN	-0.2738838	1.3815327
sp|Q8NBQ5|DHB11_HUMAN	-0.2654171	2.1800864
sp|P04179-4|SODM_HUMAN	-0.2587547	1.8444856
sp|P35908|K22E_HUMAN	-0.2524643	6.3250384
sp|P55268|LAMB2_HUMAN	-0.2415085	2.7087963
sp|O96009|NAPSA_HUMAN	-0.2304745	1.4278674
sp|P10412|H14_HUMAN	-0.2224007	1.5054473
sp|P07237|PDIA1_HUMAN	-0.2118359	2.207884
sp|P13645|K1C10_HUMAN	-0.203516	5.2685075
sp|Q7Z406-2|MYH14_HUMAN	-0.2029724	1.716333
sp|Q9UHG3|PCYOX_HUMAN	-0.2002239	1.3213577
sp|O95810|CAVN2_HUMAN	-0.1599655	1.515701
sp|P04264|K2C1_HUMAN	-0.0816689	3.7654986
sp|P01833|PIGR_HUMAN	0.1484375	1.8842831
sp|P19971|TYPH_HUMAN	0.16301918	1.6661506
sp|P13647|K2C5_HUMAN	0.17661476	1.9153107
sp|P22105-1|TENX_HUMAN	0.18163681	3.472686
sp|P15088|CBPA3_HUMAN	0.19852448	1.4278674
sp|P02730|B3AT_HUMAN	0.20829391	2.2535503
sp|P35555|FBN1_HUMAN	0.22310448	2.6576467
sp|P62269|RS18_HUMAN	0.22734642	1.6604291
sp|P02549-2|SPTA1_HUMAN	0.24033737	3.9846432
sp|P21333-2|FLNA_HUMAN	0.24609375	2.5771337
sp|P00450|CERU_HUMAN	0.24838829	4.514301
sp|P15880|RS2_HUMAN	0.25235367	2.9807727
sp|P46782|RS5_HUMAN	0.25242996	1.6624602
sp|P35749-4|MYH11_HUMAN	0.2607174	4.386148
sp|P02749|APOH_HUMAN	0.2667389	2.6997027
sp|P04217|A1BG_HUMAN	0.26778793	1.6046445
sp|P23381|SYWC_HUMAN	0.27154922	4.281241
sp|Q9NZ08-2|ERAP1_HUMAN	0.271595	1.3567923
sp|Q9UL46|PSME2_HUMAN	0.2733612	1.9509854
sp|P49411|EFTU_HUMAN	0.2747059	1.5609726
sp|P28838-2|AMPL_HUMAN	0.2819538	1.5092005
sp|Q9H223|EHD4_HUMAN	0.2869892	1.5799892
sp|Q15746-3|MYLK_HUMAN	0.29870224	1.6295882
sp|P05156|CFAI_HUMAN	0.30791664	1.6832623
sp|Q13263|TIF1B_HUMAN	0.31331635	1.5555226
sp|Q06323|PSME1_HUMAN	0.31850243	3.622396
sp|Q15063-3|POSTN_HUMAN	0.32172394	3.2009048
sp|P0DOX8|IGL1_HUMAN	0.3222828	2.6576471
sp|Q14315-2|FLNC_HUMAN	0.322834	6.797499
sp|P62424|RL7A_HUMAN	0.32369232	2.3305986
sp|Q96CX2|KCD12_HUMAN	0.32461166	1.5444825
sp|P00751|CFAB_HUMAN	0.33095932	5.9832516

sp|P23456|Trypsin	0.3378563	2.3388627
sp|P02774-3|VTDB_HUMAN	0.3423252	6.1391983
sp|P48735|IDHP_HUMAN	0.34759903	1.6046445
sp|P18124|RL7_HUMAN	0.35020065	2.817951
sp|P10909-5|CLUS_HUMAN	0.35831642	3.0019732
sp|P13671|CO6_HUMAN	0.35947418	1.6405568
sp|P07451|CAH3_HUMAN	0.37070084	1.3080103
sp|O00151|PDLI1_HUMAN	0.378994	2.12598
sp|P09874|PARP1_HUMAN	0.37918663	1.4751658
sp|P00740-2|FA9_HUMAN	0.38594055	1.8444856
sp|P43652|AFAM_HUMAN	0.3885479	4.3267694
sp|Q9UMS6-2|SYNP2_HUMAN	0.39120674	1.7703108
sp|P67936-2|TPM4_HUMAN	0.39198685	1.6997428
sp|P01031|CO5_HUMAN	0.3943634	3.288843
sp|Q9BUT1|BDH2_HUMAN	0.3968544	1.560883
sp|P08603|CFAH_HUMAN	0.40758514	11.101238
sp|Q12805-2|FBLN3_HUMAN	0.40948868	4.1295223
sp|P01019|ANGT_HUMAN	0.41119385	2.6576471
sp|P00734|THRB_HUMAN	0.41279602	3.8058946
sp|P62266|RS23_HUMAN	0.4130993	1.6960104
sp|P27635|RL10_HUMAN	0.41572762	1.6960104
sp|P46781|RS9_HUMAN	0.41714096	3.4656596
sp|P61247|RS3A_HUMAN	0.41906357	2.2323174
sp|P11171-2|41_HUMAN	0.42181015	1.6960104
sp|P17661|DESM_HUMAN	0.42771912	13.275162
sp|P02790|HEMO_HUMAN	0.43245506	6.2466874
sp|P00966|ASSY_HUMAN	0.43442917	1.5569298
sp|P54136|SYRC_HUMAN	0.4409752	1.8595492
sp|P04196|HRG_HUMAN	0.44153595	2.987788
sp|P01008|ANT3_HUMAN	0.44491005	4.9172535
sp|P04792|HSPB1_HUMAN	0.44491577	5.805384
sp|Q8N2S1-3|LTBP4_HUMAN	0.44492912	2.702259
sp|P21291|CSRP1_HUMAN	0.44965363	3.658668
sp|P36578|RL4_HUMAN	0.4525032	3.4003167
sp|Q9NZN4|EHD2_HUMAN	0.4532814	7.040581
sp|P69891|HBG1_HUMAN	0.45342827	2.6576471
sp|P01042-2|KNG1_HUMAN	0.4542427	3.9849691
sp|P01023|A2MG_HUMAN	0.4583931	12.067423
sp|P61353|RL27_HUMAN	0.46709824	1.6960104
sp|P11277-3|SPTB1_HUMAN	0.46987915	3.1092827
sp|Q05682|CALD1_HUMAN	0.47275925	1.7590232
sp|P02511|CRYAB_HUMAN	0.48090363	1.6960104
sp|P62913|RL11_HUMAN	0.4866333	1.6960104
sp|Q14624-2|ITIH4_HUMAN	0.49407387	4.530363
sp|P29466-2|CASP1_HUMAN	0.49465942	1.6960104

sp|P62241|RS8_HUMAN	0.50107193	2.4829872
sp|P50225|ST1A1_HUMAN	0.5039253	1.6960104
sp|P02787|TRFE_HUMAN	0.50450516	14.592862
sp|Q9NR12-2|PDLI7_HUMAN	0.5208168	2.441399
sp|P0DOY2|IGLC2_HUMAN	0.5208397	2.1818786
sp|P25311|ZA2G_HUMAN	0.5265789	4.051346
sp|P62736|ACTA_HUMAN	0.53154564	3.545398
sp|P01876|IGHA1_HUMAN	0.53406143	2.9310203
sp|P00747|PLMN_HUMAN	0.54634476	4.461395
sp|P01861|IGHG4_HUMAN	0.54769325	2.6576471
sp|Q14767|LTBP2_HUMAN	0.5610771	7.2792387
sp|P26373|RL13_HUMAN	0.5648861	2.6576471
sp|P05546|HEP2_HUMAN	0.5679283	2.4982266
sp|Q9NZU5|LMCD1_HUMAN	0.57027054	5.6000037
sp|P84098|RL19_HUMAN	0.5884857	1.4104178
sp|P29622|KAIN_HUMAN	0.5897064	2.1818786
sp|P04003|C4BPA_HUMAN	0.5950031	4.367871
sp|P52943|CRIP2_HUMAN	0.60881805	1.9647322
sp|P30043|BLVRB_HUMAN	0.61424637	1.7590232
sp|P15428-5|PGDH_HUMAN	0.6161003	1.6960104
sp|Q01995|TAGL_HUMAN	0.63765335	6.264521
sp|P32969|RL9_HUMAN	0.6384182	1.6960104
sp|P32455|GBP1_HUMAN	0.6443615	3.9417877
sp|P00738-2|HPT_HUMAN	0.65237045	5.417897
sp|P00918|CAH2_HUMAN	0.66083527	4.876761
sp|P19823|ITIH2_HUMAN	0.66166306	5.869831
sp|P21266|GSTM3_HUMAN	0.67009354	3.4551415
sp|P19827|ITIH1_HUMAN	0.67022324	1.827201
sp|Q96PD5-2|PGRP2_HUMAN	0.67350197	1.6960104
sp|P61254|RL26_HUMAN	0.6896839	2.1818786
sp|P42224-2|STAT1_HUMAN	0.68989754	2.977915
sp|P32119|PRDX2_HUMAN	0.7154255	4.964395
sp|P07738|PMGE_HUMAN	0.7240448	2.1818786
sp|P02766|TTHY_HUMAN	0.7544441	1.3815327
sp|P09493-9|TPM1_HUMAN	0.764061	1.6960104
sp|P0DOX5|IGG1_HUMAN	0.7719612	4.509013
sp|P27169|PON1_HUMAN	0.7856159	1.6960104
sp|P04114|APOB_HUMAN	0.84806633	11.703171
sp|P48668|K2C6C_HUMAN	0.8779774	2.4829872
sp|Q9UBX5|FBLN5_HUMAN	0.91659164	4.051346
sp|P06727|APOA4_HUMAN	0.94646835	7.2183566
sp|P02042|HBD_HUMAN	0.97548103	3.5908275
sp|P00915|CAH1_HUMAN	1.1656532	6.3204336
sp|P68871|HBB_HUMAN	1.1691666	4.051346
sp|P02647|APOA1_HUMAN	1.2948189	13.420564

sp|P02652|APOA2_HUMAN	1.3353729	2.5644996
sp|P69905|HBA_HUMAN	1.3428421	3.5908275
sp|P19013|K2C4_HUMAN	1.6130104	2.6576471
sp|P13646-3|K1C13_HUMAN	2.6910114	2.6576471
sp|P04229|2B11_HUMAN	-3.0526848	1.1932944
sp|P13761|2B17_HUMAN	-1.8588295	0.656254
sp|P08246|ELNE_HUMAN	-1.4635429	1.1932944
sp|P30453|1A34_HUMAN	-1.444376	0.656254
sp|P49913|CAMP_HUMAN	-1.1465454	1.1932944
sp|Q31612|1B73_HUMAN	-1.0801373	0
sp|P10316|1A69_HUMAN	-1.0369205	0.656254
sp|Q01518-2|CAP1_HUMAN	-0.9289055	0.656254
sp|P25815|S100P_HUMAN	-0.8826485	0.656254
sp|P61224|RAP1B_HUMAN	-0.8797817	0.656254
sp|O00757|F16P2_HUMAN	-0.8717346	0.7827403
sp|Q16777|H2A2C_HUMAN	-0.6185513	0.656254
sp|P13498|CY24A_HUMAN	-0.5984535	1.1932944
sp|P25774|CATS_HUMAN	-0.5674019	0.7827403
sp|Q07075|AMPE_HUMAN	-0.5594025	0.7061832
sp|O95716|RAB3D_HUMAN	-0.5234032	0.656254
sp|P37235|HPCL1_HUMAN	-0.5232678	0.656254
sp|Q13231-2|CHIT1_HUMAN	-0.5085526	0.6070219
sp|Q14956-2|GPNMB_HUMAN	-0.4913063	1.1932944
sp|P06703|S10A6_HUMAN	-0.4888897	0.5204253
sp|P38159|RBMX_HUMAN	-0.4632702	1.1932944
sp|P04908|H2A1B_HUMAN	-0.4234314	0.656254
sp|Q14011|CIRBP_HUMAN	-0.4224787	0.7827403
sp|P13284|GILT_HUMAN	-0.4155703	1.1932944
sp|O95994|AGR2_HUMAN	-0.4152622	1.0634323
sp|P55290-4|CAD13_HUMAN	-0.4109879	0.7061832
sp|P15559-3|NQO1_HUMAN	-0.4086494	0.7061832
sp|P30491|1B53_HUMAN	-0.405592	0.19149946
sp|Q01518|CAP1_HUMAN	-0.402668	0.656254
sp|Q9NNW7|TRXR2_HUMAN	-0.4008408	1.2095301
sp|P05109|S10A8_HUMAN	-0.3782997	0.4075265
sp|P04839|CY24B_HUMAN	-0.3774128	0.45033538
sp|P51608|MECP2_HUMAN	-0.377388	1.1932944
sp|O43615|TIM44_HUMAN	-0.375638	0.45033538
sp|P05534|1A24_HUMAN	-0.3620033	0
sp|P99999|CYC_HUMAN	-0.355978	0.65625405
sp|Q15056-2|IF4H_HUMAN	-0.3371124	0.7827403
sp|Q6P4A8|PLBL1_HUMAN	-0.3354626	0.7061832
sp|P02795|MT2_HUMAN	-0.3352308	0.19149946
sp|P17213|BPI_HUMAN	-0.3241692	0
sp|P00167-2|CYB5_HUMAN	-0.3207378	0.91601294

sp|P12694-2|ODBA_HUMAN	-0.3185663	0.7827403
sp|Q9UFN0|NPS3A_HUMAN	-0.3179894	0.80804527
sp|P63218|GBG5_HUMAN	-0.3128929	0.7588735
sp|Q9NPY3|C1QR1_HUMAN	-0.3117733	0.5692702
sp|P20702|ITAX_HUMAN	-0.3112698	0.312067
sp|P09429|HMGB1_HUMAN	-0.3111649	0.45033538
sp|Q13751|LAMB3_HUMAN	-0.310051	0.6298893
sp|P62314|SMD1_HUMAN	-0.3078966	0.7827403
sp|Q08722-2|CD47_HUMAN	-0.3068657	0.7061832
sp|P11215-2|ITAM_HUMAN	-0.3041248	0.90036625
sp|P05107|ITB2_HUMAN	-0.3019733	1.1386513
sp|P16671-4|CD36_HUMAN	-0.3005905	0.40256184
sp|P43304|GPDM_HUMAN	-0.297081	0.5896263
sp|A5A3E0|POTEF_HUMAN	-0.2957726	0
sp|P04066|FUCO_HUMAN	-0.2945776	1.1932944
sp|P28676|GRAN_HUMAN	-0.2920857	0.45563722
sp|P61421|VA0D1_HUMAN	-0.2900829	0.19410844
sp|P30838|AL3A1_HUMAN	-0.2880173	0.7827403
sp|Q9NZA1-2|CLIC5_HUMAN	-0.2822132	0.8059303
sp|Q9BXM0|PRAX_HUMAN	-0.2804794	0
sp|Q53FA7|QORX_HUMAN	-0.278616	0.19149946
sp|Q9NZN3|EHD3_HUMAN	-0.2772903	0.09894868
sp|Q9HB40|RISC_HUMAN	-0.2760563	1.1932944
sp|Q04941|PLP2_HUMAN	-0.2739372	0
sp|P24557-2|THAS_HUMAN	-0.2711182	0.45033538
sp|P26447|S10A4_HUMAN	-0.2661018	0.09339783
sp|Q9UBR2|CATZ_HUMAN	-0.265131	0
sp|P62714|PP2AB_HUMAN	-0.2627335	0.656254
sp|P06702|S10A9_HUMAN	-0.2596664	0.16077396
sp|P35241|RADI_HUMAN	-0.2596378	0.6115017
sp|Q9H8H3|MET7A_HUMAN	-0.2594223	0
sp|P67809|YBOX1_HUMAN	-0.2561007	0.7827403
sp|Q03135-2|CAV1_HUMAN	-0.2523365	0
sp|Q92522|H1X_HUMAN	-0.2507172	0.45033538
sp|P11686-2|PSPC_HUMAN	-0.2504635	0
sp|P23229-4|ITA6_HUMAN	-0.2502422	0
sp|P20292|AL5AP_HUMAN	-0.248766	1.1932944
sp|P17931|LEG3_HUMAN	-0.247366	0.30372584
sp|Q92973-2|TNPO1_HUMAN	-0.2414017	0.5204253
sp|P26440|IVD_HUMAN	-0.2414017	0.1342476
sp|P02462|CO4A1_HUMAN	-0.2399168	1.0634323
sp|Q9H2U2-2|IPYR2_HUMAN	-0.2360859	0.19149946
sp|P10523|ARRS_HUMAN	-0.2300301	0.656254
sp|O15296|LX15B_HUMAN	-0.2268829	0
sp|P11279|LAMP1_HUMAN	-0.2268791	1.1932944

sp|Q9Y2Q5|LTOR2_HUMAN	-0.2259483	0.45033538
sp|P11047|LAMC1_HUMAN	-0.2249184	1.0584712
sp|Q9HDC9|APMAP_HUMAN	-0.2246056	0.694615
sp|P05026-2|AT1B1_HUMAN	-0.2236481	0.84879977
sp|P09622|DLDH_HUMAN	-0.2200928	0.22362706
sp|P22894|MMP8_HUMAN	-0.219595	0
sp|P12236|ADT3_HUMAN	-0.219183	0.19149946
sp|P16284-3|PECA1_HUMAN	-0.218401	1.1371965
sp|Q9BXP5-5|SRRT_HUMAN	-0.2180214	0.40256184
sp|P40926|MDHM_HUMAN	-0.2152767	1.0728569
sp|P49756|RBM25_HUMAN	-0.2130871	0.45033538
sp|Q8TD06|AGR3_HUMAN	-0.2119484	0
sp|P07099|HYEP_HUMAN	-0.21064	0.8524118
sp|P21980|TGM2_HUMAN	-0.2082539	0.7152521
sp|Q96AP7|ESAM_HUMAN	-0.2065353	0
sp|P02750|A2GL_HUMAN	-0.2042446	0.26737198
sp|Q96EE3-1|SEH1_HUMAN	-0.2035618	0
sp|Q9Y2J2-4|E41L3_HUMAN	-0.2031326	0
sp|O15230|LAMA5_HUMAN	-0.2029724	0.7118947
sp|P01920|DQB1_HUMAN	-0.2022743	0
sp|Q13488|VPP3_HUMAN	-0.2021322	0.43633315
sp|P61960|UFM1_HUMAN	-0.2006588	0.45033538
sp|P13686|PPA5_HUMAN	-0.1991882	0.5204253
sp|P51659|DHB4_HUMAN	-0.1984463	0.7588735
sp|O95837|GNA14_HUMAN	-0.1980686	0
sp|P12429|ANXA3_HUMAN	-0.1974239	0.01290371
sp|P55327-3|TPD52_HUMAN	-0.1969528	0.5204253
sp|A0A0B4J2D5|GAL3B_HUMAN	-0.1959209	0.06291623
sp|Q9UBW8|CSN7A_HUMAN	-0.1951809	0.09894868
sp|P30048-2|PRDX3_HUMAN	-0.1935196	0.20467198
sp|P07355|ANXA2_HUMAN	-0.1927452	0.01178462
sp|P43353-2|AL3B1_HUMAN	-0.1915665	0
sp|P35914|HMGCL_HUMAN	-0.189949	0.09894868
sp|Q96AG4|LRC59_HUMAN	-0.1897831	1.1791906
sp|P40121|CAPG_HUMAN	-0.1882629	0
sp|P00352|AL1A1_HUMAN	-0.1872463	0.11367966
sp|P11310-2|ACADM_HUMAN	-0.1862106	0.01551361
sp|Q04837|SSBP_HUMAN	-0.185833	0.35795313
sp|P61106|RAB14_HUMAN	-0.1851044	0.81595695
sp|P11717|MPRI_HUMAN	-0.1840725	0.35795313
sp|P09668|CATH_HUMAN	-0.183548	0.3005443
sp|P14780|MMP9_HUMAN	-0.1835365	0.4075265
sp|P22307-8|NLTP_HUMAN	-0.1817551	0.9533461
sp|P0CG47|UBB_HUMAN	-0.1813164	0.95332193
sp|Q01130-2|SRSF2_HUMAN	-0.1806221	0.7061832

sp|P61604|CH10_HUMAN	-0.1799355	0.40256184
sp|Q99715-4|COCA1_HUMAN	-0.1790695	0.25789237
sp|Q99436|PSB7_HUMAN	-0.1788321	0
sp|P20340-2|RAB6A_HUMAN	-0.1760368	0
sp|Q9NVJ2|ARL8B_HUMAN	-0.1760101	0
sp|P08236-2|BGLR_HUMAN	-0.1732636	0.35193655
sp|O00159|MYO1C_HUMAN	-0.1731663	0.82648736
sp|P07996|TSP1_HUMAN	-0.1725655	0.65625405
sp|Q6NY19-2|KANK3_HUMAN	-0.1715584	0.2178309
sp|P61026|RAB10_HUMAN	-0.1710463	0
sp|Q99536|VAT1_HUMAN	-0.1708355	0.21680334
sp|Q13813-2|SPTN1_HUMAN	-0.1707592	0
sp|Q13555-7|KCC2G_HUMAN	-0.1700821	0
sp|Q92820|GGH_HUMAN	-0.1700258	0.45033538
sp|P05362|ICAM1_HUMAN	-0.1684799	0.5818213
sp|Q13228-4|SBP1_HUMAN	-0.1676388	0.306704
sp|P14543-2|NID1_HUMAN	-0.1659756	0.3096179
sp|P09110|THIK_HUMAN	-0.1651955	0.633381
sp|P63220|RS21_HUMAN	-0.1641922	0
sp|Q15833-2|STXB2_HUMAN	-0.1639099	0.09894868
sp|P42126-2|ECI1_HUMAN	-0.1625214	0
sp|Q07955-2|SRSF1_HUMAN	-0.1599922	1.0475321
sp|P04080|CYTB_HUMAN	-0.159565	0
sp|P01903|DRA_HUMAN	-0.1584892	0.4546338
sp|P30044|PRDX5_HUMAN	-0.1575623	0.29762787
sp|P28074|PSB5_HUMAN	-0.1566639	0
sp|P16435|NCPR_HUMAN	-0.1558456	0.01044662
sp|O75695|XRP2_HUMAN	-0.15555	0
sp|Q14254|FLOT2_HUMAN	-0.1542568	0.2699497
sp|P33121-3|ACSL1_HUMAN	-0.1540756	0.03367973
sp|Q9NYL9|TMOD3_HUMAN	-0.1538582	0.4075265
sp|Q13813-3|SPTN1_HUMAN	-0.1536636	0
sp|Q9GZM7-3|TINAL_HUMAN	-0.1532898	0
sp|Q02252-2|MMSA_HUMAN	-0.1532135	0.12054814
sp|O75131|CPNE3_HUMAN	-0.1523438	0
sp|Q96TC7|RMD3_HUMAN	-0.1517277	0.95332193
sp|P51149|RAB7A_HUMAN	-0.1498852	0.1804695
sp|P30040|ERP29_HUMAN	-0.14958	0.7740364
sp|A0A0B4J1X8|HV343_HUMAN	-0.1491127	0.656254
sp|P09960|LKHA4_HUMAN	-0.14888	0.869505
sp|P27824-2|CALX_HUMAN	-0.1483135	0.368128
sp|P62820|RAB1A_HUMAN	-0.1478024	0
sp|P23634-2|AT2B4_HUMAN	-0.1476955	0.03367973
sp|Q9H0U4|RAB1B_HUMAN	-0.1468487	0.1342476
sp|P10599-2|THIO_HUMAN	-0.1460133	0.1342476

sp|P07910-2|HNRPC_HUMAN	-0.145998	0.7498006
sp|Q969H8|MYDGF_HUMAN	-0.1453133	0.2178309
sp|P00403|COX2_HUMAN	-0.1441727	0
sp|Q9Y3D6|FIS1_HUMAN	-0.1434031	0.2178309
sp|Q8TC12-2|RDH11_HUMAN	-0.1429596	0
sp|P13804-2|ETFA_HUMAN	-0.1427193	0
sp|P35754|GLRX1_HUMAN	-0.1425228	0
sp|P06737-2|PYGL_HUMAN	-0.1413193	0.19818917
sp|O00186|STXB3_HUMAN	-0.1412916	0
sp|P16278-2|BGAL_HUMAN	-0.1403198	0.35795313
sp|O75390|CISY_HUMAN	-0.1394444	0.05621411
sp|O94760|DDAH1_HUMAN	-0.1385021	0
sp|Q00839|HNRPU_HUMAN	-0.1378307	0.45033535
sp|P01009|A1AT_HUMAN	-0.1371861	0.09165865
sp|Q13636|RAB31_HUMAN	-0.1358948	0.09894868
sp|P15144|AMPN_HUMAN	-0.1350517	0.45563722
sp|Q13011|ECH1_HUMAN	-0.1345844	0.4075265
sp|P49748-2|ACADV_HUMAN	-0.133915	0.04487669
sp|P29401-2|TKT_HUMAN	-0.1338005	0.01727676
sp|O75348|VATG1_HUMAN	-0.1326294	0
sp|O14786|NRP1_HUMAN	-0.1320419	0
sp|Q09666|AHNK_HUMAN	-0.131897	0.5848035
sp|P06748-3|NPM_HUMAN	-0.1310711	0
sp|P52790|HXK3_HUMAN	-0.1309929	0
sp|P41250|GARS_HUMAN	-0.1306114	0.12054814
sp|P53992|SC24C_HUMAN	-0.1282024	0.26737198
sp|O43760-2|SNG2_HUMAN	-0.1273441	0
sp|P61019|RAB2A_HUMAN	-0.1237545	0
sp|P42167-2|LAP2B_HUMAN	-0.1233902	0.6509351
sp|P62873|GBB1_HUMAN	-0.1232948	0
sp|O00391|QSOX1_HUMAN	-0.1225529	0
sp|Q16698-2|DECR_HUMAN	-0.1224766	0.34244674
sp|P54802|ANAG_HUMAN	-0.1218462	0.2178309
sp|Q9H0R4|HDHD2_HUMAN	-0.1207686	0
sp|Q9ULV4-3|COR1C_HUMAN	-0.1199436	0
sp|P09601|HMOX1_HUMAN	-0.1197605	0.04707252
sp|P00505|AATM_HUMAN	-0.1197109	0.15517515
sp|P61225|RAP2B_HUMAN	-0.11936	0
sp|Q16853|AOC3_HUMAN	-0.1190605	0.13882811
sp|P38606-2|VATA_HUMAN	-0.1183853	0.08684197
sp|P05556|ITB1_HUMAN	-0.1175995	0.56076217
sp|P10515|ODP2_HUMAN	-0.1169262	0.02662771
sp|Q14165|MLEC_HUMAN	-0.1160774	0.35795313
sp|P09619|PGFRB_HUMAN	-0.1150608	0.40256184
sp|P36543-2|VATE1_HUMAN	-0.1132469	0.2178309

sp|P17655|CAN2_HUMAN	-0.1122952	0.44116515
sp|P52209-2|6PGD_HUMAN	-0.1107845	0.20449738
sp|Q16630-3|CPSF6_HUMAN	-0.1104717	0.35795313
sp|P53041|PPP5_HUMAN	-0.1104603	0.06842031
sp|P60953|CDC42_HUMAN	-0.1094742	0
sp|O00468-7|AGRIN_HUMAN	-0.1092157	0
sp|O15143|ARC1B_HUMAN	-0.1088924	0
sp|Q07507|DERM_HUMAN	-0.1086731	0.34137914
sp|Q10567-2|AP1B1_HUMAN	-0.1073608	0.06291623
sp|P23786|CPT2_HUMAN	-0.1063919	0.0846519
sp|Q15907|RB11B_HUMAN	-0.1063881	0.2207585
sp|Q9HAV0|GBB4_HUMAN	-0.1058464	0
sp|P0DP25|CALM3_HUMAN	-0.1058311	0.06842031
sp|Q01105-2|SET_HUMAN	-0.1056252	0.09894868
sp|O15400-2|STX7_HUMAN	-0.1048622	0.25789237
sp|O15247|CLIC2_HUMAN	-0.1047096	0.03367973
sp|P13987-2|CD59_HUMAN	-0.1044598	0
sp|P12109|CO6A1_HUMAN	-0.1042366	1.0589138
sp|P04004|VTNC_HUMAN	-0.1040764	0.04454162
sp|P27487|DPP4_HUMAN	-0.1040535	0.28516325
sp|P62995-3|TRA2B_HUMAN	-0.1039429	0.19149946
sp|Q9UBQ0-2|VPS29_HUMAN	-0.1037617	0
sp|P35579|MYH9_HUMAN	-0.1037426	0.6473716
sp|P06733|ENOA_HUMAN	-0.1030598	0.00699881
sp|P10809|CH60_HUMAN	-0.102562	0.00415907
sp|P62070-4|RRAS2_HUMAN	-0.1022739	0
sp|P25325-2|THTM_HUMAN	-0.1016769	0
sp|Q8WWI1-5|LMO7_HUMAN	-0.1013374	0.29066643
sp|P50440-3|GATM_HUMAN	-0.0997219	0.19149946
sp|P50452|SPB8_HUMAN	-0.0982819	0.19149946
sp|Q9NRN5|OLFL3_HUMAN	-0.0978012	0.10017801
sp|Q9Y2X3|NOP58_HUMAN	-0.0977135	0.19149946
sp|P37837|TALDO_HUMAN	-0.0977097	0.01700861
sp|O95831-3|AIFM1_HUMAN	-0.0972366	0
sp|Q9HCC0-2|MCCB_HUMAN	-0.0971584	0
sp|P68036-3|UB2L3_HUMAN	-0.0969391	0
sp|Q02318|CP27A_HUMAN	-0.0964985	0
sp|Q53H82|LACB2_HUMAN	-0.096426	0
sp|P04440|DPB1_HUMAN	-0.0957661	0
sp|P00338|LDHA_HUMAN	-0.0953922	0.00697919
sp|O75955-2|FLOT1_HUMAN	-0.095089	0
sp|Q9BWD1|THIC_HUMAN	-0.0949154	0
sp|Q99598|TSNAX_HUMAN	-0.0945139	0.09894868
sp|Q92542|NICA_HUMAN	-0.0935097	0.09894868
sp|Q8IWB7|WDFY1_HUMAN	-0.0915222	0

sp|P05141|ADT2_HUMAN	-0.0896912	0.26737198
sp|P49591|SYSC_HUMAN	-0.0893822	0.09851671
sp|P61088|UBE2N_HUMAN	-0.0886612	0
sp|P01871-2|IGHM_HUMAN	-0.0885735	0
sp|Q9HD89|RETN_HUMAN	-0.0883827	0.45033538
sp|Q9Y2A7-2|NCKP1_HUMAN	-0.0880928	0
sp|Q03135|CAV1_HUMAN	-0.0877075	0
sp|Q15582|BGH3_HUMAN	-0.0876236	0.18948217
sp|P13796|PLSL_HUMAN	-0.0873718	0.30111516
sp|P20774|MIME_HUMAN	-0.0870686	0.04454162
sp|P08575-10|PTPRC_HUMAN	-0.0869026	0.16702904
sp|P21912|SDHB_HUMAN	-0.0867672	0.09894868
sp|P22897|MRC1_HUMAN	-0.086525	0.49008074
sp|Q6UWY5|OLFL1_HUMAN	-0.0862637	0.6255959
sp|O94832|MYO1D_HUMAN	-0.0861588	0
sp|Q92552-2|RT27_HUMAN	-0.0859299	0
sp|P60033|CD81_HUMAN	-0.0857048	0
sp|Q14344|GNA13_HUMAN	-0.085331	0.06291623
sp|O60437|PEPL_HUMAN	-0.084507	0.10950502
sp|Q9BRF8|CPPED_HUMAN	-0.083683	0
sp|P34896-2|GLYC_HUMAN	-0.0836372	0.06291623
sp|Q3KQV9|UAP1L_HUMAN	-0.0831947	0.45033538
sp|Q14533|KRT81_HUMAN	-0.0829735	0
sp|Q13451|FKBP5_HUMAN	-0.0827141	0
sp|Q5JWF2-2|GNAS1_HUMAN	-0.0825138	0.09339783
sp|Q13496|MTM1_HUMAN	-0.0823851	0.19149946
sp|Q9H0W9-2|CK054_HUMAN	-0.0819626	0
sp|O75439|MPPB_HUMAN	-0.0815945	0.28516325
sp|Q9UKV3-5|ACINU_HUMAN	-0.0801353	0.40256184
sp|A6NMZ7|CO6A6_HUMAN	-0.0799179	0.37277833
sp|P30740|ILEU_HUMAN	-0.0782814	0
sp|P24752|THIL_HUMAN	-0.0778885	0.31423286
sp|Q15149-9|PLEC_HUMAN	-0.0775204	0
sp|P84090|ERH_HUMAN	-0.0771618	0
sp|P36551|HEM6_HUMAN	-0.0764732	0
sp|Q00169|PIPNA_HUMAN	-0.0761051	0
sp|P08572|CO4A2_HUMAN	-0.0752525	0.26737198
sp|P31040|SDHA_HUMAN	-0.0752449	0.09851671
sp|P04075|ALDOA_HUMAN	-0.0750008	0.17235917
sp|P62258|1433E_HUMAN	-0.0746002	0.19959533
sp|P21397|AOFA_HUMAN	-0.0743008	0
sp|Q9NZM1-3|MYOF_HUMAN	-0.0740051	0
sp|P15170-2|ERF3A_HUMAN	-0.072731	0
sp|P98160|PGBM_HUMAN	-0.0722332	0.19525568
sp|Q08380|LG3BP_HUMAN	-0.0722008	0.74639726

sp|P51648-2|AL3A2_HUMAN	-0.0721149	0.04454162
sp|Q16658|FSCN1_HUMAN	-0.0719204	0.02114108
sp|P30626-2|SORCN_HUMAN	-0.0703297	0
sp|Q9ULA0|DNPEP_HUMAN	-0.0700417	0.56808305
sp|Q7Z6Z7-2|HUWE1_HUMAN	-0.0695915	0
sp|P21281|VATB2_HUMAN	-0.0694923	0.04817784
sp|Q04760-2|LGUL_HUMAN	-0.0692368	0
sp|P51148-2|RAB5C_HUMAN	-0.0690575	0.04454162
sp|P20700|LMNB1_HUMAN	-0.0686913	0.6666559
sp|Q9Y5S9-2|RBM8A_HUMAN	-0.0685825	0.09894868
sp|O94811|TPPP_HUMAN	-0.0684357	0
sp|P31323|KAP3_HUMAN	-0.0681038	0.312067
sp|P06753|TPM3_HUMAN	-0.0680828	0
sp|Q9NTX5-3|ECHD1_HUMAN	-0.0670776	0
sp|P55263|ADK_HUMAN	-0.0668936	0
sp|P30084|ECHM_HUMAN	-0.0665455	0
sp|Q13217|DNJC3_HUMAN	-0.06633	0.17319627
sp|P38646|GRP75_HUMAN	-0.0647202	0.18005672
sp|Q96P70|IPO9_HUMAN	-0.0646896	0
sp|P12110|CO6A2_HUMAN	-0.0641747	0.02228302
sp|Q8NFW8|NEUA_HUMAN	-0.0640183	0
sp|P50148|GNAQ_HUMAN	-0.0639076	0.06291623
sp|P09382|LEG1_HUMAN	-0.0635986	0.16702904
sp|Q9Y394-2|DHRS7_HUMAN	-0.0634041	0
sp|O75923-15|DYSF_HUMAN	-0.0626011	0
sp|P61158|ARP3_HUMAN	-0.0624542	0.11648214
sp|O43175|SERA_HUMAN	-0.0621948	0.08077049
sp|P02743|SAMP_HUMAN	-0.0620651	0
sp|P13489|RINI_HUMAN	-0.0619679	0.1007388
sp|P37802|TAGL2_HUMAN	-0.0618477	0
sp|Q06830|PRDX1_HUMAN	-0.0618248	0
sp|O75643|U520_HUMAN	-0.0615997	0
sp|P09871|C1S_HUMAN	-0.0613422	0.30372584
sp|P63167|DYL1_HUMAN	-0.0612755	0
sp|Q9NSE4|SYIM_HUMAN	-0.061142	0.16214149
sp|P00387-3|NB5R3_HUMAN	-0.0607147	0
sp|P78417|GSTO1_HUMAN	-0.0603256	0.04153777
sp|P02545|LMNA_HUMAN	-0.0602779	0.01551361
sp|P51636-2|CAV2_HUMAN	-0.0601501	0.19149946
sp|P11216|PYGB_HUMAN	-0.0597534	0.50252277
sp|Q15084-5|PDIA6_HUMAN	-0.0592365	0.15332921
sp|Q07157|ZO1_HUMAN	-0.0591297	0.46260658
sp|Q96S97|MYADM_HUMAN	-0.0590744	0
sp|Q01082|SPTB2_HUMAN	-0.0584106	0.21964629
sp|P61586|RHOA_HUMAN	-0.0582533	0.09894868

sp|P06396-2|GELS_HUMAN	-0.057045	0
sp|P55072|TERA_HUMAN	-0.0570164	0.06431034
sp|P24666-2|PPAC_HUMAN	-0.0566854	0
sp|Q92597|NDRG1_HUMAN	-0.0564308	0.06291623
sp|P63104|1433Z_HUMAN	-0.0563698	0.2909411
sp|Q9NV96-3|CC50A_HUMAN	-0.0556564	0
sp|P21964-2|COMT_HUMAN	-0.0547447	0
sp|P11678|PERE_HUMAN	-0.0543556	0
sp|P30519|HMOX2_HUMAN	-0.0538006	0.06291623
sp|P50213|IDH3A_HUMAN	-0.0533485	0
sp|P23141-3|EST1_HUMAN	-0.0532227	0.37313595
sp|P54920|SNAA_HUMAN	-0.0528355	0
sp|Q9UKK9|NUDT5_HUMAN	-0.052618	0
sp|P08648|ITA5_HUMAN	-0.0525246	0
sp|Q14112-2|NID2_HUMAN	-0.0525169	0.34137914
sp|Q6NZI2|CAVN1_HUMAN	-0.0523338	0.09139413
sp|P31948|STIP1_HUMAN	-0.0521946	0
sp|Q07954|LRP1_HUMAN	-0.0520191	0.12618111
sp|Q10589-2|BST2_HUMAN	-0.0513134	0
sp|O95373|IPO7_HUMAN	-0.050518	0
sp|P05166-2|PCCB_HUMAN	-0.0504761	0.01343579
sp|P27797|CALR_HUMAN	-0.0498962	0.16702904
sp|P07948-2|LYN_HUMAN	-0.0498924	0
sp|P45880-2|VDAC2_HUMAN	-0.0497055	0
sp|P28070|PSB4_HUMAN	-0.0493889	0.14672586
sp|Q86X76-2|NIT1_HUMAN	-0.0492573	0
sp|Q16363-2|LAMA4_HUMAN	-0.0486145	0.01178462
sp|P13639|EF2_HUMAN	-0.0482101	0.00226418
sp|O95202|LETM1_HUMAN	-0.0474777	0.25789237
sp|P67775-2|PP2AA_HUMAN	-0.04739	0
sp|Q14651|PLSI_HUMAN	-0.0465183	0
sp|Q13126-2|MTAP_HUMAN	-0.0465031	0
sp|Q6YN16|HSDL2_HUMAN	-0.046442	0.05483269
sp|O43148-2|MCES_HUMAN	-0.0460644	0
sp|Q9BUJ2-4|HNRL1_HUMAN	-0.0459309	0
sp|Q6P1N9|TATD1_HUMAN	-0.0459042	0
sp|Q14240-2|IF4A2_HUMAN	-0.0456944	0.21439649
sp|P55010|IF5_HUMAN	-0.0451126	0
sp|Q6P587-3|FAHD1_HUMAN	-0.0450954	0
sp|P47985|UCRI_HUMAN	-0.0450745	0.35795313
sp|O75947-2|ATP5H_HUMAN	-0.0446911	0.04454162
sp|Q96N66-3|MBOA7_HUMAN	-0.0420036	0
sp|P14625|ENPL_HUMAN	-0.0410385	0.40633747
sp|Q709C8-2|VP13C_HUMAN	-0.040535	0
sp|Q04826|1B40_HUMAN	-0.0403748	0

sp|P52788|SPSY_HUMAN	-0.0387383	0.7061832
sp|Q15029-2|U5S1_HUMAN	-0.0383949	0.1159057
sp|Q8TAT6-2|NPL4_HUMAN	-0.0383291	0
sp|P04406|G3P_HUMAN	-0.0376205	0.05919517
sp|P29218|IMPA1_HUMAN	-0.0374184	0.14672586
sp|Q13938-4|CAYP1_HUMAN	-0.0371094	0
sp|P06744|G6PI_HUMAN	-0.0367031	0
sp|Q02218-2|ODO1_HUMAN	-0.0366545	0
sp|P42765|THIM_HUMAN	-0.0365047	0.09124502
sp|P39060-1|COIA1_HUMAN	-0.0362549	0.1063047
sp|O43813|LANC1_HUMAN	-0.0358181	0
sp|P51991|ROA3_HUMAN	-0.0355682	0
sp|P01780|HV307_HUMAN	-0.034977	0
sp|P84077|ARF1_HUMAN	-0.0345726	0.06291623
sp|Q99829|CPNE1_HUMAN	-0.0344753	0
sp|Q6P179|ERAP2_HUMAN	-0.0344505	0
sp|Q13043|STK4_HUMAN	-0.0336437	0
sp|O60825-2|F262_HUMAN	-0.0335293	0
sp|P12830|CADH1_HUMAN	-0.0333481	0
sp|Q8IV08|PLD3_HUMAN	-0.0332432	0.26737198
sp|Q32MZ4-3|LRRF1_HUMAN	-0.0322456	0
sp|P61981|1433G_HUMAN	-0.0321674	0
sp|P49407-2|ARRB1_HUMAN	-0.032156	0
sp|P53634|CATC_HUMAN	-0.0319462	0.06291623
sp|P62879|GBB2_HUMAN	-0.031168	0
sp|Q8N335|GPD1L_HUMAN	-0.0308933	0.30372584
sp|P39687|AN32A_HUMAN	-0.0296268	0
sp|P21796|VDAC1_HUMAN	-0.0288467	0
sp|Q86Y82|STX12_HUMAN	-0.0287571	0
sp|P25685-2|DNJB1_HUMAN	-0.0285034	0
sp|Q15599-2|NHRF2_HUMAN	-0.0283756	0
sp|P60228|EIF3E_HUMAN	-0.0282784	0.30372584
sp|Q5T440|CAF17_HUMAN	-0.0280495	0
sp|Q13308-6|PTK7_HUMAN	-0.0278797	0.2207585
sp|Q96I99|SUCB2_HUMAN	-0.0275784	0
sp|P22626|ROA2_HUMAN	-0.0273552	0.02575658
sp|Q03252|LMNB2_HUMAN	-0.0272121	0.23348935
sp|Q14108-2|SCRB2_HUMAN	-0.0271034	0
sp|Q08211|DHX9_HUMAN	-0.0269127	0.02421166
sp|Q6NUK1-2|SCMC1_HUMAN	-0.0264111	0.04454162
sp|P11021|BIP_HUMAN	-0.0259285	0.00246678
sp|Q9UDY2-3|ZO2_HUMAN	-0.0249672	0.356152
sp|Q8WXF1|PSPC1_HUMAN	-0.0247688	0
sp|O75521-2|ECI2_HUMAN	-0.0243588	0
sp|P49458|SRP09_HUMAN	-0.0238876	0

sp|Q03154-4|ACY1_HUMAN	-0.0236797	0
sp|Q9HB07|MYG1_HUMAN	-0.0231934	0
sp|P49755|TMEDA_HUMAN	-0.0228252	0
sp|P63261|ACTG_HUMAN	-0.0224628	0
sp|P16219|ACADS_HUMAN	-0.0220871	0.06291623
sp|O94919|ENDD1_HUMAN	-0.0220394	0.06291623
sp|Q12905|ILF2_HUMAN	-0.0220013	0
sp|P0C0L4|CO4A_HUMAN	-0.0219994	0
sp|P17174|AATC_HUMAN	-0.0219307	0.02174174
sp|Q12931-2|TRAP1_HUMAN	-0.0215836	0
sp|Q00325-2|MPCP_HUMAN	-0.0211639	0.26737198
sp|Q53GQ0|DHB12_HUMAN	-0.0210953	0
sp|Q5EBM0-3|CMPK2_HUMAN	-0.020834	0.09894868
sp|P19367-3|HXK1_HUMAN	-0.0207596	0.01100408
sp|P14618-3|KPYM_HUMAN	-0.0205765	0
sp|P49189|AL9A1_HUMAN	-0.0204849	0
sp|Q15121|PEA15_HUMAN	-0.0194016	0.09894868
sp|Q9BVG4|PBDC1_HUMAN	-0.019043	0
sp|P00367|DHE3_HUMAN	-0.0188484	0.01551361
sp|P06899|H2B1J_HUMAN	-0.0186653	0
sp|P60983|GMFB_HUMAN	-0.0184937	0
sp|P49065|ALBU_RABIT	-0.018362	0
sp|P60660-2|MYL6_HUMAN	-0.0178776	0
sp|Q15369-2|ELOC_HUMAN	-0.0178604	0
sp|P07942|LAMB1_HUMAN	-0.0168514	0.19852771
sp|P78347-2|GTF2I_HUMAN	-0.0167751	0.19149946
sp|P61601|NCALD_HUMAN	-0.0160408	0
sp|P26641|EF1G_HUMAN	-0.0159149	0.18378536
sp|O60234|GMFG_HUMAN	-0.0152721	0.04454162
sp|Q9Y315|DEOC_HUMAN	-0.015029	0
sp|P09543-2|CN37_HUMAN	-0.0150108	0.09750395
sp|P84243|H33_HUMAN	-0.0147266	0.2178309
sp|Q9UJ70-2|NAGK_HUMAN	-0.0146255	0
sp|Q9BS26|ERP44_HUMAN	-0.014389	0
sp|Q15836|VAMP3_HUMAN	-0.013237	0
sp|O95571|ETHE1_HUMAN	-0.0127563	0.26546443
sp|Q9UI12-2|VATH_HUMAN	-0.0127296	0.11949348
sp|P51649-2|SSDH_HUMAN	-0.0123329	0.09750395
sp|P62993|GRB2_HUMAN	-0.0122833	0
sp|Q9NYU2-2|UGGG1_HUMAN	-0.0121841	0.02858656
sp|Q92598-2|HS105_HUMAN	-0.0115299	0
sp|P23246|SFPQ_HUMAN	-0.0110989	0
sp|P22234-2|PUR6_HUMAN	-0.0109787	0
sp|P55196-5|AFAD_HUMAN	-0.0109177	0
sp|Q8NBJ5|GT251_HUMAN	-0.0108986	0.1558116

sp|P09211|GSTP1_HUMAN	-0.0108585	0
sp|P27105|STOM_HUMAN	-0.0107918	0.13973783
sp|Q9Y277-2|VDAC3_HUMAN	-0.010498	0
sp|P12270|TPR_HUMAN	-0.0103874	0
sp|P63151-2|2ABA_HUMAN	-0.0100594	0
sp|Q9UL25|RAB21_HUMAN	-0.0096455	0.06291623
sp|Q16629-4|SRSF7_HUMAN	-0.0093174	0
sp|Q9NZ01|TECR_HUMAN	-0.0092688	0
sp|P30085|KCY_HUMAN	-0.0092335	0
sp|Q96BM9|ARL8A_HUMAN	-0.0091305	0
sp|P04350|TBB4A_HUMAN	-0.0089035	0
sp|Q9P265|DIP2B_HUMAN	-0.0087757	0
sp|P48643|TCPE_HUMAN	-0.0087528	0.11004404
sp|P16070-16|CD44_HUMAN	-0.008606	0
sp|P62937|PPIA_HUMAN	-0.0083885	0
sp|Q8NBJ7|SUMF2_HUMAN	-0.0081024	0
sp|O95197-2|RTN3_HUMAN	-0.0070229	0
sp|Q96KP4|CNDP2_HUMAN	-0.0067081	0
sp|Q5K4L6-2|S27A3_HUMAN	-0.0065098	0
sp|P06576|ATPB_HUMAN	-0.0063133	0.05779712
sp|Q16762|THTR_HUMAN	-0.0057831	0
sp|O00264|PGRC1_HUMAN	-0.0050354	0
sp|P35221|CTNA1_HUMAN	-0.0046501	0.2237808
sp|P26368-2|U2AF2_HUMAN	-0.0044727	0
sp|P48449-3|ERG7_HUMAN	-0.004406	0.09339783
sp|Q02818|NUCB1_HUMAN	-0.0042934	0.16293353
sp|Q96FV2-2|SCRN2_HUMAN	-0.0037746	0
sp|P34897-3|GLYM_HUMAN	-0.0034027	0
sp|P05452|TETN_HUMAN	-0.0032272	0.09894868
sp|P25787|PSA2_HUMAN	-0.0030937	0.20467198
sp|P14207|FOLR2_HUMAN	-0.0029993	0
sp|O75608-2|LYPA1_HUMAN	-0.0020676	0
sp|Q86UP2-4|KTN1_HUMAN	-0.0019856	0
sp|P63027|VAMP2_HUMAN	-0.0019302	0
sp|Q02083-2|NAAA_HUMAN	-0.0015221	0
sp|O00423-3|EMAL1_HUMAN	-0.0013332	0.09894868
sp|O94826|TOM70_HUMAN	-0.0012903	0.11949348
sp|P98095-2|FBLN2_HUMAN	-0.0011559	0.12026574
sp|Q96HD1-2|CREL1_HUMAN	-0.0011292	0
sp|P48960-2|CD97_HUMAN	-7.46E-04	0
sp|P33316|DUT_HUMAN	-1.72E-04	0
sp|P23142|FBLN1_HUMAN	0.00154495	0
sp|Q9Y2B0|CNPY2_HUMAN	0.0015564	0
sp|P61081|UBC12_HUMAN	0.00163078	0
sp|P56537|IF6_HUMAN	0.00174713	0.5111962

sp|P22033|MUTA_HUMAN	0.00255966	0
sp|P36957|ODO2_HUMAN	0.00260735	0
sp|O60701|UGDH_HUMAN	0.00270462	0
sp|P10768|ESTD_HUMAN	0.00285149	0
sp|Q15661|TRYB1_HUMAN	0.003582	0
sp|O94973-2|AP2A2_HUMAN	0.00362206	0
sp|Q9NX46|ARHL2_HUMAN	0.00428391	0
sp|P29590|PML_HUMAN	0.00470924	0
sp|Q9H008|LHPP_HUMAN	0.00517082	0
sp|Q14697|GANAB_HUMAN	0.00540924	0
sp|Q92734-2|TFG_HUMAN	0.00577354	0
sp|Q13557-10|KCC2D_HUMAN	0.00579071	0
sp|Q9Y6E0|STK24_HUMAN	0.00579071	0.19149946
sp|P16615|AT2A2_HUMAN	0.00582886	0
sp|P35606-2|COPB2_HUMAN	0.00583649	0
sp|O43776|SYNC_HUMAN	0.00604439	0
sp|P25398|RS12_HUMAN	0.00634289	0
sp|O43684-2|BUB3_HUMAN	0.00646973	0
sp|P43034|LIS1_HUMAN	0.00681686	0
sp|P09651-3|ROA1_HUMAN	0.00699043	0
sp|Q96HE7|ERO1A_HUMAN	0.00708199	0.09894868
sp|P49327|FAS_HUMAN	0.00709724	0.01335202
sp|P80303-2|NUCB2_HUMAN	0.00715446	0
sp|P00488|F13A_HUMAN	0.00724411	0.02575658
sp|Q7Z4W1|DCXR_HUMAN	0.00785637	0
sp|P50995-2|ANX11_HUMAN	0.00831223	0.22817776
sp|O15031|PLXB2_HUMAN	0.00862122	0.4075265
sp|Q9BTZ2|DHRS4_HUMAN	0.00865555	0
sp|P00390-2|GSHR_HUMAN	0.00939941	0
sp|P35237|SPB6_HUMAN	0.00954056	0
sp|P51572-2|BAP31_HUMAN	0.01036835	0
sp|Q96EP5-2|DAZP1_HUMAN	0.0105629	0
sp|Q16539-2|MK14_HUMAN	0.01104546	0
sp|P41091|IF2G_HUMAN	0.01114464	0.09339783
sp|Q9H8L6|MMRN2_HUMAN	0.01131821	0.312067
sp|O94788-4|AL1A2_HUMAN	0.01198196	0
sp|Q9NUJ1|ABHDA_HUMAN	0.01257324	0
sp|Q04206-3|TF65_HUMAN	0.01298714	0.19149946
sp|O75369-2|FLNB_HUMAN	0.0134182	0.0538261
sp|O15144|ARPC2_HUMAN	0.01389313	0.05681695
sp|Q9BTW9-4|TBCD_HUMAN	0.01407242	0.2178309
sp|Q8NBX0|SCPDL_HUMAN	0.0144043	0
sp|P62318-2|SMD3_HUMAN	0.01449776	0
sp|Q86VP6|CAND1_HUMAN	0.01451111	0
sp|P50579-2|MAP2_HUMAN	0.01451874	0

sp|Q9ULC5-3|ACSL5_HUMAN	0.01470566	0
sp|P55735|SEC13_HUMAN	0.01507378	0
sp|Q6P2Q9|PRP8_HUMAN	0.01509094	0
sp|P19338|NUCL_HUMAN	0.01518059	0.00590555
sp|P62316|SMD2_HUMAN	0.01535797	0
sp|P07737|PROF1_HUMAN	0.01581955	0
sp|P46926|GNPI1_HUMAN	0.01594925	0
sp|O60547-2|GMDS_HUMAN	0.01601028	0
sp|Q8WUY1|THEM6_HUMAN	0.01642609	0
sp|Q15637-3|SF01_HUMAN	0.01646805	0
sp|P24539|AT5F1_HUMAN	0.01701355	0.03367973
sp|Q13185|CBX3_HUMAN	0.01710701	0
sp|Q96RQ3|MCCA_HUMAN	0.01753998	0
sp|P60174|TPIS_HUMAN	0.01762962	0.01290371
sp|P10155-3|RO60_HUMAN	0.01767159	0.02662771
sp|P21399|ACOC_HUMAN	0.01779175	0
sp|Q15393|SF3B3_HUMAN	0.01794434	0.01178462
sp|P50990|TCPQ_HUMAN	0.01804733	0.02720552
sp|O95352|ATG7_HUMAN	0.01822853	0
sp|Q06787-10|FMR1_HUMAN	0.01872444	0
sp|Q9NY33|DPP3_HUMAN	0.01911354	0.08684197
sp|O15382|BCAT2_HUMAN	0.01915932	0
sp|P20339-2|RAB5A_HUMAN	0.01981735	0
sp|O75155|CAND2_HUMAN	0.0198288	0
sp|Q9H299|SH3L3_HUMAN	0.02031708	0.09894868
sp|P51452-2|DUS3_HUMAN	0.02035904	0
sp|P23528|COF1_HUMAN	0.02077866	0
sp|Q92696|PGTA_HUMAN	0.0208931	0
sp|P47756-2|CAPZB_HUMAN	0.02152634	0.02388411
sp|P09497-2|CLCB_HUMAN	0.02172089	0
sp|P04899-3|GNAI2_HUMAN	0.0218277	0
sp|O75306-2|NDUS2_HUMAN	0.02214241	0.2178309
sp|P55786|PSA_HUMAN	0.02218628	0.19276452
sp|P62304|RUXE_HUMAN	0.02231026	0
sp|Q9BSJ8-2|ESYT1_HUMAN	0.02278519	0.01044662
sp|P46939-2|UTRO_HUMAN	0.02289581	0.04973141
sp|P61923-5|COPZ1_HUMAN	0.0229702	0
sp|Q96HY6|DDRGK_HUMAN	0.02315521	0.06291623
sp|P17612|KAPCA_HUMAN	0.02331734	0
sp|Q13404|UB2V1_HUMAN	0.02334976	0
sp|O94905|ERLN2_HUMAN	0.02359963	0
sp|Q16775-2|GLO2_HUMAN	0.0236845	0
sp|Q9UBV8|PEF1_HUMAN	0.02434921	0
sp|Q96QK1|VPS35_HUMAN	0.02451897	0.19149946
sp|P15090|FABP4_HUMAN	0.02486992	0

sp|Q16543|CDC37_HUMAN	0.02512932	0
sp|Q13188|STK3_HUMAN	0.0255909	0
sp|O00499-10|BIN1_HUMAN	0.02565002	0
sp|Q9Y224|RTRAF_HUMAN	0.02578735	0.20467198
sp|E9PAV3|NACAM_HUMAN	0.02585602	0
sp|Q16787-3|LAMA3_HUMAN	0.02597809	0.19149946
sp|Q3LXA3|TKFC_HUMAN	0.0263195	0.10280301
sp|P49593|PPM1F_HUMAN	0.02634144	0
sp|Q6DKJ4|NXN_HUMAN	0.02635002	0
sp|Q9H845|ACAD9_HUMAN	0.02658081	0
sp|P24821-4|TENA_HUMAN	0.02688789	0.071984
sp|P22695|QCR2_HUMAN	0.02749443	0.02174174
sp|Q13409-2|DC1I2_HUMAN	0.0281868	0
sp|Q9NY15|STAB1_HUMAN	0.02863693	0
sp|P06753-2|TPM3_HUMAN	0.02917862	0
sp|P15311|EZRI_HUMAN	0.02918053	0
sp|O00410|IPO5_HUMAN	0.02920914	0.06986027
sp|Q6XQN6|PNCB_HUMAN	0.02937698	0
sp|P09525|ANXA4_HUMAN	0.0294075	0.01406953
sp|Q9NSD9|SYFB_HUMAN	0.02985764	0
sp|Q9NPJ3-2|ACO13_HUMAN	0.03058052	0
sp|Q9NQC3|RTN4_HUMAN	0.03091812	0.26737198
sp|P14678|RSMB_HUMAN	0.03134155	0.19149946
sp|Q9H0D6|XRN2_HUMAN	0.03320885	0.25789237
sp|P40939|ECHA_HUMAN	0.03330231	0.07369066
sp|Q9P2T1-2|GMPR2_HUMAN	0.03363609	0
sp|P07384|CAN1_HUMAN	0.03373718	0
sp|P13797|PLST_HUMAN	0.03376389	0.00697919
sp|Q16647|PTGIS_HUMAN	0.03455544	0
sp|Q06136|KDSR_HUMAN	0.03455734	0
sp|P04632|CPNS1_HUMAN	0.03457832	0.02174174
sp|P18669|PGAM1_HUMAN	0.03470802	0
sp|P28072|PSB6_HUMAN	0.03494453	0
sp|O95865|DDAH2_HUMAN	0.03538132	0.01819076
sp|P38919|IF4A3_HUMAN	0.03542709	0.02174174
sp|P50454|SERPH_HUMAN	0.03556442	0.02662771
sp|P22392-2|NDKB_HUMAN	0.03581619	0
sp|P35268|RL22_HUMAN	0.03596306	0
sp|P25705|ATPA_HUMAN	0.03596878	0.07107546
sp|P46940|IQGA1_HUMAN	0.03609085	0.05398579
sp|Q09161|NCBP1_HUMAN	0.03625107	0
sp|P84095|RHOG_HUMAN	0.03674126	0.5204253
sp|P48147|PPCE_HUMAN	0.03683853	0
sp|P12081-4|SYHC_HUMAN	0.03694916	0
sp|O75083|WDR1_HUMAN	0.03722572	0

sp|P0DJI8|SAA1_HUMAN	0.03725815	0
sp|P50552|VASP_HUMAN	0.03735542	0
sp|Q08379|GOGA2_HUMAN	0.03795815	0
sp|O75915|PRAF3_HUMAN	0.03881264	0
sp|Q9H0E2|TOLIP_HUMAN	0.03893471	0
sp|P49902-2|5NTC_HUMAN	0.03933525	0
sp|Q13596-2|SNX1_HUMAN	0.03938294	0
sp|O00764-2|PDXK_HUMAN	0.03957844	0.2207585
sp|O43865|SAHH2_HUMAN	0.0397377	0
sp|Q13884|SNTB1_HUMAN	0.03975296	0
sp|P49903-2|SPS1_HUMAN	0.03979397	0
sp|Q96BW5-2|PTER_HUMAN	0.04001617	0
sp|Q04917|1433F_HUMAN	0.04035568	0.09215608
sp|P08758|ANXA5_HUMAN	0.04094124	0
sp|P48059-3|LIMS1_HUMAN	0.04157066	0
sp|P30566|PUR8_HUMAN	0.04160309	0
sp|O60832-2|DKC1_HUMAN	0.04233551	0
sp|P52597|HNRPF_HUMAN	0.04255867	0
sp|Q9Y6C2|EMIL1_HUMAN	0.04256821	0.00764478
sp|O43747-2|AP1G1_HUMAN	0.04354858	0
sp|O43707|ACTN4_HUMAN	0.04380989	0
sp|Q16822|PCKGM_HUMAN	0.04404068	0
sp|P54727|RD23B_HUMAN	0.04471493	0
sp|P14868|SYDC_HUMAN	0.04482079	0
sp|P40925-2|MDHC_HUMAN	0.0454731	0
sp|Q9P2M7|CING_HUMAN	0.04577732	0
sp|Q92688-2|AN32B_HUMAN	0.04577828	0
sp|Q9UMS4|PRP19_HUMAN	0.04583931	0
sp|Q13505-3|MTX1_HUMAN	0.04594231	0
sp|P27348|1433T_HUMAN	0.04659462	0
sp|Q14103-3|HNRPD_HUMAN	0.04702568	0.20791115
sp|P61970|NTF2_HUMAN	0.04706383	0
sp|O94979-10|SC31A_HUMAN	0.04795647	0
sp|P63010-2|AP2B1_HUMAN	0.04810715	0.04053069
sp|Q68EM7-2|RHG17_HUMAN	0.04829216	0
sp|O95425-4|SVIL_HUMAN	0.04852676	0
sp|Q9Y2S2|CRYL1_HUMAN	0.04853058	0.1342476
sp|Q13724-2|MOGS_HUMAN	0.04875183	0
sp|P51884|LUM_HUMAN	0.049263	0.02127174
sp|P13667|PDIA4_HUMAN	0.0494175	0.01189081
sp|O15173-2|PGRC2_HUMAN	0.04988289	0.17319627
sp|Q99497|PARK7_HUMAN	0.049963	0.44572112
sp|P46736-3|BRCC3_HUMAN	0.0501976	0
sp|Q5SSJ5-2|HP1B3_HUMAN	0.05023575	0.02662771
sp|Q9H4A4|AMPB_HUMAN	0.05032539	0.00464747

sp|Q15366-2|PCBP2_HUMAN	0.05088806	0
sp|P28482|MK01_HUMAN	0.05089378	0
sp|O75367-2|H2AY_HUMAN	0.05127525	0.06953826
sp|O43795-2|MYO1B_HUMAN	0.05137539	0.09339783
sp|P42025|ACTY_HUMAN	0.05206299	0
sp|Q13162|PRDX4_HUMAN	0.05254173	0
sp|Q9P0K7-2|RAI14_HUMAN	0.05269241	0
sp|Q9Y678|COPG1_HUMAN	0.05288696	0.01406953
sp|P08238|HS90B_HUMAN	0.05313873	0.05299055
sp|Q00765|REEP5_HUMAN	0.05316162	0.0846519
sp|Q15181|IPYR_HUMAN	0.05328369	0
sp|P30419-2|NMT1_HUMAN	0.05348396	0
sp|O00231-2|PSD11_HUMAN	0.05355454	0.1500082
sp|P55795|HNRH2_HUMAN	0.05355644	0
sp|Q8TBC4|UBA3_HUMAN	0.05377197	0
sp|Q86VS8|HOOK3_HUMAN	0.05378723	0
sp|Q9Y6B6|SAR1B_HUMAN	0.0538826	0
sp|Q9UH99-2|SUN2_HUMAN	0.0540762	0
sp|P52907|CAZA1_HUMAN	0.05429459	0.18414244
sp|Q969X5|ERGI1_HUMAN	0.05429459	0
sp|Q8IWL2-2|SFTA1_HUMAN	0.05452347	0.32654193
sp|Q7Z7G0|TARSH_HUMAN	0.0547905	0
sp|Q96I15|SCLY_HUMAN	0.05496407	0
sp|P25789|PSA4_HUMAN	0.05509567	0
sp|Q9NX63|MIC19_HUMAN	0.05510521	0
sp|P61006|RAB8A_HUMAN	0.05515099	0
sp|Q5T013-4|HYI_HUMAN	0.05515862	0
sp|P09496-2|CLCA_HUMAN	0.05535507	0
sp|Q1KMD3|HNRL2_HUMAN	0.05540276	0
sp|P50453|SPB9_HUMAN	0.0554142	0.01343579
sp|P30050|RL12_HUMAN	0.05583	0.20467198
sp|P60900|PSA6_HUMAN	0.05598068	0
sp|P31930|QCR1_HUMAN	0.05611801	0
sp|O95050-2|INMT_HUMAN	0.05612946	0
sp|Q12907|LMAN2_HUMAN	0.05643082	0
sp|Q92882|OSTF1_HUMAN	0.05709744	0.06291623
sp|P15121|ALDR_HUMAN	0.05713272	0
sp|P36776-2|LONM_HUMAN	0.05719757	0
sp|P01911|2B1F_HUMAN	0.05741501	0
sp|Q86TX2|ACOT1_HUMAN	0.05788803	0
sp|Q99729-2|ROAA_HUMAN	0.05807686	0.5204253
sp|P07195|LDHB_HUMAN	0.05825996	0
sp|Q15417|CNN3_HUMAN	0.05882645	0
sp|P36871|PGM1_HUMAN	0.05898285	0
sp|O95782-2|AP2A1_HUMAN	0.05920029	0

sp|Q9P258|RCC2_HUMAN	0.05974388	0
sp|Q92947|GCDH_HUMAN	0.0597477	0
sp|Q7L2H7|EIF3M_HUMAN	0.05982018	0.09894868
sp|Q8WZA0-2|LZIC_HUMAN	0.05993748	0
sp|P10301|RRAS_HUMAN	0.06015015	0
sp|O14980|XPO1_HUMAN	0.06015587	0
sp|P05165-2|PCCA_HUMAN	0.06088638	0
sp|O60506-3|HNRPQ_HUMAN	0.06090927	0
sp|P09972|ALDOC_HUMAN	0.06105614	0
sp|P63279|UBC9_HUMAN	0.062603	0
sp|Q8N1G4|LRC47_HUMAN	0.06296921	0.06986027
sp|P48444|COPD_HUMAN	0.06298637	0
sp|P22102|PUR2_HUMAN	0.06307697	0
sp|Q9Y696|CLIC4_HUMAN	0.06321716	0
sp|P28066|PSA5_HUMAN	0.06330681	0
sp|Q9P0L0-2|VAPA_HUMAN	0.06342125	0.02174174
sp|Q9Y5P6-2|GMPPB_HUMAN	0.06374359	0
sp|Q13247-3|SRSF6_HUMAN	0.06394959	0.19149946
sp|Q9NP72-2|RAB18_HUMAN	0.06402969	0
sp|O00483|NDUA4_HUMAN	0.06420326	0
sp|Q15691|MARE1_HUMAN	0.06432343	0
sp|P17987|TCPA_HUMAN	0.06433678	0.12893641
sp|P19474|RO52_HUMAN	0.06467629	0
sp|Q96C23|GALM_HUMAN	0.06474018	0
sp|O75436|VP26A_HUMAN	0.06506348	0
sp|Q9NZL9-2|MAT2B_HUMAN	0.06511307	0
sp|P02768|ALBU_HUMAN	0.06556702	0.74178255
sp|P26639-2|SYTC_HUMAN	0.06572151	0
sp|Q9NR45|SIAS_HUMAN	0.06584168	0
sp|Q15185-3|TEBP_HUMAN	0.06595802	0
sp|P78527|PRKDC_HUMAN	0.06599045	0
sp|Q9Y281|COF2_HUMAN	0.06718254	0
sp|P53621-2|COPA_HUMAN	0.06768417	0
sp|P12111|CO6A3_HUMAN	0.06774712	0.35520625
sp|Q99460-2|PSMD1_HUMAN	0.06791687	0
sp|P14174|MIF_HUMAN	0.06825447	0
sp|P58546|MTPN_HUMAN	0.06827927	0
sp|P23526|SAHH_HUMAN	0.06846046	0.08041298
sp|P26599-2|PTBP1_HUMAN	0.06925964	0
sp|O14617-4|AP3D1_HUMAN	0.06937695	0
sp|P04275|VWF_HUMAN	0.06959152	0
sp|Q14204|DYHC1_HUMAN	0.06960869	0.12838145
sp|Q9P2R7-2|SUCB1_HUMAN	0.06964302	0
sp|P48047|ATPO_HUMAN	0.06988144	0.1329664
sp|O76011|KRT34_HUMAN	0.07020569	0

sp|Q6KB66-2|K2C80_HUMAN	0.07066727	0
sp|P06756-3|ITAV_HUMAN	0.07076836	0
sp|Q16531|DDB1_HUMAN	0.0709877	0.4210128
sp|Q9H9G7-2|AGO3_HUMAN	0.07123566	0
sp|P00746|CFAD_HUMAN	0.07250214	0
sp|P49257|LMAN1_HUMAN	0.07339287	0
sp|P12956|XRCC6_HUMAN	0.07395554	0
sp|Q6YHK3-4|CD109_HUMAN	0.07396698	0.2178309
sp|P27695|APEX1_HUMAN	0.0740242	0
sp|Q14139-2|UBE4A_HUMAN	0.07470703	0
sp|P19525-2|E2AK2_HUMAN	0.07481289	0
sp|Q15075|EEA1_HUMAN	0.07523727	0.13150784
sp|P11413-3|G6PD_HUMAN	0.0753479	0
sp|Q14764|MVP_HUMAN	0.07541084	0.15575379
sp|P01116-2|RASK_HUMAN	0.07650566	0
sp|P36542|ATPG_HUMAN	0.07652855	0
sp|Q14203-4|DCTN1_HUMAN	0.07679939	0.03712561
sp|P33151|CADH5_HUMAN	0.07760811	0
sp|Q15172-2|2A5A_HUMAN	0.07770157	0
sp|Q02978|M2OM_HUMAN	0.07783508	0.24097534
sp|P49368|TCPG_HUMAN	0.07785797	0.3915664
sp|P02649|APOE_HUMAN	0.07857513	0.02863072
sp|P39656-3|OST48_HUMAN	0.0789032	0.2591514
sp|P35232|PHB_HUMAN	0.0792923	0.07131392
sp|Q9Y5Z4-2|HEBP2_HUMAN	0.07992554	0
sp|Q5JPE7-2|NOMO2_HUMAN	0.08025742	0
sp|Q96A33-2|CCD47_HUMAN	0.08034515	0
sp|P11233|RALA_HUMAN	0.0803566	0.5204253
sp|Q99798|ACON_HUMAN	0.08040047	0
sp|P10606|COX5B_HUMAN	0.08049774	0
sp|Q99459|CDC5L_HUMAN	0.0809021	0
sp|O75396|SC22B_HUMAN	0.08211136	0.03367973
sp|O95336|6PGL_HUMAN	0.0821743	0.11612091
sp|P49720|PSB3_HUMAN	0.08240891	0.04454162
sp|P09104-2|ENOG_HUMAN	0.08323479	0
sp|P07900-2|HS90A_HUMAN	0.08348656	0
sp|Q9Y3F4-2|STRAP_HUMAN	0.08371925	0
sp|P00491|PNPH_HUMAN	0.08419228	0.14672586
sp|O75368|SH3L1_HUMAN	0.0845089	0
sp|Q15005|SPCS2_HUMAN	0.08472443	0
sp|Q15365|PCBP1_HUMAN	0.08480454	0.0846519
sp|Q9BR76|COR1B_HUMAN	0.08488464	0
sp|P49961|ENTP1_HUMAN	0.08503914	0.04454162
sp|P13798|ACPH_HUMAN	0.08532333	0
sp|P61160|ARP2_HUMAN	0.08600044	0

sp|P23083|HV102_HUMAN	0.08623886	0
sp|P63096|GNAI1_HUMAN	0.08634186	0
sp|Q08257|QOR_HUMAN	0.08642006	0.15510327
sp|A0MZ66-5|SHOT1_HUMAN	0.08650017	0
sp|P46821|MAP1B_HUMAN	0.08656311	0
sp|O75937|DNJC8_HUMAN	0.08678627	0
sp|O00299|CLIC1_HUMAN	0.08690643	0.00842351
sp|P53597|SUCA_HUMAN	0.08693314	0
sp|P35998|PRS7_HUMAN	0.08713913	0.13973783
sp|Q9HC35-2|EMAL4_HUMAN	0.08736992	0.55270666
sp|P13693|TCTP_HUMAN	0.08814812	0
sp|Q969G5|CAVN3_HUMAN	0.08820152	0.06291623
sp|P62495-2|ERF1_HUMAN	0.08820725	0
sp|P48163|MAOX_HUMAN	0.08895302	0.06986027
sp|P49821-2|NDUV1_HUMAN	0.08932877	0
sp|Q16851-2|UGPA_HUMAN	0.08937264	0
sp|P13073|COX41_HUMAN	0.08974075	0
sp|P11766|ADHX_HUMAN	0.09071922	0.33432218
sp|P56199|ITA1_HUMAN	0.09140968	0.4488957
sp|O60256-3|KPRB_HUMAN	0.09157944	0.21439649
sp|P05198|IF2A_HUMAN	0.09230423	0
sp|Q15046-2|SYK_HUMAN	0.09237862	0
sp|P59998|ARPC4_HUMAN	0.09270477	0
sp|P48637|GSHB_HUMAN	0.09293175	0.09215608
sp|Q96M27-3|PRRC1_HUMAN	0.09315682	0
sp|P55809|SCOT1_HUMAN	0.09344673	0
sp|Q9BPW8|NIPS1_HUMAN	0.09347916	0
sp|P30520|PURA2_HUMAN	0.0937748	0
sp|Q9Y262|EIF3L_HUMAN	0.09386253	0
sp|Q13423|NNTM_HUMAN	0.0939045	0.07727676
sp|Q92506|DHB8_HUMAN	0.09469223	0
sp|P47755|CAZA2_HUMAN	0.09495926	0
sp|P07988|PSPB_HUMAN	0.09502029	0.7980934
sp|Q96AC1|FERM2_HUMAN	0.09533405	0.14237618
sp|P55036-2|PSMD4_HUMAN	0.09540558	0
sp|Q96CN7|ISOC1_HUMAN	0.09569168	0
sp|Q08945|SSRP1_HUMAN	0.09576607	0
sp|P49354|FNTA_HUMAN	0.09583283	0.35795313
sp|Q86WV6|STING_HUMAN	0.09630394	0
sp|Q9BU23-3|LMF2_HUMAN	0.0968647	0
sp|Q8NBF2-2|NHLC2_HUMAN	0.09689331	0
sp|Q92499|DDX1_HUMAN	0.09703827	0
sp|Q14980-2|NUMA1_HUMAN	0.09794426	0.6092752
sp|Q86UX7-2|URP2_HUMAN	0.09838867	0
sp|Q9UQ80|PA2G4_HUMAN	0.09879875	0

sp|P21283|VATC1_HUMAN	0.09938431	0
sp|Q9NTJ5|SAC1_HUMAN	0.09958267	0.03043297
sp|P08865|RSSA_HUMAN	0.09967423	0
sp|P51153|RAB13_HUMAN	0.1000042	0
sp|P04424-2|ARLY_HUMAN	0.10026169	0
sp|Q00577|PURA_HUMAN	0.10042191	0.06291623
sp|Q9P2E9|RRBP1_HUMAN	0.10050774	0.19478811
sp|P30153|2AAA_HUMAN	0.10056305	0
sp|P19404|NDUV2_HUMAN	0.10072327	0
sp|P49419-2|AL7A1_HUMAN	0.10089684	0
sp|Q9BUQ8|DDX23_HUMAN	0.10125732	0
sp|Q9Y3I0|RTCB_HUMAN	0.10133743	0.21989624
sp|O95861-4|BPNT1_HUMAN	0.10147476	0
sp|O75881|CP7B1_HUMAN	0.10165024	0
sp|O75874|IDHC_HUMAN	0.10169792	0
sp|Q13045-3|FLII_HUMAN	0.10178566	0
sp|P53602|MVD1_HUMAN	0.10233116	0
sp|Q96FW1|OTUB1_HUMAN	0.10240173	0
sp|Q9Y490|TLN1_HUMAN	0.10301209	0.09570572
sp|P42330|AK1C3_HUMAN	0.10333633	0
sp|P0DMV9|HS71B_HUMAN	0.10349274	0
sp|O14818|PSA7_HUMAN	0.10361099	0.04454162
sp|P24534|EF1B_HUMAN	0.10389519	0
sp|Q8WUM4|PDC6I_HUMAN	0.10393524	0.16644415
sp|O15498-2|YKT6_HUMAN	0.10422516	0
sp|O43143|DHX15_HUMAN	0.10428619	0.3630891
sp|P61769|B2MG_HUMAN	0.10549927	0
sp|P54886-2|P5CS_HUMAN	0.10551071	0
sp|Q8N5K1|CISD2_HUMAN	0.10592842	0.45033538
sp|Q99439|CNN2_HUMAN	0.10597801	0
sp|Q02543|RL18A_HUMAN	0.1060524	0.04454162
sp|P53618|COPB_HUMAN	0.10629845	0.04904167
sp|P08571|CD14_HUMAN	0.10648918	0.37060758
sp|O94911-3|ABCA8_HUMAN	0.1065197	0.45033538
sp|Q15126|PMVK_HUMAN	0.10664749	0.19149946
sp|Q09028-3|RBBP4_HUMAN	0.10680389	0
sp|O15511|ARPC5_HUMAN	0.10683441	0
sp|O15372|EIF3H_HUMAN	0.10684776	0
sp|Q92930|RAB8B_HUMAN	0.10691643	0
sp|Q9UHX1-2|PUF60_HUMAN	0.10700989	0
sp|O14828-2|SCAM3_HUMAN	0.1070652	0
sp|Q9UBF2|COPG2_HUMAN	0.10707855	0
sp|Q9UPQ0-3|LIMC1_HUMAN	0.10735893	0.24097534
sp|Q29865|1C18_HUMAN	0.10759926	0.2178309
sp|P51553-2|IDH3G_HUMAN	0.10764694	0

sp|Q71U36-2|TBA1A_HUMAN	0.10790062	0
sp|P12955|PEPD_HUMAN	0.10832596	0
sp|P62330|ARF6_HUMAN	0.10846138	0
sp|P30086|PEBP1_HUMAN	0.10893631	0
sp|P08729|K2C7_HUMAN	0.10906792	0.19248605
sp|P61457|PHS_HUMAN	0.10921288	0
sp|O95834|EMAL2_HUMAN	0.10939407	0.02174174
sp|P08559-2|ODPA_HUMAN	0.10948753	0
sp|P07741|APT_HUMAN	0.10956955	0
sp|P52566|GDIR2_HUMAN	0.11003113	0
sp|Q92817|EVPL_HUMAN	0.1101799	0
sp|Q30134|2B18_HUMAN	0.11049652	0
sp|P31943|HNRH1_HUMAN	0.11090279	0.03367973
sp|O75340-2|PDCD6_HUMAN	0.11099815	0
sp|P30041|PRDX6_HUMAN	0.11139488	0.01576272
sp|P31942-2|HNRH3_HUMAN	0.11150932	0.48520416
sp|Q12906-2|ILF3_HUMAN	0.1115303	0.01700861
sp|Q13148|TADBP_HUMAN	0.11176109	0.35795313
sp|P45974-2|UBP5_HUMAN	0.11190796	0.04872547
sp|Q9UKG1|DP13A_HUMAN	0.11194992	0
sp|Q93009-3|UBP7_HUMAN	0.11199951	0
sp|Q8IY17-3|PLPL6_HUMAN	0.11215591	0
sp|P68104|EF1A1_HUMAN	0.11245155	0.14871502
sp|Q5JTV8-3|TOIP1_HUMAN	0.11271858	0
sp|P08754|GNAI3_HUMAN	0.11314201	0
sp|Q2TAY7|SMU1_HUMAN	0.11333466	0
sp|Q9UBS4|DJB11_HUMAN	0.1135006	0
sp|P40306|PSB10_HUMAN	0.11367226	0.61034113
sp|P23284|PPIB_HUMAN	0.11381912	0.21680334
sp|P52306|GDS1_HUMAN	0.11383057	0
sp|Q16401-2|PSMD5_HUMAN	0.11392784	0.05621411
sp|Q9P0V9-2|SEP10_HUMAN	0.11458778	0
sp|P60981-2|DEST_HUMAN	0.1146183	0
sp|Q14558-2|KPRA_HUMAN	0.11473179	0.656254
sp|P55884-2|EIF3B_HUMAN	0.11509514	0.23541966
sp|P04040|CATA_HUMAN	0.11517143	0.6542169
sp|P46063|RECQ1_HUMAN	0.11519241	0.05483269
sp|P56134-3|ATPK_HUMAN	0.11530304	0
sp|P40227|TCPZ_HUMAN	0.11621094	0.10328458
sp|P04844|RPN2_HUMAN	0.11645699	0.33495146
sp|Q96C19|EFHD2_HUMAN	0.1169014	0
sp|Q8N1B4-2|VPS52_HUMAN	0.11702728	0
sp|Q5TFE4|NT5D1_HUMAN	0.11785317	0
sp|P29992|GNA11_HUMAN	0.11810684	0
sp|P63241|IF5A1_HUMAN	0.11818314	0.7588735

sp|Q6UVK1|CSPG4_HUMAN	0.11835861	0
sp|P53396-2|ACLY_HUMAN	0.11844635	0.11475069
sp|P08294|SODE_HUMAN	0.11888886	0
sp|Q9Y3Z3|SAMH1_HUMAN	0.11917496	0
sp|P01859|IGHG2_HUMAN	0.11936569	0.84879977
sp|O00116|ADAS_HUMAN	0.12017632	0
sp|O60814|H2B1K_HUMAN	0.12043381	1.1932944
sp|Q9UBQ7|GRHPR_HUMAN	0.1204586	0
sp|Q9NQR4|NIT2_HUMAN	0.12055588	0.06291623
sp|P46976|GLYG_HUMAN	0.12067604	0.30372584
sp|Q96AE4-2|FUBP1_HUMAN	0.12072372	0
sp|P51888|PRELP_HUMAN	0.12074661	0
sp|Q14498-2|RBM39_HUMAN	0.12094116	0.40256184
sp|O60271-5|JIP4_HUMAN	0.12113667	0.19149946
sp|Q9Y3A3-3|PHOCN_HUMAN	0.12120438	0
sp|P00441|SODC_HUMAN	0.12145996	0
sp|O00291|HIP1_HUMAN	0.12179184	0
sp|P27338|AOFB_HUMAN	0.12202454	0
sp|P00558|PGK1_HUMAN	0.12207413	0
sp|P09917-3|LOX5_HUMAN	0.12222481	0.09894868
sp|O43390-2|HNRPR_HUMAN	0.12270927	0.06210651
sp|P14314-2|GLU2B_HUMAN	0.12322998	0.0846519
sp|Q9BY32|ITPA_HUMAN	0.1232605	0
sp|Q08209-2|PP2BA_HUMAN	0.12385178	0
sp|Q15404|RSU1_HUMAN	0.12454605	0.14969608
sp|Q9P289|STK26_HUMAN	0.12611198	0.656254
sp|P31937|3HIDH_HUMAN	0.12624931	0
sp|Q9Y376|CAB39_HUMAN	0.12631989	0
sp|Q92841-1|DDX17_HUMAN	0.12633896	0
sp|P35580|MYH10_HUMAN	0.1263504	0.42482498
sp|P13861|KAP2_HUMAN	0.12640381	0.290567
sp|P43307|SSRA_HUMAN	0.12641525	0.45033538
sp|O00567|NOP56_HUMAN	0.12711143	0
sp|Q9UJU6-2|DBNL_HUMAN	0.12804985	0
sp|P05455|LA_HUMAN	0.128191	0.07727676
sp|Q96G03|PGM2_HUMAN	0.12831879	0.03367973
sp|O00170|AIP_HUMAN	0.128479	0
sp|O15260-2|SURF4_HUMAN	0.12930107	0
sp|P50395|GDIB_HUMAN	0.1296196	0.01290371
sp|O00534|VMA5A_HUMAN	0.12993813	0
sp|O60504-2|VINEX_HUMAN	0.12998962	0.09894868
sp|P11217-2|PYGM_HUMAN	0.13007355	0
sp|P55060-3|XPO2_HUMAN	0.13021469	0.05634482
sp|Q9Y5X3|SNX5_HUMAN	0.13076401	0.37061578
sp|P23368|MAOM_HUMAN	0.13085556	0.04454162

sp|P07585|PGS2_HUMAN	0.13103485	0.2718748
sp|Q92896-2|GSLG1_HUMAN	0.1310463	0.09339783
sp|Q6UW02|CP20A_HUMAN	0.13110352	0.19149946
sp|O60763-2|USO1_HUMAN	0.13110542	0
sp|P22314-2|UBA1_HUMAN	0.13131523	0
sp|P08133|ANXA6_HUMAN	0.13162613	0.01620588
sp|Q14974|IMB1_HUMAN	0.13197899	0.13745423
sp|Q15642-3|CIP4_HUMAN	0.13368988	0
sp|O95833|CLIC3_HUMAN	0.1339035	0
sp|P20591|MX1_HUMAN	0.13434029	0.7061832
sp|P54819-2|KAD2_HUMAN	0.13444519	0
sp|Q53EL6-2|PDCD4_HUMAN	0.13461113	0
sp|Q8N8S7-2|ENAH_HUMAN	0.13465881	0
sp|P02751-15|FINC_HUMAN	0.13515854	0
sp|Q9UBB4-2|ATX10_HUMAN	0.1356163	0
sp|Q9Y4L1|HYOU1_HUMAN	0.13562012	0.14750385
sp|P62136|PP1A_HUMAN	0.13591385	0
sp|Q13098-5|CSN1_HUMAN	0.13596725	0.04454162
sp|P11940-2|PABP1_HUMAN	0.13610458	0
sp|P62942|FKB1A_HUMAN	0.13714981	0
sp|Q8TE77-3|SSH3_HUMAN	0.13715744	0
sp|P20073-2|ANXA7_HUMAN	0.13724709	0.34244674
sp|P17980|PRS6A_HUMAN	0.13738823	0
sp|P14618-2|KPYM_HUMAN	0.13747025	0
sp|Q13310-2|PABP4_HUMAN	0.13748932	0
sp|Q8NF37|PCAT1_HUMAN	0.13759327	0.1500082
sp|O43491|E41L2_HUMAN	0.1376667	0.02174174
sp|Q9P0J0-2|NDUAD_HUMAN	0.1383133	0
sp|Q9BT78|CSN4_HUMAN	0.13832855	0.03367973
sp|P40261|NNMT_HUMAN	0.13840675	0.19149946
sp|Q6WCQ1-2|MPRIP_HUMAN	0.13840675	0.26295313
sp|P60842|IF4A1_HUMAN	0.13853073	0.14750385
sp|Q07065|CKAP4_HUMAN	0.13858414	0.33825856
sp|Q15435|PP1R7_HUMAN	0.13877201	0
sp|Q7L1Q6-2|BZW1_HUMAN	0.13878632	0
sp|P15153|RAC2_HUMAN	0.13899231	0.656254
sp|Q9NZT2-2|OGFR_HUMAN	0.13899708	0.35795313
sp|Q12797-10|ASPH_HUMAN	0.13910484	0.44572112
sp|P18206-2|VINC_HUMAN	0.13926697	0.11062484
sp|Q9Y4D7-2|PLXD1_HUMAN	0.13948345	0
sp|P05388-2|RLA0_HUMAN	0.1397438	0
sp|P61163|ACTZ_HUMAN	0.13982391	0
sp|P01111|RASN_HUMAN	0.14011002	0
sp|O60313-10|OPA1_HUMAN	0.14019775	0
sp|P47897|SYQ_HUMAN	0.14038086	0

sp|Q6PCB0|VWA1_HUMAN	0.1413536	0
sp|O14950|ML12B_HUMAN	0.1415062	0
sp|Q9BTV4|TMM43_HUMAN	0.14159775	0.33495146
sp|P46777|RL5_HUMAN	0.14180946	0.13604218
sp|B5ME19|EIFCL_HUMAN	0.14181137	0.05483269
sp|Q92900-2|RENT1_HUMAN	0.14185143	0.3412366
sp|Q96IU4|ABHEB_HUMAN	0.14194107	0
sp|O60716-14|CTND1_HUMAN	0.14229202	0
sp|Q99623|PHB2_HUMAN	0.14240456	0.39446053
sp|Q07960|RHG01_HUMAN	0.14263916	0.23973103
sp|P35270|SPRE_HUMAN	0.14266396	0
sp|Q16204|CCDC6_HUMAN	0.14352226	0
sp|P26038|MOES_HUMAN	0.14470482	0.10086016
sp|O60664-4|PLIN3_HUMAN	0.1452179	0
sp|P00325|ADH1B_HUMAN	0.14522362	0.03712561
sp|Q9UJW0-3|DCTN4_HUMAN	0.14565277	0
sp|Q02790|FKBP4_HUMAN	0.14577103	0.20467198
sp|Q15102|PA1B3_HUMAN	0.14586067	0.6298893
sp|Q9NP79|VTA1_HUMAN	0.14597893	0
sp|Q96CW1-2|AP2M1_HUMAN	0.14605904	0
sp|P61978-3|HNRPK_HUMAN	0.1467247	0.07488067
sp|Q92945|FUBP2_HUMAN	0.14679909	0.1897449
sp|P51571|SSRD_HUMAN	0.14687729	0
sp|Q7LG56-6|RIR2B_HUMAN	0.14735794	0
sp|P05387|RLA2_HUMAN	0.14764404	0
sp|P78371|TCPB_HUMAN	0.14805412	0.05908025
sp|Q9BVK6|TMED9_HUMAN	0.14848328	0
sp|P25786-2|PSA1_HUMAN	0.14895439	0.01178462
sp|Q96TA1-2|NIBL1_HUMAN	0.14910698	0
sp|P01860|IGHG3_HUMAN	0.14911842	0.56808305
sp|P20618|PSB1_HUMAN	0.14990997	0
sp|Q6IBS0|TWF2_HUMAN	0.15007591	0
sp|P02760|AMBP_HUMAN	0.15027809	0.1500082
sp|Q92890-1|UFD1_HUMAN	0.15028	0.19149946
sp|O75688|PPM1B_HUMAN	0.15064621	0
sp|Q9Y3A5|SBDS_HUMAN	0.15094566	0
sp|P30101|PDIA3_HUMAN	0.15097046	0.07107546
sp|P52565|GDIR1_HUMAN	0.1517353	0.02174174
sp|P04843|RPN1_HUMAN	0.1519413	0.13882811
sp|P39019|RS19_HUMAN	0.15207672	0.09894868
sp|P12277|KCRB_HUMAN	0.15208054	0.41804093
sp|P55084|ECHB_HUMAN	0.15303993	0.26665917
sp|Q8IZ83-3|A16A1_HUMAN	0.15306473	0.24295025
sp|P30533|AMRP_HUMAN	0.15317154	0
sp|Q7Z7H5-3|TMED4_HUMAN	0.1532917	0

sp|O00303|EIF3F_HUMAN	0.15349007	0
sp|Q05655-2|KPCD_HUMAN	0.1535492	0.28516325
sp|Q99714|HCD2_HUMAN	0.15355301	0.03712561
sp|P54578-2|UBP14_HUMAN	0.15397644	0
sp|P17301|ITA2_HUMAN	0.15424538	0
sp|Q9NUQ9|FA49B_HUMAN	0.15428162	0.30372584
sp|O00487|PSDE_HUMAN	0.15481186	0
sp|P13010|XRCC5_HUMAN	0.15483284	0
sp|P00736|C1R_HUMAN	0.15493774	0
sp|Q9BRA2|TXD17_HUMAN	0.15522003	0
sp|Q00610-2|CLH1_HUMAN	0.1552887	0.1483452
sp|P22061-2|PIMT_HUMAN	0.15533447	0.39873424
sp|Q9UEY8|ADDG_HUMAN	0.15555954	0
sp|Q00796|DHSO_HUMAN	0.15589714	0
sp|O15145|ARPC3_HUMAN	0.15629578	0
sp|Q9NR31|SAR1A_HUMAN	0.15648651	0.19149946
sp|Q9NQG5|RPR1B_HUMAN	0.15683937	0
sp|P08727|K1C19_HUMAN	0.15688515	0.45995986
sp|Q8WVM8|SCFD1_HUMAN	0.15707588	0
sp|P0DOX7|IGK_HUMAN	0.15735245	0.45033538
sp|Q99538-2|LGMN_HUMAN	0.1575203	0
sp|Q16527|CSRP2_HUMAN	0.15754128	0
sp|P31153|METK2_HUMAN	0.15759468	0
sp|Q8NHP8|PLBL2_HUMAN	0.15778732	0
sp|O14745|NHRF1_HUMAN	0.1581707	0.48757824
sp|Q13243-3|SRSF5_HUMAN	0.15847397	0.19149946
sp|Q13561-2|DCTN2_HUMAN	0.15870857	0.01178462
sp|P51858|HDGF_HUMAN	0.15906525	0
sp|P63244|RACK1_HUMAN	0.15925026	0
sp|O60784|TOM1_HUMAN	0.15960884	0
sp|Q13464|ROCK1_HUMAN	0.1603756	0
sp|Q9Y230|RUVB2_HUMAN	0.16064835	0
sp|Q9UHQ9|NB5R1_HUMAN	0.16070747	0
sp|Q92888-2|ARHG1_HUMAN	0.16075993	0
sp|Q6NVY1|HIBCH_HUMAN	0.161026	0
sp|Q14914-2|PTGR1_HUMAN	0.1614685	0
sp|Q9UBI6|GBG12_HUMAN	0.16147232	0
sp|Q05707-2|COEA1_HUMAN	0.16181564	0.9582053
sp|O15127|SCAM2_HUMAN	0.16199303	0.35795313
sp|O00232|PSD12_HUMAN	0.1629324	0.29066643
sp|P16298-2|PP2BB_HUMAN	0.16360855	0
sp|Q99873-2|ANM1_HUMAN	0.16369247	0
sp|Q9BXS5-2|AP1M1_HUMAN	0.16379738	0
sp|P12235|ADT1_HUMAN	0.16383171	0
sp|P49588-2|SYAC_HUMAN	0.16394043	0.3503162

sp|O43852-5|CALU_HUMAN	0.16402054	0
sp|P60709|ACTB_HUMAN	0.16453171	0
sp|P12814|ACTN1_HUMAN	0.16453934	0
sp|A0AVT1|UBA6_HUMAN	0.16469574	0
sp|Q15942|ZYX_HUMAN	0.16547203	0.6634523
sp|P40763-2|STAT3_HUMAN	0.1658516	0.10138063
sp|Q9UNH7|SNX6_HUMAN	0.16606522	0
sp|P31939|PUR9_HUMAN	0.16615486	0.14899042
sp|Q6DD88|ATLA3_HUMAN	0.16619682	0.1500082
sp|P06310|KV230_HUMAN	0.16648674	0
sp|Q9UNF0-2|PACN2_HUMAN	0.16651154	0
sp|Q15286-2|RAB35_HUMAN	0.16664696	0
sp|P55039|DRG2_HUMAN	0.16681862	0
sp|Q96HC4|PDLI5_HUMAN	0.1671009	0.74639726
sp|P07437|TBB5_HUMAN	0.16713333	0.06291623
sp|O00429-3|DNM1L_HUMAN	0.16753769	0.52598757
sp|Q15233|NONO_HUMAN	0.16804504	0.09215608
sp|P35222|CTNB1_HUMAN	0.1685543	0
sp|Q14697-2|GANAB_HUMAN	0.16865158	0
sp|P11177-3|ODPB_HUMAN	0.16942596	0
sp|O43681|ASNA_HUMAN	0.16965675	0
sp|P35611-2|ADDA_HUMAN	0.16983032	0
sp|Q02952-2|AKA12_HUMAN	0.1701355	0
sp|Q9HCB6|SPON1_HUMAN	0.17065239	0
sp|Q96MM6|HS12B_HUMAN	0.1707325	0.43407488
sp|Q8N9N7|LRC57_HUMAN	0.17090034	0
sp|Q14118|DAG1_HUMAN	0.17213821	0
sp|Q15370-2|ELOB_HUMAN	0.1722393	0
sp|P08621-2|RU17_HUMAN	0.1728611	0.04454162
sp|Q13232|NDK3_HUMAN	0.17298698	0.312067
sp|Q9UGI8-2|TES_HUMAN	0.17322063	0
sp|P61018|RAB4B_HUMAN	0.17375183	0.45033538
sp|P43121|MUC18_HUMAN	0.17392349	0
sp|P04083|ANXA1_HUMAN	0.17417145	0.07916547
sp|O76003|GLRX3_HUMAN	0.17424774	0
sp|P01782|HV309_HUMAN	0.17769241	0
sp|Q8WVV9-5|HNRLL_HUMAN	0.17773914	0
sp|P68371|TBB4B_HUMAN	0.17785072	0
sp|O60884|DNJA2_HUMAN	0.17886257	0
sp|Q8WUP2-3|FBLI1_HUMAN	0.17887306	0
sp|O60879-2|DIAP2_HUMAN	0.17888641	0
sp|Q9Y265|RUVB1_HUMAN	0.17892838	0
sp|Q9NR19-2|ACSA_HUMAN	0.17975235	0
sp|Q9Y6W5|WASF2_HUMAN	0.18025589	0.09339783
sp|P51178-2|PLCD1_HUMAN	0.18029404	0.09894868

sp|P02452|CO1A1_HUMAN	0.18079948	0.6509351
sp|Q9NT62-2|ATG3_HUMAN	0.18167305	0
sp|Q27J81-2|INF2_HUMAN	0.18243027	0
sp|P07360|CO8G_HUMAN	0.18350983	0.45033538
sp|Q93052|LPP_HUMAN	0.18353653	0.4490988
sp|Q9UBG0|MRC2_HUMAN	0.18474102	0.29934928
sp|P27361|MK03_HUMAN	0.18616486	0.04454162
sp|P61956-2|SUMO2_HUMAN	0.18621826	0
sp|P55854-2|SUMO3_HUMAN	0.18645859	0
sp|Q13492-2|PICAL_HUMAN	0.18687057	0
sp|Q9Y285|SYFA_HUMAN	0.1871357	0
sp|Q9UHL4|DPP2_HUMAN	0.1876564	0
sp|P58107|EPIPL_HUMAN	0.18779564	0.24295025
sp|P54725-2|RD23A_HUMAN	0.18782806	0.08779849
sp|P02765|FETUA_HUMAN	0.18794155	0.70235044
sp|Q9NRX4|PHP14_HUMAN	0.18808174	0.5204253
sp|P08842|STS_HUMAN	0.18817139	0
sp|O95340-2|PAPS2_HUMAN	0.18822479	0.2591514
sp|P28331-3|NDUS1_HUMAN	0.18825722	0.06986027
sp|Q07866-10|KLC1_HUMAN	0.1884346	0
sp|P63000-2|RAC1_HUMAN	0.18878555	0
sp|Q9BW30|TPPP3_HUMAN	0.1889	0
sp|Q16891-2|MIC60_HUMAN	0.18910408	0.2838047
sp|O43242|PSMD3_HUMAN	0.18929291	0
sp|P23142-4|FBLN1_HUMAN	0.18967819	0
sp|O14964-2|HGS_HUMAN	0.18971634	0.21439649
sp|P50991|TCPD_HUMAN	0.18983269	0
sp|Q14152-2|EIF3A_HUMAN	0.18986511	0
sp|P20674|COX5A_HUMAN	0.18996048	0.7827403
sp|P29144|TPP2_HUMAN	0.18999672	0.21719235
sp|Q9UIJ7|KAD3_HUMAN	0.19032097	0
sp|A1L0T0|ILVBL_HUMAN	0.19055176	0
sp|Q9Y3C8|UFC1_HUMAN	0.19062424	0
sp|Q9HBL0|TENS1_HUMAN	0.19073868	0.11977793
sp|P10643|CO7_HUMAN	0.19092941	0
sp|Q92747|ARC1A_HUMAN	0.1910286	0
sp|A0A0C4DH38|HV551_HUMAN	0.191185	0
sp|P61020|RAB5B_HUMAN	0.19133377	0.35795313
sp|Q5JRX3-2|PREP_HUMAN	0.19191933	0
sp|P53004|BIEA_HUMAN	0.19201088	0
sp|P36873-2|PP1G_HUMAN	0.19207191	0
sp|Q53GG5-2|PDLI3_HUMAN	0.1920929	0
sp|P62191-2|PRS4_HUMAN	0.19264221	0.1342476
sp|P62701|RS4X_HUMAN	0.19292259	0.6298893
sp|P49915-2|GUAA_HUMAN	0.1931839	0

sp|Q9BVC6|TM109_HUMAN	0.19325256	0.5204253
sp|P34932|HSP74_HUMAN	0.19380188	0.13308895
sp|Q15124|PGM5_HUMAN	0.1939888	0.17253529
sp|Q9H4G4|GAPR1_HUMAN	0.19403839	0
sp|P08670|VIME_HUMAN	0.19415283	0.7731812
sp|P12268|IMDH2_HUMAN	0.19457817	0.48520416
sp|P00568|KAD1_HUMAN	0.1948967	0
sp|P52272-2|HNRPM_HUMAN	0.1955719	0.7947966
sp|O43837|IDH3B_HUMAN	0.19574165	0.2178309
sp|P62857|RS28_HUMAN	0.19582176	0.06291623
sp|Q06278|AOXA_HUMAN	0.1960659	0.45033538
sp|P62906|RL10A_HUMAN	0.1967907	0
sp|Q15019-2|SEPT2_HUMAN	0.19800949	0.46317288
sp|Q5TDH0-3|DDI2_HUMAN	0.19865036	0
sp|Q15717|ELAV1_HUMAN	0.1989727	0
sp|Q5TZA2|CROCC_HUMAN	0.19908714	0.65625405
sp|P07814|SYEP_HUMAN	0.19917488	0.13242109
sp|P12111-4|CO6A3_HUMAN	0.19932556	0
sp|P62888|RL30_HUMAN	0.19999886	0
sp|Q96DG6|CMBL_HUMAN	0.2000637	0
sp|Q15257-2|PTPA_HUMAN	0.20051384	0.21719235
sp|P02748|CO9_HUMAN	0.20060539	0.19484499
sp|Q99426|TBCB_HUMAN	0.20085716	0
sp|Q96RU3-3|FNBP1_HUMAN	0.20178127	0
sp|Q04828|AK1C1_HUMAN	0.20215988	0.2178309
sp|P27694|RFA1_HUMAN	0.20220566	0
sp|Q15274|NADC_HUMAN	0.20221329	0.1342476
sp|O75746-2|CMC1_HUMAN	0.20251846	0
sp|Q08431|MFGM_HUMAN	0.2026329	0.21439649
sp|Q9H9B4|SFXN1_HUMAN	0.20336533	0
sp|Q8WXF7-2|ATLA1_HUMAN	0.20343208	0
sp|P0CG39|POTEJ_HUMAN	0.20354462	0
sp|Q16555|DPYL2_HUMAN	0.2036171	0.10808486
sp|Q8NHV1|GIMA7_HUMAN	0.2038536	0.7061832
sp|P48506|GSH1_HUMAN	0.2039814	0.45033538
sp|O43252|PAPS1_HUMAN	0.20443916	0
sp|Q07812-2|BAX_HUMAN	0.20446587	0
sp|P50570-5|DYN2_HUMAN	0.20479584	0
sp|P08397-2|HEM3_HUMAN	0.20557785	0.35795313
sp|O95372|LYPA2_HUMAN	0.20656586	0
sp|P46108|CRK_HUMAN	0.20695686	0.2178309
sp|Q5VW32|BROX_HUMAN	0.20710373	0.19149946
sp|O75534-2|CSDE1_HUMAN	0.20792389	0.28516325
sp|Q9Y3B3|TMED7_HUMAN	0.20843124	0
sp|Q9UHD8-3|SEPT9_HUMAN	0.20849991	0

sp|O14787-2|TNPO2_HUMAN	0.20867538	0
sp|Q92556|ELMO1_HUMAN	0.2094593	0.35795313
sp|O75475|PSIP1_HUMAN	0.20973206	0
sp|Q9HC38-2|GLOD4_HUMAN	0.20981407	0
sp|O95479|G6PE_HUMAN	0.20981407	0.14672586
sp|Q8N163-2|CCAR2_HUMAN	0.21007633	0.30372584
sp|Q9P2X0-2|DPM3_HUMAN	0.21040726	0
sp|P62140|PP1B_HUMAN	0.21049118	0.09894868
sp|Q10713-2|MPPA_HUMAN	0.21154022	0.19149946
sp|P53990-2|IST1_HUMAN	0.211586	0
sp|Q8N392|RHG18_HUMAN	0.21194649	0
sp|P07357|CO8A_HUMAN	0.21208191	0.7759099
sp|Q9UH65|SWP70_HUMAN	0.21215439	0
sp|Q15631|TSN_HUMAN	0.21238708	0.30372584
sp|O95573|ACSL3_HUMAN	0.2123928	0
sp|P62826|RAN_HUMAN	0.21253777	0.03367973
sp|Q9NRV9|HEBP1_HUMAN	0.21342087	0.21439649
sp|Q63ZY3-3|KANK2_HUMAN	0.21342087	0.3201336
sp|P62829|RL23_HUMAN	0.213768	0
sp|P25788-2|PSA3_HUMAN	0.21467209	0.45720983
sp|O60610-2|DIAP1_HUMAN	0.21526718	0.29066643
sp|Q9UJZ1-2|STML2_HUMAN	0.21574211	0.7827403
sp|O94776|MTA2_HUMAN	0.21588516	0
sp|P00748|FA12_HUMAN	0.21629524	0.19149946
sp|P57737-4|CORO7_HUMAN	0.21717262	0
sp|Q9H3N1|TMX1_HUMAN	0.21727371	0
sp|Q8IUD2-2|RB6I2_HUMAN	0.21775436	0
sp|Q99832|TCPH_HUMAN	0.21782684	0.02662771
sp|O60831|PRAF2_HUMAN	0.21858025	0.35795313
sp|Q92878-2|RAD50_HUMAN	0.2191124	0
sp|Q9UBE0|SAE1_HUMAN	0.21956348	0.35193655
sp|Q9H3H3-1|CK068_HUMAN	0.21964073	0
sp|Q9UPN3|MACF1_HUMAN	0.21984291	0
sp|P33176|KINH_HUMAN	0.220644	0.85449874
sp|Q7Z4H8|PLGT3_HUMAN	0.2206459	0.7588735
sp|Q8TDL5|BPIB1_HUMAN	0.2213459	0.17171621
sp|P17858-2|PFKAL_HUMAN	0.22171783	0.4490988
sp|Q8IUX7|AEBP1_HUMAN	0.22190094	0
sp|P23946|CMA1_HUMAN	0.22190666	0.69308156
sp|Q14258|TRI25_HUMAN	0.22211647	0.48353976
sp|O00571-2|DDX3X_HUMAN	0.22223663	0.2178309
sp|P62854|RS26_HUMAN	0.22249603	0.7827403
sp|P02794|FRIH_HUMAN	0.22273827	0.14672586
sp|P67936|TPM4_HUMAN	0.2229023	0.01819076
sp|Q7L576|CYFP1_HUMAN	0.22320938	0

sp|P50502|F10A1_HUMAN	0.223526	0.04454162
sp|P43686|PRS6B_HUMAN	0.22371578	0.04454162
sp|Q13838-2|DX39B_HUMAN	0.22430038	0.1500082
sp|P14550|AK1A1_HUMAN	0.22436523	0.13745423
sp|O00478-2|BT3A3_HUMAN	0.22466469	0.19149946
sp|P0C0S5|H2AZ_HUMAN	0.22473907	0.656254
sp|Q13618-2|CUL3_HUMAN	0.22502136	0.7217418
sp|P31689|DNJA1_HUMAN	0.22504807	0.35795313
sp|P16152|CBR1_HUMAN	0.22513008	0.02388411
sp|Q96AQ6-2|PBIP1_HUMAN	0.22566032	0.3005443
sp|P00492|HPRT_HUMAN	0.22631073	0.7588735
sp|Q02878|RL6_HUMAN	0.22691154	0.45033538
sp|Q14019|COTL1_HUMAN	0.22704124	0.91272414
sp|Q9BZZ5-3|API5_HUMAN	0.22716713	0.09339783
sp|O15254-2|ACOX3_HUMAN	0.22736645	0
sp|P23396|RS3_HUMAN	0.22744751	1.2535655
sp|P31150|GDIA_HUMAN	0.22749329	0
sp|P09417|DHPR_HUMAN	0.22812653	0
sp|Q9H3K6-2|BOLA2_HUMAN	0.22838974	0
sp|P55209-2|NP1L1_HUMAN	0.2285862	0.43633315
sp|Q13438-4|OS9_HUMAN	0.22907448	0
sp|Q96JJ3-3|ELMO2_HUMAN	0.22942543	0
sp|P28161|GSTM2_HUMAN	0.23040771	0.7827403
sp|Q8TD19|NEK9_HUMAN	0.23049736	0
sp|Q16181-2|SEPT7_HUMAN	0.2309246	0.2746514
sp|P14317|HCLS1_HUMAN	0.23214531	0.19149946
sp|P02671-2|FIBA_HUMAN	0.2321949	0.35838646
sp|Q9Y6N5|SQOR_HUMAN	0.23241806	1.1470993
sp|Q13642-1|FHL1_HUMAN	0.23365402	0
sp|Q9UHB6-4|LIMA1_HUMAN	0.2339611	0.14672586
sp|P22352|GPX3_HUMAN	0.2344017	0
sp|P46109|CRKL_HUMAN	0.23484802	0.09894868
sp|Q13363|CTBP1_HUMAN	0.23516846	0
sp|Q7Z5L7-2|PODN_HUMAN	0.23582458	0.5204253
sp|Q9BUF5|TBB6_HUMAN	0.23608017	0.09894868
sp|Q9H2G2-2|SLK_HUMAN	0.23611069	0
sp|Q08170|SRSF4_HUMAN	0.23633385	0.656254
sp|P62834|RAP1A_HUMAN	0.23634338	0
sp|P55265-5|DSRAD_HUMAN	0.23661804	0.45033538
sp|P36269-2|GGT5_HUMAN	0.23692322	1.2392054
sp|P43243|MATR3_HUMAN	0.2369976	0.52598757
sp|O75116|ROCK2_HUMAN	0.2379036	0
sp|P18084|ITB5_HUMAN	0.23832703	0.7061832
sp|P30046|DOPD_HUMAN	0.2390995	0.19149946
sp|P11498|PYC_HUMAN	0.23952103	0.312067

sp|Q7KZF4|SND1_HUMAN	0.23985481	0
sp|P62195|PRS8_HUMAN	0.23994827	0
sp|Q8IUZ5|AT2L2_HUMAN	0.24009514	0
sp|P02675|FIBB_HUMAN	0.24020004	0.19687796
sp|Q8WYA6-2|CTBL1_HUMAN	0.24025154	0.19149946
sp|O15355|PPM1G_HUMAN	0.24036789	0
sp|O96000-2|NDUBA_HUMAN	0.24045944	0.7061832
sp|Q15436|SC23A_HUMAN	0.24092865	0.04454162
sp|Q14BN4-2|SLMAP_HUMAN	0.24316406	0
sp|Q96C86|DCPS_HUMAN	0.24502563	0.29066643
sp|Q04637-4|IF4G1_HUMAN	0.24633217	0.21439649
sp|Q96IJ6-2|GMPPA_HUMAN	0.2467537	0.7498006
sp|Q99758|ABCA3_HUMAN	0.24783325	0.29066643
sp|P68366-2|TBA4A_HUMAN	0.24818993	0
sp|Q06124-2|PTN11_HUMAN	0.24838257	0.21439649
sp|Q99961-2|SH3G1_HUMAN	0.25049782	0
sp|Q13425|SNTB2_HUMAN	0.25079155	0.20467198
sp|P32456|GBP2_HUMAN	0.25165176	0
sp|P11142|HSP7C_HUMAN	0.2519436	0.08300373
sp|Q15293|RCN1_HUMAN	0.2541275	0
sp|Q01813|PFKAP_HUMAN	0.25429153	0.10861364
sp|P14923|PLAK_HUMAN	0.25447083	0.14672586
sp|P35573|GDE_HUMAN	0.25450134	0.7061832
sp|Q13177|PAK2_HUMAN	0.25463104	0
sp|P29350-4|PTN6_HUMAN	0.25538254	0.8339169
sp|P02679-2|FIBG_HUMAN	0.25559616	0.09165865
sp|P07358|CO8B_HUMAN	0.2557125	0.8983557
sp|O14558|HSPB6_HUMAN	0.25616837	0.35193655
sp|P62249|RS16_HUMAN	0.2566719	0.7827403
sp|Q7Z4I7-3|LIMS2_HUMAN	0.25737762	0.2178309
sp|P18077|RL35A_HUMAN	0.25751495	0.7827403
sp|P02533|K1C14_HUMAN	0.25759315	0.656254
sp|Q16576-2|RBBP7_HUMAN	0.25762558	0
sp|O14672|ADA10_HUMAN	0.25781822	0
sp|Q8TDZ2-4|MICA1_HUMAN	0.2581215	0
sp|P17844-2|DDX5_HUMAN	0.25942993	0.312067
sp|P10644|KAP0_HUMAN	0.25967598	0.63371044
sp|P42704|LPPRC_HUMAN	0.26208305	0.40256184
sp|Q9H3U1-2|UN45A_HUMAN	0.2625847	0
sp|Q9Y266|NUDC_HUMAN	0.26268005	0.09894868
sp|P05783|K1C18_HUMAN	0.26273727	1.08751
sp|P28062-2|PSB8_HUMAN	0.26286125	0.97209185
sp|Q8WXH0-2|SYNE2_HUMAN	0.26352692	0.312067
sp|P02792|FRIL_HUMAN	0.2653904	0
sp|Q00341|VIGLN_HUMAN	0.26558113	1.3006523

sp|O15061-2|SYNEM_HUMAN	0.26927948	1.120602
sp|P62917|RL8_HUMAN	0.2694397	0.90036625
sp|O95816|BAG2_HUMAN	0.27159882	0.7827403
sp|P31946|1433B_HUMAN	0.27175903	0
sp|P62081|RS7_HUMAN	0.27291298	0.35795313
sp|P07203|GPX1_HUMAN	0.27308846	0.56808305
sp|Q14195-2|DPYL3_HUMAN	0.2732315	0.8397521
sp|P62750|RL23A_HUMAN	0.27324486	0.7827403
sp|O75323|NIPS2_HUMAN	0.27415466	0.7061832
sp|P24844|MYL9_HUMAN	0.27492905	0.7061832
sp|P07951|TPM2_HUMAN	0.27505875	1.071201
sp|Q9Y570-2|PPME1_HUMAN	0.27534866	0.6070219
sp|Q9UNM6-2|PSD13_HUMAN	0.27666473	0
sp|P54577|SYYC_HUMAN	0.27731705	0.61034113
sp|Q9UBT2|SAE2_HUMAN	0.27773857	0.7217418
sp|Q9BWM7|SFXN3_HUMAN	0.27791023	0
sp|Q9Y295|DRG1_HUMAN	0.27881432	0.19149946
sp|P36955|PEDF_HUMAN	0.2808323	0.47871065
sp|P55058|PLTP_HUMAN	0.28089905	0
sp|P08123|CO1A2_HUMAN	0.28119946	0.26737198
sp|P08708|RS17_HUMAN	0.28144264	0.21439649
sp|O75964|ATP5L_HUMAN	0.28146362	0.45033538
sp|Q06210-2|GFPT1_HUMAN	0.28191948	0.6634523
sp|P39059|COFA1_HUMAN	0.28256416	0.06291623
sp|O14579|COPE_HUMAN	0.28272057	0.43103603
sp|P51911|CNN1_HUMAN	0.2827778	1.1054204
sp|O75489|NDUS3_HUMAN	0.28310776	0.35795313
sp|P26640|SYVC_HUMAN	0.28361893	0.44572112
sp|Q13630|FCL_HUMAN	0.28578568	0.35795313
sp|P30837|AL1B1_HUMAN	0.2908497	0.84879977
sp|P02461|CO3A1_HUMAN	0.29183388	0
sp|Q13885|TBB2A_HUMAN	0.2921772	0.656254
sp|Q02750|MP2K1_HUMAN	0.29278183	0
sp|P46459|NSF_HUMAN	0.29379272	0.09339783
sp|P22059|OSBP1_HUMAN	0.29489136	0.06291623
sp|Q13418|ILK_HUMAN	0.29777145	0.32654193
sp|P09493-5|TPM1_HUMAN	0.2980709	0.45033538
sp|P14866|HNRPL_HUMAN	0.29821968	0
sp|Q13547|HDAC1_HUMAN	0.29824066	0
sp|Q13435|SF3B2_HUMAN	0.29841423	0.19149946
sp|Q04446|GLGB_HUMAN	0.29878235	0.2591514
sp|P39023|RL3_HUMAN	0.29898643	0.19149946
sp|P54619-2|AAKG1_HUMAN	0.2994213	0.19149946
sp|Q9C0C2-2|TB182_HUMAN	0.3013668	0.25789237
sp|P40429|RL13A_HUMAN	0.30141068	0.7061832

sp|P06681|CO2_HUMAN	0.30257034	0.68888646
sp|Q969V3-2|NCLN_HUMAN	0.30286217	0.5111962
sp|O95394-3|AGM1_HUMAN	0.30306625	0
sp|P49721|PSB2_HUMAN	0.3036213	0.656254
sp|P15374|UCHL3_HUMAN	0.30438805	0
sp|P26196|DDX6_HUMAN	0.3047905	0.2178309
sp|P49959-3|MRE11_HUMAN	0.30516052	0.45033538
sp|Q96JB5-4|CK5P3_HUMAN	0.3065281	0.06291623
sp|P09493-8|TPM1_HUMAN	0.30774307	0.7217418
sp|Q8TCJ2|STT3B_HUMAN	0.30889797	0.45033538
sp|Q99733-2|NP1L4_HUMAN	0.30986214	0.7061832
sp|P46779-2|RL28_HUMAN	0.31196404	0.7827403
sp|P0DP03|HV335_HUMAN	0.31224823	1.1932944
sp|P31146|COR1A_HUMAN	0.31550598	1.1610394
sp|P62333|PRS10_HUMAN	0.3156662	0.43103603
sp|Q14847|LASP1_HUMAN	0.31800652	0.29066643
sp|P18085|ARF4_HUMAN	0.31827164	1.1932944
sp|P50135|HNMT_HUMAN	0.31863976	0
sp|P20810-10|ICAL_HUMAN	0.31994057	0.5204253
sp|Q7Z3D6-3|GLUCM_HUMAN	0.32012177	0
sp|Q9P2B2|FPRP_HUMAN	0.32089615	0.21439649
sp|P49841-2|GSK3B_HUMAN	0.32151413	0.312067
sp|P13716-2|HEM2_HUMAN	0.3232994	0.8675019
sp|O60749-2|SNX2_HUMAN	0.3239193	0.41804093
sp|Q9NVA2|SEP11_HUMAN	0.32639885	0.86708647
sp|Q9NUV9|GIMA4_HUMAN	0.32798004	0.56808305
sp|P42226|STAT6_HUMAN	0.3289299	0.35795313
sp|P50914|RL14_HUMAN	0.32924843	0.7061832
sp|Q9NVD7|PARVA_HUMAN	0.3293476	1.2607617
sp|P05155-2|IC1_HUMAN	0.3317032	1.120602
sp|P08237-3|PFKAM_HUMAN	0.33358192	0.1342476
sp|P11166|GTR1_HUMAN	0.33421707	1.1932944
sp|Q9H4M9|EHD1_HUMAN	0.33451462	0.63371044
sp|P36542-2|ATPG_HUMAN	0.33499432	0.656254
sp|P0CG38|POTEI_HUMAN	0.33578873	0
sp|P48681|NEST_HUMAN	0.33594894	1.2435468
sp|Q9Y2Q3-3|GSTK1_HUMAN	0.33605957	0.40256184
sp|P27816-2|MAP4_HUMAN	0.33747292	0
sp|P11586|C1TC_HUMAN	0.3394165	0.68888646
sp|P01624|KV315_HUMAN	0.34037018	1.1932944
sp|P60891|PRPS1_HUMAN	0.34109497	0.19149946
sp|Q9UNZ2-5|NSF1C_HUMAN	0.3426094	1.0645995
sp|Q99584|S10AD_HUMAN	0.34304428	0.7827403
sp|Q9BS40|LXN_HUMAN	0.34340668	0
sp|O43488|ARK72_HUMAN	0.3452282	0.25789237

sp|Q9BXN1|ASPN_HUMAN	0.34817123	1.2095301
sp|P01591|IGJ_HUMAN	0.35033607	0
sp|P31321|KAP1_HUMAN	0.3506508	0
sp|A0A0C4DH31|HV118_HUMAN	0.35128403	0
sp|P62277|RS13_HUMAN	0.351511	0.19149946
sp|A0A0C4DH25|KVD20_HUMAN	0.35276794	0.656254
sp|Q13976|KGP1_HUMAN	0.35443115	1.1240381
sp|P31946-2|1433B_HUMAN	0.35684204	0
sp|Q96EM0|T3HPD_HUMAN	0.35966873	0
sp|Q9NTK5|OLA1_HUMAN	0.3610382	0.7588735
sp|P62753|RS6_HUMAN	0.36270714	0.97209185
sp|P02753|RET4_HUMAN	0.36383438	0.7827403
sp|O94925-3|GLSK_HUMAN	0.36431694	0.45033538
sp|P02747|C1QC_HUMAN	0.36667252	0.45033538
sp|P35612-2|ADDB_HUMAN	0.3678589	0
sp|Q9Y4G6|TLN2_HUMAN	0.36842346	0.19149946
sp|Q9UHB9-4|SRP68_HUMAN	0.37118912	0.28516325
sp|O00233-2|PSMD9_HUMAN	0.374897	0
sp|Q6UW68|TM205_HUMAN	0.37563896	1.1932944
sp|A1L4H1|SRCRL_HUMAN	0.3769207	0.19149946
sp|P0C0L5|CO4B_HUMAN	0.3833351	0.656254
sp|Q96RF0-2|SNX18_HUMAN	0.38542175	0.7827403
sp|P51570-2|GALK1_HUMAN	0.38601494	0.45033538
sp|P41240|CSK_HUMAN	0.3868847	0.45033538
sp|Q8WU39|MZB1_HUMAN	0.38708115	1.1505735
sp|P00739-2|HPTR_HUMAN	0.3934536	1.3006523
sp|O14974-3|MYPT1_HUMAN	0.39671326	1.1240381
sp|P02746|C1QB_HUMAN	0.39683914	1.1932944
sp|Q13347|EIF3I_HUMAN	0.397295	1.1505735
sp|P54652|HSP72_HUMAN	0.3976593	0.656254
sp|P01743|HV146_HUMAN	0.3982334	0.7827403
sp|Q0ZGT2-4|NEXN_HUMAN	0.39832687	0.656254
sp|Q8TCS8|PNPT1_HUMAN	0.39937973	0.6070219
sp|Q3SY69|AL1L2_HUMAN	0.39947128	1.1932944
sp|O94804|STK10_HUMAN	0.39949036	0.656254
sp|P51692|STA5B_HUMAN	0.40053654	0.656254
sp|Q9NWV4|CZIB_HUMAN	0.4018135	0.19149946
sp|P07951-3|TPM2_HUMAN	0.40216064	1.0485198
sp|Q9BV20|MTNA_HUMAN	0.40229225	0.45033538
sp|P35813-3|PPM1A_HUMAN	0.40454865	0.656254
sp|P0DOX2|IGA2_HUMAN	0.40766144	0.7827403
sp|P08134|RHOC_HUMAN	0.41060257	0.45033538
sp|Q92629-2|SGCD_HUMAN	0.41332436	0.656254
sp|P06396|GELS_HUMAN	0.42172623	0.656254
sp|Q8WX93-5|PALLD_HUMAN	0.42388153	0.6828178

sp|Q562R1|ACTBL_HUMAN	0.42864227	0.35795313
sp|O60488-2|ACSL4_HUMAN	0.42946815	0.656254
sp|Q00013-2|EM55_HUMAN	0.43194008	0.7827403
sp|P31513|FMO3_HUMAN	0.4336319	0.7827403
sp|P20338|RAB4A_HUMAN	0.44079208	0.656254
sp|P31947-2|1433S_HUMAN	0.44242096	0
sp|P12814-2|ACTN1_HUMAN	0.44337845	0.45033538
sp|P48426-2|PI42A_HUMAN	0.44906044	1.1932944
sp|P29692|EF1D_HUMAN	0.4495449	0.656254
sp|O43396|TXNL1_HUMAN	0.45003128	0.7827403
sp|P62851|RS25_HUMAN	0.45196915	1.1932944
sp|Q13200|PSMD2_HUMAN	0.47013283	0.35795313
sp|Q9UQ16-2|DYN3_HUMAN	0.4707489	0.656254
sp|O14791-2|APOL1_HUMAN	0.47918797	1.1932944
sp|P12931-2|SRC_HUMAN	0.48188782	0.312067
sp|P01834|IGKC_HUMAN	0.48706055	1.1932944
sp|P13746|1A11_HUMAN	0.49005127	1.1932944
sp|Q92599-2|SEPT8_HUMAN	0.49645805	0.6070219
sp|O43294|TGFI1_HUMAN	0.5017319	1.2095301
sp|P62244|RS15A_HUMAN	0.5030651	1.1932944
sp|P14324|FPPS_HUMAN	0.5036049	1.1932944
sp|P62633-3|CNBP_HUMAN	0.5131035	1.1932944
sp|P55083-2|MFAP4_HUMAN	0.5272312	0.7827403
sp|P50238|CRIP1_HUMAN	0.5277977	0.7827403
sp|O00560-2|SDCB1_HUMAN	0.5313816	0.45033538
sp|Q9BZQ8|NIBAN_HUMAN	0.5377636	0.7588735
sp|P48741|HSP77_HUMAN	0.5377655	0.656254
sp|P19012-2|K1C15_HUMAN	0.5450287	0.656254
sp|O94875-11|SRBS2_HUMAN	0.5538597	0.656254
sp|P05090|APOD_HUMAN	0.63890076	1.1505735
sp|P79483|DRB3_HUMAN	0.6720352	0.656254
sp|P01619|KV320_HUMAN	0.685482	0.656254
sp|Q14194-2|DPYL1_HUMAN	0.6965256	0.656254
sp|Q9UNS2|CSN3_HUMAN	0.69868755	0.656254
sp|Q05682-4|CALD1_HUMAN	0.70895386	0.656254
sp|P35637-2|FUS_HUMAN	0.71324444	0.45033538
sp|P30443|1A01_HUMAN	0.9847717	1.1932944
sp|P08779|K1C16_HUMAN	0.99220085	0.656254
sp|A0A075B6P5|KV228_HUMAN	1.0091572	0.656254
sp|Q04695|K1C17_HUMAN	1.0334282	0.656254
sp|P35542|SAA4_HUMAN	1.1694069	1.1932944
sp|Q9H6R3|ACSS3_HUMAN	1.6629009	0.45033538
sp|Q9TQE0|2B19_HUMAN	2.7001724	0.656254
