# Supplementary material for: Peptidylarginine deiminase 2 citrullinates MZB1 and promotes the secretion of IgM and IgA
Source: Front Immunol. 2023 Nov 29;14:1290585. doi: 10.3389/fimmu.2023.1290585 (PMC10716219; doi:10.3389/fimmu.2023.1290585)
Supplement: Supplementary file 8 [file DataSheet_8.pdf]

Supplmental Table 8: IPF2 vs controls

Accession #	Fold Change	p value (-log10)
sp|Q15109-10|RAGE_HUMAN	-1.7256279	2.1818786
sp|P13760|2B14_HUMAN	-1.2917519	1.6960104
sp|P22748|CAH4_HUMAN	-1.1331406	2.6576471
sp|P12429|ANXA3_HUMAN	-1.1135616	7.665951
sp|P02788|TRFL_HUMAN	-1.0902653	14.612167
sp|P59665|DEF1_HUMAN	-1.086132	2.1818786
sp|P05164-3|PERM_HUMAN	-1.0112572	11.224656
sp|P24158|PRTN3_HUMAN	-0.9918919	1.4104178
sp|P62805|H4_HUMAN	-0.9647751	6.3204336
sp|P49913|CAMP_HUMAN	-0.9553032	1.6960104
sp|P05109|S10A8_HUMAN	-0.9161873	3.5908275
sp|P28799-3|GRN_HUMAN	-0.9088364	1.6960104
sp|P08473|NEP_HUMAN	-0.8945522	1.6960104
sp|P61626|LYSC_HUMAN	-0.8684483	2.6576471
sp|P12821-2|ACE_HUMAN	-0.756012	2.6576471
sp|Q9NZA1-2|CLIC5_HUMAN	-0.7497425	3.5908275
sp|Q01469|FABP5_HUMAN	-0.7307911	4.051346
sp|P13686|PPA5_HUMAN	-0.7250938	1.6960104
sp|P08311|CATG_HUMAN	-0.7188339	4.051346
sp|P33121-3|ACSL1_HUMAN	-0.7165508	2.1818786
sp|P20160|CAP7_HUMAN	-0.7151642	1.6960104
sp|P80188|NGAL_HUMAN	-0.7100925	3.1266599
sp|P01011|AACT_HUMAN	-0.6720238	8.062322
sp|Q10589-2|BST2_HUMAN	-0.6703453	1.6960104
sp|Q9Y624|JAM1_HUMAN	-0.6591282	2.6576471
sp|P50895|BCAM_HUMAN	-0.6583881	6.614138
sp|P05107|ITB2_HUMAN	-0.6550903	4.3153405
sp|P41218|MNDA_HUMAN	-0.6545639	2.1818786
sp|P06702|S10A9_HUMAN	-0.6513081	3.5908275
sp|P07339|CATD_HUMAN	-0.647377	7.556109
sp|Q08722-2|CD47_HUMAN	-0.6399117	1.6960104
sp|P35241|RADI_HUMAN	-0.6261978	3.5908275
sp|Q9HB40|RISC_HUMAN	-0.6249676	1.6960104
sp|P31949|S10AB_HUMAN	-0.622303	2.6576471
sp|P09467|F16P1_HUMAN	-0.6203499	5.338249
sp|Q9UGT4|SUSD2_HUMAN	-0.6191273	2.9310203
sp|O15400-2|STX7_HUMAN	-0.6105537	1.6997428
sp|P02786|TFR1_HUMAN	-0.602993	4.051346
sp|P26447|S10A4_HUMAN	-0.59585	2.6576471
sp|Q02218-2|ODO1_HUMAN	-0.5951948	4.051346
sp|P17213|BPI_HUMAN	-0.5940933	2.1818786
sp|Q13740-2|CD166_HUMAN	-0.5887718	4.964395
sp|O95810|CAVN2_HUMAN	-0.5867577	3.8875895

sp|P61106|RAB14_HUMAN	-0.5724449	4.6186957
sp|O00159-3|MYO1C_HUMAN	-0.5696297	7.5025163
sp|P55290-4|CAD13_HUMAN	-0.5688648	1.9647322
sp|P05362|ICAM1_HUMAN	-0.5599136	5.869831
sp|P48735-2|IDHP_HUMAN	-0.5597343	4.051346
sp|P60903|S10AA_HUMAN	-0.5577927	1.6960104
sp|P18428|LBP_HUMAN	-0.5432129	1.6960104
sp|P30512|1A29_HUMAN	-0.5414009	1.6960104
sp|P02750|A2GL_HUMAN	-0.5298786	3.1266599
sp|Q86VB7-2|C163A_HUMAN	-0.5298538	8.461897
sp|Q9UHG3|PCYOX_HUMAN	-0.5223026	5.869831
sp|P51149|RAB7A_HUMAN	-0.5150337	4.7039127
sp|P43304|GPDM_HUMAN	-0.5018482	3.5908275
sp|Q6NZI2|CAVN1_HUMAN	-0.5017586	5.2874084
sp|P11233|RALA_HUMAN	-0.4967766	2.1818786
sp|Q9H0U4|RAB1B_HUMAN	-0.4911404	1.6960104
sp|P49756|RBM25_HUMAN	-0.4866924	1.6960104
sp|P04083|ANXA1_HUMAN	-0.482254	8.788385
sp|P16671-4|CD36_HUMAN	-0.4664345	2.1818786
sp|Q02318|CP27A_HUMAN	-0.4642639	1.6960104
sp|P84103-2|SRSF3_HUMAN	-0.4634495	1.9413493
sp|O96009|NAPSA_HUMAN	-0.4551907	2.6576471
sp|P51148-2|RAB5C_HUMAN	-0.4532757	1.6960104
sp|Q8N335|GPD1L_HUMAN	-0.4524803	1.6960104
sp|P21397|AOFA_HUMAN	-0.452034	5.2991114
sp|P09668|CATH_HUMAN	-0.449564	4.051346
sp|Q9UL25|RAB21_HUMAN	-0.4485207	2.6576471
sp|Q13510-2|ASAH1_HUMAN	-0.446228	6.3204336
sp|Q15907-2|RB11B_HUMAN	-0.4461498	3.5908275
sp|P49407-2|ARRB1_HUMAN	-0.4421082	2.6576471
sp|P05091|ALDH2_HUMAN	-0.4404621	11.4588375
sp|Q03135|CAV1_HUMAN	-0.439415	2.1818786
sp|P04179-4|SODM_HUMAN	-0.4362774	3.1266599
sp|Q9NZN4|EHD2_HUMAN	-0.434248	7.568668
sp|Q8WWI1-5|LMO7_HUMAN	-0.4316025	4.0772076
sp|Q14152|EIF3A_HUMAN	-0.4182596	1.6960104
sp|P27105|STOM_HUMAN	-0.4175072	4.964395
sp|P50148|GNAQ_HUMAN	-0.4168739	2.1818786
sp|P52790|HXK3_HUMAN	-0.4167614	2.6576471
sp|Q13451|FKBP5_HUMAN	-0.4161224	2.3979244
sp|P07910-2|HNRPC_HUMAN	-0.4157391	1.9647322
sp|Q07157|ZO1_HUMAN	-0.4149551	4.789934
sp|P14780|MMP9_HUMAN	-0.4136486	2.768635
sp|P09758|TACD2_HUMAN	-0.4130325	2.1818786
sp|O15247|CLIC2_HUMAN	-0.4112949	2.9807727

sp|P09622|DLDH_HUMAN	-0.4094753	3.8058946
sp|P07355-2|ANXA2_HUMAN	-0.4086952	4.247887
sp|P08758|ANXA5_HUMAN	-0.4069061	9.697888
sp|P16284-3|PECA1_HUMAN	-0.4043922	5.3137383
sp|P09525|ANXA4_HUMAN	-0.3923779	6.3667836
sp|Q96TA1-2|NIBL1_HUMAN	-0.3910923	3.4656596
sp|P22307-8|NLTP_HUMAN	-0.3891945	1.6960104
sp|O75695|XRP2_HUMAN	-0.3874474	1.6960104
sp|Q14344|GNA13_HUMAN	-0.3853264	1.6604291
sp|P09601|HMOX1_HUMAN	-0.3826771	1.3815327
sp|Q6PIU2-2|NCEH1_HUMAN	-0.3822441	2.6997027
sp|P36543-2|VATE1_HUMAN	-0.3811817	1.6960104
sp|Q86Y82|STX12_HUMAN	-0.3741388	2.6576471
sp|P05141|ADT2_HUMAN	-0.3727665	2.6576471
sp|Q6P4A8|PLBL1_HUMAN	-0.3720665	2.1818786
sp|A6NMZ7|CO6A6_HUMAN	-0.3601131	3.364204
sp|P60953|CDC42_HUMAN	-0.3596115	3.1266599
sp|P61421|VA0D1_HUMAN	-0.3570557	3.4656596
sp|P20292|AL5AP_HUMAN	-0.3556423	1.6960104
sp|P62987|RL40_HUMAN	-0.3548241	1.8444856
sp|P30711|GSTT1_HUMAN	-0.3541927	1.6960104
sp|P23786|CPT2_HUMAN	-0.3540115	1.3815327
sp|Q13228-4|SBP1_HUMAN	-0.3524017	11.411416
sp|P00167-2|CYB5_HUMAN	-0.3512821	1.5646582
sp|Q9H4M9|EHD1_HUMAN	-0.3494434	3.1208909
sp|P11413-2|G6PD_HUMAN	-0.3465729	4.033051
sp|O95994|AGR2_HUMAN	-0.3452816	2.6576471
sp|P51659|DHB4_HUMAN	-0.3424549	12.043698
sp|P04080|CYTB_HUMAN	-0.3418655	1.6960104
sp|Q9H223|EHD4_HUMAN	-0.3387985	4.051346
sp|O75955|FLOT1_HUMAN	-0.3385468	4.964395
sp|P11215-2|ITAM_HUMAN	-0.3368759	3.9417877
sp|P20340-2|RAB6A_HUMAN	-0.336443	3.9417877
sp|P36957|ODO2_HUMAN	-0.3356056	4.509013
sp|P0DP25|CALM3_HUMAN	-0.3346577	3.342776
sp|P06737-2|PYGL_HUMAN	-0.3341904	4.984084
sp|P47897|SYQ_HUMAN	-0.33354	1.8444856
sp|Q99536|VAT1_HUMAN	-0.33214	3.6087239
sp|P08571|CD14_HUMAN	-0.3320236	2.1818786
sp|Q9NYL9|TMOD3_HUMAN	-0.3317699	2.1818786
sp|P01009|A1AT_HUMAN	-0.3310165	7.629475
sp|P62258|1433E_HUMAN	-0.3281994	2.1818786
sp|Q7Z406|MYH14_HUMAN	-0.3263702	8.664913
sp|Q14254|FLOT2_HUMAN	-0.3255634	3.1266599
sp|P51648-2|AL3A2_HUMAN	-0.32411	2.56956

sp|P61604|CH10_HUMAN	-0.3238106	3.1266599
sp|Q9Y696|CLIC4_HUMAN	-0.3236885	1.6295799
sp|O43760-2|SNG2_HUMAN	-0.3228951	1.6960104
sp|O15230|LAMA5_HUMAN	-0.3220711	6.478972
sp|P15586-2|GNS_HUMAN	-0.3220329	1.6960104
sp|P10301|RRAS_HUMAN	-0.3171806	1.6960104
sp|P13987-2|CD59_HUMAN	-0.3139648	1.6960104
sp|P05026-2|AT1B1_HUMAN	-0.3132649	2.2323174
sp|Q9NP72|RAB18_HUMAN	-0.31213	2.1818786
sp|P14543-2|NID1_HUMAN	-0.3086853	5.888033
sp|Q92817|EVPL_HUMAN	-0.3063088	4.461395
sp|P09110|THIK_HUMAN	-0.3050613	2.1818786
sp|Q04760-2|LGUL_HUMAN	-0.3044758	1.9647322
sp|O60437|PEPL_HUMAN	-0.3036041	13.481833
sp|P10809|CH60_HUMAN	-0.3033466	7.5463896
sp|O00571-2|DDX3X_HUMAN	-0.301508	1.6960104
sp|Q16698-2|DECR_HUMAN	-0.2976704	4.033051
sp|P62820|RAB1A_HUMAN	-0.2963791	1.9647322
sp|P26440|IVD_HUMAN	-0.295887	1.6960104
sp|Q16881-2|TRXR1_HUMAN	-0.2932472	3.5705152
sp|Q15599-2|NHRF2_HUMAN	-0.2920933	2.3979244
sp|P05556|ITB1_HUMAN	-0.291729	8.448556
sp|P20073-2|ANXA7_HUMAN	-0.2915325	2.653196
sp|P43490|NAMPT_HUMAN	-0.2864723	6.521583
sp|Q6NUK1-2|SCMC1_HUMAN	-0.2856922	2.4829872
sp|Q9NTX5-6|ECHD1_HUMAN	-0.2851448	2.36833
sp|P55268|LAMB2_HUMAN	-0.2850113	9.150676
sp|P17844-2|DDX5_HUMAN	-0.2839851	3.5190198
sp|P09960|LKHA4_HUMAN	-0.2836151	7.2164545
sp|P62495-2|ERF1_HUMAN	-0.2835741	1.6960104
sp|P50995-2|ANX11_HUMAN	-0.2834034	2.997126
sp|P17931|LEG3_HUMAN	-0.2828884	2.6576471
sp|P63092-3|GNAS2_HUMAN	-0.2823277	1.6295799
sp|Q9UBQ0-2|VPS29_HUMAN	-0.2806435	1.6960104
sp|P10253|LYAG_HUMAN	-0.2789974	1.827201
sp|P51688|SPHM_HUMAN	-0.2770653	2.1818786
sp|P61019|RAB2A_HUMAN	-0.2763958	3.1627245
sp|Q02252-2|MMSA_HUMAN	-0.2763901	1.3635377
sp|Q6YHK3|CD109_HUMAN	-0.2759857	4.033051
sp|Q27J81-2|INF2_HUMAN	-0.2752781	1.6960104
sp|P54886-2|P5CS_HUMAN	-0.2738628	1.4104178
sp|Q01130-2|SRSF2_HUMAN	-0.2720127	1.6960104
sp|Q00325-2|MPCP_HUMAN	-0.2700386	3.1266599
sp|O14980|XPO1_HUMAN	-0.2700291	1.6960104
sp|P00403|COX2_HUMAN	-0.2680492	1.7590232

sp|P23381|SYWC_HUMAN	-0.2676868	4.7413087
sp|Q96RQ3|MCCA_HUMAN	-0.2671375	1.7590232
sp|P11177-3|ODPB_HUMAN	-0.2636738	1.5983955
sp|Q16630-2|CPSF6_HUMAN	-0.2626495	1.6960104
sp|Q96I99|SUCB2_HUMAN	-0.2622376	4.172964
sp|Q9HDC9|APMAP_HUMAN	-0.2585583	4.5526023
sp|Q9BXS5-2|AP1M1_HUMAN	-0.2585068	2.1818786
sp|Q8WUM4|PDC6I_HUMAN	-0.2557392	3.903645
sp|P07942|LAMB1_HUMAN	-0.2554445	6.57691
sp|Q99439|CNN2_HUMAN	-0.253582	1.8444856
sp|P16278-2|BGAL_HUMAN	-0.253542	2.6576471
sp|P30740|ILEU_HUMAN	-0.2479076	2.5461748
sp|P50570-2|DYN2_HUMAN	-0.2477169	1.9413493
sp|P20645|MPRD_HUMAN	-0.2466297	1.6960104
sp|P08754|GNAI3_HUMAN	-0.2456474	1.7590232
sp|P11047|LAMC1_HUMAN	-0.2438641	5.2853217
sp|P07686|HEXB_HUMAN	-0.2436714	1.827201
sp|Q99715-4|COCA1_HUMAN	-0.2423439	1.6960104
sp|P99999|CYC_HUMAN	-0.2423248	1.9647322
sp|Q8NBX0|SCPDL_HUMAN	-0.240778	1.4104178
sp|P30040|ERP29_HUMAN	-0.238348	2.3047035
sp|P61204|ARF3_HUMAN	-0.2373276	1.9951487
sp|P42330|AK1C3_HUMAN	-0.2369413	2.5461748
sp|P37837|TALDO_HUMAN	-0.2366867	5.6821094
sp|P04632|CPNS1_HUMAN	-0.2333822	2.4723146
sp|P49327|FAS_HUMAN	-0.2314682	5.371087
sp|P00352|AL1A1_HUMAN	-0.2312241	7.6453085
sp|P62873|GBB1_HUMAN	-0.2304211	2.044628
sp|P35237|SPB6_HUMAN	-0.2301884	4.6038065
sp|P04066|FUCO_HUMAN	-0.2295513	1.6960104
sp|P02743|SAMP_HUMAN	-0.2271156	1.6265627
sp|P21796|VDAC1_HUMAN	-0.2257156	3.658668
sp|O75874|IDHC_HUMAN	-0.2232895	3.1516862
sp|P30626-2|SORCN_HUMAN	-0.2224789	2.2385595
sp|Q9UBV8|PEF1_HUMAN	-0.2217217	1.6960104
sp|P35270|SPRE_HUMAN	-0.2206287	1.4278674
sp|P42765|THIM_HUMAN	-0.2206059	4.834172
sp|Q16853|AOC3_HUMAN	-0.219223	4.0218377
sp|P0DMV9|HS71B_HUMAN	-0.2179794	3.5779264
sp|P11310-2|ACADM_HUMAN	-0.2161274	3.218121
sp|Q9NQC3|RTN4_HUMAN	-0.2137947	1.5646582
sp|Q9UFN0|NPS3A_HUMAN	-0.2134247	1.4104178
sp|P84090|ERH_HUMAN	-0.2116985	2.1818786
sp|O00264|PGRC1_HUMAN	-0.2105198	2.0727112
sp|P08621-3|RU17_HUMAN	-0.2059002	1.9951487

sp|Q04917|1433F_HUMAN	-0.205204	4.611947
sp|Q13011|ECH1_HUMAN	-0.203249	4.044672
sp|P61970|NTF2_HUMAN	-0.2018204	1.4104178
sp|P56199|ITA1_HUMAN	-0.2015724	4.1295223
sp|P05023|AT1A1_HUMAN	-0.1998196	6.057522
sp|Q16363-2|LAMA4_HUMAN	-0.199688	2.8787436
sp|Q14118|DAG1_HUMAN	-0.1993675	1.4104178
sp|P21281|VATB2_HUMAN	-0.1972427	3.218121
sp|P54920|SNAA_HUMAN	-0.1971836	3.1629772
sp|Q00765|REEP5_HUMAN	-0.1956463	3.1266599
sp|P06576|ATPB_HUMAN	-0.1949883	4.9429183
sp|P59998|ARPC4_HUMAN	-0.1938248	2.3143692
sp|Q92542|NICA_HUMAN	-0.1933289	1.4104178
sp|Q01082|SPTB2_HUMAN	-0.1895866	14.374806
sp|P04040|CATA_HUMAN	-0.1880341	6.8146358
sp|O15031|PLXB2_HUMAN	-0.1864014	2.7155995
sp|P38606|VATA_HUMAN	-0.1859074	2.8451047
sp|P49755|TMEDA_HUMAN	-0.1838064	1.7590232
sp|P06733|ENOA_HUMAN	-0.1832981	1.3454111
sp|P43121|MUC18_HUMAN	-0.1827107	3.316884
sp|P29401-2|TKT_HUMAN	-0.1823311	7.6846213
sp|P09497-2|CLCB_HUMAN	-0.1809406	1.9647322
sp|P49961-6|ENTP1_HUMAN	-0.180521	1.4104178
sp|P24752|THIL_HUMAN	-0.1797218	2.0740998
sp|P46939-2|UTRO_HUMAN	-0.1790505	3.3572266
sp|P19971|TYPH_HUMAN	-0.178997	4.339341
sp|P35221|CTNA1_HUMAN	-0.1780167	6.2760777
sp|P13804|ETFA_HUMAN	-0.1768112	2.845279
sp|O00764-2|PDXK_HUMAN	-0.1746101	3.1266599
sp|P62993|GRB2_HUMAN	-0.1740894	1.4050349
sp|P51572-2|BAP31_HUMAN	-0.173645	1.515701
sp|Q9Y277-2|VDAC3_HUMAN	-0.1723862	1.3957126
sp|P38646|GRP75_HUMAN	-0.171669	3.9272358
sp|O75348|VATG1_HUMAN	-0.1716251	1.6960104
sp|P25789|PSA4_HUMAN	-0.1670742	3.671968
sp|P49748-2|ACADV_HUMAN	-0.1667252	4.8317146
sp|P50213|IDH3A_HUMAN	-0.1646729	1.6092666
sp|P07954-2|FUMH_HUMAN	-0.1627808	1.4278674
sp|P35579|MYH9_HUMAN	-0.1614037	9.9857025
sp|P50552|VASP_HUMAN	-0.1589184	2.1800864
sp|Q9UPN3|MACF1_HUMAN	-0.1577759	2.9337258
sp|P23634-8|AT2B4_HUMAN	-0.1571121	1.4351699
sp|Q99729-3|ROAA_HUMAN	-0.1566277	1.4548165
sp|Q9Y224|RTRAF_HUMAN	-0.1559868	1.6997428
sp|Q14112-2|NID2_HUMAN	-0.1552677	1.8087089

sp|Q02818|NUCB1_HUMAN	-0.1545677	2.2535503
sp|O95831-3|AIFM1_HUMAN	-0.1539974	1.8842831
sp|Q08945|SSRP1_HUMAN	-0.1538162	2.3143692
sp|P35232|PHB_HUMAN	-0.1537247	2.8793902
sp|Q9NZ08-2|ERAP1_HUMAN	-0.1537247	2.544769
sp|P23141-2|EST1_HUMAN	-0.1518459	5.832636
sp|P27824-2|CALX_HUMAN	-0.151516	2.400925
sp|O60234|GMFG_HUMAN	-0.1506901	1.6960104
sp|O75390|CISY_HUMAN	-0.1499577	1.8842831
sp|Q6UWY5|OLFL1_HUMAN	-0.1494217	1.4564189
sp|Q15417|CNN3_HUMAN	-0.1488609	1.4104178
sp|O43813|LANC1_HUMAN	-0.1451626	1.4104178
sp|Q9ULA0|DNPEP_HUMAN	-0.1445732	3.0139608
sp|Q9BSJ8-2|ESYT1_HUMAN	-0.1440144	4.3259945
sp|P09382|LEG1_HUMAN	-0.1435814	3.364204
sp|Q9NRN5-2|OLFL3_HUMAN	-0.1423779	2.817951
sp|Q16658|FSCN1_HUMAN	-0.14118	2.6947098
sp|P07988|PSPB_HUMAN	-0.1408291	1.5983955
sp|P16435|NCPR_HUMAN	-0.1407766	1.9076298
sp|Q03252|LMNB2_HUMAN	-0.1400757	5.6768107
sp|P08575-10|PTPRC_HUMAN	-0.1399841	1.4936475
sp|Q06830|PRDX1_HUMAN	-0.1396637	2.2243123
sp|P30520|PURA2_HUMAN	-0.1390762	1.827201
sp|P52209-2|6PGD_HUMAN	-0.1364975	1.6613601
sp|P04275|VWF_HUMAN	-0.13554	2.2135153
sp|Q99623|PHB2_HUMAN	-0.1335564	1.4068714
sp|P08572|CO4A2_HUMAN	-0.1331673	1.7451575
sp|Q9HCC0|MCCB_HUMAN	-0.1323891	2.1800864
sp|P54819-2|KAD2_HUMAN	-0.1272316	1.4104178
sp|Q99460|PSMD1_HUMAN	-0.1252823	1.3908802
sp|P35914|HMGCL_HUMAN	-0.1235848	1.6997428
sp|P40926|MDHM_HUMAN	-0.1230812	3.3269932
sp|P52907|CAZA1_HUMAN	-0.1210232	1.4351699
sp|P13073|COX41_HUMAN	-0.1168461	1.4104178
sp|P35580-3|MYH10_HUMAN	-0.1133843	6.249163
sp|P00390-2|GSHR_HUMAN	-0.1132965	1.7713763
sp|P00505|AATM_HUMAN	-0.1114922	3.15452
sp|P07099|HYEP_HUMAN	-0.1074944	1.5294853
sp|P60900|PSA6_HUMAN	-0.1056862	1.8087089
sp|P13489|RINI_HUMAN	-0.1056404	2.330168
sp|P05198|IF2A_HUMAN	-0.1053829	1.3815327
sp|P48643|TCPE_HUMAN	-0.1052704	1.9819717
sp|P13796|PLSL_HUMAN	-0.1050262	3.8791635
sp|Q96HE7|ERO1A_HUMAN	-0.1045837	1.4104178
sp|P21980|TGM2_HUMAN	-0.1040154	2.6854048

sp|P04004|VTNC_HUMAN	-0.1029739	2.3979244
sp|Q9UL46|PSME2_HUMAN	-0.1029148	1.6997428
sp|Q00839|HNRPU_HUMAN	-0.1012993	1.3379446
sp|P41250|GARS_HUMAN	-0.1006136	1.4564189
sp|Q07954|LRP1_HUMAN	-0.1005592	3.646963
sp|Q06323|PSME1_HUMAN	-0.0996857	1.5270832
sp|Q9UHQ9|NB5R1_HUMAN	-0.0961533	1.3815327
sp|Q9UIJ7|KAD3_HUMAN	-0.0938187	2.058973
sp|P61158|ARP3_HUMAN	-0.0936184	1.3801578
sp|O15144|ARPC2_HUMAN	-0.0926895	1.4050349
sp|P17655|CAN2_HUMAN	-0.0888519	2.3202872
sp|P20700|LMNB1_HUMAN	-0.0876827	2.7836905
sp|P08729|K2C7_HUMAN	-0.0860157	1.7871698
sp|Q08257|QOR_HUMAN	-0.0844765	1.5071542
sp|O60504-2|VINEX_HUMAN	-0.0815887	1.560883
sp|O14818|PSA7_HUMAN	-0.0758934	1.8842831
sp|Q9BUJ2-4|HNRL1_HUMAN	-0.0745888	1.3668759
sp|Q9Y490|TLN1_HUMAN	-0.0707417	2.8255475
sp|P55072|TERA_HUMAN	-0.0654717	1.7263757
sp|P08133|ANXA6_HUMAN	-0.0611286	1.3304803
sp|P60842|IF4A1_HUMAN	-0.0567532	1.3481187
sp|P19338|NUCL_HUMAN	-0.0540772	1.5096362
sp|P02545|LMNA_HUMAN	-0.0539246	2.5078812
sp|P12109|CO6A1_HUMAN	-0.0438404	1.4875728
sp|P11021|BIP_HUMAN	-0.0427666	1.3693259
sp|Q07065|CKAP4_HUMAN	0.06452751	1.8029867
sp|Q99497|PARK7_HUMAN	0.06971359	1.560883
sp|O00567|NOP56_HUMAN	0.06984139	1.515701
sp|P02549-2|SPTA1_HUMAN	0.07066727	1.6728406
sp|P30101|PDIA3_HUMAN	0.07391357	2.0547411
sp|P35606|COPB2_HUMAN	0.08586502	3.068567
sp|P09972|ALDOC_HUMAN	0.09036446	1.4278674
sp|Q92900-2|RENT1_HUMAN	0.09292221	1.3184075
sp|P04843|RPN1_HUMAN	0.09348106	1.7921138
sp|P18206-2|VINC_HUMAN	0.09399796	6.316927
sp|P49257|LMAN1_HUMAN	0.10253525	1.5567774
sp|P04075|ALDOA_HUMAN	0.10853195	2.3661125
sp|P31040|SDHA_HUMAN	0.1091156	1.3559021
sp|P23284|PPIB_HUMAN	0.11219978	3.5844223
sp|P27348|1433T_HUMAN	0.11577797	1.436011
sp|P40939|ECHA_HUMAN	0.11669159	1.4881507
sp|P49419-2|AL7A1_HUMAN	0.11795425	1.7404958
sp|P31943|HNRH1_HUMAN	0.12033463	1.5054473
sp|Q13263|TIF1B_HUMAN	0.12540436	1.6797456
sp|P05156|CFAI_HUMAN	0.12926865	1.4936475

sp|Q96G03|PGM2_HUMAN	0.1305542	2.0598862
sp|Q12906-4|ILF3_HUMAN	0.13311386	3.26026
sp|P67936|TPM4_HUMAN	0.1367321	2.1377194
sp|O75083|WDR1_HUMAN	0.14050102	2.4186769
sp|P04844|RPN2_HUMAN	0.14097786	3.0251918
sp|O15372|EIF3H_HUMAN	0.14101791	1.6960104
sp|Q15075|EEA1_HUMAN	0.14233398	2.015472
sp|O94776|MTA2_HUMAN	0.14594841	1.5646582
sp|P23526|SAHH_HUMAN	0.14605522	1.3503746
sp|Q9Y315|DEOC_HUMAN	0.14617729	1.5646582
sp|Q86VP6|CAND1_HUMAN	0.14670944	1.6891338
sp|P11766|ADHX_HUMAN	0.14783859	3.0495367
sp|P62316-2|SMD2_HUMAN	0.14912033	1.6960104
sp|O60884|DNJA2_HUMAN	0.14958382	1.3815327
sp|P02774-3|VTDB_HUMAN	0.14971733	3.2431045
sp|P13798|ACPH_HUMAN	0.15115356	2.747965
sp|Q15942|ZYX_HUMAN	0.15135002	1.8444856
sp|P08727|K1C19_HUMAN	0.1533699	2.6788433
sp|Q9UBT2|SAE2_HUMAN	0.1630249	1.9647322
sp|Q9HCB6|SPON1_HUMAN	0.16519356	1.3635377
sp|Q8IZ83-3|A16A1_HUMAN	0.167099	3.3703115
sp|P13645|K1C10_HUMAN	0.17503357	2.5292609
sp|O00534|VMA5A_HUMAN	0.17681313	1.6295799
sp|P25311|ZA2G_HUMAN	0.17969513	3.1693146
sp|P08670|VIME_HUMAN	0.17995453	8.414972
sp|P14625|ENPL_HUMAN	0.18278503	5.598441
sp|P11277-2|SPTB1_HUMAN	0.18607903	4.0486217
sp|P32119|PRDX2_HUMAN	0.186903	4.056798
sp|Q15582|BGH3_HUMAN	0.18848038	2.4678369
sp|P98095-2|FBLN2_HUMAN	0.18944931	1.800942
sp|P55084|ECHB_HUMAN	0.19316673	2.3305986
sp|Q16643-3|DREB_HUMAN	0.19510078	1.4716977
sp|Q14847|LASP1_HUMAN	0.19893646	1.6832623
sp|P02790|HEMO_HUMAN	0.19935799	3.729114
sp|P06681-3|CO2_HUMAN	0.2007389	2.3143692
sp|P80303-2|NUCB2_HUMAN	0.20617867	2.1818786
sp|Q0ZGT2-4|NEXN_HUMAN	0.2084961	2.923088
sp|Q5TDH0-3|DDI2_HUMAN	0.2179985	1.4104178
sp|Q8IUX7|AEBP1_HUMAN	0.22303009	1.4050349
sp|Q92896-2|GSLG1_HUMAN	0.22632027	4.051346
sp|P36955|PEDF_HUMAN	0.22869301	2.3143692
sp|P15090|FABP4_HUMAN	0.23130417	2.36833
sp|P10909-5|CLUS_HUMAN	0.23461723	4.534284
sp|P02042|HBD_HUMAN	0.24206161	2.3979244
sp|P01008|ANT3_HUMAN	0.24249268	4.937407

sp|P28331-2|NDUS1_HUMAN	0.24377632	1.560883
sp|P35555|FBN1_HUMAN	0.24386978	8.268259
sp|P23219-2|PGH1_HUMAN	0.24842453	1.9647322
sp|P09493-9|TPM1_HUMAN	0.25156212	1.6960104
sp|Q15746-2|MYLK_HUMAN	0.25204086	3.26026
sp|Q9UBG0|MRC2_HUMAN	0.25398064	1.8595492
sp|Q9NZU5-2|LMCD1_HUMAN	0.25655937	1.8060131
sp|P13671|CO6_HUMAN	0.25896835	1.4104178
sp|O14558|HSPB6_HUMAN	0.2592678	1.4278674
sp|Q16181-2|SEPT7_HUMAN	0.26430702	4.6295204
sp|P23946|CMA1_HUMAN	0.2674675	1.6997428
sp|Q14019|COTL1_HUMAN	0.26768875	2.6576471
sp|P62753|RS6_HUMAN	0.2694168	1.6960104
sp|P13667|PDIA4_HUMAN	0.27235413	8.084617
sp|P10643|CO7_HUMAN	0.27420807	3.1015959
sp|P04196|HRG_HUMAN	0.27703857	3.2925725
sp|P05783|K1C18_HUMAN	0.27786255	6.68389
sp|P07951|TPM2_HUMAN	0.28624153	1.3080103
sp|P03952|KLKB1_HUMAN	0.28657532	2.1818786
sp|P08708|RS17_HUMAN	0.29084015	2.3143692
sp|P51911|CNN1_HUMAN	0.2914276	2.3787699
sp|P00450|CERU_HUMAN	0.29290962	7.740138
sp|P09493-8|TPM1_HUMAN	0.2933178	2.7624686
sp|A1L4H1|SRCRL_HUMAN	0.29749107	1.6960104
sp|Q15124|PGM5_HUMAN	0.29950714	7.01345
sp|P13611|CSPG2_HUMAN	0.3089695	4.393969
sp|P36578|RL4_HUMAN	0.3185768	3.5908275
sp|P08123|CO1A2_HUMAN	0.33423042	1.6997428
sp|P62241|RS8_HUMAN	0.33778954	2.8384566
sp|P22105-1|TENX_HUMAN	0.3393097	10.5570965
sp|P04792|HSPB1_HUMAN	0.34099197	6.5900383
sp|P00488|F13A_HUMAN	0.34155083	4.984084
sp|Q93052|LPP_HUMAN	0.34933662	3.5945017
sp|Q9BXN1|ASPN_HUMAN	0.3520527	2.6576471
sp|Q9NYL4|FKB11_HUMAN	0.35588074	1.4104178
sp|Q12805-2|FBLN3_HUMAN	0.35663795	4.80653
sp|P67936-2|TPM4_HUMAN	0.35853577	1.6960104
sp|P21291|CSRP1_HUMAN	0.3588333	5.417897
sp|Q12765|SCRN1_HUMAN	0.35939598	2.1818786
sp|P00751|CFAB_HUMAN	0.36118126	8.874068
sp|P68871|HBB_HUMAN	0.36701584	2.838374
sp|P27169|PON1_HUMAN	0.36768723	1.6960104
sp|Q14195-2|DPYL3_HUMAN	0.36808968	7.605871
sp|P00918|CAH2_HUMAN	0.3697834	4.509013
sp|Q9P2B2|FPRP_HUMAN	0.3741417	3.1266599

sp|P00734|THRB_HUMAN	0.37493515	4.017758
sp|P07585|PGS2_HUMAN	0.37509537	4.2870226
sp|P06727|APOA4_HUMAN	0.3801632	4.865345
sp|P35749|MYH11_HUMAN	0.38070107	14.436076
sp|P01042-2|KNG1_HUMAN	0.38121414	5.938259
sp|Q96C86|DCPS_HUMAN	0.39722633	1.6960104
sp|Q8N2S1|LTBP4_HUMAN	0.39870453	4.0486217
sp|P01833|PIGR_HUMAN	0.4012413	4.964395
sp|P15088|CBPA3_HUMAN	0.4054451	2.1818786
sp|Q9P2E9|RRBP1_HUMAN	0.4067669	12.329277
sp|P04114|APOB_HUMAN	0.40989685	10.952644
sp|P69905|HBA_HUMAN	0.41264343	3.316884
sp|P07738|PMGE_HUMAN	0.41810226	2.1818786
sp|P05787-2|K2C8_HUMAN	0.42713928	10.888629
sp|P19827|ITIH1_HUMAN	0.42729187	4.80653
sp|P02452|CO1A1_HUMAN	0.43024635	2.058973
sp|P24844|MYL9_HUMAN	0.4360485	1.6960104
sp|P62266|RS23_HUMAN	0.44197464	2.6576471
sp|O00151|PDLI1_HUMAN	0.44431114	2.400925
sp|Q9UBX5|FBLN5_HUMAN	0.4636593	4.033051
sp|Q9NR12-2|PDLI7_HUMAN	0.4684143	2.1800864
sp|Q8WU39|MZB1_HUMAN	0.47790527	2.3047035
sp|Q13162|PRDX4_HUMAN	0.47974014	2.432837
sp|P49821-2|NDUV1_HUMAN	0.4839363	2.441399
sp|P29622|KAIN_HUMAN	0.4841652	2.6576471
sp|P01031|CO5_HUMAN	0.48708344	2.753709
sp|P02671|FIBA_HUMAN	0.49538994	8.574463
sp|Q9UMS6-2|SYNP2_HUMAN	0.4996872	2.9310203
sp|P00748|FA12_HUMAN	0.5012665	1.6960104
sp|P07451|CAH3_HUMAN	0.50237274	1.827201
sp|Q14315-2|FLNC_HUMAN	0.5033512	14.539617
sp|P00915|CAH1_HUMAN	0.5163994	5.7298145
sp|Q14767|LTBP2_HUMAN	0.5288372	4.3492994
sp|Q8TDL5|BPIB1_HUMAN	0.53274536	2.044628
sp|Q01995|TAGL_HUMAN	0.53463936	7.8047314
sp|P02675|FIBB_HUMAN	0.5466118	9.180728
sp|Q14192|FHL2_HUMAN	0.54758453	2.1818786
sp|P05546|HEP2_HUMAN	0.55908203	2.6576471
sp|P19823|ITIH2_HUMAN	0.5642929	5.417897
sp|P46779-2|RL28_HUMAN	0.5662422	2.1818786
sp|P02679-2|FIBG_HUMAN	0.57214546	6.2420893
sp|P17661|DESM_HUMAN	0.577261	14.808462
sp|Q8NBS9|TXND5_HUMAN	0.5908718	7.665951
sp|P04259|K2C6B_HUMAN	0.6235943	2.1818786
sp|P23456|Trypsin	0.64588165	3.4656596

sp|P04003|C4BPA_HUMAN	0.65763474	5.869831
sp|P13647|K2C5_HUMAN	0.6654587	6.7698927
sp|P02647|APOA1_HUMAN	0.6998539	12.519223
sp|P00747|PLMN_HUMAN	0.7055321	6.7698927
sp|P0DOX5|IGG1_HUMAN	0.8013725	4.367871
sp|P0DOY3|IGLC3_HUMAN	0.8267708	2.1818786
sp|Q05707|COEA1_HUMAN	0.9431114	15.25562
sp|P0DOX8|IGL1_HUMAN	0.9557438	2.1818786
sp|P02652-2|APOA2_HUMAN	1.0050583	2.058973
sp|P01859|IGHG2_HUMAN	1.0217228	4.4116287
sp|P01876|IGHA1_HUMAN	1.0276661	4.509013
sp|P04264|K2C1_HUMAN	1.0650234	12.332273
sp|P01860|IGHG3_HUMAN	1.3390617	2.6576471
sp|P01871-2|IGHM_HUMAN	1.3807735	2.1818786
sp|P35527|K1C9_HUMAN	1.4763031	5.728786
sp|P01861|IGHG4_HUMAN	1.5504074	3.5908275
sp|P04229|2B11_HUMAN	-3.0598888	0.656254
sp|P08246|ELNE_HUMAN	-1.7198162	1.1932944
sp|P08263|GSTA1_HUMAN	-1.5163345	0
sp|P54108-2|CRIS3_HUMAN	-1.4643812	0.656254
sp|Q29963|1C06_HUMAN	-1.4123783	0.656254
sp|P02741|CRP_HUMAN	-1.2031498	1.1932944
sp|Q03135-2|CAV1_HUMAN	-1.1924057	0.656254
sp|P13761|2B17_HUMAN	-1.1225281	0
sp|Q9HD89|RETN_HUMAN	-1.0432739	1.1932944
sp|Q16777|H2A2C_HUMAN	-0.9454651	1.1932944
sp|P37235|HPCL1_HUMAN	-0.8826199	0.656254
sp|Q9BW04|SARG_HUMAN	-0.8154545	1.1932944
sp|P62745|RHOB_HUMAN	-0.7995548	0.656254
sp|O00757|F16P2_HUMAN	-0.7635078	1.1932944
sp|Q93077|H2A1C_HUMAN	-0.7275238	1.1932944
sp|Q9UHN6-2|CEIP2_HUMAN	-0.7262268	1.1932944
sp|P22894|MMP8_HUMAN	-0.7195606	1.1932944
sp|P38159|RBMX_HUMAN	-0.7003403	0.656254
sp|Q14956-2|GPNMB_HUMAN	-0.6956101	1.1932944
sp|P33151|CADH5_HUMAN	-0.6946182	1.1932944
sp|Q13751|LAMB3_HUMAN	-0.6778717	1.2773042
sp|P28330|ACADL_HUMAN	-0.6626072	1.1932944
sp|P28676|GRAN_HUMAN	-0.6601563	1.1932944
sp|P06703|S10A6_HUMAN	-0.6211567	1.1932944
sp|Q8NC51-3|PAIRB_HUMAN	-0.6205502	0.7827403
sp|P20702|ITAX_HUMAN	-0.5988541	0.656254
sp|Q01518|CAP1_HUMAN	-0.5727549	0.656254
sp|Q15286|RAB35_HUMAN	-0.5663471	0.656254
sp|O00186|STXB3_HUMAN	-0.5506477	1.1932944

sp|P16403|H12_HUMAN	-0.5427036	0.656254
sp|Q9NVJ2|ARL8B_HUMAN	-0.5405426	1.1932944
sp|A6NMY6|AXA2L_HUMAN	-0.5333309	0.656254
sp|P16930|FAAA_HUMAN	-0.5313835	0.656254
sp|P63218|GBG5_HUMAN	-0.5306377	1.1932944
sp|Q01955|CO4A3_HUMAN	-0.5259914	1.1932944
sp|P78417-3|GSTO1_HUMAN	-0.5100975	1.1932944
sp|Q9P2J5-2|SYLC_HUMAN	-0.5088043	1.1932944
sp|Q15056-2|IF4H_HUMAN	-0.504097	1.1932944
sp|O95716|RAB3D_HUMAN	-0.5033312	0.656254
sp|P16422|EPCAM_HUMAN	-0.5014916	1.1932944
sp|O00592-2|PODXL_HUMAN	-0.4967194	1.1932944
sp|A5A3E0|POTEF_HUMAN	-0.4952145	0.656254
sp|P51636-2|CAV2_HUMAN	-0.482214	1.1932944
sp|Q9UDY2-3|ZO2_HUMAN	-0.4803429	1.1932944
sp|Q30154|DRB5_HUMAN	-0.4785252	0.656254
sp|P62070-4|RRAS2_HUMAN	-0.4752884	0.656254
sp|Q14011-2|CIRBP_HUMAN	-0.474905	1.2773042
sp|P51153|RAB13_HUMAN	-0.4657555	0.656254
sp|O76041-2|NEBL_HUMAN	-0.4624901	0.656254
sp|Q8NBQ5|DHB11_HUMAN	-0.4612865	1.1932944
sp|P61601|NCALD_HUMAN	-0.4600029	0.656254
sp|P08631-2|HCK_HUMAN	-0.4444637	0.656254
sp|Q6DN03|H2B2C_HUMAN	-0.4299011	1.1932944
sp|Q9NPY3|C1QR1_HUMAN	-0.4288006	1.1932944
sp|Q9Y376|CAB39_HUMAN	-0.4248447	1.2773042
sp|P61006|RAB8A_HUMAN	-0.4236908	1.1932944
sp|Q9Y4G6|TLN2_HUMAN	-0.4214706	0.656254
sp|P12830|CADH1_HUMAN	-0.4199619	1.1932944
sp|P12236|ADT3_HUMAN	-0.4179115	1.1932944
sp|P11717|MPRI_HUMAN	-0.4165268	1.1932944
sp|P61586|RHOA_HUMAN	-0.4154186	1.1932944
sp|O60271-4|JIP4_HUMAN	-0.4153996	0.35795313
sp|Q9H2U2-3|IPYR2_HUMAN	-0.4145241	0.656254
sp|Q9NPJ3|ACO13_HUMAN	-0.4141102	1.1932944
sp|P61224|RAP1B_HUMAN	-0.4116221	0
sp|O95837|GNA14_HUMAN	-0.407938	0.656254
sp|P15153|RAC2_HUMAN	-0.407177	0.656254
sp|P07948-2|LYN_HUMAN	-0.4029503	1.1932944
sp|P12694|ODBA_HUMAN	-0.4028282	0.7061832
sp|P06753-6|TPM3_HUMAN	-0.3999577	0.656254
sp|P29992|GNA11_HUMAN	-0.3929691	1.1932944
sp|Q13283|G3BP1_HUMAN	-0.3912773	0.6070219
sp|P48059-3|LIMS1_HUMAN	-0.3903294	0.656254
sp|P62314|SMD1_HUMAN	-0.3854885	1.1932944

sp|Q9Y3D6|FIS1_HUMAN	-0.384923	0.7061832
sp|Q96BM9|ARL8A_HUMAN	-0.3777542	0.656254
sp|P30049|ATPD_HUMAN	-0.3768444	1.1932944
sp|P21589-2|5NTD_HUMAN	-0.3725128	1.1932944
sp|Q9H8L6|MMRN2_HUMAN	-0.3720627	0.7827403
sp|P15559-2|NQO1_HUMAN	-0.3715496	1.1932944
sp|P13928|ANXA8_HUMAN	-0.3600941	0.7827403
sp|P06239-3|LCK_HUMAN	-0.356844	0.656254
sp|P62834|RAP1A_HUMAN	-0.352623	0.656254
sp|P30533|AMRP_HUMAN	-0.3519211	0.09339783
sp|P57088|TMM33_HUMAN	-0.3489761	1.1932944
sp|O14786|NRP1_HUMAN	-0.3486109	1.1932944
sp|P01112|RASH_HUMAN	-0.3461838	0.656254
sp|P36542-2|ATPG_HUMAN	-0.3439999	0.656254
sp|P08962-2|CD63_HUMAN	-0.3435535	0.7827403
sp|P01111|RASN_HUMAN	-0.3403816	0.656254
sp|P24557-2|THAS_HUMAN	-0.3358383	1.1932944
sp|Q9BZF9-2|UACA_HUMAN	-0.3349495	0.7827403
sp|Q12846|STX4_HUMAN	-0.3321304	0.656254
sp|Q9H8H3|MET7A_HUMAN	-0.3317375	1.1505735
sp|Q96S97|MYADM_HUMAN	-0.3264542	1.1932944
sp|Q9UBW8|CSN7A_HUMAN	-0.3236198	0.91601294
sp|P48509|CD151_HUMAN	-0.322464	1.1932944
sp|Q07812-7|BAX_HUMAN	-0.3190117	1.1932944
sp|Q07075|AMPE_HUMAN	-0.3174992	1.2095301
sp|P11234-2|RALB_HUMAN	-0.3141403	0.656254
sp|O95197-2|RTN3_HUMAN	-0.304512	1.1932944
sp|Q8NDH3|PEPL1_HUMAN	-0.3025169	1.0485198
sp|Q15149-9|PLEC_HUMAN	-0.3016481	0.656254
sp|P02462|CO4A1_HUMAN	-0.3006611	0.7061832
sp|Q96HD1|CREL1_HUMAN	-0.3004036	1.1932944
sp|O94811|TPPP_HUMAN	-0.2958365	0.45033538
sp|P52789|HXK2_HUMAN	-0.2953949	0.656254
sp|O43615|TIM44_HUMAN	-0.2921124	0.7827403
sp|Q14515-2|SPRL1_HUMAN	-0.2899771	1.1932944
sp|Q86W92-2|LIPB1_HUMAN	-0.2894392	1.1932944
sp|P52788-2|SPSY_HUMAN	-0.2874718	0.7827403
sp|Q5SSJ5-2|HP1B3_HUMAN	-0.2865028	0.8983557
sp|O60814|H2B1K_HUMAN	-0.2863846	0.7061832
sp|O00712-4|NFIB_HUMAN	-0.2835388	0.656254
sp|O43795-2|MYO1B_HUMAN	-0.2829037	1.1932944
sp|P23193-2|TCEA1_HUMAN	-0.2812386	0.7061832
sp|Q16822|PCKGM_HUMAN	-0.2809849	1.1932944
sp|P42566|EPS15_HUMAN	-0.2787018	1.1932944
sp|P08134|RHOC_HUMAN	-0.2785902	0.7827403

sp|Q08170|SRSF4_HUMAN	-0.2778091	0.656254
sp|Q9Y2Q5|LTOR2_HUMAN	-0.2776508	1.1932944
sp|Q9UPQ0|LIMC1_HUMAN	-0.2769775	1.120602
sp|Q9H2G2-2|SLK_HUMAN	-0.2762775	0.7827403
sp|P51606-2|RENBP_HUMAN	-0.2751961	1.1932944
sp|Q13813-2|SPTN1_HUMAN	-0.2749023	1.1932944
sp|Q9NUB1-2|ACS2L_HUMAN	-0.2740765	0.45033538
sp|Q8TCD5|NT5C_HUMAN	-0.2731609	0.7827403
sp|Q13404|UB2V1_HUMAN	-0.2727032	0.04707252
sp|Q14651|PLSI_HUMAN	-0.272152	0
sp|Q6YN16|HSDL2_HUMAN	-0.2716103	1.2773042
sp|P29972-2|AQP1_HUMAN	-0.2711754	1.1932944
sp|P11279|LAMP1_HUMAN	-0.2710266	0.45033538
sp|O94919|ENDD1_HUMAN	-0.2687855	0.7827403
sp|P63261|ACTG_HUMAN	-0.2655945	0
sp|P31946|1433B_HUMAN	-0.2634182	0.656254
sp|Q9GZP4-2|PITH1_HUMAN	-0.2613125	1.1932944
sp|Q9NUJ1-3|ABHDA_HUMAN	-0.260025	1.1932944
sp|P11387|TOP1_HUMAN	-0.259861	0.7827403
sp|Q9NVD7|PARVA_HUMAN	-0.2593308	0.19149946
sp|Q9UBR2|CATZ_HUMAN	-0.2586784	1.1932944
sp|P04839|CY24B_HUMAN	-0.2576122	0.19149946
sp|Q9UKK9|NUDT5_HUMAN	-0.2567864	0.7827403
sp|P42126-2|ECI1_HUMAN	-0.2567253	1.1932944
sp|Q15323|K1H1_HUMAN	-0.2559462	0.656254
sp|Q92930|RAB8B_HUMAN	-0.2556419	0.656254
sp|P13284|GILT_HUMAN	-0.2553797	1.2773042
sp|Q562R1|ACTBL_HUMAN	-0.2538471	0
sp|Q8NF37|PCAT1_HUMAN	-0.253109	1.120602
sp|P30048-2|PRDX3_HUMAN	-0.2496662	0.7498006
sp|Q9C0B1|FTO_HUMAN	-0.2477236	1.1932944
sp|P51553-2|IDH3G_HUMAN	-0.2452965	1.1932944
sp|Q15819|UB2V2_HUMAN	-0.2452622	0.656254
sp|P0DJI8|SAA1_HUMAN	-0.2428761	0.45033538
sp|Q6ZVM7-3|TM1L2_HUMAN	-0.2428722	0.45033538
sp|P06753-2|TPM3_HUMAN	-0.2421112	1.1932944
sp|Q7LG56-6|RIR2B_HUMAN	-0.2419567	1.1932944
sp|Q14108|SCRB2_HUMAN	-0.2416592	0.7827403
sp|P55327-3|TPD52_HUMAN	-0.2416115	1.1932944
sp|P25774|CATS_HUMAN	-0.2387981	0.5204253
sp|P02763|A1AG1_HUMAN	-0.2374077	1.2773042
sp|P36542|ATPG_HUMAN	-0.2372322	0.656254
sp|O75323|NIPS2_HUMAN	-0.2370224	1.1932944
sp|Q6NY19-2|KANK3_HUMAN	-0.2367497	0.7827403
sp|P10619-2|PPGB_HUMAN	-0.2339306	0.6070219

sp|P24666|PPAC_HUMAN	-0.2335548	0.7827403
sp|P67809|YBOX1_HUMAN	-0.2331009	0.45033538
sp|Q96EP5-2|DAZP1_HUMAN	-0.2328987	0.45033538
sp|P10412|H14_HUMAN	-0.2313881	0
sp|Q9BZE9-2|ASPC1_HUMAN	-0.2296782	0.656254
sp|P0CG39|POTEJ_HUMAN	-0.2292748	0
sp|P20339-2|RAB5A_HUMAN	-0.229003	0.656254
sp|Q53T59|H1BP3_HUMAN	-0.2289305	1.1932944
sp|Q7L5N1|CSN6_HUMAN	-0.2266197	0.19149946
sp|Q9P1F3|ABRAL_HUMAN	-0.2255697	0
sp|P47985|UCRI_HUMAN	-0.224083	0.7061832
sp|Q10567-2|AP1B1_HUMAN	-0.2234669	1.1505735
sp|P06396-2|GELS_HUMAN	-0.2233028	0.656254
sp|P07305-2|H10_HUMAN	-0.2232704	0.28516325
sp|O00231-2|PSD11_HUMAN	-0.2223816	1.2095301
sp|Q9NTK5|OLA1_HUMAN	-0.22159	0.19149946
sp|Q86TX2|ACOT1_HUMAN	-0.2185097	0.656254
sp|P30838|AL3A1_HUMAN	-0.2178421	0.45033538
sp|Q15181|IPYR_HUMAN	-0.2177277	1.0485198
sp|P84243|H33_HUMAN	-0.2175808	0
sp|P61020|RAB5B_HUMAN	-0.2166672	1.1505735
sp|P02769|ALBU_BOVIN	-0.2162476	0.02012722
sp|P23497|SP100_HUMAN	-0.2160912	1.1932944
sp|Q9ULZ3-2|ASC_HUMAN	-0.2149239	1.1932944
sp|P08648|ITA5_HUMAN	-0.2147923	1.0301651
sp|Q7L2H7|EIF3M_HUMAN	-0.2147064	0.656254
sp|P60709|ACTB_HUMAN	-0.2145882	0
sp|P30044-2|PRDX5_HUMAN	-0.2105808	0.656254
sp|P61225|RAP2B_HUMAN	-0.210247	1.1932944
sp|Q9GZT8|NIF3L_HUMAN	-0.2100315	1.1932944
sp|Q08209-5|PP2BA_HUMAN	-0.2074432	0.45033538
sp|P31937|3HIDH_HUMAN	-0.2025414	0.91601294
sp|P09493-5|TPM1_HUMAN	-0.2025185	1.1932944
sp|O95833|CLIC3_HUMAN	-0.198225	1.2773042
sp|P31939|PUR9_HUMAN	-0.1975594	1.2326616
sp|O15173-2|PGRC2_HUMAN	-0.1974373	0.6298893
sp|Q7L1Q6-2|BZW1_HUMAN	-0.1969137	0.7061832
sp|P30419-2|NMT1_HUMAN	-0.1966572	0.656254
sp|Q32MZ4-3|LRRF1_HUMAN	-0.1957684	0.45033538
sp|O60716-14|CTND1_HUMAN	-0.1956043	0.91601294
sp|P25685-2|DNJB1_HUMAN	-0.1945915	1.1932944
sp|Q9NNW7|TRXR2_HUMAN	-0.1945286	0.6298893
sp|Q53GQ0|DHB12_HUMAN	-0.1941586	0.7061832
sp|Q04837|SSBP_HUMAN	-0.1940289	0.7827403
sp|P61088|UBE2N_HUMAN	-0.1940136	0.45033538

sp|O15067|PUR4_HUMAN	-0.1932373	1.1932944
sp|Q9NYF8-2|BCLF1_HUMAN	-0.192234	1.1932944
sp|P53396-2|ACLY_HUMAN	-0.1921501	1.1025577
sp|P30084|ECHM_HUMAN	-0.1919403	1.3006523
sp|P62937|PPIA_HUMAN	-0.1890793	0.7061832
sp|P35754|GLRX1_HUMAN	-0.1874809	1.3006523
sp|P06865|HEXA_HUMAN	-0.1869659	0.80804527
sp|P61457|PHS_HUMAN	-0.186511	0.19149946
sp|Q15833-2|STXB2_HUMAN	-0.1862087	0.19149946
sp|O43175|SERA_HUMAN	-0.1860256	1.0241117
sp|P48163-2|MAOX_HUMAN	-0.185894	1.1505735
sp|O75131|CPNE3_HUMAN	-0.1854191	0.897507
sp|Q13867|BLMH_HUMAN	-0.1851311	1.1932944
sp|P26368-2|U2AF2_HUMAN	-0.1846075	1.1932944
sp|P68371|TBB4B_HUMAN	-0.1845017	0.19149946
sp|P50225|ST1A1_HUMAN	-0.1841164	1.1505735
sp|P62995-3|TRA2B_HUMAN	-0.183712	1.1932944
sp|P84095|RHOG_HUMAN	-0.1830769	0.91601294
sp|P40121|CAPG_HUMAN	-0.1824074	0.30819008
sp|P04222|1C03_HUMAN	-0.1791878	0
sp|P36551|HEM6_HUMAN	-0.1785717	0.19149946
sp|P49593-2|PPM1F_HUMAN	-0.1779547	1.0485198
sp|P07741|APT_HUMAN	-0.1757946	0.8059303
sp|Q99436|PSB7_HUMAN	-0.1757498	0.45033538
sp|Q16762|THTR_HUMAN	-0.1750088	0.21439649
sp|O15511|ARPC5_HUMAN	-0.1729717	0.8983557
sp|P61026|RAB10_HUMAN	-0.1729202	1.1932944
sp|P09917|LOX5_HUMAN	-0.172287	1.0634323
sp|P60033|CD81_HUMAN	-0.1719818	0.7827403
sp|O00391|QSOX1_HUMAN	-0.1717758	1.1932944
sp|P56134-3|ATPK_HUMAN	-0.1717033	0
sp|P12111-2|CO6A3_HUMAN	-0.1703224	1.1240381
sp|Q9UBC2-2|EP15R_HUMAN	-0.1680908	0.7827403
sp|P31946-2|1433B_HUMAN	-0.1676331	0.656254
sp|P50452|SPB8_HUMAN	-0.1668949	1.1932944
sp|P62879|GBB2_HUMAN	-0.1647854	0.7588735
sp|O75367-2|H2AY_HUMAN	-0.1626644	1.2392054
sp|Q8IV08|PLD3_HUMAN	-0.161993	0.5204253
sp|P55060-3|XPO2_HUMAN	-0.1612835	0.84879977
sp|O14773|TPP1_HUMAN	-0.160223	0.40256184
sp|Q9HB71|CYBP_HUMAN	-0.1595707	0.312067
sp|Q9Y281|COF2_HUMAN	-0.1594696	0.91601294
sp|Q16629-2|SRSF7_HUMAN	-0.1591911	0.5111962
sp|Q6NVY1|HIBCH_HUMAN	-0.1587086	1.0485198
sp|P25788-2|PSA3_HUMAN	-0.157753	0.84879977

sp|Q9H9B4|SFXN1_HUMAN	-0.1575737	0.19149946
sp|P42285|MTREX_HUMAN	-0.1561384	0.45033538
sp|P04440|DPB1_HUMAN	-0.1560478	1.1932944
sp|P12235|ADT1_HUMAN	-0.1559105	0.656254
sp|P09429|HMGB1_HUMAN	-0.1552124	0.7827403
sp|Q05315|LEG10_HUMAN	-0.1535254	0
sp|P62826|RAN_HUMAN	-0.1533928	0.87291557
sp|O75923-11|DYSF_HUMAN	-0.1524048	0
sp|P63010-2|AP2B1_HUMAN	-0.1521912	0.9533461
sp|Q6DKJ4|NXN_HUMAN	-0.1517677	0.19149946
sp|Q93084-2|AT2A3_HUMAN	-0.150959	0.45033538
sp|Q9NZ32|ARP10_HUMAN	-0.1504917	1.1932944
sp|Q5K4L6|S27A3_HUMAN	-0.1503773	0.7061832
sp|Q9BVC6|TM109_HUMAN	-0.1495514	0.7061832
sp|P51178-2|PLCD1_HUMAN	-0.1491547	0.2178309
sp|P10515|ODP2_HUMAN	-0.1487503	0.90036625
sp|Q969V3-2|NCLN_HUMAN	-0.1473389	0.7061832
sp|Q08380|LG3BP_HUMAN	-0.1471672	0.51870745
sp|P23229-4|ITA6_HUMAN	-0.146553	1.1932944
sp|Q8TD55|PKHO2_HUMAN	-0.1464691	0.45033538
sp|P62304|RUXE_HUMAN	-0.1459808	1.1932944
sp|P18084|ITB5_HUMAN	-0.1457424	1.1932944
sp|P29350-3|PTN6_HUMAN	-0.1455545	0.04454162
sp|P06132|DCUP_HUMAN	-0.1449566	0.5111962
sp|O43681|ASNA_HUMAN	-0.1446152	0.2178309
sp|P07858|CATB_HUMAN	-0.1446056	0.7827403
sp|Q13177|PAK2_HUMAN	-0.1440373	0
sp|Q06136|KDSR_HUMAN	-0.1426125	1.1932944
sp|P42704|LPPRC_HUMAN	-0.142395	0.4075265
sp|P62906|RL10A_HUMAN	-0.1414013	0.6509351
sp|Q13045-2|FLII_HUMAN	-0.1400032	0.79861414
sp|P09651-3|ROA1_HUMAN	-0.1391068	0.35795313
sp|O75347|TBCA_HUMAN	-0.1385613	0.91601294
sp|Q13884|SNTB1_HUMAN	-0.1375885	0.19149946
sp|Q5TFE4|NT5D1_HUMAN	-0.1373634	0.312067
sp|Q9NZM1-6|MYOF_HUMAN	-0.1370182	0.5630115
sp|P22314|UBA1_HUMAN	-0.1365204	0.38718706
sp|P63096|GNAI1_HUMAN	-0.1364784	0.09894868
sp|Q16836-3|HCDH_HUMAN	-0.1362305	0.91601294
sp|Q04695|K1C17_HUMAN	-0.135725	0.656254
sp|Q13418|ILK_HUMAN	-0.135437	0.0846519
sp|P53634|CATC_HUMAN	-0.13517	0.35795313
sp|Q15233|NONO_HUMAN	-0.1344814	1.1895121
sp|Q9H4A4|AMPB_HUMAN	-0.1344318	0.5474665
sp|Q15404|RSU1_HUMAN	-0.1339073	0.656254

sp|Q8NHV1|GIMA7_HUMAN	-0.13381	1.1932944
sp|P01903|DRA_HUMAN	-0.1337547	0.6298893
sp|Q9NQG5|RPR1B_HUMAN	-0.1332989	0.91601294
sp|P63000-2|RAC1_HUMAN	-0.1332588	0
sp|Q86UX7-2|URP2_HUMAN	-0.1331577	1.0044178
sp|Q8WVM8|SCFD1_HUMAN	-0.1329517	0.33495146
sp|O60701-2|UGDH_HUMAN	-0.1305618	0
sp|Q14204|DYHC1_HUMAN	-0.1292038	1.2669417
sp|Q13126-2|MTAP_HUMAN	-0.1288872	1.1240381
sp|P30046|DOPD_HUMAN	-0.1275425	0.45033538
sp|E9PAV3|NACAM_HUMAN	-0.1271095	0.7827403
sp|Q8NHP8|PLBL2_HUMAN	-0.1268616	0.19149946
sp|P27361|MK03_HUMAN	-0.1262837	1.1505735
sp|P53041|PPP5_HUMAN	-0.1259937	0.7061832
sp|P23246|SFPQ_HUMAN	-0.1248856	1.1470993
sp|Q92556|ELMO1_HUMAN	-0.1246605	0.5111962
sp|Q9BWD1|THIC_HUMAN	-0.1242142	0.19149946
sp|P30466|1B18_HUMAN	-0.1240883	0.656254
sp|Q7Z4W1|DCXR_HUMAN	-0.1237946	1.0485198
sp|Q9UQ80|PA2G4_HUMAN	-0.1231918	0
sp|P21964-2|COMT_HUMAN	-0.1218014	0.9968222
sp|P68400|CSK21_HUMAN	-0.1210709	0.5204253
sp|P21953|ODBB_HUMAN	-0.1205959	0.7061832
sp|Q06210-2|GFPT1_HUMAN	-0.1202183	0.4075265
sp|O43865|SAHH2_HUMAN	-0.1200924	0.09894868
sp|Q9BQE3|TBA1C_HUMAN	-0.1199951	0
sp|Q9BT78|CSN4_HUMAN	-0.1191788	0.5111962
sp|Q13185|CBX3_HUMAN	-0.1185494	0.8983557
sp|P05534|1A24_HUMAN	-0.1182041	0.2178309
sp|P36873-2|PP1G_HUMAN	-0.1178837	1.1932944
sp|P40306|PSB10_HUMAN	-0.1176453	1.0485198
sp|Q8N684-3|CPSF7_HUMAN	-0.1175079	0.6070219
sp|Q13976|KGP1_HUMAN	-0.1168537	0
sp|Q15393|SF3B3_HUMAN	-0.1165028	0.7217418
sp|Q99829|CPNE1_HUMAN	-0.1163769	0.6509351
sp|Q9UL18|AGO1_HUMAN	-0.1160278	0.35795313
sp|O75436|VP26A_HUMAN	-0.1160107	0.84879977
sp|P49354-2|FNTA_HUMAN	-0.1158781	1.2095301
sp|P26599-2|PTBP1_HUMAN	-0.1158352	0.8339169
sp|Q9Y262-2|EIF3L_HUMAN	-0.1155987	0.29066643
sp|Q9NQR4|NIT2_HUMAN	-0.1154747	0.65625405
sp|Q02978-2|M2OM_HUMAN	-0.1152802	0.7061832
sp|Q01813-2|PFKAP_HUMAN	-0.1151314	0.65625405
sp|P28482|MK01_HUMAN	-0.1131668	0.84879977
sp|Q92597|NDRG1_HUMAN	-0.1126461	0.7588735

sp|P46976-2|GLYG_HUMAN	-0.1122513	0.65625405
sp|Q9BTE1|DCTN5_HUMAN	-0.1121111	0.19149946
sp|P18085|ARF4_HUMAN	-0.1120873	0.656254
sp|P63220|RS21_HUMAN	-0.1118393	1.1932944
sp|P53007|TXTP_HUMAN	-0.1117382	0.90036625
sp|O75340-2|PDCD6_HUMAN	-0.111681	0.35795313
sp|P63104|1433Z_HUMAN	-0.1109772	0.2699497
sp|Q9UMS4|PRP19_HUMAN	-0.1109314	0.5832693
sp|Q13217|DNJC3_HUMAN	-0.1108322	0.6298893
sp|Q7Z7H5-3|TMED4_HUMAN	-0.1101551	1.1932944
sp|Q9BZZ5-5|API5_HUMAN	-0.1091843	0.84879977
sp|Q86U42-2|PABP2_HUMAN	-0.1082802	0
sp|Q8N392|RHG18_HUMAN	-0.1080341	0
sp|Q9Y6W5|WASF2_HUMAN	-0.1074524	0.7061832
sp|P30050|RL12_HUMAN	-0.1066628	0.5111962
sp|Q9H0W9-2|CK054_HUMAN	-0.1065826	0.52598757
sp|O94760|DDAH1_HUMAN	-0.1056919	0.6298893
sp|P02649|APOE_HUMAN	-0.1056004	0.89178735
sp|P07996|TSP1_HUMAN	-0.103817	0.5204253
sp|O94973-2|AP2A2_HUMAN	-0.1037483	0.7827403
sp|Q07955-3|SRSF1_HUMAN	-0.1036949	0.6255959
sp|P54802|ANAG_HUMAN	-0.1033001	0.45033538
sp|P61160|ARP2_HUMAN	-0.1027794	0.19149946
sp|Q8NF91-4|SYNE1_HUMAN	-0.101943	0.19149946
sp|P51858|HDGF_HUMAN	-0.0995159	0.30372584
sp|P67775|PP2AA_HUMAN	-0.0993099	0
sp|Q86VS8|HOOK3_HUMAN	-0.0991364	0.19149946
sp|O75937|DNJC8_HUMAN	-0.0978508	0.7588735
sp|P45954-2|ACDSB_HUMAN	-0.0972939	0
sp|Q9Y371-2|SHLB1_HUMAN	-0.0972099	0
sp|P54578-3|UBP14_HUMAN	-0.0971432	0.1342476
sp|O00429-6|DNM1L_HUMAN	-0.0971146	0.26546443
sp|Q9Y3B3|TMED7_HUMAN	-0.0968628	0.19149946
sp|P36776-3|LONM_HUMAN	-0.0967646	0.95332193
sp|P52272-2|HNRPM_HUMAN	-0.0967445	0.8469722
sp|Q16647|PTGIS_HUMAN	-0.0965643	0.6115017
sp|Q92598-2|HS105_HUMAN	-0.0963955	0.656254
sp|Q12905|ILF2_HUMAN	-0.0963593	0.5004855
sp|P48449-3|ERG7_HUMAN	-0.0958519	0.5111962
sp|P02768|ALBU_HUMAN	-0.0957909	1.0092533
sp|Q9UJS0-2|CMC2_HUMAN	-0.095356	1.1932944
sp|P25787|PSA2_HUMAN	-0.0952215	0.9968222
sp|P06748-2|NPM_HUMAN	-0.0947742	0.2178309
sp|O75947-2|ATP5H_HUMAN	-0.0938892	0.9533461
sp|P48637|GSHB_HUMAN	-0.0937634	0.6827362

sp|P55884-2|EIF3B_HUMAN	-0.0933991	0.23541966
sp|P54136|SYRC_HUMAN	-0.0933571	0.09339783
sp|Q13435|SF3B2_HUMAN	-0.0932007	0.312067
sp|P00325|ADH1B_HUMAN	-0.0931187	0.65625405
sp|P26038|MOES_HUMAN	-0.092556	0.68123025
sp|P12081-4|SYHC_HUMAN	-0.092413	0
sp|P61313|RL15_HUMAN	-0.0923767	0
sp|P26885|FKBP2_HUMAN	-0.0923615	0.45033538
sp|P00568|KAD1_HUMAN	-0.091547	0.852217
sp|Q96TC7|RMD3_HUMAN	-0.0910797	0.91601294
sp|P52758|RIDA_HUMAN	-0.0909653	0.7827403
sp|Q99714|HCD2_HUMAN	-0.0908012	1.2432404
sp|P23142-4|FBLN1_HUMAN	-0.0907688	0
sp|P30085|KCY_HUMAN	-0.090682	0.7061832
sp|Q15365|PCBP1_HUMAN	-0.0898323	0.7217418
sp|P58107|EPIPL_HUMAN	-0.0897989	0.46530083
sp|P32455|GBP1_HUMAN	-0.0894337	1.120602
sp|O43747-2|AP1G1_HUMAN	-0.0893326	0.5111962
sp|Q16775-2|GLO2_HUMAN	-0.0887814	0.2178309
sp|P20618|PSB1_HUMAN	-0.0885849	1.2805575
sp|P30566|PUR8_HUMAN	-0.0882378	0.656254
sp|Q96KP4|CNDP2_HUMAN	-0.0875359	1.0584712
sp|P02792|FRIL_HUMAN	-0.0871429	0.20467198
sp|P11142|HSP7C_HUMAN	-0.08708	0.66672885
sp|P10606|COX5B_HUMAN	-0.0865002	0.7061832
sp|Q13813-3|SPTN1_HUMAN	-0.0862503	0.19149946
sp|P68104|EF1A1_HUMAN	-0.0862312	0.57986265
sp|P07358|CO8B_HUMAN	-0.0857563	0.06291623
sp|O43684-2|BUB3_HUMAN	-0.0857544	0.19149946
sp|Q14103-3|HNRPD_HUMAN	-0.0856972	0.54838234
sp|Q66K74-2|MAP1S_HUMAN	-0.0853272	0
sp|O15260-2|SURF4_HUMAN	-0.0852308	0.45033538
sp|P61981|1433G_HUMAN	-0.0849304	1.0009397
sp|P29400-2|CO4A5_HUMAN	-0.0849152	0
sp|O75915|PRAF3_HUMAN	-0.0843878	0.91601294
sp|O43776|SYNC_HUMAN	-0.0840988	0.05634482
sp|P49588-2|SYAC_HUMAN	-0.0838833	0.4726498
sp|P08237-3|PFKAM_HUMAN	-0.0838623	0
sp|Q9Y2B0|CNPY2_HUMAN	-0.0835648	0
sp|Q92973-2|TNPO1_HUMAN	-0.0831871	0.30372584
sp|Q06033-2|ITIH3_HUMAN	-0.0826988	0.19149946
sp|P13797|PLST_HUMAN	-0.0821762	0.32367095
sp|P55010|IF5_HUMAN	-0.0821171	0.7588735
sp|P07384|CAN1_HUMAN	-0.0821114	0.37744418
sp|P37802|TAGL2_HUMAN	-0.0820923	0.45546502

sp|P41091|IF2G_HUMAN	-0.081768	0.33495146
sp|Q9HAV0|GBB4_HUMAN	-0.0814991	0
sp|O75369-2|FLNB_HUMAN	-0.0812931	1.0147437
sp|P54577|SYYC_HUMAN	-0.0811462	0.61034113
sp|Q07507|DERM_HUMAN	-0.0810204	1.2805575
sp|Q02543|RL18A_HUMAN	-0.0808353	1.214352
sp|Q14974|IMB1_HUMAN	-0.0807819	0.85449874
sp|P17612|KAPCA_HUMAN	-0.080492	0.6298893
sp|O14787-2|TNPO2_HUMAN	-0.0799246	0
sp|P46108|CRK_HUMAN	-0.0790901	0.7957244
sp|P38919|IF4A3_HUMAN	-0.0790844	0.68888646
sp|P09211|GSTP1_HUMAN	-0.0789566	0.08684197
sp|P62263|RS14_HUMAN	-0.0783138	0.2178309
sp|Q9NSE4|SYIM_HUMAN	-0.0778847	0.06842031
sp|Q13938-4|CAYP1_HUMAN	-0.0778103	0
sp|P49903-2|SPS1_HUMAN	-0.0775528	0.35795313
sp|Q00610-2|CLH1_HUMAN	-0.07728	0.26865074
sp|Q92696|PGTA_HUMAN	-0.0762901	0
sp|P40227|TCPZ_HUMAN	-0.0762482	0.25761655
sp|Q8TAT6-2|NPL4_HUMAN	-0.0760307	0.30372584
sp|P52565|GDIR1_HUMAN	-0.0752792	0.5283125
sp|Q9Y305-4|ACOT9_HUMAN	-0.0752068	0
sp|P61923|COPZ1_HUMAN	-0.0748415	0
sp|P29218|IMPA1_HUMAN	-0.0747204	0.6063746
sp|Q96AG4|LRC59_HUMAN	-0.0743656	1.1791906
sp|P06756-3|ITAV_HUMAN	-0.0741406	0.65625405
sp|P62888|RL30_HUMAN	-0.0741043	0.91601294
sp|Q02952-2|AKA12_HUMAN	-0.0738525	0.19149946
sp|P11586|C1TC_HUMAN	-0.0737228	0.2178309
sp|Q14165|MLEC_HUMAN	-0.0730686	0.7061832
sp|P35998|PRS7_HUMAN	-0.0730228	1.0009397
sp|O75964|ATP5L_HUMAN	-0.0729523	0.45033538
sp|Q9NSD9|SYFB_HUMAN	-0.0727997	0.1342476
sp|Q02790|FKBP4_HUMAN	-0.072773	1.2095301
sp|P00492|HPRT_HUMAN	-0.0726166	0.1342476
sp|O94788-3|AL1A2_HUMAN	-0.0725908	0.656254
sp|Q96FN4|CPNE2_HUMAN	-0.0723763	0
sp|P28065-2|PSB9_HUMAN	-0.0722313	0.4075265
sp|O15127|SCAM2_HUMAN	-0.0722046	0.7061832
sp|Q96QK1|VPS35_HUMAN	-0.0719128	0.48353976
sp|O94905|ERLN2_HUMAN	-0.0717545	1.1932944
sp|O95336|6PGL_HUMAN	-0.0715733	0.4075265
sp|Q9NUQ9|FA49B_HUMAN	-0.0709705	0.2178309
sp|Q9Y5K5-2|UCHL5_HUMAN	-0.070961	0.43633315
sp|Q32P44|EMAL3_HUMAN	-0.0707836	0.43633315

sp|P31948|STIP1_HUMAN	-0.0707626	0
sp|O00299|CLIC1_HUMAN	-0.0706558	0.3029608
sp|Q13425|SNTB2_HUMAN	-0.0705185	0.09750395
sp|Q3LXA3|TKFC_HUMAN	-0.0703239	0.24295025
sp|O95340-2|PAPS2_HUMAN	-0.0702934	0.35193655
sp|Q5JPE7-2|NOMO2_HUMAN	-0.0701389	0.2178309
sp|Q8NCW5|NNRE_HUMAN	-0.0687141	0
sp|O00743-3|PPP6_HUMAN	-0.0686302	0.35795313
sp|P49720|PSB3_HUMAN	-0.0684109	0.56808305
sp|O94804|STK10_HUMAN	-0.0682182	0.19149946
sp|P39060-1|COIA1_HUMAN	-0.0680008	0.44572112
sp|P25786-2|PSA1_HUMAN	-0.067955	1.2474942
sp|P35579-2|MYH9_HUMAN	-0.0677929	0.656254
sp|P22234-2|PUR6_HUMAN	-0.0671654	0.37060758
sp|P46777|RL5_HUMAN	-0.0670261	0.08307569
sp|Q9UHX1-6|PUF60_HUMAN	-0.0664654	0
sp|P16219|ACADS_HUMAN	-0.0664101	0.14672586
sp|Q15631|TSN_HUMAN	-0.0659523	0.06291623
sp|P07203|GPX1_HUMAN	-0.0652466	0.43103603
sp|O60749|SNX2_HUMAN	-0.0649891	0.76289684
sp|P39019|RS19_HUMAN	-0.0649452	0.2178309
sp|P55786|PSA_HUMAN	-0.0647259	0.3840488
sp|P15170-2|ERF3A_HUMAN	-0.064373	0
sp|P14618|KPYM_HUMAN	-0.0642414	0
sp|P29466-2|CASP1_HUMAN	-0.0640602	0
sp|P54727|RD23B_HUMAN	-0.0638886	0.5204253
sp|Q10713|MPPA_HUMAN	-0.063549	0.33495146
sp|Q96MM6|HS12B_HUMAN	-0.0634537	0.06986027
sp|Q13561-2|DCTN2_HUMAN	-0.0630188	1.1752976
sp|P26641|EF1G_HUMAN	-0.0625916	0.4808736
sp|P08236-2|BGLR_HUMAN	-0.0624828	0.30372584
sp|Q8WXF1|PSPC1_HUMAN	-0.0622787	0.33495146
sp|Q5EBM0|CMPK2_HUMAN	-0.0622721	0.09894868
sp|Q9NQW7-3|XPP1_HUMAN	-0.0622444	0.08891098
sp|P28066|PSA5_HUMAN	-0.0621796	0.7498006
sp|Q9P0L0-2|VAPA_HUMAN	-0.061882	0.45720983
sp|Q96JB5-4|CK5P3_HUMAN	-0.061821	0.09894868
sp|P24539|AT5F1_HUMAN	-0.0615196	0
sp|Q16539-2|MK14_HUMAN	-0.0615025	0.19149946
sp|P15144|AMPN_HUMAN	-0.0609169	0.316588
sp|Q9Y6A4|CFA20_HUMAN	-0.0607166	0.19149946
sp|P28074|PSB5_HUMAN	-0.0607147	0
sp|P35268|RL22_HUMAN	-0.0606346	0.35795313
sp|Q16401-2|PSMD5_HUMAN	-0.0606003	0.48520416
sp|P60983|GMFB_HUMAN	-0.0602951	0.35795313

sp|P15311|EZRI_HUMAN	-0.0599489	0
sp|P49189|AL9A1_HUMAN	-0.0597305	0.3037259
sp|P35222|CTNB1_HUMAN	-0.0596619	0.386649
sp|P11498|PYC_HUMAN	-0.059413	0
sp|Q9HBL0|TENS1_HUMAN	-0.0586567	0.02388411
sp|P63244|RACK1_HUMAN	-0.0579853	0.5998898
sp|P04424-2|ARLY_HUMAN	-0.057663	0.57986265
sp|Q04323-2|UBXN1_HUMAN	-0.0575333	0
sp|P46940|IQGA1_HUMAN	-0.0572071	0.33057013
sp|P29590|PML_HUMAN	-0.0566711	0.20549615
sp|P25325-2|THTM_HUMAN	-0.0566387	0.06291623
sp|P28070|PSB4_HUMAN	-0.0562515	0.316588
sp|P01920|DQB1_HUMAN	-0.0561085	0.19149946
sp|P09417-2|DHPR_HUMAN	-0.055727	0
sp|P19367-2|HXK1_HUMAN	-0.0553093	0.2909411
sp|O43837|IDH3B_HUMAN	-0.0552444	0.2178309
sp|O95571|ETHE1_HUMAN	-0.0550346	0.95332193
sp|O60256-3|KPRB_HUMAN	-0.0549641	0.45033538
sp|Q969X5-2|ERGI1_HUMAN	-0.0547962	0.09894868
sp|P16157-21|ANK1_HUMAN	-0.0545044	0.37060758
sp|O75643|U520_HUMAN	-0.0543633	0.28516325
sp|P27487|DPP4_HUMAN	-0.0543098	0.30372584
sp|P46926|GNPI1_HUMAN	-0.0538464	0.21439649
sp|P52566|GDIR2_HUMAN	-0.0535164	0.6509351
sp|Q96CN7|ISOC1_HUMAN	-0.0535011	0.20467198
sp|P51888|PRELP_HUMAN	-0.0533276	0.8826702
sp|P22897|MRC1_HUMAN	-0.0531921	1.1600966
sp|Q9P2R7-2|SUCB1_HUMAN	-0.0529766	0.1500082
sp|P61201-2|CSN2_HUMAN	-0.0520706	0.19149946
sp|Q9Y3A5|SBDS_HUMAN	-0.0518684	0.02174174
sp|Q9BS26|ERP44_HUMAN	-0.0517159	0.6115017
sp|P15374|UCHL3_HUMAN	-0.0509949	0.17319627
sp|P55263|ADK_HUMAN	-0.0509758	0.1342476
sp|P00387-3|NB5R3_HUMAN	-0.0507183	0.95332193
sp|P31942-2|HNRH3_HUMAN	-0.0503616	0.56808305
sp|P10155|RO60_HUMAN	-0.0500374	0.04454162
sp|P61081|UBC12_HUMAN	-0.049511	0.11949348
sp|Q96C23|GALM_HUMAN	-0.0493193	0
sp|Q15029-2|U5S1_HUMAN	-0.0492687	0.6828178
sp|P17980|PRS6A_HUMAN	-0.0491009	0.2838047
sp|Q96FV2-2|SCRN2_HUMAN	-0.0487289	0.45033538
sp|O15498-2|YKT6_HUMAN	-0.0486279	0
sp|P47755|CAZA2_HUMAN	-0.0481968	0.56639653
sp|P00736|C1R_HUMAN	-0.0480814	0.35795313
sp|P55209-2|NP1L1_HUMAN	-0.047823	0

sp|Q03154-4|ACY1_HUMAN	-0.0477085	0.2178309
sp|Q9NP79|VTA1_HUMAN	-0.0463638	0.09894868
sp|P60866-2|RS20_HUMAN	-0.0463409	0
sp|P50914|RL14_HUMAN	-0.0460129	0.35795313
sp|B5ME19|EIFCL_HUMAN	-0.045557	0.35795313
sp|P11166|GTR1_HUMAN	-0.0453548	0
sp|P49411|EFTU_HUMAN	-0.0446491	0.22817776
sp|O95865|DDAH2_HUMAN	-0.0446434	0.1318641
sp|Q9Y2X3|NOP58_HUMAN	-0.044548	0.33495146
sp|Q15366-2|PCBP2_HUMAN	-0.0437698	0.2178309
sp|Q969H8|MYDGF_HUMAN	-0.0436459	0.35795313
sp|P0C0S5|H2AZ_HUMAN	-0.0434856	0
sp|P37108|SRP14_HUMAN	-0.0432529	0.19149946
sp|O43491|E41L2_HUMAN	-0.0428371	0.09851671
sp|P09619|PGFRB_HUMAN	-0.0428314	0
sp|P62136|PP1A_HUMAN	-0.0426178	0.35795313
sp|Q92888-2|ARHG1_HUMAN	-0.0425758	0
sp|Q12907|LMAN2_HUMAN	-0.0424614	0.7957244
sp|P13861|KAP2_HUMAN	-0.0424004	0.1017544
sp|Q07020-2|RL18_HUMAN	-0.0423145	0.7061832
sp|P33316|DUT_HUMAN	-0.0413361	0.19149946
sp|O95782-2|AP2A1_HUMAN	-0.0412827	0.33495146
sp|Q13409-3|DC1I2_HUMAN	-0.0410566	0.03367973
sp|O60784-3|TOM1_HUMAN	-0.0409584	0
sp|Q9Y3I0|RTCB_HUMAN	-0.040062	0.29934928
sp|Q8N163-2|CCAR2_HUMAN	-0.0399914	0
sp|P40123-2|CAP2_HUMAN	-0.0399532	0
sp|O43390-2|HNRPR_HUMAN	-0.0399284	0.24660711
sp|Q8N1G4|LRC47_HUMAN	-0.039463	0.11612091
sp|P53597|SUCA_HUMAN	-0.0393639	0
sp|Q96HN2-2|SAHH3_HUMAN	-0.0368423	0
sp|Q9UNM6-2|PSD13_HUMAN	-0.0367928	0
sp|Q9UNF0-2|PACN2_HUMAN	-0.0367088	0.20467198
sp|Q9UN86-2|G3BP2_HUMAN	-0.036602	0
sp|Q16134-3|ETFD_HUMAN	-0.0360365	0.19149946
sp|P07900-2|HS90A_HUMAN	-0.0359478	0.24801621
sp|Q9BUF5|TBB6_HUMAN	-0.035635	0.30372584
sp|P22626|ROA2_HUMAN	-0.0354233	0.90892553
sp|Q99598|TSNAX_HUMAN	-0.0353832	0
sp|P50502|F10A1_HUMAN	-0.0351906	0.04454162
sp|Q13526|PIN1_HUMAN	-0.0345364	0
sp|P27797|CALR_HUMAN	-0.0342979	0.45915356
sp|Q8NBJ5|GT251_HUMAN	-0.0339432	0.09894868
sp|Q8TCJ2|STT3B_HUMAN	-0.0337677	0.35795313
sp|P25705|ATPA_HUMAN	-0.0335064	0.09750395

sp|Q08211|DHX9_HUMAN	-0.0329437	0.29317275
sp|P31153|METK2_HUMAN	-0.0327816	0
sp|Q92499|DDX1_HUMAN	-0.0323505	0.3852666
sp|P08238|HS90B_HUMAN	-0.0322266	0.5360584
sp|P22061-2|PIMT_HUMAN	-0.0320377	0.30372584
sp|Q9H6S3|ES8L2_HUMAN	-0.0319052	0.19149946
sp|Q15717-2|ELAV1_HUMAN	-0.031353	0.09894868
sp|P08559-2|ODPA_HUMAN	-0.0301933	0.07727676
sp|Q7Z4I7-3|LIMS2_HUMAN	-0.0299683	0.19149946
sp|Q92522|H1X_HUMAN	-0.029871	0.19149946
sp|Q9NY15|STAB1_HUMAN	-0.0298462	0
sp|P05455|LA_HUMAN	-0.0297737	0.44572112
sp|Q9Y6N5|SQOR_HUMAN	-0.0296116	0.03435687
sp|Q12931-2|TRAP1_HUMAN	-0.0294609	0.35795313
sp|P43034|LIS1_HUMAN	-0.0293274	0.03367973
sp|P02794|FRIH_HUMAN	-0.0293007	0
sp|Q6IAA8|LTOR1_HUMAN	-0.0290546	0.19149946
sp|P23588|IF4B_HUMAN	-0.0289764	0.1342476
sp|Q9BS40|LXN_HUMAN	-0.028944	0.06291623
sp|Q16543|CDC37_HUMAN	-0.0285397	0.4918224
sp|O75489|NDUS3_HUMAN	-0.0278931	0.1342476
sp|P00338|LDHA_HUMAN	-0.0277729	0.03956581
sp|O43790|KRT86_HUMAN	-0.0277615	0
sp|Q9NZK5|ADA2_HUMAN	-0.0271149	0.45033538
sp|Q16774|KGUA_HUMAN	-0.02672	0
sp|P11216|PYGB_HUMAN	-0.0262146	0.06468696
sp|Q9UKV3-5|ACINU_HUMAN	-0.0262051	0.21439649
sp|P16298-4|PP2BB_HUMAN	-0.0257359	0
sp|Q9UH99-3|SUN2_HUMAN	-0.025692	0.21439649
sp|P98160|PGBM_HUMAN	-0.0245514	0.2929586
sp|P27694|RFA1_HUMAN	-0.0244694	0.20467198
sp|P39656-3|OST48_HUMAN	-0.0242844	0.53506804
sp|P50990|TCPQ_HUMAN	-0.0236511	0.1024875
sp|P28072|PSB6_HUMAN	-0.0234489	0.09894868
sp|P0DPI2-2|GAL3A_HUMAN	-0.0229511	0.09894868
sp|Q15102|PA1B3_HUMAN	-0.0229149	0.2178309
sp|P10599-2|THIO_HUMAN	-0.022913	0.21439649
sp|Q969G5|CAVN3_HUMAN	-0.0226154	0.2178309
sp|P12277|KCRB_HUMAN	-0.0222225	0.02347357
sp|O14617-4|AP3D1_HUMAN	-0.0219345	0
sp|P30041|PRDX6_HUMAN	-0.0219021	0.308482
sp|P17987|TCPA_HUMAN	-0.0218391	0.16800755
sp|P12955|PEPD_HUMAN	-0.0215054	0.10861364
sp|O75368|SH3L1_HUMAN	-0.0211754	0.09894868
sp|O43143|DHX15_HUMAN	-0.0211449	0

sp|P49591|SYSC_HUMAN	-0.0209522	0.05681695
sp|Q9NZL9|MAT2B_HUMAN	-0.020813	0
sp|P51665|PSMD7_HUMAN	-0.0204697	0.19149946
sp|P08865|RSSA_HUMAN	-0.0196114	0.01819076
sp|P78417-2|GSTO1_HUMAN	-0.0192776	0.656254
sp|Q96CW1-2|AP2M1_HUMAN	-0.018877	0.40256184
sp|Q16891-2|MIC60_HUMAN	-0.0187168	0.16702904
sp|Q86UP2-4|KTN1_HUMAN	-0.0185623	0.08255737
sp|P16070-7|CD44_HUMAN	-0.0181046	0
sp|P00367|DHE3_HUMAN	-0.0179462	0.29301754
sp|P55265-4|DSRAD_HUMAN	-0.0177841	0.04707252
sp|P12270|TPR_HUMAN	-0.0175743	0.09621262
sp|P20674|COX5A_HUMAN	-0.0171833	0.2178309
sp|P05387|RLA2_HUMAN	-0.0164661	0.19149946
sp|P42167|LAP2B_HUMAN	-0.0163918	0
sp|P47756-2|CAPZB_HUMAN	-0.0161057	0.15517515
sp|P26196|DDX6_HUMAN	-0.0160904	0
sp|Q6XQN6-2|PNCB_HUMAN	-0.0159493	0.10138063
sp|P53004|BIEA_HUMAN	-0.0152626	0.20467198
sp|P11217|PYGM_HUMAN	-0.0143528	0
sp|P02511|CRYAB_HUMAN	-0.0143185	0.1558116
sp|P52597|HNRPF_HUMAN	-0.0142612	0.10861364
sp|P01019|ANGT_HUMAN	-0.0141678	0.1342476
sp|P51692|STA5B_HUMAN	-0.0137444	0.45033538
sp|P34897-2|GLYM_HUMAN	-0.0132236	0.1500082
sp|P45974-2|UBP5_HUMAN	-0.0130653	0.3761346
sp|P56537|IF6_HUMAN	-0.0130501	0.09339783
sp|Q92905|CSN5_HUMAN	-0.0126476	0
sp|P00966|ASSY_HUMAN	-0.0124435	0.10861364
sp|Q5R3I4|TTC38_HUMAN	-0.0120115	0.19149946
sp|P09104-2|ENOG_HUMAN	-0.0117722	0.26546443
sp|O14579|COPE_HUMAN	-0.0116577	0.33495146
sp|Q9BTV4|TMM43_HUMAN	-0.0114441	0
sp|Q6UVK1|CSPG4_HUMAN	-0.0104103	0.4075265
sp|P62244|RS15A_HUMAN	-0.0104065	0
sp|P48047|ATPO_HUMAN	-0.0102081	0.21989624
sp|P01911|2B1F_HUMAN	-0.0097561	0.19149946
sp|O75396|SC22B_HUMAN	-0.0096741	0.03367973
sp|Q86WV6|STING_HUMAN	-0.0094528	0
sp|P05166-2|PCCB_HUMAN	-0.0090599	0.26737198
sp|A0FGR8-2|ESYT2_HUMAN	-0.0085754	0
sp|O94979-10|SC31A_HUMAN	-0.0083485	0.09894868
sp|O15145|ARPC3_HUMAN	-0.0082932	0
sp|Q14498-2|RBM39_HUMAN	-0.0078087	0.14672586
sp|P16144-2|ITB4_HUMAN	-0.0076666	0.19149946

sp|O00487|PSDE_HUMAN	-0.0074711	0.45033538
sp|Q9HC35-2|EMAL4_HUMAN	-0.0072689	0.48520416
sp|Q14203-3|DCTN1_HUMAN	-0.0062733	0.06986027
sp|P31930|QCR1_HUMAN	-0.0055485	0.26295313
sp|P22695|QCR2_HUMAN	-0.0054111	0
sp|P61978-3|HNRPK_HUMAN	-0.0051365	0.10537347
sp|P14866-2|HNRPL_HUMAN	-0.0050602	0
sp|Q7KZF4|SND1_HUMAN	-0.0050354	0.00900315
sp|P20591|MX1_HUMAN	-0.0048466	0.4075265
sp|P02730|B3AT_HUMAN	-0.0045662	0.13745423
sp|Q13232|NDK3_HUMAN	-0.0042744	0.1342476
sp|O00170|AIP_HUMAN	-0.0042171	0
sp|Q9Y4L1|HYOU1_HUMAN	-0.003315	0.14223771
sp|P14923|PLAK_HUMAN	-0.003252	0.06291623
sp|Q15185-3|TEBP_HUMAN	-0.0031204	0
sp|Q9Y3Z3|SAMH1_HUMAN	-0.0028877	0.02127174
sp|P46459|NSF_HUMAN	-0.0026493	0.06291623
sp|P08294|SODE_HUMAN	-0.0025978	0.03367973
sp|P01743|HV146_HUMAN	-0.0020771	0
sp|O14828-2|SCAM3_HUMAN	-0.0020084	0.35795313
sp|Q12882|DPYD_HUMAN	-0.0017223	0
sp|P62942|FKB1A_HUMAN	-0.0015831	0
sp|P26640|SYVC_HUMAN	-0.0014801	0.2591514
sp|Q9UI12-2|VATH_HUMAN	-7.63E-04	0.04454162
sp|P54652|HSP72_HUMAN	2.63E-04	0.09894868
sp|O75608-2|LYPA1_HUMAN	0.00107765	0
sp|Q08378|GOGA3_HUMAN	0.00115013	0.35795313
sp|Q13557-12|KCC2D_HUMAN	0.00175285	0.06986027
sp|Q96CX2|KCD12_HUMAN	0.0019722	0.1742838
sp|Q9NPH2|INO1_HUMAN	0.00252533	0.1500082
sp|P63167|DYL1_HUMAN	0.00264549	0
sp|Q13363-2|CTBP1_HUMAN	0.00268364	0.19149946
sp|P23528|COF1_HUMAN	0.00302506	0.20174193
sp|P68366-2|TBA4A_HUMAN	0.00310135	0
sp|Q00577|PURA_HUMAN	0.00311279	0.04454162
sp|O43242|PSMD3_HUMAN	0.00356293	0.01073991
sp|P10768|ESTD_HUMAN	0.0038929	0.20467198
sp|Q9Y3A3-3|PHOCN_HUMAN	0.00438404	0.19149946
sp|P07237|PDIA1_HUMAN	0.00553513	0.30662137
sp|P27695|APEX1_HUMAN	0.00560188	0
sp|P49721|PSB2_HUMAN	0.00595284	0.19149946
sp|Q8NBF2-2|NHLC2_HUMAN	0.00613403	0
sp|P18077|RL35A_HUMAN	0.00629234	0
sp|Q15008-4|PSMD6_HUMAN	0.00661659	0.19149946
sp|O14745|NHRF1_HUMAN	0.00668526	0.2178309

sp|P18124|RL7_HUMAN	0.00697708	0.26737198
sp|P07737|PROF1_HUMAN	0.00714874	0.09157685
sp|P22059|OSBP1_HUMAN	0.00797653	0
sp|Q96DG6|CMBL_HUMAN	0.0081234	0
sp|Q96IJ6-2|GMPPA_HUMAN	0.00816631	0
sp|P21810|PGS1_HUMAN	0.00882721	0.11768232
sp|Q13243-3|SRSF5_HUMAN	0.0090065	0
sp|P20774|MIME_HUMAN	0.00906277	0.04454162
sp|P46063|RECQ1_HUMAN	0.00930786	0
sp|P15121|ALDR_HUMAN	0.00967026	0.01819076
sp|P60174|TPIS_HUMAN	0.00986862	0.02228302
sp|P40429|RL13A_HUMAN	0.0102787	0.09894868
sp|Q86UX2-2|ITIH5_HUMAN	0.01057243	0.312067
sp|P12110|CO6A2_HUMAN	0.0106411	0.06269496
sp|P33176|KINH_HUMAN	0.01134872	0.46889037
sp|P42224-2|STAT1_HUMAN	0.01155472	0.20467198
sp|Q13423|NNTM_HUMAN	0.01271248	0.17511293
sp|O43707|ACTN4_HUMAN	0.01282692	0.6205528
sp|Q6WCQ1-2|MPRIP_HUMAN	0.01292133	0.1063047
sp|O43301|HS12A_HUMAN	0.01348686	0.1342476
sp|Q9UNZ2-5|NSF1C_HUMAN	0.01371574	0.14927356
sp|P49368|TCPG_HUMAN	0.01391411	0.38040894
sp|Q13464|ROCK1_HUMAN	0.01453018	0
sp|P30153|2AAA_HUMAN	0.01506043	0.21116015
sp|Q9UBQ5|EIF3K_HUMAN	0.01510239	0.19149946
sp|P50991-2|TCPD_HUMAN	0.01566124	0.01551361
sp|Q99832|TCPH_HUMAN	0.01668739	0.44843918
sp|Q02878|RL6_HUMAN	0.01670647	0.35795313
sp|P00491|PNPH_HUMAN	0.01716042	0.21680334
sp|P62424|RL7A_HUMAN	0.01829147	0.09339783
sp|O75112-7|LDB3_HUMAN	0.01877022	0.35795313
sp|P28062|PSB8_HUMAN	0.01880646	0.5111962
sp|Q14240-2|IF4A2_HUMAN	0.01911259	0.06291623
sp|O43252|PAPS1_HUMAN	0.01913834	0
sp|Q9H299|SH3L3_HUMAN	0.01919746	0
sp|P78371|TCPB_HUMAN	0.0204277	0.2552695
sp|P48681|NEST_HUMAN	0.02079201	0.1558116
sp|P42785-2|PCP_HUMAN	0.02090263	0.09894868
sp|Q9Y3F4-2|STRAP_HUMAN	0.02092552	0.35795313
sp|P15529-10|MCP_HUMAN	0.02094841	0
sp|O15143|ARC1B_HUMAN	0.02102661	0.02174174
sp|Q92841-3|DDX17_HUMAN	0.0211544	0.13150784
sp|P00558|PGK1_HUMAN	0.02119446	0.01406953
sp|Q92688-2|AN32B_HUMAN	0.02120018	0.35795313
sp|O14950|ML12B_HUMAN	0.02140617	0.40256184

sp|Q9P0V9-2|SEP10_HUMAN	0.02160072	0.17511293
sp|P12004|PCNA_HUMAN	0.02183533	0
sp|O15296|LX15B_HUMAN	0.02200699	0.25789237
sp|Q9NY33|DPP3_HUMAN	0.02285767	0.16077396
sp|Q9UK22|FBX2_HUMAN	0.02301788	0.19149946
sp|P62333|PRS10_HUMAN	0.02305222	0.11949348
sp|Q96C19|EFHD2_HUMAN	0.02346993	0
sp|Q9UEY8|ADDG_HUMAN	0.02376938	0.05634482
sp|P04217|A1BG_HUMAN	0.02401733	0.31741163
sp|P14868|SYDC_HUMAN	0.02432442	0.51669085
sp|Q14764|MVP_HUMAN	0.02462769	0.3325435
sp|P09874|PARP1_HUMAN	0.0247364	0.19410844
sp|P62701|RS4X_HUMAN	0.02474976	0.06291623
sp|P51649-2|SSDH_HUMAN	0.0255661	0.06291623
sp|O95466-2|FMNL1_HUMAN	0.02561474	0
sp|P35611-2|ADDA_HUMAN	0.02566147	0.02174174
sp|Q15121|PEA15_HUMAN	0.02616692	0
sp|Q15084-2|PDIA6_HUMAN	0.02658844	0.1558116
sp|Q9BRR6-2|ADPGK_HUMAN	0.0266304	0.19149946
sp|Q9UHL4|DPP2_HUMAN	0.02669716	0.3852666
sp|P23142|FBLN1_HUMAN	0.02698326	0.06986027
sp|Q9Y394-2|DHRS7_HUMAN	0.02736664	0.06291623
sp|Q13492-2|PICAL_HUMAN	0.02808952	0
sp|P16152|CBR1_HUMAN	0.02826691	0.55270666
sp|O76003|GLRX3_HUMAN	0.02935982	0
sp|Q96BW5-2|PTER_HUMAN	0.03058243	0.19149946
sp|P50453|SPB9_HUMAN	0.03091621	0.3201336
sp|O76074-2|PDE5A_HUMAN	0.03158474	0.09894868
sp|Q16531|DDB1_HUMAN	0.03188133	0.30084842
sp|Q96P70|IPO9_HUMAN	0.03191757	0.1342476
sp|P14174|MIF_HUMAN	0.03211212	0
sp|P22033|MUTA_HUMAN	0.03272247	0.35795313
sp|P06744|G6PI_HUMAN	0.03273201	0.26694477
sp|P28838-2|AMPL_HUMAN	0.03281403	0.6077942
sp|Q1KMD3|HNRL2_HUMAN	0.0333004	0.3661873
sp|Q15691|MARE1_HUMAN	0.03347015	0.02174174
sp|Q5TZA2|CROCC_HUMAN	0.03391647	0.1342476
sp|P30043|BLVRB_HUMAN	0.0340004	0.30372584
sp|O14744|ANM5_HUMAN	0.03488541	0
sp|P05388|RLA0_HUMAN	0.03495026	0.2178309
sp|O60313-10|OPA1_HUMAN	0.03549194	0.19149946
sp|P61758|PFD3_HUMAN	0.0359726	0.19149946
sp|P22392-2|NDKB_HUMAN	0.03624344	0.48520416
sp|Q92769|HDAC2_HUMAN	0.03636742	0
sp|P58546|MTPN_HUMAN	0.03732109	0.5204253

sp|Q9BVK6|TMED9_HUMAN	0.03765678	0.35795313
sp|Q709C8-3|VP13C_HUMAN	0.03768539	0.7957244
sp|Q9H3N1|TMX1_HUMAN	0.03775024	0.2178309
sp|P32456|GBP2_HUMAN	0.03800583	0.19149946
sp|P30086|PEBP1_HUMAN	0.0382576	0.79861414
sp|Q13308-6|PTK7_HUMAN	0.03833389	0.6298893
sp|Q12797-10|ASPH_HUMAN	0.03833771	0.86708647
sp|P35858-2|ALS_HUMAN	0.03899956	0
sp|P39687|AN32A_HUMAN	0.03905296	0.5204253
sp|O43488|ARK72_HUMAN	0.03908348	0.1342476
sp|P61956-2|SUMO2_HUMAN	0.03983307	0
sp|P07437|TBB5_HUMAN	0.04009247	0.1342476
sp|Q14980-2|NUMA1_HUMAN	0.04023552	0.7871646
sp|Q16527|CSRP2_HUMAN	0.04083633	0.09894868
sp|Q6DD88|ATLA3_HUMAN	0.04119492	0.03367973
sp|P02748|CO9_HUMAN	0.04137421	0.49004218
sp|Q6P2Q9|PRP8_HUMAN	0.04153824	0.09894868
sp|Q92629-3|SGCD_HUMAN	0.04164314	0.06291623
sp|Q9Y383-3|LC7L2_HUMAN	0.04169464	0.7061832
sp|Q13347|EIF3I_HUMAN	0.04174042	0.09339783
sp|O00754-2|MA2B1_HUMAN	0.04180527	0.40256184
sp|P62269|RS18_HUMAN	0.04191017	0.40256184
sp|P29692-2|EF1D_HUMAN	0.0421505	0.09894868
sp|P48739|PIPNB_HUMAN	0.04233742	0
sp|P34896-2|GLYC_HUMAN	0.04253197	0
sp|P50579-2|MAP2_HUMAN	0.04339409	0
sp|Q9Y265|RUVB1_HUMAN	0.04339409	0
sp|Q9Y5M8|SRPRB_HUMAN	0.04365158	0.30372584
sp|P52888|THOP1_HUMAN	0.04420281	0.45033538
sp|Q9NYU2-2|UGGG1_HUMAN	0.04420471	0.03956581
sp|Q9NUV9|GIMA4_HUMAN	0.0444603	0.19410844
sp|P61916-2|NPC2_HUMAN	0.04505348	0.19149946
sp|Q99873-3|ANM1_HUMAN	0.04560852	0.14672586
sp|Q9ULV4-3|COR1C_HUMAN	0.0456543	0.3852666
sp|P46782|RS5_HUMAN	0.04626274	0.16077396
sp|P26373|RL13_HUMAN	0.0465622	0.7588735
sp|P23368|MAOM_HUMAN	0.04663849	0.16649151
sp|Q8WXX5|DNJC9_HUMAN	0.04674912	0.19149946
sp|P43652|AFAM_HUMAN	0.04697418	0.1318641
sp|O00232|PSD12_HUMAN	0.04712772	0
sp|Q9BXP5-2|SRRT_HUMAN	0.04719925	0.40256184
sp|P11171-7|41_HUMAN	0.04776573	1.2095301
sp|P62081|RS7_HUMAN	0.04823875	0.09894868
sp|P19404|NDUV2_HUMAN	0.04848099	0.312067
sp|Q9BRF8-2|CPPED_HUMAN	0.04894257	0.43633315

sp|Q9UJU6-2|DBNL_HUMAN	0.04928207	0.06291623
sp|Q13885|TBB2A_HUMAN	0.04940414	0
sp|P18669|PGAM1_HUMAN	0.04965019	0.4488957
sp|P17174|AATC_HUMAN	0.04995346	0.897507
sp|P61163|ACTZ_HUMAN	0.05005264	0.30372584
sp|P09496-2|CLCA_HUMAN	0.05059242	0.09750395
sp|P07195|LDHB_HUMAN	0.05081558	0.4488957
sp|P61247|RS3A_HUMAN	0.05082703	0.95332193
sp|Q9NX63|MIC19_HUMAN	0.05184937	0
sp|Q16851|UGPA_HUMAN	0.05190659	0.06644611
sp|P14314-2|GLU2B_HUMAN	0.05222321	1.0992733
sp|Q15005|SPCS2_HUMAN	0.05226135	0.35795313
sp|P23396|RS3_HUMAN	0.05293083	1.2669417
sp|Q13724-2|MOGS_HUMAN	0.05331993	0.05145278
sp|P69891|HBG1_HUMAN	0.0533905	0.656254
sp|O60763-2|USO1_HUMAN	0.05361557	0.3516469
sp|P51991|ROA3_HUMAN	0.05365372	0.1342476
sp|Q7Z7G0|TARSH_HUMAN	0.05392456	0.19149946
sp|Q9BRA2|TXD17_HUMAN	0.05410385	0.1342476
sp|Q9UNE7-2|CHIP_HUMAN	0.05423355	0.19149946
sp|O75746-2|CMC1_HUMAN	0.05453682	0.09894868
sp|Q9UBE0|SAE1_HUMAN	0.0547781	0.28516325
sp|P26639-2|SYTC_HUMAN	0.0554657	0.22362706
sp|Q9Y639-4|NPTN_HUMAN	0.05578613	0
sp|O95479|G6PE_HUMAN	0.05582047	0
sp|P61803|DAD1_HUMAN	0.0562973	0
sp|O95394-3|AGM1_HUMAN	0.0563488	0
sp|P30837|AL1B1_HUMAN	0.05654717	0.09339783
sp|P13639|EF2_HUMAN	0.05683327	0.39459047
sp|P49458|SRP09_HUMAN	0.0571003	0
sp|Q15149-3|PLEC_HUMAN	0.05733299	0.656254
sp|Q13247-3|SRSF6_HUMAN	0.05754662	0.19149946
sp|Q9Y6C2|EMIL1_HUMAN	0.05785942	0.64083964
sp|O60664-4|PLIN3_HUMAN	0.05792236	0.65625405
sp|P57737-4|CORO7_HUMAN	0.05827236	0.45033538
sp|O14936-2|CSKP_HUMAN	0.05860996	0.14672586
sp|P00740|FA9_HUMAN	0.059412	0
sp|P48444|COPD_HUMAN	0.05976105	0.7223306
sp|O75503|CLN5_HUMAN	0.0600071	0.2178309
sp|P63027|VAMP2_HUMAN	0.06035614	0
sp|P55145|MANF_HUMAN	0.06132317	0.7827403
sp|Q9H4G4|GAPR1_HUMAN	0.0621109	0.2178309
sp|P31146|COR1A_HUMAN	0.06214142	0.9764444
sp|P53992|SC24C_HUMAN	0.0626049	0.1342476
sp|Q9P2X0-2|DPM3_HUMAN	0.06373787	0

sp|Q9NZB2-6|F120A_HUMAN	0.06434059	0.312067
sp|A0A0C4DH31|HV118_HUMAN	0.06534576	0
sp|Q9NZ01|TECR_HUMAN	0.0655756	0.7588735
sp|Q96HY6|DDRGK_HUMAN	0.06646919	0.656254
sp|P13716-2|HEM2_HUMAN	0.0670948	0.5283125
sp|P23083|HV102_HUMAN	0.06715012	0
sp|P27338|AOFB_HUMAN	0.06744576	0.27686286
sp|P62277|RS13_HUMAN	0.06745338	0.8983557
sp|P09543-2|CN37_HUMAN	0.06770706	0.3005443
sp|P22102|PUR2_HUMAN	0.06850243	0.21439649
sp|P24534|EF1B_HUMAN	0.06903648	0.19149946
sp|P30613-2|KPYR_HUMAN	0.06912041	0.656254
sp|Q13630|FCL_HUMAN	0.07008934	0
sp|O14791-2|APOL1_HUMAN	0.07025147	0.19149946
sp|Q09028-3|RBBP4_HUMAN	0.07098389	0.656254
sp|Q15019-2|SEPT2_HUMAN	0.07228851	0.771065
sp|P63151-2|2ABA_HUMAN	0.07284355	0.312067
sp|P07814|SYEP_HUMAN	0.07522965	0.5283125
sp|P12814|ACTN1_HUMAN	0.0757103	0
sp|P21912|SDHB_HUMAN	0.07612038	0.7061832
sp|Q15257-2|PTPA_HUMAN	0.07618141	0.4075265
sp|Q9Y5S9-2|RBM8A_HUMAN	0.07631016	0.5204253
sp|P30519|HMOX2_HUMAN	0.07645035	0
sp|P43686|PRS6B_HUMAN	0.07771206	0.10138063
sp|P48147|PPCE_HUMAN	0.07792282	0.27902296
sp|Q9HC38|GLOD4_HUMAN	0.07801628	0.48757824
sp|Q00341-2|VIGLN_HUMAN	0.07844162	0.27902296
sp|P01034|CYTC_HUMAN	0.07861328	0.45033538
sp|P63267|ACTH_HUMAN	0.07891083	0.656254
sp|Q99798|ACON_HUMAN	0.07900047	0.20517272
sp|P25398|RS12_HUMAN	0.07924271	0.6115017
sp|P16615|AT2A2_HUMAN	0.07971001	0.57727534
sp|P78406|RAE1L_HUMAN	0.08184433	0
sp|O15061|SYNEM_HUMAN	0.08187676	0.65625405
sp|Q15661|TRYB1_HUMAN	0.0819397	0.5176613
sp|P31689-2|DNJA1_HUMAN	0.08240509	0.45033538
sp|P40763-3|STAT3_HUMAN	0.08269119	0.40256184
sp|Q9UGI8-2|TES_HUMAN	0.08270454	1.2954973
sp|P52306-4|GDS1_HUMAN	0.08348846	0.5204253
sp|Q96AE4-2|FUBP1_HUMAN	0.08349419	0.95533764
sp|P53621-2|COPA_HUMAN	0.08400345	0.3555403
sp|P55795|HNRH2_HUMAN	0.08461952	0.6298893
sp|Q9UNH7-2|SNX6_HUMAN	0.08496094	0
sp|A0AVT1|UBA6_HUMAN	0.08568764	0.1342476
sp|Q16555|DPYL2_HUMAN	0.08603287	0.28339192

sp|Q9UBS4|DJB11_HUMAN	0.08604622	0.45033538
sp|Q92945|FUBP2_HUMAN	0.08635521	0.52598757
sp|P13010|XRCC5_HUMAN	0.08901405	0.36969694
sp|Q08431|MFGM_HUMAN	0.0891571	0.35795313
sp|O14974-3|MYPT1_HUMAN	0.09013367	0.84879977
sp|Q9Y678|COPG1_HUMAN	0.0915432	0.81613266
sp|P02760|AMBP_HUMAN	0.09161568	0.8059303
sp|Q63ZY3-3|KANK2_HUMAN	0.09178925	0.56808305
sp|Q6PCB0|VWA1_HUMAN	0.09190941	0.61034113
sp|Q9UBQ7|GRHPR_HUMAN	0.09210205	0.25789237
sp|O60547-2|GMDS_HUMAN	0.09236908	0.7061832
sp|Q9Y230|RUVB2_HUMAN	0.0927105	0.08449883
sp|Q5JRX3-3|PREP_HUMAN	0.09334946	1.1932944
sp|P35908|K22E_HUMAN	0.09469986	0.5025228
sp|Q93009-3|UBP7_HUMAN	0.09488297	0.19149946
sp|P60228|EIF3E_HUMAN	0.09569168	1.0485198
sp|P61353|RL27_HUMAN	0.09589005	1.1932944
sp|P51884|LUM_HUMAN	0.0963707	0.8898569
sp|P46783|RS10_HUMAN	0.09718514	0.45033538
sp|P21266|GSTM3_HUMAN	0.09727097	0.93112767
sp|Q6UW68|TM205_HUMAN	0.09753799	0.7827403
sp|Q15274|NADC_HUMAN	0.09959412	0.35795313
sp|P05155-2|IC1_HUMAN	0.1000824	0.45033538
sp|P05165-2|PCCA_HUMAN	0.1020813	1.1073172
sp|Q13200|PSMD2_HUMAN	0.10212898	0.2178309
sp|Q9NRV9|HEBP1_HUMAN	0.10293579	1.0485198
sp|O60506-3|HNRPQ_HUMAN	0.10312653	0.6298893
sp|Q9H2D6-2|TARA_HUMAN	0.10355759	1.0645995
sp|P36871|PGM1_HUMAN	0.10406494	0
sp|Q9HD45|TM9S3_HUMAN	0.10450363	0
sp|Q07960|RHG01_HUMAN	0.10606766	1.120602
sp|Q13838-2|DX39B_HUMAN	0.10643768	1.2095301
sp|Q13596-2|SNX1_HUMAN	0.10665894	0
sp|P27918|PROP_HUMAN	0.10770798	0.19149946
sp|P50454|SERPH_HUMAN	0.10779381	0.84879977
sp|P55058|PLTP_HUMAN	0.10789108	0.19149946
sp|Q92747|ARC1A_HUMAN	0.10928726	0
sp|Q9P2T1-2|GMPR2_HUMAN	0.10967827	0.70235044
sp|Q7L576|CYFP1_HUMAN	0.10975456	0.312067
sp|P00441|SODC_HUMAN	0.11000824	0.84879977
sp|Q14914-2|PTGR1_HUMAN	0.11235809	0
sp|P34932|HSP74_HUMAN	0.11249352	0.67256224
sp|Q13642-1|FHL1_HUMAN	0.11410713	0.656254
sp|Q9P258|RCC2_HUMAN	0.11423874	0.7588735
sp|P62140|PP1B_HUMAN	0.11433792	0.35795313

sp|Q8TD19|NEK9_HUMAN	0.11474037	0.45033538
sp|Q9NR45|SIAS_HUMAN	0.11583901	0.7114226
sp|O60831|PRAF2_HUMAN	0.11603737	0.91601294
sp|O75306-2|NDUS2_HUMAN	0.11604214	1.2095301
sp|P22352|GPX3_HUMAN	0.11749077	0.5204253
sp|P62857|RS28_HUMAN	0.11828232	0.5204253
sp|P50395|GDIB_HUMAN	0.12011147	0.2005696
sp|Q14258|TRI25_HUMAN	0.12201309	1.2392054
sp|Q9BR76|COR1B_HUMAN	0.12316322	0.2591514
sp|P05452|TETN_HUMAN	0.12491226	0.5204253
sp|O43396|TXNL1_HUMAN	0.12552261	0
sp|Q9Y5Z4|HEBP2_HUMAN	0.12642479	1.2773042
sp|P55735-2|SEC13_HUMAN	0.12724876	0.8983557
sp|P51991-2|ROA3_HUMAN	0.12728119	0
sp|Q06278|AOXA_HUMAN	0.12758064	0.45033538
sp|P62917|RL8_HUMAN	0.12782669	0.95332193
sp|Q16787-3|LAMA3_HUMAN	0.12856483	0.656254
sp|Q9C0C2|TB182_HUMAN	0.12984657	1.0634323
sp|Q96M27-3|PRRC1_HUMAN	0.12997341	0.45033538
sp|Q9GZM7-3|TINAL_HUMAN	0.13040543	0
sp|P53602|MVD1_HUMAN	0.13089752	0.09894868
sp|Q15293|RCN1_HUMAN	0.13171577	0.84879977
sp|P36269-3|GGT5_HUMAN	0.13200665	0.9533461
sp|P12268|IMDH2_HUMAN	0.13424683	0.7827403
sp|P21399|ACOC_HUMAN	0.13466358	0.83646035
sp|P09012|SNRPA_HUMAN	0.13475418	0.6070219
sp|P12956|XRCC6_HUMAN	0.1351757	1.2191175
sp|Q12792-3|TWF1_HUMAN	0.13543701	0.7827403
sp|P10644|KAP0_HUMAN	0.1371994	0.312067
sp|Q03113|GNA12_HUMAN	0.13757896	0.656254
sp|P08185|CBG_HUMAN	0.138031	1.1932944
sp|P15880|RS2_HUMAN	0.13851738	0.6298893
sp|Q15637-2|SF01_HUMAN	0.1405487	1.1932944
sp|P29144|TPP2_HUMAN	0.14171982	0.61034113
sp|Q93034|CUL5_HUMAN	0.14255524	0.19149946
sp|P53618|COPB_HUMAN	0.14258003	1.0604883
sp|Q9BUT1|BDH2_HUMAN	0.14411736	1.120602
sp|Q99426|TBCB_HUMAN	0.14556885	0.7061832
sp|P31150|GDIA_HUMAN	0.14625168	0.3516469
sp|P04062-2|GLCM_HUMAN	0.14670753	0.45033538
sp|P11940-2|PABP1_HUMAN	0.14828491	1.1505735
sp|Q04446|GLGB_HUMAN	0.14934158	0
sp|O95302-3|FKBP9_HUMAN	0.15023041	0.7827403
BirA-TRIP6_BirAT6	0.15033245	0.19149946
sp|P55809|SCOT1_HUMAN	0.15109825	0.35795313

sp|Q9NSK0-5|KLC4_HUMAN	0.15120506	0.656254
sp|P24821-4|TENA_HUMAN	0.15139961	0.907945
sp|P39023|RL3_HUMAN	0.1514206	0.5111962
sp|P84085|ARF5_HUMAN	0.15215874	0.656254
sp|P07360|CO8G_HUMAN	0.1527977	0.7827403
sp|P14550|AK1A1_HUMAN	0.15385818	0.6385048
sp|P11678|PERE_HUMAN	0.15507889	0.28516325
sp|Q9UHB6-4|LIMA1_HUMAN	0.15666962	0.7588735
sp|P42226|STAT6_HUMAN	0.15782166	0
sp|P60660-2|MYL6_HUMAN	0.15930367	0.7827403
sp|O75828|CBR3_HUMAN	0.15983582	0
sp|O60832|DKC1_HUMAN	0.16145897	1.1932944
sp|P32969|RL9_HUMAN	0.16238976	0.91601294
sp|A0A0C4DH38|HV551_HUMAN	0.16260147	1.1932944
sp|P07357|CO8A_HUMAN	0.16570282	0.95332193
sp|O43852-3|CALU_HUMAN	0.1672802	0.5111962
sp|Q9UJZ1-2|STML2_HUMAN	0.16790009	0.35795313
sp|P98082-2|DAB2_HUMAN	0.1694336	1.1932944
sp|P46781|RS9_HUMAN	0.17037201	0.8339169
sp|P28161|GSTM2_HUMAN	0.17126656	0.656254
sp|P31321|KAP1_HUMAN	0.17234612	0.656254
sp|Q9C0E8-4|LNP_HUMAN	0.17348099	0.19149946
sp|P13693|TCTP_HUMAN	0.1741333	1.1932944
sp|Q99733-2|NP1L4_HUMAN	0.17434025	0.48520416
sp|Q96D15|RCN3_HUMAN	0.17623234	0.80804527
sp|P09871|C1S_HUMAN	0.17664146	0
sp|P52895|AK1C2_HUMAN	0.17729282	0
sp|O60610-2|DIAP1_HUMAN	0.17787838	0.6298893
sp|O94855-2|SC24D_HUMAN	0.17914963	0
sp|P07951-3|TPM2_HUMAN	0.18091393	0.30372584
sp|P0CG38|POTEI_HUMAN	0.18518639	0.656254
sp|P62714|PP2AB_HUMAN	0.18554306	0.656254
sp|O95861-4|BPNT1_HUMAN	0.18642807	0.30372584
sp|P18583-10|SON_HUMAN	0.18720245	0.6070219
sp|P46821|MAP1B_HUMAN	0.19052696	0.7588735
sp|P84098|RL19_HUMAN	0.1905613	0.7827403
sp|Q99538-2|LGMN_HUMAN	0.19428825	1.1932944
sp|P02753|RET4_HUMAN	0.19549942	1.0301651
sp|Q9BTE3-2|MCMBP_HUMAN	0.19579887	0.45033538
sp|P62195|PRS8_HUMAN	0.19704437	0.7827403
sp|P63241|IF5A1_HUMAN	0.19750786	0.656254
sp|O00483|NDUA4_HUMAN	0.19888687	1.1932944
sp|O00233-2|PSMD9_HUMAN	0.200037	1.1932944
sp|Q06828|FMOD_HUMAN	0.20061493	1.1932944
sp|Q9HCN8|SDF2L_HUMAN	0.20548248	0.7827403

sp|P04406|G3P_HUMAN	0.2058487	0.656254
sp|Q15436|SC23A_HUMAN	0.20792294	0.1342476
sp|P0C0L5|CO4B_HUMAN	0.20871735	1.1932944
sp|P43243|MATR3_HUMAN	0.20907593	0.95332193
sp|Q9BX97|PLVAP_HUMAN	0.21100616	0
sp|P62913-2|RL11_HUMAN	0.21405983	1.1932944
sp|O43294|TGFI1_HUMAN	0.21412277	1.1932944
sp|Q9BW30|TPPP3_HUMAN	0.21626854	0
sp|P01892|1A02_HUMAN	0.21832275	0.91601294
sp|Q9H008|LHPP_HUMAN	0.21845436	0.7957244
sp|Q8TAQ2-2|SMRC2_HUMAN	0.21977234	0.2178309
sp|P62191-2|PRS4_HUMAN	0.22154236	0.7061832
sp|Q00796|DHSO_HUMAN	0.22399521	1.1505735
sp|Q9UNS2|CSN3_HUMAN	0.22559929	0.656254
sp|P60981|DEST_HUMAN	0.22732544	0.84879977
sp|Q53GG5-2|PDLI3_HUMAN	0.22751236	1.0485198
sp|P14618-2|KPYM_HUMAN	0.23034859	0.5204253
sp|Q99961-3|SH3G1_HUMAN	0.23050499	0.656254
sp|P60891|PRPS1_HUMAN	0.23163986	0.656254
sp|P62854|RS26_HUMAN	0.2372818	0.7827403
sp|A0A0B4J1X8|HV343_HUMAN	0.23983383	0
sp|Q15437|SC23B_HUMAN	0.24015236	0.656254
sp|Q96PD5-2|PGRP2_HUMAN	0.24511719	1.1276597
sp|P40261|NNMT_HUMAN	0.2456665	0.91601294
sp|P02749|APOH_HUMAN	0.24912262	0.27902296
sp|P02765|FETUA_HUMAN	0.24927711	1.0353775
sp|P20810-4|ICAL_HUMAN	0.2506237	0.91601294
sp|Q8TD06|AGR3_HUMAN	0.25260544	0
sp|Q03519|TAP2_HUMAN	0.25284576	0.656254
sp|Q13310-2|PABP4_HUMAN	0.2568798	0.656254
sp|P31947|1433S_HUMAN	0.25946426	0.6298893
sp|P62736|ACTA_HUMAN	0.26127052	0.656254
sp|P62249|RS16_HUMAN	0.26145363	0.91601294
sp|P12814-2|ACTN1_HUMAN	0.2649994	1.1932944
sp|P02766|TTHY_HUMAN	0.26673126	1.0485198
sp|P02747|C1QC_HUMAN	0.27113533	1.1932944
sp|P27635|RL10_HUMAN	0.28592682	0.656254
sp|Q71UM5|RS27L_HUMAN	0.28954506	1.1932944
sp|P62910|RL32_HUMAN	0.29045105	1.1932944
sp|P46977|STT3A_HUMAN	0.2950592	1.1932944
sp|Q9BWS9-3|CHID1_HUMAN	0.29943848	0.656254
sp|O60825-2|F262_HUMAN	0.30013275	0.656254
sp|Q7Z4H8|PLGT3_HUMAN	0.30786133	0.656254
sp|P39059|COFA1_HUMAN	0.30911255	1.2095301
sp|P12111-4|CO6A3_HUMAN	0.3194294	0.656254

sp|Q8TBC4-2|UBA3_HUMAN	0.32211685	0.7827403
sp|Q9Y5P6-2|GMPPB_HUMAN	0.32845116	0.35795313
sp|P0DOX3|IGD_HUMAN	0.33233833	0.656254
sp|P14207|FOLR2_HUMAN	0.33938026	0.656254
sp|Q96AY3|FKB10_HUMAN	0.34247208	1.1932944
sp|O75533|SF3B1_HUMAN	0.34399223	1.1932944
sp|Q687X5|STEA4_HUMAN	0.34662247	1.1932944
sp|Q08379|GOGA2_HUMAN	0.3489027	1.1932944
sp|P16104|H2AX_HUMAN	0.3490486	0
sp|Q9NR56-2|MBNL1_HUMAN	0.35448837	1.1932944
sp|Q14141-2|SEPT6_HUMAN	0.3569088	0.656254
sp|Q8WX93-5|PALLD_HUMAN	0.36261177	0.7061832
sp|P62829|RL23_HUMAN	0.38051224	1.1932944
sp|A0A0C4DH29|HV103_HUMAN	0.39317703	0.656254
sp|Q92599-3|SEPT8_HUMAN	0.39941406	1.1932944
sp|P0DOX2|IGA2_HUMAN	0.40577507	1.1505735
sp|Q15046|SYK_HUMAN	0.4100952	0.656254
sp|P02746|C1QB_HUMAN	0.414196	1.1932944
sp|P62633-3|CNBP_HUMAN	0.44272327	1.1932944
sp|P01619|KV320_HUMAN	0.44629288	0.656254
sp|P00738|HPT_HUMAN	0.4470272	0.656254
sp|Q14697|GANAB_HUMAN	0.4565487	1.1932944
sp|P08779|K1C16_HUMAN	0.45977783	1.1932944
sp|P0C0L4|CO4A_HUMAN	0.46147156	1.1932944
sp|P02751-15|FINC_HUMAN	0.46273994	0.656254
sp|P0DOX7|IGK_HUMAN	0.46954536	1.2095301
sp|P35637-2|FUS_HUMAN	0.47401237	1.1932944
sp|P69892|HBG2_HUMAN	0.4740162	0.656254
sp|P47895|AL1A3_HUMAN	0.50132847	0.656254
sp|P35542|SAA4_HUMAN	0.5135155	1.1932944
sp|Q14894|CRYM_HUMAN	0.5332279	0.7827403
sp|Q05682|CALD1_HUMAN	0.53640175	1.1505735
sp|P35080-2|PROF2_HUMAN	0.5511799	0.656254
sp|P50135|HNMT_HUMAN	0.55207825	1.1932944
sp|Q96HC4|PDLI5_HUMAN	0.5705948	0.656254
sp|Q15063-3|POSTN_HUMAN	0.6360855	1.1932944
sp|P01782|HV309_HUMAN	0.6711464	0.656254
sp|P0DP03|HV335_HUMAN	0.69286346	1.1932944
sp|P01624|KV315_HUMAN	0.7023983	1.1932944
sp|O76011|KRT34_HUMAN	0.70775795	0.656254
sp|P48741|HSP77_HUMAN	0.72177505	0.656254
sp|P61018-2|RAB4B_HUMAN	0.78552246	0.656254
sp|A0A0C4DH25|KVD20_HUMAN	0.8185253	0.656254
sp|A0A0C4DH41|HV461_HUMAN	0.84370804	0.656254
sp|Q03591|FHR1_HUMAN	0.85528183	1.1932944

sp|Q15063-2|POSTN_HUMAN	0.8555012	0.656254
sp|P02533|K1C14_HUMAN	0.889966	0.45033538
sp|P02461|CO3A1_HUMAN	0.89585686	1.1932944
sp|P01591|IGJ_HUMAN	0.9861374	1.1932944
sp|P01834|IGKC_HUMAN	1.0725155	1.1932944
sp|Q13976-2|KGP1_HUMAN	1.1705475	0.656254
sp|P01780|HV307_HUMAN	1.2133827	0.656254
sp|A0A075B6P5|KV228_HUMAN	1.2322216	0.656254
sp|P06310|KV230_HUMAN	1.8338547	0.656254
sp|P20039|2B1B_HUMAN	1.914423	0.656254
