# Supplementary material for: Peptidylarginine deiminase 2 citrullinates MZB1 and promotes the secretion of IgM and IgA
Source: Front Immunol. 2023 Nov 29;14:1290585. doi: 10.3389/fimmu.2023.1290585 (PMC10716219; doi:10.3389/fimmu.2023.1290585)
Supplement: Supplementary file 9 [file DataSheet_9.pdf]

Supplemental Table 9: IPF3 vs controls

Accession #	Fold Change	p value (-log10)
sp|P12429|ANXA3_HUMAN	-1.6271172	7.665951
sp|Q15109-10|RAGE_HUMAN	-1.5686646	2.1818786
sp|P22748|CAH4_HUMAN	-1.4466152	2.6576471
sp|Q13510-2|ASAH1_HUMAN	-1.3556271	6.3204336
sp|P31947|1433S_HUMAN	-1.3444767	2.1818786
sp|O96009|NAPSA_HUMAN	-1.3268375	2.6576471
sp|Q10589-2|BST2_HUMAN	-1.3022957	1.6960104
sp|Q9NZA1-2|CLIC5_HUMAN	-1.2464657	3.5908275
sp|P09668|CATH_HUMAN	-1.2287712	3.218121
sp|P49407-2|ARRB1_HUMAN	-1.2282457	2.6576471
sp|P05141|ADT2_HUMAN	-1.2128143	2.6576471
sp|P62805|H4_HUMAN	-1.2096996	6.3204336
sp|Q01469|FABP5_HUMAN	-1.2020054	4.051346
sp|P07339|CATD_HUMAN	-1.1577797	8.112776
sp|P59665|DEF1_HUMAN	-1.1527138	2.1818786
sp|P99999|CYC_HUMAN	-1.1524124	2.1818786
sp|P05091|ALDH2_HUMAN	-1.1451511	9.04321
sp|O43760-2|SNG2_HUMAN	-1.1372395	1.6960104
sp|P21397|AOFA_HUMAN	-1.1177063	5.869831
sp|P08729|K2C7_HUMAN	-1.1137123	14.311137
sp|O95810|CAVN2_HUMAN	-1.0748329	3.7803535
sp|P31949|S10AB_HUMAN	-1.0689125	2.6576471
sp|P84103-2|SRSF3_HUMAN	-1.0538063	3.1266599
sp|P02788|TRFL_HUMAN	-1.0407887	15.051499
sp|Q6NUK1-2|SCMC1_HUMAN	-1.0320415	2.6576471
sp|P35241|RADI_HUMAN	-1.0239401	3.5908275
sp|P50895|BCAM_HUMAN	-1.0207748	5.1321483
sp|P05362|ICAM1_HUMAN	-1.0161095	4.247887
sp|P00325|ADH1B_HUMAN	-1.0047016	4.051346
sp|Q96TA1-2|NIBL1_HUMAN	-1.0021286	2.7624686
sp|P09758|TACD2_HUMAN	-0.9999943	2.1818786
sp|P51659|DHB4_HUMAN	-0.9987984	10.489762
sp|P40121|CAPG_HUMAN	-0.9936905	5.180782
sp|Q16698-2|DECR_HUMAN	-0.9910545	3.3283036
sp|O00743-3|PPP6_HUMAN	-0.9844971	1.6960104
sp|Q8WWI1-5|LMO7_HUMAN	-0.9769344	2.3787699
sp|Q9UPQ0|LIMC1_HUMAN	-0.9669437	1.9951487
sp|P51149|RAB7A_HUMAN	-0.9643917	4.044672
sp|P29401-2|TKT_HUMAN	-0.9531841	12.078198
sp|P04179-4|SODM_HUMAN	-0.9256954	3.1266599
sp|P49756|RBM25_HUMAN	-0.9176559	1.6960104
sp|P09467|F16P1_HUMAN	-0.9150848	3.5442007
sp|P01903|DRA_HUMAN	-0.9133682	2.1818786

sp|P84090|ERH_HUMAN	-0.895052	2.1818786
sp|P23381|SYWC_HUMAN	-0.8869019	4.7413087
sp|O60814|H2B1K_HUMAN	-0.8854408	1.4104178
sp|P60903|S10AA_HUMAN	-0.8835373	1.6960104
sp|Q9Y624|JAM1_HUMAN	-0.8819485	2.6576471
sp|P13073|COX41_HUMAN	-0.8770142	1.6960104
sp|Q00325-2|MPCP_HUMAN	-0.8701935	3.1266599
sp|P48735-2|IDHP_HUMAN	-0.8642883	4.051346
sp|P09960|LKHA4_HUMAN	-0.8636017	9.625019
sp|P05109|S10A8_HUMAN	-0.8607922	3.5908275
sp|Q08722-2|CD47_HUMAN	-0.8580704	1.6960104
sp|Q9HC35-2|EMAL4_HUMAN	-0.8539848	2.6576471
sp|P00505|AATM_HUMAN	-0.8482266	4.8244376
sp|P41218|MNDA_HUMAN	-0.8461285	2.1818786
sp|Q03135|CAV1_HUMAN	-0.8460617	2.1818786
sp|P20292|AL5AP_HUMAN	-0.8367462	1.4104178
sp|P61626|LYSC_HUMAN	-0.8357964	2.6576471
sp|P40926|MDHM_HUMAN	-0.8237	6.596239
sp|Q00765|REEP5_HUMAN	-0.8226395	3.1266599
sp|P12821-2|ACE_HUMAN	-0.8207836	2.6576471
sp|O00159-3|MYO1C_HUMAN	-0.8196411	6.5096054
sp|Q13751|LAMB3_HUMAN	-0.8105183	1.6960104
sp|O75695|XRP2_HUMAN	-0.8095589	1.6960104
sp|P02786|TFR1_HUMAN	-0.8083725	4.051346
sp|Q15599-2|NHRF2_HUMAN	-0.8061409	1.6997428
sp|P11310-2|ACADM_HUMAN	-0.8020229	4.051346
sp|P05164-3|PERM_HUMAN	-0.7909775	7.9773483
sp|Q6PIU2-2|NCEH1_HUMAN	-0.7890072	2.432837
sp|P25774|CATS_HUMAN	-0.7850552	1.6960104
sp|P33121-3|ACSL1_HUMAN	-0.7844334	2.1818786
sp|P51648-2|AL3A2_HUMAN	-0.7693386	2.4829872
sp|Q9UHG3|PCYOX_HUMAN	-0.7686997	4.247887
sp|P13796|PLSL_HUMAN	-0.7679939	11.224656
sp|Q13740-2|CD166_HUMAN	-0.7621956	3.2925725
sp|P43304|GPDM_HUMAN	-0.758297	2.8739974
sp|P05026-2|AT1B1_HUMAN	-0.756525	2.6576471
sp|P22626|ROA2_HUMAN	-0.7515755	6.2055726
sp|P05023|AT1A1_HUMAN	-0.7497368	7.6602716
sp|O60271-4|JIP4_HUMAN	-0.7483597	1.6960104
sp|P11233|RALA_HUMAN	-0.7421303	2.1818786
sp|Q07157|ZO1_HUMAN	-0.7389755	4.964395
sp|P17931|LEG3_HUMAN	-0.7385216	2.6576471
sp|O95340-2|PAPS2_HUMAN	-0.7363434	2.1818786
sp|Q9UGT4|SUSD2_HUMAN	-0.7353687	2.151766
sp|Q6NZI2|CAVN1_HUMAN	-0.7173271	6.3204336

sp|P50225|ST1A1_HUMAN	-0.7158432	1.6960104
sp|P23246|SFPQ_HUMAN	-0.7128372	4.044672
sp|P10515|ODP2_HUMAN	-0.7115688	2.6576471
sp|P36957|ODO2_HUMAN	-0.7102165	2.923088
sp|O43707|ACTN4_HUMAN	-0.7097054	13.694518
sp|Q13011|ECH1_HUMAN	-0.7070198	4.964395
sp|Q9ULV4-3|COR1C_HUMAN	-0.70051	3.342776
sp|P10253|LYAG_HUMAN	-0.6992531	3.1266599
sp|P15311|EZRI_HUMAN	-0.6930618	8.079533
sp|P00352|AL1A1_HUMAN	-0.6922112	10.298183
sp|P09622|DLDH_HUMAN	-0.6897411	3.673847
sp|O15143|ARC1B_HUMAN	-0.6874962	2.3143692
sp|P29590|PML_HUMAN	-0.6872349	2.5815322
sp|P63096|GNAI1_HUMAN	-0.6827412	1.6960104
sp|Q9NZN4|EHD2_HUMAN	-0.6799774	6.429521
sp|Q8TCJ2|STT3B_HUMAN	-0.6791592	1.6960104
sp|P63092-3|GNAS2_HUMAN	-0.6781216	2.9310203
sp|P13804|ETFA_HUMAN	-0.6759281	4.4116287
sp|O95994|AGR2_HUMAN	-0.6758099	2.6576471
sp|P19338|NUCL_HUMAN	-0.6734963	8.112776
sp|Q8NF37|PCAT1_HUMAN	-0.6704006	1.560883
sp|P53007|TXTP_HUMAN	-0.6696415	2.6576471
sp|Q8N335|GPD1L_HUMAN	-0.6686649	1.6960104
sp|P05107|ITB2_HUMAN	-0.6658154	2.544769
sp|Q02252-2|MMSA_HUMAN	-0.6604443	3.8337784
sp|P07988|PSPB_HUMAN	-0.6563664	2.7624686
sp|P0DPI2-2|GAL3A_HUMAN	-0.6559296	1.6960104
sp|Q07955-3|SRSF1_HUMAN	-0.6551609	4.509013
sp|P61106|RAB14_HUMAN	-0.655098	3.5098467
sp|Q9UL18|AGO1_HUMAN	-0.6541824	1.4104178
sp|P51649-2|SSDH_HUMAN	-0.6540051	2.1818786
sp|P62917|RL8_HUMAN	-0.6529388	2.3143692
sp|P31040|SDHA_HUMAN	-0.6509819	3.5945017
sp|P06702|S10A9_HUMAN	-0.6508751	3.2221727
sp|P56199|ITA1_HUMAN	-0.6478806	6.253077
sp|P37837|TALDO_HUMAN	-0.6475067	6.458061
sp|P09110|THIK_HUMAN	-0.6468315	1.9647322
sp|P40429|RL13A_HUMAN	-0.6462536	1.6960104
sp|P16284-3|PECA1_HUMAN	-0.6451798	4.1424985
sp|Q8NBX0|SCPDL_HUMAN	-0.6451035	1.6960104
sp|Q16762|THTR_HUMAN	-0.6438599	1.9647322
sp|P61604|CH10_HUMAN	-0.64361	3.1266599
sp|P22307-8|NLTP_HUMAN	-0.6429863	1.6960104
sp|O75390|CISY_HUMAN	-0.6414661	3.9403465
sp|Q04917|1433F_HUMAN	-0.641449	5.417897

sp|Q02878|RL6_HUMAN	-0.6405907	1.6960104
sp|P07099|HYEP_HUMAN	-0.6376648	6.6615443
sp|Q9H0U4|RAB1B_HUMAN	-0.6365671	1.6960104
sp|P42765|THIM_HUMAN	-0.6338539	5.172756
sp|Q14118|DAG1_HUMAN	-0.6327705	1.6960104
sp|P49591|SYSC_HUMAN	-0.6277714	4.033051
sp|Q02543|RL18A_HUMAN	-0.6266327	2.9807727
sp|P62906|RL10A_HUMAN	-0.6222553	1.827201
sp|Q92597|NDRG1_HUMAN	-0.6185532	2.1818786
sp|Q00839|HNRPU_HUMAN	-0.6182785	3.863062
sp|P51991|ROA3_HUMAN	-0.6115427	2.1818786
sp|P08575-10|PTPRC_HUMAN	-0.6091805	4.051346
sp|P04080|CYTB_HUMAN	-0.6073475	1.6960104
sp|P62258|1433E_HUMAN	-0.6066799	2.1818786
sp|P20700|LMNB1_HUMAN	-0.6053047	10.772392
sp|P35222|CTNB1_HUMAN	-0.6041012	2.3305986
sp|P51572-2|BAP31_HUMAN	-0.5996628	3.342776
sp|P25786-2|PSA1_HUMAN	-0.5980072	6.3204336
sp|Q9P0L0-2|VAPA_HUMAN	-0.5947342	3.1266599
sp|P30040|ERP29_HUMAN	-0.5939045	1.9413493
sp|Q02218-2|ODO1_HUMAN	-0.5927277	3.218121
sp|P11177-3|ODPB_HUMAN	-0.5917015	2.5461748
sp|P51858|HDGF_HUMAN	-0.5907421	2.1818786
sp|P17844-2|DDX5_HUMAN	-0.5899277	3.8337784
sp|P10606|COX5B_HUMAN	-0.5897713	1.6960104
sp|P26447|S10A4_HUMAN	-0.5887337	2.6576471
sp|Q9UBQ0-2|VPS29_HUMAN	-0.5858364	1.6960104
sp|P07954-2|FUMH_HUMAN	-0.5854607	2.6576471
sp|Q13228-4|SBP1_HUMAN	-0.584137	11.398167
sp|Q15907-2|RB11B_HUMAN	-0.5836983	2.1404836
sp|P30740|ILEU_HUMAN	-0.5829735	2.044628
sp|Q92888-2|ARHG1_HUMAN	-0.5812111	1.6960104
sp|P35221|CTNA1_HUMAN	-0.578085	9.140666
sp|P07910-2|HNRPC_HUMAN	-0.5765228	1.9647322
sp|P01833|PIGR_HUMAN	-0.5708561	4.0843706
sp|Q92841-3|DDX17_HUMAN	-0.5708427	4.5016932
sp|P38646|GRP75_HUMAN	-0.5698547	5.0099254
sp|P46781|RS9_HUMAN	-0.5681877	2.3047035
sp|P15121|ALDR_HUMAN	-0.5681229	3.5844223
sp|P53597|SUCA_HUMAN	-0.5677147	1.6960104
sp|P68366-2|TBA4A_HUMAN	-0.5669346	1.9647322
sp|P24752|THIL_HUMAN	-0.5645809	4.4994555
sp|P38606|VATA_HUMAN	-0.5626392	1.3668759
sp|Q16836-3|HCDH_HUMAN	-0.5614471	1.6960104
sp|P62873|GBB1_HUMAN	-0.559927	3.4656596

sp|Q9UL25|RAB21_HUMAN	-0.5594597	2.3143692
sp|P11413-2|G6PD_HUMAN	-0.5579186	3.1629772
sp|P40939|ECHA_HUMAN	-0.5563726	6.3886313
sp|P05556|ITB1_HUMAN	-0.5558357	10.022388
sp|Q9UBQ7|GRHPR_HUMAN	-0.5557499	1.7590232
sp|P07355-2|ANXA2_HUMAN	-0.5543556	2.894491
sp|Q14103-3|HNRPD_HUMAN	-0.5534134	3.3433785
sp|P08311|CATG_HUMAN	-0.5497284	4.051346
sp|P43490|NAMPT_HUMAN	-0.5478573	5.661589
sp|P10155|RO60_HUMAN	-0.5469046	1.7713763
sp|P17655|CAN2_HUMAN	-0.5465431	5.359168
sp|P35237|SPB6_HUMAN	-0.5464973	5.186614
sp|P30048-2|PRDX3_HUMAN	-0.5459938	2.6576471
sp|P62249|RS16_HUMAN	-0.5440321	1.6960104
sp|P21796|VDAC1_HUMAN	-0.5438938	4.876761
sp|Q6YHK3|CD109_HUMAN	-0.5420094	3.2450993
sp|Q06830|PRDX1_HUMAN	-0.541563	5.417897
sp|O94760|DDAH1_HUMAN	-0.539753	2.1818786
sp|P27824-2|CALX_HUMAN	-0.5378075	1.5121045
sp|Q9Y6N5|SQOR_HUMAN	-0.5369587	4.2164803
sp|P09211|GSTP1_HUMAN	-0.5361996	4.0772076
sp|P49748-2|ACADV_HUMAN	-0.5334911	8.234994
sp|P43034|LIS1_HUMAN	-0.5327892	3.1266599
sp|Q02978-2|M2OM_HUMAN	-0.5325546	1.4104178
sp|P61421|VA0D1_HUMAN	-0.5313129	2.653196
sp|P31146|COR1A_HUMAN	-0.5305443	4.2427664
sp|P25788-2|PSA3_HUMAN	-0.5284157	2.6576471
sp|P09525|ANXA4_HUMAN	-0.5248528	5.2000947
sp|Q9H8H3|MET7A_HUMAN	-0.5235882	1.6960104
sp|Q6WCQ1-2|MPRIP_HUMAN	-0.5232315	2.7472508
sp|Q9BR76|COR1B_HUMAN	-0.5144768	3.4656596
sp|Q9UMS4|PRP19_HUMAN	-0.5142098	2.6997027
sp|P55290-4|CAD13_HUMAN	-0.5123329	1.3815327
sp|Q9UFN0|NPS3A_HUMAN	-0.5102501	1.6960104
sp|P14543-2|NID1_HUMAN	-0.5098896	2.8734877
sp|P52272-2|HNRPM_HUMAN	-0.5071278	6.7073655
sp|P09651-3|ROA1_HUMAN	-0.5067596	1.4104178
sp|P0DP25|CALM3_HUMAN	-0.5067463	3.5908275
sp|O75874|IDHC_HUMAN	-0.5065174	3.9658737
sp|P16435|NCPR_HUMAN	-0.5039644	2.0265307
sp|P50213|IDH3A_HUMAN	-0.5036717	3.1266599
sp|P15586-2|GNS_HUMAN	-0.5032253	1.4104178
sp|P60900|PSA6_HUMAN	-0.4990044	4.534284
sp|P62987|RL40_HUMAN	-0.4950447	2.4829872
sp|O15230|LAMA5_HUMAN	-0.4942703	4.2145605

sp|P22897|MRC1_HUMAN	-0.4937077	3.9156206
sp|P08727|K1C19_HUMAN	-0.4936409	7.1634564
sp|P10768|ESTD_HUMAN	-0.4932575	1.8444856
sp|P14866-2|HNRPL_HUMAN	-0.489872	1.6960104
sp|P61158|ARP3_HUMAN	-0.489357	6.4715614
sp|P23141-2|EST1_HUMAN	-0.487812	9.673478
sp|Q00577|PURA_HUMAN	-0.4877586	2.6576471
sp|P63104|1433Z_HUMAN	-0.4873352	4.509013
sp|P02769|ALBU_BOVIN	-0.4870682	2.1743186
sp|P08559-2|ODPA_HUMAN	-0.4856815	3.8053288
sp|Q9HB40|RISC_HUMAN	-0.4849854	1.6960104
sp|Q9NQR4|NIT2_HUMAN	-0.4842644	2.6576471
sp|O00299|CLIC1_HUMAN	-0.4842567	5.2234774
sp|P49327|FAS_HUMAN	-0.482645	7.720088
sp|P00367|DHE3_HUMAN	-0.48106	2.2535503
sp|Q9Y5P6-2|GMPPB_HUMAN	-0.4806042	1.6960104
sp|P0DMV9|HS71B_HUMAN	-0.4769573	5.815963
sp|P28482|MK01_HUMAN	-0.4749546	2.6576471
sp|Q15233|NONO_HUMAN	-0.473938	4.044672
sp|P61247|RS3A_HUMAN	-0.4736443	3.417183
sp|P07305-2|H10_HUMAN	-0.4735203	1.6960104
sp|P37802|TAGL2_HUMAN	-0.4734364	4.655308
sp|Q9UNF0-2|PACN2_HUMAN	-0.4719315	1.6997428
sp|P52209-2|6PGD_HUMAN	-0.4718952	5.736467
sp|P46940|IQGA1_HUMAN	-0.4718647	11.63615
sp|Q9P2R7-2|SUCB1_HUMAN	-0.4711552	2.3047035
sp|Q9H223|EHD4_HUMAN	-0.4691925	2.7472508
sp|Q9BUJ2-4|HNRL1_HUMAN	-0.4669495	3.6087239
sp|Q07020-2|RL18_HUMAN	-0.4668903	1.4104178
sp|P49189|AL9A1_HUMAN	-0.4666634	5.7898192
sp|Q99536|VAT1_HUMAN	-0.4661388	3.673847
sp|Q86UP2-4|KTN1_HUMAN	-0.4656448	6.8152957
sp|Q02318|CP27A_HUMAN	-0.4650135	1.6960104
sp|Q15365|PCBP1_HUMAN	-0.4642601	2.5644996
sp|Q16555|DPYL2_HUMAN	-0.4635811	6.7267804
sp|P42330|AK1C3_HUMAN	-0.4590426	3.5908275
sp|O15144|ARPC2_HUMAN	-0.4574947	3.1266599
sp|P11021|BIP_HUMAN	-0.4553223	14.632371
sp|P46926|GNPI1_HUMAN	-0.4545097	2.1818786
sp|O75348|VATG1_HUMAN	-0.4541798	1.6960104
sp|P04083|ANXA1_HUMAN	-0.4535236	5.6658998
sp|P51178-2|PLCD1_HUMAN	-0.4530525	1.6960104
sp|Q9ULA0|DNPEP_HUMAN	-0.4527874	3.186611
sp|Q13045-2|FLII_HUMAN	-0.4526844	1.560883
sp|P29350-3|PTN6_HUMAN	-0.4514008	1.6997428

sp|P19367-2|HXK1_HUMAN	-0.4511433	6.57691
sp|P52565|GDIR1_HUMAN	-0.4492722	3.5908275
sp|Q1KMD3|HNRL2_HUMAN	-0.4492683	3.2079036
sp|Q86VB7-2|C163A_HUMAN	-0.446106	8.365451
sp|P50995-2|ANX11_HUMAN	-0.4459572	4.3100786
sp|P00390-2|GSHR_HUMAN	-0.4456177	1.6295799
sp|P30626-2|SORCN_HUMAN	-0.4438801	3.2221727
sp|P60953|CDC42_HUMAN	-0.4438591	2.8384566
sp|P05783|K1C18_HUMAN	-0.4435062	6.1187267
sp|P62937|PPIA_HUMAN	-0.4434586	1.4104178
sp|P51148-2|RAB5C_HUMAN	-0.4420471	1.6960104
sp|Q9BS26|ERP44_HUMAN	-0.4404831	3.316884
sp|Q13451|FKBP5_HUMAN	-0.4397945	2.4829872
sp|P36578|RL4_HUMAN	-0.4389172	1.4351699
sp|P55072|TERA_HUMAN	-0.4374828	11.713043
sp|P10809|CH60_HUMAN	-0.4370518	4.293713
sp|P35270|SPRE_HUMAN	-0.4370251	2.6576471
sp|O43813|LANC1_HUMAN	-0.436573	1.6960104
sp|P27361|MK03_HUMAN	-0.4363861	1.6960104
sp|Q13425|SNTB2_HUMAN	-0.4361458	1.6604291
sp|Q04760-2|LGUL_HUMAN	-0.4332829	1.8604537
sp|P02545|LMNA_HUMAN	-0.4332066	14.65356
sp|P22695|QCR2_HUMAN	-0.4310684	1.3635377
sp|Q96KP4|CNDP2_HUMAN	-0.4297581	5.6946616
sp|O43837|IDH3B_HUMAN	-0.4284248	1.6960104
sp|P07737|PROF1_HUMAN	-0.4280109	4.0829325
sp|Q969V3-2|NCLN_HUMAN	-0.4268055	1.4104178
sp|Q06323|PSME1_HUMAN	-0.4245758	3.6746628
sp|Q14019|COTL1_HUMAN	-0.4243412	1.4278674
sp|Q7Z406|MYH14_HUMAN	-0.4232445	8.059228
sp|Q92817|EVPL_HUMAN	-0.4219933	2.193074
sp|P11766|ADHX_HUMAN	-0.4219894	2.816493
sp|O15247|CLIC2_HUMAN	-0.4197388	2.058973
sp|Q13217|DNJC3_HUMAN	-0.4184036	2.1818786
sp|P06733|ENOA_HUMAN	-0.4176159	7.501921
sp|P61978-3|HNRPK_HUMAN	-0.4173851	4.5301065
sp|P61981|1433G_HUMAN	-0.4158897	2.365629
sp|Q9UBV8|PEF1_HUMAN	-0.4156094	1.6960104
sp|P59998|ARPC4_HUMAN	-0.4150505	2.6576471
sp|P07858|CATB_HUMAN	-0.4135132	2.6182911
sp|Q9UHB6-4|LIMA1_HUMAN	-0.4127331	1.9647322
sp|Q16543|CDC37_HUMAN	-0.4126091	4.964395
sp|Q96I99|SUCB2_HUMAN	-0.4125748	2.923088
sp|P10301|RRAS_HUMAN	-0.4124832	1.6960104
sp|Q9UL46|PSME2_HUMAN	-0.4124184	2.2323174

sp|Q16531|DDB1_HUMAN	-0.41008	5.172756
sp|P20645|MPRD_HUMAN	-0.4096928	1.6960104
sp|Q6P2Q9|PRP8_HUMAN	-0.4095688	1.4104178
sp|P07900-2|HS90A_HUMAN	-0.4081593	4.7504272
sp|Q969X5-2|ERGI1_HUMAN	-0.4049473	1.6960104
sp|P11940-2|PABP1_HUMAN	-0.4043274	1.4104178
sp|P55084|ECHB_HUMAN	-0.4039383	3.0019732
sp|O75369-2|FLNB_HUMAN	-0.4023171	12.388624
sp|Q9P0V9-2|SEP10_HUMAN	-0.4016094	1.6997428
sp|Q9Y5S9-2|RBM8A_HUMAN	-0.4007778	1.6960104
sp|P35232|PHB_HUMAN	-0.3969307	2.8132849
sp|Q13347|EIF3I_HUMAN	-0.3962879	1.8444856
sp|O95782-2|AP2A1_HUMAN	-0.3956871	1.4278674
sp|Q9HDC9|APMAP_HUMAN	-0.3948517	3.545398
sp|Q6UWY5|OLFL1_HUMAN	-0.3925304	4.509013
sp|P54886-2|P5CS_HUMAN	-0.3915386	1.4104178
sp|P24539|AT5F1_HUMAN	-0.3915234	1.8595492
sp|P09497-2|CLCB_HUMAN	-0.390974	2.1818786
sp|P50453|SPB9_HUMAN	-0.3902874	3.342776
sp|P07686|HEXB_HUMAN	-0.3866615	2.058973
sp|O75643|U520_HUMAN	-0.3858051	1.6960104
sp|Q8WXF1|PSPC1_HUMAN	-0.3854828	2.6576471
sp|Q9H4M9|EHD1_HUMAN	-0.3849335	2.151766
sp|P13797|PLST_HUMAN	-0.3840046	4.0754967
sp|Q14764|MVP_HUMAN	-0.3835526	8.221417
sp|P28065-2|PSB9_HUMAN	-0.3822155	1.560883
sp|O75947-2|ATP5H_HUMAN	-0.3809471	2.9807727
sp|Q9BVC6|TM109_HUMAN	-0.3795624	1.6960104
sp|Q12905|ILF2_HUMAN	-0.3793354	2.923088
sp|P29218|IMPA1_HUMAN	-0.3792114	2.1404836
sp|O43491|E41L2_HUMAN	-0.3771629	2.0598862
sp|P13489|RINI_HUMAN	-0.3770618	6.988256
sp|Q9HBL0|TENS1_HUMAN	-0.3758335	4.5635147
sp|P05198|IF2A_HUMAN	-0.3755322	2.1818786
sp|P23396|RS3_HUMAN	-0.3751755	5.074809
sp|Q03252|LMNB2_HUMAN	-0.3740273	8.540238
sp|P52566|GDIR2_HUMAN	-0.3731937	3.1266599
sp|O00231-2|PSD11_HUMAN	-0.3710213	2.1818786
sp|P04066|FUCO_HUMAN	-0.3679886	1.6960104
sp|P05455|LA_HUMAN	-0.3674183	2.7472508
sp|P62993|GRB2_HUMAN	-0.3673306	2.6997027
sp|P11047|LAMC1_HUMAN	-0.3666782	5.2853217
sp|O75083|WDR1_HUMAN	-0.3666229	7.0304866
sp|P30086|PEBP1_HUMAN	-0.3639565	2.3143692
sp|P25705|ATPA_HUMAN	-0.3628597	1.4716977

sp|Q13557-12|KCC2D_HUMAN	-0.3627501	2.9807727
sp|Q15393|SF3B3_HUMAN	-0.3622494	2.6997027
sp|P49411|EFTU_HUMAN	-0.3618336	3.1015959
sp|P13639|EF2_HUMAN	-0.3603897	7.2852974
sp|P20340-2|RAB6A_HUMAN	-0.3603478	3.8337784
sp|P23634-8|AT2B4_HUMAN	-0.3595429	1.8595492
sp|P55268|LAMB2_HUMAN	-0.3582039	5.589603
sp|O95865|DDAH2_HUMAN	-0.3560753	3.0019732
sp|P05787-2|K2C8_HUMAN	-0.3543663	10.393089
sp|Q16853|AOC3_HUMAN	-0.3543568	3.1184776
sp|Q14240-2|IF4A2_HUMAN	-0.353981	1.3815327
sp|O00764-2|PDXK_HUMAN	-0.3535595	2.058973
sp|P21964-2|COMT_HUMAN	-0.351162	1.8595492
sp|P25398|RS12_HUMAN	-0.3510933	1.9951487
sp|P20618|PSB1_HUMAN	-0.3504181	3.5908275
sp|Q12931-2|TRAP1_HUMAN	-0.3503723	1.4104178
sp|P61019|RAB2A_HUMAN	-0.3489437	1.6405568
sp|P11142|HSP7C_HUMAN	-0.3476219	4.399835
sp|Q08211|DHX9_HUMAN	-0.3466606	4.0051374
sp|Q96AE4-2|FUBP1_HUMAN	-0.3462486	1.4564189
sp|Q14344|GNA13_HUMAN	-0.3460884	1.6604291
sp|Q9Y277-2|VDAC3_HUMAN	-0.3458786	3.2221727
sp|Q14258|TRI25_HUMAN	-0.3447647	2.0727112
sp|P62753|RS6_HUMAN	-0.3447189	1.6960104
sp|Q9Y2X3|NOP58_HUMAN	-0.3402672	1.4278674
sp|O75367-2|H2AY_HUMAN	-0.3401909	2.4829872
sp|Q9BXP5-2|SRRT_HUMAN	-0.3401527	1.7590232
sp|P00966|ASSY_HUMAN	-0.3384075	2.058973
sp|P50552|VASP_HUMAN	-0.3379908	2.5644996
sp|P28838-2|AMPL_HUMAN	-0.3363075	7.016219
sp|P06576|ATPB_HUMAN	-0.3360806	5.4506283
sp|Q9NZ01|TECR_HUMAN	-0.3348885	1.6604291
sp|O15127|SCAM2_HUMAN	-0.334795	1.6960104
sp|P30050|RL12_HUMAN	-0.3322735	2.1818786
sp|P46976-2|GLYG_HUMAN	-0.3315811	2.6576471
sp|P13010|XRCC5_HUMAN	-0.3313904	2.0308967
sp|P31930|QCR1_HUMAN	-0.328598	2.894491
sp|Q99623|PHB2_HUMAN	-0.3237114	1.3274423
sp|Q9UPN3|MACF1_HUMAN	-0.3236942	2.4140475
sp|Q9P258|RCC2_HUMAN	-0.3234501	1.5646582
sp|Q15717-2|ELAV1_HUMAN	-0.3229313	1.4104178
sp|Q9Y3Z3|SAMH1_HUMAN	-0.3221989	2.5080707
sp|P38919|IF4A3_HUMAN	-0.3218308	2.441399
sp|P28070|PSB4_HUMAN	-0.3215408	1.4050349
sp|P40227|TCPZ_HUMAN	-0.3204117	4.7039127

sp|P68104|EF1A1_HUMAN	-0.320406	2.044628
sp|Q10713|MPPA_HUMAN	-0.3203297	1.560883
sp|P63244|RACK1_HUMAN	-0.317709	4.276259
sp|O14950|ML12B_HUMAN	-0.3169212	2.1818786
sp|Q16181-2|SEPT7_HUMAN	-0.3166962	4.080888
sp|O43390-2|HNRPR_HUMAN	-0.3157082	1.792336
sp|Q8WUM4|PDC6I_HUMAN	-0.3155422	2.3285198
sp|Q13418|ILK_HUMAN	-0.3142109	1.7703108
sp|Q8IZ83-3|A16A1_HUMAN	-0.3106709	1.9948454
sp|Q9BXS5-2|AP1M1_HUMAN	-0.3095512	2.1818786
sp|P62820|RAB1A_HUMAN	-0.3072739	2.1818786
sp|P08648|ITA5_HUMAN	-0.3064976	1.6960104
sp|O75131|CPNE3_HUMAN	-0.3044395	2.816493
sp|P60174|TPIS_HUMAN	-0.3029728	5.362944
sp|P18124|RL7_HUMAN	-0.3021374	1.4278674
sp|Q9Y3I0|RTCB_HUMAN	-0.3020706	2.3388627
sp|P52597|HNRPF_HUMAN	-0.3012524	1.5054473
sp|P16152|CBR1_HUMAN	-0.3008747	1.6853416
sp|Q92945|FUBP2_HUMAN	-0.3008347	2.4402673
sp|Q01130-2|SRSF2_HUMAN	-0.2987366	1.4104178
sp|P07942|LAMB1_HUMAN	-0.2978287	2.5529532
sp|P12956|XRCC6_HUMAN	-0.2955608	3.8047774
sp|P53004|BIEA_HUMAN	-0.2952805	1.8444856
sp|P49354-2|FNTA_HUMAN	-0.2929668	1.3815327
sp|Q16881-2|TRXR1_HUMAN	-0.2910862	1.8077462
sp|P32969|RL9_HUMAN	-0.290823	1.6960104
sp|O95831-3|AIFM1_HUMAN	-0.2906818	3.416951
sp|Q12907|LMAN2_HUMAN	-0.2898102	1.4050349
sp|P46939-2|UTRO_HUMAN	-0.2897978	2.7574906
sp|P47897|SYQ_HUMAN	-0.2888031	1.4278674
sp|P47756-2|CAPZB_HUMAN	-0.2884712	3.481736
sp|Q9NPH2|INO1_HUMAN	-0.2878609	1.7165122
sp|Q99832|TCPH_HUMAN	-0.2873612	2.2270947
sp|Q9BSJ8-2|ESYT1_HUMAN	-0.2861328	5.222092
sp|Q99798|ACON_HUMAN	-0.2848282	3.535148
sp|Q99426|TBCB_HUMAN	-0.2843628	1.4104178
sp|P14780|MMP9_HUMAN	-0.2841682	1.827201
sp|P21953|ODBB_HUMAN	-0.2838898	1.6960104
sp|Q15084-2|PDIA6_HUMAN	-0.2802982	3.0253785
sp|Q9H4A4|AMPB_HUMAN	-0.2796021	1.6277974
sp|Q9Y383-3|LC7L2_HUMAN	-0.279562	1.4104178
sp|Q16891-2|MIC60_HUMAN	-0.2792625	1.3481187
sp|P49368|TCPG_HUMAN	-0.2792206	3.2295208
sp|Q92973-2|TNPO1_HUMAN	-0.2788982	1.5646582
sp|P48637|GSHB_HUMAN	-0.2765369	2.0598862

sp|P27797|CALR_HUMAN	-0.2748489	4.890839
sp|Q96DG6|CMBL_HUMAN	-0.2743301	1.6960104
sp|P27348|1433T_HUMAN	-0.273056	1.6046445
sp|P04843|RPN1_HUMAN	-0.2724476	3.6971807
sp|P06737-2|PYGL_HUMAN	-0.2711582	3.5053878
sp|P30153|2AAA_HUMAN	-0.2699814	3.8683267
sp|Q13308-6|PTK7_HUMAN	-0.2668228	2.1818786
sp|Q86UX7-2|URP2_HUMAN	-0.2661057	1.5071542
sp|P53621-2|COPA_HUMAN	-0.26577	1.9108402
sp|P46777|RL5_HUMAN	-0.2647247	2.1377194
sp|Q99733-2|NP1L4_HUMAN	-0.2640533	1.4278674
sp|Q9NZM1-6|MYOF_HUMAN	-0.2633896	2.368217
sp|Q9Y490|TLN1_HUMAN	-0.2632656	13.875408
sp|P16219|ACADS_HUMAN	-0.2606106	1.6997428
sp|P07195|LDHB_HUMAN	-0.2602367	3.656485
sp|P35914|HMGCL_HUMAN	-0.2598	2.3143692
sp|P35606|COPB2_HUMAN	-0.2580338	4.857963
sp|Q96QK1|VPS35_HUMAN	-0.2568321	1.7754551
sp|P26641|EF1G_HUMAN	-0.2528667	2.8438601
sp|O60749|SNX2_HUMAN	-0.2516975	3.8106754
sp|P11216|PYGB_HUMAN	-0.2508202	2.1795774
sp|Q96AG4|LRC59_HUMAN	-0.2504673	1.560883
sp|P49419-2|AL7A1_HUMAN	-0.2497311	2.4402673
sp|P27338|AOFB_HUMAN	-0.2496777	2.2752137
sp|P08758|ANXA5_HUMAN	-0.2478371	3.1785138
sp|P35998|PRS7_HUMAN	-0.2461147	1.9627187
sp|O15031|PLXB2_HUMAN	-0.2440262	1.5555226
sp|P46782|RS5_HUMAN	-0.2438355	2.6576471
sp|O75955|FLOT1_HUMAN	-0.2429009	3.0139608
sp|P14625|ENPL_HUMAN	-0.2425041	7.3195586
sp|P25789|PSA4_HUMAN	-0.23983	1.9948454
sp|P50570-2|DYN2_HUMAN	-0.2393894	2.058973
sp|P43121|MUC18_HUMAN	-0.2370834	2.56956
sp|P11215-2|ITAM_HUMAN	-0.236721	1.9951487
sp|P36543-2|VATE1_HUMAN	-0.2360687	1.4104178
sp|Q15005|SPCS2_HUMAN	-0.2357426	1.4104178
sp|P50990|TCPQ_HUMAN	-0.2351799	4.6997666
sp|Q9H2D6-2|TARA_HUMAN	-0.233633	1.4351699
sp|P48444|COPD_HUMAN	-0.230402	3.3101625
sp|P31150|GDIA_HUMAN	-0.2277927	2.5385003
sp|P00558|PGK1_HUMAN	-0.2273941	1.3400655
sp|O14974-3|MYPT1_HUMAN	-0.2272062	1.7713763
sp|O43242|PSMD3_HUMAN	-0.2263088	2.2323174
sp|P60842|IF4A1_HUMAN	-0.2251625	2.400925
sp|Q16851|UGPA_HUMAN	-0.2233162	2.096182

sp|P62241|RS8_HUMAN	-0.2231712	3.1266599
sp|P98160|PGBM_HUMAN	-0.219904	3.1189184
sp|P50502|F10A1_HUMAN	-0.2190781	1.3635377
sp|P39060-1|COIA1_HUMAN	-0.2190723	1.4278674
sp|O14818|PSA7_HUMAN	-0.2183762	2.6479273
sp|Q13409-3|DC1I2_HUMAN	-0.2165384	1.3080103
sp|P21980|TGM2_HUMAN	-0.2164497	2.795657
sp|Q99714|HCD2_HUMAN	-0.2143402	1.7703108
sp|Q12906-4|ILF3_HUMAN	-0.2131424	2.5322723
sp|P30101|PDIA3_HUMAN	-0.2131138	5.501058
sp|Q14203-3|DCTN1_HUMAN	-0.2108707	1.4050349
sp|P13667|PDIA4_HUMAN	-0.2100906	5.101818
sp|P21281|VATB2_HUMAN	-0.2098084	3.417183
sp|P46063|RECQ1_HUMAN	-0.2095966	1.8444856
sp|P00403|COX2_HUMAN	-0.2095432	1.3815327
sp|P14314-2|GLU2B_HUMAN	-0.2050037	1.5983955
sp|Q14974|IMB1_HUMAN	-0.2037125	2.2270947
sp|Q9NZ08-2|ERAP1_HUMAN	-0.2034874	1.6853416
sp|Q63ZY3-3|KANK2_HUMAN	-0.200695	1.4278674
sp|P04075|ALDOA_HUMAN	-0.2002525	5.0293546
sp|P07384|CAN1_HUMAN	-0.2002029	1.7936871
sp|P28062|PSB8_HUMAN	-0.199234	1.3815327
sp|Q8N1G4|LRC47_HUMAN	-0.1984882	2.5461748
sp|Q13185|CBX3_HUMAN	-0.1979637	1.9647322
sp|P30085|KCY_HUMAN	-0.1943026	1.4104178
sp|P06744|G6PI_HUMAN	-0.190279	3.2259803
sp|P39023|RL3_HUMAN	-0.1884728	1.5646582
sp|Q00610-2|CLH1_HUMAN	-0.1865215	3.2772517
sp|Q99497|PARK7_HUMAN	-0.183054	1.6997428
sp|Q969G5|CAVN3_HUMAN	-0.1830483	1.4104178
sp|P23526|SAHH_HUMAN	-0.1823654	2.8274305
sp|Q13126-2|MTAP_HUMAN	-0.1811123	1.4050349
sp|P16615|AT2A2_HUMAN	-0.1754093	1.7662107
sp|Q07954|LRP1_HUMAN	-0.1724243	1.596566
sp|P60983|GMFB_HUMAN	-0.1720944	1.6960104
sp|P07237|PDIA1_HUMAN	-0.1704178	7.046882
sp|P08133|ANXA6_HUMAN	-0.1658516	3.7303731
sp|Q9UBE0|SAE1_HUMAN	-0.1647902	1.4104178
sp|Q9NYU2-2|UGGG1_HUMAN	-0.164114	2.9343572
sp|P62888|RL30_HUMAN	-0.1600609	1.6960104
sp|P49913|CAMP_HUMAN	-0.1599579	1.4104178
sp|P17987|TCPA_HUMAN	-0.1546192	1.3431479
sp|P50991-2|TCPD_HUMAN	-0.1540117	1.7565529
sp|Q15029-2|U5S1_HUMAN	-0.1538458	1.7026864
sp|Q15019-2|SEPT2_HUMAN	-0.1481476	2.8046327

sp|O15145|ARPC3_HUMAN	-0.1449223	1.6960104
sp|Q16658|FSCN1_HUMAN	-0.1369305	1.3908802
sp|P21333-2|FLNA_HUMAN	-0.1366901	8.863618
sp|P47755|CAZA2_HUMAN	-0.1353779	1.9153107
sp|P55786|PSA_HUMAN	-0.1335259	1.8521893
sp|P23284|PPIB_HUMAN	-0.1321716	1.8060131
sp|P35579|MYH9_HUMAN	-0.1320419	3.2562804
sp|P52907|CAZA1_HUMAN	-0.1237755	1.515701
sp|P19971|TYPH_HUMAN	-0.0998402	1.8786486
sp|P60660-2|MYL6_HUMAN	-0.0793419	1.4017582
sp|Q9NRN5-2|OLFL3_HUMAN	0.08413506	1.4351699
sp|Q15582|BGH3_HUMAN	0.09831047	1.500785
sp|P13647|K2C5_HUMAN	0.09856606	1.647208
sp|P22105-1|TENX_HUMAN	0.14392853	4.9096303
sp|Q9Y6C2|EMIL1_HUMAN	0.1618805	2.1546397
sp|Q0ZGT2-4|NEXN_HUMAN	0.16736221	1.3481187
sp|Q13162|PRDX4_HUMAN	0.16912079	2.3047035
sp|O00754-2|MA2B1_HUMAN	0.17090034	1.9647322
sp|P35555|FBN1_HUMAN	0.18336678	4.9985876
sp|P08670|VIME_HUMAN	0.18417549	11.33357
sp|P24821-4|TENA_HUMAN	0.19094086	2.2535503
sp|Q92896-2|GSLG1_HUMAN	0.1916275	1.8444856
sp|Q07507|DERM_HUMAN	0.19601822	2.7624686
sp|P21266|GSTM3_HUMAN	0.19797134	2.044628
sp|P30041|PRDX6_HUMAN	0.20548439	3.1280005
sp|P30711|GSTT1_HUMAN	0.20690632	1.6960104
sp|P00491|PNPH_HUMAN	0.22642899	2.923088
sp|Q8TDL5|BPIB1_HUMAN	0.23704529	1.515701
sp|Q9NR12-2|PDLI7_HUMAN	0.24579048	1.827201
sp|P28331-2|NDUS1_HUMAN	0.26221657	2.3143692
sp|P11498|PYC_HUMAN	0.26953697	1.6960104
sp|Q8N2S1|LTBP4_HUMAN	0.2717495	3.5053878
sp|Q14195-2|DPYL3_HUMAN	0.27277374	4.01034
sp|P00488|F13A_HUMAN	0.2812786	2.4970746
sp|P36269-3|GGT5_HUMAN	0.28225994	2.5644996
sp|P22352|GPX3_HUMAN	0.28427696	1.6960104
sp|P08294|SODE_HUMAN	0.28476334	1.3080103
sp|P06132|DCUP_HUMAN	0.29065323	1.5646582
sp|Q8NBS9|TXND5_HUMAN	0.29108047	5.450629
sp|P51911|CNN1_HUMAN	0.32061005	3.9403465
sp|Q9UMS6-2|SYNP2_HUMAN	0.32098007	2.7472508
sp|Q5TDH0-3|DDI2_HUMAN	0.33494186	1.4104178
sp|Q07065|CKAP4_HUMAN	0.3459549	9.607684
sp|P67936-2|TPM4_HUMAN	0.3604641	1.6960104
sp|P04264|K2C1_HUMAN	0.36687088	10.259592

sp|Q53GG5-2|PDLI3_HUMAN	0.38015175	1.3815327
sp|Q9UBX5|FBLN5_HUMAN	0.38614464	2.12598
sp|P01011|AACT_HUMAN	0.40455627	7.086946
sp|P23219-2|PGH1_HUMAN	0.40616035	2.1818786
sp|Q15102|PA1B3_HUMAN	0.41086197	1.4104178
sp|O60831|PRAF2_HUMAN	0.41267014	1.6960104
sp|Q8WU39|MZB1_HUMAN	0.41846085	2.5644996
sp|P35527|K1C9_HUMAN	0.42078972	4.881816
sp|P02765|FETUA_HUMAN	0.4324665	2.0727112
sp|P04259|K2C6B_HUMAN	0.43745995	1.9647322
sp|Q9Y315|DEOC_HUMAN	0.44018555	1.9647322
sp|P07951|TPM2_HUMAN	0.45230103	1.827201
sp|P00748|FA12_HUMAN	0.45306587	1.6960104
sp|P98095-2|FBLN2_HUMAN	0.45435333	4.0218377
sp|P23946|CMA1_HUMAN	0.45511246	2.3143692
sp|A1L4H1|SRCRL_HUMAN	0.4587736	1.6960104
sp|P00736|C1R_HUMAN	0.48395824	1.6960104
sp|P13611|CSPG2_HUMAN	0.48461914	5.417897
sp|P24844|MYL9_HUMAN	0.4852867	1.6960104
sp|Q00796|DHSO_HUMAN	0.4931755	1.6960104
sp|P11171-7|41_HUMAN	0.5433693	2.1818786
sp|Q14192|FHL2_HUMAN	0.55152893	2.1818786
sp|P13716-2|HEM2_HUMAN	0.5685692	3.5908275
sp|P02792|FRIL_HUMAN	0.57741165	3.3668416
sp|Q16647|PTGIS_HUMAN	0.5784168	2.6576471
sp|P50454|SERPH_HUMAN	0.588562	3.8337784
sp|P09493-8|TPM1_HUMAN	0.5894642	3.5908275
sp|Q8TAQ2-2|SMRC2_HUMAN	0.6193638	1.6960104
sp|Q01995|TAGL_HUMAN	0.62096786	7.8905663
sp|P04196|HRG_HUMAN	0.62278175	3.9658737
sp|P23142|FBLN1_HUMAN	0.6278534	3.1266599
sp|P51888|PRELP_HUMAN	0.6377964	5.869831
sp|P01009|A1AT_HUMAN	0.6476307	9.893875
sp|Q12805-2|FBLN3_HUMAN	0.65185165	5.727641
sp|P05156|CFAI_HUMAN	0.65800095	3.1208909
sp|P02753|RET4_HUMAN	0.67069435	1.6960104
sp|P09493-9|TPM1_HUMAN	0.6779232	1.6960104
sp|P04004|VTNC_HUMAN	0.67943764	4.051346
sp|P01019|ANGT_HUMAN	0.687973	1.3815327
sp|P02743|SAMP_HUMAN	0.7080326	4.509013
sp|P07357|CO8A_HUMAN	0.7080765	2.6576471
sp|P02649|APOE_HUMAN	0.7267418	4.578167
sp|P03952|KLKB1_HUMAN	0.7280731	2.1818786
sp|O14558|HSPB6_HUMAN	0.7299051	1.8444856
sp|P17661|DESM_HUMAN	0.78365135	14.4774685

sp|P00450|CERU_HUMAN	0.7925682	10.210825
sp|P16157-21|ANK1_HUMAN	0.79299164	4.051346
sp|Q9NYL4|FKB11_HUMAN	0.79729843	1.6960104
sp|P06681-3|CO2_HUMAN	0.7995491	2.6576471
sp|Q96PD5-2|PGRP2_HUMAN	0.8237438	1.7590232
sp|P04217|A1BG_HUMAN	0.8264046	4.964395
sp|P30043|BLVRB_HUMAN	0.83176804	2.1818786
sp|P07585|PGS2_HUMAN	0.8396492	5.417897
sp|P02452|CO1A1_HUMAN	0.87186813	2.058973
sp|Q96D15|RCN3_HUMAN	0.87590694	1.6960104
sp|P23456|Trypsin	0.88295174	3.5908275
sp|P02766|TTHY_HUMAN	0.88648224	2.1818786
sp|P19827|ITIH1_HUMAN	0.88695717	4.405163
sp|P00734|THRB_HUMAN	0.8885002	5.523198
sp|P11277-2|SPTB1_HUMAN	0.9027176	6.3204336
sp|P08123|CO1A2_HUMAN	0.91880894	2.6576471
sp|P07996|TSP1_HUMAN	0.9202385	1.6960104
sp|P10643|CO7_HUMAN	0.9302101	4.3100786
sp|P13671|CO6_HUMAN	0.93118286	1.6960104
sp|P02790|HEMO_HUMAN	0.9642296	7.831889
sp|P43652|AFAM_HUMAN	0.9649868	4.509013
sp|P20774|MIME_HUMAN	0.976387	4.051346
sp|P02748|CO9_HUMAN	0.98711014	7.2183566
sp|P00751|CFAB_HUMAN	0.9912491	10.583587
sp|P07738|PMGE_HUMAN	0.99913025	2.1818786
sp|P02774-3|VTDB_HUMAN	1.0003338	9.004438
sp|P51884|LUM_HUMAN	1.003931	8.112776
sp|P25311|ZA2G_HUMAN	1.008606	4.051346
sp|P02549-2|SPTA1_HUMAN	1.0203056	8.471928
sp|P02760|AMBP_HUMAN	1.0324078	3.5908275
sp|P04003|C4BPA_HUMAN	1.077776	5.869831
sp|P07451|CAH3_HUMAN	1.0798988	2.058973
sp|P01008|ANT3_HUMAN	1.094841	8.112776
sp|P36955|PEDF_HUMAN	1.1082611	4.051346
sp|P02730|B3AT_HUMAN	1.1383438	6.521583
sp|P0DOX7|IGK_HUMAN	1.1580391	2.1818786
sp|Q9BXN1|ASPN_HUMAN	1.1677647	2.6576471
sp|P01042-2|KNG1_HUMAN	1.196188	7.2183566
sp|P27169|PON1_HUMAN	1.205225	1.6960104
sp|P29622|KAIN_HUMAN	1.2494411	2.6576471
sp|P08603|CFAH_HUMAN	1.2791233	14.875408
sp|P01859|IGHG2_HUMAN	1.2951946	4.509013
sp|P02749|APOH_HUMAN	1.3094406	3.5908275
sp|P04114|APOB_HUMAN	1.3231277	14.238586
sp|P01031|CO5_HUMAN	1.3237534	7.3945704

sp|P01023|A2MG_HUMAN	1.3319321	15.95459
sp|P32119|PRDX2_HUMAN	1.3356304	6.7698927
sp|P19823|ITIH2_HUMAN	1.3398952	5.417897
sp|P00918|CAH2_HUMAN	1.3896675	4.509013
sp|P10909-5|CLUS_HUMAN	1.3995953	4.964395
sp|P0DOX2|IGA2_HUMAN	1.4191971	1.6960104
sp|P05546|HEP2_HUMAN	1.4532719	2.6576471
sp|P15088|CBPA3_HUMAN	1.5031815	2.1818786
sp|P06727|APOA4_HUMAN	1.5233841	5.98738
sp|P00915|CAH1_HUMAN	1.6297035	7.665951
sp|P0DOX8|IGL1_HUMAN	1.655674	2.1818786
sp|P02042|HBD_HUMAN	1.6788445	4.051346
sp|P0DOY3|IGLC3_HUMAN	1.6990528	2.1818786
sp|P0DOX5|IGG1_HUMAN	1.7674084	4.964395
sp|P02652-2|APOA2_HUMAN	1.7806282	3.1266599
sp|P01876|IGHA1_HUMAN	1.8204575	4.509013
sp|P01871-2|IGHM_HUMAN	1.8457603	2.1818786
sp|P00747|PLMN_HUMAN	1.8601665	6.7698927
sp|P01861|IGHG4_HUMAN	1.9844398	3.5908275
sp|P68871|HBB_HUMAN	2.0491219	4.051346
sp|P02671|FIBA_HUMAN	2.114399	11.667864
sp|P02675|FIBB_HUMAN	2.1256409	10.337908
sp|P02647|APOA1_HUMAN	2.1534443	13.420564
sp|P02679-2|FIBG_HUMAN	2.1585693	8.032379
sp|P69905|HBA_HUMAN	2.2797031	4.051346
sp|Q9BW04|SARG_HUMAN	-2.3990269	1.1932944
sp|O00757|F16P2_HUMAN	-2.229309	1.1932944
sp|P04229|2B11_HUMAN	-1.8554554	0.656254
sp|Q16777|H2A2C_HUMAN	-1.4719543	1.1932944
sp|P33151|CADH5_HUMAN	-1.4601202	1.1932944
sp|P28330|ACADL_HUMAN	-1.3883457	1.1932944
sp|P26440|IVD_HUMAN	-1.2620544	0.35795313
sp|P08263|GSTA1_HUMAN	-1.2538662	0
sp|P08246|ELNE_HUMAN	-1.2312889	1.1932944
sp|Q13813-3|SPTN1_HUMAN	-1.2164555	0.45033538
sp|Q93077|H2A1C_HUMAN	-1.2126312	0.7827403
sp|P51606-2|RENBP_HUMAN	-1.1966057	1.1932944
sp|Q9H2U2-3|IPYR2_HUMAN	-1.1861115	0.656254
sp|P38159|RBMX_HUMAN	-1.1740837	0.656254
sp|Q9Y4G6|TLN2_HUMAN	-1.1711655	0.656254
sp|P37235|HPCL1_HUMAN	-1.168849	0.656254
sp|P12236|ADT3_HUMAN	-1.1578922	1.1932944
sp|P08473|NEP_HUMAN	-1.1403656	0.5204253
sp|Q6DN03|H2B2C_HUMAN	-1.1010475	1.1932944
sp|P12830|CADH1_HUMAN	-1.0985146	0.7827403

sp|P10412|H14_HUMAN	-1.0641441	1.1932944
sp|P16422|EPCAM_HUMAN	-1.0591497	1.1932944
sp|P13686|PPA5_HUMAN	-1.058115	1.2773042
sp|Q29963|1C06_HUMAN	-1.0549297	0.656254
sp|Q13976|KGP1_HUMAN	-1.0517998	0.656254
sp|O43795-2|MYO1B_HUMAN	-1.0262566	1.1932944
sp|P20702|ITAX_HUMAN	-1.0191116	0.656254
sp|Q01518|CAP1_HUMAN	-1.0175076	0.656254
sp|Q08209-5|PP2BA_HUMAN	-0.9845486	0
sp|A6NMY6|AXA2L_HUMAN	-0.9678974	0.656254
sp|P68371|TBB4B_HUMAN	-0.9622536	1.1932944
sp|Q8NBQ5|DHB11_HUMAN	-0.9567337	1.1932944
sp|Q03113|GNA12_HUMAN	-0.9566402	0.656254
sp|P63218|GBG5_HUMAN	-0.9474373	1.1932944
sp|Q9UDY2-3|ZO2_HUMAN	-0.9432335	0.7827403
sp|P62834|RAP1A_HUMAN	-0.9392176	0.656254
sp|O95837|GNA14_HUMAN	-0.9356175	0.656254
sp|P00167-2|CYB5_HUMAN	-0.9241276	0.7588735
sp|P13760|2B14_HUMAN	-0.9157887	0.35795313
sp|O00592-2|PODXL_HUMAN	-0.9061813	1.1932944
sp|P16403|H12_HUMAN	-0.8983154	0.656254
sp|Q86TX2|ACOT1_HUMAN	-0.8947792	0.656254
sp|P63167|DYL1_HUMAN	-0.8944588	0.656254
sp|Q03135-2|CAV1_HUMAN	-0.8930092	0.656254
sp|P15170-2|ERF3A_HUMAN	-0.8843517	1.1932944
sp|Q9NUB1-2|ACS2L_HUMAN	-0.8756123	1.1932944
sp|Q9UHN6-2|CEIP2_HUMAN	-0.8734646	1.1932944
sp|Q9NPY3|C1QR1_HUMAN	-0.8456869	1.1932944
sp|P13761|2B17_HUMAN	-0.8415566	0
sp|P06396-2|GELS_HUMAN	-0.8409748	0.656254
sp|P62263|RS14_HUMAN	-0.8321686	0.7061832
sp|Q14956-2|GPNMB_HUMAN	-0.8242798	1.1932944
sp|P61956-2|SUMO2_HUMAN	-0.8232918	0.656254
sp|P61916-2|NPC2_HUMAN	-0.8198433	1.1932944
sp|P02462|CO4A1_HUMAN	-0.8111248	0.35795313
sp|Q15185-3|TEBP_HUMAN	-0.8107376	0.19149946
sp|P28799-3|GRN_HUMAN	-0.7921963	0.5204253
sp|Q7L2H7|EIF3M_HUMAN	-0.7911072	0.656254
sp|Q9Y394-2|DHRS7_HUMAN	-0.7859459	0.7588735
sp|P04839|CY24B_HUMAN	-0.782939	0
sp|P27635|RL10_HUMAN	-0.7805595	0.656254
sp|P12235|ADT1_HUMAN	-0.7774696	0.656254
sp|P16104|H2AX_HUMAN	-0.7733955	0.656254
sp|Q15286|RAB35_HUMAN	-0.7589693	0.656254
sp|Q05315|LEG10_HUMAN	-0.7585125	1.1932944

sp|O94811|TPPP_HUMAN	-0.7580013	1.1932944
sp|P61224|RAP1B_HUMAN	-0.7505608	0.656254
sp|Q9NPJ3|ACO13_HUMAN	-0.7464733	1.1932944
sp|O95716|RAB3D_HUMAN	-0.745944	0.656254
sp|P61586|RHOA_HUMAN	-0.7441158	1.1932944
sp|Q9HD89|RETN_HUMAN	-0.7410297	1.1932944
sp|P62745|RHOB_HUMAN	-0.734437	0.656254
sp|Q15056-2|IF4H_HUMAN	-0.7316876	1.1932944
sp|O76041-2|NEBL_HUMAN	-0.7254105	0.656254
sp|Q12846|STX4_HUMAN	-0.7253094	0.656254
sp|P22894|MMP8_HUMAN	-0.7251968	0
sp|O00712-4|NFIB_HUMAN	-0.7243271	0.656254
sp|Q01955|CO4A3_HUMAN	-0.7185288	1.1932944
sp|P11387|TOP1_HUMAN	-0.7177906	1.1932944
sp|Q9NVJ2|ARL8B_HUMAN	-0.7166348	1.1932944
sp|P08962-2|CD63_HUMAN	-0.7019539	1.1932944
sp|P15559-2|NQO1_HUMAN	-0.6899967	1.1932944
sp|Q96FV2-2|SCRN2_HUMAN	-0.6884117	1.1932944
sp|P42167|LAP2B_HUMAN	-0.6855087	1.1932944
sp|O95197-2|RTN3_HUMAN	-0.6790352	1.1932944
sp|P16930|FAAA_HUMAN	-0.6746769	0.656254
sp|Q9UBR2|CATZ_HUMAN	-0.6736412	1.1932944
sp|P52788-2|SPSY_HUMAN	-0.6457806	1.1932944
sp|Q13283|G3BP1_HUMAN	-0.6445217	1.1932944
sp|Q92522|H1X_HUMAN	-0.6408482	1.1932944
sp|P61601|NCALD_HUMAN	-0.6402178	0.656254
sp|Q13813-2|SPTN1_HUMAN	-0.6209335	1.1932944
sp|Q15637-2|SF01_HUMAN	-0.6205444	1.1932944
sp|P31946-2|1433B_HUMAN	-0.6197014	0.656254
sp|Q32MZ4-3|LRRF1_HUMAN	-0.6183491	1.1932944
sp|P31153|METK2_HUMAN	-0.6175098	0.656254
sp|P48509|CD151_HUMAN	-0.6122685	1.1932944
sp|P54108-2|CRIS3_HUMAN	-0.6093102	0.656254
sp|P28676|GRAN_HUMAN	-0.595995	1.1932944
sp|Q9H8L6|MMRN2_HUMAN	-0.5947304	1.1932944
sp|P10619-2|PPGB_HUMAN	-0.5943699	1.1932944
sp|Q53GQ0|DHB12_HUMAN	-0.5911293	0.35795313
sp|P18077|RL35A_HUMAN	-0.5818653	0.7588735
sp|O60504-2|VINEX_HUMAN	-0.5816498	1.1791906
sp|Q5SSJ5-2|HP1B3_HUMAN	-0.5810852	1.0485198
sp|P01892|1A02_HUMAN	-0.5765915	0.7061832
sp|Q07075|AMPE_HUMAN	-0.575882	0.40256184
sp|Q8NBF2-2|NHLC2_HUMAN	-0.5754433	1.1932944
sp|P07948-2|LYN_HUMAN	-0.5676804	1.1932944
sp|Q6YN16|HSDL2_HUMAN	-0.5666533	0.5204253

sp|A5A3E0|POTEF_HUMAN	-0.5655975	0.656254
sp|Q16822|PCKGM_HUMAN	-0.5609779	1.1932944
sp|P06703|S10A6_HUMAN	-0.5554199	1.1932944
sp|P51153|RAB13_HUMAN	-0.5526638	0.656254
sp|Q7Z4I7-3|LIMS2_HUMAN	-0.5521679	1.1932944
sp|Q13404|UB2V1_HUMAN	-0.5459404	0.35795313
sp|Q13938-4|CAYP1_HUMAN	-0.5442753	0
sp|P26368-2|U2AF2_HUMAN	-0.5435963	1.1932944
sp|P31948|STIP1_HUMAN	-0.5432472	0.656254
sp|Q9HB71|CYBP_HUMAN	-0.5415335	1.1932944
sp|P01111|RASN_HUMAN	-0.529398	0.656254
sp|Q9NX63|MIC19_HUMAN	-0.5273209	1.1932944
sp|P55327-3|TPD52_HUMAN	-0.5262871	1.1932944
sp|O76003|GLRX3_HUMAN	-0.5217857	0.656254
sp|Q9NYF8-2|BCLF1_HUMAN	-0.5206013	1.1932944
sp|Q8NF91-4|SYNE1_HUMAN	-0.5163097	1.1932944
sp|P02741|CRP_HUMAN	-0.5151634	1.1932944
sp|Q15181|IPYR_HUMAN	-0.5111656	0.6298893
sp|Q9H2G2-2|SLK_HUMAN	-0.5098763	1.1932944
sp|P51991-2|ROA3_HUMAN	-0.5060215	0.656254
sp|P01920|DQB1_HUMAN	-0.5044251	1.1932944
sp|P42126-2|ECI1_HUMAN	-0.5030556	1.1932944
sp|P04222|1C03_HUMAN	-0.5027523	0.656254
sp|P29972-2|AQP1_HUMAN	-0.5013275	1.1932944
sp|P23786|CPT2_HUMAN	-0.5007935	0.6298893
sp|P11234-2|RALB_HUMAN	-0.4998837	0.656254
sp|Q9NTX5-6|ECHD1_HUMAN	-0.4980316	1.0370445
sp|P31946|1433B_HUMAN	-0.4904842	0.656254
sp|Q9NNW7|TRXR2_HUMAN	-0.489727	0.6298893
sp|E9PAV3|NACAM_HUMAN	-0.4771118	1.1932944
sp|Q6ZVM7-3|TM1L2_HUMAN	-0.4725647	1.1932944
sp|P08621-3|RU17_HUMAN	-0.4671021	0.84879977
sp|Q04837|SSBP_HUMAN	-0.4654331	1.1932944
sp|Q9UH99-3|SUN2_HUMAN	-0.4631825	0.7588735
sp|P62854|RS26_HUMAN	-0.4628353	1.1932944
sp|P62244|RS15A_HUMAN	-0.4622421	1.1932944
sp|Q8TD06|AGR3_HUMAN	-0.4597416	0.7827403
sp|P08236-2|BGLR_HUMAN	-0.4553299	0.7588735
sp|Q13435|SF3B2_HUMAN	-0.4550533	1.1932944
sp|Q71UM5|RS27L_HUMAN	-0.4548531	1.1932944
sp|Q9ULZ3-2|ASC_HUMAN	-0.4542522	0.7827403
sp|Q6P4A8|PLBL1_HUMAN	-0.4541788	0.7588735
sp|P35268|RL22_HUMAN	-0.4510155	0.7061832
sp|P84243|H33_HUMAN	-0.4500084	0.656254
sp|Q09028-3|RBBP4_HUMAN	-0.448534	0.656254

sp|P48047|ATPO_HUMAN	-0.4480419	0.56808305
sp|Q5TFE4|NT5D1_HUMAN	-0.4473324	1.1932944
sp|Q86W92-2|LIPB1_HUMAN	-0.4465818	1.1932944
sp|P04440|DPB1_HUMAN	-0.4457512	1.1932944
sp|P29466-2|CASP1_HUMAN	-0.4420166	0.656254
sp|Q96HD1|CREL1_HUMAN	-0.4332867	0.7827403
sp|Q9UQ80|PA2G4_HUMAN	-0.4320259	0.656254
sp|P61160|ARP2_HUMAN	-0.4308262	1.1932944
sp|P62826|RAN_HUMAN	-0.4283295	0.87291557
sp|Q9UNE7-2|CHIP_HUMAN	-0.424551	1.1932944
sp|Q96JB5-4|CK5P3_HUMAN	-0.4224472	0.7061832
sp|P60033|CD81_HUMAN	-0.4212418	1.1932944
sp|Q9Y3A3-3|PHOCN_HUMAN	-0.4208012	0.45033538
sp|Q9UBS4|DJB11_HUMAN	-0.4152489	1.1932944
sp|P61313|RL15_HUMAN	-0.4148121	0.19149946
sp|Q7L1Q6-2|BZW1_HUMAN	-0.4144335	1.0301651
sp|P20160|CAP7_HUMAN	-0.4134865	1.1505735
sp|Q08170|SRSF4_HUMAN	-0.4131107	0.656254
sp|P35754|GLRX1_HUMAN	-0.4123421	0.95332193
sp|P23193-2|TCEA1_HUMAN	-0.4121523	0.35795313
sp|Q14697|GANAB_HUMAN	-0.4115458	1.1932944
sp|P31943|HNRH1_HUMAN	-0.4083214	0.6509351
sp|Q96TC7|RMD3_HUMAN	-0.4072056	0.5204253
sp|P62910|RL32_HUMAN	-0.4066734	1.1932944
sp|Q9NVD7|PARVA_HUMAN	-0.4061165	0.19149946
sp|P29400-2|CO4A5_HUMAN	-0.404397	0.656254
sp|Q9BRA2|TXD17_HUMAN	-0.40028	1.0485198
sp|Q13177|PAK2_HUMAN	-0.3999119	1.1932944
sp|Q8N684-3|CPSF7_HUMAN	-0.3988466	0.6070219
sp|P29992|GNA11_HUMAN	-0.396656	1.1932944
sp|Q13200|PSMD2_HUMAN	-0.3952713	0.7061832
sp|P62995-3|TRA2B_HUMAN	-0.3950386	1.1932944
sp|P06239-3|LCK_HUMAN	-0.3925095	0.656254
sp|Q9HCC0|MCCB_HUMAN	-0.3919983	0.6509351
sp|Q9NUJ1-3|ABHDA_HUMAN	-0.3918152	0
sp|P12081-4|SYHC_HUMAN	-0.391386	0.91601294
sp|Q13838-2|DX39B_HUMAN	-0.3909435	0.6298893
sp|Q9BQE3|TBA1C_HUMAN	-0.3900061	0.656254
sp|P51553-2|IDH3G_HUMAN	-0.3868084	1.1932944
sp|P40306|PSB10_HUMAN	-0.3864136	1.0485198
sp|Q9UBW8|CSN7A_HUMAN	-0.3834124	1.1505735
sp|Q96IJ6-2|GMPPA_HUMAN	-0.3825779	0.7827403
sp|Q8NC51-3|PAIRB_HUMAN	-0.381237	1.1932944
sp|Q06136|KDSR_HUMAN	-0.3801785	1.1932944
sp|P63000-2|RAC1_HUMAN	-0.3797512	0.91601294

sp|P09601|HMOX1_HUMAN	-0.3792114	0.8983557
sp|O14744|ANM5_HUMAN	-0.3790913	1.1932944
sp|Q86Y82|STX12_HUMAN	-0.3778172	1.1791906
sp|P48163-2|MAOX_HUMAN	-0.3768244	0.5204253
sp|P63220|RS21_HUMAN	-0.3711052	1.1932944
sp|Q9Y371-2|SHLB1_HUMAN	-0.3703404	0.656254
sp|P61457|PHS_HUMAN	-0.3692532	0.7827403
sp|P07437|TBB5_HUMAN	-0.3680992	1.0485198
sp|P62495-2|ERF1_HUMAN	-0.3666143	0.7061832
sp|O00186|STXB3_HUMAN	-0.3632689	0.45033538
sp|Q03154-4|ACY1_HUMAN	-0.3631201	0.5204253
sp|P11717|MPRI_HUMAN	-0.362299	1.1932944
sp|Q13642-1|FHL1_HUMAN	-0.3599415	0.656254
sp|P62316-2|SMD2_HUMAN	-0.3584671	1.2773042
sp|P30838|AL3A1_HUMAN	-0.3564644	0.7827403
sp|P30419-2|NMT1_HUMAN	-0.3558598	0.656254
sp|P36871|PGM1_HUMAN	-0.3540859	0.7827403
sp|Q9NSE4|SYIM_HUMAN	-0.3531151	0.95332193
sp|Q14914-2|PTGR1_HUMAN	-0.3526859	0.6298893
sp|Q9C0B1|FTO_HUMAN	-0.3490791	0
sp|O60784-3|TOM1_HUMAN	-0.3485413	0.656254
sp|P50452|SPB8_HUMAN	-0.347641	1.1932944
sp|P60228|EIF3E_HUMAN	-0.3466415	0.6298893
sp|P36551|HEM6_HUMAN	-0.3464699	0.45033538
sp|P11217|PYGM_HUMAN	-0.3462925	0.656254
sp|P08134|RHOC_HUMAN	-0.346056	0.19149946
sp|Q9Y305-4|ACOT9_HUMAN	-0.3446331	0.7827403
sp|O75608-2|LYPA1_HUMAN	-0.344326	1.1932944
sp|P17213|BPI_HUMAN	-0.3441563	1.2095301
sp|P62136|PP1A_HUMAN	-0.3432388	1.1505735
sp|Q9NZB2-6|F120A_HUMAN	-0.3428078	1.1932944
sp|P16070-7|CD44_HUMAN	-0.3409843	0.91601294
sp|O00483|NDUA4_HUMAN	-0.3396587	1.1932944
sp|Q9Y639-4|NPTN_HUMAN	-0.3393498	1.1932944
sp|Q9Y2Q5|LTOR2_HUMAN	-0.3389454	0.7827403
sp|Q86U42-2|PABP2_HUMAN	-0.3382225	0.656254
sp|Q14894|CRYM_HUMAN	-0.335825	0
sp|O14936-2|CSKP_HUMAN	-0.3344803	0.65625405
sp|P53634|CATC_HUMAN	-0.3338261	0.5204253
sp|Q99729-3|ROAA_HUMAN	-0.3330402	0.7957244
sp|P60866-2|RS20_HUMAN	-0.3329086	1.1932944
sp|P12955|PEPD_HUMAN	-0.3319321	0.34323573
sp|Q32P44|EMAL3_HUMAN	-0.3312359	1.2773042
sp|Q14152|EIF3A_HUMAN	-0.3308945	0.35795313
sp|P53041|PPP5_HUMAN	-0.3297539	0.5204253

sp|Q96BM9|ARL8A_HUMAN	-0.3293686	0.656254
sp|Q08257|QOR_HUMAN	-0.3283958	0.9847622
sp|P48059-3|LIMS1_HUMAN	-0.3279991	0.656254
sp|Q14498-2|RBM39_HUMAN	-0.3271027	0.14672586
sp|P63261|ACTG_HUMAN	-0.3267708	0.656254
sp|P28072|PSB6_HUMAN	-0.3264465	0.91601294
sp|P52789|HXK2_HUMAN	-0.3239899	0.656254
sp|P08754|GNAI3_HUMAN	-0.3234291	1.0485198
sp|P78406|RAE1L_HUMAN	-0.3229294	1.1932944
sp|Q06278|AOXA_HUMAN	-0.3226738	0.7827403
sp|P10599-2|THIO_HUMAN	-0.3211651	0.40256184
sp|P09917|LOX5_HUMAN	-0.3209229	1.1791906
sp|Q9BTE1|DCTN5_HUMAN	-0.3190403	1.1932944
sp|Q6NVY1|HIBCH_HUMAN	-0.3181191	0.5111962
sp|O94905|ERLN2_HUMAN	-0.3164482	1.1932944
sp|P63241|IF5A1_HUMAN	-0.3137636	0.656254
sp|P14923|PLAK_HUMAN	-0.3135834	1.0485198
sp|O14745|NHRF1_HUMAN	-0.3117981	0.5204253
sp|Q15149-3|PLEC_HUMAN	-0.3114891	0.656254
sp|P63151-2|2ABA_HUMAN	-0.3094063	1.1932944
sp|Q99715-4|COCA1_HUMAN	-0.3079319	0.5204253
sp|O43143|DHX15_HUMAN	-0.3077774	1.0634323
sp|Q9BWS9-3|CHID1_HUMAN	-0.3077164	0.656254
sp|P80188|NGAL_HUMAN	-0.3072796	1.0370445
sp|Q9UKK9|NUDT5_HUMAN	-0.3037071	0.7827403
sp|Q9H3N1|TMX1_HUMAN	-0.3032684	0.7061832
sp|Q9NR56-2|MBNL1_HUMAN	-0.3031731	1.1932944
sp|Q9UHQ9|NB5R1_HUMAN	-0.3019428	1.0485198
sp|Q96S97|MYADM_HUMAN	-0.300745	1.1932944
sp|P52790|HXK3_HUMAN	-0.3002071	1.1791906
sp|P49458|SRP09_HUMAN	-0.2992039	0.7827403
sp|P30049|ATPD_HUMAN	-0.2985115	1.1932944
sp|O75828|CBR3_HUMAN	-0.2984085	1.1932944
sp|Q9UGI8-2|TES_HUMAN	-0.2969837	0.91072154
sp|P42566|EPS15_HUMAN	-0.2958756	1.1932944
sp|Q5JPE7-2|NOMO2_HUMAN	-0.2958202	0.5204253
sp|Q9P2J5-2|SYLC_HUMAN	-0.2957869	0.19149946
sp|P30566|PUR8_HUMAN	-0.2956886	0.656254
sp|Q9H0W9-2|CK054_HUMAN	-0.2954874	1.3006523
sp|Q14011-2|CIRBP_HUMAN	-0.2936173	1.0301651
sp|O60832|DKC1_HUMAN	-0.2931099	1.1932944
sp|Q9BW30|TPPP3_HUMAN	-0.2912846	0.9533461
sp|O14786|NRP1_HUMAN	-0.2911253	0.7827403
sp|Q6IAA8|LTOR1_HUMAN	-0.2906761	1.1932944
sp|Q8IV08|PLD3_HUMAN	-0.2906113	0.5204253

sp|P78417-3|GSTO1_HUMAN	-0.2888222	0.7827403
sp|P61006|RAB8A_HUMAN	-0.2880087	1.1932944
sp|Q9HAV0|GBB4_HUMAN	-0.2850351	0.2178309
sp|Q96C86|DCPS_HUMAN	-0.2837124	0.5204253
sp|Q15366-2|PCBP2_HUMAN	-0.2836685	0.91601294
sp|Q7LG56-6|RIR2B_HUMAN	-0.2834005	0.7827403
sp|P63010-2|AP2B1_HUMAN	-0.283186	1.214352
sp|P30084|ECHM_HUMAN	-0.2814617	1.3006523
sp|Q9H4G4|GAPR1_HUMAN	-0.2805405	1.1505735
sp|Q8NHP8|PLBL2_HUMAN	-0.2784824	0.19149946
sp|Q96C23|GALM_HUMAN	-0.2784004	0.5204253
sp|A0AVT1|UBA6_HUMAN	-0.2769051	1.2941489
sp|O43684-2|BUB3_HUMAN	-0.2765408	1.1932944
sp|P06753-2|TPM3_HUMAN	-0.274292	0.7827403
sp|O14773|TPP1_HUMAN	-0.2731648	0.7588735
sp|P04424-2|ARLY_HUMAN	-0.2724819	0.74639726
sp|Q8TD55|PKHO2_HUMAN	-0.2709131	0
sp|P09417-2|DHPR_HUMAN	-0.2708931	0.19149946
sp|P54819-2|KAD2_HUMAN	-0.2694664	0.35795313
sp|Q9H299|SH3L3_HUMAN	-0.2691193	1.1932944
sp|Q13247-3|SRSF6_HUMAN	-0.2689304	0.7827403
sp|P13284|GILT_HUMAN	-0.2686806	0.91601294
sp|Q96RQ3|MCCA_HUMAN	-0.267376	1.2095301
sp|P26373|RL13_HUMAN	-0.2673264	0.8983557
sp|Q96BW5-2|PTER_HUMAN	-0.2657356	0
sp|Q9Y376|CAB39_HUMAN	-0.2656651	1.0301651
sp|Q9NZL9|MAT2B_HUMAN	-0.2646008	0.6298893
sp|P51688|SPHM_HUMAN	-0.2639923	1.2095301
sp|Q9NZU5-2|LMCD1_HUMAN	-0.2629719	1.0475321
sp|P61353|RL27_HUMAN	-0.2622356	1.1932944
sp|P36873-2|PP1G_HUMAN	-0.2622299	1.1932944
sp|P62714|PP2AB_HUMAN	-0.2622109	0.656254
sp|O43747-2|AP1G1_HUMAN	-0.260685	0.8983557
sp|Q7Z7H5-3|TMED4_HUMAN	-0.2591667	1.1932944
sp|P62424|RL7A_HUMAN	-0.2575016	1.0634323
sp|P17612|KAPCA_HUMAN	-0.2568932	0.8983557
sp|O95833|CLIC3_HUMAN	-0.2566814	0.1558116
sp|Q9BZF9-2|UACA_HUMAN	-0.2563515	0.45033538
sp|Q9NSD9|SYFB_HUMAN	-0.2562103	0.8983557
sp|Q96CW1-2|AP2M1_HUMAN	-0.2555294	0.8983557
sp|P61163|ACTZ_HUMAN	-0.2536926	0.8983557
sp|Q9NP72|RAB18_HUMAN	-0.2536526	0.8983557
sp|P62942|FKB1A_HUMAN	-0.2528687	0.656254
sp|O15372|EIF3H_HUMAN	-0.252347	0.35795313
sp|P21912|SDHB_HUMAN	-0.2523346	0.7061832

sp|O15260-2|SURF4_HUMAN	-0.2516632	0.45033538
sp|Q13263|TIF1B_HUMAN	-0.2512665	1.2109947
sp|P61758|PFD3_HUMAN	-0.2499809	0.7827403
sp|P47985|UCRI_HUMAN	-0.2471008	0.7061832
sp|Q9NQC3|RTN4_HUMAN	-0.2470551	1.2095301
sp|Q02952-2|AKA12_HUMAN	-0.2460671	0
sp|P12004|PCNA_HUMAN	-0.2456703	1.1932944
sp|P61225|RAP2B_HUMAN	-0.2455263	1.1932944
sp|P0C0S5|H2AZ_HUMAN	-0.244442	0.656254
sp|Q9UNM6-2|PSD13_HUMAN	-0.2443142	1.0634323
sp|P10644|KAP0_HUMAN	-0.2441559	0.6070219
sp|P53396-2|ACLY_HUMAN	-0.2430153	0.99757636
sp|P42226|STAT6_HUMAN	-0.2428532	0.656254
sp|P62266|RS23_HUMAN	-0.2417908	0.65625405
sp|P23368|MAOM_HUMAN	-0.240696	0.93112767
sp|Q96C19|EFHD2_HUMAN	-0.2406769	1.1505735
sp|P12694|ODBA_HUMAN	-0.2400513	0.7061832
sp|P55809|SCOT1_HUMAN	-0.2399292	0.35795313
sp|O75923-11|DYSF_HUMAN	-0.2398949	0.04707252
sp|P60709|ACTB_HUMAN	-0.238327	0
sp|P29144|TPP2_HUMAN	-0.2347889	0.1558116
sp|P26599-2|PTBP1_HUMAN	-0.2311459	1.1687785
sp|Q92905|CSN5_HUMAN	-0.2304936	0
sp|O43252|PAPS1_HUMAN	-0.2302322	0.09894868
sp|Q15274|NADC_HUMAN	-0.2296143	0.5204253
sp|Q9UIJ7|KAD3_HUMAN	-0.2293263	1.1240381
sp|Q02790|FKBP4_HUMAN	-0.2291393	1.0485198
sp|O43776|SYNC_HUMAN	-0.2289372	0.79861414
sp|P22059|OSBP1_HUMAN	-0.2279854	1.1932944
sp|P15153|RAC2_HUMAN	-0.2277737	0.656254
sp|O00567|NOP56_HUMAN	-0.2266712	0.34137914
sp|Q96MM6|HS12B_HUMAN	-0.226017	0.24097534
sp|P62140|PP1B_HUMAN	-0.2240238	1.1505735
sp|P31942-2|HNRH3_HUMAN	-0.2217197	1.0634323
sp|O00534|VMA5A_HUMAN	-0.2216549	0.3005443
sp|P12268|IMDH2_HUMAN	-0.220747	0
sp|O14828-2|SCAM3_HUMAN	-0.2202301	0.35795313
sp|Q15833-2|STXB2_HUMAN	-0.2200851	0.7827403
sp|P84095|RHOG_HUMAN	-0.2183323	0.5204253
sp|P55209-2|NP1L1_HUMAN	-0.2179508	0.656254
sp|P04632|CPNS1_HUMAN	-0.217268	0.9329067
sp|O60701-2|UGDH_HUMAN	-0.216444	0
sp|Q9H9B4|SFXN1_HUMAN	-0.2156639	1.1932944
sp|Q8NDH3|PEPL1_HUMAN	-0.2156563	1.0485198
sp|P13693|TCTP_HUMAN	-0.2138596	0.19149946

sp|P16671-4|CD36_HUMAN	-0.2136784	1.2095301
sp|P28066|PSA5_HUMAN	-0.2126808	1.3006523
sp|Q9Y2B0|CNPY2_HUMAN	-0.2110195	0.09894868
sp|P55265-4|DSRAD_HUMAN	-0.2096806	0.35795313
sp|P68400|CSK21_HUMAN	-0.2092476	0.61034113
sp|Q9NYL9|TMOD3_HUMAN	-0.2087517	0
sp|P48739|PIPNB_HUMAN	-0.2081757	0.45033538
sp|P24158|PRTN3_HUMAN	-0.2076092	0.2178309
sp|Q9UKV3-5|ACINU_HUMAN	-0.2073059	0.8983557
sp|P06865|HEXA_HUMAN	-0.2071686	0.35795313
sp|P39687|AN32A_HUMAN	-0.2052918	1.1505735
sp|Q6NY19-2|KANK3_HUMAN	-0.2045479	0.7827403
sp|P13861|KAP2_HUMAN	-0.2045002	1.1034192
sp|P05534|1A24_HUMAN	-0.2041206	0.5204253
sp|O14617-4|AP3D1_HUMAN	-0.2037735	0.45033538
sp|Q16539-2|MK14_HUMAN	-0.2036743	0.19149946
sp|P36542-2|ATPG_HUMAN	-0.2026434	0.656254
sp|O75340-2|PDCD6_HUMAN	-0.2024212	0.35795313
sp|Q99829|CPNE1_HUMAN	-0.2004013	0.6509351
sp|P50395|GDIB_HUMAN	-0.1990891	0.82809293
sp|Q92769|HDAC2_HUMAN	-0.1990471	0.656254
sp|Q15631|TSN_HUMAN	-0.1975956	0.06291623
sp|P49961-6|ENTP1_HUMAN	-0.1975021	0.35795313
sp|Q13232|NDK3_HUMAN	-0.1974106	0.6298893
sp|Q9HD45|TM9S3_HUMAN	-0.1967049	0
sp|P62701|RS4X_HUMAN	-0.1963139	0.5111962
sp|P62314|SMD1_HUMAN	-0.1961021	0.45033538
sp|P61081|UBC12_HUMAN	-0.1955872	0.79861414
sp|P08572|CO4A2_HUMAN	-0.1951428	1.0951192
sp|P55263|ADK_HUMAN	-0.1929112	0.8983557
BirA-TRIP6_BirAT6	-0.1922684	0.45033538
sp|P16298-4|PP2BB_HUMAN	-0.1922379	0.6070219
sp|Q92696|PGTA_HUMAN	-0.1916466	0.79861414
sp|Q8NBJ5|GT251_HUMAN	-0.19137	1.1505735
sp|Q15121|PEA15_HUMAN	-0.1904755	0.2178309
sp|P11279|LAMP1_HUMAN	-0.190115	0.19149946
sp|O60234|GMFG_HUMAN	-0.1900387	0.2178309
sp|P54727|RD23B_HUMAN	-0.1893253	0.91601294
sp|Q9P2T1-2|GMPR2_HUMAN	-0.1889095	0.09339783
sp|P57088|TMM33_HUMAN	-0.1876469	0.45033538
sp|P54136|SYRC_HUMAN	-0.1870594	0.65625405
sp|P61088|UBE2N_HUMAN	-0.1857834	0.45033538
sp|P45954-2|ACDSB_HUMAN	-0.1855202	0.19149946
sp|Q9HC38|GLOD4_HUMAN	-0.1844177	0.8339169
sp|P11586|C1TC_HUMAN	-0.1836376	0.5204253

sp|P24557-2|THAS_HUMAN	-0.1829262	0.45033538
sp|P07741|APT_HUMAN	-0.1824532	0.43103603
sp|A6NMZ7|CO6A6_HUMAN	-0.1816902	0.9847622
sp|Q13423|NNTM_HUMAN	-0.1816559	0.6115017
sp|Q13492-2|PICAL_HUMAN	-0.1812382	0.2178309
sp|O15511|ARPC5_HUMAN	-0.1810036	1.2095301
sp|Q08945|SSRP1_HUMAN	-0.1807556	0.84879977
sp|P09874|PARP1_HUMAN	-0.1801186	0.7957244
sp|Q13884|SNTB1_HUMAN	-0.1796837	0
sp|P52895|AK1C2_HUMAN	-0.17941	0.19149946
sp|Q9Y262-2|EIF3L_HUMAN	-0.1792469	0.5832693
sp|Q9Y696|CLIC4_HUMAN	-0.1781616	0.23541966
sp|Q9Y3F4-2|STRAP_HUMAN	-0.1773987	0.35795313
sp|Q01813-2|PFKAP_HUMAN	-0.1759987	0.37060758
sp|O75112-7|LDB3_HUMAN	-0.1751938	0.35795313
sp|O00232|PSD12_HUMAN	-0.1751804	0.35193655
sp|Q6DD88|ATLA3_HUMAN	-0.1743622	0.7957244
sp|P07203|GPX1_HUMAN	-0.1743279	0
sp|P62879|GBB2_HUMAN	-0.1726532	0.30372584
sp|P24534|EF1B_HUMAN	-0.1726017	0.7827403
sp|O15067|PUR4_HUMAN	-0.1722183	1.1932944
sp|P49593-2|PPM1F_HUMAN	-0.1720772	0.30372584
sp|Q9NUV9|GIMA4_HUMAN	-0.1718903	0.17171621
sp|Q16363-2|LAMA4_HUMAN	-0.1714029	0.95703053
sp|Q14165|MLEC_HUMAN	-0.1707001	0.7061832
sp|Q9Y5M8|SRPRB_HUMAN	-0.1705074	0.40256184
sp|Q9BRF8-2|CPPED_HUMAN	-0.1700916	0.35795313
sp|P48681|NEST_HUMAN	-0.1700325	0.5474665
sp|Q30154|DRB5_HUMAN	-0.1697693	0
sp|Q16629-2|SRSF7_HUMAN	-0.1697216	0.40256184
sp|Q9Y6A4|CFA20_HUMAN	-0.1689777	0.45033538
sp|P22314|UBA1_HUMAN	-0.1684513	0.62441224
sp|P0CG39|POTEJ_HUMAN	-0.1682377	0
sp|P33176|KINH_HUMAN	-0.1677656	0.7118947
sp|P34897-2|GLYM_HUMAN	-0.1671352	1.0370445
sp|P22033|MUTA_HUMAN	-0.1655178	0.5204253
sp|P22061-2|PIMT_HUMAN	-0.163353	1.2095301
sp|P31937|3HIDH_HUMAN	-0.1628838	0.35795313
sp|Q86UX2-2|ITIH5_HUMAN	-0.162281	0.312067
sp|Q562R1|ACTBL_HUMAN	-0.1615028	0
sp|P62277|RS13_HUMAN	-0.1603108	1.0485198
sp|Q9Y3B3|TMED7_HUMAN	-0.1596642	1.1932944
sp|Q9Y3D6|FIS1_HUMAN	-0.1586695	0.5204253
sp|Q9UN86-2|G3BP2_HUMAN	-0.1584501	0
sp|P55884-2|EIF3B_HUMAN	-0.1583366	0.26737198

sp|Q14254|FLOT2_HUMAN	-0.158186	0.45720983
sp|O15173-2|PGRC2_HUMAN	-0.1575871	0.40256184
sp|P30520|PURA2_HUMAN	-0.157095	0.45720983
sp|Q96G03|PGM2_HUMAN	-0.1566505	0.7022581
sp|P23229-4|ITA6_HUMAN	-0.1561813	1.1932944
sp|O15400-2|STX7_HUMAN	-0.1559467	0.37060758
sp|P40763-3|STAT3_HUMAN	-0.1558456	1.0485198
sp|Q27J81-2|INF2_HUMAN	-0.1553707	0.2178309
sp|P61923|COPZ1_HUMAN	-0.1549311	0.19149946
sp|O75436|VP26A_HUMAN	-0.1545162	0.56808305
sp|P20073-2|ANXA7_HUMAN	-0.1540241	0.93112767
sp|P55735-2|SEC13_HUMAN	-0.1534157	0.5111962
sp|O43615|TIM44_HUMAN	-0.1523752	0.19149946
sp|O43865|SAHH2_HUMAN	-0.1506205	0.35795313
sp|Q8NHV1|GIMA7_HUMAN	-0.1504784	0.7827403
sp|P43686|PRS6B_HUMAN	-0.1501875	1.0242546
sp|O94973-2|AP2A2_HUMAN	-0.1492653	0.37128913
sp|O75937|DNJC8_HUMAN	-0.1462212	0.40256184
sp|P46108|CRK_HUMAN	-0.1459999	0.6509351
sp|Q16787-3|LAMA3_HUMAN	-0.1459293	0.656254
sp|P48643|TCPE_HUMAN	-0.1439438	0.80320555
sp|P26196|DDX6_HUMAN	-0.1436501	0.09894868
sp|P09496-2|CLCA_HUMAN	-0.1425285	0.21439649
sp|P67775|PP2AA_HUMAN	-0.1415978	0
sp|Q93052|LPP_HUMAN	-0.1409588	1.0604883
sp|P13987-2|CD59_HUMAN	-0.1405716	0.5204253
sp|Q8TAT6-2|NPL4_HUMAN	-0.1399822	0.40256184
sp|Q14108|SCRB2_HUMAN	-0.1385899	1.1932944
sp|Q13363-2|CTBP1_HUMAN	-0.1383572	0.19149946
sp|P58107|EPIPL_HUMAN	-0.1379566	0.6115017
sp|P50914|RL14_HUMAN	-0.1379089	0.2178309
sp|P42224-2|STAT1_HUMAN	-0.1377792	0.33495146
sp|P42704|LPPRC_HUMAN	-0.1377487	0.90036625
sp|P49903-2|SPS1_HUMAN	-0.1369476	0.2178309
sp|O75396|SC22B_HUMAN	-0.1362915	0.6509351
sp|P26885|FKBP2_HUMAN	-0.1360378	0
sp|Q99460|PSMD1_HUMAN	-0.1359406	0.3761346
sp|P23528|COF1_HUMAN	-0.1356468	1.1470993
sp|Q6DKJ4|NXN_HUMAN	-0.1350031	0.19149946
sp|P49720|PSB3_HUMAN	-0.1346817	0.95332193
sp|P05165-2|PCCA_HUMAN	-0.1340275	1.0604883
sp|O95571|ETHE1_HUMAN	-0.1319809	0.56808305
sp|O75347|TBCA_HUMAN	-0.1307278	0.09894868
sp|Q14515-2|SPRL1_HUMAN	-0.1291733	0.45033538
sp|Q9BX97|PLVAP_HUMAN	-0.1290817	0.45033538

sp|P09493-5|TPM1_HUMAN	-0.1288967	0.7827403
sp|Q9UJZ1-2|STML2_HUMAN	-0.1284008	0.7061832
sp|P32456|GBP2_HUMAN	-0.1273136	0.45033538
sp|P61970|NTF2_HUMAN	-0.1254654	0.5204253
sp|P22234-2|PUR6_HUMAN	-0.1253624	0.70235044
sp|Q14980-2|NUMA1_HUMAN	-0.1249523	1.145722
sp|Q53T59|H1BP3_HUMAN	-0.1249332	0.7827403
sp|P84085|ARF5_HUMAN	-0.1247597	0.656254
sp|Q96EP5-2|DAZP1_HUMAN	-0.1242733	0.19149946
sp|Q13561-2|DCTN2_HUMAN	-0.1223984	0.59872687
sp|P29692-2|EF1D_HUMAN	-0.1218185	1.1505735
sp|P01911|2B1F_HUMAN	-0.1213837	0.45033538
sp|P46459|NSF_HUMAN	-0.1211662	0.1342476
sp|O15296|LX15B_HUMAN	-0.1211433	0.1342476
sp|Q96FN4|CPNE2_HUMAN	-0.12006	0
sp|P61204|ARF3_HUMAN	-0.1195717	0.4075265
sp|Q9NY33|DPP3_HUMAN	-0.1185131	0.46530083
sp|Q9HCN8|SDF2L_HUMAN	-0.1183548	0.19149946
sp|P26639-2|SYTC_HUMAN	-0.1179199	0.56639653
sp|P09543-2|CN37_HUMAN	-0.116684	0.06842031
sp|Q16527|CSRP2_HUMAN	-0.1164074	0.5204253
sp|P06748-2|NPM_HUMAN	-0.115427	0.35795313
sp|P18669|PGAM1_HUMAN	-0.1154194	0.51708394
sp|P14618|KPYM_HUMAN	-0.1154079	0.04454162
sp|P08865|RSSA_HUMAN	-0.115345	0.7827403
sp|Q969H8|MYDGF_HUMAN	-0.1151009	0.2178309
sp|Q13630|FCL_HUMAN	-0.1141396	0.45033538
sp|P39656-3|OST48_HUMAN	-0.113615	0.86708647
sp|Q15293|RCN1_HUMAN	-0.1131287	0.95332193
sp|P07814|SYEP_HUMAN	-0.1127634	0.5283125
sp|Q9P1F3|ABRAL_HUMAN	-0.1125431	0
sp|Q96CX2|KCD12_HUMAN	-0.1117992	0.3731982
sp|P15529-10|MCP_HUMAN	-0.1113529	0
sp|P46779-2|RL28_HUMAN	-0.1111259	0.40256184
sp|Q15008-4|PSMD6_HUMAN	-0.1101151	0
sp|Q92542|NICA_HUMAN	-0.1096077	0.7061832
sp|Q10567-2|AP1B1_HUMAN	-0.1086044	0.5204253
sp|P20674|COX5A_HUMAN	-0.1080666	0.09894868
sp|P32455|GBP1_HUMAN	-0.1073017	1.120602
sp|Q13596-2|SNX1_HUMAN	-0.1067677	0
sp|Q8TD19|NEK9_HUMAN	-0.1044712	0.45033538
sp|P16278-2|BGAL_HUMAN	-0.1041126	0.7498006
sp|P26640|SYVC_HUMAN	-0.1040306	0.2207585
sp|Q96CN7|ISOC1_HUMAN	-0.1039429	0.33495146
sp|P27694|RFA1_HUMAN	-0.1033993	0.26737198

sp|P05166-2|PCCB_HUMAN	-0.1033669	0.6115017
sp|Q15746-2|MYLK_HUMAN	-0.1031418	0.27907842
sp|P58546|MTPN_HUMAN	-0.1027699	0.35795313
sp|P49588-2|SYAC_HUMAN	-0.0989838	0.525685
sp|P51636-2|CAV2_HUMAN	-0.0988159	0.19149946
sp|P09429|HMGB1_HUMAN	-0.0987396	0.19149946
sp|Q9GZM7-3|TINAL_HUMAN	-0.098424	0
sp|Q02818|NUCB1_HUMAN	-0.0956516	0.5360584
sp|A0FGR8-2|ESYT2_HUMAN	-0.0949879	0.5111962
sp|Q9UNZ2-5|NSF1C_HUMAN	-0.0949154	0.43103603
sp|P62633-3|CNBP_HUMAN	-0.0948505	0
sp|Q92499|DDX1_HUMAN	-0.0943861	0.57986265
sp|P15144|AMPN_HUMAN	-0.0937691	0.51870745
sp|P09382|LEG1_HUMAN	-0.0930729	0.56639653
sp|Q7KZF4|SND1_HUMAN	-0.0929852	0.312067
sp|Q9UBQ5|EIF3K_HUMAN	-0.0928574	0
sp|O95861-4|BPNT1_HUMAN	-0.0925903	0.5111962
sp|Q9BUT1|BDH2_HUMAN	-0.0923557	0.6115017
sp|O00264|PGRC1_HUMAN	-0.0922871	0.70235044
sp|P17174|AATC_HUMAN	-0.092104	0.37410614
sp|Q9Y4L1|HYOU1_HUMAN	-0.0918198	0.5681111
sp|P30519|HMOX2_HUMAN	-0.0907717	0.19149946
sp|P41091|IF2G_HUMAN	-0.0905476	0.04454162
sp|O60763-2|USO1_HUMAN	-0.0896759	0.01343579
sp|P62913-2|RL11_HUMAN	-0.08951	0
sp|Q9Y230|RUVB2_HUMAN	-0.0894756	0.01044662
sp|P33316|DUT_HUMAN	-0.088604	1.1932944
sp|O60547-2|GMDS_HUMAN	-0.0884628	0.35795313
sp|P37108|SRP14_HUMAN	-0.0879316	0.19149946
sp|P78371|TCPB_HUMAN	-0.0874977	0.2711284
sp|P31689-2|DNJA1_HUMAN	-0.0870171	0.19149946
sp|P54652|HSP72_HUMAN	-0.0865955	0
sp|Q9Y678|COPG1_HUMAN	-0.0861607	0.5474665
sp|Q9UEY8|ADDG_HUMAN	-0.0855942	1.0918401
sp|P48449-3|ERG7_HUMAN	-0.0848427	0.5111962
sp|Q15149-9|PLEC_HUMAN	-0.0848236	0.656254
sp|Q9UHL4|DPP2_HUMAN	-0.084816	0.74639726
sp|Q9NQG5|RPR1B_HUMAN	-0.0833244	0.35795313
sp|Q08431|MFGM_HUMAN	-0.0817394	0.35795313
sp|O95466-2|FMNL1_HUMAN	-0.0803299	1.1932944
sp|Q99439|CNN2_HUMAN	-0.0796528	0.02174174
sp|Q709C8-3|VP13C_HUMAN	-0.0784817	0.17171621
sp|Q92629-3|SGCD_HUMAN	-0.0781059	0.40256184
sp|P13928|ANXA8_HUMAN	-0.0780411	0
sp|Q9NRV9|HEBP1_HUMAN	-0.0774002	0.40256184

sp|P22102|PUR2_HUMAN	-0.0757637	0.21439649
sp|O00571-2|DDX3X_HUMAN	-0.0747204	0.7061832
sp|Q04323-2|UBXN1_HUMAN	-0.0740185	0
sp|P60981|DEST_HUMAN	-0.0735512	0.09339783
sp|Q99436|PSB7_HUMAN	-0.0731058	0.19149946
sp|P35579-2|MYH9_HUMAN	-0.0725164	0.656254
sp|B5ME19|EIFCL_HUMAN	-0.0724373	0.2178309
sp|P01112|RASH_HUMAN	-0.0722866	0
sp|O94804|STK10_HUMAN	-0.0721092	0.19149946
sp|Q9P2E9|RRBP1_HUMAN	-0.0715218	1.2731345
sp|P02794|FRIH_HUMAN	-0.0707245	0.26737198
sp|P45974-2|UBP5_HUMAN	-0.0693283	0.26295313
sp|P50135|HNMT_HUMAN	-0.0691433	0
sp|P61020|RAB5B_HUMAN	-0.0676289	0.2178309
sp|Q92556|ELMO1_HUMAN	-0.067193	0.21439649
sp|P23588|IF4B_HUMAN	-0.0668793	0.21439649
sp|Q8WX93-5|PALLD_HUMAN	-0.0664921	0.2178309
sp|O75489|NDUS3_HUMAN	-0.0651407	0.30372584
sp|P08238|HS90B_HUMAN	-0.0645485	0.34244674
sp|P31939|PUR9_HUMAN	-0.063983	0.13973783
sp|Q9UHX1-6|PUF60_HUMAN	-0.0637913	0.2178309
sp|P30044-2|PRDX5_HUMAN	-0.0636978	0.656254
sp|P49821-2|NDUV1_HUMAN	-0.0631838	0.06953826
sp|Q9Y6W5|WASF2_HUMAN	-0.062624	0.5204253
sp|P04844|RPN2_HUMAN	-0.0624676	0.70235044
sp|Q93084-2|AT2A3_HUMAN	-0.0624199	0.45033538
sp|P14868|SYDC_HUMAN	-0.060606	0.85807747
sp|O95336|6PGL_HUMAN	-0.0596199	0.02174174
sp|P09972|ALDOC_HUMAN	-0.0576382	0.26737198
sp|P09619|PGFRB_HUMAN	-0.0574245	0.09894868
sp|Q92688-2|AN32B_HUMAN	-0.0572701	0.35795313
sp|P46977|STT3A_HUMAN	-0.0568333	0.45033538
sp|P25325-2|THTM_HUMAN	-0.0567694	0.25789237
sp|P54920|SNAA_HUMAN	-0.0561543	0.6255959
sp|Q7L576|CYFP1_HUMAN	-0.0541325	0.19149946
sp|Q15942|ZYX_HUMAN	-0.0523491	0.09339783
sp|P41250|GARS_HUMAN	-0.0522776	0.4668234
sp|Q6UW68|TM205_HUMAN	-0.051693	0.45033538
sp|P62081|RS7_HUMAN	-0.0510235	0.7061832
sp|O43681|ASNA_HUMAN	-0.0503845	0.2178309
sp|Q9HCB6|SPON1_HUMAN	-0.0499268	0
sp|Q3LXA3|TKFC_HUMAN	-0.0498981	0.1318641
sp|O60313-10|OPA1_HUMAN	-0.0487309	0.19149946
sp|P00387-3|NB5R3_HUMAN	-0.0478554	0.3005443
sp|P61026|RAB10_HUMAN	-0.0476408	0.19149946

sp|O95394-3|AGM1_HUMAN	-0.0471401	0
sp|P08631-2|HCK_HUMAN	-0.0470819	0
sp|Q9BT78|CSN4_HUMAN	-0.0470467	0.06291623
sp|P27105|STOM_HUMAN	-0.045784	0.1804695
sp|O75306-2|NDUS2_HUMAN	-0.0451946	0.06291623
sp|P35611-2|ADDA_HUMAN	-0.0446072	0.02174174
sp|Q99873-3|ANM1_HUMAN	-0.0425453	0.14672586
sp|P46783|RS10_HUMAN	-0.0418558	0.19149946
sp|P84098|RL19_HUMAN	-0.0417519	0
sp|Q8NCW5|NNRE_HUMAN	-0.0415916	0.21439649
sp|P39019|RS19_HUMAN	-0.0409985	0.2178309
sp|P21291|CSRP1_HUMAN	-0.0409431	0.39705694
sp|Q13724-2|MOGS_HUMAN	-0.0402584	0.8339169
sp|Q9UBC2-2|EP15R_HUMAN	-0.0401592	0
sp|P62195|PRS8_HUMAN	-0.0387783	0
sp|Q12797-10|ASPH_HUMAN	-0.0371933	0.16702904
sp|Q9NR45|SIAS_HUMAN	-0.0368538	0.31741163
sp|O43488|ARK72_HUMAN	-0.036293	0.06291623
sp|P25787|PSA2_HUMAN	-0.0352135	0.2207585
sp|P23497|SP100_HUMAN	-0.0345612	0.45033538
sp|Q15075|EEA1_HUMAN	-0.0339527	0.18700324
sp|P53618|COPB_HUMAN	-0.0339432	0.0426314
sp|Q7Z4W1|DCXR_HUMAN	-0.0333366	0.45563722
sp|O75915|PRAF3_HUMAN	-0.0333157	0.35795313
sp|Q06210-2|GFPT1_HUMAN	-0.0301228	0.26737198
sp|P08571|CD14_HUMAN	-0.0298405	0.1342476
sp|P55010|IF5_HUMAN	-0.0288944	0
sp|O14980|XPO1_HUMAN	-0.0277443	0.09894868
sp|Q14141-2|SEPT6_HUMAN	-0.0274506	0.656254
sp|O00170|AIP_HUMAN	-0.0270004	0.5111962
sp|Q9NUQ9|FA49B_HUMAN	-0.0263767	0
sp|P48147|PPCE_HUMAN	-0.0256748	0.14927356
sp|Q16630-2|CPSF6_HUMAN	-0.0254631	0.09894868
sp|Q92598-2|HS105_HUMAN	-0.0246983	0
sp|P62304|RUXE_HUMAN	-0.0236645	0.19149946
sp|Q9BZZ5-5|API5_HUMAN	-0.0234489	0.09339783
sp|P17980|PRS6A_HUMAN	-0.0230465	0.09339783
sp|P12270|TPR_HUMAN	-0.0229683	0.2718748
sp|P12110|CO6A2_HUMAN	-0.0210533	0.01189081
sp|P15090|FABP4_HUMAN	-0.0208626	0.29066643
sp|Q15819|UB2V2_HUMAN	-0.0195999	0
sp|Q8WXX5|DNJC9_HUMAN	-0.0188141	0.19149946
sp|P30512|1A29_HUMAN	-0.0186005	0.35795313
sp|Q9BUF5|TBB6_HUMAN	-0.018384	0.40256184
sp|Q13885|TBB2A_HUMAN	-0.0169811	0.19149946

sp|P00441|SODC_HUMAN	-0.0163536	0.26737198
sp|O75503|CLN5_HUMAN	-0.0159435	0.09894868
sp|P52758|RIDA_HUMAN	-0.0158463	0
sp|O43852-3|CALU_HUMAN	-0.0129547	0
sp|P14550|AK1A1_HUMAN	-0.0099106	0.03857618
sp|Q8N392|RHG18_HUMAN	-0.0085945	0.2178309
sp|P34932|HSP74_HUMAN	-0.008049	0.20037685
sp|P55060-3|XPO2_HUMAN	-0.0077572	0.37060758
sp|Q14204|DYHC1_HUMAN	-0.0038185	0.34157792
sp|Q8N163-2|CCAR2_HUMAN	-0.0036983	0
sp|P27487|DPP4_HUMAN	-0.0012398	0.21439649
sp|P67936|TPM4_HUMAN	-7.00E-04	0.15980355
sp|P78417-2|GSTO1_HUMAN	-4.50E-04	0
sp|P35908|K22E_HUMAN	-3.24E-05	0.03956581
sp|P55795|HNRH2_HUMAN	2.86E-05	0.17319627
sp|Q16775-2|GLO2_HUMAN	9.63E-04	0.09894868
sp|Q07812-7|BAX_HUMAN	0.0013504	0
sp|Q9BTV4|TMM43_HUMAN	0.00180435	0.20467198
sp|P51665|PSMD7_HUMAN	0.00189972	0.19149946
sp|P21810|PGS1_HUMAN	0.00282288	0.29919147
sp|Q86VP6|CAND1_HUMAN	0.00309181	0.12540545
sp|Q13464|ROCK1_HUMAN	0.00310135	0
sp|Q13526|PIN1_HUMAN	0.00333595	0.19149946
sp|P49257|LMAN1_HUMAN	0.00388527	0.23973103
sp|O60716-14|CTND1_HUMAN	0.00398064	0.09894868
sp|Q16134-3|ETFD_HUMAN	0.00635815	0
sp|P18583-10|SON_HUMAN	0.00715256	0
sp|O60884|DNJA2_HUMAN	0.00748825	0.1342476
sp|Q9UJU6-2|DBNL_HUMAN	0.00782013	0
sp|P00338|LDHA_HUMAN	0.00788879	0.09157685
sp|P28074|PSB5_HUMAN	0.00802422	0.14672586
sp|O43396|TXNL1_HUMAN	0.00848961	0.19149946
sp|P67809|YBOX1_HUMAN	0.01111794	0.45033538
sp|O14787-2|TNPO2_HUMAN	0.01132202	0
sp|P12277|KCRB_HUMAN	0.01162148	0.02347357
sp|Q9UBG0|MRC2_HUMAN	0.01188374	0.3630891
sp|Q8WVM8|SCFD1_HUMAN	0.01241493	0.17511293
sp|Q9NP79|VTA1_HUMAN	0.01251602	0.09894868
sp|P52888|THOP1_HUMAN	0.0138607	0.19149946
sp|P09104-2|ENOG_HUMAN	0.0144043	0.29066643
sp|P36776-3|LONM_HUMAN	0.0157938	0.09339783
sp|Q6UVK1|CSPG4_HUMAN	0.01695252	0.14672586
sp|P05387|RLA2_HUMAN	0.01774979	0.19149946
sp|Q9UNS2|CSN3_HUMAN	0.01825714	0.656254
sp|Q93034|CUL5_HUMAN	0.01905823	0

sp|P14174|MIF_HUMAN	0.01927948	0.2178309
sp|Q14651|PLSI_HUMAN	0.01940918	0
sp|P12109|CO6A1_HUMAN	0.01964569	0.03984664
sp|Q96HE7|ERO1A_HUMAN	0.02066422	0.2178309
sp|Q15124|PGM5_HUMAN	0.02070236	0.2220352
sp|O14579|COPE_HUMAN	0.02210808	0.04454162
sp|Q6XQN6-2|PNCB_HUMAN	0.02308846	0.31741163
sp|O15498-2|YKT6_HUMAN	0.02447701	0
sp|P18428|LBP_HUMAN	0.02462006	0
sp|P30046|DOPD_HUMAN	0.02571487	0
sp|Q9Y3A5|SBDS_HUMAN	0.02589035	0.11949348
sp|Q5EBM0|CMPK2_HUMAN	0.02669907	0.35795313
sp|P49755|TMEDA_HUMAN	0.0267477	0.1342476
sp|Q9UBT2|SAE2_HUMAN	0.0273056	0.30372584
sp|Q12792-3|TWF1_HUMAN	0.02772141	0
sp|Q9BVK6|TMED9_HUMAN	0.02832413	0
sp|Q08380|LG3BP_HUMAN	0.0297966	0.37061578
sp|Q15257-2|PTPA_HUMAN	0.0301609	0.09339783
sp|P27918|PROP_HUMAN	0.03129387	0
sp|P15880|RS2_HUMAN	0.03166962	0.1342476
sp|P80303-2|NUCB2_HUMAN	0.03265381	0.35193655
sp|Q9NQW7-3|XPP1_HUMAN	0.03285599	0.17171621
sp|Q92747|ARC1A_HUMAN	0.03653336	0
sp|Q16401-2|PSMD5_HUMAN	0.03747177	0.17511293
sp|P25685-2|DNJB1_HUMAN	0.03782845	0.19149946
sp|Q9Y5K5-2|UCHL5_HUMAN	0.03815079	0
sp|Q12765|SCRN1_HUMAN	0.03853226	0.40256184
sp|Q13243-3|SRSF5_HUMAN	0.03871918	0
sp|P12814|ACTN1_HUMAN	0.03941536	0
sp|O00487|PSDE_HUMAN	0.03975296	0.19149946
sp|O43294|TGFI1_HUMAN	0.0405159	0.19149946
sp|Q15691|MARE1_HUMAN	0.04193211	0.26737198
sp|P20591|MX1_HUMAN	0.04377556	0
sp|P13798|ACPH_HUMAN	0.04426575	0.368128
sp|O00151|PDLI1_HUMAN	0.0443058	0.29781067
sp|O43175|SERA_HUMAN	0.04465866	0.7220187
sp|P54578-3|UBP14_HUMAN	0.04481506	0.21439649
sp|Q93009-3|UBP7_HUMAN	0.04609299	0
sp|P02750|A2GL_HUMAN	0.04667282	0.01653191
sp|Q9Y224|RTRAF_HUMAN	0.04802704	0.14672586
sp|Q9NZ32|ARP10_HUMAN	0.05029678	0.7827403
sp|P30533|AMRP_HUMAN	0.05116272	0.26737198
sp|P62333|PRS10_HUMAN	0.05125237	0.06842031
sp|P31321|KAP1_HUMAN	0.05453682	0
sp|O94979-10|SC31A_HUMAN	0.05490303	0.04707252

sp|P12111-2|CO6A3_HUMAN	0.05517578	0.1500082
sp|Q96M27-3|PRRC1_HUMAN	0.05553055	0.19149946
sp|P04275|VWF_HUMAN	0.05575752	0.97661924
sp|Q9UI12-2|VATH_HUMAN	0.05659676	0.26737198
sp|P21589-2|5NTD_HUMAN	0.05721474	0
sp|P04792|HSPB1_HUMAN	0.05777931	1.0586762
sp|P04040|CATA_HUMAN	0.05855942	1.0524595
sp|Q9NTK5|OLA1_HUMAN	0.05897236	0.19149946
sp|O60506-3|HNRPQ_HUMAN	0.05900478	0.7588735
sp|P61201-2|CSN2_HUMAN	0.05902672	0.19149946
sp|P50148|GNAQ_HUMAN	0.05976677	0.6298893
sp|Q86VS8|HOOK3_HUMAN	0.05986786	0.45033538
sp|Q9NZK5|ADA2_HUMAN	0.06057739	0.45033538
sp|Q9Y265|RUVB1_HUMAN	0.06059074	0.42646354
sp|P52306-4|GDS1_HUMAN	0.06123352	0.09894868
sp|Q15404|RSU1_HUMAN	0.06371689	0.656254
sp|Q9P2X0-2|DPM3_HUMAN	0.06537056	0
sp|P35580-3|MYH10_HUMAN	0.06591034	0.55290085
sp|P00568|KAD1_HUMAN	0.06592369	0.7284051
sp|P62070-4|RRAS2_HUMAN	0.06636047	0.656254
sp|P57737-4|CORO7_HUMAN	0.06642056	0.19149946
sp|P56134-3|ATPK_HUMAN	0.06839371	0
sp|Q92930|RAB8B_HUMAN	0.06845856	0.656254
sp|P18084|ITB5_HUMAN	0.06905174	0
sp|P06756-3|ITAV_HUMAN	0.06916237	0.7498006
sp|Q15417|CNN3_HUMAN	0.07099724	0.7061832
sp|Q9BS40|LXN_HUMAN	0.07287788	0.1342476
sp|P55145|MANF_HUMAN	0.07406616	0
sp|P46821|MAP1B_HUMAN	0.07590866	0.45563722
sp|P53602|MVD1_HUMAN	0.07735252	0.5204253
sp|P04406|G3P_HUMAN	0.08153534	0
sp|P02511|CRYAB_HUMAN	0.08383179	0.35795313
sp|P00492|HPRT_HUMAN	0.08453751	1.2095301
sp|Q9GZT8|NIF3L_HUMAN	0.08526135	0.19149946
sp|Q8TBC4-2|UBA3_HUMAN	0.08779526	0.45033538
sp|Q9BWD1|THIC_HUMAN	0.08916473	0.19149946
sp|P35637-2|FUS_HUMAN	0.0908823	0
sp|Q16774|KGUA_HUMAN	0.09288788	0
sp|Q14112-2|NID2_HUMAN	0.09632111	0.76289684
sp|Q92900-2|RENT1_HUMAN	0.09783363	0.16649151
sp|Q8IUX7|AEBP1_HUMAN	0.09789276	0.29066643
sp|Q9NY15|STAB1_HUMAN	0.09963226	0.19149946
sp|P61018-2|RAB4B_HUMAN	0.10134602	0.656254
sp|Q6PCB0|VWA1_HUMAN	0.10329628	0.2178309
sp|O94855-2|SC24D_HUMAN	0.1058712	0.19149946

sp|P18085|ARF4_HUMAN	0.1075058	0.656254
sp|P49721|PSB2_HUMAN	0.10857964	0.19149946
sp|Q14847|LASP1_HUMAN	0.10955811	0.2591514
sp|Q9H008|LHPP_HUMAN	0.11166763	0.19410844
sp|P13645|K1C10_HUMAN	0.11439514	0.9357687
sp|Q9GZP4-2|PITH1_HUMAN	0.11668396	0.19149946
sp|O00233-2|PSMD9_HUMAN	0.11686897	0.19149946
sp|P98082-2|DAB2_HUMAN	0.1192379	0.19149946
sp|P22392-2|NDKB_HUMAN	0.11961556	1.1791906
sp|P51692|STA5B_HUMAN	0.12194252	1.1932944
sp|Q12882|DPYD_HUMAN	0.12417793	0.19149946
sp|P30466|1B18_HUMAN	0.12433243	0.656254
sp|O75368|SH3L1_HUMAN	0.12571144	0.5204253
sp|P62857|RS28_HUMAN	0.12592316	0.2178309
sp|Q9BRR6-2|ADPGK_HUMAN	0.12743759	0.45033538
sp|Q15436|SC23A_HUMAN	0.12750435	0.5111962
sp|Q9C0C2|TB182_HUMAN	0.12765121	0.48520416
sp|Q16643-3|DREB_HUMAN	0.12899399	0.06291623
sp|P27695|APEX1_HUMAN	0.1294384	0
sp|P30837|AL1B1_HUMAN	0.13170242	0.33495146
sp|Q03519|TAP2_HUMAN	0.1342659	0.656254
sp|P08237-3|PFKAM_HUMAN	0.13575554	0.95332193
sp|P56537|IF6_HUMAN	0.13582802	0.7498006
sp|O60256-3|KPRB_HUMAN	0.13649368	0.7827403
sp|Q13310-2|PABP4_HUMAN	0.13698196	0.656254
sp|Q5K4L6|S27A3_HUMAN	0.13990021	0.7061832
sp|P42285|MTREX_HUMAN	0.14125156	0.19149946
sp|P43243|MATR3_HUMAN	0.14200783	0.04454162
sp|Q13867|BLMH_HUMAN	0.14506531	0.45033538
sp|P39059|COFA1_HUMAN	0.14800644	1.0485198
sp|P54577|SYYC_HUMAN	0.14897346	0.35795313
sp|O00429-6|DNM1L_HUMAN	0.15422821	0.1500082
sp|O60664-4|PLIN3_HUMAN	0.15435028	0.56808305
sp|O75533|SF3B1_HUMAN	0.15912628	0.19149946
sp|Q15437|SC23B_HUMAN	0.1604414	0.656254
sp|Q9P2B2|FPRP_HUMAN	0.16052818	1.0801278
sp|Q9Y281|COF2_HUMAN	0.1607151	1.1505735
sp|P15374|UCHL3_HUMAN	0.16147232	1.1276597
sp|P05388|RLA0_HUMAN	0.17217064	0.2178309
sp|Q9UJS0-2|CMC2_HUMAN	0.17376709	0
sp|Q07960|RHG01_HUMAN	0.1738739	1.0076748
sp|P35858-2|ALS_HUMAN	0.17431068	0.19149946
sp|O94776|MTA2_HUMAN	0.17557907	0.6298893
sp|P14618-2|KPYM_HUMAN	0.17583656	0.5204253
sp|P00740|FA9_HUMAN	0.1769085	0.312067

sp|Q5JRX3-3|PREP_HUMAN	0.18254471	0
sp|Q86WV6|STING_HUMAN	0.18442345	0.7061832
sp|P61803|DAD1_HUMAN	0.18490219	0.656254
sp|P53992|SC24C_HUMAN	0.18556213	0.40256184
sp|O76074-2|PDE5A_HUMAN	0.18687248	0.7061832
sp|O15061|SYNEM_HUMAN	0.18734169	0.7498006
sp|Q9C0E8-4|LNP_HUMAN	0.18767262	1.1932944
sp|Q5TZA2|CROCC_HUMAN	0.19128036	0.8983557
sp|Q7L5N1|CSN6_HUMAN	0.19210339	1.1932944
sp|P08708|RS17_HUMAN	0.19241714	1.120602
sp|P60891|PRPS1_HUMAN	0.19440079	0.656254
sp|P01034|CYTC_HUMAN	0.19847488	1.1932944
sp|P21399|ACOC_HUMAN	0.19892883	1.0645995
sp|P20039|2B1B_HUMAN	0.20091724	0.656254
sp|P50579-2|MAP2_HUMAN	0.20149422	0
sp|Q14767|LTBP2_HUMAN	0.20227432	0.95482355
sp|Q96HY6|DDRGK_HUMAN	0.20481491	0.656254
sp|P0DJI8|SAA1_HUMAN	0.20571136	0
sp|Q96P70|IPO9_HUMAN	0.2070179	0.21439649
sp|P06753-6|TPM3_HUMAN	0.21187401	0.656254
sp|P20810-4|ICAL_HUMAN	0.21310043	0.91601294
sp|O75323|NIPS2_HUMAN	0.21816444	0.45033538
sp|O00391|QSOX1_HUMAN	0.22060204	0
sp|P62829|RL23_HUMAN	0.2319336	0.7827403
sp|O95479|G6PE_HUMAN	0.23674202	0.7588735
sp|O60610-2|DIAP1_HUMAN	0.23854828	1.0485198
sp|Q15661|TRYB1_HUMAN	0.24377632	1.0475321
sp|P24666|PPAC_HUMAN	0.24435616	0.19149946
sp|P30613-2|KPYR_HUMAN	0.24459457	0.656254
sp|Q96HC4|PDLI5_HUMAN	0.2450962	0.656254
sp|Q5R3I4|TTC38_HUMAN	0.24586487	0
sp|P04062-2|GLCM_HUMAN	0.24596214	0.6070219
sp|Q66K74-2|MAP1S_HUMAN	0.25109863	1.1932944
sp|P36542|ATPG_HUMAN	0.25956917	0.656254
sp|Q9Y5Z4|HEBP2_HUMAN	0.26172066	0.2178309
sp|O75746-2|CMC1_HUMAN	0.26558304	0.7061832
sp|P62191-2|PRS4_HUMAN	0.27272987	0.35795313
sp|P42785-2|PCP_HUMAN	0.27282715	1.2773042
sp|Q9BZE9-2|ASPC1_HUMAN	0.2786274	0.656254
sp|Q9UNH7-2|SNX6_HUMAN	0.28132915	0.45033538
sp|Q99538-2|LGMN_HUMAN	0.28157043	0.19149946
sp|Q08379|GOGA2_HUMAN	0.28295898	0.19149946
sp|P16144-2|ITB4_HUMAN	0.2965479	0.7827403
sp|Q99598|TSNAX_HUMAN	0.29809475	0.7061832
sp|P54802|ANAG_HUMAN	0.3036642	0.19149946

sp|P01743|HV146_HUMAN	0.310503	0.656254
sp|P08779|K1C16_HUMAN	0.3129387	0.7827403
sp|P62269|RS18_HUMAN	0.31328583	0.6298893
sp|P11678|PERE_HUMAN	0.31446838	0.91601294
sp|Q08378|GOGA3_HUMAN	0.31825352	0.91601294
sp|Q7Z4H8|PLGT3_HUMAN	0.32453346	0.656254
sp|Q00341-2|VIGLN_HUMAN	0.32650375	1.2432404
sp|P35080-2|PROF2_HUMAN	0.32922935	0.656254
sp|P09012|SNRPA_HUMAN	0.3337078	0.19149946
sp|P62736|ACTA_HUMAN	0.33613014	0.656254
sp|P63027|VAMP2_HUMAN	0.34398842	1.1932944
sp|P02533|K1C14_HUMAN	0.3593397	0.45033538
sp|Q96HN2-2|SAHH3_HUMAN	0.36075783	0.656254
sp|P34896-2|GLYC_HUMAN	0.37167645	0.656254
sp|Q9UK22|FBX2_HUMAN	0.38382435	1.1932944
sp|P07951-3|TPM2_HUMAN	0.38465118	0.7588735
sp|P0CG38|POTEI_HUMAN	0.38873768	0.656254
sp|P63267|ACTH_HUMAN	0.3948784	0.656254
sp|O60825-2|F262_HUMAN	0.40759277	0.656254
sp|Q687X5|STEA4_HUMAN	0.4255829	0.7827403
sp|O14791-2|APOL1_HUMAN	0.42950058	1.1932944
sp|Q05682|CALD1_HUMAN	0.4320774	0.5204253
sp|P0DOX3|IGD_HUMAN	0.43770218	0.656254
sp|P05452|TETN_HUMAN	0.45760918	0.7061832
sp|P40261|NNMT_HUMAN	0.46194172	1.1505735
sp|A0A0C4DH31|HV118_HUMAN	0.47339058	0.656254
sp|Q9H6S3|ES8L2_HUMAN	0.48140907	0.7827403
sp|P12814-2|ACTN1_HUMAN	0.48213577	1.1932944
sp|P07358|CO8B_HUMAN	0.4863882	0.8983557
sp|Q92599-3|SEPT8_HUMAN	0.49816895	1.1932944
sp|O95302-3|FKBP9_HUMAN	0.50089264	1.1932944
sp|P40123-2|CAP2_HUMAN	0.5144787	0.656254
sp|P20339-2|RAB5A_HUMAN	0.5168266	0.656254
sp|P69891|HBG1_HUMAN	0.5176201	0.656254
sp|P14207|FOLR2_HUMAN	0.52077866	0.656254
sp|Q9NSK0-5|KLC4_HUMAN	0.5327072	0.656254
sp|Q7Z7G0|TARSH_HUMAN	0.536993	1.1932944
sp|O94788-3|AL1A2_HUMAN	0.54286957	0.656254
sp|Q99961-3|SH3G1_HUMAN	0.54821014	0.656254
sp|O43301|HS12A_HUMAN	0.5489216	1.0485198
sp|Q8TCD5|NT5C_HUMAN	0.5606575	1.1932944
sp|P07360|CO8G_HUMAN	0.5797806	1.1932944
sp|Q04695|K1C17_HUMAN	0.58003616	0.656254
sp|P23142-4|FBLN1_HUMAN	0.5899782	0.656254
sp|Q06033-2|ITIH3_HUMAN	0.59802246	0.7827403

sp|P28161|GSTM2_HUMAN	0.63067245	0.656254
sp|P19404|NDUV2_HUMAN	0.6472397	0.19149946
sp|A0A0C4DH29|HV103_HUMAN	0.6519718	0.656254
sp|P12111-4|CO6A3_HUMAN	0.65527153	0.656254
sp|O75964|ATP5L_HUMAN	0.65802383	1.1932944
sp|Q13976-2|KGP1_HUMAN	0.7385731	0.656254
sp|P47895|AL1A3_HUMAN	0.7514458	0.656254
sp|P09871|C1S_HUMAN	0.75982666	0.5204253
sp|P01591|IGJ_HUMAN	0.77490425	1.1932944
sp|Q9BTE3-2|MCMBP_HUMAN	0.77521515	0.45033538
sp|A0A0C4DH38|HV551_HUMAN	0.77970123	1.1932944
sp|Q06828|FMOD_HUMAN	0.7826252	1.1932944
sp|P08185|CBG_HUMAN	0.8255844	1.1932944
sp|P02461|CO3A1_HUMAN	0.8493881	1.1932944
sp|Q04446|GLGB_HUMAN	0.8618021	0.25789237
sp|O76011|KRT34_HUMAN	0.8685894	0.656254
sp|P55058|PLTP_HUMAN	0.8787508	1.1932944
sp|P02751-15|FINC_HUMAN	0.8891201	0.656254
sp|P01860|IGHG3_HUMAN	0.89302826	1.3006523
sp|A0A0B4J1X8|HV343_HUMAN	0.8977623	0.656254
sp|P01619|KV320_HUMAN	1.010294	0.656254
sp|P23083|HV102_HUMAN	1.0324707	0.656254
sp|Q96AY3|FKB10_HUMAN	1.0562553	1.1932944
sp|P05155-2|IC1_HUMAN	1.0744972	1.1932944
sp|Q15063-3|POSTN_HUMAN	1.0897789	1.1932944
sp|Q15063-2|POSTN_HUMAN	1.1104622	0.656254
sp|P35542|SAA4_HUMAN	1.1216011	1.1932944
sp|P02763|A1AG1_HUMAN	1.1613846	1.2773042
sp|Q15046|SYK_HUMAN	1.185154	0.656254
sp|P0C0L4|CO4A_HUMAN	1.1941061	1.1932944
sp|P02747|C1QC_HUMAN	1.2514343	1.1932944
sp|O94919|ENDD1_HUMAN	1.3012028	0
sp|P11166|GTR1_HUMAN	1.3800373	1.1932944
sp|P01782|HV309_HUMAN	1.3998756	0.656254
sp|P0C0L5|CO4B_HUMAN	1.4002514	1.1932944
sp|P48741|HSP77_HUMAN	1.4329243	0.656254
sp|P01624|KV315_HUMAN	1.4354477	1.1932944
sp|P02746|C1QB_HUMAN	1.5147228	1.1932944
sp|A0A0C4DH25|KVD20_HUMAN	1.6677189	0.656254
sp|P01834|IGKC_HUMAN	1.7494774	1.1932944
sp|P01780|HV307_HUMAN	1.7503128	0.656254
sp|P0DP03|HV335_HUMAN	1.7626495	1.1932944
sp|P69892|HBG2_HUMAN	1.7663689	0.656254
sp|P00738|HPT_HUMAN	1.9446831	0.656254
sp|P06310|KV230_HUMAN	1.9644623	0.656254

sp|Q03591|FHR1_HUMAN	2.2304182	1.1932944
sp|A0A0C4DH41|HV461_HUMAN	2.3162384	0.656254
sp|A0A075B6P5|KV228_HUMAN	2.5711517	0.656254
sp|O43790|KRT86_HUMAN	3.32802	0.656254
sp|Q15323|K1H1_HUMAN	3.9766417	0.656254
