# Supplementary material for: Peptidylarginine deiminase 2 citrullinates MZB1 and promotes the secretion of IgM and IgA
Source: Front Immunol. 2023 Nov 29;14:1290585. doi: 10.3389/fimmu.2023.1290585 (PMC10716219; doi:10.3389/fimmu.2023.1290585)
Supplement: Supplementary file 10 [file DataSheet_10.pdf]

Supplemental Table 10: COPD1 vs controls

Accession #	Fold Change	p value (-log10)
sp|P62805|H4_HUMAN	-0.645504	5.226002
sp|P84243|H33_HUMAN	-0.5726891	1.6960104
sp|P22748|CAH4_HUMAN	-0.2734909	1.5646582
sp|P02788|TRFL_HUMAN	-0.1877365	2.2798014
sp|P08311|CATG_HUMAN	0.19815445	1.3635377
sp|P05107|ITB2_HUMAN	0.2017746	1.4068714
sp|P10412|H14_HUMAN	0.21715736	1.6092666
sp|P02768|ALBU_HUMAN	0.21965408	7.8594766
sp|P0CG47|UBB_HUMAN	0.23016167	2.151766
sp|P05362|ICAM1_HUMAN	0.24691391	1.4832523
sp|Q16629-4|SRSF7_HUMAN	0.25896835	2.1818786
sp|P01903|DRA_HUMAN	0.2614441	1.9509854
sp|Q07157|ZO1_HUMAN	0.2658329	3.4304368
sp|Q9Y624|JAM1_HUMAN	0.26625824	2.6997027
sp|Q8NBQ5|DHB11_HUMAN	0.26987457	3.1266599
sp|P51149|RAB7A_HUMAN	0.27315903	1.9153107
sp|P11215-2|ITAM_HUMAN	0.2759266	1.3635377
sp|P42167-2|LAP2B_HUMAN	0.28160286	2.3047035
sp|Q15102|PA1B3_HUMAN	0.29572868	2.1818786
sp|P11233|RALA_HUMAN	0.29699707	1.4104178
sp|Q07955-2|SRSF1_HUMAN	0.3065014	4.220149
sp|P13647|K2C5_HUMAN	0.30781174	4.7039127
sp|Q9BVC6|TM109_HUMAN	0.3122921	1.6960104
sp|Q15907|RB11B_HUMAN	0.31320572	2.653196
sp|P09467|F16P1_HUMAN	0.3175106	1.9311212
sp|P01009|A1AT_HUMAN	0.32048607	3.3967228
sp|Q6NZI2|CAVN1_HUMAN	0.3204956	4.9172535
sp|P39019|RS19_HUMAN	0.32600212	1.6960104
sp|P51572-2|BAP31_HUMAN	0.33575058	1.4278674
sp|P05026-2|AT1B1_HUMAN	0.33698273	1.8444856
sp|P19013|K2C4_HUMAN	0.338459	2.4829872
sp|P07686|HEXB_HUMAN	0.34049225	3.2221727
sp|Q9UGT4|SUSD2_HUMAN	0.3407421	2.6997027
sp|Q03135|CAV1_HUMAN	0.3433628	1.3815327
sp|P26006-1|ITA3_HUMAN	0.3437538	3.0824287
sp|Q9H0U4|RAB1B_HUMAN	0.3440838	1.9647322
sp|P61106|RAB14_HUMAN	0.35243034	4.367871
sp|O75367-2|H2AY_HUMAN	0.35640144	3.4656596
sp|P10606|COX5B_HUMAN	0.35805893	1.7590232
sp|P02671-2|FIBA_HUMAN	0.35974884	3.3699222
sp|P08575-10|PTPRC_HUMA	0.36309242	2.2613342
sp|P20700|LMNB1_HUMAN	0.36699104	14.14841
sp|P24539|AT5F1_HUMAN	0.36839676	1.9997257

sp|P06753-2|TPM3_HUMAN	0.3707962	2.7060583
sp|O75955-2|FLOT1_HUMAN	0.3750782	2.4829872
sp|Q02818|NUCB1_HUMAN	0.37739372	2.7192829
sp|P05783|K1C18_HUMAN	0.3789425	5.7283993
sp|P49407-2|ARRB1_HUMAN	0.3814621	1.5567774
sp|P01011|AACT_HUMAN	0.38251495	9.004438
sp|P30740|ILEU_HUMAN	0.3827362	3.1822758
sp|P04179-4|SODM_HUMAN	0.383873	2.6576471
sp|Q14498-2|RBM39_HUMA	0.38721275	1.5646582
sp|P16284-3|PECA1_HUMAN	0.3881588	5.5660806
sp|Q9H299|SH3L3_HUMAN	0.38983536	1.4104178
sp|P07996|TSP1_HUMAN	0.39102554	2.6576471
sp|P0DP25|CALM3_HUMAN	0.39130783	2.7472508
sp|Q96AG4|LRC59_HUMAN	0.39413452	2.6576471
sp|Q9H223|EHD4_HUMAN	0.39499664	2.6514559
sp|Q02543|RL18A_HUMAN	0.39587593	2.6576471
sp|P29218|IMPA1_HUMAN	0.39597702	1.7713763
sp|P59998|ARPC4_HUMAN	0.39669037	1.9951487
sp|P61019|RAB2A_HUMAN	0.40132523	1.6960104
sp|P22307-8|NLTP_HUMAN	0.40190887	3.1266599
sp|P30048-2|PRDX3_HUMAN	0.40324402	2.3143692
sp|Q9NX63|MIC19_HUMAN	0.40501785	2.6576471
sp|Q96C19|EFHD2_HUMAN	0.40690613	1.9647322
sp|P62269|RS18_HUMAN	0.40931702	2.1818786
sp|P27105|STOM_HUMAN	0.41056824	3.364204
sp|Q9HDC9|APMAP_HUMAN	0.41270447	4.789934
sp|P22626|ROA2_HUMAN	0.41283226	6.044454
sp|P13646-3|K1C13_HUMAN	0.41877937	1.8444856
sp|P13073|COX41_HUMAN	0.42206383	1.9647322
sp|P31942-2|HNRH3_HUMAI	0.42249107	2.3143692
sp|P00491|PNPH_HUMAN	0.423357	3.417183
sp|Q9UDY2-3|ZO2_HUMAN	0.4244423	2.6554532
sp|P13804-2|ETFA_HUMAN	0.42605972	2.6134143
sp|Q99715-4|COCA1_HUMAI	0.4288864	1.6604291
sp|Q9H3N1|TMX1_HUMAN	0.43113136	1.4104178
sp|Q8NF37|PCAT1_HUMAN	0.4355812	1.827201
sp|P48735|IDHP_HUMAN	0.4360218	4.12427
sp|P04080|CYTB_HUMAN	0.43782043	1.6960104
sp|O14745|NHRF1_HUMAN	0.43815613	1.3561004
sp|P21399|ACOC_HUMAN	0.4389534	1.7713763
sp|Q9NP72-2|RAB18_HUMA	0.44081497	2.1818786
sp|Q13740-2|CD166_HUMAN	0.44129944	3.5838592
sp|P30040|ERP29_HUMAN	0.44153976	2.0727112
sp|P07305|H10_HUMAN	0.44421387	1.6960104
sp|Q99623|PHB2_HUMAN	0.44445038	4.044672

sp|P30050|RL12_HUMAN	0.4455185	2.6576471
sp|P84095|RHOG_HUMAN	0.44740105	1.6960104
sp|P35908|K22E_HUMAN	0.44848633	8.558914
sp|P02545|LMNA_HUMAN	0.44997597	3.658668
sp|P21796|VDAC1_HUMAN	0.4502697	5.161142
sp|Q86Y82|STX12_HUMAN	0.45149803	2.6576471
sp|Q96AE4-2|FUBP1_HUMA	0.45379066	3.2221727
sp|Q92542|NICA_HUMAN	0.4543724	1.6960104
sp|Q5TZA2|CROCC_HUMAN	0.4543743	1.8444856
sp|Q16698-2|DECR_HUMAN	0.45641708	4.964395
sp|P46781|RS9_HUMAN	0.45705414	3.5908275
sp|P40429|RL13A_HUMAN	0.45931244	1.6960104
sp|O15400-2|STX7_HUMAN	0.46050835	2.1818786
sp|P80303-2|NUCB2_HUMA	0.46074677	2.1818786
sp|Q8WXF1|PSPC1_HUMAN	0.4615326	1.6092666
sp|Q969H8|MYDGF_HUMAN	0.4620266	1.6960104
sp|P23246|SFPQ_HUMAN	0.46251488	5.161142
sp|P31146|COR1A_HUMAN	0.46338272	3.97461
sp|P35527|K1C9_HUMAN	0.46556282	4.964395
sp|P58107|EPIPL_HUMAN	0.46571732	3.760603
sp|P43652|AFAM_HUMAN	0.46614456	4.876761
sp|P23396|RS3_HUMAN	0.4665718	5.869831
sp|P01871-2|IGHM_HUMAN	0.46715546	1.7590232
sp|P62136|PP1A_HUMAN	0.46803665	1.6960104
sp|P51648-2|AL3A2_HUMAN	0.47057724	4.051346
sp|P69905|HBA_HUMAN	0.47094345	3.5908275
sp|P09601|HMOX1_HUMAN	0.4717865	1.6960104
sp|P02766|TTHY_HUMAN	0.4724083	2.1818786
sp|O75947-2|ATP5H_HUMA	0.4737358	2.6576471
sp|P62424|RL7A_HUMAN	0.4750023	4.126053
sp|P21397|AOFA_HUMAN	0.47767258	4.953735
sp|Q13185|CBX3_HUMAN	0.47772217	1.6960104
sp|P02649|APOE_HUMAN	0.47821426	5.407453
sp|Q15181|IPYR_HUMAN	0.4782524	1.6960104
sp|Q13308-6|PTK7_HUMAN	0.47880936	3.5908275
sp|P09525|ANXA4_HUMAN	0.47963905	3.5924494
sp|P62081|RS7_HUMAN	0.4798584	1.6960104
sp|O00264|PGRC1_HUMAN	0.4799137	2.6576471
sp|P56537|IF6_HUMAN	0.48107147	2.1818786
sp|P43304|GPDM_HUMAN	0.4813366	4.051346
sp|O75937|DNJC8_HUMAN	0.48171806	1.5646582
sp|Q02978|M2OM_HUMAN	0.4822216	3.1266599
sp|P23284|PPIB_HUMAN	0.48491096	4.509013
sp|P50914|RL14_HUMAN	0.4849968	1.6960104
sp|P22695|QCR2_HUMAN	0.48695946	4.051346

sp|Q00765|REEP5_HUMAN	0.48721695	1.515701
sp|P35754|GLRX1_HUMAN	0.48804855	2.1818786
sp|Q9P0L0-2|VAPA_HUMAN	0.48815155	2.8845065
sp|P15880|RS2_HUMAN	0.48837662	3.1266599
sp|Q14103-3|HNRPD_HUMA	0.48908806	4.575238
sp|P11310-2|ACADM_HUMA	0.4895172	4.7039127
sp|Q9Y277-2|VDAC3_HUMAI	0.4902954	2.6576471
sp|P39656-3|OST48_HUMAN	0.4912758	3.5908275
sp|P37837|TALDO_HUMAN	0.4914627	5.074809
sp|P62993|GRB2_HUMAN	0.4917946	2.1818786
sp|P18124|RL7_HUMAN	0.4922428	3.5908275
sp|Q12907|LMAN2_HUMAN	0.4936638	3.1266599
sp|P09110|THIK_HUMAN	0.49400902	3.5908275
sp|P35241|RADI_HUMAN	0.49432182	3.0251918
sp|P02549-2|SPTA1_HUMAN	0.49489594	7.5025163
sp|Q9UHB6-4|LIMA1_HUMA	0.49534416	1.9951487
sp|P02730|B3AT_HUMAN	0.49563217	4.285869
sp|P09874|PARP1_HUMAN	0.4957199	3.5908275
sp|P62316|SMD2_HUMAN	0.49572372	1.9647322
sp|Q9Y6N5|SQOR_HUMAN	0.49684715	4.2046685
sp|Q92817|EVPL_HUMAN	0.49723053	4.281241
sp|P10599-2|THIO_HUMAN	0.4973259	1.7590232
sp|P07237|PDIA1_HUMAN	0.4990883	11.579804
sp|P55268|LAMB2_HUMAN	0.49936104	12.717298
sp|P62906|RL10A_HUMAN	0.4996109	3.1266599
sp|P62820|RAB1A_HUMAN	0.5011997	2.6576471
sp|Q9H4M9|EHD1_HUMAN	0.50185776	3.218121
sp|B5ME19|EIFCL_HUMAN	0.5020237	2.5461748
sp|Q14344|GNA13_HUMAN	0.50219345	2.1818786
sp|P00492|HPRT_HUMAN	0.5024586	2.1818786
sp|P52272-2|HNRPM_HUMA	0.5027504	7.7524443
sp|Q9UL25|RAB21_HUMAN	0.50395584	1.9647322
sp|Q13636|RAB31_HUMAN	0.5043316	1.6960104
sp|Q8NBJ5|GT251_HUMAN	0.5044956	1.6960104
sp|P43490|NAMPT_HUMAN	0.50460434	6.3204336
sp|O96009|NAPSA_HUMAN	0.50478745	2.6576471
sp|P0DOX8|IGL1_HUMAN	0.50506973	2.6576471
sp|P27797|CALR_HUMAN	0.5057144	4.509013
sp|P29350-4|PTN6_HUMAN	0.5063534	1.8838416
sp|P01861|IGHG4_HUMAN	0.507246	2.4829872
sp|Q15293|RCN1_HUMAN	0.5095215	1.6960104
sp|P38919|IF4A3_HUMAN	0.5095272	4.051346
sp|P26373|RL13_HUMAN	0.5105858	2.151766
sp|P61353|RL27_HUMAN	0.51122284	1.6960104
sp|Q15365|PCBP1_HUMAN	0.5112686	2.8739974

sp|P35232|PHB_HUMAN	0.51138306	5.417897
sp|P60953|CDC42_HUMAN	0.5119724	3.7273152
sp|P11047|LAMC1_HUMAN	0.51264954	8.931878
sp|Q9NNW7|TRXR2_HUMAN	0.5126629	2.1818786
sp|E9PAV3|NACAM_HUMAN	0.5128212	2.1818786
sp|Q9Y230|RUVB2_HUMAN	0.5131397	2.894491
sp|P10253|LYAG_HUMAN	0.51372147	3.5908275
sp|O75396|SC22B_HUMAN	0.5138855	3.1266599
sp|P50213|IDH3A_HUMAN	0.51390266	1.6960104
sp|Q9C0C2-2|TB182_HUMAN	0.5139675	2.1818786
sp|P36543-2|VATE1_HUMAN	0.51405525	1.6960104
sp|P02774-3|VTDB_HUMAN	0.51499176	8.558914
sp|Q00325-2|MPCP_HUMAN	0.5162716	1.9951487
sp|O95571|ETHE1_HUMAN	0.5177746	2.36833
sp|O60437|PEPL_HUMAN	0.5184536	14.539617
sp|Q05655-2|KPCD_HUMAN	0.5193424	1.4104178
sp|Q969V3-2|NCLN_HUMAN	0.5204296	2.1818786
sp|P04264|K2C1_HUMAN	0.5206814	12.996504
sp|Q14254|FLOT2_HUMAN	0.5214653	4.509013
sp|P26599-2|PTBP1_HUMAN	0.5219536	2.6576471
sp|Q6YHK3-4|CD109_HUMAN	0.52228165	1.6960104
sp|P48047|ATPO_HUMAN	0.5226078	3.9417877
sp|Q9P258|RCC2_HUMAN	0.5232601	2.6576471
sp|Q9UBS4|DJB11_HUMAN	0.5242386	1.6960104
sp|P02042|HBD_HUMAN	0.52505875	3.5908275
sp|P16278-2|BGAL_HUMAN	0.525177	1.4104178
sp|P10809|CH60_HUMAN	0.52575684	9.952936
sp|Q6P587-3|FAHD1_HUMAN	0.52591133	1.6960104
sp|Q8WUY1|THEM6_HUMAN	0.52706146	1.6960104
sp|P30533|AMRP_HUMAN	0.528738	1.6960104
sp|Q13162|PRDX4_HUMAN	0.52876663	3.1266599
sp|Q03252|LMNB2_HUMAN	0.52978134	14.55665
sp|P61981|1433G_HUMAN	0.53035927	4.051346
sp|Q96TA1-2|NIBL1_HUMAN	0.53053856	2.9807727
sp|Q9UKV3-5|ACINU_HUMA	0.5315323	2.1818786
sp|Q7Z406-2|MYH14_HUMA	0.5325165	11.224656
sp|O96000-2|NDUBA_HUMA	0.5328407	1.6960104
sp|Q9BS40|LXN_HUMAN	0.5329399	1.4104178
sp|Q92882|OSTF1_HUMAN	0.53307533	1.3815327
sp|O15230|LAMA5_HUMAN	0.53336334	5.417897
sp|Q8WU39|MZB1_HUMAN	0.5344353	1.6960104
sp|Q9BS26|ERP44_HUMAN	0.53549576	3.1266599
sp|P19338|NUCL_HUMAN	0.5358162	9.004438
sp|P28066|PSA5_HUMAN	0.53593826	1.6960104
sp|P35268|RL22_HUMAN	0.5359745	1.5646582

sp|P26038|MOES_HUMAN	0.53599167	10.066691
sp|P14618-3|KPYM_HUMAN	0.5360184	2.6576471
sp|Q9UFN0|NPS3A_HUMAN	0.5360851	1.6960104
sp|O75489|NDUS3_HUMAN	0.53658295	1.6960104
sp|P05388-2|RLA0_HUMAN	0.538023	3.5908275
sp|Q9NTK5|OLA1_HUMAN	0.538126	2.1818786
sp|P25398|RS12_HUMAN	0.539732	4.051346
sp|P04196|HRG_HUMAN	0.5399437	3.5908275
sp|P09871|C1S_HUMAN	0.5399895	2.1818786
sp|Q5SSJ5-2|HP1B3_HUMAN	0.54011536	3.5908275
sp|P40926|MDHM_HUMAN	0.54146004	8.413603
sp|Q96RQ3|MCCA_HUMAN	0.5419979	2.1818786
sp|P51649-2|SSDH_HUMAN	0.5423889	2.1818786
sp|P51148-2|RAB5C_HUMAN	0.5438843	1.8444856
sp|P61978-3|HNRPK_HUMAN	0.5449772	7.3098264
sp|O00231-2|PSD11_HUMAN	0.54504204	3.1266599
sp|P61421|VA0D1_HUMAN	0.5454807	3.1266599
sp|Q9UHG3|PCYOX_HUMAN	0.5455227	5.869831
sp|Q14165|MLEC_HUMAN	0.5462818	1.6960104
sp|O15511|ARPC5_HUMAN	0.5465431	1.4716977
sp|P31040|SDHA_HUMAN	0.54849243	4.509013
sp|P06733|ENOA_HUMAN	0.5488663	8.815352
sp|P02675|FIBB_HUMAN	0.5492821	6.57528
sp|P19971|TYPH_HUMAN	0.5493641	8.061325
sp|P09497-2|CLCB_HUMAN	0.5496559	1.6960104
sp|P22392-2|NDKB_HUMAN	0.550333	4.509013
sp|Q8N392|RHG18_HUMAN	0.5506935	1.9951487
sp|P52597|HNRPF_HUMAN	0.5507927	2.6576471
sp|Q13148|TADBP_HUMAN	0.550869	1.6960104
sp|P05141|ADT2_HUMAN	0.5509529	2.6576471
sp|P52566|GDIR2_HUMAN	0.5520115	2.3388627
sp|Q14112-2|NID2_HUMAN	0.55205154	3.5908275
sp|P04004|VTNC_HUMAN	0.5523243	4.051346
sp|Q06323|PSME1_HUMAN	0.5526562	2.7472508
sp|Q9BTZ2|DHRS4_HUMAN	0.55366135	1.6960104
sp|P46782|RS5_HUMAN	0.55391693	3.1266599
sp|P42765|THIM_HUMAN	0.55431366	8.112776
sp|P48449-3|ERG7_HUMAN	0.5545769	2.6576471
sp|P63244|RACK1_HUMAN	0.55495834	4.126053
sp|P17844-2|DDX5_HUMAN	0.5553894	3.7161493
sp|P07203|GPX1_HUMAN	0.55545616	4.051346
sp|O15247|CLIC2_HUMAN	0.5557289	2.9807727
sp|P46777|RL5_HUMAN	0.55592346	4.797763
sp|Q08945|SSRP1_HUMAN	0.5562229	2.6576471
sp|Q86UX7-2|URP2_HUMAN	0.55657005	5.180782

sp|Q9HB07|MYG1_HUMAN	0.5576019	1.6960104
sp|Q8N1G4|LRC47_HUMAN	0.5576439	2.432837
sp|Q13724-2|MOGS_HUMAN	0.55854225	2.1818786
sp|P62266|RS23_HUMAN	0.5590992	1.6960104
sp|Q96HY6|DDRGK_HUMAN	0.559782	1.6604291
sp|P02750|A2GL_HUMAN	0.56027985	1.8444856
sp|Q9Y224|RTRAF_HUMAN	0.5606289	2.6576471
sp|P62258|1433E_HUMAN	0.5614548	8.112776
sp|Q15661|TRYB1_HUMAN	0.5614815	4.575238
sp|Q08380|LG3BP_HUMAN	0.5617676	3.5908275
sp|Q53GQ0|DHB12_HUMAN	0.56193733	2.6576471
sp|P52788|SPSY_HUMAN	0.56204605	1.6960104
sp|Q00796|DHSO_HUMAN	0.56318283	1.6960104
sp|P23528|COF1_HUMAN	0.5639572	4.789934
sp|Q5T440|CAF17_HUMAN	0.5654068	1.6960104
sp|Q13217|DNJC3_HUMAN	0.5654354	2.1818786
sp|P53597|SUCA_HUMAN	0.56622887	2.1818786
sp|Q92896-2|GSLG1_HUMAN	0.5662842	1.9190748
sp|P99999|CYC_HUMAN	0.5664482	2.6576471
sp|P61160|ARP2_HUMAN	0.5672989	5.417897
sp|P20073-2|ANXA7_HUMAN	0.5673485	4.876761
sp|Q9NQC3|RTN4_HUMAN	0.5673599	2.6576471
sp|Q9Y3D6|FIS1_HUMAN	0.5678234	1.6960104
sp|Q9NUV9|GIMA4_HUMAN	0.5681	2.6576471
sp|Q12931-2|TRAP1_HUMAN	0.56827164	1.7713763
sp|P11277-3|SPTB1_HUMAN	0.56983566	5.417897
sp|P06576|ATPB_HUMAN	0.57029724	9.449409
sp|P06748-3|NPM_HUMAN	0.5715256	1.9647322
sp|P49755|TMEDA_HUMAN	0.5720215	2.432837
sp|P26440|IVD_HUMAN	0.57229805	2.1818786
sp|P25705|ATPA_HUMAN	0.5724182	12.553017
sp|O75915|PRAF3_HUMAN	0.5724983	2.1818786
sp|Q9Y265|RUVB1_HUMAN	0.5725708	2.56956
sp|O15143|ARC1B_HUMAN	0.5726471	1.6644778
sp|P61586|RHOA_HUMAN	0.57292366	1.6960104
sp|Q02252-2|MMSA_HUMAN	0.57292557	4.4116287
sp|Q8IV08|PLD3_HUMAN	0.57328796	2.6576471
sp|P27635|RL10_HUMAN	0.5734062	1.6960104
sp|O00483|NDUA4_HUMAN	0.57421494	1.6960104
sp|P21912|SDHB_HUMAN	0.5747833	1.6960104
sp|P27824-2|CALX_HUMAN	0.5760422	4.876761
sp|P00505|AATM_HUMAN	0.5763359	5.869831
sp|P52907|CAZA1_HUMAN	0.5765114	3.5908275
sp|P01876|IGHA1_HUMAN	0.5766754	4.051346
sp|Q13011|ECH1_HUMAN	0.5771637	4.051346

sp|P41091|IF2G_HUMAN	0.57748795	2.6576471
sp|P13645|K1C10_HUMAN	0.5784073	9.8522625
sp|O75323|NIPS2_HUMAN	0.57853127	1.6960104
sp|P01042-2|KNG1_HUMAN	0.5790367	6.7698927
sp|P25685-2|DNJB1_HUMAN	0.5795078	1.6960104
sp|P08621-2|RU17_HUMAN	0.5796776	2.6576471
sp|P49257|LMAN1_HUMAN	0.58042717	2.1818786
sp|P60228|EIF3E_HUMAN	0.58052826	1.5646582
sp|O60256-3|KPRB_HUMAN	0.5809097	2.1818786
sp|Q6NUK1-2|SCMC1_HUMA	0.58109283	4.051346
sp|P68871|HBB_HUMAN	0.5817814	4.051346
sp|P14868|SYDC_HUMAN	0.5819874	4.964395
sp|P01008|ANT3_HUMAN	0.5823097	6.7073655
sp|P13667|PDIA4_HUMAN	0.58231163	9.004438
sp|Q9BTV4|TMM43_HUMAN	0.5825043	4.051346
sp|P02679-2|FIBG_HUMAN	0.58276176	4.9297023
sp|Q9Y266|NUDC_HUMAN	0.5841236	1.6960104
sp|P08648|ITA5_HUMAN	0.5857048	1.6960104
sp|Q99714|HCD2_HUMAN	0.58727455	4.509013
sp|O60716-14|CTND1_HUMA	0.5878258	2.5461748
sp|P00367|DHE3_HUMAN	0.58855057	4.044672
sp|P30044|PRDX5_HUMAN	0.5886402	4.3225203
sp|Q9Y2S2|CRYL1_HUMAN	0.5890465	2.1818786
sp|Q9HCC0-2|MCCB_HUMAN	0.58919334	2.9807727
sp|Q9NYL9|TMOD3_HUMAN	0.5898762	2.6576471
sp|P06865|HEXA_HUMAN	0.5904026	2.1818786
sp|Q07065|CKAP4_HUMAN	0.59183884	11.15183
sp|P12081-4|SYHC_HUMAN	0.5932064	3.1266599
sp|O14828-2|SCAM3_HUMA	0.59405327	1.6960104
sp|Q9NZT2-2|OGFR_HUMAN	0.5945816	1.6960104
sp|O00233-2|PSMD9_HUMA	0.5950451	1.6960104
sp|P36578|RL4_HUMAN	0.5967045	4.964395
sp|Q9H8H3|MET7A_HUMAN	0.5970459	1.6960104
sp|P49748-2|ACADV_HUMAN	0.59716606	12.593429
sp|P04899-3|GNAI2_HUMAN	0.59799194	4.4116287
sp|P35914|HMGCL_HUMAN	0.5989227	1.6960104
sp|P63241|IF5A1_HUMAN	0.59970474	2.1818786
sp|P09382|LEG1_HUMAN	0.59970856	4.509013
sp|Q9UPQ0-3|LIMC1_HUMA	0.59991455	2.432837
sp|P39060-1|COIA1_HUMAN	0.60022926	4.051346
sp|Q9NR45|SIAS_HUMAN	0.6006603	4.051346
sp|P34897-3|GLYM_HUMAN	0.6009598	2.1818786
sp|P14314-2|GLU2B_HUMAN	0.6011257	3.5908275
sp|Q07812-2|BAX_HUMAN	0.6011734	2.1818786
sp|Q16363-2|LAMA4_HUMA	0.6013851	4.991322

sp|P28072|PSB6_HUMAN	0.6018009	1.6960104
sp|P48444|COPD_HUMAN	0.6027355	6.253077
sp|P35237|SPB6_HUMAN	0.603281	7.665951
sp|P25786-2|PSA1_HUMAN	0.6035862	5.869831
sp|P07741|APT_HUMAN	0.6039238	4.051346
sp|P62826|RAN_HUMAN	0.6045246	2.8384566
sp|P04406|G3P_HUMAN	0.6045761	9.198758
sp|P62917|RL8_HUMAN	0.60471725	1.9190748
sp|P40306|PSB10_HUMAN	0.605072	1.6960104
sp|Q06830|PRDX1_HUMAN	0.6051216	5.7968407
sp|O95831-3|AIFM1_HUMAN	0.60578346	5.417897
sp|P50148|GNAQ_HUMAN	0.6065388	2.1818786
sp|Q96CX2|KCD12_HUMAN	0.6065407	6.253077
sp|Q9UH65|SWP70_HUMAN	0.6072521	2.6576471
sp|P13796|PLSL_HUMAN	0.60726166	10.554664
sp|P50454|SERPH_HUMAN	0.60736847	3.4656596
sp|O00423-3|EMAL1_HUMA	0.60803986	1.6960104
sp|P17174|AATC_HUMAN	0.60811234	4.051346
sp|P33121-3|ACSL1_HUMAN	0.6083069	3.1266599
sp|Q9UIJ7|KAD3_HUMAN	0.6083622	2.1818786
sp|Q9P2E9|RRBP1_HUMAN	0.6088295	12.996504
sp|P49961|ENTP1_HUMAN	0.6090355	2.6576471
sp|P62879|GBB2_HUMAN	0.60904694	2.6576471
sp|O75390|CISY_HUMAN	0.6090927	5.417897
sp|P24534|EF1B_HUMAN	0.6091614	1.6960104
sp|O95833|CLIC3_HUMAN	0.60972214	2.6576471
sp|Q15084-5|PDIA6_HUMAN	0.60978127	5.417897
sp|Q5TDH0-3|DDI2_HUMAN	0.610548	2.1818786
sp|O60832-2|DKC1_HUMAN	0.6111107	1.6960104
sp|P60900|PSA6_HUMAN	0.6124325	1.6960104
sp|O75439|MPPB_HUMAN	0.61270905	1.6960104
sp|Q9UKK9|NUDT5_HUMAN	0.6128788	1.6960104
sp|P39687|AN32A_HUMAN	0.6132088	2.1818786
sp|P19823|ITIH2_HUMAN	0.6134949	5.869831
sp|Q9P2T1-2|GMPR2_HUMA	0.6145477	1.6960104
sp|Q06787-10|FMR1_HUMA	0.6149616	1.6960104
sp|P02652|APOA2_HUMAN	0.61579895	3.1266599
sp|Q04917|1433F_HUMAN	0.61706734	4.964395
sp|P50990|TCPQ_HUMAN	0.61714554	7.592264
sp|P23381|SYWC_HUMAN	0.6172848	7.2183566
sp|P49821-2|NDUV1_HUMA	0.61792946	4.051346
sp|P11717|MPRI_HUMAN	0.6182823	1.6960104
sp|Q9H0D6|XRN2_HUMAN	0.6186657	1.9647322
sp|P10909-5|CLUS_HUMAN	0.6189747	4.509013
sp|Q14624-2|ITIH4_HUMAN	0.6196327	7.2183566

sp|P38646|GRP75_HUMAN	0.62060356	9.449409
sp|Q14019|COTL1_HUMAN	0.62073517	2.3047035
sp|Q86WV6|STING_HUMAN	0.6215553	1.6960104
sp|P62330|ARF6_HUMAN	0.62189674	1.6960104
sp|P49593|PPM1F_HUMAN	0.6227474	1.7590232
sp|O75306-2|NDUS2_HUMA	0.6231842	1.6960104
sp|P23368|MAOM_HUMAN	0.6239395	2.6576471
sp|O95834|EMAL2_HUMAN	0.62428284	4.051346
sp|O00159|MYO1C_HUMAN	0.6250229	8.804842
sp|P04424-2|ARLY_HUMAN	0.6255188	2.6576471
sp|P52565|GDIR1_HUMAN	0.62556267	3.218121
sp|P54727|RD23B_HUMAN	0.6260433	2.1818786
sp|P05091|ALDH2_HUMAN	0.62701225	11.236403
sp|P78417|GSTO1_HUMAN	0.6275425	4.797763
sp|Q08722-2|CD47_HUMAN	0.6277504	1.6960104
sp|P28062-2|PSB8_HUMAN	0.6278076	2.1818786
sp|P62191-2|PRS4_HUMAN	0.62786865	2.1818786
sp|O00116|ADAS_HUMAN	0.6279335	2.1818786
sp|P61247|RS3A_HUMAN	0.62903595	4.051346
sp|P15170-2|ERF3A_HUMAN	0.629076	1.6960104
sp|Q14258|TRI25_HUMAN	0.62926674	4.509013
sp|P63104|1433Z_HUMAN	0.62960815	7.665951
sp|P61254|RL26_HUMAN	0.6296272	2.1818786
sp|Q9NTX5-3|ECHD1_HUMA	0.62979126	3.5908275
sp|P51659|DHB4_HUMAN	0.63007355	2.1818786
sp|Q9UNZ2-5|NSF1C_HUMA	0.631073	3.5908275
sp|P0DOY2|IGLC2_HUMAN	0.63145065	2.1818786
sp|P53634|CATC_HUMAN	0.63160324	2.1818786
sp|P25311|ZA2G_HUMAN	0.6324711	4.051346
sp|Q9H2G2-2|SLK_HUMAN	0.63347244	1.9647322
sp|P02790|HEMO_HUMAN	0.63357544	9.004438
sp|Q13838-2|DX39B_HUMAN	0.63383484	3.1266599
sp|P10644|KAP0_HUMAN	0.63391876	4.051346
sp|P09211|GSTP1_HUMAN	0.6351738	5.2991114
sp|O43837|IDH3B_HUMAN	0.63518906	1.6960104
sp|P60983|GMFB_HUMAN	0.63550186	1.6960104
sp|P55327-3|TPD52_HUMAN	0.63554955	1.6960104
sp|Q9UJ70-2|NAGK_HUMAN	0.63573074	2.6576471
sp|P62873|GBB1_HUMAN	0.63620186	3.1266599
sp|P50579-2|MAP2_HUMAN	0.63628197	1.6960104
sp|P14923|PLAK_HUMAN	0.6370735	2.6576471
sp|Q96AQ6-2|PBIP1_HUMAN	0.6372051	2.6576471
sp|O00299|CLIC1_HUMAN	0.6372223	7.2183566
sp|Q9NZ01|TECR_HUMAN	0.6374035	2.1818786
sp|Q99439|CNN2_HUMAN	0.63760185	2.6576471

sp|P61158|ARP3_HUMAN	0.6376133	10.337908
sp|O43747-2|AP1G1_HUMAI	0.637764	2.1818786
sp|P08865|RSSA_HUMAN	0.6377926	4.964395
sp|P04844|RPN2_HUMAN	0.63783455	2.6576471
sp|P45880-2|VDAC2_HUMAN	0.63786316	5.417897
sp|P04003|C4BPA_HUMAN	0.63809204	4.964395
sp|P07942|LAMB1_HUMAN	0.6391678	5.417897
sp|Q9UL46|PSME2_HUMAN	0.63981056	3.5908275
sp|Q9NZM1-3|MYOF_HUMA	0.63991547	8.958427
sp|P09622|DLDH_HUMAN	0.6400833	4.964395
sp|P23786|CPT2_HUMAN	0.6408348	3.5908275
sp|P84077|ARF1_HUMAN	0.64087105	2.1818786
sp|O00567|NOP56_HUMAN	0.64115334	2.1818786
sp|O00391|QSOX1_HUMAN	0.64144325	2.1818786
sp|O94905|ERLN2_HUMAN	0.6420803	1.6960104
sp|P00747|PLMN_HUMAN	0.64247704	6.3204336
sp|P29590|PML_HUMAN	0.6425781	8.112776
sp|P14543-2|NID1_HUMAN	0.64289284	8.720673
sp|Q15233|NONO_HUMAN	0.6430359	4.12427
sp|Q13751|LAMB3_HUMAN	0.64344215	2.1818786
sp|P62701|RS4X_HUMAN	0.64356804	2.1818786
sp|Q9UHQ9|NB5R1_HUMAN	0.6436291	2.1818786
sp|P11940-2|PABP1_HUMAN	0.6451397	3.5908275
sp|Q14152-2|EIF3A_HUMAN	0.6455364	3.1266599
sp|P26641|EF1G_HUMAN	0.6456337	6.3204336
sp|P04843|RPN1_HUMAN	0.646328	6.6106963
sp|P69891|HBG1_HUMAN	0.64655495	2.6576471
sp|P30085|KCY_HUMAN	0.6469841	1.6960104
sp|Q7L2H7|EIF3M_HUMAN	0.6472168	1.6960104
sp|P04075|ALDOA_HUMAN	0.64793015	9.893875
sp|O75436|VP26A_HUMAN	0.6487503	2.1818786
sp|O15372|EIF3H_HUMAN	0.64888954	1.6960104
sp|P52790|HXK3_HUMAN	0.64907455	3.1266599
sp|Q96TC7|RMD3_HUMAN	0.6493225	2.6576471
sp|P21964-2|COMT_HUMAN	0.6499138	3.760603
sp|P61020|RAB5B_HUMAN	0.650795	1.6960104
sp|O43491|E41L2_HUMAN	0.6512642	3.5190198
sp|P14625|ENPL_HUMAN	0.6512661	11.874758
sp|Q13347|EIF3I_HUMAN	0.65156555	1.6960104
sp|P07988|PSPB_HUMAN	0.6530094	4.367871
sp|A0A0B4J2D5|GAL3B_HUN	0.65353394	2.1818786
sp|Q02218-2|ODO1_HUMAN	0.6540756	5.417897
sp|P40227|TCPZ_HUMAN	0.6546383	5.869831
sp|O15127|SCAM2_HUMAN	0.6554146	1.6960104
sp|P08758|ANXA5_HUMAN	0.6556225	11.224656

sp|P25787|PSA2_HUMAN	0.6557236	4.051346
sp|P20339-2|RAB5A_HUMAN	0.6563511	1.6960104
sp|P61970|NTF2_HUMAN	0.65636826	1.6960104
sp|Q00341|VIGLN_HUMAN	0.6565628	4.051346
sp|P31930|QCR1_HUMAN	0.6567955	4.964395
sp|P20618|PSB1_HUMAN	0.65690804	3.1266599
sp|Q5JRX3-2|PREP_HUMAN	0.6571808	1.6960104
sp|P37802|TAGL2_HUMAN	0.65758896	5.5367417
sp|P08708|RS17_HUMAN	0.657959	2.1818786
sp|P25788-2|PSA3_HUMAN	0.6589737	3.1266599
sp|Q6NY19-2|KANK3_HUMA	0.65979385	1.6960104
sp|P49354|FNTA_HUMAN	0.66002655	1.6960104
sp|O15382|BCAT2_HUMAN	0.6601906	2.1818786
sp|Q9Y5X3|SNX5_HUMAN	0.6604061	3.1266599
sp|O60664-4|PLIN3_HUMAN	0.6604786	1.3080103
sp|Q9Y394-2|DHRS7_HUMAI	0.6615429	2.1818786
sp|P47756-2|CAPZB_HUMAN	0.6618519	5.7968407
sp|O00764-2|PDXK_HUMAN	0.66199017	3.5908275
sp|Q9Y315|DEOC_HUMAN	0.6621723	1.6960104
sp|P43243|MATR3_HUMAN	0.6622181	4.051346
sp|P62241|RS8_HUMAN	0.6623688	2.6576471
sp|P32119|PRDX2_HUMAN	0.66348076	4.964395
sp|Q13492-2|PICAL_HUMAN	0.6640644	1.6960104
sp|P14618-2|KPYM_HUMAN	0.6647606	3.1266599
sp|P50552|VASP_HUMAN	0.6647606	4.051346
sp|P04217|A1BG_HUMAN	0.6649189	4.964395
sp|O00303|EIF3F_HUMAN	0.66509247	2.1818786
sp|O14786|NRP1_HUMAN	0.66529846	1.6960104
sp|P07737|PROF1_HUMAN	0.66542625	6.4343157
sp|Q99536|VAT1_HUMAN	0.66566086	4.509013
sp|Q12906-2|ILF3_HUMAN	0.66620636	7.2183566
sp|P48681|NEST_HUMAN	0.66698074	6.6106963
sp|O15144|ARPC2_HUMAN	0.6672554	4.509013
sp|O60547-2|GMDS_HUMAN	0.6679077	2.1818786
sp|P62913|RL11_HUMAN	0.6679878	1.6960104
sp|P05455|LA_HUMAN	0.6685009	4.509013
sp|Q9UHX1-2|PUF60_HUMA	0.6696663	1.6960104
sp|Q00577|PURA_HUMAN	0.66973495	2.1818786
sp|P19827|ITIH1_HUMAN	0.670084	3.1266599
sp|P27169|PON1_HUMAN	0.6712704	1.6960104
sp|Q8NBJ7|SUMF2_HUMAN	0.6717377	2.1818786
sp|O43852-5|CALU_HUMAN	0.672102	1.6960104
sp|P02647|APOA1_HUMAN	0.6724167	13.420564
sp|P30519|HMOX2_HUMAN	0.6726227	2.1818786
sp|P55809|SCOT1_HUMAN	0.6733322	2.1818786

sp|P28331-3|NDUS1_HUMAI	0.6733608	3.1266599
sp|Q8WVM8|SCFD1_HUMAI	0.67386246	2.1818786
sp|O75340-2|PDCD6_HUMAI	0.6746063	1.6960104
sp|Q9Y262|EIF3L_HUMAN	0.67466545	4.509013
sp|P47897|SYQ_HUMAN	0.67484665	2.6576471
sp|Q9NZN4|EHD2_HUMAN	0.6748562	9.893875
sp|Q04637-4|IF4G1_HUMAN	0.6751785	2.1818786
sp|P11171-2|41_HUMAN	0.6762314	1.6960104
sp|P13693|TCTP_HUMAN	0.676466	1.6960104
sp|P00751|CFAB_HUMAN	0.676651	12.553017
sp|P35573|GDE_HUMAN	0.6772232	1.6960104
sp|P55084|ECHB_HUMAN	0.678566	5.417897
sp|P00915|CAH1_HUMAN	0.67910767	6.3204336
sp|Q9BUJ2-4|HNRL1_HUMA	0.6795311	3.1266599
sp|O95202|LETM1_HUMAN	0.6798382	2.1818786
sp|Q13177|PAK2_HUMAN	0.6798935	2.1818786
sp|P28070|PSB4_HUMAN	0.6801014	2.6576471
sp|Q6YN16|HSDL2_HUMAN	0.6801386	3.5908275
sp|Q9BWM7|SFXN3_HUMAI	0.68032074	2.1818786
sp|P21281|VATB2_HUMAN	0.68114376	4.964395
sp|P16435|NCPR_HUMAN	0.6813946	6.3204336
sp|P04040|CATA_HUMAN	0.68141174	10.743512
sp|P02462|CO4A1_HUMAN	0.6825161	2.6576471
sp|P23142|FBLN1_HUMAN	0.6829376	2.4829872
sp|P09917-3|LOX5_HUMAN	0.6829529	1.6960104
sp|P42126-2|ECI1_HUMAN	0.6829796	1.6960104
sp|O75874|IDHC_HUMAN	0.68301773	5.0928617
sp|Q10567-2|AP1B1_HUMAI	0.68323517	2.1818786
sp|P24666-2|PPAC_HUMAN	0.6840668	1.6960104
sp|Q13884|SNTB1_HUMAN	0.68449783	1.6960104
sp|P54920|SNAA_HUMAN	0.68450165	4.964395
sp|Q9UMS4|PRP19_HUMAN	0.68475914	1.6960104
sp|P84098|RL19_HUMAN	0.6853695	1.6960104
sp|O60504-2|VINEX_HUMAN	0.68574715	1.6960104
sp|P26196|DDX6_HUMAN	0.68639183	1.6960104
sp|Q16658|FSCN1_HUMAN	0.68821526	6.3204336
sp|Q92598-2|HS105_HUMAI	0.6883888	2.7624686
sp|P25789|PSA4_HUMAN	0.68867874	3.1266599
sp|O75695|XRP2_HUMAN	0.6891651	1.6960104
sp|P05023-4|AT1A1_HUMAN	0.6893387	9.893875
sp|O14818|PSA7_HUMAN	0.68961906	2.6576471
sp|Q15393|SF3B3_HUMAN	0.68972206	5.869831
sp|Q9UJU6-2|DBNL_HUMAN	0.6912689	2.1818786
sp|Q92945|FUBP2_HUMAN	0.69148254	4.051346
sp|P05198|IF2A_HUMAN	0.6918869	2.1818786

sp|P26368-2|U2AF2_HUMAN	0.69213676	1.6960104
sp|P01023|A2MG_HUMAN	0.6921902	14.423111
sp|Q15691|MARE1_HUMAN	0.69246864	3.5908275
sp|Q06136|KDSR_HUMAN	0.69257164	2.1818786
sp|P15144|AMPN_HUMAN	0.6926975	2.1818786
sp|P16615|AT2A2_HUMAN	0.69296646	4.575238
sp|P67936|TPM4_HUMAN	0.692997	4.509013
sp|Q5JWF2-2|GNAS1_HUMA	0.6938591	2.6576471
sp|P04114|APOB_HUMAN	0.69519806	12.996504
sp|P12111|CO6A3_HUMAN	0.6957321	4.2451677
sp|O14579|COPE_HUMAN	0.69584656	3.5908275
sp|P02749|APOH_HUMAN	0.69628143	3.1266599
sp|P07738|PMGE_HUMAN	0.6978836	2.1818786
sp|Q1KMD3|HNRL2_HUMAN	0.6989536	6.3204336
sp|P50502|F10A1_HUMAN	0.6994648	2.151766
sp|Q9BTW9-4|TBCD_HUMAN	0.69997406	1.6960104
sp|O43390-2|HNRPR_HUMA	0.7002754	5.869831
sp|Q92597|NDRG1_HUMAN	0.7014408	2.1818786
sp|Q9ULA0|DNPEP_HUMAN	0.7023983	4.051346
sp|P38606-2|VATA_HUMAN	0.70370865	5.417897
sp|P30043|BLVRB_HUMAN	0.70384216	2.1818786
sp|Q9HC35-2|EMAL4_HUMA	0.70405006	4.509013
sp|P27816-2|MAP4_HUMAN	0.7040596	1.6960104
sp|P55884-2|EIF3B_HUMAN	0.70425797	2.6576471
sp|P07384|CAN1_HUMAN	0.7042656	9.762187
sp|P30520|PURA2_HUMAN	0.704895	1.9647322
sp|Q9UNH7|SNX6_HUMAN	0.704916	2.6576471
sp|P61163|ACTZ_HUMAN	0.70677185	2.6576471
sp|P48643|TCPE_HUMAN	0.70840454	6.918803
sp|Q6UVK1|CSPG4_HUMAN	0.70853615	3.1266599
sp|Q9BSJ8-2|ESYT1_HUMAN	0.7090244	6.3204336
sp|Q6XQN6|PNCB_HUMAN	0.70934296	5.869831
sp|Q15631|TSN_HUMAN	0.7094612	2.1818786
sp|Q16891-2|MIC60_HUMAN	0.7096386	4.051346
sp|O94919|ENDD1_HUMAN	0.71105194	2.1818786
sp|Q99798|ACON_HUMAN	0.7111454	8.558914
sp|P10155-3|RO60_HUMAN	0.71154594	3.5908275
sp|Q9Y3Z3|SAMH1_HUMAN	0.71287155	8.107274
sp|O75643|U520_HUMAN	0.7137108	1.6960104
sp|P53041|PPP5_HUMAN	0.71411324	4.051346
sp|Q9UBV8|PEF1_HUMAN	0.7144222	1.6960104
sp|O60701|UGDH_HUMAN	0.7144871	3.1266599
sp|P16219|ACADS_HUMAN	0.714592	2.1818786
sp|P68104|EF1A1_HUMAN	0.71468353	6.9708595
sp|P08754|GNAI3_HUMAN	0.7151737	1.6960104

sp|P14174|MIF_HUMAN	0.71533394	1.6960104
sp|P17987|TCPA_HUMAN	0.7159786	6.3204336
sp|P60842|IF4A1_HUMAN	0.7165718	5.869831
sp|O00170|AIP_HUMAN	0.71679497	1.6960104
sp|P09972|ALDOC_HUMAN	0.717453	3.5908275
sp|Q9NYU2-2|UGGG1_HUM	0.7176113	8.112776
sp|P50991|TCPD_HUMAN	0.71837616	4.964395
sp|O75131|CPNE3_HUMAN	0.71907425	4.876761
sp|Q6WCQ1-2|MPRIP_HUMA	0.71938324	5.869831
sp|Q9UH99-2|SUN2_HUMAN	0.7200527	2.6576471
sp|P53004|BIEA_HUMAN	0.7202015	3.5908275
sp|P18669|PGAM1_HUMAN	0.72058296	5.417897
sp|P01859|IGHG2_HUMAN	0.7207451	4.051346
sp|P47755|CAZA2_HUMAN	0.721035	3.760603
sp|P49368|TCPG_HUMAN	0.72141266	9.449409
sp|Q96IJ6-2|GMPPA_HUMAN	0.72143936	2.6576471
sp|Q9NTJ5|SAC1_HUMAN	0.7217026	2.1818786
sp|Q7Z4W1|DCXR_HUMAN	0.72174454	3.5908275
sp|P05090|APOD_HUMAN	0.7219238	1.6960104
sp|Q96I99|SUCB2_HUMAN	0.7223606	4.051346
sp|Q12905|ILF2_HUMAN	0.72265244	5.417897
sp|O95479|G6PE_HUMAN	0.7226734	3.417183
sp|O60749-2|SNX2_HUMAN	0.7226944	4.509013
sp|P11142|HSP7C_HUMAN	0.722908	8.112776
sp|Q13561-2|DCTN2_HUMAI	0.72306824	5.869831
sp|Q14980-2|NUMA1_HUMA	0.72333336	9.449409
sp|P30101|PDIA3_HUMAN	0.72340393	12.553017
sp|P10515|ODP2_HUMAN	0.7247162	3.5908275
sp|Q9BT78|CSN4_HUMAN	0.7249489	3.1266599
sp|P62829|RL23_HUMAN	0.72520065	1.6960104
sp|O75746-2|CMC1_HUMAN	0.72545624	2.1818786
sp|P62333|PRS10_HUMAN	0.7256775	3.5908275
sp|P35606-2|COPB2_HUMAN	0.72581863	6.3204336
sp|Q13263|TIF1B_HUMAN	0.7262726	4.964395
sp|P17980|PRS6A_HUMAN	0.726326	4.509013
sp|P55209-2|NP1L1_HUMAN	0.7263298	1.6960104
sp|P49720|PSB3_HUMAN	0.72660446	2.6576471
sp|P60174|TPIS_HUMAN	0.7270012	8.558914
sp|Q15046-2|SYK_HUMAN	0.7273369	3.5908275
sp|P04632|CPNS1_HUMAN	0.72743416	3.8337784
sp|Q92900-2|RENT1_HUMAI	0.7297077	4.509013
sp|O00534|VMA5A_HUMAN	0.7299557	4.509013
sp|Q15717|ELAV1_HUMAN	0.72997856	1.6960104
sp|Q08257|QOR_HUMAN	0.7301407	4.509013
sp|Q96MM6|HS12B_HUMAN	0.73020554	4.509013

sp|P63167|DYL1_HUMAN	0.73023605	2.1818786
sp|O95336|6PGL_HUMAN	0.7311592	2.2385595
sp|P31943|HNRH1_HUMAN	0.7315922	3.1266599
sp|Q9H0W9-2|CK054_HUMA	0.7316017	2.36833
sp|Q99497|PARK7_HUMAN	0.7317791	4.051346
sp|Q07075|AMPE_HUMAN	0.7325916	1.6960104
sp|P05155-2|IC1_HUMAN	0.7326603	2.6576471
sp|Q03154-4|ACY1_HUMAN	0.7333832	2.6576471
sp|P11177-3|ODPB_HUMAN	0.733387	2.6576471
sp|P06737-2|PYGL_HUMAN	0.7334366	6.253077
sp|P27361|MK03_HUMAN	0.7342186	2.6576471
sp|Q9NSE4|SYIM_HUMAN	0.73423195	5.417897
sp|O14964-2|HGS_HUMAN	0.7347069	2.1818786
sp|P08236-2|BGLR_HUMAN	0.73524094	2.1818786
sp|P12268|IMDH2_HUMAN	0.73526	2.6576471
sp|Q9UNM6-2|PSD13_HUM/	0.73584366	2.6576471
sp|Q15366-2|PCBP2_HUMAN	0.7359352	1.4104178
sp|P35221|CTNA1_HUMAN	0.7362976	10.776877
sp|Q9Y4L1|HYOU1_HUMAN	0.7366257	5.869831
sp|P02760|AMBP_HUMAN	0.7371826	3.1266599
sp|P12830|CADH1_HUMAN	0.73742676	1.6960104
sp|O75923-15|DYSF_HUMAN	0.7376766	1.6960104
sp|P55795|HNRH2_HUMAN	0.73773575	1.6960104
sp|Q9BZZ5-3|API5_HUMAN	0.73794365	2.6576471
sp|Q9NQG5|RPR1B_HUMAN	0.7380276	1.6960104
sp|O43242|PSMD3_HUMAN	0.7380905	4.509013
sp|Q71U36-2|TBA1A_HUMA	0.7382088	3.4656596
sp|P05156|CFAI_HUMAN	0.7387676	3.5908275
sp|P30566|PUR8_HUMAN	0.7389946	1.6960104
sp|Q99829|CPNE1_HUMAN	0.73908997	2.1818786
sp|P49411|EFTU_HUMAN	0.73950577	6.3204336
sp|P05546|HEP2_HUMAN	0.73962593	3.1266599
sp|Q16630-3|CPSF6_HUMAN	0.73968315	1.6960104
sp|P25325-2|THTM_HUMAN	0.7402992	2.1818786
sp|P46108|CRK_HUMAN	0.7403774	1.6960104
sp|O43795-2|MYO1B_HUMA	0.7406082	2.6576471
sp|Q9Y6W5|WASF2_HUMAN	0.7406235	2.6576471
sp|P06727|APOA4_HUMAN	0.740675	7.2183566
sp|Q9UI12-2|VATH_HUMAN	0.7408085	2.6576471
sp|P29622|KAIN_HUMAN	0.7417011	2.1818786
sp|P05556|ITB1_HUMAN	0.7436981	12.077333
sp|P31948|STIP1_HUMAN	0.7442169	6.7698927
sp|P22059|OSBP1_HUMAN	0.74445915	2.1818786
sp|Q9NY33|DPP3_HUMAN	0.7446499	5.417897
sp|Q16543|CDC37_HUMAN	0.7447281	4.4116287

sp|Q9H4A4|AMPB_HUMAN	0.74487686	6.2179356
sp|Q9Y376|CAB39_HUMAN	0.74523926	1.6960104
sp|Q9UNF0-2|PACN2_HUMA	0.74563026	2.6576471
sp|P10301|RRAS_HUMAN	0.74669266	1.6960104
sp|P04275|VWF_HUMAN	0.7481842	9.615948
sp|Q96JB5-4|CK5P3_HUMAN	0.7484112	2.1818786
sp|P53992|SC24C_HUMAN	0.7492008	2.6576471
sp|Q93009-3|UBP7_HUMAN	0.74973106	2.6576471
sp|Q8N335|GPD1L_HUMAN	0.75001526	2.1818786
sp|O15145|ARPC3_HUMAN	0.7501774	1.6960104
sp|P29401-2|TKT_HUMAN	0.7510681	14.311137
sp|Q9UBW8|CSN7A_HUMAN	0.7517395	1.6960104
sp|Q96C23|GALM_HUMAN	0.75212	1.6960104
sp|Q92841-1|DDX17_HUMAN	0.7521534	7.2183566
sp|O60506-3|HNRPQ_HUMA	0.75229454	3.5908275
sp|Q96CW1-2|AP2M1_HUMA	0.7524624	3.1266599
sp|P41250|GARS_HUMAN	0.7527113	4.509013
sp|P23456|Trypsin	0.7534466	3.5908275
sp|P55072|TERA_HUMAN	0.7546463	14.423111
sp|P22061-2|PIMT_HUMAN	0.75530624	3.1266599
sp|O94973-2|AP2A2_HUMAN	0.7558899	4.509013
sp|P50453|SPB9_HUMAN	0.7560959	5.417897
sp|Q16822|PCKGM_HUMAN	0.7565346	2.6576471
sp|Q9Y5P6-2|GMPPB_HUMA	0.7567005	3.5908275
sp|P08559-2|ODPA_HUMAN	0.75798225	4.509013
sp|Q9UBF2|COPG2_HUMAN	0.75868416	1.6960104
sp|P07099|HYEP_HUMAN	0.759037	9.449409
sp|P12955|PEPD_HUMAN	0.7593498	3.5908275
sp|P63000-2|RAC1_HUMAN	0.7596111	1.6960104
sp|P43034|LIS1_HUMAN	0.75961876	4.509013
sp|P36776-2|LONM_HUMAN	0.75984	4.051346
sp|Q5EBM0-3|CMPK2_HUMA	0.7599449	1.6960104
sp|Q04446|GLGB_HUMAN	0.7599716	3.5908275
sp|P35998|PRS7_HUMAN	0.76024437	4.964395
sp|Q14914-2|PTGR1_HUMAI	0.760458	2.1818786
sp|P78527|PRKDC_HUMAN	0.7612152	2.1818786
sp|P12270|TPR_HUMAN	0.76135635	3.4979637
sp|Q9NUQ9|FA49B_HUMAN	0.76410675	2.1818786
sp|O60884|DNJA2_HUMAN	0.76584435	2.6576471
sp|P19367-3|HXK1_HUMAN	0.76649857	9.449409
sp|P09619|PGFRB_HUMAN	0.7690582	1.3815327
sp|P31939|PUR9_HUMAN	0.7692909	6.3204336
sp|P13639|EF2_HUMAN	0.770422	15.4774685
sp|Q9NQR4|NIT2_HUMAN	0.7709961	2.1818786
sp|P00734|THRB_HUMAN	0.7710228	5.417897

sp|P27348|1433T_HUMAN	0.77150726	3.5908275
sp|P00390-2|GSHR_HUMAN	0.7721863	2.6576471
sp|O95372|LYPA2_HUMAN	0.7733402	1.6960104
sp|Q3LXA3|TKFC_HUMAN	0.7747383	3.8058946
sp|P54136|SYRC_HUMAN	0.7751808	3.5908275
sp|Q9Y3F4-2|STRAP_HUMAN	0.7756939	1.6960104
sp|Q9NRN5|OLFL3_HUMAN	0.7759361	3.5908275
sp|Q9BY32|ITPA_HUMAN	0.77740765	2.1818786
sp|P52209-2|6PGD_HUMAN	0.77832794	8.112776
sp|Q99598|TSNAX_HUMAN	0.7785835	1.6960104
sp|O43865|SAHH2_HUMAN	0.77861404	2.6576471
sp|Q96KP4|CNDP2_HUMAN	0.77967834	9.893875
sp|Q86X76-2|NIT1_HUMAN	0.7798004	1.6960104
sp|Q5K4L6-2|S27A3_HUMAN	0.7804241	1.6960104
sp|O95861-4|BPNT1_HUMAN	0.78053284	3.1266599
sp|Q6NVY1|HIBCH_HUMAN	0.7807083	2.1818786
sp|Q15942|ZYX_HUMAN	0.78110886	4.509013
sp|P19474|RO52_HUMAN	0.781332	1.6960104
sp|Q9P2R7-2|SUCB1_HUMAN	0.7813492	3.1266599
sp|P53602|MVD1_HUMAN	0.78147316	1.6960104
sp|P43686|PRS6B_HUMAN	0.78235626	4.051346
sp|O15031|PLXB2_HUMAN	0.7829895	4.051346
sp|P62140|PP1B_HUMAN	0.7833328	1.6960104
sp|Q15257-2|PTPA_HUMAN	0.78339386	3.1266599
sp|P54577|SYYC_HUMAN	0.783823	1.6960104
sp|P27695|APEX1_HUMAN	0.7840309	2.1818786
sp|Q99733-2|NP1L4_HUMAN	0.78410625	1.6960104
sp|O94776|MTA2_HUMAN	0.78453636	1.6960104
sp|P68366-2|TBA4A_HUMAN	0.7847309	2.1818786
sp|O95340-2|PAPS2_HUMAN	0.78551865	3.5908275
sp|P63010-2|AP2B1_HUMAN	0.7859726	3.5908275
sp|P15121|ALDR_HUMAN	0.7862816	4.964395
sp|P05165-2|PCCA_HUMAN	0.78678894	5.869831
sp|P00450|CERU_HUMAN	0.7869339	11.224656
sp|O94760|DDAH1_HUMAN	0.7871895	2.6576471
sp|Q13425|SNTB2_HUMAN	0.7879486	2.6576471
sp|P35222|CTNB1_HUMAN	0.7888546	4.509013
sp|Q12797-10|ASPH_HUMAN	0.7891159	4.051346
sp|Q13423|NNTM_HUMAN	0.78920364	4.509013
sp|Q7Z4I7-3|LIMS2_HUMAN	0.7894325	1.6960104
sp|Q96G03|PGM2_HUMAN	0.7896557	3.1266599
sp|P07900-2|HS90A_HUMAN	0.78990173	9.004438
sp|P46976|GLYG_HUMAN	0.78992844	2.1818786
sp|P78371|TCPB_HUMAN	0.7899952	6.7073655
sp|P48637|GSHB_HUMAN	0.79016876	4.964395

sp|P11686-2|PSPC_HUMAN	0.79063797	1.6960104
sp|P00352|AL1A1_HUMAN	0.79070663	10.781506
sp|P46063|RECQ1_HUMAN	0.79083633	3.5908275
sp|Q96QK1|VPS35_HUMAN	0.79099274	4.4116287
sp|O43776|SYNC_HUMAN	0.79105186	3.5908275
sp|P00441|SODC_HUMAN	0.79286194	2.6576471
sp|P62753|RS6_HUMAN	0.7930565	2.1818786
sp|Q15833-2|STXB2_HUMAN	0.7933254	1.6960104
sp|P42224-2|STAT1_HUMAN	0.7933464	4.051346
sp|Q7KZF4|SND1_HUMAN	0.7935982	4.051346
sp|O00429-3|DNM1L_HUMA	0.7957363	4.051346
sp|Q8NHV1|GIMA7_HUMAN	0.79647446	1.6960104
sp|P05166-2|PCCB_HUMAN	0.7966919	5.417897
sp|Q08211|DHX9_HUMAN	0.79685783	10.179513
sp|P26640|SYVC_HUMAN	0.7970104	4.051346
sp|Q16775-2|GLO2_HUMAN	0.7982054	1.6960104
sp|Q13098-5|CSN1_HUMAN	0.799757	2.6576471
sp|P48668|K2C6C_HUMAN	0.7997818	1.9951487
sp|Q9Y678|COPG1_HUMAN	0.79990387	8.112776
sp|O14950|ML12B_HUMAN	0.7999172	2.1818786
sp|Q9HCB6|SPON1_HUMAN	0.8001785	4.509013
sp|P12956|XRCC6_HUMAN	0.8007355	8.112776
sp|Q15029-2|U5S1_HUMAN	0.8010845	6.3204336
sp|P23946|CMA1_HUMAN	0.80146027	2.1818786
sp|O15355|PPM1G_HUMAN	0.80153084	1.6960104
sp|P62937|PPIA_HUMAN	0.8019924	1.6960104
sp|Q8N163-2|CCAR2_HUMA	0.80282974	2.1818786
sp|P08571|CD14_HUMAN	0.803215	2.6576471
sp|Q9BR76|COR1B_HUMAN	0.80358124	4.051346
sp|P07814|SYEP_HUMAN	0.8038063	5.869831
sp|O43143|DHX15_HUMAN	0.80399513	3.5908275
sp|Q99832|TCPH_HUMAN	0.80519676	3.5908275
sp|P16152|CBR1_HUMAN	0.8055248	5.869831
sp|P02765|FETUA_HUMAN	0.80742836	1.8444856
sp|Q9Y6C2|EMIL1_HUMAN	0.808733	7.665951
sp|O75368|SH3L1_HUMAN	0.8093376	1.6960104
sp|O60784|TOM1_HUMAN	0.80973434	2.1818786
sp|Q6P2Q9|PRP8_HUMAN	0.81106186	3.1266599
sp|Q9HBL0|TENS1_HUMAN	0.8111973	6.7698927
sp|Q9UKG1|DP13A_HUMAN	0.8112335	1.6960104
sp|Q16853|AOC3_HUMAN	0.8119106	7.665951
sp|Q15075|EEA1_HUMAN	0.8125019	6.7698927
sp|O00571-2|DDX3X_HUMAN	0.8130474	1.6960104
sp|P08238|HS90B_HUMAN	0.81427765	7.2183566
sp|Q9Y3A5|SBDS_HUMAN	0.81427956	2.1818786

sp|P13010|XRCC5_HUMAN	0.8148327	8.558914
sp|P62195|PRS8_HUMAN	0.8149414	2.1818786
sp|P53618|COPB_HUMAN	0.81515884	5.869831
sp|P13798|ACPH_HUMAN	0.81518173	5.417897
sp|Q16851-2|UGPA_HUMAN	0.81529045	5.869831
sp|Q13488|VPP3_HUMAN	0.816061	1.6960104
sp|Q53GG5-2|PDLI3_HUMAN	0.81663513	1.6960104
sp|Q7Z4H8|PLGT3_HUMAN	0.8166981	2.1818786
sp|P48147|PPCE_HUMAN	0.8168316	3.5908275
sp|P00488|F13A_HUMAN	0.81767654	7.2183566
sp|Q16531|DDB1_HUMAN	0.8178196	8.112776
sp|P30084|ECHM_HUMAN	0.8183193	2.6576471
sp|Q5T013-4|HYI_HUMAN	0.81840897	1.6960104
sp|Q00610-2|CLH1_HUMAN	0.81847763	12.110734
sp|P28838-2|AMPL_HUMAN	0.8191891	10.781506
sp|O60610-2|DIAP1_HUMAN	0.8195095	3.1266599
sp|P30041|PRDX6_HUMAN	0.8195133	5.442546
sp|Q13045-3|FLII_HUMAN	0.8200226	3.1266599
sp|Q02790|FKBP4_HUMAN	0.8202934	2.6576471
sp|Q92888-2|ARHG1_HUMA	0.8212185	1.6960104
sp|Q86VS8|HOOK3_HUMAN	0.8223076	1.6960104
sp|P43121|MUC18_HUMAN	0.82268524	1.6960104
sp|Q01469|FABP5_HUMAN	0.8251457	4.964395
sp|Q9UBE0|SAE1_HUMAN	0.82611847	2.1818786
sp|Q04760-2|LGUL_HUMAN	0.82748604	2.1818786
sp|Q13363|CTBP1_HUMAN	0.8275795	2.1818786
sp|O95782-2|AP2A1_HUMAN	0.8281002	4.509013
sp|Q92556|ELMO1_HUMAN	0.8285084	1.6960104
sp|P24752|THIL_HUMAN	0.82870865	7.2183566
sp|P0DMV9|HS71B_HUMAN	0.82935905	10.4031105
sp|Q13200|PSMD2_HUMAN	0.83013535	1.6960104
sp|Q9BXN1|ASPN_HUMAN	0.8307228	2.1818786
sp|Q562R1|ACTBL_HUMAN	0.83073235	1.6960104
sp|Q9UPN3|MACF1_HUMAN	0.83294106	1.6960104
sp|Q13618-2|CUL3_HUMAN	0.8335724	3.1266599
sp|O95865|DDAH2_HUMAN	0.8337841	4.509013
sp|P29144|TPP2_HUMAN	0.83657265	3.1266599
sp|Q9Y3I0|RTCB_HUMAN	0.8373146	4.051346
sp|P53621-2|COPA_HUMAN	0.8374653	8.112776
sp|P00558|PGK1_HUMAN	0.83821297	10.337908
sp|Q8WVV9-5|HNRLL_HUMA	0.8398247	1.6960104
sp|Q7L1Q6-2|BZW1_HUMAN	0.839859	2.1818786
sp|Q9P2B2|FPRP_HUMAN	0.8405876	2.1818786
sp|Q99873-2|ANM1_HUMAN	0.84198	2.1818786
sp|Q06210-2|GFPT1_HUMAN	0.842823	4.509013

sp|P51178-2|PLCD1_HUMAN	0.843338	1.6960104
sp|Q99460-2|PSMD1_HUMA	0.84338	3.5908275
sp|P35611-2|ADDA_HUMAN	0.84462357	3.5908275
sp|P00387-3|NB5R3_HUMAI	0.8450947	4.509013
sp|P08294|SODE_HUMAN	0.84540176	2.6576471
sp|P31153|METK2_HUMAN	0.8463974	1.6960104
sp|Q7Z5L7-2|PODN_HUMAN	0.84640884	1.6960104
sp|Q14204|DYHC1_HUMAN	0.8465729	10.337908
sp|P46109|CRKL_HUMAN	0.846756	1.6960104
sp|O94826|TOM70_HUMAN	0.8473091	2.6576471
sp|P47985|UCRI_HUMAN	0.84845257	1.6960104
sp|P09104-2|ENOG_HUMAN	0.8485527	2.6576471
sp|Q96HC4|PDLI5_HUMAN	0.8529968	3.5908275
sp|P23141-3|EST1_HUMAN	0.8530445	11.224656
sp|O00151|PDLI1_HUMAN	0.8532047	4.4116287
sp|Q01813|PFKAP_HUMAN	0.8536396	3.1266599
sp|P01031|CO5_HUMAN	0.8540821	5.869831
sp|Q14240-2|IF4A2_HUMAN	0.85463333	2.1818786
sp|Q8IWL2-2|SFTA1_HUMAI	0.85541344	4.509013
sp|P55735|SEC13_HUMAN	0.8557339	1.6960104
sp|P28482|MK01_HUMAN	0.85770035	2.6576471
sp|P09417|DHPR_HUMAN	0.8587036	1.6960104
sp|P52943|CRIP2_HUMAN	0.8591194	2.1818786
sp|P23634-2|AT2B4_HUMAN	0.8596153	3.1266599
sp|P33176|KINH_HUMAN	0.8598404	5.869831
sp|P46926|GNPI1_HUMAN	0.86058044	2.1818786
sp|P00966|ASSY_HUMAN	0.86125374	3.1266599
sp|Q15404|RSU1_HUMAN	0.86125755	4.964395
sp|P55786|PSA_HUMAN	0.86164093	9.004438
sp|P27694|RFA1_HUMAN	0.8622761	2.6576471
sp|P51884|LUM_HUMAN	0.862833	8.112776
sp|P63096|GNAI1_HUMAN	0.8630676	1.6960104
sp|Q15019-2|SEPT2_HUMAN	0.86340714	4.964395
sp|Q07954|LRP1_HUMAN	0.86481476	9.449409
sp|P50395|GDIB_HUMAN	0.86533546	8.558914
sp|P46459|NSF_HUMAN	0.8659115	4.051346
sp|P13861|KAP2_HUMAN	0.86603546	6.3204336
sp|P40939|ECHA_HUMAN	0.86701584	13.151816
sp|P32455|GBP1_HUMAN	0.8677521	4.051346
sp|Q96CN7|ISOC1_HUMAN	0.86816597	3.1266599
sp|P67936-2|TPM4_HUMAN	0.8700237	2.6576471
sp|Q969G5|CAVN3_HUMAN	0.87070656	2.1818786
sp|P00338|LDHA_HUMAN	0.8709297	8.112776
sp|Q13630|FCL_HUMAN	0.8709755	1.6960104
sp|Q9UGI8-2|TES_HUMAN	0.8712158	3.267311

sp|Q9UHB9-4|SRP68_HUMA	0.8715515	1.6960104
sp|Q14203-4|DCTN1_HUMAI	0.87194824	3.760603
sp|O75534-2|CSDE1_HUMAN	0.8721962	1.6960104
sp|P11586|C1TC_HUMAN	0.87239075	3.5908275
sp|P09960|LKHA4_HUMAN	0.8735981	9.893875
sp|Q9BRA2|TXD17_HUMAN	0.8739815	1.6960104
sp|Q16401-2|PSMD5_HUMA	0.874279	5.417897
sp|P18428|LBP_HUMAN	0.8767128	2.1818786
sp|Q9NRV9|HEBP1_HUMAN	0.87690544	2.1818786
sp|P40925-2|MDHC_HUMAN	0.8779259	5.869831
sp|Q9UEY8|ADDG_HUMAN	0.8824539	3.5908275
sp|O00232|PSD12_HUMAN	0.88269234	3.1266599
sp|P52306|GDS1_HUMAN	0.8828869	1.6960104
sp|P13716-2|HEM2_HUMAN	0.8836136	3.4656596
sp|P02787|TRFE_HUMAN	0.88481903	15.35253
sp|P35270|SPRE_HUMAN	0.884922	2.1818786
sp|O60879-2|DIAP2_HUMAN	0.8860855	1.6960104
sp|P34932|HSP74_HUMAN	0.886158	7.2183566
sp|O14974-3|MYPT1_HUMAI	0.88624954	3.1266599
sp|Q13126-2|MTAP_HUMAN	0.88694954	2.6576471
sp|Q9NVD7|PARVA_HUMAN	0.88752747	3.1266599
sp|Q8IZ83-3|A16A1_HUMAN	0.8888893	4.509013
sp|P50570-5|DYN2_HUMAN	0.88993454	3.5908275
sp|P11413-3|G6PD_HUMAN	0.8911648	5.417897
sp|O14558|HSPB6_HUMAN	0.89258575	2.1818786
sp|O14980|XPO1_HUMAN	0.89279366	2.1818786
sp|Q9BUF5|TBB6_HUMAN	0.89439774	1.6960104
sp|P26639-2|SYTC_HUMAN	0.8963852	3.5908275
sp|P00568|KAD1_HUMAN	0.89810944	4.964395
sp|Q13409-2|DC1I2_HUMAN	0.8986492	3.1266599
sp|P22105-1|TENX_HUMAN	0.89974594	11.667864
sp|Q92696|PGTA_HUMAN	0.8998928	2.1818786
sp|Q14847|LASP1_HUMAN	0.900301	3.1266599
sp|P54802|ANAG_HUMAN	0.90137386	1.6960104
sp|P0DOX5|IGG1_HUMAN	0.9021969	2.7685447
sp|P34896-2|GLYC_HUMAN	0.90231514	2.1818786
sp|P30153|2AAA_HUMAN	0.90418625	7.665951
sp|Q96C86|DCPS_HUMAN	0.904253	3.1266599
sp|A0AVT1|UBA6_HUMAN	0.90460396	2.1818786
sp|Q14764|MVP_HUMAN	0.90491486	13.420564
sp|P24821-4|TENA_HUMAN	0.9065857	7.2183566
sp|P23526|SAHH_HUMAN	0.907444	6.7698927
sp|Q93052|LPP_HUMAN	0.90802765	4.964395
sp|Q92499|DDX1_HUMAN	0.90812683	5.417897
sp|P17858-2|PFKAL_HUMAN	0.91038895	4.964395

sp|Q07866-10|KLC1_HUMAN	0.9107094	1.6960104
sp|Q6P179|ERAP2_HUMAN	0.911726	2.1818786
sp|P07357|CO8A_HUMAN	0.9117737	3.5908275
sp|Q8N2S1-3|LTBP4_HUMAN	0.91186523	4.051346
sp|P53396-2|ACLY_HUMAN	0.9121494	7.665951
sp|Q6DD88|ATLA3_HUMAN	0.9121895	3.1266599
sp|Q9NRX4|PHP14_HUMAN	0.91241074	1.6960104
sp|P17655|CAN2_HUMAN	0.9135647	10.337908
sp|O60763-2|USO1_HUMAN	0.91495895	4.509013
sp|P98095-2|FBLN2_HUMAN	0.91610336	4.285869
sp|P08572|CO4A2_HUMAN	0.9161911	4.051346
sp|P20591|MX1_HUMAN	0.9162941	1.6960104
sp|Q9NVA2|SEP11_HUMAN	0.9164009	4.509013
sp|O43813|LANC1_HUMAN	0.9198704	2.6576471
sp|Q9HC38-2|GLOD4_HUMA	0.9199772	3.1266599
sp|O15061-2|SYNEM_HUMA	0.92007256	4.051346
sp|Q12805-2|FBLN3_HUMAN	0.9225292	6.3204336
sp|P08133|ANXA6_HUMAN	0.92287064	15.95459
sp|P01019|ANGT_HUMAN	0.92414093	2.6576471
sp|O00291|HIP1_HUMAN	0.9248009	1.9647322
sp|P42330|AK1C3_HUMAN	0.9255905	2.1818786
sp|P12277|KCRB_HUMAN	0.92560005	4.509013
sp|P30086|PEBP1_HUMAN	0.92731285	4.051346
sp|Q8WUM4|PDC6I_HUMAN	0.92856216	11.667864
sp|P36955|PEDF_HUMAN	0.9296112	3.5908275
sp|Q99758|ABCA3_HUMAN	0.9299469	3.1266599
sp|Q15436|SC23A_HUMAN	0.93017197	2.6576471
sp|Q709C8-2|VP13C_HUMAN	0.9304056	1.6960104
sp|O00410|IPO5_HUMAN	0.93053055	3.1266599
sp|Q9UBT2|SAE2_HUMAN	0.93066406	3.1266599
sp|Q06124-2|PTN11_HUMAN	0.9316063	2.1818786
sp|Q9NSD9|SYFB_HUMAN	0.93164825	1.6960104
sp|P02743|SAMP_HUMAN	0.93309593	4.051346
sp|Q8TBC4|UBA3_HUMAN	0.9333992	2.6576471
sp|P13489|RINI_HUMAN	0.93445396	9.893875
sp|P22314-2|UBA1_HUMAN	0.93634796	12.996504
sp|P49327|FAS_HUMAN	0.9367542	12.996504
sp|P48163|MAOX_HUMAN	0.9406948	3.1266599
sp|Q16555|DPYL2_HUMAN	0.94083023	8.112776
sp|P06756-3|ITAV_HUMAN	0.9409752	3.5908275
sp|Q15582|BGH3_HUMAN	0.9419174	8.558914
sp|Q27J81-2|INF2_HUMAN	0.94209385	1.6960104
sp|O75083|WDR1_HUMAN	0.9438553	10.337908
sp|Q15121|PEA15_HUMAN	0.9510803	1.6960104
sp|Q15274|NADC_HUMAN	0.9520302	2.1818786

sp|Q9ULV4-3|COR1C_HUMA	0.95223045	6.3204336
sp|P49915-2|GUAA_HUMAN	0.95495605	1.6960104
sp|P21980|TGM2_HUMAN	0.9551754	8.558914
sp|Q8NBF2-2|NHLC2_HUMA	0.95519257	1.6960104
sp|P07437|TBB5_HUMAN	0.95975685	2.1818786
sp|P08603|CFAH_HUMAN	0.9604988	15.176438
sp|Q16181-2|SEPT7_HUMAN	0.9605503	7.2183566
sp|Q14974|IMB1_HUMAN	0.96253204	6.7698927
sp|Q9UBG0|MRC2_HUMAN	0.9682503	3.5908275
sp|P55060-3|XPO2_HUMAN	0.97229576	4.051346
sp|Q7Z7G0|TARSH_HUMAN	0.97302246	1.6960104
sp|P27338|AOFB_HUMAN	0.9742298	5.869831
sp|P49591|SYSC_HUMAN	0.97502136	4.509013
sp|P29466-2|CASP1_HUMAN	0.97525215	1.6960104
sp|Q86VP6|CAND1_HUMAN	0.97608566	1.6960104
sp|P00918|CAH2_HUMAN	0.97735214	4.964395
sp|Q13451|FKBP5_HUMAN	0.97817993	2.6576471
sp|Q8IUX7|AEBP1_HUMAN	0.97891235	1.6960104
sp|P56199|ITA1_HUMAN	0.9810219	6.3204336
sp|P14550|AK1A1_HUMAN	0.9825096	6.7698927
sp|P46939-2|UTRO_HUMAN	0.9836674	10.337908
sp|P51888|PRELP_HUMAN	0.9849796	6.3204336
sp|P22897|MRC1_HUMAN	0.9853077	6.7698927
sp|Q6UWY5|OLFL1_HUMAN	0.9906597	4.509013
sp|P35555|FBN1_HUMAN	0.9908447	14.398287
sp|P49189|AL9A1_HUMAN	0.9921799	7.665951
sp|Q9P0V9-2|SEP10_HUMAN	0.9966984	3.1266599
sp|Q9H008|LHPP_HUMAN	0.9979553	2.6576471
sp|P39059|COFA1_HUMAN	0.9992256	2.1818786
sp|O60831|PRAF2_HUMAN	1.001955	1.6960104
sp|Q9BZQ8|NIBAN_HUMAN	1.0023556	2.1818786
sp|P20774|MIME_HUMAN	1.0024014	4.051346
sp|Q9Y696|CLIC4_HUMAN	1.0026894	3.1266599
sp|P60660-2|MYL6_HUMAN	1.0029526	4.051346
sp|P31150|GDIA_HUMAN	1.0040798	4.964395
sp|Q9NZ08-2|ERAP1_HUMA	1.0042019	3.5908275
sp|P40763-2|STAT3_HUMAN	1.0056744	4.964395
sp|P42704|LPPRC_HUMAN	1.007103	2.1818786
sp|Q14767|LTBP2_HUMAN	1.0074787	8.3491535
sp|Q04828|AK1C1_HUMAN	1.012495	1.6960104
sp|P36871|PGM1_HUMAN	1.0183372	9.004438
sp|P18084|ITB5_HUMAN	1.0217152	1.6960104
sp|P49588-2|SYAC_HUMAN	1.0238838	8.558914
sp|Q14195-2|DPYL3_HUMAN	1.0241661	8.558914
sp|Q13813-2|SPTN1_HUMAN	1.0275269	1.6960104

sp|Q9UBQ7|GRHPR_HUMAN	1.0287685	1.6960104
sp|P49419-2|AL7A1_HUMAN	1.0307484	9.004438
sp|Q96AC1|FERM2_HUMAN	1.030941	8.558914
sp|Q07507|DERM_HUMAN	1.0312881	3.5908275
sp|P13797|PLST_HUMAN	1.0313969	8.112776
sp|P07195|LDHB_HUMAN	1.0316677	6.7698927
sp|O43488|ARK72_HUMAN	1.0330563	2.1818786
sp|P45974-2|UBP5_HUMAN	1.036581	6.7698927
sp|Q63ZY3-3|KANK2_HUMAI	1.0410957	3.5908275
sp|P06681|CO2_HUMAN	1.0414696	3.5908275
sp|P07358|CO8B_HUMAN	1.0441036	2.1818786
sp|Q9BUT1|BDH2_HUMAN	1.0467987	2.6576471
sp|Q13418|ILK_HUMAN	1.0470257	4.509013
sp|P02748|CO9_HUMAN	1.0475979	5.869831
sp|P10643|CO7_HUMAN	1.0540695	4.964395
sp|P13671|CO6_HUMAN	1.0557022	3.5908275
sp|P10768|ESTD_HUMAN	1.0654564	2.6576471
sp|P21810|PGS1_HUMAN	1.066288	5.869831
sp|P08237-3|PFKAM_HUMAI	1.069706	2.1818786
sp|Q9Y2A7-2|NCKP1_HUMAI	1.0699844	1.6960104
sp|O43707|ACTN4_HUMAN	1.0736923	14.75047
sp|P07585|PGS2_HUMAN	1.0751305	5.869831
sp|Q8WX93-5|PALLD_HUMA	1.075592	5.417897
sp|P02452|CO1A1_HUMAN	1.078085	1.9413493
sp|Q96PD5-2|PGRP2_HUMAI	1.0850658	1.6960104
sp|P11216|PYGB_HUMAN	1.088028	9.893875
sp|P42226|STAT6_HUMAN	1.0887871	1.6960104
sp|Q13228-4|SBP1_HUMAN	1.0916424	13.94599
sp|P12109|CO6A1_HUMAN	1.0953541	9.004438
sp|Q07960|RHG01_HUMAN	1.0968971	3.5908275
sp|P12110|CO6A2_HUMAN	1.1057243	9.449409
sp|P00325|ADH1B_HUMAN	1.1126595	4.509013
sp|O94979-10|SC31A_HUMA	1.1174173	1.5646582
sp|Q92878-2|RAD50_HUMAI	1.1181984	1.6960104
sp|Q08431|MFGM_HUMAN	1.1191597	2.1818786
sp|Q16647|PTGIS_HUMAN	1.119709	2.6576471
sp|Q15063-3|POSTN_HUMAI	1.1211491	9.449409
sp|P04792|HSPB1_HUMAN	1.126339	6.7698927
sp|P60981-2|DEST_HUMAN	1.1295681	2.6576471
sp|P36269-2|GGT5_HUMAN	1.1355	2.6576471
sp|Q13557-10|KCC2D_HUMA	1.1418533	3.5908275
sp|P22033|MUTA_HUMAN	1.1427498	1.6960104
sp|P50135|HNMT_HUMAN	1.1490746	1.6960104
sp|P01860|IGHG3_HUMAN	1.158184	2.6576471
sp|P22102|PUR2_HUMAN	1.1678371	2.1818786

sp|P48059-3|LIMS1_HUMAN	1.1756592	2.1818786
sp|P15088|CBPA3_HUMAN	1.1990242	2.6576471
sp|P24844|MYL9_HUMAN	1.1996956	1.6960104
sp|Q15124|PGM5_HUMAN	1.2163506	9.004438
sp|O43175|SERA_HUMAN	1.2298946	4.051346
sp|P00740-2|FA9_HUMAN	1.2442608	2.6576471
sp|P35580|MYH10_HUMAN	1.2497044	15.35253
sp|A6NMZ7|CO6A6_HUMAN	1.2500267	9.449409
sp|P11766|ADHX_HUMAN	1.257534	6.7698927
sp|O43294|TGFI1_HUMAN	1.2634926	2.1818786
sp|P50225|ST1A1_HUMAN	1.2986374	1.6960104
sp|P15428-5|PGDH_HUMAN	1.3036098	1.6960104
sp|P08123|CO1A2_HUMAN	1.3178577	2.6576471
sp|P18206-2|VINC_HUMAN	1.321495	14.840647
sp|P01833|PIGR_HUMAN	1.3372192	5.417897
sp|Q13976|KGP1_HUMAN	1.3607788	3.1266599
sp|Q9UBX5|FBLN5_HUMAN	1.3765259	4.051346
sp|Q9NZU5|LMCD1_HUMAN	1.4248524	6.3204336
sp|Q9UMS6-2|SYNP2_HUMA	1.4356117	3.5908275
sp|P02794|FRIH_HUMAN	1.4457741	2.6576471
sp|P21291|CSRP1_HUMAN	1.4528923	4.964395
sp|Q15746-3|MYLK_HUMAN	1.4535484	10.781506
sp|Q9NR12-2|PDLI7_HUMAN	1.4588127	3.5908275
sp|P02792|FRIL_HUMAN	1.4913712	3.1266599
sp|P07951|TPM2_HUMAN	1.507824	4.509013
sp|P51911|CNN1_HUMAN	1.5106812	4.964395
sp|P09493-9|TPM1_HUMAN	1.5158215	1.6960104
sp|P07951-3|TPM2_HUMAN	1.5393085	2.1818786
sp|P62736|ACTA_HUMAN	1.5403309	6.7698927
sp|Q05682|CALD1_HUMAN	1.5722351	2.1818786
sp|P09493-8|TPM1_HUMAN	1.5833015	3.1266599
sp|P21333-2|FLNA_HUMAN	1.5921688	13.651394
sp|P30837|AL1B1_HUMAN	1.6111813	2.6576471
sp|P17661|DESM_HUMAN	1.6647968	14.778499
sp|P02511|CRYAB_HUMAN	1.6936283	1.6960104
sp|P21266|GSTM3_HUMAN	1.7187538	4.509013
sp|P15090|FABP4_HUMAN	1.7299442	3.1266599
sp|Q01995|TAGL_HUMAN	1.9732094	8.57438
sp|P07451|CAH3_HUMAN	2.0863323	3.1266599
sp|Q04826|1B40_HUMAN	-1.4876137	0.656254
sp|P13761|2B17_HUMAN	-1.051136	0.656254
sp|P30453|1A34_HUMAN	-0.8772936	0.656254
sp|Q16777|H2A2C_HUMAN	-0.8654175	0.656254
sp|P08246|ELNE_HUMAN	-0.7145748	1.1932944
sp|P49913|CAMP_HUMAN	-0.4787922	1.1932944

sp|P04908|H2A1B_HUMAN	-0.474247	0.656254
sp|P25815|S100P_HUMAN	-0.3963356	0.656254
sp|P41218|MNDA_HUMAN	-0.3615398	0.06291623
sp|O60814|H2B1K_HUMAN	-0.3188286	1.1932944
sp|P06899|H2B1J_HUMAN	-0.2472229	1.1932944
sp|Q15109-10|RAGE_HUMAI	-0.1943436	0.84879977
sp|P59666|DEF3_HUMAN	-0.1813583	0.06291623
sp|P05164-3|PERM_HUMAN	-0.1524048	1.0261611
sp|P12821-2|ACE_HUMAN	-0.136404	0.37060758
sp|P13760|2B14_HUMAN	-0.0846424	0
sp|Q8TD06|AGR3_HUMAN	-0.0843391	0
sp|P38159|RBMX_HUMAN	-0.0822277	0.19149946
sp|P67809|YBOX1_HUMAN	-0.046298	0
sp|Q92522|H1X_HUMAN	-0.044035	0.19149946
sp|P31947-2|1433S_HUMAN	-0.0437183	0
sp|P80188|NGAL_HUMAN	-0.028801	0.06986027
sp|P15559-3|NQO1_HUMAN	-0.0219193	0.35795313
sp|P07339|CATD_HUMAN	-0.0101357	0.31423286
sp|P20702|ITAX_HUMAN	-0.0097542	0
sp|P19012-2|K1C15_HUMAN	0.0097065	0
sp|P50897-2|PPT1_HUMAN	0.0137825	0.2178309
sp|P05109|S10A8_HUMAN	0.01556587	0.06842031
sp|P63218|GBG5_HUMAN	0.02409744	0.17319627
sp|P00738-2|HPT_HUMAN	0.02537537	0.22702569
sp|P23229-4|ITA6_HUMAN	0.02682686	0
sp|P20160|CAP7_HUMAN	0.04096222	0.2178309
sp|Q13938-4|CAYP1_HUMAN	0.044384	0
sp|P06703|S10A6_HUMAN	0.0603714	0
sp|P18077|RL35A_HUMAN	0.06687164	0.19149946
sp|Q9UBR2|CATZ_HUMAN	0.06947517	0
sp|P84103-2|SRSF3_HUMAN	0.07398224	0.7498006
sp|P02786|TFR1_HUMAN	0.07824516	0.08684197
sp|P24158|PRTN3_HUMAN	0.0791359	0.09894868
sp|P05534|1A24_HUMAN	0.08354759	0
sp|O95810|CAVN2_HUMAN	0.10145378	0.8059303
sp|P60903|S10AA_HUMAN	0.10464668	0
sp|P08729|K2C7_HUMAN	0.11673546	0.7021525
sp|P43353-2|AL3B1_HUMAN	0.12232971	0
sp|Q13510-2|ASAH1_HUMAI	0.12563515	0.99757636
sp|P13686|PPA5_HUMAN	0.13054085	0.2178309
sp|P31949|S10AB_HUMAN	0.13254929	0.48520416
sp|P13498|CY24A_HUMAN	0.13759995	0
sp|P25774|CATS_HUMAN	0.14257812	0
sp|P61626|LYSC_HUMAN	0.14291382	0.87291557
sp|Q14956-2|GPNMB_HUMA	0.14641953	0.45033538

sp|Q14011|CIRBP_HUMAN	0.14945316	0.45033538
sp|Q9Y5S9-2|RBM8A_HUMA	0.15189743	0.7061832
sp|Q7Z3D6-3|GLUCM_HUMA	0.15275955	0
sp|P08174-3|DAF_HUMAN	0.15427208	0.2178309
sp|P00167-2|CYB5_HUMAN	0.15481949	0.7061832
sp|P02769|ALBU_BOVIN	0.16009903	0.24990007
sp|P16671-4|CD36_HUMAN	0.1610241	0.06291623
sp|P00739-2|HPTR_HUMAN	0.16213226	0.65625405
sp|P56134-3|ATPK_HUMAN	0.1723957	0
sp|P07858|CATB_HUMAN	0.17547989	1.0951192
sp|Q6P4A8|PLBL1_HUMAN	0.17871475	0.35795313
sp|Q9NPY3|C1QR1_HUMAN	0.17897224	0.40256184
sp|Q13247-3|SRSF6_HUMAN	0.18217468	1.1932944
sp|P0C0S5|H2AZ_HUMAN	0.18300438	0.656254
sp|Q29865|1C18_HUMAN	0.18540955	0.7061832
sp|O00757|F16P2_HUMAN	0.19205475	0.45033538
sp|O75155|CAND2_HUMAN	0.19525146	0.656254
sp|P09429|HMGB1_HUMAN	0.19994736	0.19149946
sp|P28676|GRAN_HUMAN	0.20148468	1.0485198
sp|Q8WWI1-5|LMO7_HUMA	0.20188332	0.87291557
sp|Q13243-3|SRSF5_HUMAN	0.20908928	0.45033538
sp|P04229|2B11_HUMAN	0.21119308	0.7827403
sp|P50895|BCAM_HUMAN	0.21408463	0.6615227
sp|Q9UM07|PADI4_HUMAN	0.21507263	0.7061832
sp|P30838|AL3A1_HUMAN	0.2169838	0.45033538
sp|P17213|BPI_HUMAN	0.21891785	0
sp|P14780|MMP9_HUMAN	0.22148705	0.56808305
sp|O95716|RAB3D_HUMAN	0.22312737	0
sp|P27487|DPP4_HUMAN	0.22627258	0.61034113
sp|P61604|CH10_HUMAN	0.22968102	0.5111962
sp|P11678|PERE_HUMAN	0.23331451	0
sp|Q53FA7|QORX_HUMAN	0.23898697	0.7827403
sp|P20340-2|RAB6A_HUMAN	0.23914146	0.656254
sp|P04066|FUCO_HUMAN	0.24085236	0.45033538
sp|P13987-2|CD59_HUMAN	0.24890327	0.7827403
sp|P09758|TACD2_HUMAN	0.2506733	0.90036625
sp|P63220|RS21_HUMAN	0.25068283	0
sp|P62714|PP2AB_HUMAN	0.25229263	0.656254
sp|P50995-2|ANX11_HUMAN	0.25307274	0.50298375
sp|P08727|K1C19_HUMAN	0.25334358	1.176857
sp|O43615|TIM44_HUMAN	0.25879383	0.45033538
sp|P09668|CATH_HUMAN	0.26002312	0.8744098
sp|Q32MZ4-3|LRRF1_HUMA	0.26161194	0.43633315
sp|P51991|ROA3_HUMAN	0.26214027	0
sp|Q01130-2|SRSF2_HUMAN	0.26706314	1.1505735

sp|P51153|RAB13_HUMAN	0.26712036	0
sp|Q15126|PMVK_HUMAN	0.26714897	0
sp|Q99729-2|ROAA_HUMAN	0.2721691	1.1505735
sp|Q03135-2|CAV1_HUMAN	0.27230835	0
sp|Q13231-2|CHIT1_HUMAN	0.27311707	0.312067
sp|P06702|S10A9_HUMAN	0.27545166	0.2838047
sp|Q00839|HNRPU_HUMAN	0.2773819	1.1437052
sp|P49065|ALBU_RABIT	0.27796936	0.656254
sp|P19525-2|E2AK2_HUMAN	0.28163433	0
sp|P16070-16|CD44_HUMAN	0.28239822	0.28516325
sp|Q9NZA1-2|CLIC5_HUMAN	0.2824688	0.5283125
sp|Q01105-2|SET_HUMAN	0.2832489	1.1505735
sp|P07910-2|HNRPC_HUMAN	0.28440285	1.0634323
sp|P12429|ANXA3_HUMAN	0.28470993	0.1806812
sp|P09651-3|ROA1_HUMAN	0.29624176	1.0634323
sp|Q6PIU2-2|NCEH1_HUMAN	0.29768753	1.0634323
sp|P33151|CADH5_HUMAN	0.29821396	0.7061832
sp|Q8NBX0|SCPDL_HUMAN	0.30376434	1.0485198
sp|Q96HE7|ERO1A_HUMAN	0.30657768	0.5204253
sp|Q9BXM0|PRAX_HUMAN	0.31501007	0.7827403
sp|P51608|MECP2_HUMAN	0.3157196	1.1932944
sp|P78347-2|GTF2I_HUMAN	0.31734276	0.45033538
sp|Q8TDL5|BPIB1_HUMAN	0.32038498	0.91272414
sp|P15153|RAC2_HUMAN	0.3247261	0.656254
sp|Q04837|SSBP_HUMAN	0.3252411	0.91601294
sp|O95994|AGR2_HUMAN	0.32761574	0.84879977
sp|P26447|S10A4_HUMAN	0.33115196	0.4075265
sp|P04440|DPB1_HUMAN	0.3342743	0.7827403
sp|P51571|SSRD_HUMAN	0.33458328	0.7061832
sp|P61769|B2MG_HUMAN	0.3362999	1.1932944
sp|Q96AP7|ESAM_HUMAN	0.33781433	0.7827403
sp|Q14533|KRT81_HUMAN	0.33873367	0.6070219
sp|Q02318|CP27A_HUMAN	0.34090614	1.1932944
sp|Q04941|PLP2_HUMAN	0.34344864	0
sp|Q15637-3|SF01_HUMAN	0.34493065	0.91601294
sp|Q8TC12-2|RDH11_HUMAN	0.34661674	1.1932944
sp|P62750|RL23A_HUMAN	0.34663963	0.45033538
sp|O14672|ADA10_HUMAN	0.35002136	0.7827403
sp|P15311|EZRI_HUMAN	0.35158157	0.19134797
sp|Q9NZN3|EHD3_HUMAN	0.3562889	0.35795313
sp|Q10589-2|BST2_HUMAN	0.35751343	1.1932944
sp|P0DOX2|IGA2_HUMAN	0.3704605	0.45033538
sp|O75521-2|ECI2_HUMAN	0.3719349	0.312067
sp|Q9UHL4|DPP2_HUMAN	0.37213135	0.84879977
sp|P01920|DQB1_HUMAN	0.37278366	0

sp|P30491|1B53_HUMAN	0.37434387	0
sp|O60234|GMFG_HUMAN	0.37455177	0.7498006
sp|Q13404|UB2V1_HUMAN	0.37505913	0.7827403
sp|P06753|TPM3_HUMAN	0.38567162	0.656254
sp|P07355|ANXA2_HUMAN	0.38676834	0.3761346
sp|P11279|LAMP1_HUMAN	0.3891449	1.1932944
sp|P05387|RLA2_HUMAN	0.39034462	1.1932944
sp|P48960-2|CD97_HUMAN	0.39170647	1.1932944
sp|P84090|ERH_HUMAN	0.39402962	1.1505735
sp|Q9BRF8|CPPED_HUMAN	0.39995956	0
sp|P35637-2|FUS_HUMAN	0.40268135	1.1932944
sp|Q16787-3|LAMA3_HUMA	0.41191292	1.1932944
sp|Q9UBQ0-2|VPS29_HUMA	0.4141426	1.1932944
sp|P55036-2|PSMD4_HUMAI	0.4168434	0.6070219
sp|P09493-5|TPM1_HUMAN	0.4172058	1.1932944
sp|P30626-2|SORCN_HUMAI	0.41797256	0.95332193
sp|O43760-2|SNG2_HUMAN	0.41817093	0.7827403
sp|Q15599-2|NHRF2_HUMA	0.41820908	0.8983557
sp|P17931|LEG3_HUMAN	0.41942787	1.0485198
sp|P62244|RS15A_HUMAN	0.4208603	1.1932944
sp|Q96EE3-1|SEH1_HUMAN	0.42388725	1.1932944
sp|O95837|GNA14_HUMAN	0.42650795	0.656254
sp|P08842|STS_HUMAN	0.42654228	1.1932944
sp|Q8WXH0-2|SYNE2_HUMA	0.42879105	1.1932944
sp|O75348|VATG1_HUMAN	0.4313755	0.7061832
sp|P55196-5|AFAD_HUMAN	0.43245125	1.0485198
sp|Q16762|THTR_HUMAN	0.43327332	0.69308156
sp|Q9HB40|RISC_HUMAN	0.43755722	1.1932944
sp|P04083|ANXA1_HUMAN	0.43992233	0.40707567
sp|Q8TCJ2|STT3B_HUMAN	0.4431076	1.1932944
sp|Q02878|RL6_HUMAN	0.44394302	1.1932944
sp|P08397-2|HEM3_HUMAN	0.44452667	1.1505735
sp|P54819-2|KAD2_HUMAN	0.44586182	1.0485198
sp|Q9NV96-3|CC50A_HUMA	0.4459648	0.656254
sp|Q9H6R3|ACSS3_HUMAN	0.4465027	0
sp|P29692|EF1D_HUMAN	0.44779015	0.656254
sp|Q9BUQ8|DDX23_HUMAN	0.44909286	1.1932944
sp|Q99584|S10AD_HUMAN	0.4504013	0.7827403
sp|Q15836|VAMP3_HUMAN	0.45145988	0.656254
sp|Q08170|SRSF4_HUMAN	0.45340347	0.656254
sp|Q8N5K1|CISD2_HUMAN	0.45360184	1.1932944
sp|Q9P0J0-2|NDUAD_HUMA	0.45449448	0.45033538
sp|O75475|PSIP1_HUMAN	0.45614433	0.656254
sp|P14678|RSMB_HUMAN	0.4583912	1.1932944
sp|P11166|GTR1_HUMAN	0.45980072	1.1932944

sp|A0A0B4J1X8|HV343_HUM	0.46011543	0.656254
sp|P01911|2B1F_HUMAN	0.4601841	0.656254
sp|Q9Y2Q3-3|GSTK1_HUMA	0.46051788	0.6298893
sp|P01111|RASN_HUMAN	0.46559906	0.656254
sp|O15173-2|PGRC2_HUMA	0.46579742	0.97209185
sp|P62314|SMD1_HUMAN	0.46695423	1.1932944
sp|P19404|NDUV2_HUMAN	0.4676075	1.1932944
sp|P62995-3|TRA2B_HUMAN	0.46874046	1.1932944
sp|Q15286-2|RAB35_HUMA	0.46902466	0.656254
sp|Q15056-2|IF4H_HUMAN	0.46928024	1.1932944
sp|Q9P265|DIP2B_HUMAN	0.4708004	0.656254
sp|P37235|HPCL1_HUMAN	0.47247887	0.656254
sp|Q9BXP5-5|SRRT_HUMAN	0.47322273	1.1276597
sp|P55290-4|CAD13_HUMAN	0.4733162	1.1505735
sp|P00403|COX2_HUMAN	0.47634315	1.1932944
sp|P07948-2|LYN_HUMAN	0.47743034	0.656254
sp|O75608-2|LYPA1_HUMAN	0.4804325	0.45033538
sp|P62857|RS28_HUMAN	0.48050117	0.8983557
sp|O75964|ATP5L_HUMAN	0.4831581	1.1932944
sp|Q92506|DHB8_HUMAN	0.48324966	1.1932944
sp|P14317|HCLS1_HUMAN	0.48557472	0.45033538
sp|Q92734-2|TFG_HUMAN	0.48635292	1.1932944
sp|P10523|ARRS_HUMAN	0.48970795	0.656254
sp|Q53H82|LACB2_HUMAN	0.49230766	1.1932944
sp|O43684-2|BUB3_HUMAN	0.4934044	1.1932944
sp|P51636-2|CAV2_HUMAN	0.49692535	1.1932944
sp|P40121|CAPG_HUMAN	0.4970169	0.3761346
sp|Q9HD89|RETN_HUMAN	0.5024071	0.45033538
sp|Q96I15|SCLY_HUMAN	0.5027561	1.1932944
sp|Q9Y2Q5|LTOR2_HUMAN	0.50442886	1.1932944
sp|P01116-2|RASK_HUMAN	0.50701904	1.1932944
sp|P62070-4|RRAS2_HUMAN	0.5107155	0.656254
sp|Q92930|RAB8B_HUMAN	0.51340675	0.656254
sp|P61960|UFM1_HUMAN	0.51428604	1.1932944
sp|P06396-2|GELS_HUMAN	0.5160389	0.656254
sp|Q6IBS0|TWF2_HUMAN	0.51651955	0.19149946
sp|O15254-2|ACOX3_HUMA	0.51727486	0.656254
sp|Q9NVJ2|ARL8B_HUMAN	0.5178566	1.1932944
sp|Q8IY17-3|PLPL6_HUMAN	0.5185852	1.1932944
sp|P36957|ODO2_HUMAN	0.51966286	1.1932944
sp|Q9BW30|TPPP3_HUMAN	0.5212097	0.29066643
sp|P49458|SRP09_HUMAN	0.52254295	1.1932944
sp|Q6KB66-2|K2C80_HUMAN	0.52539825	0.656254
sp|P01591|IGJ_HUMAN	0.5275364	0.7827403
sp|Q7Z7H5-3|TMED4_HUMA	0.52957535	0.656254

sp|Q13232|NDK3_HUMAN	0.5342388	1.1932944
sp|Q9BVK6|TMED9_HUMAN	0.5347805	1.1932944
sp|P22234-2|PUR6_HUMAN	0.53845215	1.2095301
sp|Q96M27-3|PRRC1_HUMA	0.54123306	1.1932944
sp|P31689|DNJA1_HUMAN	0.5415535	1.1505735
sp|Q16539-2|MK14_HUMAN	0.54169464	1.0301651
sp|P62942|FKB1A_HUMAN	0.5425434	0.656254
sp|O95197-2|RTN3_HUMAN	0.54299545	1.1932944
sp|Q9P2M7|CING_HUMAN	0.5438309	1.0485198
sp|P51858|HDGF_HUMAN	0.5444145	0.656254
sp|P62277|RS13_HUMAN	0.5452194	1.1932944
sp|P55010|IF5_HUMAN	0.54559517	1.1932944
sp|Q9UJZ1-2|STML2_HUMAI	0.5472374	1.1932944
sp|Q9H845|ACAD9_HUMAN	0.5495472	1.1932944
sp|P12236|ADT3_HUMAN	0.54964066	1.1932944
sp|Q5JTV8-3|TOIP1_HUMAN	0.55038834	0.8983557
sp|P32969|RL9_HUMAN	0.5513954	0.91601294
sp|Q9NPJ3-2|ACO13_HUMAI	0.55184555	1.1932944
sp|P51553-2|IDH3G_HUMAN	0.55221176	1.1932944
sp|P30443|1A01_HUMAN	0.5534859	1.1932944
sp|Q9P2X0-2|DPM3_HUMAN	0.5579319	1.1932944
sp|P62318-2|SMD3_HUMAN	0.5580044	1.1932944
sp|Q9Y3B3|TMED7_HUMAN	0.5594883	1.1932944
sp|P09496-2|CLCA_HUMAN	0.5596638	1.0301651
sp|P62854|RS26_HUMAN	0.560318	1.1932944
sp|P20810-10|ICAL_HUMAN	0.5630512	1.1505735
sp|Q9H2U2-2|IPYR2_HUMAN	0.5653248	1.1932944
sp|P61956-2|SUMO2_HUMA	0.5662823	0.656254
sp|O14791-2|APOL1_HUMAN	0.5674267	1.1932944
sp|P36551|HEM6_HUMAN	0.5681	1.2941489
sp|Q8NFW8|NEUA_HUMAN	0.56993866	1.1932944
sp|Q9Y6E0|STK24_HUMAN	0.5727682	0.7827403
sp|P50452|SPB8_HUMAN	0.572958	0.45033538
sp|Q9BPW8|NIPS1_HUMAN	0.5781269	1.1932944
sp|P20674|COX5A_HUMAN	0.5853214	1.1932944
sp|P49721|PSB2_HUMAN	0.58596134	0.656254
sp|P23083|HV102_HUMAN	0.5865898	0.656254
sp|P18085|ARF4_HUMAN	0.5873604	1.1932944
sp|Q04695|K1C17_HUMAN	0.5876236	0.656254
sp|Q15369-2|ELOC_HUMAN	0.5876293	1.1932944
sp|P28074|PSB5_HUMAN	0.5937538	1.1932944
sp|Q9Y3C8|UFC1_HUMAN	0.5951519	1.1932944
sp|P61923-5|COPZ1_HUMAN	0.5953598	1.1932944
sp|Q96RF0-2|SNX18_HUMAI	0.59975624	1.1932944
sp|Q3KQV9|UAP1L_HUMAN	0.599802	0

sp|P60891|PRPS1_HUMAN	0.6030712	1.1932944
sp|P51570-2|GALK1_HUMAN	0.6034584	1.1932944
sp|Q6P1N9|TATD1_HUMAN	0.6061115	1.1932944
sp|A0A0C4DH25|KVD20_HUM	0.60806084	0.656254
sp|Q9NX46|ARHL2_HUMAN	0.60928726	1.1932944
sp|P21283|VATC1_HUMAN	0.6093693	1.1932944
sp|P61026|RAB10_HUMAN	0.61037064	0.656254
sp|P10316|1A69_HUMAN	0.6112137	0.656254
sp|Q00013-2|EM55_HUMAN	0.61153793	1.1932944
sp|P05452|TETN_HUMAN	0.6115608	0.91601294
sp|P02753|RET4_HUMAN	0.61257744	1.1932944
sp|Q9H3H3-1|CK068_HUMAN	0.6128807	1.1932944
sp|Q9Y2J2-4|E41L3_HUMAN	0.61511993	0.656254
sp|P49902-2|5NTC_HUMAN	0.6158638	1.1932944
sp|Q9BXS5-2|AP1M1_HUMA	0.61652756	1.1932944
sp|Q9Y295|DRG1_HUMAN	0.6189785	1.1932944
sp|A0A0C4DH38|HV551_HUM	0.61961555	1.1932944
sp|P63261|ACTG_HUMAN	0.6199379	0.656254
sp|P55265-5|DSRAD_HUMAN	0.6207695	1.1932944
sp|Q9Y285|SYFA_HUMAN	0.62078667	0.656254
sp|Q15005|SPCS2_HUMAN	0.62192726	1.1932944
sp|Q9H4G4|GAPR1_HUMAN	0.62293625	1.1932944
sp|P35542|SAA4_HUMAN	0.6236267	1.1932944
sp|O43681|ASNA_HUMAN	0.62449837	1.1932944
sp|O00560-2|SDCB1_HUMAN	0.6249485	1.1932944
sp|P13284|GILT_HUMAN	0.62683487	0.7827403
sp|P61225|RAP2B_HUMAN	0.6268444	1.1932944
sp|Q10713-2|MPPA_HUMAN	0.6283722	1.1932944
sp|Q9UQ80|PA2G4_HUMAN	0.6287041	0.656254
sp|Q9Y2X3|NOP58_HUMAN	0.62921715	1.1932944
sp|P24557-2|THAS_HUMAN	0.6293678	1.1932944
sp|P0DOX7|IGK_HUMAN	0.6317749	1.1932944
sp|P08779|K1C16_HUMAN	0.6328869	0.656254
sp|P49756|RBM25_HUMAN	0.634634	1.1932944
sp|Q92973-2|TNPO1_HUMA	0.6348038	1.0301651
sp|P61088|UBE2N_HUMAN	0.6380787	0.656254
sp|Q96N66-3|MBOA7_HUM	0.63886833	1.1932944
sp|Q16204|CCDC6_HUMAN	0.6397228	1.1932944
sp|P68371|TBB4B_HUMAN	0.6403332	0.19149946
sp|O00478-2|BT3A3_HUMAN	0.6405239	1.1932944
sp|P60033|CD81_HUMAN	0.64128876	1.1932944
sp|P13746|1A11_HUMAN	0.64155006	1.1932944
sp|P62851|RS25_HUMAN	0.6419411	1.1932944
sp|P14866|HNRPL_HUMAN	0.64264107	1.2095301
sp|P62888|RL30_HUMAN	0.6429901	1.1932944

sp|Q8TCS8|PNPT1_HUMAN	0.64411163	1.1932944
sp|P31946|1433B_HUMAN	0.64465904	0.656254
sp|O95573|ACSL3_HUMAN	0.64756966	0.656254
sp|Q9H9B4|SFXN1_HUMAN	0.6479492	1.1932944
sp|P68036-3|UB2L3_HUMAN	0.6481266	1.1932944
sp|Q8IWB7|WDFY1_HUMAN	0.64865685	1.1932944
sp|P43307|SSRA_HUMAN	0.65036964	1.1932944
sp|O15498-2|YKT6_HUMAN	0.6506462	1.1932944
sp|P61457|PHS_HUMAN	0.65078926	1.1932944
sp|P42025|ACTY_HUMAN	0.651474	1.1932944
sp|Q96A33-2|CCD47_HUMAN	0.65252876	1.1932944
sp|P12694-2|ODBA_HUMAN	0.65256214	1.1932944
sp|P02533|K1C14_HUMAN	0.65488243	0.656254
sp|P54725-2|RD23A_HUMAN	0.65496063	1.1932944
sp|P62834|RAP1A_HUMAN	0.65579605	0.656254
sp|Q96EM0|T3HPD_HUMAN	0.6598568	1.1932944
sp|Q14118|DAG1_HUMAN	0.6616478	1.1932944
sp|Q9NUJ1|ABHDA_HUMAN	0.66171455	1.1932944
sp|P00748|FA12_HUMAN	0.6624279	1.1932944
sp|P01780|HV307_HUMAN	0.66438293	0.656254
sp|Q9UJW0-3|DCTN4_HUMA	0.6646404	1.1932944
sp|Q9H0E2|TOLIP_HUMAN	0.6647701	1.1932944
sp|Q53EL6-2|PDCD4_HUMAN	0.6664963	1.1932944
sp|P16298-2|PP2BB_HUMAN	0.6688118	0.656254
sp|Q9H3K6-2|BOLA2_HUMA	0.66983414	0.656254
sp|P17612|KAPCA_HUMAN	0.670208	0.656254
sp|Q969X5|ERGI1_HUMAN	0.673172	1.1932944
sp|Q8TD19|NEK9_HUMAN	0.6732006	1.1932944
sp|P09543-2|CN37_HUMAN	0.67383194	0.7588735
sp|Q92890-1|UFD1_HUMAN	0.6748562	1.1932944
sp|P02795|MT2_HUMAN	0.67773056	1.1932944
sp|Q96IU4|ABHEB_HUMAN	0.67941666	1.1932944
sp|Q15185-3|TEBP_HUMAN	0.68019104	1.1932944
sp|Q13435|SF3B2_HUMAN	0.6816368	1.1932944
sp|Q08209-2|PP2BA_HUMAN	0.68177986	0.656254
sp|P39023|RL3_HUMAN	0.6840725	1.1932944
sp|Q92552-2|RT27_HUMAN	0.68481636	1.1932944
sp|Q13596-2|SNX1_HUMAN	0.68639755	0.656254
sp|P46779-2|RL28_HUMAN	0.6864166	1.1932944
sp|P61006|RAB8A_HUMAN	0.68741417	0.656254
sp|Q7Z6Z7-2|HUWE1_HUMA	0.6887207	1.1932944
sp|Q9Y570-2|PPME1_HUMA	0.69023705	1.1932944
sp|P06310|KV230_HUMAN	0.69052696	0.656254
sp|P0DJI8|SAA1_HUMAN	0.69184685	0.656254
sp|Q9H9G7-2|AGO3_HUMAN	0.69249344	1.1932944

sp|Q96FV2-2|SCRN2_HUMA	0.69282913	1.1932944
sp|P54578-2|UBP14_HUMAN	0.6934681	1.1932944
sp|P04839|CY24B_HUMAN	0.694252	1.1932944
sp|Q99436|PSB7_HUMAN	0.6961994	1.1505735
sp|A0MZ66-5|SHOT1_HUMA	0.6970558	1.1932944
sp|O43148-2|MCES_HUMAN	0.69890594	1.1932944
sp|P62495-2|ERF1_HUMAN	0.69942665	1.1932944
sp|Q13438-4|OS9_HUMAN	0.6994343	1.1932944
sp|Q9Y6B6|SAR1B_HUMAN	0.70047	1.1932944
sp|P0CG39|POTEJ_HUMAN	0.7012863	0.656254
sp|P33316|DUT_HUMAN	0.7018566	1.1932944
sp|P62633-3|CNBP_HUMAN	0.7019348	1.1932944
sp|Q92688-2|AN32B_HUMA	0.7023716	1.1932944
sp|P11498|PYC_HUMAN	0.7043648	0.6070219
sp|Q04206-3|TF65_HUMAN	0.70454025	1.1932944
sp|P58546|MTPN_HUMAN	0.70586777	1.1932944
sp|O15296|LX15B_HUMAN	0.70641327	1.1932944
sp|P48506|GSH1_HUMAN	0.70656204	1.1932944
sp|Q6UW68|TM205_HUMAN	0.7084274	1.1932944
sp|Q96EP5-2|DAZP1_HUMAN	0.7116585	1.1932944
sp|Q9H8L6|MMRN2_HUMAN	0.7150326	1.1932944
sp|P55854-2|SUMO3_HUMA	0.71567154	0.656254
sp|P01782|HV309_HUMAN	0.71816635	0.656254
sp|Q86UP2-4|KTN1_HUMAN	0.71948624	1.1932944
sp|P61018|RAB4B_HUMAN	0.72076035	1.1932944
sp|Q9TQE0|2B19_HUMAN	0.7211914	0.656254
sp|Q86TX2|ACOT1_HUMAN	0.7248993	1.1932944
sp|Q9BVG4|PBDC1_HUMAN	0.725605	1.1932944
sp|P29992|GNA11_HUMAN	0.7276535	1.1932944
sp|Q8WXF7-2|ATLA1_HUMA	0.72774124	1.1932944
sp|Q9H3U1-2|UN45A_HUMA	0.72802544	1.1932944
sp|O94875-11|SRBS2_HUMA	0.7284603	0.656254
sp|P50440-3|GATM_HUMAN	0.72942734	1.1932944
sp|P06744|G6PI_HUMAN	0.7312832	0.656254
sp|P20338|RAB4A_HUMAN	0.7321396	0.656254
sp|O94804|STK10_HUMAN	0.73238754	0.656254
sp|P62249|RS16_HUMAN	0.73316	1.1932944
sp|P07360|CO8G_HUMAN	0.7342205	1.1932944
sp|Q96FW1|OTUB1_HUMAN	0.7347622	1.1932944
sp|Q09028-3|RBBP4_HUMA	0.7352333	0.656254
sp|Q6PCB0|VWA1_HUMAN	0.7358837	1.1932944
sp|Q13547|HDAC1_HUMAN	0.73594093	0.656254
sp|Q08379|GOGA2_HUMAN	0.73612976	1.1932944
sp|P0C0L5|CO4B_HUMAN	0.7366562	0.656254
sp|P61081|UBC12_HUMAN	0.7367668	1.1932944

sp|O94925-3|GLSK_HUMAN	0.73726654	1.1932944
sp|P60709|ACTB_HUMAN	0.73775864	0.656254
sp|Q9Y4D7-2|PLXD1_HUMAN	0.7397995	1.1932944
sp|A0A075B6P5|KV228_HUM	0.74038696	0.656254
sp|Q02083-2|NAAA_HUMAN	0.7423096	1.1932944
sp|Q9NR19-2|ACSA_HUMAN	0.74518013	1.1932944
sp|Q2TAY7|SMU1_HUMAN	0.7455864	1.1932944
sp|P61224|RAP1B_HUMAN	0.7468672	0.656254
sp|O00468-7|AGRIN_HUMAI	0.7473583	1.1932944
sp|Q96HD1-2|CREL1_HUMAN	0.74762917	1.1932944
sp|P51452-2|DUS3_HUMAN	0.7479782	1.1932944
sp|Q9Y5Z4-2|HEBP2_HUMAN	0.7482872	1.1932944
sp|Q8TDZ2-4|MICA1_HUMAI	0.7483158	1.1932944
sp|Q6UW02|CP20A_HUMAN	0.74832535	1.1932944
sp|P63027|VAMP2_HUMAN	0.74852943	0.656254
sp|O60271-5|JIP4_HUMAN	0.74874115	1.1932944
sp|Q05682-4|CALD1_HUMAN	0.7496891	0.656254
sp|P17301|ITA2_HUMAN	0.75076294	1.1932944
sp|Q8WYA6-2|CTBL1_HUMA	0.75135994	1.1932944
sp|P62304|RUXE_HUMAN	0.7514	1.1932944
sp|P01743|HV146_HUMAN	0.751915	1.1932944
sp|P46736-3|BRCC3_HUMAN	0.7541504	1.1932944
sp|P12235|ADT1_HUMAN	0.75834656	0.656254
sp|Q8NHP8|PLBL2_HUMAN	0.7588997	1.1932944
sp|P36542-2|ATPG_HUMAN	0.7631407	0.656254
sp|P14207|FOLR2_HUMAN	0.7633734	1.1932944
sp|O95425-4|SVIL_HUMAN	0.76340866	1.1932944
sp|Q00169|PIPNA_HUMAN	0.7669487	1.1932944
sp|O15260-2|SURF4_HUMAI	0.7670641	1.1932944
sp|Q9UHD8-3|SEPT9_HUMAI	0.76779366	1.1932944
sp|P36873-2|PP1G_HUMAN	0.7694988	1.1932944
sp|O76003|GLRX3_HUMAN	0.7695217	0.656254
sp|Q99961-2|SH3G1_HUMAI	0.7696972	1.1932944
sp|A0A0C4DH31|HV118_HUN	0.770689	0.656254
sp|P49959-3|MRE11_HUMAI	0.7722473	1.1932944
sp|P31513|FMO3_HUMAN	0.7733917	1.1932944
sp|Q30134|2B18_HUMAN	0.7744179	0.656254
sp|Q9NY15|STAB1_HUMAN	0.77443695	1.1932944
sp|Q99459|CDC5L_HUMAN	0.7797966	1.1932944
sp|Q92820|GGH_HUMAN	0.7808037	1.1932944
sp|P63279|UBC9_HUMAN	0.78323174	1.1932944
sp|P50238|CRIP1_HUMAN	0.7851219	1.1932944
sp|Q15370-2|ELOB_HUMAN	0.7864914	0.656254
sp|P15374|UCHL3_HUMAN	0.78738594	1.1932944
sp|O43396|TXNL1_HUMAN	0.78972435	1.1932944

sp|Q16576-2|RBBP7_HUMAI	0.79021454	0.656254
sp|Q9NP79|VTA1_HUMAN	0.79022217	1.1932944
sp|Q15149-9|PLEC_HUMAN	0.7914486	0.656254
sp|Q9Y3A3-3|PHOCN_HUMA	0.79164505	1.1932944
sp|P49903-2|SPS1_HUMAN	0.7922878	1.1932944
sp|Q9NT62-2|ATG3_HUMAN	0.7925377	1.1932944
sp|P0DP03|HV335_HUMAN	0.79343224	1.1932944
sp|O43252|PAPS1_HUMAN	0.79576874	1.1932944
sp|Q09161|NCBP1_HUMAN	0.79621506	1.1932944
sp|P41240|CSK_HUMAN	0.79632187	1.1932944
sp|Q92747|ARC1A_HUMAN	0.7965641	0.656254
sp|Q9NR31|SAR1A_HUMAN	0.7975445	1.1932944
sp|P31937|3HIDH_HUMAN	0.79896164	1.1932944
sp|P22894|MMP8_HUMAN	0.79927444	1.1932944
sp|P48426-2|PI42A_HUMAN	0.80029106	1.1932944
sp|O14617-4|AP3D1_HUMAI	0.8008089	1.1932944
sp|A1L4H1|SRCRL_HUMAN	0.8047104	1.1932944
sp|P63151-2|2ABA_HUMAN	0.8072376	0.656254
sp|O95352|ATG7_HUMAN	0.8084316	1.1932944
sp|Q9NZL9-2|MAT2B_HUMA	0.80884933	1.1932944
sp|P53990-2|IST1_HUMAN	0.8088913	1.1932944
sp|P36542|ATPG_HUMAN	0.8100624	0.656254
sp|Q9P0K7-2|RAI14_HUMAN	0.81085587	1.1932944
sp|Q5TFE4|NT5D1_HUMAN	0.8114624	1.1932944
sp|Q96P70|IPO9_HUMAN	0.81471825	1.1932944
sp|Q96S97|MYADM_HUMAN	0.8153534	1.1932944
sp|P79483|DRB3_HUMAN	0.81637	0.656254
sp|Q02952-2|AKA12_HUMAI	0.81741905	1.1932944
sp|O60488-2|ACSL4_HUMAN	0.8189888	0.656254
sp|Q9BV20|MTNA_HUMAN	0.8198509	1.1932944
sp|P02746|C1QB_HUMAN	0.8229885	1.1932944
sp|Q9UNS2|CSN3_HUMAN	0.82387733	0.656254
sp|P01624|KV315_HUMAN	0.82536507	1.1932944
sp|P54886-2|P5CS_HUMAN	0.8268013	1.1932944
sp|Q96RU3-3|FNBP1_HUMA	0.82912064	1.1932944
sp|Q16527|CSRP2_HUMAN	0.8297291	1.1932944
sp|Q15172-2|2A5A_HUMAN	0.83234596	1.1932944
sp|Q15642-3|CIP4_HUMAN	0.83239937	0.656254
sp|Q13505-3|MTX1_HUMAN	0.8339186	1.1932944
sp|P01619|KV320_HUMAN	0.8357887	0.656254
sp|P23142-4|FBLN1_HUMAN	0.836113	1.1932944
sp|P57737-4|CORO7_HUMAI	0.8370342	1.1932944
sp|O60825-2|F262_HUMAN	0.84105873	0.656254
sp|P02747|C1QC_HUMAN	0.84189034	1.1932944
sp|Q68EM7-2|RHG17_HUMA	0.84300995	1.1932944

sp|Q13555-7|KCC2G_HUMAN	0.84500504	0.656254
sp|Q96BM9|ARL8A_HUMAN	0.8462162	0.656254
sp|Q9BU23-3|LMF2_HUMAN	0.8479748	1.1932944
sp|P30419-2|NMT1_HUMAN	0.8483448	1.1932944
sp|P20292|AL5AP_HUMAN	0.84898853	0.7827403
sp|P54619-2|AAKG1_HUMAN	0.85053253	1.1932944
sp|Q8TE77-3|SSH3_HUMAN	0.8508854	1.1932944
sp|O75688|PPM1B_HUMAN	0.8510399	1.1932944
sp|P22352|GPX3_HUMAN	0.8517246	1.1932944
sp|P02751-15|FINC_HUMAN	0.85287094	0.656254
sp|Q99538-2|LGMN_HUMAN	0.85765076	1.1932944
sp|O00487|PSDE_HUMAN	0.8579426	1.1932944
sp|Q13043|STK4_HUMAN	0.86018753	1.1932944
sp|Q9UBI6|GBG12_HUMAN	0.8608093	1.1932944
sp|Q13813-3|SPTN1_HUMAN	0.8629608	1.1932944
sp|Q5VW32|BROX_HUMAN	0.8652382	1.1932944
sp|O95373|IPO7_HUMAN	0.86610794	1.1932944
sp|Q6DKJ4|NXN_HUMAN	0.8678398	1.1932944
sp|O94811|TPPP_HUMAN	0.8687048	1.1932944
sp|Q8N9N7|LRC57_HUMAN	0.8689232	1.1932944
sp|Q9Y2B0|CNPY2_HUMAN	0.871068	1.1932944
sp|P55058|PLTP_HUMAN	0.87511253	0.656254
sp|Q14697|GANAB_HUMAN	0.87521935	1.1932944
sp|Q92947|GCDH_HUMAN	0.884779	1.1932944
sp|P08134|RHOC_HUMAN	0.8859024	1.1932944
sp|A1L0T0|ILVBL_HUMAN	0.88726425	1.1932944
sp|P55263|ADK_HUMAN	0.8873997	1.1932944
sp|P01834|IGKC_HUMAN	0.8881016	1.1932944
sp|O76011|KRT34_HUMAN	0.8889885	0.656254
sp|Q96DG6|CMBL_HUMAN	0.8981781	1.1932944
sp|P04350|TBB4A_HUMAN	0.89946175	0.656254
sp|Q02750|MP2K1_HUMAN	0.90161896	1.1932944
sp|Q96BW5-2|PTER_HUMAN	0.9018669	1.1932944
sp|Q9HAV0|GBB4_HUMAN	0.9024277	1.1932944
sp|Q8N1B4-2|VPS52_HUMA	0.90395355	1.1932944
sp|P55039|DRG2_HUMAN	0.90400696	1.1932944
sp|Q99426|TBCB_HUMAN	0.90447426	1.1932944
sp|P30046|DOPD_HUMAN	0.90597343	1.1932944
sp|P32456|GBP2_HUMAN	0.9076538	1.1932944
sp|Q9Y281|COF2_HUMAN	0.90880585	1.1932944
sp|Q13464|ROCK1_HUMAN	0.91028595	0.656254
sp|Q9H0R4|HDHD2_HUMAN	0.9131012	1.1932944
sp|O60313-10|OPA1_HUMAI	0.9160633	1.1932944
sp|P35813-3|PPM1A_HUMAI	0.9239445	0.656254
sp|P06396|GELS_HUMAN	0.92774963	0.656254

sp|P67775-2|PP2AA_HUMAN	0.9277649	0.656254
sp|Q8WZA0-2|LZIC_HUMAN	0.92811966	1.1932944
sp|P48741|HSP77_HUMAN	0.92988586	0.656254
sp|Q13496|MTM1_HUMAN	0.93530464	1.1932944
sp|P31321|KAP1_HUMAN	0.93650246	0.656254
sp|Q8TAT6-2|NPL4_HUMAN	0.93733025	1.1932944
sp|Q8N8S7-2|ENAH_HUMAN	0.9389992	1.1932944
sp|Q92629-2|SGCD_HUMAN	0.9430027	0.656254
sp|Q14108-2|SCRB2_HUMAN	0.94644547	1.1932944
sp|P12931-2|SRC_HUMAN	0.94823456	1.1932944
sp|P31946-2|1433B_HUMAN	0.9494953	0.656254
sp|P12814|ACTN1_HUMAN	0.9498558	0.656254
sp|Q7L576|CYFP1_HUMAN	0.9504776	0.656254
sp|Q13188|STK3_HUMAN	0.9505768	0.656254
sp|Q13885|TBB2A_HUMAN	0.9606228	0.656254
sp|Q15435|PP1R7_HUMAN	0.9624376	1.1932944
sp|P49841-2|GSK3B_HUMAN	0.96341324	1.1932944
sp|Q7LG56-6|RIR2B_HUMAN	0.969059	1.1932944
sp|Q5JPE7-2|NOMO2_HUMA	0.97125816	0.656254
sp|O95394-3|AGM1_HUMAN	0.9729023	1.1932944
sp|Q13310-2|PABP4_HUMAN	0.97660065	0.656254
sp|P00736|C1R_HUMAN	0.9849682	1.1932944
sp|O94788-4|AL1A2_HUMAN	0.9871521	0.656254
sp|O95816|BAG2_HUMAN	0.98739433	1.1932944
sp|Q9P289|STK26_HUMAN	0.99326324	0.656254
sp|Q8IUZ5|AT2L2_HUMAN	0.9977188	1.1932944
sp|Q9NWV4|CZIB_HUMAN	1.0192261	1.1932944
sp|P31323|KAP3_HUMAN	1.0231533	1.1932944
sp|Q9UQ16-2|DYN3_HUMAN	1.0273991	0.656254
sp|P40261|NNMT_HUMAN	1.0342674	1.1932944
sp|O75116|ROCK2_HUMAN	1.0586624	1.1932944
sp|Q14139-2|UBE4A_HUMA	1.0587826	1.1932944
sp|Q9GZM7-3|TINAL_HUMA	1.0591831	1.1932944
sp|O00186|STXB3_HUMAN	1.0605698	1.1932944
sp|P14324|FPPS_HUMAN	1.061451	1.1932944
sp|P00746|CFAD_HUMAN	1.0689907	1.1932944
sp|Q14BN4-2|SLMAP_HUMA	1.0704193	0.656254
sp|A5A3E0|POTEF_HUMAN	1.0800667	0.656254
sp|Q9UBB4-2|ATX10_HUMA	1.0924835	1.1932944
sp|Q14194-2|DPYL1_HUMAN	1.0961552	0.656254
sp|Q13642-1|FHL1_HUMAN	1.105835	0.656254
sp|Q14697-2|GANAB_HUMA	1.1162376	0.656254
sp|O94832|MYO1D_HUMAN	1.1219692	1.1932944
sp|Q96JJ3-3|ELMO2_HUMAN	1.1343489	0.656254
sp|Q15417|CNN3_HUMAN	1.1371155	1.1932944

sp|Q8IUD2-2|RB6I2_HUMAN	1.148016	0.656254
sp|Q14558-2|KPRA_HUMAN	1.1644859	0.656254
sp|P61601|NCALD_HUMAN	1.1730728	0.656254
sp|P54652|HSP72_HUMAN	1.1732521	0.656254
sp|Q06278|AOXA_HUMAN	1.1802559	1.1932944
sp|Q9ULC5-3|ACSL5_HUMAN	1.1862621	0.656254
sp|Q9BWD1|THIC_HUMAN	1.1912355	0.656254
sp|P02461|CO3A1_HUMAN	1.1973248	1.1932944
sp|Q14651|PLSI_HUMAN	1.2018976	0.656254
sp|Q92599-2|SEPT8_HUMAN	1.2073612	1.1932944
sp|Q8WUP2-3|FBLI1_HUMA	1.2081871	1.1932944
sp|O00499-10|BIN1_HUMAN	1.2191963	1.1932944
sp|P28161|GSTM2_HUMAN	1.2301655	1.1932944
sp|Q01518|CAP1_HUMAN	1.2425175	0.656254
sp|P12111-4|CO6A3_HUMAN	1.2472153	0.656254
sp|Q31612|1B73_HUMAN	1.2532406	0.656254
sp|P35612-2|ADDB_HUMAN	1.2534161	0.656254
sp|P0C0L4|CO4A_HUMAN	1.2758293	1.1932944
sp|P51692|STA5B_HUMAN	1.30937	0.656254
sp|P11217-2|PYGM_HUMAN	1.315197	0.656254
sp|O75881|CP7B1_HUMAN	1.3291264	1.1932944
sp|P0CG38|POTEI_HUMAN	1.3546848	0.656254
sp|Q0ZGT2-4|NEXN_HUMAN	1.3724575	0.656254
sp|Q01518-2|CAP1_HUMAN	1.375186	0.656254
sp|P12814-2|ACTN1_HUMAN	1.3782387	1.1932944
sp|O94911-3|ABCA8_HUMA	1.4074078	1.1932944
sp|Q3SY69|AL1L2_HUMAN	1.4197178	1.1932944
sp|P46821|MAP1B_HUMAN	1.4627838	1.1932944
sp|P55083-2|MFAP4_HUMA	1.5163555	1.1932944
sp|O14787-2|TNPO2_HUMA	1.5996323	0.656254
sp|Q9Y4G6|TLN2_HUMAN	1.6377687	1.1932944
sp|O95050-2|INMT_HUMAN	3.6173725	1.1932944
