# Supplementary material for: Peptidylarginine deiminase 2 citrullinates MZB1 and promotes the secretion of IgM and IgA
Source: Front Immunol. 2023 Nov 29;14:1290585. doi: 10.3389/fimmu.2023.1290585 (PMC10716219; doi:10.3389/fimmu.2023.1290585)
Supplement: Supplementary file 11 [file DataSheet_11.pdf]

Supplemental Table 11: COPD2 vs controls

Accession #	Fold Change	p value (-log10)
sp|P59665|DEF1_HUMAN	-1.1434555	2.1818786
sp|P48735-2|IDHP_HUMAN	-1.1072845	4.051346
sp|Q96TA1-2|NIBL1_HUMAN	-1.0869656	2.7624686
sp|P99999|CYC_HUMAN	-1.0351162	2.1818786
sp|P05109|S10A8_HUMAN	-1.001688	3.5908275
sp|P26440|IVD_HUMAN	-0.9941597	1.6960104
sp|O75874|IDHC_HUMAN	-0.9063435	4.964395
sp|Q86VB7-2|C163A_HUMAN	-0.8977127	8.558914
sp|P51149|RAB7A_HUMAN	-0.8947601	4.12427
sp|P31146|COR1A_HUMAN	-0.8842259	6.409319
sp|P05141|ADT2_HUMAN	-0.8831215	2.6576471
sp|Q16698-2|DECR_HUMAN	-0.7982159	3.5844223
sp|P08311|CATG_HUMAN	-0.7981072	4.051346
sp|P84090|ERH_HUMAN	-0.7936516	2.1818786
sp|P17844-2|DDX5_HUMAN	-0.7801628	4.051346
sp|Q00325-2|MPCP_HUMAN	-0.7710629	3.1266599
sp|P05164-3|PERM_HUMAN	-0.7599506	9.782528
sp|P51659|DHB4_HUMAN	-0.7505779	12.077333
sp|Q02318|CP27A_HUMAN	-0.7488728	1.6960104
sp|P51649-2|SSDH_HUMAN	-0.7459621	2.1818786
sp|Q9P2R7-2|SUCB1_HUMAN	-0.7422466	3.1266599
sp|P11413-2|G6PD_HUMAN	-0.737751	4.3153405
sp|P14866-2|HNRPL_HUMAN	-0.7370338	1.6960104
sp|P43490|NAMPT_HUMAN	-0.7261505	6.7073655
sp|O00159-3|MYO1C_HUMAN	-0.7256584	7.665951
sp|P40121|CAPG_HUMAN	-0.7208271	5.259211
sp|Q13217|DNJC3_HUMAN	-0.7165871	2.1818786
sp|P02786|TFR1_HUMAN	-0.7158203	4.051346
sp|Q96TC7|RMD3_HUMAN	-0.7115784	1.6960104
sp|P17213|BPI_HUMAN	-0.7111015	2.1818786
sp|Q02218-2|ODO1_HUMAN	-0.7066155	4.051346
sp|P31949|S10AB_HUMAN	-0.7009144	2.6576471
sp|Q8NBX0|SCPDL_HUMAN	-0.7007408	1.6960104
sp|Q9HC35-2|EMAL4_HUMAN	-0.7004871	2.6576471
sp|P11177-3|ODPB_HUMAN	-0.6996956	3.5908275
sp|P07858|CATB_HUMAN	-0.6947651	4.3153405
sp|P12429|ANXA3_HUMAN	-0.6852989	7.36771
sp|Q9Y5P6-2|GMPPB_HUMAN	-0.6823635	1.6960104
sp|P06733|ENOA_HUMAN	-0.6767731	11.808928
sp|P04179-4|SODM_HUMAN	-0.6729813	3.1266599
sp|O00743-3|PPP6_HUMAN	-0.6708756	1.6960104
sp|P08559-2|ODPA_HUMAN	-0.662096	4.509013
sp|Q02543|RL18A_HUMAN	-0.6577835	2.9807727

sp|O15143|ARC1B_HUMAN	-0.6547623	3.7803535
sp|P07954-2|FUMH_HUMAN	-0.6531849	2.6576471
sp|O75390|CISY_HUMAN	-0.6417103	5.417897
sp|Q9P258|RCC2_HUMAN	-0.6400223	2.1818786
sp|P12821-2|ACE_HUMAN	-0.6392441	2.6576471
sp|P21397|AOFA_HUMAN	-0.638443	5.7968407
sp|P05107|ITB2_HUMAN	-0.634552	3.0019732
sp|P04083|ANXA1_HUMAN	-0.6333618	9.57498
sp|P84103-2|SRSF3_HUMAN	-0.6314449	3.1266599
sp|P62263|RS14_HUMAN	-0.630249	1.6960104
sp|P61626|LYSC_HUMAN	-0.624691	2.6576471
sp|P33121-3|ACSL1_HUMAN	-0.6244488	1.5646582
sp|Q6NUK1-2|SCMC1_HUMAN	-0.6216774	2.6576471
sp|Q9BW30|TPPP3_HUMAN	-0.6205025	2.9807727
sp|Q8NF37|PCAT1_HUMAN	-0.6150093	1.6997428
sp|P18428|LBP_HUMAN	-0.612915	1.6960104
sp|P53597|SUCA_HUMAN	-0.6098576	1.6960104
sp|P07355-2|ANXA2_HUMAN	-0.6084957	4.628043
sp|Q13510-2|ASAH1_HUMAN	-0.6075344	5.792018
sp|P00505|AATM_HUMAN	-0.602478	5.652275
sp|P54886-2|P5CS_HUMAN	-0.5996552	1.6960104
sp|P62917|RL8_HUMAN	-0.5975304	1.4278674
sp|P80188|NGAL_HUMAN	-0.5926628	2.1800864
sp|P13073|COX41_HUMAN	-0.5902176	1.6960104
sp|Q99715-4|COCA1_HUMAN	-0.5862274	1.6960104
sp|P68104|EF1A1_HUMAN	-0.5826492	3.5908275
sp|P49327|FAS_HUMAN	-0.5810318	10.275428
sp|P19367-2|HXK1_HUMAN	-0.5803223	7.8127522
sp|P25774|CATS_HUMAN	-0.5785027	1.6960104
sp|Q9Y3Z3|SAMH1_HUMAN	-0.5771332	6.782829
sp|P62826|RAN_HUMAN	-0.5769367	1.9413493
sp|P50995-2|ANX11_HUMAN	-0.5757771	5.7968407
sp|Q9HB40|RISC_HUMAN	-0.5726852	1.6960104
sp|P31040|SDHA_HUMAN	-0.5721893	4.372737
sp|P62906|RL10A_HUMAN	-0.5705013	2.9807727
sp|Q92888-2|ARHG1_HUMAN	-0.5704041	1.6960104
sp|P13686|PPA5_HUMAN	-0.5698643	1.6960104
sp|Q02252-2|MMSA_HUMAN	-0.5689354	4.051346
sp|P01903|DRA_HUMAN	-0.5687962	2.1818786
sp|P26038|MOES_HUMAN	-0.5682888	14.398287
sp|P51648-2|AL3A2_HUMAN	-0.5680809	4.051346
sp|P63244|RACK1_HUMAN	-0.5668678	5.9217625
sp|P05091|ALDH2_HUMAN	-0.5667572	9.794418
sp|Q8IV08|PLD3_HUMAN	-0.5657501	1.6960104
sp|P23786|CPT2_HUMAN	-0.5654602	1.3815327

sp|P53007|TXTP_HUMAN	-0.5623589	2.6576471
sp|P18077|RL35A_HUMAN	-0.5607681	2.1818786
sp|Q92841-3|DDX17_HUMAN	-0.5592842	4.400762
sp|P49756|RBM25_HUMAN	-0.5578699	1.6960104
sp|P40429|RL13A_HUMAN	-0.5558682	1.6960104
sp|Q9BXP5-2|SRRT_HUMAN	-0.5548058	2.1818786
sp|P41218|MNDA_HUMAN	-0.5538101	1.9647322
sp|P23246|SFPQ_HUMAN	-0.5528526	4.964395
sp|Q9NZM1-6|MYOF_HUMAN	-0.5517998	8.341435
sp|P06702|S10A9_HUMAN	-0.5512161	3.4656596
sp|Q6PIU2-2|NCEH1_HUMAN	-0.5505581	2.432837
sp|P43304|GPDM_HUMAN	-0.5500031	2.441399
sp|P53621-2|COPA_HUMAN	-0.5486031	5.2716713
sp|P53396-2|ACLY_HUMAN	-0.548542	3.1092827
sp|P63000-2|RAC1_HUMAN	-0.5475597	1.6960104
sp|P13804|ETFA_HUMAN	-0.5473862	4.509013
sp|P31947|1433S_HUMAN	-0.5462494	2.1818786
sp|P61106|RAB14_HUMAN	-0.5427322	4.12427
sp|P49913|CAMP_HUMAN	-0.542551	1.6960104
sp|Q16762|THTR_HUMAN	-0.5382919	1.9647322
sp|P40926|MDHM_HUMAN	-0.534195	8.061325
sp|O95994|AGR2_HUMAN	-0.5324917	2.6576471
sp|P23381|SYWC_HUMAN	-0.5318985	6.3098373
sp|O43837|IDH3B_HUMAN	-0.5309601	1.6960104
sp|Q9Y6N5|SQOR_HUMAN	-0.5304413	4.1814356
sp|Q9UFN0|NPS3A_HUMAN	-0.5295658	1.6960104
sp|P07686|HEXB_HUMAN	-0.5278606	3.1266599
sp|P07339|CATD_HUMAN	-0.5274468	5.533072
sp|P09525|ANXA4_HUMAN	-0.5254059	6.9721084
sp|Q9NNW7|TRXR2_HUMAN	-0.5228291	2.1818786
sp|P08575-10|PTPRC_HUMAN	-0.5202255	3.8337784
sp|Q9UL25|RAB21_HUMAN	-0.5200005	2.6576471
sp|P11940-2|PABP1_HUMAN	-0.5192127	1.6960104
sp|P09467|F16P1_HUMAN	-0.516531	5.417897
sp|P26641|EF1G_HUMAN	-0.5132008	4.3100786
sp|Q12907|LMAN2_HUMAN	-0.5090218	1.7165122
sp|Q10713|MPPA_HUMAN	-0.5083714	1.9190748
sp|Q9BR76|COR1B_HUMAN	-0.5082703	3.5908275
sp|Q9H0U4|RAB1B_HUMAN	-0.5078335	1.6960104
sp|P61158|ARP3_HUMAN	-0.5016403	8.365451
sp|P05023|AT1A1_HUMAN	-0.5005245	8.558914
sp|Q13347|EIF3I_HUMAN	-0.4990711	2.3143692
sp|P46777|RL5_HUMAN	-0.4988174	4.285869
sp|P07384|CAN1_HUMAN	-0.4967613	7.508114
sp|P29144|TPP2_HUMAN	-0.4958153	1.6960104

sp|O75695|XRP2_HUMAN	-0.4931946	1.6960104
sp|Q13418|ILK_HUMAN	-0.4916821	3.103845
sp|P09211|GSTP1_HUMAN	-0.4914589	4.0084753
sp|Q8WXF1|PSPC1_HUMAN	-0.491024	2.6576471
sp|P63092-3|GNAS2_HUMAN	-0.4904347	2.9310203
sp|P35268|RL22_HUMAN	-0.4868946	1.4104178
sp|Q15907-2|RB11B_HUMAN	-0.4847012	3.4656596
sp|P23284|PPIB_HUMAN	-0.4833088	4.220149
sp|Q13045-2|FLII_HUMAN	-0.4831924	2.6576471
sp|P05787-2|K2C8_HUMAN	-0.4823704	12.761465
sp|P10155|RO60_HUMAN	-0.4814701	3.316884
sp|P62873|GBB1_HUMAN	-0.4808426	3.4656596
sp|P29350-3|PTN6_HUMAN	-0.4790278	2.4829872
sp|P40939|ECHA_HUMAN	-0.4789772	5.8299637
sp|Q9UBQ0-2|VPS29_HUMAN	-0.4781075	1.6960104
sp|P07099|HYEP_HUMAN	-0.4773693	5.824478
sp|P10515|ODP2_HUMAN	-0.4772053	2.838374
sp|P05362|ICAM1_HUMAN	-0.475029	4.1861625
sp|P50213|IDH3A_HUMAN	-0.4747849	3.1266599
sp|Q9ULV4-3|COR1C_HUMAN	-0.473402	2.7624686
sp|P61421|VA0D1_HUMAN	-0.4716587	3.4656596
sp|P52272-2|HNRPM_HUMAN	-0.469883	5.9217625
sp|Q07020-2|RL18_HUMAN	-0.4688282	1.6960104
sp|P47897|SYQ_HUMAN	-0.4674492	2.6576471
sp|P13639|EF2_HUMAN	-0.4653492	11.32408
sp|P29401-2|TKT_HUMAN	-0.4648171	12.187731
sp|P13796|PLSL_HUMAN	-0.4644299	10.021521
sp|Q969X5-2|ERGI1_HUMAN	-0.4597931	1.6960104
sp|P23396|RS3_HUMAN	-0.4580097	6.815099
sp|Q96HE7|ERO1A_HUMAN	-0.4579983	1.6960104
sp|Q8NBJ5|GT251_HUMAN	-0.4570656	1.6960104
sp|Q9Y3I0|RTCB_HUMAN	-0.4568558	3.5908275
sp|Q9BUJ2-4|HNRL1_HUMAN	-0.4565067	3.673847
sp|P29590|PML_HUMAN	-0.4563885	3.656485
sp|Q9H8H3|MET7A_HUMAN	-0.4526634	1.6960104
sp|Q15233|NONO_HUMAN	-0.4511871	3.2925725
sp|P11021|BIP_HUMAN	-0.4504318	13.726703
sp|O43813|LANC1_HUMAN	-0.4503956	1.6960104
sp|P61247|RS3A_HUMAN	-0.4492722	3.9417877
sp|Q96AG4|LRC59_HUMAN	-0.4491272	1.6997428
sp|P60953|CDC42_HUMAN	-0.4485779	2.9807727
sp|P46781|RS9_HUMAN	-0.4474907	2.9807727
sp|P60842|IF4A1_HUMAN	-0.4467735	3.412591
sp|P20700|LMNB1_HUMAN	-0.4450245	13.583522
sp|P30740|ILEU_HUMAN	-0.4449635	3.5908275

sp|P36578|RL4_HUMAN	-0.4425507	3.5908275
sp|P08621-3|RU17_HUMAN	-0.4421024	2.6576471
sp|P11310-2|ACADM_HUMAN	-0.4419594	3.1208909
sp|P62249|RS16_HUMAN	-0.4416485	1.6960104
sp|O43760-2|SNG2_HUMAN	-0.4400826	1.6960104
sp|Q8TCJ2|STT3B_HUMAN	-0.438797	1.6960104
sp|P00558|PGK1_HUMAN	-0.4378834	7.019849
sp|P35241|RADI_HUMAN	-0.437602	2.441399
sp|P32455|GBP1_HUMAN	-0.4372253	2.6576471
sp|P62805|H4_HUMAN	-0.4368134	2.5815322
sp|Q13011|ECH1_HUMAN	-0.4362221	3.887875
sp|Q12931-2|TRAP1_HUMAN	-0.435627	1.6960104
sp|Q07955-3|SRSF1_HUMAN	-0.4353027	4.509013
sp|O00299|CLIC1_HUMAN	-0.4350395	5.919944
sp|P60903|S10AA_HUMAN	-0.4347076	1.6960104
sp|P16435|NCPR_HUMAN	-0.4338694	3.7726574
sp|P10253|LYAG_HUMAN	-0.4337273	3.1266599
sp|Q9NSE4|SYIM_HUMAN	-0.4320259	3.417183
sp|P21796|VDAC1_HUMAN	-0.4316711	3.8106754
sp|P55884-2|EIF3B_HUMAN	-0.4310894	2.3143692
sp|P04080|CYTB_HUMAN	-0.427639	1.6960104
sp|P09651-3|ROA1_HUMAN	-0.4261437	1.6960104
sp|P08754|GNAI3_HUMAN	-0.4227409	1.7590232
sp|O75643|U520_HUMAN	-0.4215488	1.6960104
sp|O75083|WDR1_HUMAN	-0.4203568	8.974431
sp|Q96KP4|CNDP2_HUMAN	-0.4193516	5.8986344
sp|P15311|EZRI_HUMAN	-0.4188862	4.3687334
sp|P38646|GRP75_HUMAN	-0.4182358	7.2959604
sp|P11142|HSP7C_HUMAN	-0.4178524	7.238645
sp|P24539|AT5F1_HUMAN	-0.4173355	3.5908275
sp|P16615|AT2A2_HUMAN	-0.4162216	6.409319
sp|P08865|RSSA_HUMAN	-0.4154778	4.509013
sp|Q00839|HNRPU_HUMAN	-0.413723	3.759042
sp|Q9UMS4|PRP19_HUMAN	-0.4132004	2.9807727
sp|P51148-2|RAB5C_HUMAN	-0.412838	1.6960104
sp|Q14764|MVP_HUMAN	-0.4122639	9.594336
sp|P20073-2|ANXA7_HUMAN	-0.4099999	3.103845
sp|P68366-2|TBA4A_HUMAN	-0.4093037	1.3815327
sp|P0DPI2-2|GAL3A_HUMAN	-0.4089451	1.6960104
sp|Q92973-2|TNPO1_HUMAN	-0.4086609	2.1818786
sp|Q04917|1433F_HUMAN	-0.4082508	4.0084753
sp|P26639-2|SYTC_HUMAN	-0.4080772	3.8106754
sp|P00167-2|CYB5_HUMAN	-0.407259	2.1818786
sp|P59998|ARPC4_HUMAN	-0.4071388	2.6576471
sp|P35914|HMGCL_HUMAN	-0.4069614	2.6576471

sp|P30050|RL12_HUMAN	-0.4068279	1.9647322
sp|Q9H3N1|TMX1_HUMAN	-0.4063931	1.6960104
sp|Q9NZN4|EHD2_HUMAN	-0.4058056	4.936604
sp|P49748-2|ACADV_HUMAN	-0.4052429	9.181708
sp|P42330|AK1C3_HUMAN	-0.4044123	2.7624686
sp|P50395|GDIB_HUMAN	-0.4021492	6.044454
sp|P53004|BIEA_HUMAN	-0.401516	3.0251918
sp|Q15181|IPYR_HUMAN	-0.4010353	1.6604291
sp|P01833|PIGR_HUMAN	-0.4009476	3.734273
sp|Q00610-2|CLH1_HUMAN	-0.400362	9.515519
sp|P14618|KPYM_HUMAN	-0.3996773	2.3143692
sp|P23368|MAOM_HUMAN	-0.3987694	2.653196
sp|P60900|PSA6_HUMAN	-0.3982277	4.4091725
sp|P07237|PDIA1_HUMAN	-0.3980484	12.207023
sp|P39023|RL3_HUMAN	-0.3972645	2.1818786
sp|Q13724-2|MOGS_HUMAN	-0.3948135	3.1266599
sp|Q9HCC0|MCCB_HUMAN	-0.3939247	2.8384566
sp|P28838-2|AMPL_HUMAN	-0.3925266	8.489705
sp|P61019|RAB2A_HUMAN	-0.3914127	2.1404836
sp|P49407-2|ARRB1_HUMAN	-0.3900051	2.6576471
sp|Q16629-2|SRSF7_HUMAN	-0.389473	1.9647322
sp|Q9NQR4|NIT2_HUMAN	-0.3880844	2.6576471
sp|Q01469|FABP5_HUMAN	-0.3876438	4.051346
sp|Q86UX7-2|URP2_HUMAN	-0.387598	3.0824287
sp|P46782|RS5_HUMAN	-0.3872948	4.051346
sp|P61604|CH10_HUMAN	-0.3871746	3.1266599
sp|P35606|COPB2_HUMAN	-0.3865624	4.953735
sp|P07910-2|HNRPC_HUMAN	-0.3860493	1.5646582
sp|Q92597|NDRG1_HUMAN	-0.3853073	1.5646582
sp|P15121|ALDR_HUMAN	-0.3845635	3.4979637
sp|Q9Y383-3|LC7L2_HUMAN	-0.38414	1.6960104
sp|P07203|GPX1_HUMAN	-0.3822899	1.5567774
sp|P30048-2|PRDX3_HUMAN	-0.3822155	2.6576471
sp|Q8N335|GPD1L_HUMAN	-0.3819771	1.6960104
sp|P52209-2|6PGD_HUMAN	-0.3806915	4.773267
sp|P14625|ENPL_HUMAN	-0.3805542	10.328907
sp|Q13451|FKBP5_HUMAN	-0.3801727	1.560883
sp|Q13425|SNTB2_HUMAN	-0.3800755	2.1818786
sp|P00966|ASSY_HUMAN	-0.3796864	1.827201
sp|P12081-4|SYHC_HUMAN	-0.3787422	1.6960104
sp|Q99798|ACON_HUMAN	-0.3785954	5.4543324
sp|P22897|MRC1_HUMAN	-0.3781967	4.9245296
sp|Q02878|RL6_HUMAN	-0.3771515	1.6960104
sp|P0DMV9|HS71B_HUMAN	-0.375473	8.238753
sp|Q99829|CPNE1_HUMAN	-0.3744144	1.6092666

sp|P26373|RL13_HUMAN	-0.3738422	1.9647322
sp|P49411|EFTU_HUMAN	-0.3724957	4.8244376
sp|P27361|MK03_HUMAN	-0.3724823	1.6960104
sp|O43776|SYNC_HUMAN	-0.3720589	1.6295799
sp|Q99536|VAT1_HUMAN	-0.3717194	2.816493
sp|Q9UL18|AGO1_HUMAN	-0.3711071	1.6960104
sp|Q9UNM6-2|PSD13_HUMAN	-0.3706589	1.8444856
sp|P09622|DLDH_HUMAN	-0.3704605	3.416951
sp|Q96I99|SUCB2_HUMAN	-0.3703098	4.509013
sp|Q8WUM4|PDC6I_HUMAN	-0.3702126	4.1549106
sp|P09960|LKHA4_HUMAN	-0.3700733	7.430463
sp|P25788-2|PSA3_HUMAN	-0.3695736	2.4829872
sp|O00231-2|PSD11_HUMAN	-0.3693085	2.1818786
sp|Q9Y5S9-2|RBM8A_HUMAN	-0.3689995	1.6960104
sp|P24752|THIL_HUMAN	-0.3687515	5.161142
sp|Q9NYU2-2|UGGG1_HUMAN	-0.3685074	4.5929456
sp|P09668|CATH_HUMAN	-0.3684788	3.0251918
sp|Q9NZ08-2|ERAP1_HUMAN	-0.3663139	2.923088
sp|P17655|CAN2_HUMAN	-0.366127	4.229326
sp|Q03135|CAV1_HUMAN	-0.3654747	2.1818786
sp|P53634|CATC_HUMAN	-0.3647728	1.4104178
sp|P55735-2|SEC13_HUMAN	-0.3631249	2.1818786
sp|A0AVT1|UBA6_HUMAN	-0.3627205	1.4716977
sp|P13667|PDIA4_HUMAN	-0.3616791	10.720789
sp|Q13740-2|CD166_HUMAN	-0.3608437	4.285869
sp|P51858|HDGF_HUMAN	-0.3607369	2.1818786
sp|Q15393|SF3B3_HUMAN	-0.3606186	3.1266599
sp|P18124|RL7_HUMAN	-0.3593712	1.8444856
sp|Q1KMD3|HNRL2_HUMAN	-0.3592033	4.628043
sp|P63104|1433Z_HUMAN	-0.3578472	4.4116287
sp|P30040|ERP29_HUMAN	-0.3577328	2.1800864
sp|P10768|ESTD_HUMAN	-0.35639	2.3143692
sp|Q9BS26|ERP44_HUMAN	-0.3555679	3.316884
sp|Q15084-2|PDIA6_HUMAN	-0.3552761	6.0532455
sp|P42765|THIM_HUMAN	-0.3552742	4.599569
sp|P16278-2|BGAL_HUMAN	-0.3551617	2.6576471
sp|P04843|RPN1_HUMAN	-0.3549614	4.870417
sp|P49189|AL9A1_HUMAN	-0.3545303	5.490865
sp|P50453|SPB9_HUMAN	-0.3541489	3.5908275
sp|P28482|MK01_HUMAN	-0.3537369	2.6576471
sp|P07737|PROF1_HUMAN	-0.3532639	6.359477
sp|P84095|RHOG_HUMAN	-0.3529072	1.6960104
sp|P30101|PDIA3_HUMAN	-0.3509502	10.661194
sp|P60228|EIF3E_HUMAN	-0.3508472	1.8604537
sp|P55060-3|XPO2_HUMAN	-0.3505364	1.9951487

sp|P22626|ROA2_HUMAN	-0.3502598	6.2597647
sp|O43707|ACTN4_HUMAN	-0.3498097	12.6026
sp|P46940|IQGA1_HUMAN	-0.3497829	12.901511
sp|Q9P0L0-2|VAPA_HUMAN	-0.3490028	2.9807727
sp|P31943|HNRH1_HUMAN	-0.348753	2.5644996
sp|P15880|RS2_HUMAN	-0.3482781	1.7590232
sp|P02750|A2GL_HUMAN	-0.3478355	3.1266599
sp|P51991|ROA3_HUMAN	-0.3475857	1.7590232
sp|P35237|SPB6_HUMAN	-0.347292	4.7853546
sp|Q9HDC9|APMAP_HUMAN	-0.3464394	5.241378
sp|P50914|RL14_HUMAN	-0.3453312	1.6960104
sp|Q99873-3|ANM1_HUMAN	-0.3441849	1.8444856
sp|P38919|IF4A3_HUMAN	-0.3439884	2.987788
sp|P62258|1433E_HUMAN	-0.3426476	2.1818786
sp|Q99714|HCD2_HUMAN	-0.3424492	3.5908275
sp|P46063|RECQ1_HUMAN	-0.3418617	2.6576471
sp|Q02978-2|M2OM_HUMAN	-0.3415833	1.6960104
sp|Q96RQ3|MCCA_HUMAN	-0.3407764	1.7590232
sp|P22314|UBA1_HUMAN	-0.3392563	4.824669
sp|Q16543|CDC37_HUMAN	-0.3392239	4.3267694
sp|P54136|SYRC_HUMAN	-0.3386231	2.6576471
sp|P08727|K1C19_HUMAN	-0.3370667	8.999274
sp|P08236-2|BGLR_HUMAN	-0.3369751	1.5646582
sp|Q08211|DHX9_HUMAN	-0.3366089	5.688027
sp|P63096|GNAI1_HUMAN	-0.336277	1.4104178
sp|Q9NSD9|SYFB_HUMAN	-0.3355141	2.1818786
sp|O43143|DHX15_HUMAN	-0.3352356	1.6997428
sp|P04066|FUCO_HUMAN	-0.3346691	1.6960104
sp|Q15109-10|RAGE_HUMAN	-0.3343773	1.7590232
sp|Q9Y678|COPG1_HUMAN	-0.3343716	5.1649675
sp|P51572-2|BAP31_HUMAN	-0.3336945	3.5908275
sp|P35270|SPRE_HUMAN	-0.3336182	1.4278674
sp|P14314-2|GLU2B_HUMAN	-0.3323975	3.5908275
sp|Q9UHQ9|NB5R1_HUMAN	-0.3304615	1.9647322
sp|P10606|COX5B_HUMAN	-0.3299446	1.4104178
sp|P63010-2|AP2B1_HUMAN	-0.3294354	1.6092666
sp|P42224-2|STAT1_HUMAN	-0.326128	1.4936475
sp|P07900-2|HS90A_HUMAN	-0.3257885	4.833581
sp|Q9H4A4|AMPB_HUMAN	-0.3236752	3.707581
sp|Q13409-3|DC1I2_HUMAN	-0.3231163	1.5054473
sp|P35222|CTNB1_HUMAN	-0.3227863	3.4979637
sp|P16219|ACADS_HUMAN	-0.3222065	2.6576471
sp|P05165-2|PCCA_HUMAN	-0.3215561	5.229891
sp|Q9Y230|RUVB2_HUMAN	-0.3205471	2.4761837
sp|P00390-2|GSHR_HUMAN	-0.320221	4.051346

sp|Q96QK1|VPS35_HUMAN	-0.3186073	2.400925
sp|P00367|DHE3_HUMAN	-0.3184815	3.1516862
sp|Q5JPE7-2|NOMO2_HUMAN	-0.3184605	1.6960104
sp|P55084|ECHB_HUMAN	-0.31637	3.1223214
sp|Q9NQW7-3|XPP1_HUMAN	-0.3160515	1.9413493
sp|O95782-2|AP2A1_HUMAN	-0.3154373	2.3143692
sp|P54577|SYYC_HUMAN	-0.3144932	1.6960104
sp|Q8TDL5|BPIB1_HUMAN	-0.3136826	2.5461748
sp|P12956|XRCC6_HUMAN	-0.3135071	5.2716713
sp|P13010|XRCC5_HUMAN	-0.3134575	4.553372
sp|P22695|QCR2_HUMAN	-0.3133469	3.417183
sp|O43747-2|AP1G1_HUMAN	-0.3122635	2.1818786
sp|P21953|ODBB_HUMAN	-0.311758	1.6960104
sp|P15144|AMPN_HUMAN	-0.3114338	1.4050349
sp|P14550|AK1A1_HUMAN	-0.3114262	3.7734513
sp|P27797|CALR_HUMAN	-0.3111057	5.869831
sp|P49354-2|FNTA_HUMAN	-0.3105125	2.1818786
sp|Q7L1Q6-2|BZW1_HUMAN	-0.3100357	1.4104178
sp|P30153|2AAA_HUMAN	-0.3092766	4.5929456
sp|Q16851|UGPA_HUMAN	-0.3082333	4.984084
sp|P28062|PSB8_HUMAN	-0.3081322	2.1818786
sp|Q15293|RCN1_HUMAN	-0.3056603	2.6576471
sp|Q14974|IMB1_HUMAN	-0.3055801	4.372737
sp|O75131|CPNE3_HUMAN	-0.3046284	2.4846697
sp|P05534|1A24_HUMAN	-0.3041077	1.6960104
sp|P43034|LIS1_HUMAN	-0.3034611	2.5644996
sp|Q9UHB6-4|LIMA1_HUMAN	-0.3032246	1.9647322
sp|P62820|RAB1A_HUMAN	-0.3024616	2.1818786
sp|O96009|NAPSA_HUMAN	-0.3024178	1.6997428
sp|P05783|K1C18_HUMAN	-0.3023891	6.965214
sp|P16152|CBR1_HUMAN	-0.3019199	2.6182911
sp|P40763-3|STAT3_HUMAN	-0.301899	1.9647322
sp|P07988|PSPB_HUMAN	-0.3015461	1.7703108
sp|O75436|VP26A_HUMAN	-0.3015232	2.151766
sp|P62136|PP1A_HUMAN	-0.300972	1.6960104
sp|O75348|VATG1_HUMAN	-0.2997055	1.6960104
sp|O43175|SERA_HUMAN	-0.299324	4.033051
sp|P17931|LEG3_HUMAN	-0.2991943	2.6576471
sp|Q14103-3|HNRPD_HUMAN	-0.2986527	2.997126
sp|P48444|COPD_HUMAN	-0.2984905	6.9433603
sp|Q6P2Q9|PRP8_HUMAN	-0.2984085	1.6960104
sp|P04075|ALDOA_HUMAN	-0.2974892	7.232633
sp|P14543-2|NID1_HUMAN	-0.2974129	4.0566006
sp|Q9NQC3|RTN4_HUMAN	-0.2972984	1.9647322
sp|O15144|ARPC2_HUMAN	-0.2972755	2.1800864

sp|P55263|ADK_HUMAN	-0.2967472	1.9647322
sp|Q96CW1-2|AP2M1_HUMAN	-0.2952614	2.1818786
sp|P36957|ODO2_HUMAN	-0.295187	3.412591
sp|Q96AE4-2|FUBP1_HUMAN	-0.293808	1.6853416
sp|Q9NP72|RAB18_HUMAN	-0.292984	2.1818786
sp|O95479|G6PE_HUMAN	-0.2906647	1.3815327
sp|P23634-8|AT2B4_HUMAN	-0.2895489	1.6832623
sp|Q9Y3F4-2|STRAP_HUMAN	-0.2891102	1.4104178
sp|P49593-2|PPM1F_HUMAN	-0.2882328	1.9647322
sp|Q92945|FUBP2_HUMAN	-0.2872543	2.6134143
sp|Q07157|ZO1_HUMAN	-0.2868862	3.887875
sp|Q9BXS5-2|AP1M1_HUMAN	-0.2867699	2.1818786
sp|Q92688-2|AN32B_HUMAN	-0.2864399	1.6960104
sp|P13284|GILT_HUMAN	-0.2857323	1.6960104
sp|P13489|RINI_HUMAN	-0.2853355	5.6671495
sp|P34897-2|GLYM_HUMAN	-0.284811	1.9997257
sp|P18669|PGAM1_HUMAN	-0.2840881	4.66111
sp|P46926|GNPI1_HUMAN	-0.2835255	1.9647322
sp|P50454|SERPH_HUMAN	-0.2816353	3.622396
sp|P05026-2|AT1B1_HUMAN	-0.2815971	2.4829872
sp|O14773|TPP1_HUMAN	-0.2813759	1.9647322
sp|P13797|PLST_HUMAN	-0.2812138	2.9110386
sp|Q13423|NNTM_HUMAN	-0.2809906	1.6295799
sp|Q9H223|EHD4_HUMAN	-0.2804833	3.6746628
sp|P09110|THIK_HUMAN	-0.2800827	1.9647322
sp|P30084|ECHM_HUMAN	-0.2796726	1.560883
sp|Q14165|MLEC_HUMAN	-0.2794304	1.6960104
sp|P31939|PUR9_HUMAN	-0.279417	2.2535503
sp|O14936-2|CSKP_HUMAN	-0.2782097	1.9951487
sp|P62316-2|SMD2_HUMAN	-0.2767181	1.6960104
sp|P31150|GDIA_HUMAN	-0.2745285	2.9903855
sp|P09917|LOX5_HUMAN	-0.2743282	1.8444856
sp|Q16658|FSCN1_HUMAN	-0.2742176	2.7936864
sp|Q9NR45|SIAS_HUMAN	-0.2739391	1.9972724
sp|Q16531|DDB1_HUMAN	-0.2727757	3.5570853
sp|P62140|PP1B_HUMAN	-0.2725411	1.4104178
sp|O00170|AIP_HUMAN	-0.2719269	1.9647322
sp|P62993|GRB2_HUMAN	-0.2710953	1.9413493
sp|Q9UNF0-2|PACN2_HUMAN	-0.271019	2.4829872
sp|P27348|1433T_HUMAN	-0.2703285	1.9153107
sp|P06744|G6PI_HUMAN	-0.2676029	6.054049
sp|O43242|PSMD3_HUMAN	-0.2656918	2.112052
sp|Q16881-2|TRXR1_HUMAN	-0.2653294	2.2731557
sp|Q13162|PRDX4_HUMAN	-0.2644463	1.3080103
sp|Q9Y277-2|VDAC3_HUMAN	-0.2639427	2.5461748

sp|P49419-2|AL7A1_HUMAN	-0.2639351	3.8491135
sp|P14923|PLAK_HUMAN	-0.2637138	1.6604291
sp|Q9ULA0|DNPEP_HUMAN	-0.2631054	4.4923797
sp|Q96G03|PGM2_HUMAN	-0.2626438	2.6182911
sp|Q8WWI1-5|LMO7_HUMAN	-0.2621384	2.3787699
sp|P26196|DDX6_HUMAN	-0.2618351	1.4104178
sp|Q12906-4|ILF3_HUMAN	-0.2617378	5.178646
sp|P50552|VASP_HUMAN	-0.2612362	1.6092666
sp|Q8IZ83-3|A16A1_HUMAN	-0.2594872	2.2958338
sp|O14745|NHRF1_HUMAN	-0.2586975	1.6960104
sp|P10809|CH60_HUMAN	-0.2576599	3.9826555
sp|Q15717-2|ELAV1_HUMAN	-0.2561588	1.4104178
sp|P20618|PSB1_HUMAN	-0.2561092	3.5908275
sp|P23526|SAHH_HUMAN	-0.2547798	4.798874
sp|P42704|LPPRC_HUMAN	-0.2547379	1.4278674
sp|Q9NPH2|INO1_HUMAN	-0.2535782	2.6997027
sp|P52597|HNRPF_HUMAN	-0.2532616	1.3080103
sp|P37802|TAGL2_HUMAN	-0.251215	2.7847733
sp|P25398|RS12_HUMAN	-0.250804	2.6134143
sp|P14780|MMP9_HUMAN	-0.2504082	1.7165122
sp|P55072|TERA_HUMAN	-0.2496452	9.507561
sp|Q9BRA2|TXD17_HUMAN	-0.2489719	1.3815327
sp|Q9NUV9|GIMA4_HUMAN	-0.2484646	1.6092666
sp|P05198|IF2A_HUMAN	-0.2475243	2.1818786
sp|P20340-2|RAB6A_HUMAN	-0.2469273	3.417183
sp|Q9BVK6|TMED9_HUMAN	-0.2465153	1.6960104
sp|Q9BSJ8-2|ESYT1_HUMAN	-0.2455711	5.074809
sp|O15247|CLIC2_HUMAN	-0.2445698	1.7165122
sp|Q9UIJ7|KAD3_HUMAN	-0.2437096	1.7165122
sp|P25705|ATPA_HUMAN	-0.2429581	1.6604291
sp|P11215-2|ITAM_HUMAN	-0.2420521	2.7472508
sp|P28065-2|PSB9_HUMAN	-0.241539	1.9951487
sp|P60174|TPIS_HUMAN	-0.2414398	4.28272
sp|O14579|COPE_HUMAN	-0.2402325	2.3143692
sp|Q9BUT1|BDH2_HUMAN	-0.2396145	1.9190748
sp|Q9NZA1-2|CLIC5_HUMAN	-0.2394066	1.6832623
sp|O60749|SNX2_HUMAN	-0.2393398	3.9658737
sp|P06737-2|PYGL_HUMAN	-0.238163	3.455751
sp|P49368|TCPG_HUMAN	-0.2379723	4.6680183
sp|P35232|PHB_HUMAN	-0.2363358	2.8132849
sp|P37837|TALDO_HUMAN	-0.2341328	5.2000947
sp|Q7KZF4|SND1_HUMAN	-0.2336559	2.2613342
sp|P29218|IMPA1_HUMAN	-0.2331219	1.7703108
sp|P09758|TACD2_HUMAN	-0.2321911	2.1818786
sp|P14868|SYDC_HUMAN	-0.2313461	3.9138026

sp|O94760|DDAH1_HUMAN	-0.2306995	2.1818786
sp|P39656-3|OST48_HUMAN	-0.2303619	3.1629772
sp|P47756-2|CAPZB_HUMAN	-0.2299652	2.549662
sp|O75369-2|FLNB_HUMAN	-0.2288151	10.731803
sp|P30837|AL1B1_HUMAN	-0.2275314	2.3143692
sp|Q01130-2|SRSF2_HUMAN	-0.2274513	1.4104178
sp|P20674|COX5A_HUMAN	-0.2274303	1.6960104
sp|P36776-3|LONM_HUMAN	-0.2269688	1.4278674
sp|P24821-4|TENA_HUMAN	-0.2268753	1.5071542
sp|P08133|ANXA6_HUMAN	-0.226593	8.74701
sp|P07814|SYEP_HUMAN	-0.2245312	1.4351699
sp|P09874|PARP1_HUMAN	-0.2241554	1.827201
sp|P31930|QCR1_HUMAN	-0.2236977	1.7647069
sp|O15511|ARPC5_HUMAN	-0.2235909	1.6604291
sp|P08729|K2C7_HUMAN	-0.2233391	11.867092
sp|Q99729-3|ROAA_HUMAN	-0.2215405	2.2419565
sp|P26447|S10A4_HUMAN	-0.2210503	2.151766
sp|P50990|TCPQ_HUMAN	-0.2206936	5.627495
sp|P25786-2|PSA1_HUMAN	-0.2204113	3.9685462
sp|P34932|HSP74_HUMAN	-0.22015	3.0865371
sp|Q15366-2|PCBP2_HUMAN	-0.2197495	1.6960104
sp|P28331-2|NDUS1_HUMAN	-0.2193537	2.151766
sp|P61978-3|HNRPK_HUMAN	-0.218544	4.656231
sp|O75947-2|ATP5H_HUMAN	-0.2183609	2.432837
sp|Q14204|DYHC1_HUMAN	-0.2180748	1.709563
sp|P05455|LA_HUMAN	-0.2174816	1.9190748
sp|Q13263|TIF1B_HUMAN	-0.215786	1.3895186
sp|P49588-2|SYAC_HUMAN	-0.2154465	2.9661083
sp|Q08257|QOR_HUMAN	-0.2134972	1.4832523
sp|O00567|NOP56_HUMAN	-0.2133427	1.3567923
sp|P61163|ACTZ_HUMAN	-0.2115288	1.9647322
sp|O95831-3|AIFM1_HUMAN	-0.2111797	3.0495367
sp|P49591|SYSC_HUMAN	-0.2076263	1.4289604
sp|P29692-2|EF1D_HUMAN	-0.2076149	1.6960104
sp|P13861|KAP2_HUMAN	-0.2068939	3.3572266
sp|P00403|COX2_HUMAN	-0.2067528	1.5646582
sp|P19338|NUCL_HUMAN	-0.2064343	3.903645
sp|P62241|RS8_HUMAN	-0.2060108	3.1266599
sp|Q9UL46|PSME2_HUMAN	-0.2038422	1.560883
sp|Q9P2B2|FPRP_HUMAN	-0.2028084	1.4050349
sp|P22234-2|PUR6_HUMAN	-0.2023468	2.151766
sp|P62081|RS7_HUMAN	-0.2021008	1.6960104
sp|Q15631|TSN_HUMAN	-0.2017441	1.3815327
sp|P13611|CSPG2_HUMAN	-0.2005577	3.5442007
sp|P00338|LDHA_HUMAN	-0.1999397	2.0895255

sp|O43491|E41L2_HUMAN	-0.1991234	1.9308543
sp|Q9UHG3|PCYOX_HUMAN	-0.1990414	3.5378873
sp|P53992|SC24C_HUMAN	-0.1988802	2.1818786
sp|P00488|F13A_HUMAN	-0.1977081	1.9828281
sp|P08670|VIME_HUMAN	-0.1974621	11.281799
sp|P35998|PRS7_HUMAN	-0.1970387	1.6853416
sp|Q16891-2|MIC60_HUMAN	-0.1960983	1.7451575
sp|P61081|UBC12_HUMAN	-0.1940651	2.6576471
sp|P52566|GDIR2_HUMAN	-0.1935501	2.432837
sp|Q99832|TCPH_HUMAN	-0.1935177	2.8438601
sp|O15173-2|PGRC2_HUMAN	-0.1902542	1.7590232
sp|P46976-2|GLYG_HUMAN	-0.1893539	1.560883
sp|Q15582|BGH3_HUMAN	-0.1891365	2.3051164
sp|Q9UKV3-5|ACINU_HUMAN	-0.1872253	1.9647322
sp|Q8NBS9|TXND5_HUMAN	-0.1863403	5.001318
sp|Q12905|ILF2_HUMAN	-0.1802921	2.2613342
sp|P62266|RS23_HUMAN	-0.1794853	1.560883
sp|P00387-3|NB5R3_HUMAN	-0.1791229	1.4278674
sp|P52565|GDIR1_HUMAN	-0.1788998	2.3388627
sp|P00352|AL1A1_HUMAN	-0.1787834	4.450073
sp|Q13308-6|PTK7_HUMAN	-0.1769295	1.9647322
sp|Q02790|FKBP4_HUMAN	-0.1759605	2.1818786
sp|Q15365|PCBP1_HUMAN	-0.1759148	1.4050349
sp|Q99460|PSMD1_HUMAN	-0.1753292	1.804513
sp|O43390-2|HNRPR_HUMAN	-0.1751957	1.8380218
sp|O75396|SC22B_HUMAN	-0.1735649	1.4050349
sp|P50570-2|DYN2_HUMAN	-0.1706619	1.6092666
sp|Q13228-4|SBP1_HUMAN	-0.1706162	4.0222406
sp|P25789|PSA4_HUMAN	-0.1694527	1.9308543
sp|Q99623|PHB2_HUMAN	-0.1688328	2.0265307
sp|P61981|1433G_HUMAN	-0.1680832	1.5121045
sp|Q6DD88|ATLA3_HUMAN	-0.1674004	1.3080103
sp|P55786|PSA_HUMAN	-0.1643906	3.201616
sp|P21281|VATB2_HUMAN	-0.1623888	1.9951487
sp|Q8N1G4|LRC47_HUMAN	-0.1622963	3.2221727
sp|P47755|CAZA2_HUMAN	-0.1613388	2.1663873
sp|P32969|RL9_HUMAN	-0.1606369	1.6960104
sp|Q06830|PRDX1_HUMAN	-0.1605682	3.6087239
sp|P50895|BCAM_HUMAN	-0.1600246	2.9456708
sp|P62277|RS13_HUMAN	-0.1587296	2.1818786
sp|Q16555|DPYL2_HUMAN	-0.1581001	2.8510797
sp|P52907|CAZA1_HUMAN	-0.1560116	1.7703108
sp|Q9UBE0|SAE1_HUMAN	-0.1556168	1.4104178
sp|Q9P2E9|RRBP1_HUMAN	-0.1555672	7.8947906
sp|P54920|SNAA_HUMAN	-0.1550922	2.4364867

sp|Q6YN16|HSDL2_HUMAN	-0.154582	1.6960104
sp|P09972|ALDOC_HUMAN	-0.1537094	1.9951487
sp|Q15599-2|NHRF2_HUMAN	-0.151001	1.6997428
sp|O00264|PGRC1_HUMAN	-0.1486282	1.6997428
sp|P62888|RL30_HUMAN	-0.1481209	1.4104178
sp|Q14203-3|DCTN1_HUMAN	-0.1480484	1.3080103
sp|P35221|CTNA1_HUMAN	-0.1476898	3.5868163
sp|Q13185|CBX3_HUMAN	-0.1471272	1.5646582
sp|Q9Y624|JAM1_HUMAN	-0.1455498	1.3635377
sp|P43121|MUC18_HUMAN	-0.1454296	1.8444856
sp|P49821-2|NDUV1_HUMAN	-0.1438141	2.1404836
sp|P19971|TYPH_HUMAN	-0.1420021	1.4342331
sp|O14818|PSA7_HUMAN	-0.1414108	1.9547566
sp|Q07065|CKAP4_HUMAN	-0.1396027	4.8765783
sp|Q06323|PSME1_HUMAN	-0.1387177	2.0727112
sp|P08758|ANXA5_HUMAN	-0.1386623	1.4431648
sp|P04844|RPN2_HUMAN	-0.1381187	1.4278674
sp|Q15124|PGM5_HUMAN	-0.1376114	1.5565699
sp|P11766|ADHX_HUMAN	-0.134491	1.3274423
sp|P09496-2|CLCA_HUMAN	-0.1316109	1.7590232
sp|Q9H2D6-2|TARA_HUMAN	-0.12957	1.515701
sp|P40227|TCPZ_HUMAN	-0.1294231	1.6545225
sp|P06576|ATPB_HUMAN	-0.1249275	3.5483983
sp|P16284-3|PECA1_HUMAN	-0.121769	2.0337436
sp|O15230|LAMA5_HUMAN	-0.1212273	2.0156884
sp|Q9UBG0|MRC2_HUMAN	-0.1091328	1.3184075
sp|P23141-2|EST1_HUMAN	-0.1078587	2.735238
sp|Q02818|NUCB1_HUMAN	-0.1036758	1.3437729
sp|P98160|PGBM_HUMAN	-0.1035366	2.6836658
sp|P18206-2|VINC_HUMAN	-0.1015472	5.9874315
sp|Q8IUX7|AEBP1_HUMAN	-0.0954533	1.827201
sp|Q14195-2|DPYL3_HUMAN	-0.0918236	1.9030465
sp|P02545|LMNA_HUMAN	-0.0901241	6.3223624
sp|P46939-2|UTRO_HUMAN	-0.0862579	1.3156533
sp|Q86UP2-4|KTN1_HUMAN	-0.080019	1.8444856
sp|Q03252|LMNB2_HUMAN	-0.0682697	1.9998069
sp|Q14767|LTBP2_HUMAN	-0.0661459	1.5117614
sp|P09497-2|CLCB_HUMAN	-0.0553894	1.3815327
sp|Q15019-2|SEPT2_HUMAN	-0.0534782	1.8831652
sp|Q05707|COEA1_HUMAN	0.067379	2.774158
sp|P04792|HSPB1_HUMAN	0.07087708	1.7659291
sp|P11047|LAMC1_HUMAN	0.0709095	1.858108
sp|Q07507|DERM_HUMAN	0.10429001	1.7703108
sp|P51911|CNN1_HUMAN	0.1061821	1.4068714
sp|Q7Z406|MYH14_HUMAN	0.12259293	3.381076

sp|P00751|CFAB_HUMAN	0.12550163	3.6288962
sp|Q16363-2|LAMA4_HUMAN	0.12610245	3.4914024
sp|P02671|FIBA_HUMAN	0.12709236	1.9274682
sp|P00450|CERU_HUMAN	0.12908554	3.2943964
sp|A6NMZ7|CO6A6_HUMAN	0.1383667	1.8350728
sp|Q8N2S1|LTBP4_HUMAN	0.14060402	2.5391243
sp|P05556|ITB1_HUMAN	0.15581131	6.041628
sp|P00492|HPRT_HUMAN	0.15593338	2.1818786
sp|Q14315-2|FLNC_HUMAN	0.15694046	10.35943
sp|P21291|CSRP1_HUMAN	0.15939903	3.0793355
sp|P02768|ALBU_HUMAN	0.16853333	1.3906018
sp|P06681-3|CO2_HUMAN	0.17321014	1.9951487
sp|P04217|A1BG_HUMAN	0.17682457	2.8793902
sp|P02511|CRYAB_HUMAN	0.18787003	1.6960104
sp|P01876|IGHA1_HUMAN	0.18862915	3.3283036
sp|P07357|CO8A_HUMAN	0.19548035	1.4278674
sp|Q6XQN6-2|PNCB_HUMAN	0.19822693	1.5071542
sp|P21266|GSTM3_HUMAN	0.20181656	2.044628
sp|P56199|ITA1_HUMAN	0.20426369	4.546434
sp|P01860|IGHG3_HUMAN	0.21148682	1.560883
sp|P27105|STOM_HUMAN	0.21913719	4.044672
sp|P02748|CO9_HUMAN	0.22149658	2.975305
sp|P60660-2|MYL6_HUMAN	0.2262268	3.671968
sp|P36955|PEDF_HUMAN	0.22843552	1.9951487
sp|P01031|CO5_HUMAN	0.23292351	5.134014
sp|P02452|CO1A1_HUMAN	0.23876762	2.058973
sp|P98095-2|FBLN2_HUMAN	0.24297333	3.455751
sp|P04040|CATA_HUMAN	0.24402046	6.1563954
sp|P07358|CO8B_HUMAN	0.24963188	2.1818786
sp|Q12805-2|FBLN3_HUMAN	0.25226593	4.748085
sp|Q9Y6C2|EMIL1_HUMAN	0.2540226	4.192454
sp|P07951|TPM2_HUMAN	0.25456047	1.827201
sp|P00734|THRB_HUMAN	0.26342964	4.4162235
sp|Q9Y3D6|FIS1_HUMAN	0.26423264	1.6960104
sp|P00441|SODC_HUMAN	0.27708817	2.3143692
sp|P0DOX5|IGG1_HUMAN	0.27853775	3.9658737
sp|P05156|CFAI_HUMAN	0.27938843	4.051346
sp|P21810|PGS1_HUMAN	0.2801361	3.261748
sp|P08572|CO4A2_HUMAN	0.28465462	2.7305796
sp|Q01995|TAGL_HUMAN	0.2856617	5.742677
sp|P08473|NEP_HUMAN	0.28759193	1.6960104
sp|P36269-3|GGT5_HUMAN	0.29522133	1.6092666
sp|P01009|A1AT_HUMAN	0.29598618	7.023117
sp|Q9UMS6-2|SYNP2_HUMAN	0.29974747	2.2323174
sp|A1L4H1|SRCRL_HUMAN	0.30083466	1.6960104

sp|Q0ZGT2-4|NEXN_HUMAN	0.302763	3.412591
sp|P03952|KLKB1_HUMAN	0.30330658	2.1818786
sp|Q07075|AMPE_HUMAN	0.3085518	1.7590232
sp|P09493-9|TPM1_HUMAN	0.31389618	1.6960104
sp|P16157-21|ANK1_HUMAN	0.33254623	3.622396
sp|P24844|MYL9_HUMAN	0.34386444	1.6960104
sp|P02790|HEMO_HUMAN	0.35511017	5.303003
sp|P25311|ZA2G_HUMAN	0.35788536	4.051346
sp|P00325|ADH1B_HUMAN	0.363163	3.5190198
sp|P19827|ITIH1_HUMAN	0.36735916	3.0253785
sp|P17661|DESM_HUMAN	0.38835144	14.699317
sp|P30041|PRDX6_HUMAN	0.38887405	4.5314264
sp|P16671-4|CD36_HUMAN	0.39097214	2.1818786
sp|P09493-8|TPM1_HUMAN	0.39201736	3.5908275
sp|P02774-3|VTDB_HUMAN	0.39401817	7.9121003
sp|P02766|TTHY_HUMAN	0.3955307	1.9647322
sp|P00491|PNPH_HUMAN	0.3961029	3.2450993
sp|P08603|CFAH_HUMAN	0.39652252	12.649239
sp|Q9UBX5|FBLN5_HUMAN	0.41771698	3.412591
sp|P35527|K1C9_HUMAN	0.42129326	5.6770253
sp|P00747|PLMN_HUMAN	0.42140198	6.1581025
sp|P35580-3|MYH10_HUMAN	0.42673302	14.282492
sp|P15090|FABP4_HUMAN	0.4310379	3.1266599
sp|P51888|PRELP_HUMAN	0.43401718	5.869831
sp|P30043|BLVRB_HUMAN	0.4383793	1.7590232
sp|P11678|PERE_HUMAN	0.4480133	1.4104178
sp|P04003|C4BPA_HUMAN	0.44812202	5.7968407
sp|P01019|ANGT_HUMAN	0.45259285	1.9647322
sp|P27169|PON1_HUMAN	0.46069717	1.6960104
sp|P35749|MYH11_HUMAN	0.46853828	15.051499
sp|P02649|APOE_HUMAN	0.4686203	4.5016932
sp|P04259|K2C6B_HUMAN	0.48851204	1.9647322
sp|P08123|CO1A2_HUMAN	0.4899025	1.8444856
sp|P02760|AMBP_HUMAN	0.49378586	3.5908275
sp|P23456|Trypsin	0.49433327	3.5908275
sp|P11277-2|SPTB1_HUMAN	0.5000839	4.95426
sp|P19823|ITIH2_HUMAN	0.52166176	5.417897
sp|P21980|TGM2_HUMAN	0.5339699	9.789072
sp|P01871-2|IGHM_HUMAN	0.5422306	2.1818786
sp|P43652|AFAM_HUMAN	0.545166	4.509013
sp|P05546|HEP2_HUMAN	0.56792927	2.6576471
sp|P02749|APOH_HUMAN	0.57450867	2.2385595
sp|P04114|APOB_HUMAN	0.57496643	10.942365
sp|P01008|ANT3_HUMAN	0.5877552	8.010044
sp|P13716-2|HEM2_HUMAN	0.59879494	3.5908275

sp|P08294|SODE_HUMAN	0.6058388	2.8384566
sp|O14558|HSPB6_HUMAN	0.6175957	1.9951487
sp|P29622|KAIN_HUMAN	0.61922836	2.6576471
sp|P02743|SAMP_HUMAN	0.6318455	4.509013
sp|P02787|TRFE_HUMAN	0.65704346	14.724141
sp|P51884|LUM_HUMAN	0.6701145	8.112776
sp|P01042-2|KNG1_HUMAN	0.67518806	7.2183566
sp|P04264|K2C1_HUMAN	0.70025826	12.045783
sp|P35908|K22E_HUMAN	0.717186	7.665951
sp|P07585|PGS2_HUMAN	0.7200966	5.338249
sp|P13645|K1C10_HUMAN	0.7252445	10.200565
sp|Q5TDH0-3|DDI2_HUMAN	0.7323704	1.6960104
sp|P02730|B3AT_HUMAN	0.73609734	6.7698927
sp|P10909-5|CLUS_HUMAN	0.7464943	4.964395
sp|P02549-2|SPTA1_HUMAN	0.8312626	9.058883
sp|P07451|CAH3_HUMAN	0.8336315	3.1266599
sp|P06727|APOA4_HUMAN	0.8424301	5.98738
sp|P07738|PMGE_HUMAN	0.90335464	2.1818786
sp|P20774|MIME_HUMAN	1.0496197	4.051346
sp|P02652-2|APOA2_HUMAN	1.1846085	3.1266599
sp|P00918|CAH2_HUMAN	1.2519054	4.509013
sp|P02647|APOA1_HUMAN	1.274868	13.420564
sp|P32119|PRDX2_HUMAN	1.4412308	6.7698927
sp|P02042|HBD_HUMAN	1.6058521	4.051346
sp|P00915|CAH1_HUMAN	1.721487	7.665951
sp|P68871|HBB_HUMAN	1.8555679	4.051346
sp|P69905|HBA_HUMAN	2.1029243	4.051346
sp|P04229|2B11_HUMAN	-2.2219257	0.656254
sp|P08263|GSTA1_HUMAN	-2.1355228	0.656254
sp|P08246|ELNE_HUMAN	-1.3340645	1.1932944
sp|P13760|2B14_HUMAN	-1.232172	1.2773042
sp|P12236|ADT3_HUMAN	-1.0901146	1.1932944
sp|P13761|2B17_HUMAN	-1.0802727	0
sp|Q86TX2|ACOT1_HUMAN	-1.0426483	0.656254
sp|P02741|CRP_HUMAN	-1.0062256	1.1932944
sp|O00757|F16P2_HUMAN	-1.0049801	1.1932944
sp|P68371|TBB4B_HUMAN	-0.9974442	1.1932944
sp|P51153|RAB13_HUMAN	-0.9821014	0.656254
sp|P54108-2|CRIS3_HUMAN	-0.9708176	0.656254
sp|Q9UBR2|CATZ_HUMAN	-0.9673367	1.1932944
sp|Q9HD89|RETN_HUMAN	-0.9641037	1.1932944
sp|Q13938-4|CAYP1_HUMAN	-0.9013062	0.656254
sp|Q9H2U2-3|IPYR2_HUMAN	-0.8743992	0.656254
sp|Q9BWS9-3|CHID1_HUMAN	-0.854763	0.656254
sp|P15559-2|NQO1_HUMAN	-0.8539333	1.1932944

sp|P38159|RBMX_HUMAN	-0.8475208	0.656254
sp|P11586|C1TC_HUMAN	-0.8423853	0.7061832
sp|A6NMY6|AXA2L_HUMAN	-0.8380165	0.656254
sp|P06396-2|GELS_HUMAN	-0.8181171	0.656254
sp|Q29963|1C06_HUMAN	-0.7859402	0.656254
sp|Q12846|STX4_HUMAN	-0.7844124	0.656254
sp|P11387|TOP1_HUMAN	-0.7710934	1.1932944
sp|Q96IJ6-2|GMPPA_HUMAN	-0.7543345	1.1932944
sp|P51606-2|RENBP_HUMAN	-0.7510128	1.1932944
sp|O95197-2|RTN3_HUMAN	-0.749445	1.1932944
sp|Q15185-3|TEBP_HUMAN	-0.7354126	1.1932944
sp|P63167|DYL1_HUMAN	-0.711937	0.656254
sp|P0CG39|POTEJ_HUMAN	-0.7092247	0.656254
sp|P62834|RAP1A_HUMAN	-0.705574	0.656254
sp|Q9NUB1-2|ACS2L_HUMAN	-0.698225	1.1932944
sp|O43795-2|MYO1B_HUMAN	-0.6936245	0.7827403
sp|A0A0B4J1X8|HV343_HUMAI	-0.6877708	0.656254
sp|P28330|ACADL_HUMAN	-0.6522293	1.1932944
sp|Q03135-2|CAV1_HUMAN	-0.6492806	0.656254
sp|P27635|RL10_HUMAN	-0.6467896	0.656254
sp|Q16822|PCKGM_HUMAN	-0.6280899	1.1932944
sp|Q8NBQ5|DHB11_HUMAN	-0.6153679	0.7827403
sp|O60701-2|UGDH_HUMAN	-0.6132889	0.656254
sp|P63261|ACTG_HUMAN	-0.6130772	0.656254
sp|Q562R1|ACTBL_HUMAN	-0.6006661	0.6070219
sp|P60709|ACTB_HUMAN	-0.5986881	0.656254
sp|Q13884|SNTB1_HUMAN	-0.5885353	0.7827403
sp|P08962-2|CD63_HUMAN	-0.587883	1.1932944
sp|Q15056-2|IF4H_HUMAN	-0.5872421	1.1932944
sp|P20702|ITAX_HUMAN	-0.5785523	0.656254
sp|Q8TD06|AGR3_HUMAN	-0.5775299	1.1932944
sp|O75533|SF3B1_HUMAN	-0.5772476	0.7827403
sp|Q96FV2-2|SCRN2_HUMAN	-0.5754013	1.1932944
sp|O95394-3|AGM1_HUMAN	-0.5680809	1.1932944
sp|P04839|CY24B_HUMAN	-0.5673189	1.1932944
sp|Q9Y394-2|DHRS7_HUMAN	-0.5621166	1.2095301
sp|P10619-2|PPGB_HUMAN	-0.5587711	1.1932944
sp|Q9P2J5-2|SYLC_HUMAN	-0.5575085	0.7827403
sp|Q01518|CAP1_HUMAN	-0.5542402	0.656254
sp|P24158|PRTN3_HUMAN	-0.5539551	1.1505735
sp|P52788-2|SPSY_HUMAN	-0.5516262	1.1932944
sp|Q14956-2|GPNMB_HUMAN	-0.5465889	1.1932944
sp|P07948-2|LYN_HUMAN	-0.5422115	1.1932944
sp|P62244|RS15A_HUMAN	-0.5230141	1.1932944
sp|P30419-2|NMT1_HUMAN	-0.5208435	0.656254

sp|P18085|ARF4_HUMAN	-0.5197945	0.656254
sp|Q16539-2|MK14_HUMAN	-0.5161457	1.1932944
sp|P15153|RAC2_HUMAN	-0.5131683	0.656254
sp|Q13283|G3BP1_HUMAN	-0.5041752	0.6070219
sp|P63218|GBG5_HUMAN	-0.5029736	1.1932944
sp|Q8N684-3|CPSF7_HUMAN	-0.4998703	0.6070219
sp|Q7L576|CYFP1_HUMAN	-0.4980688	1.1932944
sp|P45954-2|ACDSB_HUMAN	-0.4918842	1.1932944
sp|P78406|RAE1L_HUMAN	-0.4898758	1.1932944
sp|P15170-2|ERF3A_HUMAN	-0.4879723	1.1932944
sp|P27824-2|CALX_HUMAN	-0.4847984	1.0475321
sp|O75923-11|DYSF_HUMAN	-0.4806671	0.35795313
sp|Q9UHN6-2|CEIP2_HUMAN	-0.4787102	0.7827403
sp|P24534|EF1B_HUMAN	-0.4741688	1.1932944
sp|Q9NVJ2|ARL8B_HUMAN	-0.473917	1.1932944
sp|P08134|RHOC_HUMAN	-0.4707813	0.7827403
sp|P37235|HPCL1_HUMAN	-0.4697075	0.656254
sp|P61586|RHOA_HUMAN	-0.466507	1.1932944
sp|Q9Y262-2|EIF3L_HUMAN	-0.4656086	1.0370445
sp|P35080-2|PROF2_HUMAN	-0.459877	0.656254
sp|P51991-2|ROA3_HUMAN	-0.4571457	0.656254
sp|P19404|NDUV2_HUMAN	-0.4501343	1.1932944
sp|P50579-2|MAP2_HUMAN	-0.4476681	1.1932944
sp|Q9UBS4|DJB11_HUMAN	-0.4453831	1.1932944
sp|Q8NHP8|PLBL2_HUMAN	-0.4417439	1.1932944
sp|Q99538-2|LGMN_HUMAN	-0.4414682	0.7827403
sp|P30566|PUR8_HUMAN	-0.4413261	0.656254
sp|Q9HD45|TM9S3_HUMAN	-0.4389744	1.1932944
sp|P12694|ODBA_HUMAN	-0.4387684	0.7061832
sp|P62854|RS26_HUMAN	-0.4378357	1.1932944
sp|P61313|RL15_HUMAN	-0.4375725	1.1932944
sp|P04222|1C03_HUMAN	-0.4375649	0.656254
sp|P12235|ADT1_HUMAN	-0.4358978	0.656254
sp|Q14697|GANAB_HUMAN	-0.4299164	1.1932944
sp|P01111|RASN_HUMAN	-0.4293423	0.656254
sp|P30838|AL3A1_HUMAN	-0.4284649	1.1932944
sp|P28676|GRAN_HUMAN	-0.4268494	1.1932944
sp|Q71UM5|RS27L_HUMAN	-0.426405	1.1932944
sp|Q14344|GNA13_HUMAN	-0.4231491	0.8983557
sp|Q9UQ80|PA2G4_HUMAN	-0.4225426	0.656254
sp|O76003|GLRX3_HUMAN	-0.4121933	0.656254
sp|P11717|MPRI_HUMAN	-0.4086895	1.1932944
sp|Q96JB5-4|CK5P3_HUMAN	-0.4079342	0.7061832
sp|Q8NC51-3|PAIRB_HUMAN	-0.4077358	1.1932944
sp|Q07812-7|BAX_HUMAN	-0.4071026	1.1932944

sp|P06239-3|LCK_HUMAN	-0.4054699	0.656254
sp|O00712-4|NFIB_HUMAN	-0.404953	0.656254
sp|P62745|RHOB_HUMAN	-0.402195	0.656254
sp|P09417-2|DHPR_HUMAN	-0.4013786	0.45033538
sp|P06865|HEXA_HUMAN	-0.3977661	1.2773042
sp|P51553-2|IDH3G_HUMAN	-0.3975678	1.1932944
sp|P46977|STT3A_HUMAN	-0.3947601	1.1932944
sp|Q14152|EIF3A_HUMAN	-0.3932705	0.35795313
sp|O75828|CBR3_HUMAN	-0.3920307	1.1932944
sp|P52895|AK1C2_HUMAN	-0.3891993	0.45033538
sp|Q9ULZ3-2|ASC_HUMAN	-0.3877678	0
sp|P20160|CAP7_HUMAN	-0.3840103	0.91601294
sp|P26368-2|U2AF2_HUMAN	-0.3839188	1.1932944
sp|P31946-2|1433B_HUMAN	-0.3810577	0.656254
sp|P36551|HEM6_HUMAN	-0.3786755	0.19149946
sp|Q13310-2|PABP4_HUMAN	-0.3765907	0.656254
sp|P61160|ARP2_HUMAN	-0.374876	1.1932944
sp|Q9NPJ3|ACO13_HUMAN	-0.3742695	1.1932944
sp|O94905|ERLN2_HUMAN	-0.3707714	1.1932944
sp|Q8TBC4-2|UBA3_HUMAN	-0.3698387	0.7827403
sp|Q32P44|EMAL3_HUMAN	-0.3693543	1.2773042
sp|Q9UH99-3|SUN2_HUMAN	-0.3683872	1.0485198
sp|P62714|PP2AB_HUMAN	-0.3637085	0.656254
sp|Q7L2H7|EIF3M_HUMAN	-0.3622532	0.656254
sp|Q8NDH3|PEPL1_HUMAN	-0.3610382	1.2095301
sp|P61916-2|NPC2_HUMAN	-0.3596306	1.1932944
sp|P42226|STAT6_HUMAN	-0.3578796	0.656254
sp|O00186|STXB3_HUMAN	-0.3552275	0.45033538
sp|P63151-2|2ABA_HUMAN	-0.3543987	1.1932944
sp|Q06828|FMOD_HUMAN	-0.3537445	1.1932944
sp|P61353|RL27_HUMAN	-0.3535042	1.1932944
sp|O95716|RAB3D_HUMAN	-0.3523026	0.656254
sp|P62995-3|TRA2B_HUMAN	-0.3521347	1.1932944
sp|Q9NPY3|C1QR1_HUMAN	-0.352066	1.1932944
sp|Q16134-3|ETFD_HUMAN	-0.3515568	1.1932944
sp|O43252|PAPS1_HUMAN	-0.3508263	1.0301651
sp|Q5SSJ5-2|HP1B3_HUMAN	-0.3506928	1.0485198
sp|P31689-2|DNJA1_HUMAN	-0.3500481	1.1932944
sp|Q9BQE3|TBA1C_HUMAN	-0.3491592	0.656254
sp|P22059|OSBP1_HUMAN	-0.3484116	1.1932944
sp|P11233|RALA_HUMAN	-0.3469429	0.8983557
sp|Q03113|GNA12_HUMAN	-0.3462658	0.656254
sp|P52789|HXK2_HUMAN	-0.3460026	0.656254
sp|Q13200|PSMD2_HUMAN	-0.3452511	0.91601294
sp|O60271-4|JIP4_HUMAN	-0.3450584	0.7061832

sp|O43684-2|BUB3_HUMAN	-0.3435268	1.1932944
sp|P61956-2|SUMO2_HUMAN	-0.343338	0.656254
sp|Q9Y305-4|ACOT9_HUMAN	-0.3432121	1.1932944
sp|Q15691|MARE1_HUMAN	-0.3426037	0.95332193
sp|P63220|RS21_HUMAN	-0.3420677	1.1932944
sp|Q92696|PGTA_HUMAN	-0.3418961	1.1791906
sp|Q13813-3|SPTN1_HUMAN	-0.3415871	0.19149946
sp|P20292|AL5AP_HUMAN	-0.340538	0.35795313
sp|P49257|LMAN1_HUMAN	-0.3361244	0.96371543
sp|P60866-2|RS20_HUMAN	-0.3336277	1.1932944
sp|Q9H2G2-2|SLK_HUMAN	-0.3330212	0.7827403
sp|P26885|FKBP2_HUMAN	-0.3321838	1.1932944
sp|Q6P4A8|PLBL1_HUMAN	-0.3321114	0.7588735
sp|P07437|TBB5_HUMAN	-0.3306255	0.6298893
sp|Q96C23|GALM_HUMAN	-0.3300076	1.1505735
sp|P46459|NSF_HUMAN	-0.3271275	1.2095301
sp|P04062-2|GLCM_HUMAN	-0.3267632	1.1932944
sp|O00534|VMA5A_HUMAN	-0.3239784	1.0634323
sp|P31153|METK2_HUMAN	-0.3218136	0.656254
sp|O60784-3|TOM1_HUMAN	-0.3205376	0.656254
sp|P53041|PPP5_HUMAN	-0.3198147	0.7061832
sp|P36871|PGM1_HUMAN	-0.3194313	1.1932944
sp|A5A3E0|POTEF_HUMAN	-0.3193693	0
sp|Q8TD19|NEK9_HUMAN	-0.3185234	1.1932944
sp|P31946|1433B_HUMAN	-0.3176098	0.656254
sp|Q01813-2|PFKAP_HUMAN	-0.3172779	0.90036625
sp|O75323|NIPS2_HUMAN	-0.314867	1.1932944
sp|P49458|SRP09_HUMAN	-0.3116608	1.1932944
sp|O14744|ANM5_HUMAN	-0.3102551	1.1932944
sp|P08631-2|HCK_HUMAN	-0.3070431	0.656254
sp|Q9NZ01|TECR_HUMAN	-0.3069658	1.0485198
sp|P50452|SPB8_HUMAN	-0.3061066	1.1932944
sp|O95340-2|PAPS2_HUMAN	-0.3044529	1.1276597
sp|Q13435|SF3B2_HUMAN	-0.3043251	1.1932944
sp|P55265-4|DSRAD_HUMAN	-0.3041019	0.7061832
sp|P57088|TMM33_HUMAN	-0.3038807	1.1932944
sp|Q7Z4I7-3|LIMS2_HUMAN	-0.3033857	1.1932944
sp|P33151|CADH5_HUMAN	-0.2989311	1.1932944
sp|P48047|ATPO_HUMAN	-0.2967587	0.8235348
sp|Q9NYF8-2|BCLF1_HUMAN	-0.2948875	0.6070219
sp|Q15149-3|PLEC_HUMAN	-0.2945995	0.656254
sp|P09543-2|CN37_HUMAN	-0.2940807	1.1791906
sp|P24557-2|THAS_HUMAN	-0.2901859	1.1932944
sp|P36542-2|ATPG_HUMAN	-0.2888966	0.656254
sp|O14828-2|SCAM3_HUMAN	-0.2881165	0.5204253

sp|Q03154-4|ACY1_HUMAN	-0.2877178	0.91601294
sp|Q16836-3|HCDH_HUMAN	-0.2875843	1.1505735
sp|Q10567-2|AP1B1_HUMAN	-0.2871323	0.7061832
sp|O43615|TIM44_HUMAN	-0.2865048	1.1932944
sp|P62701|RS4X_HUMAN	-0.2864418	0.6298893
sp|Q14011-2|CIRBP_HUMAN	-0.2850285	0.5204253
sp|Q9UBQ5|EIF3K_HUMAN	-0.2834797	1.1932944
sp|A0A0C4DH31|HV118_HUMA	-0.2827778	0.656254
sp|Q93034|CUL5_HUMAN	-0.281332	0.7827403
sp|P61204|ARF3_HUMAN	-0.2800617	1.3006523
sp|P54819-2|KAD2_HUMAN	-0.2785626	0.35795313
sp|O00483|NDUA4_HUMAN	-0.2774773	0.7827403
sp|P48059-3|LIMS1_HUMAN	-0.2769623	0.656254
sp|P02769|ALBU_BOVIN	-0.2768211	0.0816602
sp|Q9H9B4|SFXN1_HUMAN	-0.2764607	1.1932944
sp|Q08209-5|PP2BA_HUMAN	-0.2752457	0
sp|O60832|DKC1_HUMAN	-0.2713032	1.1932944
sp|Q96AY3|FKB10_HUMAN	-0.2708492	0.6070219
sp|P55327-3|TPD52_HUMAN	-0.2700539	0.7827403
sp|Q06278|AOXA_HUMAN	-0.2694244	0.45033538
sp|Q15274|NADC_HUMAN	-0.2693882	0.5204253
sp|Q9BTE1|DCTN5_HUMAN	-0.2682991	1.1932944
sp|Q86W92-2|LIPB1_HUMAN	-0.2679157	1.1932944
sp|P04440|DPB1_HUMAN	-0.2678185	1.1932944
sp|P55809|SCOT1_HUMAN	-0.2675724	0.35795313
sp|P38606|VATA_HUMAN	-0.267026	0.77163273
sp|Q14498-2|RBM39_HUMAN	-0.2666817	0.48520416
sp|Q32MZ4-3|LRRF1_HUMAN	-0.2666359	0.45033538
sp|P21964-2|COMT_HUMAN	-0.2663155	1.0992733
sp|Q9UN86-2|G3BP2_HUMAN	-0.2636776	1.1932944
sp|P67775|PP2AA_HUMAN	-0.2629433	0.656254
sp|P62495-2|ERF1_HUMAN	-0.2626934	0.35795313
sp|Q9NX63|MIC19_HUMAN	-0.2623463	1.1932944
sp|P48509|CD151_HUMAN	-0.2620373	1.1932944
sp|P61601|NCALD_HUMAN	-0.2614155	0.656254
sp|P02794|FRIH_HUMAN	-0.2608013	1.1791906
sp|Q08170|SRSF4_HUMAN	-0.2583561	0.656254
sp|Q9P1F3|ABRAL_HUMAN	-0.257865	0.656254
sp|Q9NTX5-6|ECHD1_HUMAN	-0.2578182	1.214352
sp|P62424|RL7A_HUMAN	-0.257225	0.79861414
sp|P22894|MMP8_HUMAN	-0.2565117	0.19149946
sp|Q13885|TBB2A_HUMAN	-0.2559147	0.7827403
sp|O43865|SAHH2_HUMAN	-0.2551737	0.91601294
sp|Q16777|H2A2C_HUMAN	-0.2542992	0
sp|Q9UDY2-3|ZO2_HUMAN	-0.2542629	0.19149946

sp|P49903-2|SPS1_HUMAN	-0.2540073	1.1505735
sp|Q13363-2|CTBP1_HUMAN	-0.2539043	1.1932944
sp|O60831|PRAF2_HUMAN	-0.252573	0.91601294
sp|P29466-2|CASP1_HUMAN	-0.2521401	0.656254
sp|Q5TFE4|NT5D1_HUMAN	-0.251194	1.1932944
sp|P29992|GNA11_HUMAN	-0.2511463	1.1932944
sp|P41091|IF2G_HUMAN	-0.2494392	0.95332193
sp|Q13976|KGP1_HUMAN	-0.2494259	0
sp|P09601|HMOX1_HUMAN	-0.2460861	0.40256184
sp|P62942|FKB1A_HUMAN	-0.2459984	0.656254
sp|Q9Y371-2|SHLB1_HUMAN	-0.2413292	0
sp|Q8NBF2-2|NHLC2_HUMAN	-0.2408772	1.1932944
sp|Q96MM6|HS12B_HUMAN	-0.2402744	1.214352
sp|E9PAV3|NACAM_HUMAN	-0.2394524	1.1932944
sp|Q53T59|H1BP3_HUMAN	-0.2387676	1.1932944
sp|Q9Y5M8|SRPRB_HUMAN	-0.2384224	1.2941489
sp|O15127|SCAM2_HUMAN	-0.2381058	0.7061832
sp|Q9BRR6-2|ADPGK_HUMAN	-0.2375755	0.7827403
sp|P12830|CADH1_HUMAN	-0.2365055	0
sp|Q92769|HDAC2_HUMAN	-0.2343254	0.656254
sp|Q00765|REEP5_HUMAN	-0.2342224	1.1240381
sp|P61923|COPZ1_HUMAN	-0.2338963	0.19149946
sp|Q9HCN8|SDF2L_HUMAN	-0.2336655	1.1932944
sp|P28070|PSB4_HUMAN	-0.2332211	0.6167118
sp|Q6UW68|TM205_HUMAN	-0.2323494	1.1932944
sp|O60234|GMFG_HUMAN	-0.2321491	1.1505735
sp|Q9Y376|CAB39_HUMAN	-0.2310677	0.80804527
sp|P21912|SDHB_HUMAN	-0.2263317	1.1505735
sp|P84085|ARF5_HUMAN	-0.2257061	0.656254
sp|P24666|PPAC_HUMAN	-0.2251511	0.7827403
sp|P80303-2|NUCB2_HUMAN	-0.2244511	0.8272904
sp|P17612|KAPCA_HUMAN	-0.2240009	1.2095301
sp|Q96S97|MYADM_HUMAN	-0.223917	1.1932944
sp|O94804|STK10_HUMAN	-0.222559	1.1932944
sp|P04406|G3P_HUMAN	-0.2209168	0.656254
sp|P15586-2|GNS_HUMAN	-0.2201347	1.1505735
sp|Q8TD55|PKHO2_HUMAN	-0.2199211	0
sp|P07741|APT_HUMAN	-0.2184086	0.9968222
sp|Q9NR56-2|MBNL1_HUMAN	-0.2181892	1.1932944
sp|Q15008-4|PSMD6_HUMAN	-0.2180634	0.19149946
sp|P62879|GBB2_HUMAN	-0.2164574	0.5111962
sp|P15529-10|MCP_HUMAN	-0.2163391	0.656254
sp|P48739|PIPNB_HUMAN	-0.2151375	0.45033538
sp|P39060-1|COIA1_HUMAN	-0.214695	0.95332193
sp|Q09028-3|RBBP4_HUMAN	-0.2142677	0.656254

sp|P53618|COPB_HUMAN	-0.2140293	1.203852
sp|P52790|HXK3_HUMAN	-0.2136498	1.3006523
sp|O60547-2|GMDS_HUMAN	-0.2132721	1.1505735
sp|Q06210-2|GFPT1_HUMAN	-0.2116852	1.3006523
sp|P60981|DEST_HUMAN	-0.2109699	0.95332193
sp|Q99426|TBCB_HUMAN	-0.2100678	1.0301651
sp|Q969V3-2|NCLN_HUMAN	-0.2098198	0.91601294
sp|O43488|ARK72_HUMAN	-0.2075615	0.7588735
sp|P08648|ITA5_HUMAN	-0.2074471	0.91601294
sp|P98082-2|DAB2_HUMAN	-0.2072916	1.1932944
sp|P06703|S10A6_HUMAN	-0.2065983	0.7827403
sp|Q9Y3A3-3|PHOCN_HUMAN	-0.2054453	0
sp|P28799-3|GRN_HUMAN	-0.2051048	0.09894868
sp|Q13177|PAK2_HUMAN	-0.2049408	0.7827403
sp|P61457|PHS_HUMAN	-0.2045317	0.7827403
sp|Q03519|TAP2_HUMAN	-0.2044678	0.656254
sp|Q93084-2|AT2A3_HUMAN	-0.203949	0.7827403
sp|Q8N392|RHG18_HUMAN	-0.2037029	1.1505735
sp|P62910|RL32_HUMAN	-0.2034721	0.6070219
sp|Q9UBQ7|GRHPR_HUMAN	-0.202879	1.2941489
sp|Q9NUJ1-3|ABHDA_HUMAN	-0.2025814	0
sp|P01782|HV309_HUMAN	-0.2023468	0.656254
sp|O95571|ETHE1_HUMAN	-0.201767	1.1791906
sp|P51688|SPHM_HUMAN	-0.2012405	0.97209185
sp|Q96HD1|CREL1_HUMAN	-0.2007523	0.45033538
sp|P30519|HMOX2_HUMAN	-0.2004471	0.7827403
sp|Q9BT78|CSN4_HUMAN	-0.1999245	0.7588735
sp|Q06136|KDSR_HUMAN	-0.1997566	1.1932944
sp|Q9H4M9|EHD1_HUMAN	-0.1997414	1.120602
sp|P62195|PRS8_HUMAN	-0.1996803	0.45033538
sp|P60033|CD81_HUMAN	-0.1996403	1.1932944
sp|Q14240-2|IF4A2_HUMAN	-0.1992703	0.8983557
sp|P12955|PEPD_HUMAN	-0.1992645	0.29066643
sp|P20645|MPRD_HUMAN	-0.1990089	1.0301651
sp|P62304|RUXE_HUMAN	-0.1988516	1.1932944
sp|Q15819|UB2V2_HUMAN	-0.1984062	0.656254
sp|Q14258|TRI25_HUMAN	-0.1983757	0.5896263
sp|P42566|EPS15_HUMAN	-0.1977463	1.1932944
sp|P16930|FAAA_HUMAN	-0.1973105	0.656254
sp|P51178-2|PLCD1_HUMAN	-0.1958485	0.43633315
sp|P16422|EPCAM_HUMAN	-0.1956978	1.1932944
sp|Q15637-2|SF01_HUMAN	-0.1955147	1.1932944
sp|O43852-3|CALU_HUMAN	-0.1949444	1.2095301
sp|Q13492-2|PICAL_HUMAN	-0.1944618	0.91601294
sp|Q9Y2X3|NOP58_HUMAN	-0.1937084	0.65625405

sp|P39059|COFA1_HUMAN	-0.1934528	1.2095301
sp|P04424-2|ARLY_HUMAN	-0.1929092	1.0645995
sp|P22307-8|NLTP_HUMAN	-0.1916513	0.7061832
sp|Q9BW04|SARG_HUMAN	-0.1914482	0.45033538
sp|Q53GQ0|DHB12_HUMAN	-0.1910152	0.35795313
sp|P54727|RD23B_HUMAN	-0.188427	0.7061832
sp|Q12792-3|TWF1_HUMAN	-0.1879349	0.19149946
sp|O00487|PSDE_HUMAN	-0.1874733	0.19149946
sp|P48163-2|MAOX_HUMAN	-0.1873312	0.35795313
sp|P11498|PYC_HUMAN	-0.1861687	0.61034113
sp|P39019|RS19_HUMAN	-0.1854305	0.5204253
sp|P29972-2|AQP1_HUMAN	-0.1840248	0.45033538
sp|Q92556|ELMO1_HUMAN	-0.1821613	0.8983557
sp|Q9NZU5-2|LMCD1_HUMAN	-0.1821404	0.5886828
sp|P30626-2|SORCN_HUMAN	-0.1819267	0.68888646
sp|Q86U42-2|PABP2_HUMAN	-0.1816673	0.656254
sp|Q13232|NDK3_HUMAN	-0.1813622	0.5111962
sp|P18583-10|SON_HUMAN	-0.1809502	0.19149946
sp|Q709C8-3|VP13C_HUMAN	-0.1808586	1.1240381
sp|O94973-2|AP2A2_HUMAN	-0.1804142	1.1683742
sp|P26640|SYVC_HUMAN	-0.1803227	0.6063746
sp|Q92747|ARC1A_HUMAN	-0.1795769	0.656254
sp|Q8NF91-4|SYNE1_HUMAN	-0.1784315	0.45033538
sp|Q99436|PSB7_HUMAN	-0.1773558	0.45033538
sp|P0DJI8|SAA1_HUMAN	-0.1772423	0
sp|Q6DN03|H2B2C_HUMAN	-0.1756973	0
sp|P29400-2|CO4A5_HUMAN	-0.1752863	0
sp|P33316|DUT_HUMAN	-0.1747952	1.1932944
sp|P55795|HNRH2_HUMAN	-0.1734467	0.30372584
sp|Q96C86|DCPS_HUMAN	-0.1717777	0.91601294
sp|O15372|EIF3H_HUMAN	-0.1716538	0.5204253
sp|Q7Z7H5-3|TMED4_HUMAN	-0.1711483	0.19149946
sp|O94855-2|SC24D_HUMAN	-0.171133	0.19149946
sp|O75112-7|LDB3_HUMAN	-0.1710606	1.1505735
sp|O75340-2|PDCD6_HUMAN	-0.1699657	0.09894868
sp|Q13838-2|DX39B_HUMAN	-0.1699028	0.1342476
sp|P12277|KCRB_HUMAN	-0.169548	0.907945
sp|P13693|TCTP_HUMAN	-0.1693268	0.7827403
sp|Q9Y4L1|HYOU1_HUMAN	-0.1686974	1.1316391
sp|Q9UEY8|ADDG_HUMAN	-0.1686935	0.98032326
sp|Q12797-10|ASPH_HUMAN	-0.168499	0.7022581
sp|P07305-2|H10_HUMAN	-0.1684227	0
sp|O95833|CLIC3_HUMAN	-0.1682072	0.09894868
sp|P49961-6|ENTP1_HUMAN	-0.1681671	0.35795313
sp|P62987|RL40_HUMAN	-0.1680145	1.0634323

sp|P31948|STIP1_HUMAN	-0.1679916	0.656254
sp|P53602|MVD1_HUMAN	-0.1670647	0.91601294
sp|P08238|HS90B_HUMAN	-0.1670513	1.0242546
sp|P10644|KAP0_HUMAN	-0.1660824	0.312067
sp|P25325-2|THTM_HUMAN	-0.1654959	0.5692702
sp|O00764-2|PDXK_HUMAN	-0.1635056	1.1240381
sp|Q9P2T1-2|GMPR2_HUMAN	-0.1634846	0.48520416
sp|Q9UGI8-2|TES_HUMAN	-0.1628361	0.7420189
sp|P11234-2|RALB_HUMAN	-0.1617909	0.656254
sp|Q96DG6|CMBL_HUMAN	-0.1608505	0.91601294
sp|P55145|MANF_HUMAN	-0.1604424	1.1932944
sp|O14980|XPO1_HUMAN	-0.1604137	0.91601294
sp|O94776|MTA2_HUMAN	-0.1590519	0.8983557
sp|P33176|KINH_HUMAN	-0.1586494	0.77163273
sp|O75608-2|LYPA1_HUMAN	-0.1580792	1.1932944
sp|P22102|PUR2_HUMAN	-0.1580219	0.6298893
sp|P56134-3|ATPK_HUMAN	-0.157856	0
sp|Q96EP5-2|DAZP1_HUMAN	-0.156723	0.45033538
sp|O75937|DNJC8_HUMAN	-0.1562347	1.0485198
sp|P42167|LAP2B_HUMAN	-0.1562157	1.1932944
sp|P23229-4|ITA6_HUMAN	-0.1555119	1.1932944
sp|O95466-2|FMNL1_HUMAN	-0.1554909	1.1932944
sp|P01892|1A02_HUMAN	-0.1552296	0.35795313
sp|O95837|GNA14_HUMAN	-0.1551476	0.656254
sp|P49720|PSB3_HUMAN	-0.1549854	1.1791906
sp|P61026|RAB10_HUMAN	-0.1540794	0.7827403
sp|Q9UBV8|PEF1_HUMAN	-0.1538487	0.91601294
sp|Q9Y6A4|CFA20_HUMAN	-0.1537018	0.45033538
sp|P30049|ATPD_HUMAN	-0.1534405	1.1932944
sp|Q9NTK5|OLA1_HUMAN	-0.1534195	0.19149946
sp|O60763-2|USO1_HUMAN	-0.1532021	0.7414989
sp|Q9UNE7-2|CHIP_HUMAN	-0.1530609	0.45033538
sp|P12268|IMDH2_HUMAN	-0.151804	0.7827403
sp|P48147|PPCE_HUMAN	-0.1506004	1.0645995
sp|O43681|ASNA_HUMAN	-0.1501274	0.5204253
sp|P40306|PSB10_HUMAN	-0.1498394	0.30372584
sp|Q9HAV0|GBB4_HUMAN	-0.1489506	0
sp|P51692|STA5B_HUMAN	-0.1483746	1.1932944
sp|P43243|MATR3_HUMAN	-0.1482315	1.3006523
sp|Q9P2X0-2|DPM3_HUMAN	-0.1475678	0.45033538
sp|Q9H299|SH3L3_HUMAN	-0.1471252	1.1932944
sp|P42126-2|ECI1_HUMAN	-0.1458321	1.1932944
sp|Q13557-12|KCC2D_HUMAN	-0.1448746	0.9533461
sp|P62314|SMD1_HUMAN	-0.1448326	0.45033538
sp|Q8WVM8|SCFD1_HUMAN	-0.144371	0.17511293

sp|P23588|IF4B_HUMAN	-0.1432781	0.8983557
sp|Q14914-2|PTGR1_HUMAN	-0.1431732	0.30372584
sp|Q96C19|EFHD2_HUMAN	-0.143158	0.7061832
sp|Q8NCW5|NNRE_HUMAN	-0.1414566	0.5111962
sp|Q96BM9|ARL8A_HUMAN	-0.1412468	0.656254
sp|P54652|HSP72_HUMAN	-0.1402931	0.35795313
sp|P20591|MX1_HUMAN	-0.1402531	0.95332193
sp|Q92598-2|HS105_HUMAN	-0.139782	0.656254
sp|Q14141-2|SEPT6_HUMAN	-0.1396103	0.656254
sp|O15067|PUR4_HUMAN	-0.1395721	0.7827403
sp|P10412|H14_HUMAN	-0.1390114	0
sp|P55010|IF5_HUMAN	-0.1388874	0.40256184
sp|P48449-3|ERG7_HUMAN	-0.1385498	1.0485198
sp|Q9NY33|DPP3_HUMAN	-0.138422	0.8235348
sp|Q9BZE9-2|ASPC1_HUMAN	-0.1377735	0
sp|Q7L5N1|CSN6_HUMAN	-0.1376991	0.7827403
sp|Q9H0W9-2|CK054_HUMAN	-0.1364155	0.4075265
sp|P30520|PURA2_HUMAN	-0.1362248	0.34323573
sp|O14617-4|AP3D1_HUMAN	-0.1359825	0.45033538
sp|Q9NZB2-6|F120A_HUMAN	-0.135891	1.1932944
sp|P01624|KV315_HUMAN	-0.1354828	0.7827403
sp|Q9NZL9|MAT2B_HUMAN	-0.1351738	0.45563722
sp|P47985|UCRI_HUMAN	-0.1349945	0.2178309
sp|Q86Y82|STX12_HUMAN	-0.1349897	0.26737198
sp|Q9UBT2|SAE2_HUMAN	-0.1345139	0.7588735
sp|P63241|IF5A1_HUMAN	-0.1343765	0.656254
sp|P30466|1B18_HUMAN	-0.1328392	0.656254
sp|O60504-2|VINEX_HUMAN	-0.132267	0.95332193
sp|P61018-2|RAB4B_HUMAN	-0.1320362	0.656254
sp|Q9Y224|RTRAF_HUMAN	-0.1301918	0.4075265
sp|Q13404|UB2V1_HUMAN	-0.1287079	0.2178309
sp|P01920|DQB1_HUMAN	-0.1284866	0.19149946
sp|Q9BVC6|TM109_HUMAN	-0.1279049	0.5204253
sp|P05166-2|PCCB_HUMAN	-0.1274796	0.79861414
sp|P61758|PFD3_HUMAN	-0.1272259	0.19149946
sp|Q6NVY1|HIBCH_HUMAN	-0.1270752	0.5111962
sp|Q9UJZ1-2|STML2_HUMAN	-0.1270533	0.91601294
sp|P31942-2|HNRH3_HUMAN	-0.1265583	0.65625405
sp|P84098|RL19_HUMAN	-0.1256638	0.19149946
sp|P02792|FRIL_HUMAN	-0.1256409	1.0353775
sp|P14618-2|KPYM_HUMAN	-0.1256104	0.2178309
sp|P55290-4|CAD13_HUMAN	-0.1243629	0.21439649
sp|Q14019|COTL1_HUMAN	-0.1239624	0.33495146
sp|P06310|KV230_HUMAN	-0.1235008	0
sp|Q53GG5-2|PDLI3_HUMAN	-0.1234474	0.40256184

sp|P46779-2|RL28_HUMAN	-0.1230145	1.0485198
sp|P12111-2|CO6A3_HUMAN	-0.1228142	0.7217418
sp|Q13464|ROCK1_HUMAN	-0.122345	0.28516325
sp|P12004|PCNA_HUMAN	-0.1220894	0.45033538
sp|Q92522|H1X_HUMAN	-0.1218967	0
sp|O00429-6|DNM1L_HUMAN	-0.1207981	0.87291557
sp|P08237-3|PFKAM_HUMAN	-0.1199608	0.09339783
sp|P13928|ANXA8_HUMAN	-0.1195564	0
sp|Q15029-2|U5S1_HUMAN	-0.1193247	0.59872687
sp|O60814|H2B1K_HUMAN	-0.1183243	0
sp|B5ME19|EIFCL_HUMAN	-0.1172295	0.5204253
sp|Q9BZZ5-5|API5_HUMAN	-0.1168966	0.56808305
sp|Q969H8|MYDGF_HUMAN	-0.1168385	0.7061832
sp|Q8TAT6-2|NPL4_HUMAN	-0.1159229	0.45563722
sp|O75503|CLN5_HUMAN	-0.1155834	0.91601294
sp|O43396|TXNL1_HUMAN	-0.1155701	0
sp|Q6YHK3|CD109_HUMAN	-0.1146965	0.5176613
sp|Q16774|KGUA_HUMAN	-0.1146774	0
sp|Q9BUF5|TBB6_HUMAN	-0.1144447	0.8983557
sp|Q13243-3|SRSF5_HUMAN	-0.1143532	0.656254
sp|Q04323-2|UBXN1_HUMAN	-0.1136723	0
sp|P16298-4|PP2BB_HUMAN	-0.1136379	0.312067
sp|Q15417|CNN3_HUMAN	-0.1133099	0.91601294
sp|P50991-2|TCPD_HUMAN	-0.1132774	1.1054204
sp|P61006|RAB8A_HUMAN	-0.1130486	0.7827403
sp|P30044-2|PRDX5_HUMAN	-0.112855	0.656254
sp|Q9HC38|GLOD4_HUMAN	-0.1125526	0.7957244
sp|P50135|HNMT_HUMAN	-0.1125431	0
sp|Q16853|AOC3_HUMAN	-0.1110916	1.2474942
sp|O95861-4|BPNT1_HUMAN	-0.1105804	0.6298893
sp|Q7Z4W1|DCXR_HUMAN	-0.1100464	0.8983557
sp|O95302-3|FKBP9_HUMAN	-0.109684	0
sp|O15260-2|SURF4_HUMAN	-0.1095867	0.19149946
sp|P68400|CSK21_HUMAN	-0.1087608	1.1505735
sp|Q969G5|CAVN3_HUMAN	-0.1087341	0.5204253
sp|P69891|HBG1_HUMAN	-0.108448	0.656254
sp|P09619|PGFRB_HUMAN	-0.1078491	0.35795313
sp|P28072|PSB6_HUMAN	-0.1066074	0.35795313
sp|Q93077|H2A1C_HUMAN	-0.105978	0
sp|P35754|GLRX1_HUMAN	-0.1048698	0.48520416
sp|Q04760-2|LGUL_HUMAN	-0.1039925	0.5111962
sp|P46108|CRK_HUMAN	-0.1039391	0.03367973
sp|P37108|SRP14_HUMAN	-0.1037502	0.19149946
sp|P60983|GMFB_HUMAN	-0.1037102	1.1505735
sp|Q3LXA3|TKFC_HUMAN	-0.1034699	0.55270666

sp|O00391|QSOX1_HUMAN	-0.1032333	0.45033538
sp|P12814|ACTN1_HUMAN	-0.1027527	0
sp|Q9H4G4|GAPR1_HUMAN	-0.1022358	0.35795313
sp|P17980|PRS6A_HUMAN	-0.1019783	0.42646354
sp|Q96CN7|ISOC1_HUMAN	-0.1017532	0.48520416
sp|Q8N163-2|CCAR2_HUMAN	-0.1017532	0.91601294
sp|P52306-4|GDS1_HUMAN	-0.1003513	1.1505735
sp|Q96CX2|KCD12_HUMAN	-0.0999985	0.89178735
sp|P16070-7|CD44_HUMAN	-0.0997524	0
sp|O60716-14|CTND1_HUMAN	-0.098608	0.2178309
sp|P58107|EPIPL_HUMAN	-0.0985556	0.63371044
sp|Q9Y265|RUVB1_HUMAN	-0.0983048	0.23541966
sp|P15374|UCHL3_HUMAN	-0.0973291	0.5692702
sp|O60664-4|PLIN3_HUMAN	-0.0967236	0.20467198
sp|P46821|MAP1B_HUMAN	-0.0963802	0.1342476
sp|P51665|PSMD7_HUMAN	-0.0960712	0.45033538
sp|O60313-10|OPA1_HUMAN	-0.09585	0.19149946
sp|Q6UVK1|CSPG4_HUMAN	-0.0949173	0.56808305
sp|P62333|PRS10_HUMAN	-0.0940266	0.7498006
sp|P62913-2|RL11_HUMAN	-0.0925903	0.7827403
sp|P30533|AMRP_HUMAN	-0.0924568	0.84879977
sp|Q13642-1|FHL1_HUMAN	-0.0921707	0.656254
sp|P22033|MUTA_HUMAN	-0.0916233	0
sp|P01112|RASH_HUMAN	-0.0913563	0
sp|P78417-2|GSTO1_HUMAN	-0.0899181	0.656254
sp|O00232|PSD12_HUMAN	-0.0898552	0.40256184
sp|Q96FN4|CPNE2_HUMAN	-0.0898476	0
sp|P61225|RAP2B_HUMAN	-0.089756	0.45033538
sp|Q08945|SSRP1_HUMAN	-0.0893211	1.0076748
sp|Q5R3I4|TTC38_HUMAN	-0.088192	0.19149946
sp|P11217|PYGM_HUMAN	-0.0876007	0.656254
sp|P45974-2|UBP5_HUMAN	-0.0874996	0.26295313
sp|Q9NZK5|ADA2_HUMAN	-0.0873947	0.19149946
sp|Q92499|DDX1_HUMAN	-0.0867767	0.83646035
sp|Q8NHV1|GIMA7_HUMAN	-0.0859146	0.45033538
sp|P36873-2|PP1G_HUMAN	-0.0839615	0.45033538
sp|Q13126-2|MTAP_HUMAN	-0.0838776	0.6509351
sp|P09429|HMGB1_HUMAN	-0.0836945	0.19149946
sp|O75955|FLOT1_HUMAN	-0.0833721	0.94599336
sp|O75306-2|NDUS2_HUMAN	-0.0824375	0.30372584
sp|Q08722-2|CD47_HUMAN	-0.0823841	0.7061832
sp|P11216|PYGB_HUMAN	-0.0821037	0.8321823
sp|P32456|GBP2_HUMAN	-0.0815258	0.45033538
sp|P39687|AN32A_HUMAN	-0.0812569	0.09894868
sp|P02675|FIBB_HUMAN	-0.0810394	0.8652954

sp|A0FGR8-2|ESYT2_HUMAN	-0.0804291	0.30372584
sp|P67809|YBOX1_HUMAN	-0.0803471	0
sp|Q9HBL0|TENS1_HUMAN	-0.0800304	0.06210651
sp|P28066|PSA5_HUMAN	-0.0798359	0.4075265
sp|P57737-4|CORO7_HUMAN	-0.0789976	0.45033538
sp|P27694|RFA1_HUMAN	-0.0788193	0.33495146
sp|P48681|NEST_HUMAN	-0.0788136	0.19818917
sp|Q9BRF8-2|CPPED_HUMAN	-0.078331	0.2178309
sp|P62753|RS6_HUMAN	-0.0783157	0.35795313
sp|O00571-2|DDX3X_HUMAN	-0.0779276	0.7061832
sp|Q16181-2|SEPT7_HUMAN	-0.0776691	0.880485
sp|P61088|UBE2N_HUMAN	-0.076849	0.19149946
sp|P21399|ACOC_HUMAN	-0.0766859	0.3852666
sp|Q9BX97|PLVAP_HUMAN	-0.076088	0
sp|P14174|MIF_HUMAN	-0.0757904	0
sp|P22748|CAH4_HUMAN	-0.0757198	0.4075265
sp|P02746|C1QB_HUMAN	-0.0736313	0
sp|Q14108|SCRB2_HUMAN	-0.073513	0.7827403
sp|P50502|F10A1_HUMAN	-0.0731144	0.46530083
sp|Q01955|CO4A3_HUMAN	-0.0726223	1.1932944
sp|Q96M27-3|PRRC1_HUMAN	-0.0716572	0.7827403
sp|P0DP25|CALM3_HUMAN	-0.0702477	0.93112767
sp|P61803|DAD1_HUMAN	-0.0700283	0
sp|Q9BTV4|TMM43_HUMAN	-0.0700111	0.4075265
sp|P02763|A1AG1_HUMAN	-0.0698643	0.43633315
sp|O15031|PLXB2_HUMAN	-0.0693684	0.34244674
sp|Q07954|LRP1_HUMAN	-0.0689468	0.7911032
sp|Q13247-3|SRSF6_HUMAN	-0.0688553	0.19149946
sp|P17987|TCPA_HUMAN	-0.0684605	0.6385048
sp|Q16787-3|LAMA3_HUMAN	-0.0684452	0.656254
sp|P42785-2|PCP_HUMAN	-0.068325	0
sp|Q15436|SC23A_HUMAN	-0.0682869	0.21439649
sp|P01034|CYTC_HUMAN	-0.0682812	0.7827403
sp|Q9Y2Q5|LTOR2_HUMAN	-0.0674095	0
sp|Q9UK22|FBX2_HUMAN	-0.0673103	0
sp|Q15046|SYK_HUMAN	-0.0667667	0
sp|P62937|PPIA_HUMAN	-0.0661545	0
sp|Q92896-2|GSLG1_HUMAN	-0.0656052	0.05634482
sp|O00754-2|MA2B1_HUMAN	-0.0645161	0.8983557
sp|P48637|GSHB_HUMAN	-0.0641727	0.5176613
sp|P27487|DPP4_HUMAN	-0.0626812	0.06291623
sp|Q86WV6|STING_HUMAN	-0.0625286	0.09894868
sp|P08571|CD14_HUMAN	-0.0617332	0.6298893
sp|Q9Y490|TLN1_HUMAN	-0.0614738	0.8146241
sp|P04632|CPNS1_HUMAN	-0.0609732	0.19149946

sp|P17174|AATC_HUMAN	-0.0603943	0.30819008
sp|P26599-2|PTBP1_HUMAN	-0.0603008	0.05145278
sp|P27338|AOFB_HUMAN	-0.0601273	0.30819008
sp|Q7LG56-6|RIR2B_HUMAN	-0.0587978	0.19149946
sp|Q96HN2-2|SAHH3_HUMAN	-0.0583954	0
sp|P55209-2|NP1L1_HUMAN	-0.0568962	0
sp|O75367-2|H2AY_HUMAN	-0.0566921	0.56808305
sp|Q12765|SCRN1_HUMAN	-0.0563622	0.1342476
sp|P02751-15|FINC_HUMAN	-0.0562725	0.656254
sp|Q08380|LG3BP_HUMAN	-0.0555229	0.12897664
sp|O00592-2|PODXL_HUMAN	-0.0545273	0.45033538
sp|Q16643-3|DREB_HUMAN	-0.0532427	0.35193655
sp|P06748-2|NPM_HUMAN	-0.0523176	0.35795313
sp|O15498-2|YKT6_HUMAN	-0.0521202	0.19149946
sp|P06756-3|ITAV_HUMAN	-0.0512848	0.84879977
sp|O14787-2|TNPO2_HUMAN	-0.0511227	0
sp|P23528|COF1_HUMAN	-0.0489273	0.4490988
sp|O15400-2|STX7_HUMAN	-0.0487576	0.33495146
sp|P41250|GARS_HUMAN	-0.0481272	0.40223262
sp|P02679-2|FIBG_HUMAN	-0.0480232	0.14652808
sp|P01011|AACT_HUMAN	-0.0472126	0.37277833
sp|Q9Y3B3|TMED7_HUMAN	-0.0470943	0
sp|O14786|NRP1_HUMAN	-0.0468578	0.19149946
sp|P62857|RS28_HUMAN	-0.0466805	0.2178309
sp|P58546|MTPN_HUMAN	-0.0463295	0.2178309
sp|Q00796|DHSO_HUMAN	-0.0456276	0.09894868
sp|Q15005|SPCS2_HUMAN	-0.045496	0.35795313
sp|P49755|TMEDA_HUMAN	-0.0454559	0.06291623
sp|Q9HCB6|SPON1_HUMAN	-0.0443592	0.04454162
sp|Q9Y696|CLIC4_HUMAN	-0.0437889	0.06842031
sp|Q9Y3A5|SBDS_HUMAN	-0.0437851	0.52598757
sp|Q9C0B1|FTO_HUMAN	-0.0436525	0.19149946
sp|O60884|DNJA2_HUMAN	-0.0432282	0.6298893
sp|P25787|PSA2_HUMAN	-0.0430298	0.02662771
sp|P11279|LAMP1_HUMAN	-0.0427322	0
sp|O60825-2|F262_HUMAN	-0.0424061	0
sp|Q9NP79|VTA1_HUMAN	-0.0423431	0.09894868
sp|Q92817|EVPL_HUMAN	-0.0422859	0.6760564
sp|Q9UKK9|NUDT5_HUMAN	-0.04212	0
sp|Q9NRN5-2|OLFL3_HUMAN	-0.0406208	0.01311907
sp|P36543-2|VATE1_HUMAN	-0.0402813	0.2178309
sp|Q92542|NICA_HUMAN	-0.0396767	0.2178309
sp|Q16401-2|PSMD5_HUMAN	-0.0393124	0.04454162
sp|O95336|6PGL_HUMAN	-0.0392723	0.3005443
sp|P01619|KV320_HUMAN	-0.0391617	0.656254

sp|Q9UHX1-6|PUF60_HUMAN	-0.0387383	0.35795313
sp|Q5JRX3-3|PREP_HUMAN	-0.0387115	0.19149946
sp|Q92900-2|RENT1_HUMAN	-0.0366135	0.10017801
sp|Q9NUQ9|FA49B_HUMAN	-0.0364056	0.09894868
sp|Q63ZY3-3|KANK2_HUMAN	-0.0360184	0.56808305
sp|P50225|ST1A1_HUMAN	-0.0357857	0
sp|O76041-2|NEBL_HUMAN	-0.0355339	0
sp|P61201-2|CSN2_HUMAN	-0.0346813	0.19149946
sp|Q9P0V9-2|SEP10_HUMAN	-0.0346298	0.23541966
sp|P23219-2|PGH1_HUMAN	-0.0342808	0.30372584
sp|Q5EBM0|CMPK2_HUMAN	-0.0341148	0.5204253
sp|Q02952-2|AKA12_HUMAN	-0.0337563	0
sp|Q08379|GOGA2_HUMAN	-0.0328751	0
sp|P43686|PRS6B_HUMAN	-0.0327091	0.4490988
sp|P23497|SP100_HUMAN	-0.0326767	0
sp|P13987-2|CD59_HUMAN	-0.0318527	0.35795313
sp|Q99598|TSNAX_HUMAN	-0.0315723	0.28516325
sp|P49721|PSB2_HUMAN	-0.0301971	0.312067
sp|P05388|RLA0_HUMAN	-0.0294781	0.04707252
sp|Q9UBC2-2|EP15R_HUMAN	-0.0288162	0.19149946
sp|Q12882|DPYD_HUMAN	-0.0286884	0
sp|O75964|ATP5L_HUMAN	-0.0272026	0.19149946
sp|P25685-2|DNJB1_HUMAN	-0.0267715	0
sp|O75347|TBCA_HUMAN	-0.0266953	0.09894868
sp|P30085|KCY_HUMAN	-0.0253906	0.09894868
sp|O15145|ARPC3_HUMAN	-0.0247612	0.09894868
sp|P09871|C1S_HUMAN	-0.0239124	0.5204253
sp|P28074|PSB5_HUMAN	-0.0237255	0.26737198
sp|P10599-2|THIO_HUMAN	-0.0228691	0.06291623
sp|P21333-2|FLNA_HUMAN	-0.020813	0.18494359
sp|Q93052|LPP_HUMAN	-0.02005	0.02388411
sp|P61020|RAB5B_HUMAN	-0.0198841	0.2178309
sp|O75915|PRAF3_HUMAN	-0.0194235	0.09894868
sp|P04275|VWF_HUMAN	-0.0189667	0.07378665
sp|P22352|GPX3_HUMAN	-0.0188541	0.09894868
sp|Q9BS40|LXN_HUMAN	-0.0188084	0.21439649
sp|Q15661|TRYB1_HUMAN	-0.0187874	0.356152
sp|Q9C0E8-4|LNP_HUMAN	-0.0175114	0.19149946
sp|Q9UJS0-2|CMC2_HUMAN	-0.0174141	0
sp|Q86UX2-2|ITIH5_HUMAN	-0.0167999	0.19149946
sp|Q13596-2|SNX1_HUMAN	-0.0166435	0
sp|P02462|CO4A1_HUMAN	-0.0165691	0.09894868
sp|P27918|PROP_HUMAN	-0.0164557	0.19149946
sp|P27695|APEX1_HUMAN	-0.016036	0
sp|Q9UNZ2-5|NSF1C_HUMAN	-0.0160103	0.14927356

sp|Q15833-2|STXB2_HUMAN	-0.0159073	0
sp|Q96BW5-2|PTER_HUMAN	-0.0146046	0
sp|Q8WU39|MZB1_HUMAN	-0.0136776	0.06986027
sp|Q86VP6|CAND1_HUMAN	-0.011982	0.28186345
sp|Q9UJU6-2|DBNL_HUMAN	-0.0116386	0.06291623
sp|P0DP03|HV335_HUMAN	-0.0105858	0
sp|Q9UBW8|CSN7A_HUMAN	-0.0104313	0.09894868
sp|Q15257-2|PTPA_HUMAN	-0.0084991	0.09339783
sp|Q99961-3|SH3G1_HUMAN	-0.008419	0
sp|Q6WCQ1-2|MPRIP_HUMAN	-0.0082731	0.08077049
sp|P62070-4|RRAS2_HUMAN	-0.0076199	0
sp|Q14112-2|NID2_HUMAN	-0.0056038	0.368128
sp|Q16527|CSRP2_HUMAN	-0.0054531	0.2178309
sp|O60437|PEPL_HUMAN	-0.0052338	0.19728164
sp|Q15075|EEA1_HUMAN	-0.0048962	0.10258728
sp|O14950|ML12B_HUMAN	-0.0048389	0.40256184
sp|O75489|NDUS3_HUMAN	-0.0045185	0.06291623
sp|Q9Y2B0|CNPY2_HUMAN	-0.0042181	0.35795313
sp|Q9UI12-2|VATH_HUMAN	-0.0021458	0.14672586
sp|P30086|PEBP1_HUMAN	-0.0019226	0.02174174
sp|P51636-2|CAV2_HUMAN	-8.16E-04	0
sp|Q6DKJ4|NXN_HUMAN	1.56E-04	0
sp|Q92930|RAB8B_HUMAN	0.00184631	0
sp|Q99439|CNN2_HUMAN	0.00239372	0.06842031
sp|P61970|NTF2_HUMAN	0.00253868	0
sp|Q09666|AHNK_HUMAN	0.00361061	0.57329684
sp|P62736|ACTA_HUMAN	0.00377464	0
sp|P62191-2|PRS4_HUMAN	0.00509262	0.09894868
sp|P22105-1|TENX_HUMAN	0.00634766	0.00778498
sp|P07360|CO8G_HUMAN	0.00676537	0.19149946
sp|Q14192|FHL2_HUMAN	0.00680923	0.5692702
sp|Q13813-2|SPTN1_HUMAN	0.00701523	0
sp|P07942|LAMB1_HUMAN	0.00707722	0.01542275
sp|P01780|HV307_HUMAN	0.00754166	0
sp|Q04837|SSBP_HUMAN	0.00865555	0.19149946
sp|O75368|SH3L1_HUMAN	0.00933838	0
sp|P05387|RLA2_HUMAN	0.00959587	0.19149946
sp|P30512|1A29_HUMAN	0.01011658	0.09894868
sp|P23193-2|TCEA1_HUMAN	0.011343	0.1558116
sp|Q14980-2|NUMA1_HUMAN	0.01163864	0.3425803
sp|P13798|ACPH_HUMAN	0.01165581	0.0315703
sp|O60506-3|HNRPQ_HUMAN	0.0119915	0.1342476
sp|P35637-2|FUS_HUMAN	0.01230907	0.45033538
sp|P09104-2|ENOG_HUMAN	0.0123291	0.5832693
sp|Q9NR12-2|PDLI7_HUMAN	0.01262283	0.06986027

sp|P48643|TCPE_HUMAN	0.01421928	0.45546502
sp|O14974-3|MYPT1_HUMAN	0.01571274	0.02174174
sp|P35858-2|ALS_HUMAN	0.01775169	0.19149946
sp|P52888|THOP1_HUMAN	0.01780129	0
sp|Q14254|FLOT2_HUMAN	0.01851463	0
sp|Q9Y639-4|NPTN_HUMAN	0.01865196	0
sp|P08708|RS17_HUMAN	0.01921463	0.4075265
sp|Q9Y6W5|WASF2_HUMAN	0.02006722	0.35795313
sp|P62633-3|CNBP_HUMAN	0.02074051	0
sp|Q99497|PARK7_HUMAN	0.02090073	0.26737198
sp|P35611-2|ADDA_HUMAN	0.02112198	0.33495146
sp|Q6NY19-2|KANK3_HUMAN	0.02114677	0.19149946
sp|Q07960|RHG01_HUMAN	0.02182198	0.11949348
sp|Q08431|MFGM_HUMAN	0.02196121	0
sp|P54578-3|UBP14_HUMAN	0.0220871	0
sp|Q9NRV9|HEBP1_HUMAN	0.02254677	0.21439649
sp|Q13561-2|DCTN2_HUMAN	0.02370834	0.2565741
sp|P40261|NNMT_HUMAN	0.02425766	0.28516325
sp|P01859|IGHG2_HUMAN	0.02439117	0.41804093
sp|P10301|RRAS_HUMAN	0.02493668	0.09894868
sp|P09382|LEG1_HUMAN	0.02534676	0.31741163
sp|P01591|IGJ_HUMAN	0.0254364	0
sp|Q7Z4H8|PLGT3_HUMAN	0.02618408	0
sp|P31937|3HIDH_HUMAN	0.02751541	0.35795313
sp|Q13630|FCL_HUMAN	0.02753067	0.19149946
sp|Q6ZVM7-3|TM1L2_HUMAN	0.0280714	0
sp|Q6UWY5|OLFL1_HUMAN	0.02905464	0.16702904
sp|P46783|RS10_HUMAN	0.03151417	0.45033538
sp|Q15063-3|POSTN_HUMAN	0.03199577	0.19149946
sp|Q9UNS2|CSN3_HUMAN	0.03209686	0.656254
sp|Q16630-2|CPSF6_HUMAN	0.03211594	0.2178309
sp|Q9UPN3|MACF1_HUMAN	0.03284073	0.4488957
sp|O43294|TGFI1_HUMAN	0.03372955	0
sp|O43790|KRT86_HUMAN	0.03388023	0
sp|Q15121|PEA15_HUMAN	0.03424835	0.2178309
sp|O95865|DDAH2_HUMAN	0.03450966	0.12054814
sp|P06753-2|TPM3_HUMAN	0.03492355	0.19149946
sp|P12270|TPR_HUMAN	0.03521729	0.5368848
sp|P67936-2|TPM4_HUMAN	0.03581619	0.2178309
sp|Q00341-2|VIGLN_HUMAN	0.03590775	0.02662771
sp|O00151|PDLI1_HUMAN	0.0371666	0.09851671
sp|O15296|LX15B_HUMAN	0.03783035	0.06291623
sp|P61224|RAP1B_HUMAN	0.03798485	0
sp|P0CG38|POTEI_HUMAN	0.03869247	0
sp|O14791-2|APOL1_HUMAN	0.03940201	0

sp|P40123-2|CAP2_HUMAN	0.03953934	0
sp|Q9UPQ0|LIMC1_HUMAN	0.03980064	0.02174174
sp|P22392-2|NDKB_HUMAN	0.04022026	0.84879977
sp|P12110|CO6A2_HUMAN	0.04149437	0.79584783
sp|P54802|ANAG_HUMAN	0.04163933	0
sp|Q04446|GLGB_HUMAN	0.04226017	0.7588735
sp|Q15746-2|MYLK_HUMAN	0.04256249	0.8092523
sp|Q13867|BLMH_HUMAN	0.04291534	0
sp|P35555|FBN1_HUMAN	0.04330063	0.1454734
sp|Q6PCB0|VWA1_HUMAN	0.04338646	0.09894868
sp|Q9HB71|CYBP_HUMAN	0.04428864	0.312067
sp|Q13526|PIN1_HUMAN	0.04504013	0
sp|Q6IAA8|LTOR1_HUMAN	0.04594994	0.19149946
sp|Q8WXX5|DNJC9_HUMAN	0.04597855	0.19149946
sp|Q9H8L6|MMRN2_HUMAN	0.04598808	0.19149946
sp|P00736|C1R_HUMAN	0.04623985	0.09894868
sp|P78371|TCPB_HUMAN	0.04645252	0.50452983
sp|Q9C0C2|TB182_HUMAN	0.04800415	0.7498006
sp|Q92629-3|SGCD_HUMAN	0.0490036	0.30372584
sp|Q9UGT4|SUSD2_HUMAN	0.04925537	0.23541966
sp|P0DOX8|IGL1_HUMAN	0.04946518	0
sp|Q9NZ32|ARP10_HUMAN	0.04979897	0.45033538
sp|P35579|MYH9_HUMAN	0.05010605	1.2301164
sp|P12109|CO6A1_HUMAN	0.05039024	0.24452867
sp|Q5K4L6|S27A3_HUMAN	0.05084992	0.04707252
sp|O60610-2|DIAP1_HUMAN	0.05100727	0.1342476
sp|Q9UHL4|DPP2_HUMAN	0.05164337	1.1344727
sp|P0DOX3|IGD_HUMAN	0.05201721	0.656254
sp|O94811|TPPP_HUMAN	0.05537605	0
sp|Q9NYL9|TMOD3_HUMAN	0.05576897	0.5111962
sp|P01861|IGHG4_HUMAN	0.05597687	0.3201336
sp|Q9NQG5|RPR1B_HUMAN	0.05735016	0
sp|Q13751|LAMB3_HUMAN	0.05862618	0.35795313
sp|A0A0C4DH38|HV551_HUMA	0.06027603	0.19149946
sp|P55268|LAMB2_HUMAN	0.0610714	1.157721
sp|Q14651|PLSI_HUMAN	0.06328678	0
sp|Q9BWD1|THIC_HUMAN	0.06396294	0.19149946
sp|Q9NVD7|PARVA_HUMAN	0.06618118	0.7827403
sp|Q14894|CRYM_HUMAN	0.0694027	0.7827403
sp|Q08378|GOGA3_HUMAN	0.07129097	0.91601294
sp|Q8WX93-5|PALLD_HUMAN	0.07131958	0.5204253
sp|P67936|TPM4_HUMAN	0.07154083	0.94599336
sp|Q14847|LASP1_HUMAN	0.07272339	0.23973103
sp|P13647|K2C5_HUMAN	0.07345009	0.94928885
sp|P50148|GNAQ_HUMAN	0.07382393	0.1342476

sp|P00568|KAD1_HUMAN	0.07630539	1.2987719
sp|P07195|LDHB_HUMAN	0.0769062	0.91601294
sp|Q15063-2|POSTN_HUMAN	0.07770157	0.656254
sp|P23142-4|FBLN1_HUMAN	0.07791042	0
sp|Q14118|DAG1_HUMAN	0.07813835	0.7061832
sp|Q93009-3|UBP7_HUMAN	0.07818604	0
sp|Q10589-2|BST2_HUMAN	0.0784626	0.35795313
sp|P56537|IF6_HUMAN	0.08096504	0.44572112
sp|P20339-2|RAB5A_HUMAN	0.08111858	0
sp|P10643|CO7_HUMAN	0.08203125	0.06210651
sp|Q9Y5K5-2|UCHL5_HUMAN	0.0822773	0.09894868
sp|P23946|CMA1_HUMAN	0.08283615	0.84879977
sp|Q66K74-2|MAP1S_HUMAN	0.08321667	0
sp|Q9BZF9-2|UACA_HUMAN	0.0839529	0.19149946
sp|Q9GZT8|NIF3L_HUMAN	0.08515739	0.45033538
sp|Q92599-3|SEPT8_HUMAN	0.0861454	0.19149946
sp|Q15942|ZYX_HUMAN	0.08640862	0.95332193
sp|P09012|SNRPA_HUMAN	0.08688736	0
sp|P78417-3|GSTO1_HUMAN	0.08784676	0
sp|Q00577|PURA_HUMAN	0.08823776	0.33495146
sp|Q06033-2|ITIH3_HUMAN	0.08884239	0.19149946
sp|P04004|VTNC_HUMAN	0.08952713	0.7498006
sp|P0DOX7|IGK_HUMAN	0.09157562	0.30372584
sp|O60256-3|KPRB_HUMAN	0.0942955	0.45033538
sp|P12111-4|CO6A3_HUMAN	0.09584618	0.656254
BirA-TRIP6_BirAT6	0.10104275	0.19149946
sp|P02747|C1QC_HUMAN	0.10310364	1.1932944
sp|O15061|SYNEM_HUMAN	0.10358143	0.33495146
sp|Q96PD5-2|PGRP2_HUMAN	0.10424805	0.5111962
sp|Q96P70|IPO9_HUMAN	0.10457993	0.21439649
sp|O43301|HS12A_HUMAN	0.1061573	0.6298893
sp|Q6NZI2|CAVN1_HUMAN	0.10638619	0.8654178
sp|Q86VS8|HOOK3_HUMAN	0.10852432	0.19149946
sp|P35579-2|MYH9_HUMAN	0.10903454	0.656254
sp|Q7Z7G0|TARSH_HUMAN	0.10959625	0
sp|P01743|HV146_HUMAN	0.10971069	0.656254
sp|P60891|PRPS1_HUMAN	0.1108284	0
sp|P63267|ACTH_HUMAN	0.11135864	0.656254
sp|Q96D15|RCN3_HUMAN	0.11221886	0.35795313
sp|Q92905|CSN5_HUMAN	0.11315441	0.7827403
sp|P04196|HRG_HUMAN	0.11454582	0.81595695
sp|Q9BXN1|ASPN_HUMAN	0.11816406	1.3006523
sp|P21589-2|5NTD_HUMAN	0.12126923	0.19149946
sp|P07996|TSP1_HUMAN	0.12399292	0.09894868
sp|P13671|CO6_HUMAN	0.12417603	0.09894868

sp|Q9H008|LHPP_HUMAN	0.12436867	0.39873424
sp|P62829|RL23_HUMAN	0.12603188	0
sp|P55058|PLTP_HUMAN	0.12862301	1.1932944
sp|Q9NYL4|FKB11_HUMAN	0.12922859	0
sp|Q27J81-2|INF2_HUMAN	0.1299839	1.1505735
sp|Q9NY15|STAB1_HUMAN	0.13059616	0
sp|P22061-2|PIMT_HUMAN	0.13645935	0.8983557
sp|Q96HY6|DDRGK_HUMAN	0.14018154	0.656254
sp|Q15102|PA1B3_HUMAN	0.14105797	0.7061832
sp|O00233-2|PSMD9_HUMAN	0.1420803	0.19149946
sp|P0DOY3|IGLC3_HUMAN	0.14329529	0.6298893
sp|P0C0S5|H2AZ_HUMAN	0.14498901	0.656254
sp|Q16647|PTGIS_HUMAN	0.1483326	0.70235044
sp|Q9Y315|DEOC_HUMAN	0.15008163	0.1342476
sp|O76074-2|PDE5A_HUMAN	0.15588188	0.91601294
sp|Q05315|LEG10_HUMAN	0.15616798	0
sp|P16403|H12_HUMAN	0.16007233	0
sp|P63027|VAMP2_HUMAN	0.16201973	1.1932944
sp|Q15286|RAB35_HUMAN	0.16278172	0.656254
sp|P62269|RS18_HUMAN	0.16395378	0.21439649
sp|P06132|DCUP_HUMAN	0.16711426	0.6298893
sp|Q30154|DRB5_HUMAN	0.16824341	0
sp|Q9GZP4-2|PITH1_HUMAN	0.16915894	0.7827403
sp|P00740|FA9_HUMAN	0.16967773	0.312067
sp|Q9UNH7-2|SNX6_HUMAN	0.17202187	0.19149946
sp|P12814-2|ACTN1_HUMAN	0.17351723	1.1932944
sp|O95810|CAVN2_HUMAN	0.17352104	1.2392054
sp|Q15149-9|PLEC_HUMAN	0.17720222	0.656254
sp|P23142|FBLN1_HUMAN	0.18018341	0.39873424
sp|Q5TZA2|CROCC_HUMAN	0.18378448	1.0485198
sp|Q16775-2|GLO2_HUMAN	0.1855011	0.91601294
sp|P09493-5|TPM1_HUMAN	0.18725967	1.1932944
sp|P00748|FA12_HUMAN	0.19519806	0.7061832
sp|P02461|CO3A1_HUMAN	0.19569016	0.7827403
sp|Q04695|K1C17_HUMAN	0.19782448	0.656254
sp|P34896-2|GLYC_HUMAN	0.19935036	0
sp|P18084|ITB5_HUMAN	0.20252228	1.1932944
sp|Q99733-2|NP1L4_HUMAN	0.20755005	0.7498006
sp|P06753-6|TPM3_HUMAN	0.20763779	0.656254
sp|Q15404|RSU1_HUMAN	0.21027946	0.656254
sp|Q96HC4|PDLI5_HUMAN	0.21030617	0.656254
sp|Q9Y4G6|TLN2_HUMAN	0.21848106	0.656254
sp|P16144-2|ITB4_HUMAN	0.21891594	1.1932944
sp|Q687X5|STEA4_HUMAN	0.21991348	0.19149946
sp|P47895|AL1A3_HUMAN	0.22136593	0.656254

sp|Q9Y281|COF2_HUMAN	0.22808647	1.1505735
sp|P14207|FOLR2_HUMAN	0.23295403	0.656254
sp|Q14515-2|SPRL1_HUMAN	0.23765755	0
sp|P05452|TETN_HUMAN	0.24341965	0.91601294
sp|P20810-4|ICAL_HUMAN	0.24577713	0.91601294
sp|Q15323|K1H1_HUMAN	0.2505741	0.656254
sp|P42285|MTREX_HUMAN	0.25122833	0.7827403
sp|P0DOX2|IGA2_HUMAN	0.2531147	1.1505735
sp|P15088|CBPA3_HUMAN	0.2545681	1.0485198
sp|P01911|2B1F_HUMAN	0.25611305	1.1932944
sp|P11171-7|41_HUMAN	0.25737	0.8983557
sp|O75746-2|CMC1_HUMAN	0.25826645	0.2178309
sp|P07951-3|TPM2_HUMAN	0.2615013	0.7588735
sp|A0A0C4DH25|KVD20_HUMA	0.26438904	0.656254
sp|P36542|ATPG_HUMAN	0.26590538	0.656254
sp|P0C0L4|CO4A_HUMAN	0.27246475	1.1932944
sp|P84243|H33_HUMAN	0.2766781	0
sp|A0A0C4DH29|HV103_HUMA	0.2800274	0.656254
sp|Q9H6S3|ES8L2_HUMAN	0.29068756	0.7827403
sp|Q8TAQ2-2|SMRC2_HUMAN	0.2915039	1.0301651
sp|O94979-10|SC31A_HUMAN	0.2937975	0.7061832
sp|P48741|HSP77_HUMAN	0.30083084	0.656254
sp|P02765|FETUA_HUMAN	0.3017311	1.0076748
sp|P31321|KAP1_HUMAN	0.3094635	0.656254
sp|Q13976-2|KGP1_HUMAN	0.31010056	0.656254
sp|P01834|IGKC_HUMAN	0.31629562	1.1932944
sp|O94919|ENDD1_HUMAN	0.31834412	0.19149946
sp|P35542|SAA4_HUMAN	0.32185364	1.1932944
sp|P30046|DOPD_HUMAN	0.32326698	1.1932944
sp|Q8TCD5|NT5C_HUMAN	0.3387184	1.1932944
sp|P30711|GSTT1_HUMAN	0.34223175	0.7061832
sp|P02753|RET4_HUMAN	0.34241295	0.5204253
sp|Q9GZM7-3|TINAL_HUMAN	0.34241867	1.1932944
sp|P52758|RIDA_HUMAN	0.361372	1.1932944
sp|Q9NSK0-5|KLC4_HUMAN	0.37992477	0.656254
sp|P16104|H2AX_HUMAN	0.43000126	0
sp|Q9Y5Z4|HEBP2_HUMAN	0.45191765	1.2773042
sp|Q15437|SC23B_HUMAN	0.49086094	0.656254
sp|P08185|CBG_HUMAN	0.5303974	1.1932944
sp|P69892|HBG2_HUMAN	0.5413246	0.656254
sp|Q05682|CALD1_HUMAN	0.55947685	0.5204253
sp|O76011|KRT34_HUMAN	0.5604229	0.656254
sp|O94788-3|AL1A2_HUMAN	0.5710945	0.656254
sp|P28161|GSTM2_HUMAN	0.57998276	0.656254
sp|A0A0C4DH41|HV461_HUMA	0.5948429	0.656254

sp|P08779|K1C16_HUMAN	0.6133766	1.1932944
sp|P0C0L5|CO4B_HUMAN	0.6143017	1.1932944
sp|P05155-2|IC1_HUMAN	0.66033936	1.1932944
sp|P30613-2|KPYR_HUMAN	0.6740303	0.656254
sp|P02533|K1C14_HUMAN	0.7000294	1.1932944
sp|Q9BTE3-2|MCMBP_HUMAN	0.79364014	0.19149946
sp|Q03591|FHR1_HUMAN	0.8157749	1.1932944
sp|A0A075B6P5|KV228_HUMA	0.8710098	0.656254
sp|P23083|HV102_HUMAN	1.1148872	0.656254
sp|P11166|GTR1_HUMAN	1.2738323	1.1932944
sp|P00738|HPT_HUMAN	1.6296806	0.656254
sp|P20039|2B1B_HUMAN	1.7304668	0.656254
