# Supplementary material for: Peptidylarginine deiminase 2 citrullinates MZB1 and promotes the secretion of IgM and IgA
Source: Front Immunol. 2023 Nov 29;14:1290585. doi: 10.3389/fimmu.2023.1290585 (PMC10716219; doi:10.3389/fimmu.2023.1290585)
Supplement: Supplementary file 12 [file DataSheet_12.pdf]

Supplemental Table 12: COPD3 vs controls

Accession #	Fold Change	p value (-log10)
sp|Q15109-10|RAGE_HUMAN	-1.4141979	2.1818786
sp|O96009|NAPSA_HUMAN	-1.106142	2.6576471
sp|P22748|CAH4_HUMAN	-1.0754547	2.6576471
sp|Q13510-2|ASAH1_HUMAN	-1.0377159	6.3204336
sp|P07339|CATD_HUMAN	-0.9732132	8.061325
sp|P31949|S10AB_HUMAN	-0.9386196	2.6576471
sp|P99999|CYC_HUMAN	-0.9363937	2.1818786
sp|P49407-2|ARRB1_HUMAN	-0.9130364	2.6576471
sp|Q10589-2|BST2_HUMAN	-0.9047298	1.6960104
sp|Q9NZA1-2|CLIC5_HUMAN	-0.9016819	3.342776
sp|Q01469|FABP5_HUMAN	-0.8719826	4.051346
sp|P05141|ADT2_HUMAN	-0.8659573	2.6576471
sp|P05362|ICAM1_HUMAN	-0.8574009	4.247887
sp|P12429|ANXA3_HUMAN	-0.8515568	7.5567927
sp|O43760-2|SNG2_HUMAN	-0.8480167	1.6960104
sp|P51659|DHB4_HUMAN	-0.8306885	12.110734
sp|P26440|IVD_HUMAN	-0.8076057	1.6960104
sp|Q86VB7-2|C163A_HUMAN	-0.8066177	8.558914
sp|P21397|AOFA_HUMAN	-0.7923279	4.628043
sp|O95810|CAVN2_HUMAN	-0.7669582	3.8337784
sp|P13686|PPA5_HUMAN	-0.7614098	1.6960104
sp|P50895|BCAM_HUMAN	-0.7367744	5.0779786
sp|P35241|RADI_HUMAN	-0.7350178	3.5908275
sp|P10253|LYAG_HUMAN	-0.7302055	3.1266599
sp|O00159-3|MYO1C_HUMAN	-0.7290688	6.7638435
sp|P51149|RAB7A_HUMAN	-0.7286568	3.4366283
sp|P62805|H4_HUMAN	-0.71978	6.253077
sp|P08729|K2C7_HUMAN	-0.7193699	14.374806
sp|P48735-2|IDHP_HUMAN	-0.7140312	4.051346
sp|Q15599-2|NHRF2_HUMAN	-0.7123432	2.6576471
sp|P05091|ALDH2_HUMAN	-0.70541	8.769468
sp|P55290-4|CAD13_HUMAN	-0.7041817	1.9647322
sp|Q8WWI1-5|LMO7_HUMAN	-0.6997643	2.0265307
sp|P84103-2|SRSF3_HUMAN	-0.6891975	2.5644996
sp|Q6PIU2-2|NCEH1_HUMAN	-0.6850281	1.7165122
sp|Q16698-2|DECR_HUMAN	-0.6815624	4.509013
sp|P05026-2|AT1B1_HUMAN	-0.6812954	2.6576471
sp|P07686|HEXB_HUMAN	-0.6776495	1.827201
sp|P04179-4|SODM_HUMAN	-0.6766319	3.1266599
sp|P31947|1433S_HUMAN	-0.6718979	2.1818786
sp|Q9UPQ0|LIMC1_HUMAN	-0.6666889	2.2323174
sp|P09758|TACD2_HUMAN	-0.6619911	1.7590232
sp|Q96TA1-2|NIBL1_HUMAN	-0.6577568	2.653196

sp|P56199|ITA1_HUMAN	-0.6498127	6.3204336
sp|Q9Y624|JAM1_HUMAN	-0.6452408	2.6576471
sp|P01903|DRA_HUMAN	-0.6436195	2.1818786
sp|P84090|ERH_HUMAN	-0.6419334	2.1818786
sp|P01833|PIGR_HUMAN	-0.6410427	4.964395
sp|P43304|GPDM_HUMAN	-0.635685	3.4656596
sp|P05023|AT1A1_HUMAN	-0.628767	8.032379
sp|P33121-3|ACSL1_HUMAN	-0.6273994	2.1818786
sp|P00167-2|CYB5_HUMAN	-0.6261482	1.5646582
sp|Q13740-2|CD166_HUMAN	-0.6227875	4.746823
sp|P09467|F16P1_HUMAN	-0.6219273	3.9403465
sp|P09668|CATH_HUMAN	-0.6212788	3.218121
sp|Q00325-2|MPCP_HUMAN	-0.618	3.1266599
sp|Q00765|REEP5_HUMAN	-0.6165772	3.1266599
sp|P05556|ITB1_HUMAN	-0.6160488	10.140124
sp|Q99715-4|COCA1_HUMAN	-0.6122818	1.6960104
sp|O15143|ARC1B_HUMAN	-0.610508	2.702259
sp|P51648-2|AL3A2_HUMAN	-0.6080132	2.3979244
sp|Q6NZI2|CAVN1_HUMAN	-0.6068497	4.3492994
sp|Q9ULV4-3|COR1C_HUMAN	-0.605093	2.7624686
sp|P40121|CAPG_HUMAN	-0.5990295	4.42992
sp|P07858|CATB_HUMAN	-0.5956535	2.6182911
sp|P26038|MOES_HUMAN	-0.5891743	14.4105215
sp|O43707|ACTN4_HUMAN	-0.5766239	14.808462
sp|P13073|COX41_HUMAN	-0.5723743	1.6960104
sp|O00743-3|PPP6_HUMAN	-0.57164	1.6960104
sp|P02786|TFR1_HUMAN	-0.5712967	3.218121
sp|Q02218-2|ODO1_HUMAN	-0.5710468	3.8337784
sp|P09960|LKHA4_HUMAN	-0.5640221	9.441114
sp|Q07157|ZO1_HUMAN	-0.5634232	4.964395
sp|P07988|PSPB_HUMAN	-0.5596371	1.6832623
sp|Q03135|CAV1_HUMAN	-0.5526619	2.1818786
sp|Q9UHG3|PCYOX_HUMAN	-0.5487995	3.7091193
sp|P29401-2|TKT_HUMAN	-0.5477924	11.596198
sp|P23381|SYWC_HUMAN	-0.5452175	5.0895834
sp|P22307-8|NLTP_HUMAN	-0.5438938	1.4104178
sp|Q9NZN4|EHD2_HUMAN	-0.5437985	6.3876214
sp|Q96TC7|RMD3_HUMAN	-0.5403976	1.6960104
sp|P40926|MDHM_HUMAN	-0.5403309	8.112776
sp|P31040|SDHA_HUMAN	-0.5402603	3.261748
sp|P07099|HYEP_HUMAN	-0.5341473	7.128238
sp|P00505|AATM_HUMAN	-0.5304089	4.1861625
sp|P09622|DLDH_HUMAN	-0.5302544	3.8058946
sp|P43034|LIS1_HUMAN	-0.5299797	3.1266599
sp|Q8N335|GPD1L_HUMAN	-0.5298767	1.6960104

sp|P13796|PLSL_HUMAN	-0.5291939	10.720789
sp|Q9UBQ0-2|VPS29_HUMAN	-0.5287685	1.6960104
sp|P51649-2|SSDH_HUMAN	-0.5269394	2.1818786
sp|Q08722-2|CD47_HUMAN	-0.5264111	1.6960104
sp|O95994|AGR2_HUMAN	-0.5262947	2.6576471
sp|O75390|CISY_HUMAN	-0.5259533	4.146542
sp|P13804|ETFA_HUMAN	-0.5241909	4.509013
sp|Q9NNW7|TRXR2_HUMAN	-0.524086	2.1818786
sp|P25774|CATS_HUMAN	-0.5237942	1.6960104
sp|P63092-3|GNAS2_HUMAN	-0.5222893	2.9310203
sp|Q7L1Q6-2|BZW1_HUMAN	-0.5186758	1.6960104
sp|P17844-2|DDX5_HUMAN	-0.5146923	3.9417877
sp|O75874|IDHC_HUMAN	-0.5132313	3.734273
sp|Q9H0U4|RAB1B_HUMAN	-0.510788	1.6960104
sp|P40429|RL13A_HUMAN	-0.5087051	1.6960104
sp|P04080|CYTB_HUMAN	-0.5070572	1.6960104
sp|P13797|PLST_HUMAN	-0.5062466	4.156673
sp|P62906|RL10A_HUMAN	-0.4941425	1.827201
sp|Q8NBX0|SCPDL_HUMAN	-0.4940586	1.6960104
sp|P11310-2|ACADM_HUMAN	-0.4881535	4.051346
sp|Q6NUK1-2|SCMC1_HUMAN	-0.4871655	1.8444856
sp|Q02252-2|MMSA_HUMAN	-0.4828453	3.1208909
sp|Q9HC35-2|EMAL4_HUMAN	-0.4827747	1.8444856
sp|P22897|MRC1_HUMAN	-0.482605	3.8108692
sp|Q02543|RL18A_HUMAN	-0.4825554	2.6997027
sp|Q04917|1433F_HUMAN	-0.4804077	5.417897
sp|P22626|ROA2_HUMAN	-0.4785652	6.423805
sp|P61158|ARP3_HUMAN	-0.4745693	8.221851
sp|P17655|CAN2_HUMAN	-0.4729996	5.6984367
sp|O95340-2|PAPS2_HUMAN	-0.4703827	1.6604291
sp|P23141-2|EST1_HUMAN	-0.4698525	8.810719
sp|P61247|RS3A_HUMAN	-0.468914	4.051346
sp|P37802|TAGL2_HUMAN	-0.466404	5.633054
sp|O60814|H2B1K_HUMAN	-0.4645672	1.4104178
sp|Q16836-3|HCDH_HUMAN	-0.4640579	1.6960104
sp|P50225|ST1A1_HUMAN	-0.4632721	1.6960104
sp|Q9P0L0-2|VAPA_HUMAN	-0.4624081	2.8384566
sp|P35237|SPB6_HUMAN	-0.462347	5.2139587
sp|P10515|ODP2_HUMAN	-0.4587879	2.56956
sp|Q14118|DAG1_HUMAN	-0.458374	1.4104178
sp|O94760|DDAH1_HUMAN	-0.4569397	1.9647322
sp|P43490|NAMPT_HUMAN	-0.456913	5.0779786
sp|P10768|ESTD_HUMAN	-0.4554844	2.6576471
sp|Q8NF37|PCAT1_HUMAN	-0.4546433	1.6997428
sp|P12821-2|ACE_HUMAN	-0.4530335	1.6997428

sp|O75695|XRP2_HUMAN	-0.4510288	1.4104178
sp|P14543-2|NID1_HUMAN	-0.4490452	3.4751866
sp|P21796|VDAC1_HUMAN	-0.4466591	3.2217336
sp|P49591|SYSC_HUMAN	-0.4417076	3.760603
sp|Q969X5-2|ERGI1_HUMAN	-0.4409332	1.4104178
sp|P14866-2|HNRPL_HUMAN	-0.438591	1.6960104
sp|P62873|GBB1_HUMAN	-0.4384556	3.4656596
sp|P61106|RAB14_HUMAN	-0.4373875	3.4366283
sp|P60903|S10AA_HUMAN	-0.4373283	1.6960104
sp|P09525|ANXA4_HUMAN	-0.4346237	5.118518
sp|O60437|PEPL_HUMAN	-0.4342728	11.381452
sp|O43837|IDH3B_HUMAN	-0.4324913	1.6960104
sp|P62263|RS14_HUMAN	-0.430748	1.4104178
sp|P21964-2|COMT_HUMAN	-0.4303017	1.3957126
sp|Q9UHB6-4|LIMA1_HUMAN	-0.429596	2.1818786
sp|Q92597|NDRG1_HUMAN	-0.4286346	1.5646582
sp|P62917|RL8_HUMAN	-0.427124	2.6576471
sp|Q07955-3|SRSF1_HUMAN	-0.4267998	3.0819354
sp|P07237|PDIA1_HUMAN	-0.4267731	12.9745865
sp|P36957|ODO2_HUMAN	-0.4228401	2.923088
sp|Q9Y6N5|SQOR_HUMAN	-0.4215565	2.1242583
sp|O43813|LANC1_HUMAN	-0.4199143	1.6960104
sp|P23246|SFPQ_HUMAN	-0.4194698	4.7039127
sp|Q00839|HNRPU_HUMAN	-0.4187527	2.4140475
sp|P38646|GRP75_HUMAN	-0.4168167	5.6107674
sp|P11413-2|G6PD_HUMAN	-0.4144898	2.400925
sp|Q13451|FKBP5_HUMAN	-0.4144287	1.4936475
sp|P00352|AL1A1_HUMAN	-0.4138145	8.810719
sp|P10301|RRAS_HUMAN	-0.4134769	1.6960104
sp|Q15907-2|RB11B_HUMAN	-0.4114265	2.8739974
sp|P53597|SUCA_HUMAN	-0.4098396	1.6960104
sp|Q07020-2|RL18_HUMAN	-0.4096298	1.6960104
sp|Q13011|ECH1_HUMAN	-0.4082127	2.1377194
sp|P49756|RBM25_HUMAN	-0.4060268	1.6960104
sp|O60271-4|JIP4_HUMAN	-0.4059143	1.6960104
sp|P60981|DEST_HUMAN	-0.4057045	2.6576471
sp|Q16543|CDC37_HUMAN	-0.4051285	4.12427
sp|P61421|VA0D1_HUMAN	-0.4046326	1.8595492
sp|Q96KP4|CNDP2_HUMAN	-0.4029961	7.086946
sp|P38606|VATA_HUMAN	-0.4022875	1.8842831
sp|P12081-4|SYHC_HUMAN	-0.400734	1.4104178
sp|P62937|PPIA_HUMAN	-0.4000149	1.6960104
sp|P42330|AK1C3_HUMAN	-0.3999128	2.653196
sp|P19338|NUCL_HUMAN	-0.3998947	7.9589314
sp|P42765|THIM_HUMAN	-0.3996773	3.3179681

sp|P35268|RL22_HUMAN	-0.3996048	1.6960104
sp|P08758|ANXA5_HUMAN	-0.3984489	7.916384
sp|P11021|BIP_HUMAN	-0.3973351	12.99033
sp|Q9NTX5-6|ECHD1_HUMAN	-0.3956871	1.3080103
sp|P06733|ENOA_HUMAN	-0.3951111	8.338646
sp|Q16853|AOC3_HUMAN	-0.3948669	3.5553887
sp|Q9P2R7-2|SUCB1_HUMAN	-0.3948479	3.1266599
sp|P13639|EF2_HUMAN	-0.3923264	9.340597
sp|P18206-2|VINC_HUMAN	-0.3919258	15.65356
sp|P51572-2|BAP31_HUMAN	-0.389595	3.5908275
sp|P08575-10|PTPRC_HUMAN	-0.389101	2.838374
sp|P35222|CTNB1_HUMAN	-0.3890019	2.1931286
sp|P11766|ADHX_HUMAN	-0.3872643	3.354223
sp|P61604|CH10_HUMAN	-0.3866272	2.5644996
sp|P62826|RAN_HUMAN	-0.3832283	1.827201
sp|Q9ULA0|DNPEP_HUMAN	-0.3827381	4.964395
sp|P09211|GSTP1_HUMAN	-0.3822975	3.292093
sp|P30040|ERP29_HUMAN	-0.3822556	2.3047035
sp|P26641|EF1G_HUMAN	-0.3763523	4.372737
sp|O75083|WDR1_HUMAN	-0.3751755	8.889428
sp|Q15366-2|PCBP2_HUMAN	-0.3750629	1.6960104
sp|Q9UMS4|PRP19_HUMAN	-0.3746586	2.3047035
sp|P53007|TXTP_HUMAN	-0.3735085	2.6576471
sp|P10155|RO60_HUMAN	-0.3705559	2.0727112
sp|P55268|LAMB2_HUMAN	-0.3691664	5.8614116
sp|P35221|CTNA1_HUMAN	-0.3691502	5.605573
sp|P27797|CALR_HUMAN	-0.3689289	4.9577103
sp|Q9H4M9|EHD1_HUMAN	-0.3685474	2.838374
sp|P15311|EZRI_HUMAN	-0.3638468	3.2214048
sp|P24752|THIL_HUMAN	-0.3610058	2.9455793
sp|P11047|LAMC1_HUMAN	-0.3607483	5.765215
sp|Q13217|DNJC3_HUMAN	-0.3601685	2.1818786
sp|P60174|TPIS_HUMAN	-0.3586569	6.8254647
sp|P23634-8|AT2B4_HUMAN	-0.3582382	2.3388627
sp|Q9H8H3|MET7A_HUMAN	-0.3579292	1.4104178
sp|P62993|GRB2_HUMAN	-0.3576641	2.3047035
sp|P61981|1433G_HUMAN	-0.355402	1.8992519
sp|P50995-2|ANX11_HUMAN	-0.3550072	2.8438601
sp|Q9UFN0|NPS3A_HUMAN	-0.3546867	1.6960104
sp|P11177-3|ODPB_HUMAN	-0.3546486	1.5983955
sp|Q13228-4|SBP1_HUMAN	-0.3530216	9.64541
sp|Q13425|SNTB2_HUMAN	-0.3525219	1.7590232
sp|P40939|ECHA_HUMAN	-0.3514633	6.1994724
sp|P62249|RS16_HUMAN	-0.3507271	1.6960104
sp|Q96CW1-2|AP2M1_HUMAN	-0.3501797	2.1818786

sp|O15144|ARPC2_HUMAN	-0.3501473	1.6092666
sp|P49189|AL9A1_HUMAN	-0.3483887	6.096973
sp|P16284-3|PECA1_HUMAN	-0.3472538	2.3798928
sp|P08648|ITA5_HUMAN	-0.3466339	1.6960104
sp|P29590|PML_HUMAN	-0.3449974	3.2608445
sp|Q02878|RL6_HUMAN	-0.3436966	1.6960104
sp|P62258|1433E_HUMAN	-0.3435192	2.1818786
sp|P61978-3|HNRPK_HUMAN	-0.3430767	5.2234774
sp|P07355-2|ANXA2_HUMAN	-0.3416996	2.2270947
sp|P22695|QCR2_HUMAN	-0.3407555	2.3143692
sp|P38919|IF4A3_HUMAN	-0.3405895	1.8595492
sp|P35914|HMGCL_HUMAN	-0.3403778	2.6576471
sp|P49327|FAS_HUMAN	-0.3397808	5.7321286
sp|Q16762|THTR_HUMAN	-0.3387032	1.3815327
sp|P17931|LEG3_HUMAN	-0.3384609	2.6576471
sp|P60660-2|MYL6_HUMAN	-0.3376617	3.760603
sp|Q9HCC0|MCCB_HUMAN	-0.3355637	1.6092666
sp|Q6YHK3|CD109_HUMAN	-0.3353119	3.0019732
sp|P11940-2|PABP1_HUMAN	-0.3347759	1.6960104
sp|P00367|DHE3_HUMAN	-0.3335419	2.1952481
sp|P01011|AACT_HUMAN	-0.3299103	8.143158
sp|Q9BUJ2-4|HNRL1_HUMAN	-0.32967	2.173994
sp|P02743|SAMP_HUMAN	-0.3293667	1.7451575
sp|Q16555|DPYL2_HUMAN	-0.3275585	6.5985336
sp|Q02978-2|M2OM_HUMAN	-0.3275356	1.4104178
sp|Q96AG4|LRC59_HUMAN	-0.3246555	2.6576471
sp|Q92945|FUBP2_HUMAN	-0.3238716	3.07285
sp|P40306|PSB10_HUMAN	-0.3236408	2.1818786
sp|Q16363-2|LAMA4_HUMAN	-0.3216476	3.3700237
sp|P55084|ECHB_HUMAN	-0.3216076	3.1629772
sp|P52272-2|HNRPM_HUMAN	-0.3206635	4.2269735
sp|P20700|LMNB1_HUMAN	-0.3193169	10.507279
sp|P59998|ARPC4_HUMAN	-0.3179474	1.9951487
sp|P46940|IQGA1_HUMAN	-0.3151856	9.950464
sp|P60900|PSA6_HUMAN	-0.3144016	3.7723744
sp|Q86UP2-4|KTN1_HUMAN	-0.314394	5.6827826
sp|P19367-2|HXK1_HUMAN	-0.3137093	2.6576467
sp|P63000-2|RAC1_HUMAN	-0.313591	1.4104178
sp|Q96I99|SUCB2_HUMAN	-0.3120613	2.845279
sp|P63010-2|AP2B1_HUMAN	-0.3107643	1.5054473
sp|Q1KMD3|HNRL2_HUMAN	-0.3107433	3.316053
sp|P37837|TALDO_HUMAN	-0.3098564	6.096973
sp|Q9BR76|COR1B_HUMAN	-0.3092041	1.9509854
sp|P62140|PP1B_HUMAN	-0.3084049	1.4104178
sp|Q16658|FSCN1_HUMAN	-0.3067951	2.3626237

sp|Q9HBL0|TENS1_HUMAN	-0.3053742	3.8255916
sp|P27348|1433T_HUMAN	-0.3048725	2.1952481
sp|P07900-2|HS90A_HUMAN	-0.3048668	5.2257013
sp|P0DP25|CALM3_HUMAN	-0.3040733	3.5908275
sp|Q6WCQ1-2|MPRIP_HUMAN	-0.3037033	2.2731557
sp|Q02318|CP27A_HUMAN	-0.3027687	1.4104178
sp|P51148-2|RAB5C_HUMAN	-0.3025932	1.6960104
sp|P55072|TERA_HUMAN	-0.3006897	9.246625
sp|P21980|TGM2_HUMAN	-0.2996407	9.255672
sp|P23284|PPIB_HUMAN	-0.299572	3.0819354
sp|P63104|1433Z_HUMAN	-0.299572	3.671968
sp|P14314-2|GLU2B_HUMAN	-0.2986012	2.5461748
sp|Q9Y2B0|CNPY2_HUMAN	-0.2985296	1.6960104
sp|P15121|ALDR_HUMAN	-0.2983913	2.2613342
sp|P07737|PROF1_HUMAN	-0.291647	4.28322
sp|Q16527|CSRP2_HUMAN	-0.2914505	1.6960104
sp|Q01082|SPTB2_HUMAN	-0.2910233	14.95459
sp|P49748-2|ACADV_HUMAN	-0.2908745	5.4615245
sp|P08559-2|ODPA_HUMAN	-0.2900124	2.4723146
sp|Q13045-2|FLII_HUMAN	-0.2889042	1.560883
sp|P36578|RL4_HUMAN	-0.2887592	2.2385595
sp|P17612|KAPCA_HUMAN	-0.2874966	1.3815327
sp|Q13347|EIF3I_HUMAN	-0.2862663	2.151766
sp|P10809|CH60_HUMAN	-0.2852936	4.6351686
sp|Q9NQC3|RTN4_HUMAN	-0.2852421	1.7590232
sp|P16615|AT2A2_HUMAN	-0.2850876	4.9138513
sp|Q14103-3|HNRPD_HUMAN	-0.2838879	2.0102134
sp|O14950|ML12B_HUMAN	-0.2833481	1.7590232
sp|P68104|EF1A1_HUMAN	-0.2833443	1.9509854
sp|Q9UBQ7|GRHPR_HUMAN	-0.2830772	1.5646582
sp|P11142|HSP7C_HUMAN	-0.2825947	4.518582
sp|P43121|MUC18_HUMAN	-0.2824955	2.2323174
sp|P21291|CSRP1_HUMAN	-0.2796478	4.7229815
sp|P30740|ILEU_HUMAN	-0.2786083	1.4351699
sp|P51991|ROA3_HUMAN	-0.2773218	1.3815327
sp|P08133|ANXA6_HUMAN	-0.276989	10.601593
sp|Q9HDC9|APMAP_HUMAN	-0.2757702	2.824564
sp|Q15084-2|PDIA6_HUMAN	-0.2737837	3.8108692
sp|Q12905|ILF2_HUMAN	-0.2722836	3.3283036
sp|P16152|CBR1_HUMAN	-0.2716675	1.7451575
sp|P07942|LAMB1_HUMAN	-0.2715702	3.6223958
sp|O00231-2|PSD11_HUMAN	-0.2704697	1.3815327
sp|Q9NSE4|SYIM_HUMAN	-0.270052	2.7472508
sp|P25398|RS12_HUMAN	-0.2692919	2.6576471
sp|P52565|GDIR1_HUMAN	-0.2687683	3.342776

sp|O00299|CLIC1_HUMAN	-0.2684479	1.9787219
sp|Q9NQR4|NIT2_HUMAN	-0.2671032	1.9190748
sp|Q7Z406|MYH14_HUMAN	-0.26684	4.737692
sp|Q9NZM1-6|MYOF_HUMAN	-0.2668304	2.735238
sp|Q14974|IMB1_HUMAN	-0.266592	2.6947098
sp|P60953|CDC42_HUMAN	-0.2651005	1.4050349
sp|O75348|VATG1_HUMAN	-0.2637367	1.4104178
sp|Q13308-6|PTK7_HUMAN	-0.2636814	2.1818786
sp|O14773|TPP1_HUMAN	-0.2635727	1.7590232
sp|P23396|RS3_HUMAN	-0.2635441	3.0873964
sp|Q16531|DDB1_HUMAN	-0.2634182	2.651758
sp|P14625|ENPL_HUMAN	-0.2629242	11.436339
sp|P05107|ITB2_HUMAN	-0.2628555	2.2613342
sp|Q8NBJ5|GT251_HUMAN	-0.2625942	1.6960104
sp|P07203|GPX1_HUMAN	-0.2624493	1.9049921
sp|P35232|PHB_HUMAN	-0.2611847	2.312627
sp|P25786-2|PSA1_HUMAN	-0.260376	4.1295223
sp|Q06830|PRDX1_HUMAN	-0.2603664	4.7229815
sp|Q01130-2|SRSF2_HUMAN	-0.2584381	1.6960104
sp|P06737-2|PYGL_HUMAN	-0.2583065	2.7989542
sp|O75369-2|FLNB_HUMAN	-0.2570648	8.935387
sp|Q9BXS5-2|AP1M1_HUMAN	-0.2568378	1.3815327
sp|Q14764|MVP_HUMAN	-0.2567692	4.293713
sp|P28482|MK01_HUMAN	-0.256609	1.9951487
sp|P21281|VATB2_HUMAN	-0.2566013	3.5190198
sp|P48444|COPD_HUMAN	-0.2564526	4.612768
sp|P0DMV9|HS71B_HUMAN	-0.2563839	4.09663
sp|P31150|GDIA_HUMAN	-0.2563267	2.4314263
sp|Q9BVC6|TM109_HUMAN	-0.2543831	1.4104178
sp|P18124|RL7_HUMAN	-0.254158	1.4278674
sp|P00390-2|GSHR_HUMAN	-0.2532482	1.8444856
sp|P08865|RSSA_HUMAN	-0.2513065	2.544769
sp|P07954-2|FUMH_HUMAN	-0.2505398	1.6997428
sp|Q14240-2|IF4A2_HUMAN	-0.2503185	1.5646582
sp|P04075|ALDOA_HUMAN	-0.2502461	5.2976747
sp|O14936-2|CSKP_HUMAN	-0.2495079	1.9951487
sp|Q99536|VAT1_HUMAN	-0.2488709	2.5929193
sp|P61626|LYSC_HUMAN	-0.2485333	2.4829872
sp|P13489|RINI_HUMAN	-0.2456799	3.0297134
sp|Q9Y277-2|VDAC3_HUMAN	-0.2446022	2.3388627
sp|P20340-2|RAB6A_HUMAN	-0.2439404	2.838374
sp|P30101|PDIA3_HUMAN	-0.2424202	6.3254604
sp|P29218|IMPA1_HUMAN	-0.2416573	2.1404836
sp|Q08211|DHX9_HUMAN	-0.2407131	3.2024403
sp|P60228|EIF3E_HUMAN	-0.2400837	1.4716977

sp|Q96AE4-2|FUBP1_HUMAN	-0.2388191	1.4564189
sp|O75643|U520_HUMAN	-0.2387848	1.6960104
sp|P98160|PGBM_HUMAN	-0.2387104	5.653564
sp|P05783|K1C18_HUMAN	-0.2384682	4.1315417
sp|P26447|S10A4_HUMAN	-0.2373047	2.6576471
sp|P60842|IF4A1_HUMAN	-0.2370186	2.6182911
sp|Q8IUX7|AEBP1_HUMAN	-0.2368107	1.4050349
sp|Q15233|NONO_HUMAN	-0.236002	1.8087089
sp|Q8WUM4|PDC6I_HUMAN	-0.2357693	2.2612653
sp|Q96QK1|VPS35_HUMAN	-0.2357578	1.4017582
sp|P00558|PGK1_HUMAN	-0.2354889	1.444889
sp|P61204|ARF3_HUMAN	-0.235096	1.560883
sp|Q9NPH2|INO1_HUMAN	-0.2335796	2.058973
sp|P28838-2|AMPL_HUMAN	-0.231741	5.311325
sp|P62987|RL40_HUMAN	-0.2306156	2.151766
sp|P06744|G6PI_HUMAN	-0.230381	4.4293685
sp|Q9Y678|COPG1_HUMAN	-0.2283783	3.707581
sp|P49411|EFTU_HUMAN	-0.2274551	2.9455793
sp|P08311|CATG_HUMAN	-0.2263641	1.4278674
sp|P52209-2|6PGD_HUMAN	-0.2253418	4.0155067
sp|O43390-2|HNRPR_HUMAN	-0.2236786	3.1092827
sp|P46777|RL5_HUMAN	-0.2236042	2.747965
sp|Q15393|SF3B3_HUMAN	-0.2233963	1.9413493
sp|P55263|ADK_HUMAN	-0.2228527	1.3815327
sp|P02750|A2GL_HUMAN	-0.2224617	1.7165122
sp|P46782|RS5_HUMAN	-0.2214947	2.3559585
sp|P63244|RACK1_HUMAN	-0.2214375	2.1697264
sp|Q16891-2|MIC60_HUMAN	-0.2203121	2.2613342
sp|Q9H223|EHD4_HUMAN	-0.2200756	1.4936475
sp|P02788|TRFL_HUMAN	-0.2193317	13.131768
sp|P09497-2|CLCB_HUMAN	-0.2186775	2.1818786
sp|P08727|K1C19_HUMAN	-0.2186298	5.389566
sp|Q07954|LRP1_HUMAN	-0.2178497	3.468374
sp|P24539|AT5F1_HUMAN	-0.2171001	2.1404836
sp|Q14011-2|CIRBP_HUMAN	-0.2152529	1.6960104
sp|P35606|COPB2_HUMAN	-0.2141247	3.2401628
sp|P53621-2|COPA_HUMAN	-0.209959	2.2338576
sp|Q9P258|RCC2_HUMAN	-0.2059956	1.7590232
sp|O75436|VP26A_HUMAN	-0.2052612	2.151766
sp|Q13557-12|KCC2D_HUMAN	-0.2042837	1.827201
sp|P13861|KAP2_HUMAN	-0.2042236	2.7220116
sp|P20618|PSB1_HUMAN	-0.2042122	3.2221727
sp|Q9Y3I0|RTCB_HUMAN	-0.2035656	2.2385595
sp|P49419-2|AL7A1_HUMAN	-0.2023106	2.0615394
sp|P39060-1|COIA1_HUMAN	-0.202282	1.9951487

sp|Q99798|ACON_HUMAN	-0.202282	3.2840328
sp|P49588-2|SYAC_HUMAN	-0.2021675	2.2047858
sp|P12956|XRCC6_HUMAN	-0.2009392	3.201616
sp|Q92841-3|DDX17_HUMAN	-0.2006531	1.5424668
sp|P09382|LEG1_HUMAN	-0.1997013	2.747965
sp|P47756-2|CAPZB_HUMAN	-0.1994686	3.2347682
sp|P02545|LMNA_HUMAN	-0.1988258	11.858376
sp|P16278-2|BGAL_HUMAN	-0.1985512	1.560883
sp|O00764-2|PDXK_HUMAN	-0.1982422	1.6092666
sp|P04632|CPNS1_HUMAN	-0.1963244	1.3481187
sp|Q9H4A4|AMPB_HUMAN	-0.1963158	1.7306389
sp|Q12906-4|ILF3_HUMAN	-0.1959648	4.40577
sp|P05198|IF2A_HUMAN	-0.1952934	1.9647322
sp|P50453|SPB9_HUMAN	-0.1931496	1.5567774
sp|P30153|2AAA_HUMAN	-0.1922112	2.0615394
sp|Q9Y4L1|HYOU1_HUMAN	-0.192193	1.3379446
sp|P14868|SYDC_HUMAN	-0.191143	3.500665
sp|P47755|CAZA2_HUMAN	-0.1908054	2.1092448
sp|P31146|COR1A_HUMAN	-0.1900578	1.7921138
sp|Q15746-2|MYLK_HUMAN	-0.1884232	2.2323174
sp|P11216|PYGB_HUMAN	-0.1872692	1.8557531
sp|P46939-2|UTRO_HUMAN	-0.1861668	3.3179681
sp|Q9UBG0|MRC2_HUMAN	-0.1857491	1.7265131
sp|P23526|SAHH_HUMAN	-0.183321	2.301498
sp|P06576|ATPB_HUMAN	-0.1830349	3.0979662
sp|Q16851|UGPA_HUMAN	-0.1814098	1.5942118
sp|Q99623|PHB2_HUMAN	-0.1809311	2.0751042
sp|Q15293|RCN1_HUMAN	-0.1804199	1.560883
sp|P49368|TCPG_HUMAN	-0.1790886	2.9122765
sp|Q9Y490|TLN1_HUMAN	-0.1779137	10.308417
sp|P05164-3|PERM_HUMAN	-0.1773911	2.3173661
sp|O75131|CPNE3_HUMAN	-0.1760063	1.3668759
sp|P13010|XRCC5_HUMAN	-0.1752396	1.6267194
sp|Q13263|TIF1B_HUMAN	-0.175024	1.3665526
sp|P18669|PGAM1_HUMAN	-0.1717377	1.3226568
sp|Q9UL46|PSME2_HUMAN	-0.1714401	1.560883
sp|Q6DD88|ATLA3_HUMAN	-0.1699066	1.3080103
sp|Q7Z4W1|DCXR_HUMAN	-0.1687336	1.7590232
sp|Q00610-2|CLH1_HUMAN	-0.1684742	2.9116788
sp|P32969|RL9_HUMAN	-0.1677589	1.6960104
sp|P27338|AOFB_HUMAN	-0.1676903	2.173994
sp|P20674|COX5A_HUMAN	-0.1673298	1.6960104
sp|P13667|PDIA4_HUMAN	-0.166502	4.6288357
sp|P08238|HS90B_HUMAN	-0.1657314	1.4126712
sp|Q9BSJ8-2|ESYT1_HUMAN	-0.1651135	2.3145256

sp|P35580-3|MYH10_HUMAN	-0.1637421	7.2392063
sp|Q15124|PGM5_HUMAN	-0.1631737	2.0178351
sp|O95831-3|AIFM1_HUMAN	-0.1623688	2.7597136
sp|O15230|LAMA5_HUMAN	-0.1616535	1.5194324
sp|P00325|ADH1B_HUMAN	-0.1613007	1.9951487
sp|Q03252|LMNB2_HUMAN	-0.1612873	3.879411
sp|P07384|CAN1_HUMAN	-0.157589	1.4567577
sp|O60749|SNX2_HUMAN	-0.1571293	2.312627
sp|P61081|UBC12_HUMAN	-0.1556816	2.3143692
sp|P52566|GDIR2_HUMAN	-0.1542931	1.5054473
sp|P04843|RPN1_HUMAN	-0.1499748	1.8976122
sp|P06756-3|ITAV_HUMAN	-0.1466827	1.4605758
sp|P12110|CO6A2_HUMAN	-0.1428623	2.1923351
sp|P01009|A1AT_HUMAN	-0.1416569	4.9720736
sp|P62241|RS8_HUMAN	-0.125906	1.9413493
sp|P62316-2|SMD2_HUMAN	-0.1218548	1.6960104
sp|Q15029-2|U5S1_HUMAN	-0.1191912	1.4068714
sp|P48637|GSHB_HUMAN	-0.1142464	2.0598862
sp|O15173-2|PGRC2_HUMAN	-0.0932884	1.3815327
sp|Q9P2E9|RRBP1_HUMAN	-0.0552254	1.6955315
sp|P17174|AATC_HUMAN	0.04894829	1.4068714
sp|P78371|TCPB_HUMAN	0.06207848	1.3607222
sp|P17661|DESM_HUMAN	0.09907341	4.1731977
sp|Q9UHL4|DPP2_HUMAN	0.10057259	1.4351699
sp|Q12805-2|FBLN3_HUMAN	0.11464691	1.800942
sp|P08670|VIME_HUMAN	0.11870575	9.746241
sp|Q9UNZ2-5|NSF1C_HUMAN	0.11901474	1.6832623
sp|P13611|CSPG2_HUMAN	0.12263107	1.3668759
sp|P04275|VWF_HUMAN	0.12509918	2.407128
sp|P13798|ACPH_HUMAN	0.1253128	1.7565529
sp|P28331-2|NDUS1_HUMAN	0.13256836	1.4278674
sp|P35555|FBN1_HUMAN	0.13557243	1.9765971
sp|P00568|KAD1_HUMAN	0.1444416	1.6046445
sp|P00450|CERU_HUMAN	0.14829063	4.670891
sp|P27105|STOM_HUMAN	0.1547718	3.2925725
sp|Q15661|TRYB1_HUMAN	0.15645218	3.0019732
sp|Q14315-2|FLNC_HUMAN	0.15871811	5.9378057
sp|P67936-2|TPM4_HUMAN	0.17012978	1.6960104
sp|P00751|CFAB_HUMAN	0.17462921	6.9483953
sp|P10643|CO7_HUMAN	0.18388367	1.3213577
sp|Q8N2S1|LTBP4_HUMAN	0.18825531	3.0253785
sp|Q16647|PTGIS_HUMAN	0.1989193	1.4278674
sp|P02790|HEMO_HUMAN	0.22452164	5.854947
sp|P04217|A1BG_HUMAN	0.2343216	1.4126712
sp|Q00341-2|VIGLN_HUMAN	0.23452187	1.4751658

sp|Q9UBX5|FBLN5_HUMAN	0.23580742	2.0598862
sp|Q8NBS9|TXND5_HUMAN	0.239151	5.0895834
sp|P01861|IGHG4_HUMAN	0.24751854	1.8595492
sp|P25311|ZA2G_HUMAN	0.24859619	2.4829872
sp|P07357|CO8A_HUMAN	0.25520897	1.8444856
sp|P35611-2|ADDA_HUMAN	0.25980377	2.3143692
sp|P02768|ALBU_HUMAN	0.2719326	3.3363256
sp|O60610-2|DIAP1_HUMAN	0.2740612	1.5646582
sp|P98095-2|FBLN2_HUMAN	0.27562714	2.9793715
sp|P01031|CO5_HUMAN	0.2762909	3.7727528
sp|P30041|PRDX6_HUMAN	0.27753067	3.5097537
sp|P36955|PEDF_HUMAN	0.28664207	3.0251918
sp|P22105-1|TENX_HUMAN	0.2888813	9.856805
sp|O00754-2|MA2B1_HUMAN	0.29742432	2.1818786
sp|P00748|FA12_HUMAN	0.30023003	1.4104178
sp|Q07507|DERM_HUMAN	0.30643463	3.103845
sp|P22392-2|NDKB_HUMAN	0.30796432	2.6576471
sp|P02765|FETUA_HUMAN	0.3093729	1.6997428
sp|P02649|APOE_HUMAN	0.31316948	1.8507931
sp|Q9UMS6-2|SYNP2_HUMAN	0.31889725	2.6576471
sp|Q99598|TSNAX_HUMAN	0.34841347	1.6960104
sp|O14558|HSPB6_HUMAN	0.36224556	1.560883
sp|Q05707|COEA1_HUMAN	0.36885834	9.764618
sp|P03952|KLKB1_HUMAN	0.36933136	2.1818786
sp|P01859|IGHG2_HUMAN	0.3821125	3.671968
sp|P39059|COFA1_HUMAN	0.38402176	2.1818786
sp|P02766|TTHY_HUMAN	0.38710403	2.1818786
sp|P49913|CAMP_HUMAN	0.40273285	1.6960104
sp|P19827|ITIH1_HUMAN	0.40452194	2.4970746
sp|P05156|CFAI_HUMAN	0.40534973	4.051346
sp|P01023|A2MG_HUMAN	0.40900993	15.25562
sp|P04003|C4BPA_HUMAN	0.41529465	4.5635147
sp|P06702|S10A9_HUMAN	0.41584396	2.1404836
sp|P07451|CAH3_HUMAN	0.4210472	1.8838416
sp|P02760|AMBP_HUMAN	0.4212799	2.441399
sp|P05452|TETN_HUMAN	0.4220066	1.6960104
sp|P23946|CMA1_HUMAN	0.42876434	2.4829872
sp|Q9Y315|DEOC_HUMAN	0.43627357	1.9647322
sp|O43301|HS12A_HUMAN	0.4588337	2.1818786
sp|P00734|THRB_HUMAN	0.45954704	6.423805
sp|P20810-4|ICAL_HUMAN	0.47285652	1.6960104
sp|P13671|CO6_HUMAN	0.47297668	1.6960104
sp|Q8WU39|MZB1_HUMAN	0.48945236	2.3047035
sp|P0DOX5|IGG1_HUMAN	0.49958038	3.887875
sp|P08603|CFAH_HUMAN	0.50346756	15.35253

sp|P07996|TSP1_HUMAN	0.5042496	1.6960104
sp|P04040|CATA_HUMAN	0.5069752	10.827353
sp|P19823|ITIH2_HUMAN	0.51641655	5.417897
sp|P04196|HRG_HUMAN	0.53082085	3.887875
sp|P09493-9|TPM1_HUMAN	0.53315735	1.6960104
sp|P02774-3|VTDB_HUMAN	0.5451584	8.958427
sp|P10909-5|CLUS_HUMAN	0.5483742	4.964395
sp|P29622|KAIN_HUMAN	0.5596771	2.6576471
sp|P43652|AFAM_HUMAN	0.5628624	4.509013
sp|P00492|HPRT_HUMAN	0.5665741	2.1818786
sp|Q9BXN1|ASPN_HUMAN	0.5900192	2.6576471
sp|P00491|PNPH_HUMAN	0.59103775	4.220149
sp|P0DOX8|IGL1_HUMAN	0.59856415	2.1818786
sp|P01876|IGHA1_HUMAN	0.6050339	4.509013
sp|P01008|ANT3_HUMAN	0.60894775	8.010044
sp|P36269-3|GGT5_HUMAN	0.61184216	3.1266599
sp|P11678|PERE_HUMAN	0.61528397	1.4104178
sp|P00747|PLMN_HUMAN	0.63069725	6.645139
sp|Q96D15|RCN3_HUMAN	0.6349659	1.6960104
sp|P04259|K2C6B_HUMAN	0.645277	2.1818786
sp|P01871-2|IGHM_HUMAN	0.69179153	2.1818786
sp|P51888|PRELP_HUMAN	0.694191	5.869831
sp|P13647|K2C5_HUMAN	0.69883347	6.7698927
sp|P35527|K1C9_HUMAN	0.70916176	5.5742316
sp|P13716-2|HEM2_HUMAN	0.720726	3.5908275
sp|P02749|APOH_HUMAN	0.72964096	2.817951
sp|P04114|APOB_HUMAN	0.73796463	12.475591
sp|Q15102|PA1B3_HUMAN	0.7380066	1.6960104
sp|P13645|K1C10_HUMAN	0.7405472	10.200565
sp|P06727|APOA4_HUMAN	0.7486286	6.3204336
sp|P35908|K22E_HUMAN	0.75609016	7.665951
sp|P01042-2|KNG1_HUMAN	0.7568741	7.2183566
sp|P0DOY3|IGLC3_HUMAN	0.8024597	2.1818786
sp|P11171-7|41_HUMAN	0.83636093	2.1818786
sp|Q9NYL4|FKB11_HUMAN	0.87648296	1.4104178
sp|P04264|K2C1_HUMAN	0.881197	12.996504
sp|P23456|Trypsin	0.90629387	3.5908275
sp|P08123|CO1A2_HUMAN	0.9159508	2.6576471
sp|P02679-2|FIBG_HUMAN	0.9273262	7.185697
sp|Q5TDH0-3|DDI2_HUMAN	0.9294262	1.6960104
sp|P02675|FIBB_HUMAN	0.9405899	10.337908
sp|P02452|CO1A1_HUMAN	0.9537945	2.1800864
sp|P11277-2|SPTB1_HUMAN	0.97397995	6.3204336
sp|P01860|IGHG3_HUMAN	0.9992695	2.6576471
sp|P15088|CBPA3_HUMAN	1.0240707	2.1818786

sp|P05546|HEP2_HUMAN	1.0361633	2.6576471
sp|P27169|PON1_HUMAN	1.0718412	1.6960104
sp|P02671|FIBA_HUMAN	1.0924168	11.667864
sp|P30043|BLVRB_HUMAN	1.1237984	2.1818786
sp|P16157-21|ANK1_HUMAN	1.1487083	4.051346
sp|P07738|PMGE_HUMAN	1.2384453	2.1818786
sp|P02549-2|SPTA1_HUMAN	1.243742	9.893875
sp|P51884|LUM_HUMAN	1.2929459	8.112776
sp|P02652-2|APOA2_HUMAN	1.3467789	3.1266599
sp|P02730|B3AT_HUMAN	1.4804726	6.7698927
sp|P02647|APOA1_HUMAN	1.5010452	13.420564
sp|P07585|PGS2_HUMAN	1.6383743	5.417897
sp|P00918|CAH2_HUMAN	1.6874275	4.509013
sp|P20774|MIME_HUMAN	1.7522964	4.051346
sp|P00915|CAH1_HUMAN	1.8656311	7.665951
sp|P32119|PRDX2_HUMAN	2.0975037	6.7698927
sp|P02042|HBD_HUMAN	2.2778378	4.051346
sp|P68871|HBB_HUMAN	2.6614857	4.051346
sp|P69905|HBA_HUMAN	2.8671894	4.051346
sp|P08263|GSTA1_HUMAN	-1.9158535	0.656254
sp|Q9BW04|SARG_HUMAN	-1.510603	1.1932944
sp|O00757|F16P2_HUMAN	-1.4904575	1.1932944
sp|P04229|2B11_HUMAN	-1.1787872	0.656254
sp|P08473|NEP_HUMAN	-1.1199198	0.5204253
sp|P30044-2|PRDX5_HUMAN	-1.0732689	0.656254
sp|Q9Y4G6|TLN2_HUMAN	-0.9276104	0.656254
sp|Q03113|GNA12_HUMAN	-0.9246178	0.656254
sp|Q9UBR2|CATZ_HUMAN	-0.8874226	1.1932944
sp|P51153|RAB13_HUMAN	-0.8838997	0.656254
sp|P28799-3|GRN_HUMAN	-0.8749914	0.7061832
sp|P63218|GBG5_HUMAN	-0.8671036	1.1932944
sp|Q8NC51-3|PAIRB_HUMAN	-0.8643608	1.1932944
sp|Q08209-5|PP2BA_HUMAN	-0.8634777	0
sp|Q96IJ6-2|GMPPA_HUMAN	-0.84762	1.1932944
sp|P12236|ADT3_HUMAN	-0.8409882	1.1932944
sp|P28330|ACADL_HUMAN	-0.8354359	1.1932944
sp|P38159|RBMX_HUMAN	-0.8292866	0.656254
sp|Q15056-2|IF4H_HUMAN	-0.8186607	1.1932944
sp|Q01518|CAP1_HUMAN	-0.8113909	0.656254
sp|Q03135-2|CAV1_HUMAN	-0.8040009	0.656254
sp|O00592-2|PODXL_HUMAN	-0.8033085	1.1932944
sp|P61601|NCALD_HUMAN	-0.7826338	0.656254
sp|Q9NUB1-2|ACS2L_HUMAN	-0.7726479	1.1932944
sp|Q16777|H2A2C_HUMAN	-0.7692757	1.1932944
sp|P51606-2|RENBP_HUMAN	-0.7625732	1.1932944

sp|P33151|CADH5_HUMAN	-0.7618408	1.1932944
sp|Q29963|1C06_HUMAN	-0.7586403	0.656254
sp|Q6DN03|H2B2C_HUMAN	-0.7406597	1.1932944
sp|Q86TX2|ACOT1_HUMAN	-0.7170544	0.656254
sp|O43795-2|MYO1B_HUMAN	-0.7159872	1.1932944
sp|Q12846|STX4_HUMAN	-0.7033958	0.656254
sp|P15170-2|ERF3A_HUMAN	-0.6948128	1.1932944
sp|Q8NBQ5|DHB11_HUMAN	-0.6936417	1.1932944
sp|P12830|CADH1_HUMAN	-0.6885319	0.7827403
sp|P02462|CO4A1_HUMAN	-0.6877041	0.5204253
sp|Q15286|RAB35_HUMAN	-0.685894	0.656254
sp|Q9UDY2-3|ZO2_HUMAN	-0.6794033	1.1932944
sp|P63167|DYL1_HUMAN	-0.6769695	0.656254
sp|Q9NVJ2|ARL8B_HUMAN	-0.6763039	1.1932944
sp|Q13813-3|SPTN1_HUMAN	-0.6742859	0
sp|Q15185-3|TEBP_HUMAN	-0.6742306	1.1932944
sp|Q01955|CO4A3_HUMAN	-0.6620178	1.1932944
sp|Q13976|KGP1_HUMAN	-0.6571674	0.656254
sp|P08962-2|CD63_HUMAN	-0.6539497	1.1932944
sp|P37235|HPCL1_HUMAN	-0.6437607	0.656254
sp|Q96FV2-2|SCRN2_HUMAN	-0.6401863	1.1932944
sp|P62834|RAP1A_HUMAN	-0.6399832	0.656254
sp|P20702|ITAX_HUMAN	-0.6384506	0.656254
sp|Q9NPY3|C1QR1_HUMAN	-0.631958	1.1932944
sp|P48509|CD151_HUMAN	-0.6298847	1.1932944
sp|P68371|TBB4B_HUMAN	-0.627739	1.1932944
sp|Q13283|G3BP1_HUMAN	-0.6268005	1.1932944
sp|P16422|EPCAM_HUMAN	-0.6067858	1.1932944
sp|O95837|GNA14_HUMAN	-0.6005173	0.656254
sp|Q93077|H2A1C_HUMAN	-0.598856	0.7827403
sp|P61586|RHOA_HUMAN	-0.5983906	1.1932944
sp|Q9Y394-2|DHRS7_HUMAN	-0.587759	0.7588735
sp|Q9H2U2-3|IPYR2_HUMAN	-0.5859108	0.656254
sp|Q14956-2|GPNMB_HUMAN	-0.5780182	1.1932944
sp|P36871|PGM1_HUMAN	-0.5719013	1.1932944
sp|A6NMY6|AXA2L_HUMAN	-0.5674095	0.656254
sp|O00186|STXB3_HUMAN	-0.5588388	1.1932944
sp|Q08431|MFGM_HUMAN	-0.547205	0.35795313
sp|Q9UHN6-2|CEIP2_HUMAN	-0.5470066	1.1932944
sp|P61916-2|NPC2_HUMAN	-0.5378094	1.1932944
sp|P04839|CY24B_HUMAN	-0.5319605	1.1932944
sp|Q7L576|CYFP1_HUMAN	-0.5296974	1.1932944
sp|Q07075|AMPE_HUMAN	-0.528223	1.0485198
sp|P06396-2|GELS_HUMAN	-0.5246544	0.656254
sp|Q32MZ4-3|LRRF1_HUMAN	-0.5228329	0.7827403

sp|Q7Z4I7-3|LIMS2_HUMAN	-0.5184174	1.1932944
sp|Q13884|SNTB1_HUMAN	-0.5163918	0.7827403
sp|P13761|2B17_HUMAN	-0.5153675	0
sp|P61956-2|SUMO2_HUMAN	-0.5114403	0.656254
sp|P08134|RHOC_HUMAN	-0.5084248	0.7827403
sp|O60504-2|VINEX_HUMAN	-0.5050869	1.1791906
sp|P52788-2|SPSY_HUMAN	-0.5048943	1.1932944
sp|Q15637-2|SF01_HUMAN	-0.5035191	1.1932944
sp|O00712-4|NFIB_HUMAN	-0.5001755	0.656254
sp|P16930|FAAA_HUMAN	-0.4940586	0.656254
sp|P62745|RHOB_HUMAN	-0.4932556	0.656254
sp|O95197-2|RTN3_HUMAN	-0.4891586	1.1932944
sp|P02741|CRP_HUMAN	-0.4886475	1.1932944
sp|P16104|H2AX_HUMAN	-0.4858141	0
sp|Q53GQ0|DHB12_HUMAN	-0.4801388	0.35795313
sp|P27635|RL10_HUMAN	-0.476347	0.656254
sp|Q9NVD7|PARVA_HUMAN	-0.4749851	0
sp|P10619-2|PPGB_HUMAN	-0.4711189	1.1932944
sp|Q96BM9|ARL8A_HUMAN	-0.4705067	0.656254
sp|P0CG39|POTEJ_HUMAN	-0.4654179	0.656254
sp|P11233|RALA_HUMAN	-0.4633789	0.7588735
sp|Q9NPJ3|ACO13_HUMAN	-0.4520187	1.1932944
sp|O94811|TPPP_HUMAN	-0.450778	0.7827403
sp|Q14697|GANAB_HUMAN	-0.4491024	1.1932944
sp|P27824-2|CALX_HUMAN	-0.4480934	0.7827403
sp|P51991-2|ROA3_HUMAN	-0.4477043	0.656254
sp|P42167|LAP2B_HUMAN	-0.4474945	1.1932944
sp|Q13751|LAMB3_HUMAN	-0.443264	0.7061832
sp|O60701-2|UGDH_HUMAN	-0.4427643	0.656254
sp|Q9NYF8-2|BCLF1_HUMAN	-0.4392967	1.1932944
sp|P10412|H14_HUMAN	-0.4392281	1.1932944
sp|Q7L2H7|EIF3M_HUMAN	-0.4324799	0.656254
sp|Q9UL25|RAB21_HUMAN	-0.4307156	1.3006523
sp|Q13813-2|SPTN1_HUMAN	-0.4277763	1.1932944
sp|P36542-2|ATPG_HUMAN	-0.4274788	0.656254
sp|P20292|AL5AP_HUMAN	-0.424902	0.5204253
sp|Q8NBF2-2|NHLC2_HUMAN	-0.4220696	0.7827403
sp|P09110|THIK_HUMAN	-0.4210596	1.2095301
sp|P11234-2|RALB_HUMAN	-0.4184609	0.656254
sp|Q8TCJ2|STT3B_HUMAN	-0.4167156	0.35795313
sp|P16403|H12_HUMAN	-0.4166088	0
sp|Q9UGT4|SUSD2_HUMAN	-0.4162254	0.84879977
sp|Q15181|IPYR_HUMAN	-0.407404	1.2941489
sp|Q9H8L6|MMRN2_HUMAN	-0.4020309	1.1932944
sp|Q9UL18|AGO1_HUMAN	-0.3989162	0.35795313

sp|P14923|PLAK_HUMAN	-0.3981018	1.2095301
sp|Q9GZM7-3|TINAL_HUMAN	-0.3954325	0.45033538
sp|P78406|RAE1L_HUMAN	-0.3949471	1.1932944
sp|P08621-3|RU17_HUMAN	-0.3932324	1.3006523
sp|Q08170|SRSF4_HUMAN	-0.392271	0.656254
sp|P11387|TOP1_HUMAN	-0.3898125	1.1932944
sp|P09601|HMOX1_HUMAN	-0.3888245	1.0485198
sp|P26373|RL13_HUMAN	-0.3871594	1.0485198
sp|P18077|RL35A_HUMAN	-0.3798161	1.2095301
sp|A5A3E0|POTEF_HUMAN	-0.3789768	0
sp|P30419-2|NMT1_HUMAN	-0.3773308	0.656254
sp|Q71UM5|RS27L_HUMAN	-0.3693848	1.1932944
sp|O75923-11|DYSF_HUMAN	-0.3688831	1.0301651
sp|Q13938-4|CAYP1_HUMAN	-0.3677959	0
sp|Q99729-3|ROAA_HUMAN	-0.3676491	0.51870745
sp|Q8TD06|AGR3_HUMAN	-0.3638172	0.7827403
sp|E9PAV3|NACAM_HUMAN	-0.3620663	1.1932944
sp|Q6YN16|HSDL2_HUMAN	-0.3606701	0.35795313
sp|Q16822|PCKGM_HUMAN	-0.3598557	0.7827403
sp|Q9NZU5-2|LMCD1_HUMAN	-0.3593102	1.1932943
sp|P53634|CATC_HUMAN	-0.3567467	0.5204253
sp|P31153|METK2_HUMAN	-0.3543358	0.656254
sp|Q8TCD5|NT5C_HUMAN	-0.3516407	0.45033538
sp|O60784-3|TOM1_HUMAN	-0.345314	0.656254
sp|Q96HD1|CREL1_HUMAN	-0.3451023	0.45033538
sp|P09651-3|ROA1_HUMAN	-0.3445778	1.2773042
sp|P11717|MPRI_HUMAN	-0.3434715	1.1932944
sp|Q5JPE7-2|NOMO2_HUMAN	-0.3434219	1.1505735
sp|Q9BWS9-3|CHID1_HUMAN	-0.3411751	0.656254
sp|P48163-2|MAOX_HUMAN	-0.3403511	0.5204253
sp|P0DPI2-2|GAL3A_HUMAN	-0.3396473	0.2178309
sp|O95394-3|AGM1_HUMAN	-0.3395577	1.1932944
sp|Q9BTE1|DCTN5_HUMAN	-0.3394489	1.1932944
sp|P12694|ODBA_HUMAN	-0.3376274	0.7061832
sp|P30049|ATPD_HUMAN	-0.3363037	1.1932944
sp|P60709|ACTB_HUMAN	-0.3354721	0.656254
sp|Q9UH99-3|SUN2_HUMAN	-0.3347397	0.5111962
sp|P63261|ACTG_HUMAN	-0.3335762	0.656254
sp|P63220|RS21_HUMAN	-0.330843	1.1932944
sp|P62995-3|TRA2B_HUMAN	-0.329422	1.1932944
sp|Q13642-1|FHL1_HUMAN	-0.3260574	0.656254
sp|P06703|S10A6_HUMAN	-0.3257027	1.1932944
sp|P29972-2|AQP1_HUMAN	-0.3255425	0
sp|Q9UQ80|PA2G4_HUMAN	-0.3225594	0.656254
sp|P11279|LAMP1_HUMAN	-0.3220005	0.7827403

sp|A0A0B4J1X8|HV343_HUMAN	-0.3219662	0
sp|O94905|ERLN2_HUMAN	-0.320879	1.1932944
sp|P62714|PP2AB_HUMAN	-0.3198471	0.656254
sp|Q13838-2|DX39B_HUMAN	-0.319561	0.5111962
sp|Q9H3N1|TMX1_HUMAN	-0.316803	0.5204253
sp|Q92522|H1X_HUMAN	-0.3143053	0.7827403
sp|Q9Y371-2|SHLB1_HUMAN	-0.3132744	0
sp|Q92888-2|ARHG1_HUMAN	-0.312789	0.80804527
sp|P02792|FRIL_HUMAN	-0.3124561	1.2090194
sp|P02769|ALBU_BOVIN	-0.3114777	0.06312432
sp|P07948-2|LYN_HUMAN	-0.3111439	0.7827403
sp|P29144|TPP2_HUMAN	-0.3106537	0.80804527
sp|P31946-2|1433B_HUMAN	-0.3095512	0.656254
sp|Q9Y696|CLIC4_HUMAN	-0.3094864	0.70235044
sp|Q9UBS4|DJB11_HUMAN	-0.3064899	1.1932944
sp|Q05315|LEG10_HUMAN	-0.3061867	0.45033538
sp|P60033|CD81_HUMAN	-0.3059845	1.1932944
sp|P07437|TBB5_HUMAN	-0.3059635	0.06291623
sp|P31943|HNRH1_HUMAN	-0.304493	1.2607617
sp|P13284|GILT_HUMAN	-0.304143	0.80804527
sp|Q9HB71|CYBP_HUMAN	-0.3039208	0.6070219
sp|P29466-2|CASP1_HUMAN	-0.3027306	0.656254
sp|P51858|HDGF_HUMAN	-0.302166	1.2095301
sp|P25788-2|PSA3_HUMAN	-0.3010216	1.3006523
sp|P06239-3|LCK_HUMAN	-0.3006439	0.656254
sp|P06865|HEXA_HUMAN	-0.3003883	0.35795313
sp|Q86Y82|STX12_HUMAN	-0.2996311	1.0634323
sp|Q6DKJ4|NXN_HUMAN	-0.2989235	0.19149946
sp|P63096|GNAI1_HUMAN	-0.2971363	0.91601294
sp|P46781|RS9_HUMAN	-0.2952652	0.758334
sp|Q9NX63|MIC19_HUMAN	-0.2951069	1.1932944
sp|Q15365|PCBP1_HUMAN	-0.2921429	1.1240381
sp|Q9BRA2|TXD17_HUMAN	-0.2905502	1.2095301
sp|P61313|RL15_HUMAN	-0.2888489	0.19149946
sp|Q92696|PGTA_HUMAN	-0.2885799	1.1791906
sp|Q07812-7|BAX_HUMAN	-0.2869225	0.19149946
sp|Q9P2J5-2|SYLC_HUMAN	-0.2842045	0.7827403
sp|Q6ZVM7-3|TM1L2_HUMAN	-0.2813854	1.1932944
sp|P62879|GBB2_HUMAN	-0.2806034	1.2095301
sp|P61160|ARP2_HUMAN	-0.2797737	1.1932944
sp|P04083|ANXA1_HUMAN	-0.2785759	1.0173417
sp|P01892|1A02_HUMAN	-0.2784958	0.2178309
sp|P52895|AK1C2_HUMAN	-0.2780962	0.45033538
sp|O15067|PUR4_HUMAN	-0.2779741	0.7827403
sp|Q13200|PSMD2_HUMAN	-0.2779236	0.7061832

sp|O94973-2|AP2A2_HUMAN	-0.2756901	1.2184663
sp|Q562R1|ACTBL_HUMAN	-0.2748184	0.312067
sp|Q92973-2|TNPO1_HUMAN	-0.2741661	1.2095301
sp|P04066|FUCO_HUMAN	-0.2735539	0.7061832
sp|P13693|TCTP_HUMAN	-0.2704716	1.1932944
sp|P09417-2|DHPR_HUMAN	-0.2698097	0.45033538
sp|P31946|1433B_HUMAN	-0.2687035	0.656254
sp|P08754|GNAI3_HUMAN	-0.2681332	0.8983557
sp|Q92817|EVPL_HUMAN	-0.2671051	1.2773042
sp|P19404|NDUV2_HUMAN	-0.2650776	1.1932944
sp|Q99436|PSB7_HUMAN	-0.2645378	0.7827403
sp|Q13435|SF3B2_HUMAN	-0.2643089	0.6070219
sp|P18428|LBP_HUMAN	-0.2609329	0.7061832
sp|P55327-3|TPD52_HUMAN	-0.26087	1.1932944
sp|P00966|ASSY_HUMAN	-0.2593384	1.214352
sp|Q9UGI8-2|TES_HUMAN	-0.2587376	0.48353976
sp|P42226|STAT6_HUMAN	-0.2583046	0.656254
sp|P54886-2|P5CS_HUMAN	-0.2578373	0.7061832
sp|P49903-2|SPS1_HUMAN	-0.2560291	0.7061832
sp|P07305-2|H10_HUMAN	-0.2556343	0.43633315
sp|Q13247-3|SRSF6_HUMAN	-0.2548723	0.45033538
sp|Q10713|MPPA_HUMAN	-0.2547417	0.70235044
sp|Q9BQE3|TBA1C_HUMAN	-0.2529163	0.656254
sp|O75533|SF3B1_HUMAN	-0.2524395	1.1932944
sp|P68366-2|TBA4A_HUMAN	-0.252348	0.8983557
sp|Q9HD45|TM9S3_HUMAN	-0.2508011	0.45033538
sp|P46976-2|GLYG_HUMAN	-0.250042	1.3006523
sp|Q96M27-3|PRRC1_HUMAN	-0.2491875	0.7827403
sp|Q96FN4|CPNE2_HUMAN	-0.2491036	0
sp|P48047|ATPO_HUMAN	-0.248497	0.33495146
sp|P49354-2|FNTA_HUMAN	-0.2484217	0.8983557
sp|P50135|HNMT_HUMAN	-0.2439041	0.45033538
sp|P50213|IDH3A_HUMAN	-0.2436619	0.48757824
sp|Q9UN86-2|G3BP2_HUMAN	-0.243578	0
sp|O43852-3|CALU_HUMAN	-0.2431221	0.40256184
sp|Q8NHP8|PLBL2_HUMAN	-0.2429314	0.19149946
sp|Q9Y3F4-2|STRAP_HUMAN	-0.2423401	0.2178309
sp|Q9NR56-2|MBNL1_HUMAN	-0.2419548	1.1932944
sp|P36551|HEM6_HUMAN	-0.2413521	0
sp|P41218|MNDA_HUMAN	-0.2412376	0.30372584
sp|P08236-2|BGLR_HUMAN	-0.2400131	0.6298893
sp|P15559-2|NQO1_HUMAN	-0.2385063	0.7827403
sp|P61019|RAB2A_HUMAN	-0.2368794	0.66088426
sp|P15586-2|GNS_HUMAN	-0.2366142	0.35795313
sp|Q9Y383-3|LC7L2_HUMAN	-0.2357864	0.5204253

sp|Q13418|ILK_HUMAN	-0.2325401	0.96371543
sp|P20645|MPRD_HUMAN	-0.2319775	1.0301651
sp|P61353|RL27_HUMAN	-0.2314682	1.1932944
sp|Q9UNF0-2|PACN2_HUMAN	-0.2310333	0.84879977
sp|P30838|AL3A1_HUMAN	-0.2308254	0.45033538
sp|Q96HE7|ERO1A_HUMAN	-0.2306175	0.91601294
sp|Q9NTK5|OLA1_HUMAN	-0.2304077	0.45033538
sp|Q8WXF1|PSPC1_HUMAN	-0.2284222	0.95332193
sp|P10644|KAP0_HUMAN	-0.228262	0.6070219
sp|P16435|NCPR_HUMAN	-0.2256432	0.7868989
sp|O95716|RAB3D_HUMAN	-0.2254114	0.656254
sp|P11217|PYGM_HUMAN	-0.2243996	0.656254
sp|P30050|RL12_HUMAN	-0.2227974	1.2095301
sp|Q9HB40|RISC_HUMAN	-0.2221756	1.0301651
sp|Q9Y3A3-3|PHOCN_HUMAN	-0.221796	0
sp|P16671-4|CD36_HUMAN	-0.2216778	0.8983557
sp|P28070|PSB4_HUMAN	-0.2206497	0.7217418
sp|P33316|DUT_HUMAN	-0.2202625	1.1932944
sp|P67775|PP2AA_HUMAN	-0.2194862	0.656254
sp|Q9UBV8|PEF1_HUMAN	-0.2191906	0.91601294
sp|Q96C86|DCPS_HUMAN	-0.2189369	0.5204253
sp|O43684-2|BUB3_HUMAN	-0.2186756	1.1932944
sp|Q99426|TBCB_HUMAN	-0.2185459	0.91601294
sp|P84095|RHOG_HUMAN	-0.2183456	0.5204253
sp|P46926|GNPI1_HUMAN	-0.2180367	1.0485198
sp|P25705|ATPA_HUMAN	-0.2178536	0.45563722
sp|Q9NZ08-2|ERAP1_HUMAN	-0.2168121	1.1932943
sp|Q7Z7H5-3|TMED4_HUMAN	-0.2166328	0.7827403
sp|P09429|HMGB1_HUMAN	-0.2163048	0.45033538
sp|Q8NDH3|PEPL1_HUMAN	-0.2158794	0.09750395
sp|P23588|IF4B_HUMAN	-0.2155094	0.5692702
sp|Q15691|MARE1_HUMAN	-0.2141657	1.0634323
sp|P02794|FRIH_HUMAN	-0.2126675	0.48520416
sp|Q03519|TAP2_HUMAN	-0.2126064	0.656254
sp|Q9BS26|ERP44_HUMAN	-0.2110882	1.1791906
sp|O95782-2|AP2A1_HUMAN	-0.2109661	0.20467198
sp|Q14108|SCRB2_HUMAN	-0.2106056	0.19149946
sp|P61923|COPZ1_HUMAN	-0.2105074	0.19149946
sp|Q6IAA8|LTOR1_HUMAN	-0.2102642	0.19149946
sp|O15260-2|SURF4_HUMAN	-0.2098341	0.45033538
sp|Q15149-3|PLEC_HUMAN	-0.2073174	0.656254
sp|P07910-2|HNRPC_HUMAN	-0.2066193	0.7588735
sp|Q04760-2|LGUL_HUMAN	-0.2055035	0.06291623
sp|P23368|MAOM_HUMAN	-0.2054634	0.93112767
sp|P53396-2|ACLY_HUMAN	-0.205101	1.1205355

sp|Q92747|ARC1A_HUMAN	-0.2043076	0.656254
sp|P50552|VASP_HUMAN	-0.2040854	0.87291557
sp|P28676|GRAN_HUMAN	-0.2039318	0.45033538
sp|Q14019|COTL1_HUMAN	-0.2038822	1.3006523
BirA-TRIP6_BirAT6	-0.2023964	0.7827403
sp|Q9P2T1-2|GMPR2_HUMAN	-0.2018871	0.33495146
sp|P62495-2|ERF1_HUMAN	-0.201807	0.35795313
sp|P62942|FKB1A_HUMAN	-0.2014885	0.656254
sp|Q9HCB6|SPON1_HUMAN	-0.2009659	1.120602
sp|Q01813-2|PFKAP_HUMAN	-0.1984959	1.3006523
sp|O00391|QSOX1_HUMAN	-0.1980534	0.45033538
sp|P55010|IF5_HUMAN	-0.1980438	0.97209185
sp|Q13596-2|SNX1_HUMAN	-0.1975117	0
sp|P47985|UCRI_HUMAN	-0.1968727	0.91601294
sp|P40123-2|CAP2_HUMAN	-0.1958523	0
sp|Q9P2X0-2|DPM3_HUMAN	-0.1957626	0.7827403
sp|O43491|E41L2_HUMAN	-0.195282	1.2438905
sp|Q9UKK9|NUDT5_HUMAN	-0.1952496	0.45033538
sp|Q9UHX1-6|PUF60_HUMAN	-0.193675	0.35795313
sp|Q96JB5-4|CK5P3_HUMAN	-0.1934624	0.35795313
sp|Q9Y5P6-2|GMPPB_HUMAN	-0.1934357	0.04707252
sp|Q96EP5-2|DAZP1_HUMAN	-0.1934109	0.7827403
sp|Q5TFE4|NT5D1_HUMAN	-0.1927662	0.312067
sp|P49458|SRP09_HUMAN	-0.1922703	1.1932944
sp|P31689-2|DNJA1_HUMAN	-0.1903915	1.1932944
sp|P62820|RAB1A_HUMAN	-0.1901436	1.0485198
sp|O43776|SYNC_HUMAN	-0.1900711	0.48520416
sp|Q32P44|EMAL3_HUMAN	-0.1899586	0.80804527
sp|P45954-2|ACDSB_HUMAN	-0.1898975	0
sp|P08246|ELNE_HUMAN	-0.1897965	1.1932944
sp|Q9Y2Q5|LTOR2_HUMAN	-0.1895332	0.45033538
sp|O14744|ANM5_HUMAN	-0.1890755	0.7827403
sp|Q9Y3Z3|SAMH1_HUMAN	-0.1887322	1.1803217
sp|Q00577|PURA_HUMAN	-0.1884632	1.3006523
sp|P42566|EPS15_HUMAN	-0.1877098	1.1932944
sp|P29350-3|PTN6_HUMAN	-0.1875629	0.65625405
sp|Q8IV08|PLD3_HUMAN	-0.1875153	0.09894868
sp|P01111|RASN_HUMAN	-0.1874924	0.656254
sp|P40763-3|STAT3_HUMAN	-0.1874523	1.2095301
sp|Q9NUJ1-3|ABHDA_HUMAN	-0.1866665	0.7827403
sp|O43252|PAPS1_HUMAN	-0.1866627	0.61034113
sp|P04440|DPB1_HUMAN	-0.1864281	0
sp|Q9BW30|TPPP3_HUMAN	-0.1859093	0.34323573
sp|Q9UPN3|MACF1_HUMAN	-0.1857252	1.1888912
sp|P61457|PHS_HUMAN	-0.1856241	0.7827403

sp|P55795|HNRH2_HUMAN	-0.1854649	0.69308156
sp|O76011|KRT34_HUMAN	-0.1848869	0
sp|P09496-2|CLCA_HUMAN	-0.1847782	1.0485198
sp|Q96C23|GALM_HUMAN	-0.1840725	0.09894868
sp|Q09028-3|RBBP4_HUMAN	-0.1831188	0.656254
sp|O14974-3|MYPT1_HUMAN	-0.1829605	1.1791906
sp|P46779-2|RL28_HUMAN	-0.1816578	0.5111962
sp|Q99829|CPNE1_HUMAN	-0.1806421	0.51870745
sp|P39687|AN32A_HUMAN	-0.1800289	0.91601294
sp|Q9UBW8|CSN7A_HUMAN	-0.1797104	0.91601294
sp|Q14344|GNA13_HUMAN	-0.179472	0.8983557
sp|P07814|SYEP_HUMAN	-0.1788397	1.2064548
sp|Q92769|HDAC2_HUMAN	-0.1785564	0.656254
sp|Q9UHQ9|NB5R1_HUMAN	-0.1785564	0.7588735
sp|P32455|GBP1_HUMAN	-0.1781864	0.3005443
sp|P10606|COX5B_HUMAN	-0.177248	0.5204253
sp|P24557-2|THAS_HUMAN	-0.1771584	0.7827403
sp|Q13232|NDK3_HUMAN	-0.176754	0.7588735
sp|Q969G5|CAVN3_HUMAN	-0.1753769	0.35795313
sp|P15529-10|MCP_HUMAN	-0.1748505	0.656254
sp|P18085|ARF4_HUMAN	-0.1746635	0.656254
sp|P00403|COX2_HUMAN	-0.1746483	0.7588735
sp|P35080-2|PROF2_HUMAN	-0.1730957	0.656254
sp|P62701|RS4X_HUMAN	-0.172781	0.6298893
sp|P12111-2|CO6A3_HUMAN	-0.1727734	1.214352
sp|O75947-2|ATP5H_HUMAN	-0.1726608	1.1240381
sp|P12955|PEPD_HUMAN	-0.1726456	0.03367973
sp|Q92542|NICA_HUMAN	-0.1722508	1.1505735
sp|Q9Y3B3|TMED7_HUMAN	-0.1712475	1.1932944
sp|P60866-2|RS20_HUMAN	-0.1705341	1.1932944
sp|Q12907|LMAN2_HUMAN	-0.169899	0.7957244
sp|Q02818|NUCB1_HUMAN	-0.1696081	0.7804068
sp|Q9ULZ3-2|ASC_HUMAN	-0.1694832	0
sp|P62266|RS23_HUMAN	-0.1692715	0.14672586
sp|Q06278|AOXA_HUMAN	-0.1684551	0.19149946
sp|P51178-2|PLCD1_HUMAN	-0.1679573	0
sp|Q96MM6|HS12B_HUMAN	-0.1674786	0.7217418
sp|Q13404|UB2V1_HUMAN	-0.1672993	0.04707252
sp|P26368-2|U2AF2_HUMAN	-0.1667595	1.1932944
sp|Q86W92-2|LIPB1_HUMAN	-0.1665487	0.45033538
sp|P42126-2|ECI1_HUMAN	-0.1659489	1.1932944
sp|Q9Y2X3|NOP58_HUMAN	-0.1651497	0.56808305
sp|P37108|SRP14_HUMAN	-0.1644878	0.7827403
sp|P30086|PEBP1_HUMAN	-0.1641159	1.0076748
sp|P23786|CPT2_HUMAN	-0.1641007	0

sp|Q96S97|MYADM_HUMAN	-0.1632462	0.45033538
sp|Q16881-2|TRXR1_HUMAN	-0.1632309	0.98032326
sp|P16070-7|CD44_HUMAN	-0.1631584	0.61034113
sp|P31942-2|HNRH3_HUMAN	-0.1623783	0.84879977
sp|P52306-4|GDS1_HUMAN	-0.1613064	0.5204253
sp|P08572|CO4A2_HUMAN	-0.1605988	1.0475321
sp|Q06136|KDSR_HUMAN	-0.1602287	1.1932944
sp|Q9UNM6-2|PSD13_HUMAN	-0.160202	1.0634323
sp|Q6UWY5|OLFL1_HUMAN	-0.1590424	0.9780152
sp|Q14258|TRI25_HUMAN	-0.158989	1.0634323
sp|P00338|LDHA_HUMAN	-0.1588745	1.1581028
sp|P54727|RD23B_HUMAN	-0.1573105	0.5204253
sp|Q9NUV9|GIMA4_HUMAN	-0.1565781	0.7957244
sp|P25325-2|THTM_HUMAN	-0.156086	0.5692702
sp|P50454|SERPH_HUMAN	-0.1557961	1.120602
sp|P04222|1C03_HUMAN	-0.1555748	0
sp|Q8TD19|NEK9_HUMAN	-0.1539841	0.45033538
sp|O15247|CLIC2_HUMAN	-0.1535339	0.39873424
sp|P53618|COPB_HUMAN	-0.1527214	0.4378217
sp|P05534|1A24_HUMAN	-0.1527138	0.7061832
sp|P20073-2|ANXA7_HUMAN	-0.1519642	0.2591514
sp|P62753|RS6_HUMAN	-0.1513481	0.7061832
sp|O75608-2|LYPA1_HUMAN	-0.1512108	0.45033538
sp|P49257|LMAN1_HUMAN	-0.1505432	0.11612091
sp|P35270|SPRE_HUMAN	-0.1502304	0.7498006
sp|P62136|PP1A_HUMAN	-0.1500111	0.5204253
sp|Q86U42-2|PABP2_HUMAN	-0.1484127	0.656254
sp|Q9Y3A5|SBDS_HUMAN	-0.1480923	0
sp|Q99439|CNN2_HUMAN	-0.1474991	0.26737198
sp|O75367-2|H2AY_HUMAN	-0.14715	0.84879977
sp|Q99873-3|ANM1_HUMAN	-0.1471481	0.26737198
sp|Q96CN7|ISOC1_HUMAN	-0.1471443	0.95332193
sp|P62244|RS15A_HUMAN	-0.1470299	0
sp|Q14165|MLEC_HUMAN	-0.1469441	1.1505735
sp|Q8NF91-4|SYNE1_HUMAN	-0.1462784	0.45033538
sp|O15145|ARPC3_HUMAN	-0.1461067	0.7061832
sp|Q99832|TCPH_HUMAN	-0.1459541	1.1073172
sp|P62854|RS26_HUMAN	-0.1459217	1.1932944
sp|O43790|KRT86_HUMAN	-0.1456604	0.656254
sp|P28062|PSB8_HUMAN	-0.1456204	0.8983557
sp|Q8IZ83-3|A16A1_HUMAN	-0.1451531	0.95533764
sp|Q9H299|SH3L3_HUMAN	-0.1445141	0.19149946
sp|Q9Y262-2|EIF3L_HUMAN	-0.1435986	0.03367973
sp|O00232|PSD12_HUMAN	-0.141325	1.0485198
sp|P47897|SYQ_HUMAN	-0.1413097	0.7498006

sp|P98082-2|DAB2_HUMAN	-0.1410017	0
sp|O14786|NRP1_HUMAN	-0.1409426	0.19149946
sp|P30084|ECHM_HUMAN	-0.1409273	1.3006523
sp|Q15942|ZYX_HUMAN	-0.1407089	0.56808305
sp|P63241|IF5A1_HUMAN	-0.1404839	0.656254
sp|P09917|LOX5_HUMAN	-0.1395798	0.4075265
sp|Q8TBC4-2|UBA3_HUMAN	-0.1392479	0.19149946
sp|P55265-4|DSRAD_HUMAN	-0.1392288	0.5204253
sp|Q969H8|MYDGF_HUMAN	-0.1392174	0.35795313
sp|Q9BXP5-2|SRRT_HUMAN	-0.1387434	0.5111962
sp|Q96RQ3|MCCA_HUMAN	-0.1386604	0.30372584
sp|Q8TD55|PKHO2_HUMAN	-0.1383495	0.19149946
sp|A6NMZ7|CO6A6_HUMAN	-0.137249	0.56639653
sp|P52790|HXK3_HUMAN	-0.1371269	0.6115017
sp|Q9H0W9-2|CK054_HUMAN	-0.1362925	0.7498006
sp|P21953|ODBB_HUMAN	-0.1358414	0.5204253
sp|P28065-2|PSB9_HUMAN	-0.1355934	0.65625405
sp|P15144|AMPN_HUMAN	-0.1350021	0.91272414
sp|P55060-3|XPO2_HUMAN	-0.1340675	0.6115017
sp|P61163|ACTZ_HUMAN	-0.133997	0.6298893
sp|P62424|RL7A_HUMAN	-0.1335907	0.56808305
sp|Q03154-4|ACY1_HUMAN	-0.1334553	0.35795313
sp|Q9P0V9-2|SEP10_HUMAN	-0.1327257	1.0076748
sp|P54652|HSP72_HUMAN	-0.1323242	0.35795313
sp|Q15417|CNN3_HUMAN	-0.1319981	0.5204253
sp|O95865|DDAH2_HUMAN	-0.1315689	0.95533764
sp|P31930|QCR1_HUMAN	-0.1310597	0.96968824
sp|P53004|BIEA_HUMAN	-0.1300831	0.79861414
sp|O43488|ARK72_HUMAN	-0.1299248	0.21439649
sp|O14787-2|TNPO2_HUMAN	-0.1297836	0
sp|P56134-3|ATPK_HUMAN	-0.128727	0
sp|O60832|DKC1_HUMAN	-0.1278448	0.7827403
sp|Q9NSD9|SYFB_HUMAN	-0.1276188	0.30372584
sp|P59665|DEF1_HUMAN	-0.1274777	0.7588735
sp|P27361|MK03_HUMAN	-0.1257229	0.35795313
sp|Q9NYU2-2|UGGG1_HUMAN	-0.1252937	1.2653638
sp|Q6P2Q9|PRP8_HUMAN	-0.1250076	0.35795313
sp|Q16539-2|MK14_HUMAN	-0.1245718	0.19149946
sp|O43242|PSMD3_HUMAN	-0.124197	0.48520416
sp|O43747-2|AP1G1_HUMAN	-0.1240425	0.35193655
sp|P31948|STIP1_HUMAN	-0.1239395	0
sp|Q709C8-3|VP13C_HUMAN	-0.1236839	0.5832693
sp|Q93052|LPP_HUMAN	-0.1234074	0.26295313
sp|Q13492-2|PICAL_HUMAN	-0.1233959	0.5204253
sp|P48739|PIPNB_HUMAN	-0.12257	0.19149946

sp|Q15717-2|ELAV1_HUMAN	-0.1221676	0.91601294
sp|P34896-2|GLYC_HUMAN	-0.1218739	0
sp|P30048-2|PRDX3_HUMAN	-0.121273	0.7498006
sp|P51553-2|IDH3G_HUMAN	-0.1202059	0.19149946
sp|Q53GG5-2|PDLI3_HUMAN	-0.1201649	0.1342476
sp|Q99733-2|NP1L4_HUMAN	-0.1200275	0.95332193
sp|P08631-2|HCK_HUMAN	-0.1189995	0
sp|P18583-10|SON_HUMAN	-0.1189785	0.19149946
sp|P40227|TCPZ_HUMAN	-0.1184349	1.1054204
sp|P13987-2|CD59_HUMAN	-0.1182365	0.91601294
sp|Q15121|PEA15_HUMAN	-0.1181965	0.2178309
sp|Q9NZL9|MAT2B_HUMAN	-0.1176891	0.6298893
sp|P04424-2|ARLY_HUMAN	-0.117651	0.43103603
sp|Q6NY19-2|KANK3_HUMAN	-0.1174936	0.45033538
sp|Q9H2D6-2|TARA_HUMAN	-0.117094	1.2064548
sp|P01920|DQB1_HUMAN	-0.1169624	0.19149946
sp|O43294|TGFI1_HUMAN	-0.1168633	0
sp|P32456|GBP2_HUMAN	-0.1161728	0.45033538
sp|P52597|HNRPF_HUMAN	-0.1157665	0.34323573
sp|P49721|PSB2_HUMAN	-0.1151085	0.6070219
sp|P12268|IMDH2_HUMAN	-0.1142273	0
sp|Q9BRF8-2|CPPED_HUMAN	-0.1136055	0.1558116
sp|Q12931-2|TRAP1_HUMAN	-0.1135445	0.35795313
sp|Q9Y376|CAB39_HUMAN	-0.112917	0.35795313
sp|Q9H4G4|GAPR1_HUMAN	-0.1125793	0.5204253
sp|Q96G03|PGM2_HUMAN	-0.1120911	0.48353976
sp|Q9NZ01|TECR_HUMAN	-0.1118965	0.40256184
sp|Q13409-3|DC1I2_HUMAN	-0.1117258	0.6509351
sp|P62314|SMD1_HUMAN	-0.1116161	0.19149946
sp|Q96BW5-2|PTER_HUMAN	-0.1114235	0
sp|Q04323-2|UBXN1_HUMAN	-0.1111908	0
sp|Q16630-2|CPSF6_HUMAN	-0.1108646	0.7061832
sp|O60313-10|OPA1_HUMAN	-0.1105928	0.19149946
sp|P22314|UBA1_HUMAN	-0.1103878	0.14575773
sp|P30626-2|SORCN_HUMAN	-0.1101437	0.43103603
sp|P07951|TPM2_HUMAN	-0.1100845	0.29066643
sp|P29692-2|EF1D_HUMAN	-0.1097412	0.7061832
sp|P07951-3|TPM2_HUMAN	-0.1091366	0.5111962
sp|P58107|EPIPL_HUMAN	-0.1082258	0.8235348
sp|P35579-2|MYH9_HUMAN	-0.1081429	0.656254
sp|Q27J81-2|INF2_HUMAN	-0.1076946	0.04707252
sp|Q10567-2|AP1B1_HUMAN	-0.1076126	0.5204253
sp|Q969V3-2|NCLN_HUMAN	-0.10742	0.5204253
sp|P35754|GLRX1_HUMAN	-0.1065254	0.48520416
sp|O95861-4|BPNT1_HUMAN	-0.1063423	0.6298893

sp|P35858-2|ALS_HUMAN	-0.1056719	0
sp|P49720|PSB3_HUMAN	-0.1053543	0.26737198
sp|O00429-6|DNM1L_HUMAN	-0.1043243	0.5832693
sp|P55786|PSA_HUMAN	-0.1042137	1.1721778
sp|P05165-2|PCCA_HUMAN	-0.1032219	0.6563392
sp|Q15274|NADC_HUMAN	-0.1032181	0.2178309
sp|P24534|EF1B_HUMAN	-0.1031761	0.7827403
sp|Q9BVK6|TMED9_HUMAN	-0.1030216	0.5204253
sp|P26599-2|PTBP1_HUMAN	-0.1028748	0.08891098
sp|P04406|G3P_HUMAN	-0.1028481	0
sp|P09972|ALDOC_HUMAN	-0.1023293	0.33495146
sp|Q93034|CUL5_HUMAN	-0.1020622	0
sp|P25789|PSA4_HUMAN	-0.1017227	1.0475321
sp|P00387-3|NB5R3_HUMAN	-0.1016903	0.44572112
sp|O60884|DNJA2_HUMAN	-0.1016254	0.1342476
sp|P50990|TCPQ_HUMAN	-0.1015625	0.83940434
sp|P29992|GNA11_HUMAN	-0.1015339	0.45033538
sp|P55735-2|SEC13_HUMAN	-0.1014118	0.30372584
sp|P33176|KINH_HUMAN	-0.1014023	0.77163273
sp|Q02790|FKBP4_HUMAN	-0.1011562	0.8983557
sp|P30566|PUR8_HUMAN	-0.100996	0.656254
sp|Q04837|SSBP_HUMAN	-0.100893	0.19149946
sp|P17987|TCPA_HUMAN	-0.1001244	1.261843
sp|P55209-2|NP1L1_HUMAN	-0.1001015	0
sp|Q9NP72|RAB18_HUMAN	-0.0990143	0.7588735
sp|Q8TAT6-2|NPL4_HUMAN	-0.0988846	0.30372584
sp|P12277|KCRB_HUMAN	-0.0981503	0.62912476
sp|P34897-2|GLYM_HUMAN	-0.0976133	0.51870745
sp|Q9BT78|CSN4_HUMAN	-0.0967197	0.06291623
sp|P42224-2|STAT1_HUMAN	-0.0963268	0.11949348
sp|Q13464|ROCK1_HUMAN	-0.0956001	0.04707252
sp|Q06210-2|GFPT1_HUMAN	-0.0955124	0.56808305
sp|Q9Y5S9-2|RBM8A_HUMAN	-0.0953732	0.7061832
sp|Q86UX7-2|URP2_HUMAN	-0.0949345	0.94599336
sp|Q14498-2|RBM39_HUMAN	-0.0943337	0.37060758
sp|P50395|GDIB_HUMAN	-0.0938435	0.2746514
sp|P36873-2|PP1G_HUMAN	-0.0936775	0.45033538
sp|P39023|RL3_HUMAN	-0.0935154	0.7588735
sp|Q9H2G2-2|SLK_HUMAN	-0.0932426	0
sp|Q9Y6W5|WASF2_HUMAN	-0.0930157	0.7061832
sp|P07741|APT_HUMAN	-0.0925474	0.02662771
sp|O00534|VMA5A_HUMAN	-0.0923729	0.11949348
sp|Q9BZF9-2|UACA_HUMAN	-0.0923481	0.19149946
sp|P07195|LDHB_HUMAN	-0.0920162	0.79197884
sp|P04792|HSPB1_HUMAN	-0.0915585	0.9296698

sp|P26639-2|SYTC_HUMAN	-0.0907135	0.50639904
sp|O60763-2|USO1_HUMAN	-0.0906239	0.08684197
sp|Q9UBQ5|EIF3K_HUMAN	-0.0906162	0
sp|Q13363-2|CTBP1_HUMAN	-0.0905495	0.19149946
sp|P14618|KPYM_HUMAN	-0.0901222	0.09339783
sp|Q9BUT1|BDH2_HUMAN	-0.0898628	0.48520416
sp|P05166-2|PCCB_HUMAN	-0.0896187	0.17511293
sp|Q9BRR6-2|ADPGK_HUMAN	-0.0891819	0.45033538
sp|Q16181-2|SEPT7_HUMAN	-0.0885201	1.1959618
sp|Q13243-3|SRSF5_HUMAN	-0.0879669	0.656254
sp|Q13185|CBX3_HUMAN	-0.0877647	0.5111962
sp|P26885|FKBP2_HUMAN	-0.0876007	0.19149946
sp|Q05682|CALD1_HUMAN	-0.0875673	0.2178309
sp|O15031|PLXB2_HUMAN	-0.0874729	0.23480128
sp|Q13724-2|MOGS_HUMAN	-0.0874176	0.26546443
sp|Q8N684-3|CPSF7_HUMAN	-0.0871544	0.312067
sp|Q8N163-2|CCAR2_HUMAN	-0.0860844	0.7061832
sp|Q12765|SCRN1_HUMAN	-0.0858765	0.1342476
sp|P05455|LA_HUMAN	-0.08496	0.02174174
sp|Q53T59|H1BP3_HUMAN	-0.0837641	0.19149946
sp|P14780|MMP9_HUMAN	-0.0837059	0.29066643
sp|P21912|SDHB_HUMAN	-0.0829811	0.2178309
sp|O60234|GMFG_HUMAN	-0.0827599	0.09894868
sp|P27487|DPP4_HUMAN	-0.0826321	0.21439649
sp|P09493-8|TPM1_HUMAN	-0.0818138	0.3852666
sp|P52789|HXK2_HUMAN	-0.0814705	0
sp|Q08380|LG3BP_HUMAN	-0.0799522	0.01653191
sp|P35998|PRS7_HUMAN	-0.0798664	0.4668234
sp|P41250|GARS_HUMAN	-0.0796166	0.24295025
sp|P39656-3|OST48_HUMAN	-0.0794125	0.5886828
sp|P46108|CRK_HUMAN	-0.0793743	0.19410844
sp|O75112-7|LDB3_HUMAN	-0.0791245	0.2178309
sp|P21266|GSTM3_HUMAN	-0.0789661	0.14927356
sp|Q9NRV9|HEBP1_HUMAN	-0.078867	0.97209185
sp|Q15582|BGH3_HUMAN	-0.0772095	0.79584783
sp|P26640|SYVC_HUMAN	-0.0771103	0.18414244
sp|P20039|2B1B_HUMAN	-0.0770245	0.656254
sp|O14579|COPE_HUMAN	-0.0764923	0.65625405
sp|P48681|NEST_HUMAN	-0.0756817	0.4116133
sp|P49961-6|ENTP1_HUMAN	-0.0755348	0.35795313
sp|Q99714|HCD2_HUMAN	-0.0753784	0.68888646
sp|P15880|RS2_HUMAN	-0.0739841	0.30372584
sp|Q9NQW7-3|XPP1_HUMAN	-0.0735226	0.10861364
sp|Q9C0B1|FTO_HUMAN	-0.0728149	0.19149946
sp|Q96DG6|CMBL_HUMAN	-0.0706968	0.35795313

sp|O75396|SC22B_HUMAN	-0.0706139	0.5832693
sp|P22894|MMP8_HUMAN	-0.070612	0
sp|P14550|AK1A1_HUMAN	-0.0697784	0.2680669
sp|P46977|STT3A_HUMAN	-0.0694847	0.45033538
sp|P12109|CO6A1_HUMAN	-0.0694008	0.9171579
sp|Q5JRX3-3|PREP_HUMAN	-0.0693893	0.45033538
sp|Q92629-3|SGCD_HUMAN	-0.0690842	0.21439649
sp|Q6UVK1|CSPG4_HUMAN	-0.0674648	0.06842031
sp|Q9Y5M8|SRPRB_HUMAN	-0.0666885	0.5111962
sp|P02511|CRYAB_HUMAN	-0.0662212	0.2178309
sp|Q5SSJ5-2|HP1B3_HUMAN	-0.0661526	0.5111962
sp|P26196|DDX6_HUMAN	-0.065155	0.09894868
sp|Q0ZGT2-4|NEXN_HUMAN	-0.0643578	0.5886828
sp|O14617-4|AP3D1_HUMAN	-0.0643101	0.19149946
sp|P24821-4|TENA_HUMAN	-0.0640354	0.04817784
sp|P46459|NSF_HUMAN	-0.0634422	0
sp|P22234-2|PUR6_HUMAN	-0.0631638	0.6115017
sp|A0AVT1|UBA6_HUMAN	-0.0626278	0.21439649
sp|Q9Y305-4|ACOT9_HUMAN	-0.0623093	0
sp|Q9NYL9|TMOD3_HUMAN	-0.062191	0.30372584
sp|Q14894|CRYM_HUMAN	-0.0617046	0.19149946
sp|P28066|PSA5_HUMAN	-0.0606689	0.26737198
sp|O75915|PRAF3_HUMAN	-0.0601864	0.35795313
sp|Q7LG56-6|RIR2B_HUMAN	-0.0598412	0.19149946
sp|Q9HAV0|GBB4_HUMAN	-0.0595341	0.09894868
sp|P62888|RL30_HUMAN	-0.0591354	0.91601294
sp|P30085|KCY_HUMAN	-0.0587988	0.91601294
sp|P24158|PRTN3_HUMAN	-0.0586929	0.5204253
sp|P57088|TMM33_HUMAN	-0.0576859	0
sp|Q07065|CKAP4_HUMAN	-0.0576096	0.86551994
sp|O76041-2|NEBL_HUMAN	-0.0574188	0
sp|O43143|DHX15_HUMAN	-0.0572929	0.09339783
sp|O15127|SCAM2_HUMAN	-0.0572662	0.35795313
sp|Q9BZZ5-5|API5_HUMAN	-0.0570822	0.26737198
sp|Q12792-3|TWF1_HUMAN	-0.0566521	0.19149946
sp|O75828|CBR3_HUMAN	-0.0560226	0
sp|P49821-2|NDUV1_HUMAN	-0.0548582	0.27902296
sp|P13760|2B14_HUMAN	-0.0536308	0.1558116
sp|Q9BZE9-2|ASPC1_HUMAN	-0.0534077	0
sp|O14980|XPO1_HUMAN	-0.0532494	0.35795313
sp|Q9UNE7-2|CHIP_HUMAN	-0.0532417	0
sp|Q9BWD1|THIC_HUMAN	-0.0529766	0.19149946
sp|O75937|DNJC8_HUMAN	-0.0523033	0.1342476
sp|B5ME19|EIFCL_HUMAN	-0.0522652	0.2178309
sp|P50991-2|TCPD_HUMAN	-0.0499001	0.47741386

sp|P54920|SNAA_HUMAN	-0.0489845	0.4668234
sp|P05787-2|K2C8_HUMAN	-0.0484962	0.38952866
sp|O14818|PSA7_HUMAN	-0.0466805	0.3516469
sp|P61224|RAP1B_HUMAN	-0.0458584	0
sp|P62633-3|CNBP_HUMAN	-0.0454121	0
sp|P19971|TYPH_HUMAN	-0.0447197	0.5939212
sp|Q92688-2|AN32B_HUMAN	-0.0446854	0.35795313
sp|Q63ZY3-3|KANK2_HUMAN	-0.0442696	0.14672586
sp|P23528|COF1_HUMAN	-0.0437012	0.368128
sp|P22059|OSBP1_HUMAN	-0.0432549	0
sp|Q8WX93-5|PALLD_HUMAN	-0.0419235	0.09894868
sp|P36543-2|VATE1_HUMAN	-0.0416126	0.09894868
sp|O00487|PSDE_HUMAN	-0.0397873	0
sp|Q9H9B4|SFXN1_HUMAN	-0.0390167	0.7827403
sp|Q92556|ELMO1_HUMAN	-0.0388546	0.1342476
sp|P43686|PRS6B_HUMAN	-0.0386486	0.30513477
sp|P15153|RAC2_HUMAN	-0.0380974	0
sp|P53602|MVD1_HUMAN	-0.0379982	0.7061832
sp|Q13630|FCL_HUMAN	-0.0369434	0.19149946
sp|P01034|CYTC_HUMAN	-0.0368557	0.19149946
sp|Q9HD89|RETN_HUMAN	-0.0365257	0
sp|P62195|PRS8_HUMAN	-0.0360355	0
sp|O95479|G6PE_HUMAN	-0.0357475	0
sp|Q9UKV3-5|ACINU_HUMAN	-0.0356064	0.1342476
sp|P55809|SCOT1_HUMAN	-0.0350037	0.09894868
sp|Q13126-2|MTAP_HUMAN	-0.034893	0.03367973
sp|P09874|PARP1_HUMAN	-0.0348301	0.39873424
sp|P41091|IF2G_HUMAN	-0.0338097	0.14672586
sp|Q16775-2|GLO2_HUMAN	-0.0337887	0.35795313
sp|P67809|YBOX1_HUMAN	-0.0337448	0.45033538
sp|O75489|NDUS3_HUMAN	-0.0332088	0.30372584
sp|P12004|PCNA_HUMAN	-0.0329094	0
sp|P62829|RL23_HUMAN	-0.0317535	0.19149946
sp|P51692|STA5B_HUMAN	-0.0302134	0.19149946
sp|O15511|ARPC5_HUMAN	-0.0301991	0.21439649
sp|Q9BUF5|TBB6_HUMAN	-0.029953	0.6298893
sp|O75955|FLOT1_HUMAN	-0.0298996	0.3812125
sp|Q14914-2|PTGR1_HUMAN	-0.0291214	0.1342476
sp|Q96AY3|FKB10_HUMAN	-0.0291042	0
sp|Q13561-2|DCTN2_HUMAN	-0.0288296	0.51929367
sp|P09543-2|CN37_HUMAN	-0.0284748	0.20467198
sp|P45974-2|UBP5_HUMAN	-0.0279255	0.110441
sp|Q7KZF4|SND1_HUMAN	-0.0267448	0.01819076
sp|P11498|PYC_HUMAN	-0.0266685	0.1558116
sp|Q86UX2-2|ITIH5_HUMAN	-0.02637	0.312067

sp|P11215-2|ITAM_HUMAN	-0.0258484	0.17511293
sp|Q9UEY8|ADDG_HUMAN	-0.0252876	0.04454162
sp|Q07960|RHG01_HUMAN	-0.0247231	0.4075265
sp|P55884-2|EIF3B_HUMAN	-0.0245533	0.09339783
sp|Q16629-2|SRSF7_HUMAN	-0.024498	0.09750395
sp|O00483|NDUA4_HUMAN	-0.0239429	0
sp|O95571|ETHE1_HUMAN	-0.0228252	0.14672586
sp|O60831|PRAF2_HUMAN	-0.0227375	0.09894868
sp|Q99497|PARK7_HUMAN	-0.0226288	0.70235044
sp|P53041|PPP5_HUMAN	-0.0225849	0
sp|P04844|RPN2_HUMAN	-0.0225306	0.04454162
sp|P52907|CAZA1_HUMAN	-0.0223389	0.3852666
sp|O00170|AIP_HUMAN	-0.022028	0.21439649
sp|Q6UW68|TM205_HUMAN	-0.0217705	0.45033538
sp|O14828-2|SCAM3_HUMAN	-0.0215645	0.09894868
sp|Q13423|NNTM_HUMAN	-0.0206928	0.11949348
sp|Q01995|TAGL_HUMAN	-0.0206833	0.20517272
sp|Q8N392|RHG18_HUMAN	-0.0206099	0
sp|Q9UBC2-2|EP15R_HUMAN	-0.0195723	0.19149946
sp|Q13885|TBB2A_HUMAN	-0.0191956	0.19149946
sp|P50452|SPB8_HUMAN	-0.019062	0.45033538
sp|Q14203-3|DCTN1_HUMAN	-0.0182419	0.19410844
sp|O00151|PDLI1_HUMAN	-0.0181236	0.2699497
sp|P48643|TCPE_HUMAN	-0.017189	0.02863072
sp|Q15436|SC23A_HUMAN	-0.0170994	0.6298893
sp|O60506-3|HNRPQ_HUMAN	-0.0168991	0.06291623
sp|P46783|RS10_HUMAN	-0.0166321	0.19149946
sp|Q9NY33|DPP3_HUMAN	-0.0161133	0.17511293
sp|P78417-3|GSTO1_HUMAN	-0.015955	0
sp|P35579|MYH9_HUMAN	-0.0155678	0.20216827
sp|P61020|RAB5B_HUMAN	-0.0153313	0.09894868
sp|Q6NVY1|HIBCH_HUMAN	-0.0152645	0.06291623
sp|Q92499|DDX1_HUMAN	-0.0144167	0.3852666
sp|P84085|ARF5_HUMAN	-0.0143929	0.656254
sp|O00567|NOP56_HUMAN	-0.0137825	0.2207585
sp|Q9Y230|RUVB2_HUMAN	-0.0135937	0.4024821
sp|P46821|MAP1B_HUMAN	-0.0122662	0.09750395
sp|P51665|PSMD7_HUMAN	-0.0099716	0
sp|P0C0S5|H2AZ_HUMAN	-0.009819	0
sp|Q96CX2|KCD12_HUMAN	-0.0097198	0.16178675
sp|Q9H008|LHPP_HUMAN	-0.009058	0.1500082
sp|Q9C0C2|TB182_HUMAN	-0.0087395	0.20467198
sp|P78417-2|GSTO1_HUMAN	-0.0086708	0
sp|Q9UIJ7|KAD3_HUMAN	-0.0082092	0.1500082
sp|P34932|HSP74_HUMAN	-0.0080681	0.27907842

sp|Q8N1G4|LRC47_HUMAN	-0.0073528	0.16649151
sp|Q12797-10|ASPH_HUMAN	-0.006794	0.21680334
sp|P01911|2B1F_HUMAN	-0.0067558	0
sp|P14174|MIF_HUMAN	-0.0044079	0.2178309
sp|O00571-2|DDX3X_HUMAN	-0.0035191	0.5204253
sp|O76003|GLRX3_HUMAN	-0.0012665	0
sp|Q08257|QOR_HUMAN	-6.56E-04	0.30513477
sp|P09619|PGFRB_HUMAN	-1.37E-04	0
sp|P50502|F10A1_HUMAN	-7.63E-05	0.1063047
sp|P06753-2|TPM3_HUMAN	2.48E-05	0.19149946
sp|Q15005|SPCS2_HUMAN	8.58E-04	0.35795313
sp|P08571|CD14_HUMAN	0.00160789	0
sp|Q15631|TSN_HUMAN	0.00243187	0.1342476
sp|P68400|CSK21_HUMAN	0.00247002	0
sp|Q14204|DYHC1_HUMAN	0.00282097	0.17735331
sp|P36776-3|LONM_HUMAN	0.00373268	0.04454162
sp|P62304|RUXE_HUMAN	0.00404549	0.19149946
sp|Q06323|PSME1_HUMAN	0.00444794	0.26737198
sp|O75306-2|NDUS2_HUMAN	0.00452995	0.30372584
sp|Q99460|PSMD1_HUMAN	0.0056324	0.33691242
sp|O75323|NIPS2_HUMAN	0.0059166	0.19149946
sp|P24844|MYL9_HUMAN	0.00601006	0.09894868
sp|P30837|AL1B1_HUMAN	0.00662804	0.14672586
sp|Q14980-2|NUMA1_HUMAN	0.00734901	0.13745387
sp|O43681|ASNA_HUMAN	0.00738907	0.09894868
sp|P0DOX3|IGD_HUMAN	0.00745392	0
sp|P25787|PSA2_HUMAN	0.00786591	0.2207585
sp|P50570-2|DYN2_HUMAN	0.00875092	0.5832693
sp|P23497|SP100_HUMAN	0.00985909	0
sp|P27695|APEX1_HUMAN	0.0099268	0
sp|O60664-4|PLIN3_HUMAN	0.01015854	0.02174174
sp|P10599-2|THIO_HUMAN	0.01155853	0.1342476
sp|P35749|MYH11_HUMAN	0.01179886	0.18928571
sp|P30533|AMRP_HUMAN	0.01296043	0.09339783
sp|P31939|PUR9_HUMAN	0.01303864	0.24613085
sp|P11586|C1TC_HUMAN	0.01366425	0.09894868
sp|P51911|CNN1_HUMAN	0.01413345	0.18925954
sp|P54819-2|KAD2_HUMAN	0.01461601	0
sp|Q86VP6|CAND1_HUMAN	0.0147934	0.03601474
sp|O43865|SAHH2_HUMAN	0.01570129	0.09894868
sp|Q8NHV1|GIMA7_HUMAN	0.0159111	0
sp|P43243|MATR3_HUMAN	0.01607895	0.26737198
sp|P84098|RL19_HUMAN	0.01673126	0
sp|A0FGR8-2|ESYT2_HUMAN	0.01717377	0.1342476
sp|O43175|SERA_HUMAN	0.01971626	0.55270666

sp|Q9UBE0|SAE1_HUMAN	0.0202713	0.35795313
sp|Q9HC38|GLOD4_HUMAN	0.02279854	0.06986027
sp|P80303-2|NUCB2_HUMAN	0.0232544	0.6298893
sp|P23193-2|TCEA1_HUMAN	0.02389622	0
sp|Q13177|PAK2_HUMAN	0.02598	0
sp|P08708|RS17_HUMAN	0.02640152	0.06842031
sp|P62277|RS13_HUMAN	0.02713585	0.45563722
sp|P17980|PRS6A_HUMAN	0.02727699	0.21989624
sp|P61201-2|CSN2_HUMAN	0.02762985	0.19149946
sp|Q96C19|EFHD2_HUMAN	0.02763367	0.09894868
sp|Q8NCW5|NNRE_HUMAN	0.0285244	0.8983557
sp|O00264|PGRC1_HUMAN	0.02887344	0.11949348
sp|O75746-2|CMC1_HUMAN	0.02970886	0.2178309
sp|Q99961-3|SH3G1_HUMAN	0.02999497	0
sp|P00488|F13A_HUMAN	0.03003883	0.16966018
sp|Q96HC4|PDLI5_HUMAN	0.03015518	0
sp|P48147|PPCE_HUMAN	0.03066826	0.11612091
sp|P61225|RAP2B_HUMAN	0.03162289	0
sp|P54578-3|UBP14_HUMAN	0.03173447	0.30372584
sp|Q15075|EEA1_HUMAN	0.03199959	0.43852544
sp|Q14152|EIF3A_HUMAN	0.03351021	0.2178309
sp|O15372|EIF3H_HUMAN	0.03407574	0
sp|P28072|PSB6_HUMAN	0.03487396	0.09894868
sp|P46063|RECQ1_HUMAN	0.03519058	0.17511293
sp|P49593-2|PPM1F_HUMAN	0.03666306	0.06291623
sp|O15061|SYNEM_HUMAN	0.03671932	0.04454162
sp|P22033|MUTA_HUMAN	0.03676987	0
sp|P51688|SPHM_HUMAN	0.03692818	0.21439649
sp|P54136|SYRC_HUMAN	0.03773689	0.11949348
sp|P50914|RL14_HUMAN	0.03795815	0.2178309
sp|P61026|RAB10_HUMAN	0.03848171	0
sp|P08237-3|PFKAM_HUMAN	0.03876114	0
sp|P50579-2|MAP2_HUMAN	0.04063797	0
sp|Q9UJZ1-2|STML2_HUMAN	0.04171753	0.2178309
sp|Q14112-2|NID2_HUMAN	0.04245949	0.23480128
sp|P23142|FBLN1_HUMAN	0.04329681	0.06986027
sp|P04004|VTNC_HUMAN	0.04371834	0.14672586
sp|P28074|PSB5_HUMAN	0.04502487	0.20467198
sp|O95336|6PGL_HUMAN	0.04512215	0.26737198
sp|P17213|BPI_HUMAN	0.04581451	0.06291623
sp|Q9Y224|RTRAF_HUMAN	0.04638863	0.09339783
sp|P09104-2|ENOG_HUMAN	0.04793644	0.51870745
sp|Q8WXX5|DNJC9_HUMAN	0.04829407	0
sp|O43615|TIM44_HUMAN	0.0483408	0
sp|Q15019-2|SEPT2_HUMAN	0.04846191	0.56228566

sp|P21399|ACOC_HUMAN	0.05005169	0.11612091
sp|P0CG38|POTEI_HUMAN	0.05065727	0
sp|Q9NR45|SIAS_HUMAN	0.05121422	0.2810592
sp|P06748-2|NPM_HUMAN	0.05129051	0.61034113
sp|P16219|ACADS_HUMAN	0.0524416	0.09339783
sp|P58546|MTPN_HUMAN	0.05247498	0.35795313
sp|Q9Y281|COF2_HUMAN	0.05509949	0.2178309
sp|Q9BS40|LXN_HUMAN	0.05510712	0.03043297
sp|P42704|LPPRC_HUMAN	0.05655861	0.11949348
sp|Q16134-3|ETFD_HUMAN	0.05716515	0.19149946
sp|Q5EBM0|CMPK2_HUMAN	0.05771828	0.7061832
sp|Q6XQN6-2|PNCB_HUMAN	0.05907631	0.7455631
sp|P12270|TPR_HUMAN	0.06000137	0.01779479
sp|Q9Y639-4|NPTN_HUMAN	0.06087112	0
sp|P49755|TMEDA_HUMAN	0.06149101	0.30372584
sp|Q9UJU6-2|DBNL_HUMAN	0.06164551	0.30372584
sp|P30520|PURA2_HUMAN	0.06276512	0
sp|Q14847|LASP1_HUMAN	0.06457329	0.01311907
sp|Q9UBT2|SAE2_HUMAN	0.06526756	0
sp|P51636-2|CAV2_HUMAN	0.06585693	0
sp|P67936|TPM4_HUMAN	0.06741524	0.47741386
sp|Q99538-2|LGMN_HUMAN	0.06756783	0.19149946
sp|P04062-2|GLCM_HUMAN	0.06759834	0.45033538
sp|O95466-2|FMNL1_HUMAN	0.06777954	0.7827403
sp|Q9NUQ9|FA49B_HUMAN	0.06778526	0.7061832
sp|Q5R3I4|TTC38_HUMAN	0.0678215	0.45033538
sp|P16298-4|PP2BB_HUMAN	0.06887054	0.312067
sp|O95302-3|FKBP9_HUMAN	0.06952286	0.45033538
sp|Q15008-4|PSMD6_HUMAN	0.07192421	0.45033538
sp|Q9UI12-2|VATH_HUMAN	0.07193565	0.20467198
sp|Q14254|FLOT2_HUMAN	0.07308006	0.1500082
sp|Q92896-2|GSLG1_HUMAN	0.07408333	1.0918401
sp|P62736|ACTA_HUMAN	0.07535744	0
sp|Q92900-2|RENT1_HUMAN	0.07561684	0.14927356
sp|Q13526|PIN1_HUMAN	0.07612419	0.45033538
sp|Q16401-2|PSMD5_HUMAN	0.07741356	0.70235044
sp|Q9NZ32|ARP10_HUMAN	0.07759857	0.45033538
sp|P61088|UBE2N_HUMAN	0.07813072	0.45033538
sp|Q15257-2|PTPA_HUMAN	0.07868004	0.20467198
sp|P61803|DAD1_HUMAN	0.0787468	0
sp|Q8WVM8|SCFD1_HUMAN	0.07989502	0.37060758
sp|O75340-2|PDCD6_HUMAN	0.08143997	0.2178309
sp|A0A0C4DH31|HV118_HUMAN	0.08166504	0
sp|O75368|SH3L1_HUMAN	0.082798	0.91601294
sp|Q9NZK5|ADA2_HUMAN	0.0834465	0.19149946

sp|P62081|RS7_HUMAN	0.08347893	0.04707252
sp|Q16774|KGUA_HUMAN	0.08499527	0
sp|P20160|CAP7_HUMAN	0.0850296	0.09894868
sp|Q08945|SSRP1_HUMAN	0.08506775	0.09339783
sp|O94776|MTA2_HUMAN	0.0852356	0.06291623
sp|Q9NZB2-6|F120A_HUMAN	0.08538818	0.312067
sp|P60983|GMFB_HUMAN	0.0857029	0.09894868
sp|Q9BTV4|TMM43_HUMAN	0.08623123	0.48520416
sp|Q13162|PRDX4_HUMAN	0.08706093	0.5832693
sp|P62191-2|PRS4_HUMAN	0.08908653	0.2178309
sp|P20591|MX1_HUMAN	0.08988953	0.56808305
sp|P27694|RFA1_HUMAN	0.09035873	0
sp|Q8TDL5|BPIB1_HUMAN	0.09057999	0.5283125
sp|P62333|PRS10_HUMAN	0.09116364	0.37060758
sp|Q14515-2|SPRL1_HUMAN	0.09238625	0.19149946
sp|Q14195-2|DPYL3_HUMAN	0.09549713	0.8150251
sp|Q9NR12-2|PDLI7_HUMAN	0.09594154	0.5832693
sp|P61758|PFD3_HUMAN	0.09721184	0.19149946
sp|O75503|CLN5_HUMAN	0.10000992	0
sp|O15400-2|STX7_HUMAN	0.10021019	0.14672586
sp|P84243|H33_HUMAN	0.10060501	0
sp|P30466|1B18_HUMAN	0.10284424	0
sp|Q9C0E8-4|LNP_HUMAN	0.10398006	0.45033538
sp|P00740|FA9_HUMAN	0.10404205	0
sp|P15374|UCHL3_HUMAN	0.10422134	0.6298893
sp|O94855-2|SC24D_HUMAN	0.10474777	0
sp|P05388|RLA0_HUMAN	0.10546684	0.35795313
sp|P36542|ATPG_HUMAN	0.10590744	0.656254
sp|P23229-4|ITA6_HUMAN	0.10641861	0.7827403
sp|Q5K4L6|S27A3_HUMAN	0.10687447	0.35795313
sp|P48449-3|ERG7_HUMAN	0.10794449	0.1342476
sp|Q9NQG5|RPR1B_HUMAN	0.10852242	0.2178309
sp|P06681-3|CO2_HUMAN	0.10959435	0.56808305
sp|P22061-2|PIMT_HUMAN	0.10973358	0.40256184
sp|Q9Y6C2|EMIL1_HUMAN	0.10983658	1.2911863
sp|P05387|RLA2_HUMAN	0.10986137	0.7827403
sp|Q6PCB0|VWA1_HUMAN	0.11120796	0.2178309
sp|P30512|1A29_HUMAN	0.11186218	0.09894868
sp|O60825-2|F262_HUMAN	0.11293602	0.656254
sp|P61970|NTF2_HUMAN	0.11328125	0.91601294
sp|Q3LXA3|TKFC_HUMAN	0.11334801	0.57057804
sp|O60547-2|GMDS_HUMAN	0.1144104	0
sp|Q9Y5Z4|HEBP2_HUMAN	0.11450577	0.35795313
sp|P55145|MANF_HUMAN	0.11548233	0.7827403
sp|Q12882|DPYD_HUMAN	0.11603546	0.45033538

sp|P61006|RAB8A_HUMAN	0.11608315	0
sp|Q02952-2|AKA12_HUMAN	0.1162796	0.7827403
sp|P62910|RL32_HUMAN	0.11692619	0.312067
sp|Q9UNS2|CSN3_HUMAN	0.11805916	0.656254
sp|Q9NRN5-2|OLFL3_HUMAN	0.11837769	0.7173901
sp|P08294|SODE_HUMAN	0.11995316	0.7217418
sp|P30519|HMOX2_HUMAN	0.1201191	0.7827403
sp|Q9UK22|FBX2_HUMAN	0.12110615	0.45033538
sp|P21810|PGS1_HUMAN	0.12182808	0.9402636
sp|O60256-3|KPRB_HUMAN	0.12416267	0.45033538
sp|P14618-2|KPYM_HUMAN	0.12431908	0.2178309
sp|P24666|PPAC_HUMAN	0.1252861	0.19149946
sp|O43396|TXNL1_HUMAN	0.125988	0.45033538
sp|P18084|ITB5_HUMAN	0.12625122	0.7827403
sp|Q93084-2|AT2A3_HUMAN	0.12639618	0.19149946
sp|O60716-14|CTND1_HUMAN	0.12645721	0.5204253
sp|Q96P70|IPO9_HUMAN	0.12784481	0.1342476
sp|Q15833-2|STXB2_HUMAN	0.12936687	0.19149946
sp|P0DJI8|SAA1_HUMAN	0.13446045	0
sp|Q86VS8|HOOK3_HUMAN	0.13574219	0.45033538
sp|Q92930|RAB8B_HUMAN	0.13642502	0.656254
sp|P22102|PUR2_HUMAN	0.13648415	0.35193655
sp|Q86WV6|STING_HUMAN	0.13687515	0.5204253
sp|Q30154|DRB5_HUMAN	0.13705444	0
sp|P53992|SC24C_HUMAN	0.13735199	0.40256184
sp|Q9P2B2|FPRP_HUMAN	0.13742256	0.9533461
sp|Q9HCN8|SDF2L_HUMAN	0.13813972	1.1932944
sp|O15498-2|YKT6_HUMAN	0.13910103	0.7827403
sp|Q15819|UB2V2_HUMAN	0.13981819	0.656254
sp|Q93009-3|UBP7_HUMAN	0.14267921	0
sp|P31937|3HIDH_HUMAN	0.14381409	1.1505735
sp|P55058|PLTP_HUMAN	0.14539146	1.1932944
sp|P39019|RS19_HUMAN	0.14544106	0.5204253
sp|Q9UJS0-2|CMC2_HUMAN	0.14588928	0
sp|Q9Y3D6|FIS1_HUMAN	0.14678669	0.7061832
sp|P15090|FABP4_HUMAN	0.14810753	1.1687785
sp|Q15046|SYK_HUMAN	0.14833832	0.656254
sp|Q9Y265|RUVB1_HUMAN	0.15053368	0.7740364
sp|P52888|THOP1_HUMAN	0.15211105	0.19149946
sp|Q9NP79|VTA1_HUMAN	0.15211582	0.35795313
sp|Q00796|DHSO_HUMAN	0.155159	1.1505735
sp|Q14767|LTBP2_HUMAN	0.15539742	1.1458259
sp|P29400-2|CO4A5_HUMAN	0.15546417	0
sp|P62857|RS28_HUMAN	0.15738487	0.91601294
sp|P56537|IF6_HUMAN	0.15920448	0.37060758

sp|P80188|NGAL_HUMAN	0.15976715	1.214352
sp|P35637-2|FUS_HUMAN	0.1614418	0.7827403
sp|O76074-2|PDE5A_HUMAN	0.16346455	0.43633315
sp|P62070-4|RRAS2_HUMAN	0.16403008	0.656254
sp|O94804|STK10_HUMAN	0.16622353	0.7827403
sp|P21589-2|5NTD_HUMAN	0.16637993	1.1932944
sp|P06132|DCUP_HUMAN	0.16862679	0.17319627
sp|P30046|DOPD_HUMAN	0.17041397	0.7827403
sp|Q13310-2|PABP4_HUMAN	0.17392159	0.656254
sp|P01112|RASH_HUMAN	0.17481804	0.656254
sp|O95833|CLIC3_HUMAN	0.17572594	0.91601294
sp|O14745|NHRF1_HUMAN	0.17724419	0.35795313
sp|Q6P4A8|PLBL1_HUMAN	0.18112183	0
sp|Q16787-3|LAMA3_HUMAN	0.18172073	0.656254
sp|Q7L5N1|CSN6_HUMAN	0.18235588	0.7827403
sp|P02748|CO9_HUMAN	0.18354034	1.1955373
sp|Q9GZT8|NIF3L_HUMAN	0.18515587	0.45033538
sp|P00736|C1R_HUMAN	0.18560886	1.1505735
sp|P05109|S10A8_HUMAN	0.19008064	0.633381
sp|P57737-4|CORO7_HUMAN	0.19169521	0.19149946
sp|P07360|CO8G_HUMAN	0.19398499	0.45033538
sp|P00441|SODC_HUMAN	0.19678497	1.1791906
sp|Q06033-2|ITIH3_HUMAN	0.19892693	0.45033538
sp|Q08378|GOGA3_HUMAN	0.19901562	0.5204253
sp|P30711|GSTT1_HUMAN	0.1994381	0.91601294
sp|P52758|RIDA_HUMAN	0.20117378	0.7827403
sp|Q16643-3|DREB_HUMAN	0.20122337	0.45563722
sp|Q14651|PLSI_HUMAN	0.2027111	0
sp|P09871|C1S_HUMAN	0.20284843	0.2178309
sp|P23142-4|FBLN1_HUMAN	0.20300579	0.656254
sp|P40261|NNMT_HUMAN	0.20323372	0.91601294
sp|P63151-2|2ABA_HUMAN	0.20518303	0
sp|O75347|TBCA_HUMAN	0.21367264	0.5204253
sp|O75964|ATP5L_HUMAN	0.21479797	1.1932944
sp|P27918|PROP_HUMAN	0.21665764	1.1932944
sp|P60891|PRPS1_HUMAN	0.21702576	0.656254
sp|P02753|RET4_HUMAN	0.21958923	1.2773042
sp|P01743|HV146_HUMAN	0.22031784	0.656254
sp|P12814|ACTN1_HUMAN	0.22034264	0
sp|Q9BX97|PLVAP_HUMAN	0.22039795	0.19149946
sp|Q9Y6A4|CFA20_HUMAN	0.22179794	0.19149946
sp|O94979-10|SC31A_HUMAN	0.22183514	0.80804527
sp|P09493-5|TPM1_HUMAN	0.22377205	1.1932944
sp|P42785-2|PCP_HUMAN	0.22517395	1.2773042
sp|Q9UNH7-2|SNX6_HUMAN	0.22527504	0

sp|Q92598-2|HS105_HUMAN	0.22595215	0.656254
sp|P35542|SAA4_HUMAN	0.22671127	1.1932944
sp|P31321|KAP1_HUMAN	0.2269268	0.656254
sp|Q13867|BLMH_HUMAN	0.22817802	0.19149946
sp|O00233-2|PSMD9_HUMAN	0.22914505	0.45033538
sp|Q14141-2|SEPT6_HUMAN	0.2311058	0.656254
sp|P61018-2|RAB4B_HUMAN	0.2346611	0.656254
sp|P07358|CO8B_HUMAN	0.23997593	0.8983557
sp|P62269|RS18_HUMAN	0.24016953	0.6298893
sp|P22352|GPX3_HUMAN	0.24386597	1.1505735
sp|Q14192|FHL2_HUMAN	0.24930954	0.8272904
sp|P01780|HV307_HUMAN	0.25279808	0.656254
sp|P09012|SNRPA_HUMAN	0.26445007	1.1932944
sp|O14791-2|APOL1_HUMAN	0.26728344	0.7827403
sp|P50148|GNAQ_HUMAN	0.2709179	0.6298893
sp|Q15404|RSU1_HUMAN	0.27944183	0.656254
sp|P42285|MTREX_HUMAN	0.28337288	0.19149946
sp|P48741|HSP77_HUMAN	0.28487587	0.656254
sp|Q15149-9|PLEC_HUMAN	0.2925415	0.656254
sp|Q04695|K1C17_HUMAN	0.2936859	0.656254
sp|Q96HN2-2|SAHH3_HUMAN	0.29668045	0.656254
sp|P54577|SYYC_HUMAN	0.29676437	0.1558116
sp|P01019|ANGT_HUMAN	0.29808235	0.8983557
sp|Q9Y5K5-2|UCHL5_HUMAN	0.29846954	1.0301651
sp|P16144-2|ITB4_HUMAN	0.30443668	1.1932944
sp|O94919|ENDD1_HUMAN	0.30462074	0
sp|Q5TZA2|CROCC_HUMAN	0.30473137	0.8983557
sp|P54802|ANAG_HUMAN	0.30673218	0.7827403
sp|Q9P1F3|ABRAL_HUMAN	0.30845833	0.656254
sp|Q96PD5-2|PGRP2_HUMAN	0.32795906	1.0485198
sp|A1L4H1|SRCRL_HUMAN	0.33002186	0.35795313
sp|P23219-2|PGH1_HUMAN	0.33017159	0.8983557
sp|A0A0C4DH38|HV551_HUMAN	0.3303833	1.1932944
sp|O15296|LX15B_HUMAN	0.3349247	0.7588735
sp|Q92905|CSN5_HUMAN	0.34166622	1.1932944
sp|P12814-2|ACTN1_HUMAN	0.34252167	1.1932944
sp|Q15063-3|POSTN_HUMAN	0.34417725	1.1932944
sp|P0DOX2|IGA2_HUMAN	0.34596443	1.1505735
sp|Q66K74-2|MAP1S_HUMAN	0.35225964	1.1932944
sp|Q08379|GOGA2_HUMAN	0.35415077	1.1932944
sp|P01591|IGJ_HUMAN	0.355011	1.1932944
sp|Q8TAQ2-2|SMRC2_HUMAN	0.35608673	1.0301651
sp|Q13976-2|KGP1_HUMAN	0.3590355	0.656254
sp|P62913-2|RL11_HUMAN	0.36660767	0.7827403
sp|Q9GZP4-2|PITH1_HUMAN	0.3700676	1.1932944

sp|P02751-15|FINC_HUMAN	0.37564468	0.656254
sp|P63267|ACTH_HUMAN	0.37917328	0.656254
sp|P05155-2|IC1_HUMAN	0.38098907	1.1932944
sp|A0A0C4DH29|HV103_HUMAN	0.383152	0.656254
sp|P0DP03|HV335_HUMAN	0.3840847	1.1932944
sp|Q9NY15|STAB1_HUMAN	0.38669205	1.1932944
sp|P02747|C1QC_HUMAN	0.3869419	1.1932944
sp|P20339-2|RAB5A_HUMAN	0.38766193	0.656254
sp|Q7Z4H8|PLGT3_HUMAN	0.38907623	0.656254
sp|P0DOX7|IGK_HUMAN	0.3927765	1.2095301
sp|P12235|ADT1_HUMAN	0.39878845	0.656254
sp|P63027|VAMP2_HUMAN	0.4085579	1.1932944
sp|Q04446|GLGB_HUMAN	0.41527843	0.1342476
sp|P02763|A1AG1_HUMAN	0.41578674	1.2773042
sp|P01782|HV309_HUMAN	0.43793678	0.656254
sp|P48059-3|LIMS1_HUMAN	0.4416027	0.656254
sp|Q9H6S3|ES8L2_HUMAN	0.4497528	0.7827403
sp|P0C0L4|CO4A_HUMAN	0.45576668	1.1932944
sp|P02746|C1QB_HUMAN	0.45635033	1.1932944
sp|P13928|ANXA8_HUMAN	0.45711136	1.1932944
sp|Q7Z7G0|TARSH_HUMAN	0.46326828	0.19149946
sp|Q15437|SC23B_HUMAN	0.46767998	0.656254
sp|P54108-2|CRIS3_HUMAN	0.470356	0.656254
sp|P01624|KV315_HUMAN	0.4849682	1.1932944
sp|Q15063-2|POSTN_HUMAN	0.51055145	0.656254
sp|A0A0C4DH25|KVD20_HUMAN	0.51397324	0.656254
sp|P12111-4|CO6A3_HUMAN	0.52407646	0.656254
sp|P25685-2|DNJB1_HUMAN	0.55208397	1.1932944
sp|P0C0L5|CO4B_HUMAN	0.5610542	1.1932944
sp|P01619|KV320_HUMAN	0.56131554	0.656254
sp|P28161|GSTM2_HUMAN	0.5817566	0.656254
sp|Q92599-3|SEPT8_HUMAN	0.62290764	1.1932944
sp|P08185|CBG_HUMAN	0.6303673	1.1932944
sp|Q15323|K1H1_HUMAN	0.6433525	0.656254
sp|Q06828|FMOD_HUMAN	0.65532494	1.1932944
sp|Q96HY6|DDRGK_HUMAN	0.67363167	0.656254
sp|Q687X5|STEA4_HUMAN	0.6975393	1.1932944
sp|Q9NSK0-5|KLC4_HUMAN	0.70474243	0.656254
sp|P02533|K1C14_HUMAN	0.75392437	1.1932944
sp|A0A0C4DH41|HV461_HUMAN	0.76514053	0.656254
sp|P06753-6|TPM3_HUMAN	0.78310966	0.656254
sp|O94788-3|AL1A2_HUMAN	0.80875206	0.656254
sp|P02461|CO3A1_HUMAN	0.8380413	1.1932944
sp|P14207|FOLR2_HUMAN	0.83867264	0.656254
sp|P30613-2|KPYR_HUMAN	0.84132576	0.656254

sp|P08779|K1C16_HUMAN	0.85953236	1.1932944
sp|P01834|IGKC_HUMAN	0.8696346	1.1932944
sp|P06310|KV230_HUMAN	0.9070759	0.656254
sp|P69891|HBG1_HUMAN	0.90751266	0.656254
sp|P23083|HV102_HUMAN	0.90756416	0.656254
sp|A0A075B6P5|KV228_HUMAN	0.9130535	0.656254
sp|P47895|AL1A3_HUMAN	0.93656445	0.656254
sp|P00738|HPT_HUMAN	0.9758568	0.656254
sp|Q03591|FHR1_HUMAN	1.2127085	1.1932944
sp|Q9BTE3-2|MCMBP_HUMAN	1.2362146	0.19149946
sp|P11166|GTR1_HUMAN	1.3974476	1.1932944
sp|P69892|HBG2_HUMAN	1.5076599	0.656254
