# Supplementary material for: Peptidylarginine deiminase 2 citrullinates MZB1 and promotes the secretion of IgM and IgA
Source: Front Immunol. 2023 Nov 29;14:1290585. doi: 10.3389/fimmu.2023.1290585 (PMC10716219; doi:10.3389/fimmu.2023.1290585)
Supplement: Supplementary file 13 [file DataSheet_13.pdf]

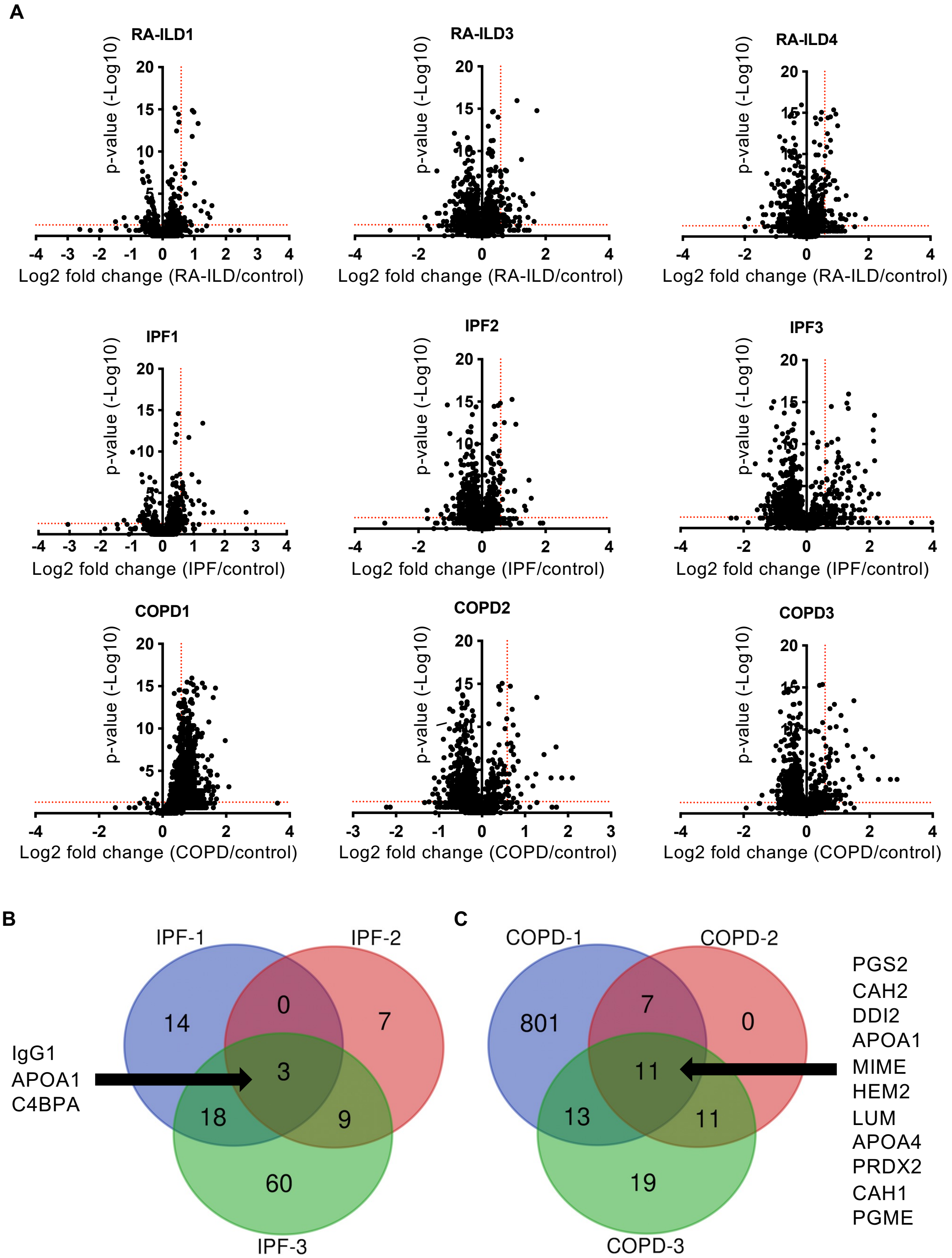

Supplemental Figure 1. Differential enrichment of PG-tagged proteins in diseased lungs. PG-tagged proteins from control and diseased lungs were subjected to mass spectrometry for protein identification. PG-tagged proteins that are differentially enriched in diseased lung samples compared to control lung samples are shown in volcano plots (**A**). The red dotted lines indicate the threshold of fold change (1.5) and p (0.05). The Venn diagrams of PG-tagged proteins enriched in IPF samples and COPD samples are shown in **B** and **C**, respectively.

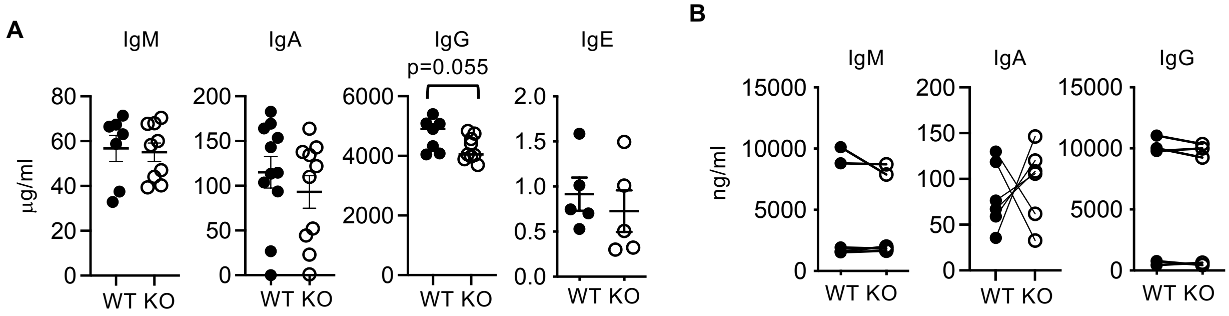

Supplemental Figure 2. Little impact of PAD2 on the secretion of immunoglobulin by mouse plasmablasts. **A**. Serum levels of indicated immunoglobulin from 4-month-old WT and PAD2KO mice were quantified with ELISA. **B**. Their B cells were subjected to in vitro differentiation according to the protocol described in the material and method. The levels of indicated cytokines in the supernatant were measure with ELISA. Data points from the same experiments are connected with lines.

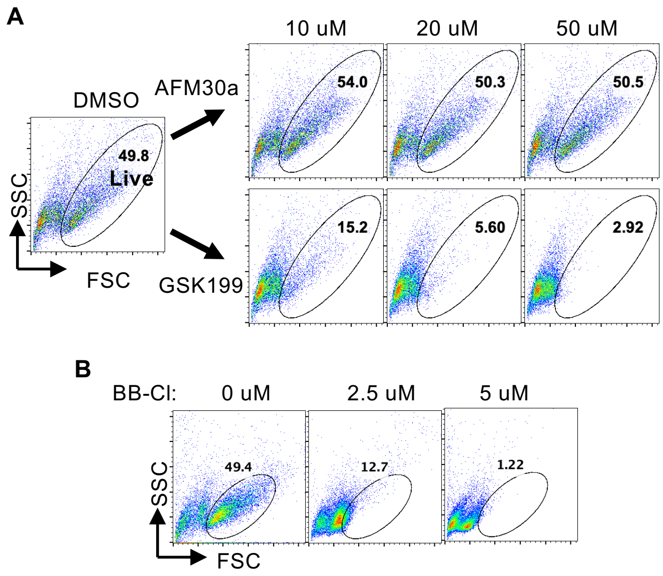

Supplemental Figure 3. Cytotoxic effect of GSK199 and BB-Cl-amidine. Human primary B cells were differentiated into plasmablasts in vitro in the absence (DMSO) or presence of AFM-30a (**A**), GSK199 (**A**), or BB-Cl-amidine (**B**) at the indicated concentration for 4 days. The cells were analyzed with FACS. Representative FSC/SSC plots are shown.

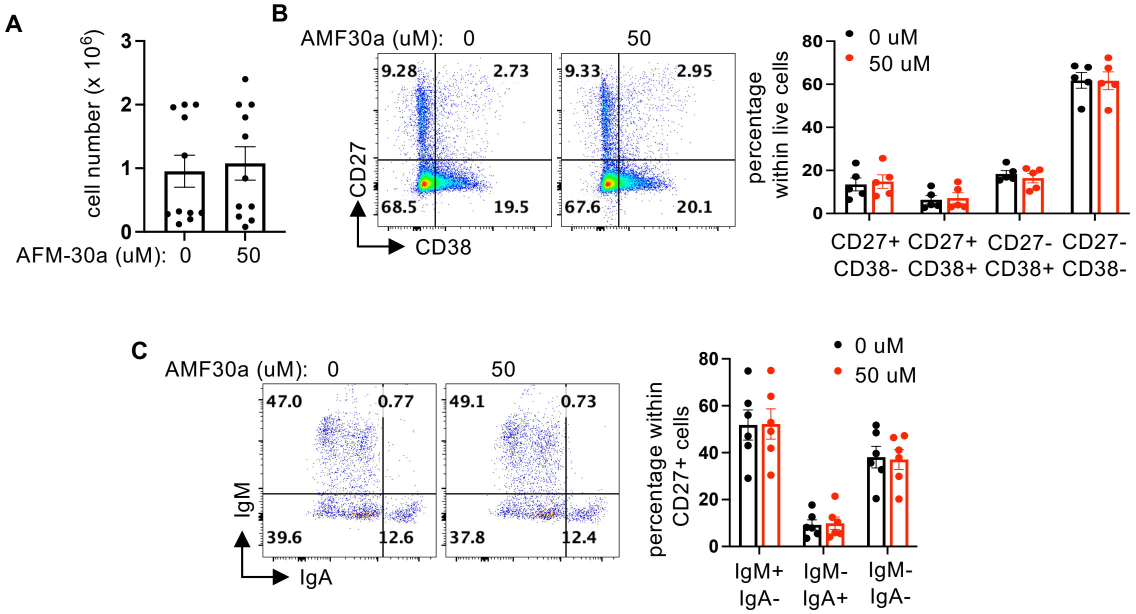

Supplemental Figure 4. Impact of AFM-30a on B cells. $CD19^+$ cells were subjected to in vitro plasmablast differentiation in the presence or absence of AFM-30a as described in Figure 5. The absolute cell counts 4 days later are shown in **A**. Representative CD27/CD38 plots of the live cells indicated in Figure 5B are shown in **B**. Representative intracellular IgM/IgA plots of $CD27^+$ cells from **B** are shown in **C**. Raw percentages of indicated gates from at least 4 experiments are shown in the bar graph of panel **B** and **C**.

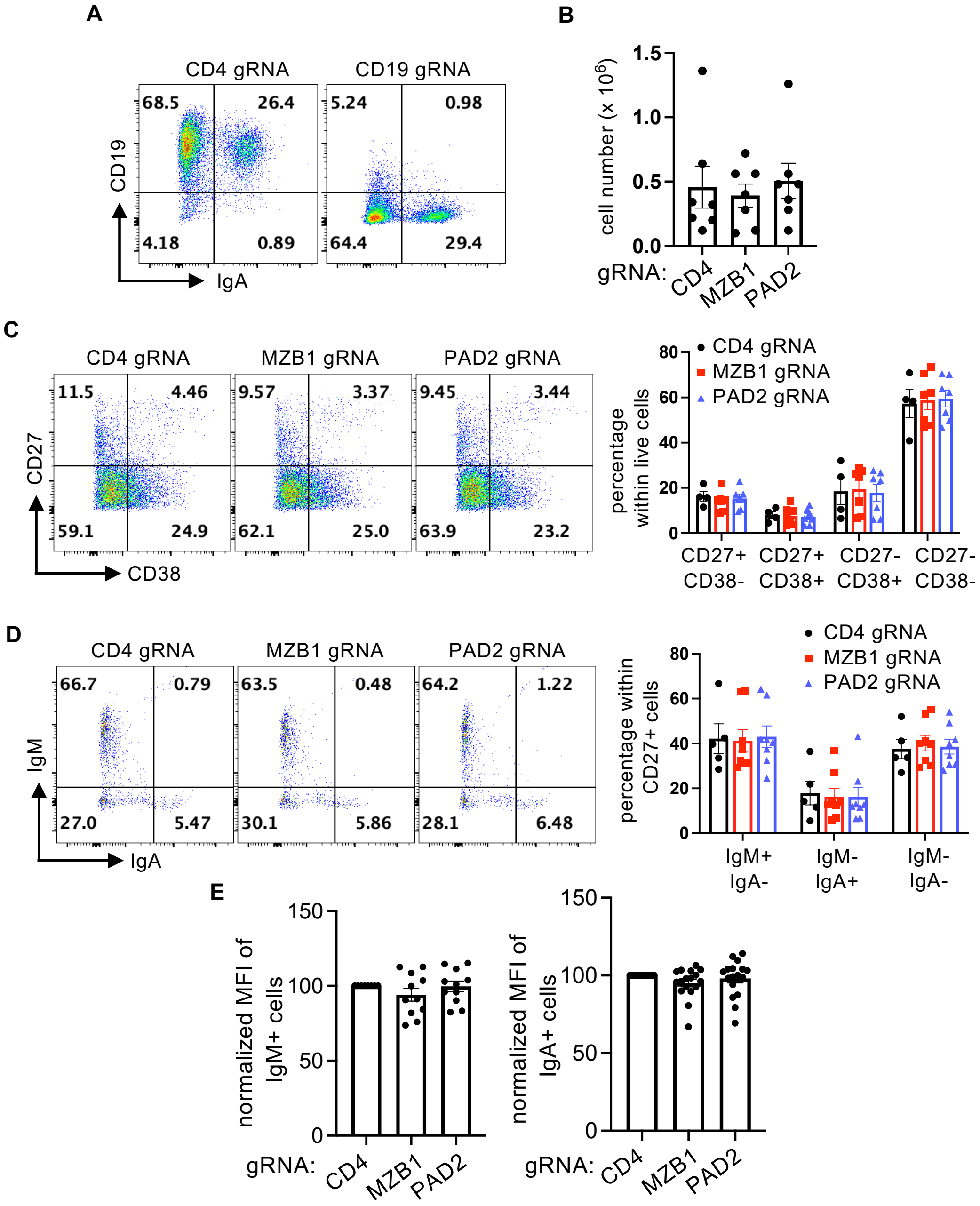

Supplemental Figure 5. CRISPR of differentiating primary human plasmablasts. A. B cells purified from health donors were subjected to CRISPR with gRNA against CD4 and CD19, and then differentiated into plasmablasts. The cells were analyzed with FACS. The plots of surface CD19 and intracellular IgA are shown. **B-E**. The absolute cell counts from the experiments described in Figure 6 are shown in **B**; representative CD27/CD38 plots of live cells are shown in **C**, Intracellular IgM/IgA plots of $CD27^+$ cells are shown in **D**, and the MFI of intracellular IgM and IgA is shown in **E**. Raw percentages of the indicated gates from at least four experiments are shown in the bar graph of **C** and **D**. In **E**, the MFI of intracellular IgM and IgA derived from CD4 gRNA-transfected samples was arbitrarily set as 100%. The raw MFI of IgM ranges from 5308 to 10311 and IgA from 4188 to 37842.

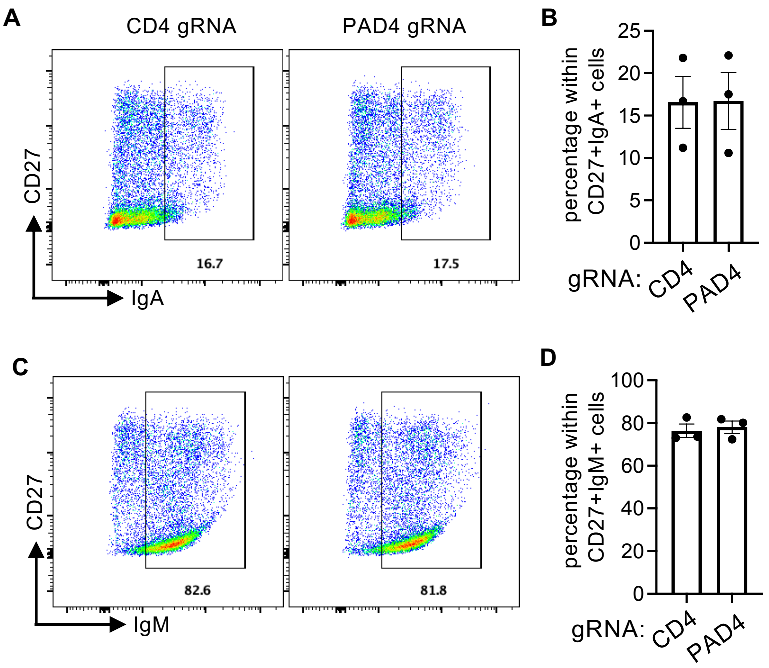

Supplemental Figure 6. Little impact of PAD4 ablation on the differentiation of plasmablasts. A-D. B cells purified from health donors were subjected to CRISPR with gRNA against CD4 or PAD4, and then differentiated into plasmablasts. Representative CD27/IgA and CD27/IgM plots of live cells are shown in **A** and **C**, respectively. The percentages of $CD27^+IgA^+$ and $CD27^+IgM^+$ cells from three independent experiments are shown in **B** and **D**, respectively.
